# Endogenous florendoviruses are major components of plant genomes and hallmarks of virus evolution

**DOI:** 10.1038/ncomms6269

**Published:** 2014-11-10

**Authors:** Andrew D. W. Geering, Florian Maumus, Dario Copetti, Nathalie Choisne, Derrick J. Zwickl, Matthias Zytnicki, Alistair R. McTaggart, Simone Scalabrin, Silvia Vezzulli, Rod A. Wing, Hadi Quesneville, Pierre-Yves Teycheney

**Affiliations:** 1 Queensland Alliance for Agriculture and Food Innovation, The University of Queensland, GPO Box 267, Brisbane, Queensland 4001, Australia; 2 INRA, UR1164 URGI, INRA de Versailles-Grignon, Route de Saint-Cyr, Versailles 78026, France; 3 Arizona Genomics Institute, School of Plant Sciences, BIO5 Institute, University of Arizona, Tucson, Arizona 85721, USA; 4 International Rice Research Institute, Genetic Resource Center, Los Baños, Laguna, The Philippines; 5 Department of Ecology and Evolutionary Biology, University of Arizona, Tucson, Arizona 85721, USA; 6 Istituto di Genomica Applicata, Parco Scientifico e Tecnologico di Udine Luigi Danieli, Via J Linussio 51, 33100 Udine, Italy; 7 Research and Innovation Centre, Fondazione Edmund Mach, Via E. Mach 1, 38010 San Michele all’Adige (TN), Italy; 8 CIRAD UMR AGAP, Station de Neufchâteau, Sainte-Marie, 97130 Capesterre Belle-Eau, Guadeloupe, France

## Abstract

The extent and importance of endogenous viral elements have been extensively described in animals but are much less well understood in plants. Here we describe a new genus of *Caulimoviridae* called ‘Florendovirus’, members of which have colonized the genomes of a large diversity of flowering plants, sometimes at very high copy numbers (>0.5% total genome content). The genome invasion of *Oryza* is dated to over 1.8 million years ago (MYA) but phylogeographic evidence points to an even older age of 20–34 MYA for this virus group. Some appear to have had a bipartite genome organization, a unique characteristic among viral retroelements. In *Vitis vinifera*, 9% of the endogenous florendovirus loci are located within introns and therefore may influence host gene expression. The frequent colocation of endogenous florendovirus loci with TA simple sequence repeats, which are associated with chromosome fragility, suggests sequence capture during repair of double-stranded DNA breaks.

## Abstract (web summary)

Endogenous viral elements have been extensively described in animals but their significance in plants is less well understood. Here, Geering *et al*. describe a new group of endogenous pararetroviruses, called florendoviruses, which have colonized the genomes of many important crop species.

## Graphical abstract

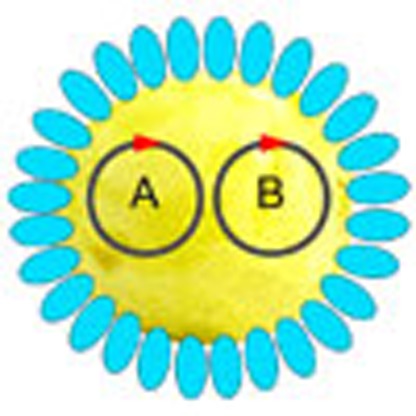

Horizontal gene transfer constitutes a significant lateral force in species evolution^1^. For multicellular eukaryotes, only DNA that is transferred into germline nuclei is able to be transmitted to the progeny. Most documented examples of horizontal gene transfer involve the transfer of DNA of either prokaryotic^2^ or viral^3^ origin. A range of endogenous viral elements (EVEs) originating from ancestral viruses with all combinations of genome (single-stranded (ss) and double-stranded (ds) DNA, ss and dsRNA, and positive and negative sense ssRNA) have been found in eukaryotic genomes^3^,^4^,^5^,^6^. Of these, only the retroviruses (family *Retroviridae*) have an active integration mechanism. For all other viruses, endogenization is thought to be accidental and may involve incorporation of viral DNA during non-homologous end-joining repair of dsDNA breaks in the chromosome or, alternatively, some hybrid mechanism involving the enzymatic machinery of retrotransposons^3^.

Although the integration of viral sequences into host genomes can induce deleterious mutations, EVEs can also have beneficial outcomes for the host. For example, retrovirus long terminal repeat (LTR) promoters in the human genome contribute to the gene regulatory network by acting as alternative promoters for mRNA transcription, as binding sites for transcription factors such as p53, Oct4 and Nanog, or as promoters of antisense or non-coding RNA^3^,^7^. The insertion of endogenous retroviral sequences near to or in non-coding parts of a gene can also modify gene expression as a result of local epigenetic remodelling, the insertion of polyadenylation signals and the generation of new splicing sites^8^,^9^,^10^,^11^. Some EVEs have also contributed proteins that have assumed new and sometimes vital roles in normal host physiology. For example, syncitin-A, which derives from an endogenous retrovirus *Env* gene, is essential to early development of the placenta in mammals^12^. Several endogenous retroviruses also contribute to viral-derived immunity against closely related exogenous viruses by producing proteins that block cell entry, disrupt virus replication or movement, or ameliorate disease symptoms^13^. In plants, the *gem* gene of the grass *Festuca pratensis*, which is linked to delayed leaf senescence (the ‘stay-green’ phenotype), has a partiviral origin^6^.

EVEs are also of interest to the scientific community because they are essentially fossils of viruses that existed in eons gone by and therefore offer unique insights into virus evolution and biogeography. For example, by screening for orthologous EVE loci in different selections of the wild banana *Musa balbisiana*, it has been possible to date the endogenization events (and therefore the minimum ages) of two extant badnavirus species, *Banana streak GF virus* and *Banana streak IM virus*, to *c*. 640,000 years ago^14^. In another example from the animal kingdom, the discovery of an endogenous lentivirus in a Madagascan prosimian has established that the absence of a dUTPase and the presence of *vpr* (and possibly *nef*) genes are not prerequisites for the infection of primate hosts^15^.

The *Caulimoviridae* (caulimovirids) is the only family of dsDNA viruses in the plant kingdom and members have an entirely episomal replication cycle^16^. Endogenous caulimovirid sequences have now been found in 27 species from 9 different plant families and derive from representatives of 4 out of 7 recognized genera, namely *Caulimovirus*, *Petuvirus*, *Badnavirus* and *Solendovirus*, as well as the tentative new genus Orendovirus^5^. The mechanism of integration is poorly understood; however, as nearly all endogenous caulimovirid sequences described so far are fragmented and rearranged when compared with the cognate ancestral viral genome, it is unlikely to be a coordinated process controlled by the virus, especially as there is no virus-encoded integrase enzyme. Furthermore, because the endogenization events are typically very ancient, many endogenous caulimovirid sequences show evidence of sequence decay rendering them replication defective^5^. However, there is strong evidence that some loci in *M. balbisiana*, *Petunia* × *hybrida* and *Nicotiana* × *edwardsonii* still contain replication-competent sequences, which can be activated to cause infection under certain environmental conditions in particular host genotypes, especially interspecific hybrids^5^. The contributions of endogenous caulimovirid sequences to plant genomes and the beneficial impacts (if any) that they could impart on plants are poorly understood, although a role in defending against infection by the cognate exogenous virus is a popular hypothesis^17^,^18^,^19^,^20^.

In this study, we build on the work of Bertsch *et al*.^17^, who described partial (*c*. 200–800 bp) caulimovirid-like sequences in the genomes of *Vitis vinifera* and *Populus trichocarpa*. We show that these sequences derive from representatives of a new genus of the *Caulimoviridae*, which we tentatively call ‘Florendovirus’, and are widespread in the genomes of cultivated and wild plant species in ANITA grade and mesangiosperm families. Our results show that there has been extensive colonization of the *Amborella trichopoda*, *Jatropha curcas*, *V. vinifera*, *Ricinus communis* and *Citrus* genomes by florendoviruses on a scale similar to that of some high copy number families of transposable elements (TEs). By searching for orthologous loci in the *Oryza* AA genome, we provide evidence that the florendovirus endogenization events took place at least 1.8 million year ago (MYA). We also provide evidence for the first time of the existence of reverse-transcribing viruses with a bipartite genome, illustrating the transition of virus genomes from simple to more complex organizations.

## Results

### Detection of novel EVEs in plant genomes

Endogenous caulimovirid sequences were initially identified in the *V. vinifera* genome using similarity searches and these were used to reconstruct complete virus genomes (Supplementary Data 1 and 2). These viral sequences were then used to search a variety of plant genomes and homologous sequences were identified in two out of six species in the Monocotyledoneae and 19 out of 25 species in the Eudicotyledonae, but were absent in the single species of the Magnoliidae that was examined (*Aquilegia caerulea*) (Table 1). Furthermore, homologous sequences were also found in the genome of *A. trichopoda* (family Amborellaceae), which belongs to the earliest group of flowering plants, termed the ANITA grade angiosperms^21^. When we extended our search to the GenBank expressed sequence tags database, we could identify a related EVE (GenBank accession FD385053.1) in another ANITA grade angiosperm, water lily (*Nuphar advena*, Nymphaeaceae). However, homologous sequences were not detected in the genomes of even more primitive plants such as the fern ally *Selaginella moellendorffii*, the moss *Physcomitrella patens* and the green algae *Chlamydomonas reinhardtii*. As these EVEs were found in flowering plants and form a novel lineage within the family *Caulimoviridae* (see below), they were named ‘Florendovirus’, after Flora, the Roman goddess of flowers (***Flor***a ***endo***genous ***virus***).

### Genome organization and classification

From all plant genomes examined, a total of 76 entire or nearly full-length florendovirus genomes of 7.2–8.5 kbp were assembled (Supplementary Data 3). Of these, 34 represented distinct species based on a 80% nt identity threshold in the reverse transcriptase (RT)-ribonuclease H1 (RH1) domains (the demarcation criterion for different species in the *Caulimoviridae*^16^) and the remainder, sequence clusters (equivalent to strain) of these species. Each species was given a name comprising the host species name, a letter of the alphabet if more than one virus species was present in a plant genome and finally the term virus.

**Figure 1:**
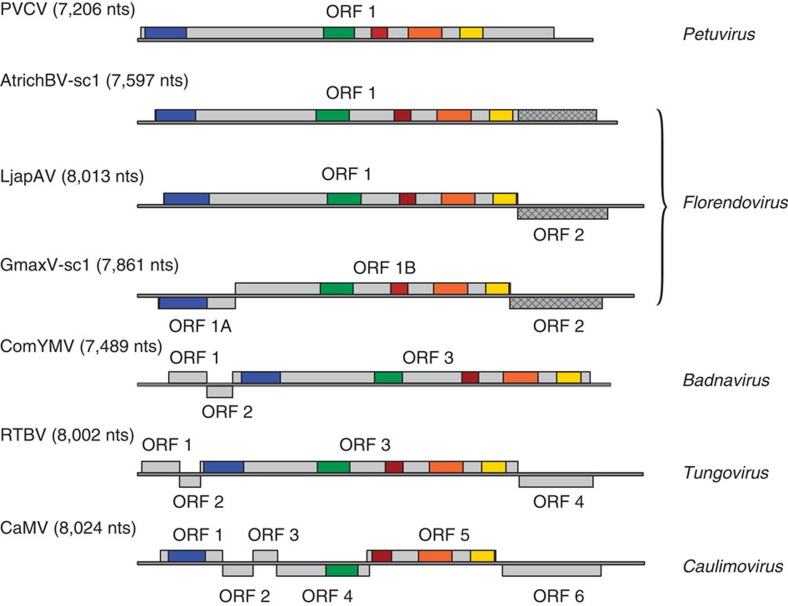
Florendovirus genome organizations as compared with other members of the *Caulimoviridae*. Schematic representation of the genomes of *Petunia vein clearing virus* (PVCV, type species of genus *Petuvirus*), Amborella trichopoda B virus sequence cluster 1 (AtrichBV-sc1), Lotus japonicus A virus (LjapAV), Glycine max virus sequence cluster 1 (GmaxV-sc1), *Commelina yellow mottle virus* (ComYMV, type species of genus *Badnavirus*), *Rice tungro bacilliform virus* (RTBV, type species of genus *Tungrovirus*) and *Cauliflower mosaic virus* (CaMV, type species of genus *Caulimovirus*). Genomes have been linearized and following convention, the first nucleotide of the tRNA^met^ consensus sequence designated the beginning of the genome. Light grey boxes mark open reading frames and coloured regions within ORFs are conserved protein domains: blue is the viral MP domain (PF01107); red is the retropepsin (pepsin-like AP) domain (CD00303); orange is the reverse transcriptase domain (CD01647); and yellow is the RNaseH1 domain (CD06222). In addition, a conserved CP domain, corresponding to L_261_–N_429_ of the CaMV ORF4 protein, is marked green. Diamond-patterned hatching marks ORF 2 of LjapAV and GmaxV-sc1 or a homologous domain in ORF 1 of AtrichBV.

The reconstructed genomes of the majority of florendoviruses contain two open reading frames (ORFs) in different translational frames (Supplementary Data 3) such as for Lotus japonicus A virus (Fig. 1). ORF1 encodes a putative 205–216 kDa polyprotein with movement protein (MP), coat protein (CP) with zinc finger, aspartic protease (AP), RT and RH1 domains (Supplementary Data 4). ORF2 encodes a putative 45–58 kDa protein that lacks significant homology to reference proteins and protein domains, and appears to be specific to the florendoviruses. Although the domains and their order is conserved, the genome organizations of Amborella trichopoda B virus and Glycine max virus differ from that of other florendoviruses; there is a single ORF in the former and three ORFs in the latter, caused by a division of ORF1 after the MP domain (Fig. 1). These atypical genome organizations are conserved between different sequence clusters of the viruses, suggesting that they are the true representations of the ancestral exogenous viral genomes.

**Figure 2:**
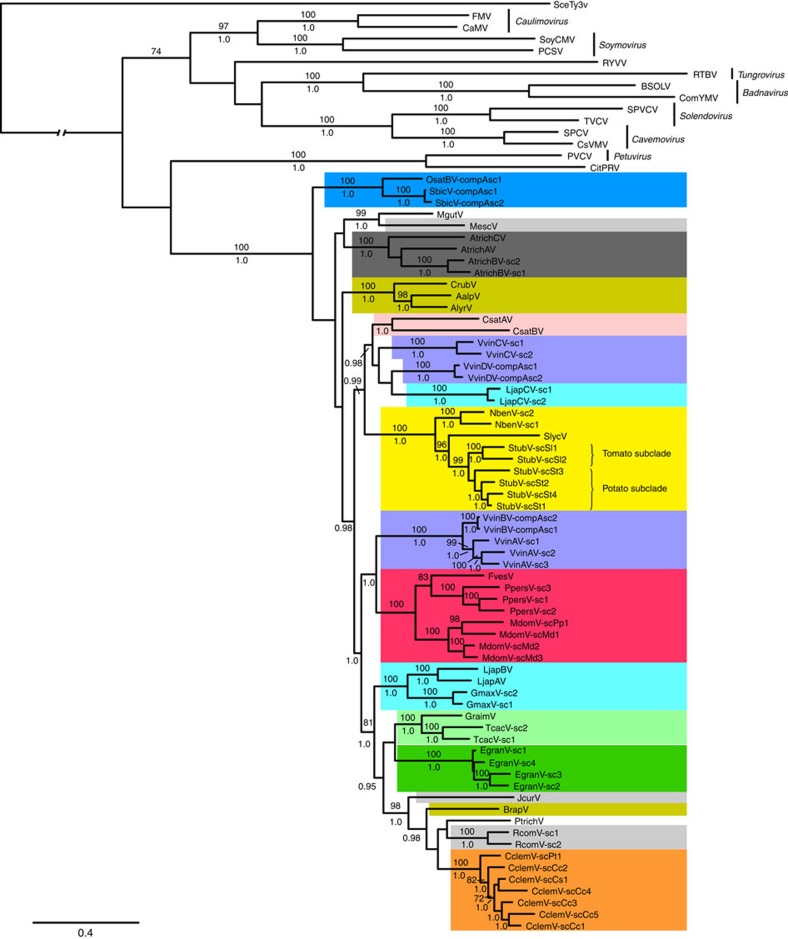
Phylogenetic relationships within the *Caulimoviridae*. Phylogram obtained from a maximum likelihood search with DNA sequence data from *AP*-*RT*-*RH1* genes. Bootstrap support (≥70%) values from 1,000 replicates above nodes. Posterior probabilities (≥0.95) summarized from 29,000 trees in a Bayesian search are shown below nodes. Virus species from each of the recognized genera are *Cauliflower mosaic virus* (CaMV), *Figwort mosaic virus* (FMV), *Soybean chlorotic mottle virus* (SoyCMV), *Peanut chlorotic streak virus* (PCSV), *Rice tungro bacilliform virus* (RTBV), *Commelina yellow mottle virus* (ComYMV), *Banana streak OL virus* (BSOLV), *Sweet potato vein clearing virus* (SPVCV), *Tobacco vein clearing virus* (TVCV), *Cassava vein mosaic virus* (CsVMV), *Sweet potato collusive virus* (SPCV), PVCV, Rose yellow vein virus (RYVV, unassigned) and Citrange pararetrovirus (CitPRV, unassigned). The outgroup is *Saccharomyces cerevisiae Ty3 virus* (SceTy3V). New florendovirus species are colour-coded to indicate the plant family in which they are found: dark blue is Poaceae, light grey is Euphorbiaceae, dark grey is Amborellaceae, olive green is Brassicaceae, pink is Cucurbitaceae, purple is Vitaceae, light blue is Fabaceae, red is Rosaceae, yellow is Solanaceae, light green is Malvaceae, dark grey is Myrtaceae and orange is Rutaceae. Scale bar, 0.4 nucleotide substitutions per site in the nucleotide alignment using the GTRGAMMA model of evolution.

Phylogenies inferred from conserved *AP-RT-RH1* gene sequences showed that all newly described florendovirus species formed a strongly supported, monophyletic clade within the *Caulimoviridae* (Fig. 2). The recently described caulimovirid from Carrizo citrange (*Citrus sinensis* × *Poncirus trifoliata*)^22^, which is thought to be endogenous, grouped apart from the florendoviruses and is much more closely related to *Petunia vein clearing virus* (PVCV), despite having a genome organization that superficially resembles that of the florendoviruses. Although most florendoviruses formed sub-clades that correlated with host plant family, some were sister to those from distantly related plant species (for example, Ricinus communis virus and Populus trichocarpa virus), suggesting a large host swap of the most recent common ancestor of these viruses.

### Evidence for bipartite viral genomes

**Figure 3:**
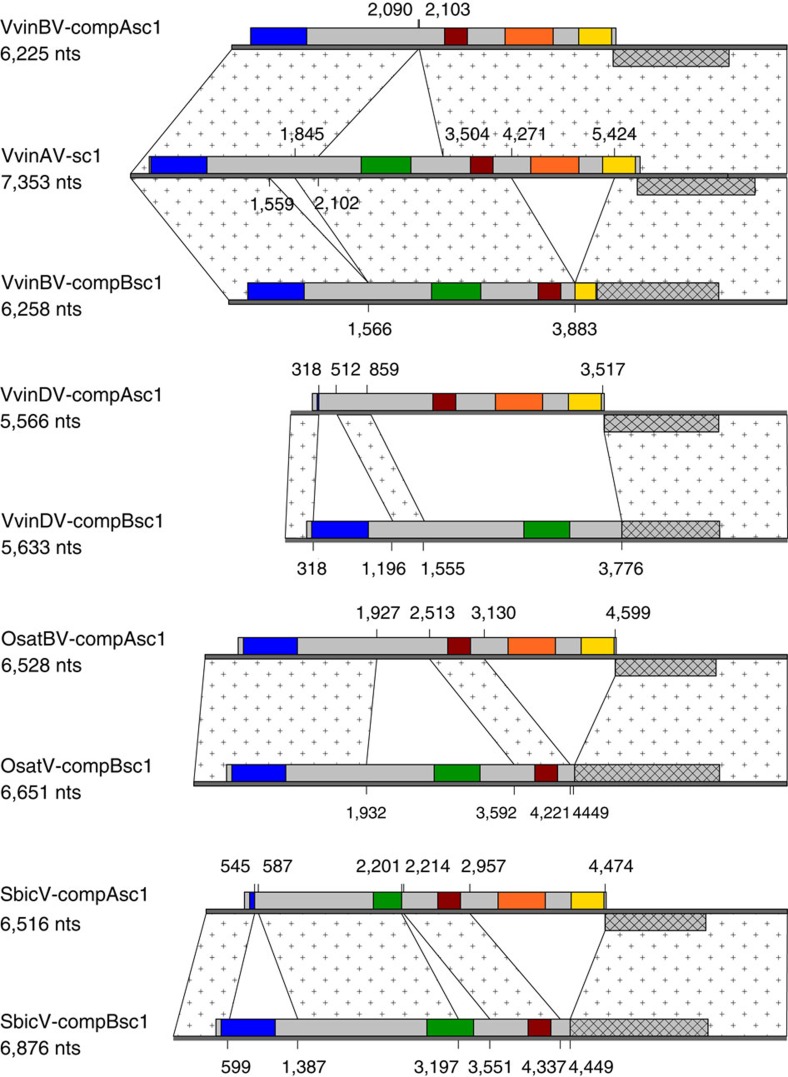
Structure of bipartite florendovirus genomes. Comparison of the genome organizations of Vitis vinifera A virus (VvinAV), Vitis vinifera B virus (VvinBV), Vitis vinifera D virus (VvinDV), Oryza sativa B virus (OsatBV) and Sorghum bicolor virus (SbicV). Genomes have been linearized and following convention, the first nucleotide of the tRNA^MET^ binding site designated the beginning of the genome. Light grey boxes mark ORFs and conserved domains within each ORF are coloured as for Fig. 1. Regions of sequence homology are represented by polygons containing crosses and the boundaries of these regions are labelled with numbers, which are the nucleotide positions in the virus genomes.

Several reconstructed EVEs in *V. vinifera*, *Oryza sativa* and *Sorghum bicolor* grouped within the florendovirus clade (Fig. 2) but are missing one or more protein domains that would theoretically be needed for completion of the replication cycle. These sequences are 5.5–6.9 kbp in length, have one or two ORFs and can be divided into two categories according to their general structure, henceforth referred to as components A and B. In *V. vinifera*, component A sequences have a typical florendovirus genome organization but the amino terminus of ORF1 is truncated because of a partial or complete deletion of the CP domain and sometimes also the MP and AP domains (Fig. 3). In contrast, component B sequences encode a single polyprotein with MP, CP and AP domains, but the RT and the N-terminal portion of the RH1 domain are missing. The component B polyprotein also presents a carboxy-terminal extension that is homologous to the ORF2 protein of component A (Fig. 3). In *O. sativa* and *S. bicolor*, component A and B sequences have the same overall structures as in *V. vinifera* albeit the ORF2 homologues are more divergent. Hence, components A and B appear to encode complementary (and partially redundant) sets of proteins that together constitute complete florendovirus proteomes. The virus from *Oryza* was called Oryza sativa B virus (OsatBV) to distinguish it from previously described endogenous caulimovirids in rice^23^.

Although component B sequences lack an RT domain, conspecificity with selected component A sequences could be established on the basis of very high sequence similarities within the intergenic region (>90% nt identity) and MP domain (>87% nt identity; Supplementary Table 1). Together, domain complementarity and sequence similarity suggest that these two sets of sequences are co-evolving entities of a bipartite viral genome. Lending support to this hypothesis, we detected several loci in the *V. vinifera* and *Oryza* genomes where components A and B form compound insertions (Supplementary Fig. 1), suggesting coexistence and probably interaction of the two DNA genomes at the time of capture in the chromosome. Evidence that these are not artefacts generated by the sequence assembly process is provided by the large number of entire or nearly entire component sequences that are present in each plant genome (Supplementary Table 2 and Supplementary Data 5). In addition, the flanking regions of the component sequences show very little redundancy, indicating that segmental duplication or transduplication do not explain the multiplicity of these sequences in *V. vinifera* (Supplementary Table 3), thereby suggesting that the majority of the copies result from independent integration events. Two virus species with putative bipartite genome organizations are present in *V. vinifera*, namely Vitis vinifera B virus (VvinBV) and Vitis vinifera D virus (VvinDV) and one each in the monocot species. Although OsatBV and Sorghum bicolor virus share a most recent common ancestor, VvinBV and VvinDV represent parallel evolution events (Fig. 2).

### Dating the genome invasion of *Oryza*

**Figure 4:**
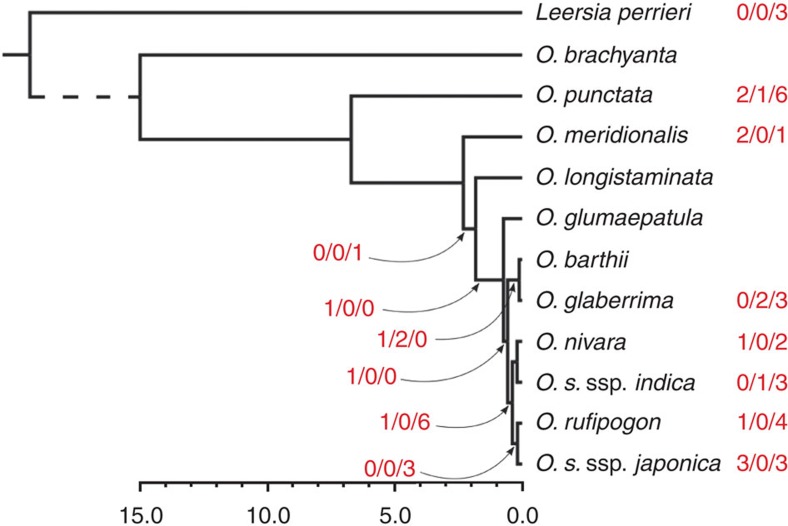
Placement of 54 Oryza sativa B virus (OsatBV) loci on the *Oryza* phylogenetic tree. OsatBV insertions were searched in orthologous loci across all 12 *Oryza* species and placed onto the phylogenetic tree according to the most parsimonious hypothesis. The red numbers represent insertions of A, a mixture of A and B, or B components, respectively. Shared insertions are indicated by arrows pointing at the corresponding branch. Because of the method adopted, the split of the outgroup *Leersia perrieri* could not be dated (dashed line), while the split of *O. brachyantha* was fixed to 15 million years ago (MYA). Other node ages are: *O. punctata* (BB genome), 6.712 MYA; all AA genome species, 2.317 MYA; *O. longistaminata*, 1.832 MYA; *O. glumaepatula*, 0.738 MYA; Asian-African AA species, 0.572 MYA; Asian species, 0.391 MYA; *O. japonica* ssp. *indica*-*O. nivara*, 0.202 MYA; *O. sativa* ssp. *japonica*-*O. rufipogon*, 0.187 MYA; *O. glaberrima*-*O. barthii*, 0.120 MYA. The scale bar represents time, with increments of one million years, and labels every five million years.

We took advantage of the availability of several closely related *Oryza* genomes (Supplementary Table 4) to date the OsatBV endogenization events by searching for orthologous OsatBV loci in the different species. Of the nine *Oryza* AA genomes and the one *Oryza* BB genome (*Oryza punctata*) that were examined, OsatBV (>99% nt identity to the OsatBV consensus) was found in all, except in *Oryza brachyantha* (Table 2). Related sequences were also found in *Leersia perrieri* albeit these were significantly different to those in *Oryza* (80–88% nt identity to the OsatBV consensus). Collectively, a total of 54 different OsatBV loci were identified, of which 13 loci contained A component sequences, 35 loci contained B component sequences and the remaining 6 loci contained a mixture of the two (Supplementary Data 6). Out of 16 loci shared by 2 or more assemblies, only 2 (japo_1_23M and japo_7_27M) had a pattern that was inconsistent with the phylogenetic tree of the species (Fig. 4), and can be explained by incomplete lineage sorting at these loci. Interestingly, one OsatBV locus was shared by all AA-genome types, except *Oryza meridionalis*, which is the basal lineage of this genome type. The seven *Oryza* species containing the OsatBV orthologues are distributed across a wide geographical area including Asia, western and sub-Saharan Africa, Madagascar, and Central and South America, suggesting that introgression of the shared OsatBV loci by interspecific hybridization is very unlikely given the considerable geographic barriers. Based on the estimated time of divergence of *O. meridionalis* from all other AA genome taxa (Fig. 4), these OsatBV insertions have occurred between 1.8 and 2.3 MYA.

When looking for evidence of recent OsatBV insertions, we found an interesting instance where a polymorphic locus between *Oryza glaberrima* (Chr10:8390000..8409999) and *Oryza barthii* (Chr10:8419000..8422000) shows an insertion flanked by (TA)n repeats in the former and the presence of an empty stretch of TA repeats in the latter. Unless this insertion was precisely eliminated in *O. barthii*, it is likely to be that this polymorphism reflects the endogenization of OsatBV after the divergence of the two species about 120,000 years ago (Fig. 4).

### Abundance and distribution in plant genomes

The reconstructed florendovirus genome sequences identified here (Supplementary Data 1) were used to mask plant genomes and we observed highly heterogeneous florendovirus sequence abundance. In *Arabidopsis thaliana*, only putative traces (*c*. 3.4 kb) of sequence were detected (Table 1). In contrast, florendovirus sequences make up >1% of the *R. communis* genome and >0.5% of the *J. curcas*, *A. trichopoda*, *Citrus clementina* and *V. vinifera* genomes. The same method was used to mask the various *Oryza* genomes, but this time only using the OsatBV consensus sequences (Table 2). Overall, the OsatBV contribution to the *Oryza* genomes is relatively modest (≤0.04% of total genome content) and also highly variable between the different species, which may reflect true differences in the copy number but also could be significantly influenced by the quality of the genome assemblies. For reasons that are unclear, component B sequences were overall, twofold more abundant than component A sequences.

**Figure 5:**
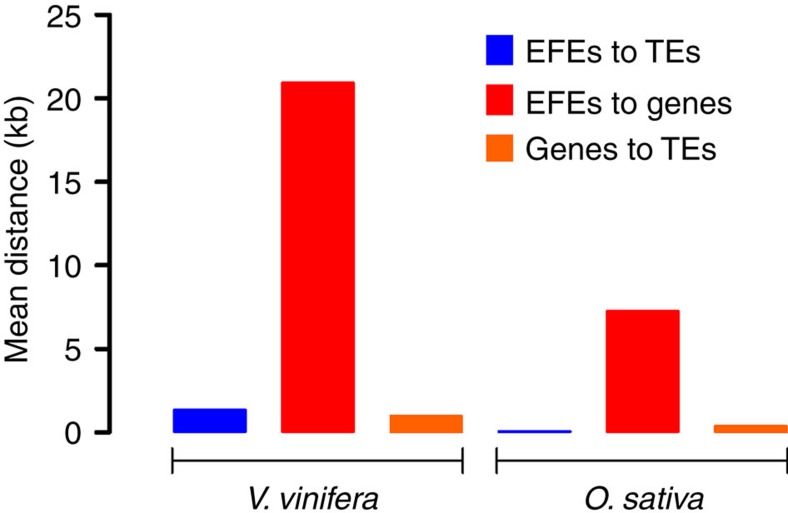
Distances between endogenous florendovirus elements (EFEs) and other plant genome features. The mean nucleotide distances that separate EFEs from either transposable elements (TEs) or genes in *Vitis vinifera* cv. Pinot Noir and *Oryza sativa* are shown.

To determine whether there was an association between florendovirus sequences and any other genome feature, we focused on the reference genomes of *V. vinifera* and *O. sativa*, which are assembled into pseudo-chromosomes. For both species, we found that florendovirus sequences are on average located much closer to TEs than to genes (Fig. 5). For *V. vinifera*, which is florendovirus-rich compared with *O. sativa*, *c*. 9% of the loci overlap with host genes, with 99% (*c*. 286 kbp) of these located within introns. To assess whether florendoviral promoters would have been selected for the transcriptional regulation of host genes, we also investigated whether they are frequent in the proximity of genes but we could not establish such a correlation.

**Figure 6:**
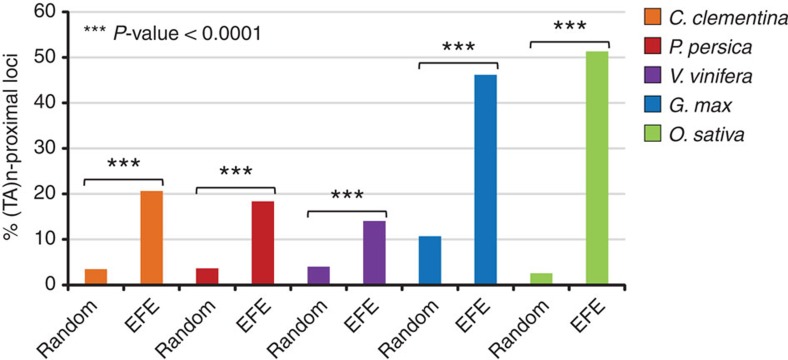
Physical concomitance of endogenous florendovirus elements (EFEs) and TA dinucleotide ((TA)n) repeats. The percentages of EFE and equal numbers of random loci that are located at less than 1 kbp from (TA)n repeats are shown. TA(n) repeats were detected with Tandem Repeat Finder. Loci sample sizes (*n*) were: *Citrus clementina* (*n*=543), *Prunus persica* (*n*=136), *Vitis vinifera* (*n*=968), *Glycine max* (*n*=468) and *Oryza sativa* (*n*=39). The statistical significance of differences in the frequency of association of EFE and random loci to (TA)n repeats was determined using a Mann–Whitney *U*-test.

Manual examination of various plant genomes led to the observation that florendovirus sequences were frequently flanked by TA dinucleotide simple sequence repeats (TA(n)). The existence of the simple sequence repeat before the integration was confirmed by inspecting several orthologous loci of related *Oryza* species that present an empty site. This analysis also revealed that as a result of the insertion, short stretches of sequence can be gained or lost (Supplementary Fig. 2). To quantify the sequence associations, we examined a subset of large (≥500 bp) endogenous florendovirus loci in five different plant genomes (Fig. 6) and found that (TA)n-proximal loci are significantly (*P*≤0.0001) more frequent than expected by chance in each species addressed. The proportion of (TA)n-proximal loci ranged from 14% in *V. vinifera* to 46% and 51% in *G. max* and *O. sativa*, respectively. Interestingly, an integration bias of the rice tungro bacilliform virus-like sequences towards (TA)n repeats within the *Oryza* genome has already been described by Kunii *et al*.^24^ Here, our results suggest that this repeated motif is present before insertion of the DNA in the *Oryza* genome and the broader association of florendoviral sequences with TA stretches supposes a similar situation in a variety of plant species.

### Evidence for replication competency

In *V. vinifera*, the sequences of endogenous Vitis vinifera A virus and Vitis vinifera C virus have decayed to a point that no loci containing an entire viral genome could be identified, nor even a fragment of sequence containing an uninterrupted ORF. In contrast, endogenous VvinBV is much more likely to be replication competent, as many loci contain entire component sequences and several also had uninterrupted ORFs (Supplementary Table 2 and Supplementary Data 5). The mean fragment length of endogenous VvinBV was also about double that of VvinAV (Supplementary Table 2). In a recent study addressing genetic diversity among four phenotypically different somaclonal variants of *V. vinifera* cv. Pinot Noir, insertional polymorphisms of a sequence called Cauliv-1 were observed^25^. Cauliv-1 was classified as a class I LTR TE by the authors but sequence comparisons by us show that it is the same as VvinBV. The domestication of grapevine probably began during the Neolithic era (6,000 to 5,000 B.C.)^26^, suggesting that VvinBV insertions and/or deletions occurred very recently on an evolutionary timescale.

We detected a *c*. 42-kbp region in *Prunus persica* containing 5.36 copies of the Prunus persica virus sequence cluster 1 (PpersV-sc1) genome, including one showing uninterrupted ORFs (Supplementary Data 5). Another *P. persica* locus contains a 1.2-mer of PpersV-sc1, which begins at the start of ORF2, continues to a tRNA^MET^ consensus sequence and is then followed by another entire, uninterrupted copy of the genome. Loci with greater than unit length florendovirus genomes and uninterrupted ORFs are also present in the nuclear genomes of *Malus* × *domestica* and *Glycine max*. For any of the aforementioned loci, an exogenous copy of the genome could potentially be released from the chromosome by either intrastrand homologous recombination or by transcription from the viral promoter in the first copy of the intergenic region.

Interestingly, florendovirus sequences are well-represented in expressed sequence tag (EST) databases (when available), indicating that they are often transcribed, which is an important prerequisite for replication. For example, ESTs covering 53% of the Citrus clementina virus sequence cluster 2 genome and 47% of the PpersV-sc1 genome were identified in libraries prepared from globular embryo tissue of *C. clementina* and from shoots, leaves and fruits of *P. persica*, respectively (Supplementary Fig. 3). In addition, inspection of assembled *O. sativa* RNA-Seq data showed that transcripts aligned to 57% of the entire OsatBV component A, while the whole of component B was transcribed, even though unevenly.

### Endogenous florendoviruses as sources of small RNAs

In general, EVEs are sources of small RNAs (sRNAs, 21–24 nt) that could be involved in antiviral defense mechanisms or play a role in shaping the epigenome^8^,^27^. We searched for sRNAs with zero mismatches to the reconstructed florendovirus genome sequences and found corresponding molecules in complementary DNA libraries from *A. trichopoda*, *C*. *clementina*, *C. sinensis*, *P. trichocarpa*, *Solanum lycopersicum*, *Solanum tuberosum*, *S. bicolor* and *V. vinifera*. Estimates of the number of sRNAs are probably greatly underestimated, as the reconstructed florendovirus genome sequences are consensus sequences and therefore do not reflect the full extent of sequence variation at different loci. Although there were some hotspots within the viral genomes from where the sRNAs derived, no consistent patterns could be ascertained (Supplementary Fig. 4).

## Discussion

We have reconstructed representative genomes of a new genus of the *Caulimoviridae*, tentatively named ‘Florendovirus’, from fragments of sequence that have been captured and preserved in plant genomes. A premise of this type of analysis is that following endogenization, the rate of evolution of the sequences greatly slows down, and because selective constraints on the viral sequence are removed those mutations that do occur are random and are eliminated on generation of a consensus sequence. A similar analytical approach has been successfully used to reconstruct the ancestral sequences of a range of TEs and is considered to give a good approximation of the ancestral sequence as long as the endogenous sequences are not so old as to be unrecognizable from the ancestral sequence and that they exist in a sufficiently high copy number to allow determination of a ‘modal’ sequence^28^,^29^. One very remarkable feature of the florendoviruses is the extraordinary diversity of host plants (ANITA grade, monocots and dicots), and at a discovery rate of >50% in the plant genomes that were examined, many additional florendovirus species are still likely to be discovered. From this study alone, the diversity of florendoviruses is greater than any other extant genera of the *Caulimoviridae* except the badnaviruses^16^.

Phylogenetic analyses showed that the proposed genus Florendovirus is sister to PVCV, the type and sole member of the genus *Petuvirus*, with which it shares the plesiomorphic trait of MP, CP, AP, RT and RH1 precursors occurring in one large polyprotein. This polyprotein is presumably processed by the virus into the mature proteins through the action of the virus-encoded AP^30^. The florendoviruses are readily distinguished from PVCV by the presence of a second ORF, which encodes a putative protein of unknown function with no homologue in any other caulimovirid. For the majority of species, ORF2 was in a different translational reading frame to ORF1, suggesting a mechanism of translation using occasional leaky ribosome scanning^31^. Interestingly, the atypical genome organization observed for Glycine max virus with split ORF1 is similar to the situation of ORF3 from the badnavirus *Sweet potato pakakuy virus*^32^. This additional division of the virus genome may allow more precise control of expression of the structural and enzymatic proteins during different parts of the replication cycle as compared with post-translational processing of a large polyprotein.

Bipartite florendovirus genomes represent a unique genome organization for viral retroelements. Retroviruses encapsidate two identical or nearly identical RNA molecules and therefore their genomes are diploid rather than bipartite^33^. One cannot discount the possibility that the putative bipartite florendoviruses depended on a helper virus for replication, although this would appear unnecessary as when viewed together, components A and B are complementary and contribute all gene-regulatory and protein-coding sequences necessary for replication. Importantly, each component contains a complete intergenic region, which is nearly identical between component A and B sequences. Interestingly, the florendovirus bipartite genome structure evolved on three independent occasions with a remarkably similar outcome in terms of gene organization. However, there are no examples of divided genomes in extant members of the *Caulimoviridae*, although it is commonplace in plant RNA viruses^16^. Complementation between cauliflower mosaic virus (CaMV) and a CaMV-derived virus vector with a foreign marker gene has been observed^34^, providing evidence that complementation between two different genome components of a caulimovirid is at least experimentally possible. Bipartite florendovirus genomes may therefore represent unsuccessful attempts in the evolution process of viral retroelements.

Analyses of the patterns of integration suggest that florendovirus sequences are more likely to be found in TE-rich regions of the plant genome and there is also a strong bias towards insertion in TA dinucleotide simple sequence repeats. The co-location of TEs and florendovirus sequences may simply reflect similar selection pressures acting to determine where in the genome these elements accumulate, as insertions in gene-rich regions are more likely to be deleterious to the individual and therefore the insertion less likely to persist in the population due to selection pressure^35^. Insertion in stretches of TA dinucleotides may, however, point to the mechanism of integration. It is thought that TA dinucleotide-rich areas of sequence are more likely to form highly stable secondary structures (for example, hairpins) that perturb DNA replication, thereby causing chromosome fragility^36^,^37^. Florendovirus DNA could then be coopted to act as filler DNA to repair the double-stranded DNA breaks by either non-homologous end joining or microhomology-mediated end joining^38^.

A minimum age of at least 1.8 million years has been provided for endogenous OsatBV, which is approximately three times older than the only other endogenous caulimovirids that have been dated, *Banana streak GF virus* and *Banana streak IM virus*^14^. The dating technique that was used does have its intrinsic limitations, as the turnover of repetitive elements in plants is relatively rapid. For example, in *Nicotiana* spp., there is near-complete genome turnover of repetitive elements in as little as five million years^39^, and for *O. sativa*, the half-life of LTR retrotransposons is less than six million years^40^. The phylogenetic relationships that were observed suggest a much older age of the florendoviruses. For instance, the florendoviruses in *Eucalyptus*, an iconic Australian plant genus in the Gondwanan family Myrtaceae, were sister to those in *Theobroma cacao* and *Gossypium raimondii*, both of which originate from South America. The discovery of *Eucalyptus* macrofossils in southern Argentina suggests that this plant genus was continuously distributed across the Antarctic land bridge between Australia and South America^41^. This floristic connection was broken about 34 MYA when the Drake Passage opened and permanent ice sheets formed in Antarctica^42^,^43^. The geographic distribution of closely related endogenous florendoviruses in *Eucalyptus grandis*, *T. cacao* and *G. raimondii* can be explained by either vicariance or long-distance dispersal of the most recent common ancestor of the viruses across the Pacific Ocean, the largest stretch of water in the world. We consider the first hypothesis much more probable, giving a minimum age of 34 million years for this virus clade. The florendoviruses from *Nicotiana benthamiana*, *S. lycopersicum* and *S. tuberosum* also formed a monophyletic clade. *N. benthamiana* is a member of *Nicotiana* section *Suaveolentes*, a section of this genus largely endemic to Australia but with South American ancestors, while *S. lycopersicum* and *S. tuberosum* originate from South America^44^. The most recent common ancestor of the Australian representatives of section *Suaveolentes* is thought to have colonized Australia at least 20 MYA^45^, which could be also considered a minimum age for this virus clade.

Definitive conclusions about the replication competency of any endogenous florendoviruses described in this study cannot be made, although the discovery of polymorphic loci in closely related *Oryza* species suggests that there were cycles of infection and endogenization of OsatBV as little as 100,000 years ago. Given that endogenous florendoviruses occur in some of the most intensively studied crops (for example, grape, rice, cotton, soybean, maize, peach, strawberry, potato and tomato), one would assume that if they still existed in an exogenous form today, then they would have been discovered in more than a century of plant virology research, even if serendipitously as a contaminant in other virus preparations. Given the long association of florendoviruses with their plant hosts, it is possible that as a consequence of coevolution, the disease symptoms caused by the viruses have attenuated to a point that they no longer cause harm to the plants and therefore have not received the attention of plant pathologists. Alternatively, endogenization may have provided plant immunity to infection by the cognate exogenous virus through induction of RNA interference pathways^17^,^19^, causing the viruses to become extinct. Finally, perhaps the florendoviruses (and members of the *Caulimoviridae* in general) flourished in prehistoric times when a particular vector group was abundant, but this vector group has now disappeared or greatly diminished in abundance due to environmental changes or domestication of the host plant species by humans. Supporting this hypothesis, PVCV, the nearest relative of the florendoviruses, has only ever been graft-transmitted and attempts at mechanical or vector transmission have been unsuccessful^46^. Many other extant members of the *Caulimoviridae* also do not have any known insect vectors, such as some caulimoviruses, all soymo-, cavemo- and solendoviruses^16^, and Rose yellow vein virus^47^. Furthermore, *Rice tungro bacilliform virus* is only transmitted by leafhoppers when present in a mixed infection with a non-related helper virus, and caulimoviruses have only acquired the ability to be transmitted by aphids, the most common vector group nowadays, through the acquisition of a novel auxiliary gene, the aphid transmission factor^16^.

The question remains as to what beneficial functions endogenous florendoviruses could confer on the plant. Plant defence against virus infection is one possible benefit but it is questionable whether this is the current function for several reasons. First, rather than preventing infection, modern experience with related badnaviruses, petuviruses and solendoviruses suggests that some endogenous forms of these viruses are paradoxically the major source of infection and when an exogenous viral genome is released, it is able to overcome RNA interference-induced resistance, perhaps by the expression of a silencing suppressor protein. Second, the copy number of most endogenous caulimovirid sequences in plant genomes (for example, this study, Jakowitsch *et al*.^48^ and Kunii *et al*.^24^) is far in excess of that which is needed to provide efficient silencing of a virus: one hairpin transgene containing sense/anti-sense arms that are as short as 98 nts is capable of providing efficient silencing^49^. Finally, endogenous florendoviruses are widespread in the plant kingdom, and if the cognate exogenous viruses are in fact either very rare or have become extinct, a role in plant defence is somewhat redundant.

It would seem more likely that the endogenous florendoviruses are contributing to plant evolution by acting as sources of novel genetic material at either the coding or transcription regulatory levels. A feature of plants that distinguishes them from animals is their highly plastic genome structure: angiosperm genome sizes vary nearly 2,000-fold compared with those of mammals and birds, whose genome sizes vary by no more than 5-fold^50^. It is theorized that this genome plasticity has allowed plants to acquire new biochemical processes or growth patterns in relatively short time frames to adapt to new predation or competition pressures or variable climatic conditions in an unstable environment. It is noteworthy that 9% of the endogenous florendovirus loci in *V. vinifera* are located within plant genes, and of these almost all are present within the introns. The presence of florendovirus sequences in introns possibly has biological consequences by affecting both the structure of the gene transcript as well as the level of its expression^10^. Overall, florendoviruses appear to have significantly contributed to the evolution of angiosperm genomes and perhaps to the emergence of phenotypes that have been domesticated such as in grape somaclonal variants.

## Methods

### Discovery and assembly of endogenous viral genomes

Uncharacterized EVEs in the *V. vinifera* genome were initially identified using the CaMV *AP-RT-RH1* (GenBank Accession NP_056728) as the query sequence in a tBLASTN search of the non-redundant nucleotide database of GenBank. High-scoring sequences were then extended by pairwise BLASTN comparisons of different loci containing identical or near-identical sequences. Fragments of virus sequence were assembled using VECTOR NTI Advance 10.3.1 (Invitrogen) operated using default settings, except that the values for maximum clearance for error rate and maximum gap length were increased to 500 and 200, respectively. Following convention for the *Caulimoviridae*, the first nucleotide of the tRNA^MET^ consensus sequence was designated the beginning of the viral genome and, accordingly, the preceding nucleotide the end of the genome. Once the first viral genomes were assembled, these in turn were used to search for similar sequences in other plant genomes including those available on the NCBI Genomes (chromosomes) and Whole-genome Shotgun Reads databases, Phytozome release v7.0 (www.phytozome.net), the peach genome v1 (http://www.rosaceae.org/peach/genome), the strawberry genome v1 (http://www.rosaceae.org/projects/strawberry_genome), the Jatropha Genome DataBase (http://www.kazusa.or.jp/jatropha/) and the Amborella Genome Database (http://www.amborella.org/). Accession numbers of sequences used in the analyses and further details of the databases are provided in Supplementary Table 1. Florendovirus genomes were screened for the homology with known protein domains using the InterPro^51^ and CDD^52^ databases.

### Phylogenetic analyses

To investigate evolutionary relationships, *AP-RT-RH1* gene sequences (sequences homologous to nts 3,732–5,650 of CaMV, NCBI Accession NC_001497.1) were used. DNA sequences of representatives of each of the genera in the *Caulimoviridae*, as well as the florendovirus consensus sequences that had uninterrupted reading frames, were conceptually translated and aligned using the MUSCLE algorithm in the MEGA v. 5.05 software package^53^, then back-translated into the nucleotide code. Florendovirus consensus sequences with interrupted reading frames were then added to this alignment using the ‘realign selected sequences’ option of CLUSTALX. Ambiguous regions of the alignment were removed using the Gblocks programme^54^ on the Phylogeny.fr server (available at http://www.phylogeny.fr/). The four domains (AP, RT, tether region and RH1) were analysed together as partitioned loci in the phylogenetic analyses.

Two phylogenetic assessment criteria were implemented: Bayesian inference using MrBayes 3 (ref. 55) and maximum likelihood using RAxML^56^. Resulting trees were observed with FigTree (http://tree.bio.ed.ac.uk/software/figtree/). The RAxML analyses were run with a rapid bootstrap analysis (command -f a) under GTRGAMMA using a random starting tree and 1,000 maximum likelihood bootstrap replicates. MrBayes 3 was used to conduct a Markov Chain Monte Carlo search with Bayesian inference. Four runs, each consisting of four chains, were implemented until the s.d. of split frequencies was below 0.01. The cold chain was heated at a temperature of 0.25. Substitution model parameters were sampled every 100 generations and trees were saved every 5,000 generations. Convergence of the Bayesian analysis was examined using the cumulative and compare analyses in AWTY^57^.

To calculate pairwise uncorrected nucleotide distances, MEGA v. 5.05 was used, choosing the pairwise deletion of gaps option^53^.

### Genome analyses

The contribution of the endogenous florendovirus sequences to plant genomes was calculated with RepeatMasker (http://www.repeatmasker.org) using the sequences listed in Supplementary Data 1 as library. RepeatMasker was run with a cutoff value=250 and a maximum divergence value=20. These parameters were chosen because they enabled discrimination between florendoviruses and closely related sequences such as *Gypsy* LTR retrotransposons and other endogenous caulimovirids, especially in highly conserved domains such as the RT. Only hits with a length >200 bp were counted in genome coverage calculations. RepeatMasker was also used with similar parameters to search OsatBV sequences in 12 genome assemblies (Supplementary Table 4) and the results were manually inspected to determine features of the insertions.

To calculate the distance of florendovirus sequences to TEs and genes in *O. sativa* and *V. vinifera*, repetitive sequences in each genome were first identified using RepeatScout^58^ with a ‘stopafter’ parameter set at 500. Both RepeatScout libraries were compared with the florendovirus genome sequences using BLAST with maximum *e*-value of 1*e*^−10^ and matching sequences were discarded. The filtered libraries were used to run RepeatMasker with default settings on the *V. vinifera* and *O. sativa* genomes, resulting in coverage of *c*. 53% and 46%, respectively. RepeatMasker hits for repeats and florendovirus were respectively clustered when the distance between two annotations was <200 bp and the position of the resulting clusters were used to determine the distances separating different features.

The TEannot pipeline^59^ available in the REPET package (http://urgi.versailles.inra.fr/index.php/urgi/Tools/REPET) was used to determine the integrity of florendovirus sequences in *V. vinifera*. After masking plant genomes using the florendovirus sequences, REPET was used to process nested sequences using a ‘long join procedure’, which connects two parts of one endogenous viral sequence interrupted by other endogenous viral sequences that have been inserted more recently.

To investigate whether there was commonality in the sequences that flanked the florendovirus sequences in *V. vinifera*, the loci were first defined by joining positions from the RepeatMasker annotation that were <1 kbp distant from one another. The 500-bp stretches of sequence that flanked the largest (≥1 kbp) and most probably the youngest loci were then extracted and clustered into groups using either an 80% or 90% nucleotide identity threshold. As a positive control for this analysis, the internal section (everything but the LTRs) of the *Gret1* LTR retrotransposon was analysed in the same way.

To investigate whether florendovirus sequences are closer than expected to TA simple sequence repeats, Tandem Repeat Finder^60^ was used in different plant genomes with the following set of parameters to detect TA microsatellites: 2,7,7,80,10,50,500. After joining the positions of the florendovirus RepeatMasker annotations that were <20 bp distant from one another, the number of loci >500 bp in each genome was counted and a random distribution of a similar number of 500 bp loci generated using the BEDTools suite (http://bedtools.readthedocs.org/en/latest/content/bedtools-suite.html). For each species, the number of endogenous florendovirus loci and the number of random annotations that are located at <1 kbp from a stretch of TA dinucleotides were then counted.

### Estimation of *Oryza* divergence times

Divergence times within *Oryza* were estimated on a phylogeny inferred using protein-coding genes from assemblies of the short arm of chromosome 3 (Supplementary Table 4). Sequences from 16 *Oryza* accessions were included, with *L. perrieri* serving as the outgroup (Zwickl *et al*.^61^ and Supplementary Table 4). Single-cop syntenic orthologue clusters were collected using the BLAST-Overlap-Synteny pipeline detailed in Zwickl *et al*.^61^. Full gene sequences (including introns) of each locus were aligned using PRANK v.140110 (ref. 62) using the -F setting. All individual alignments containing all 17 taxa (*n*=187) were concatenated into a single supermatrix (2,055,035 bp). A maximum likelihood phylogeny was inferred from the supermatrix with GARLI version 2.01 (ref. 63). A partitioned model was used that allowed each locus an independent substitution rate, while all loci shared a single general time-reversible nucleotide substitution model with gamma-distributed rate heterogeneity.

The maximum likelihood phylogeny inferred by GARLI was rooted using the outgroup *L. perrieri*, which was subsequently pruned from the tree. The tree of 16 *Oryza* taxa, with the maximum-likelihood branch length estimates obtained from GARLI, was input to PATHd8 v1.0 (ref. 64). To time calibrate the phylogeny, the divergence time of the genus *Oryza* (the root of the tree) was fixed for the PATHd8 analyses, using a divergence time consistent with several recent studies (15 MYA^65^,^66^,^67^). A time-calibrated ultrametric tree was output by PATHd8. The resulting dated phylogeny was pruned down to the taxon set of interest in this study.

### RNA transcript and sRNA analyses

Strand-specific RNA-Seq reads from three organs (leaf, root and mixed stage panicle) across 14 *Oryza* species and *L. perrieri* were assembled with Trinity^68^ to obtain a collection of assembled transcripts. These sequences were aligned to the two OsatBV component genomes using Bowtie 2 (ref. 69).

Predictions of promoter elements in the florendovirus pregenomic RNA were made by submitting the region of sequence spanning the end of ORF2 and the tRNA^MET^ consensus sequence to analysis using the BDGP Neural Network Promoter Prediction Web site (http://www.fruitfly.org/seq_tools/promoter.html).

sRNAs (21–24 nt) of florendovirus origin were searched in different tissue types (leaves, flowers, fruits, stolons, xylem) using data and tools provided by the Comparative Sequencing of Plant Small RNAs Web site (http://smallrna.udel.edu). Reads matching florendovirus sequences with no mismatch were mapped on reconstituted viral genomes using Mosaik version 1.1.0021 (http://code.google.com/p/mosaik-aligner/). Density plot and cartography of the reads on viral genomes were generated using S-MART version 1.16 (ref. 70).

## Author contributions

A.D.W.G. and F.M. independently discovered the endogenous florendoviral elements, both assisted with assembling the virus genomes and are equal first authors. M.Z. and P.-Y.T. did the sRNA analyses; A.R.Mc.T., D.J.Z. and A.D.W.G., the phylogenetic analyses; F.M., D.C., N.C., S.S. and S.V., the plant genome mapping; A.D.W.G. and D.C., the transcripts analyses and pairwise sequence comparisons; P.-Y.T., R.W. and H.Q., project coordination; and A.D.W.G., F.M., D.C. and P.-Y.T. have written the manuscript.

## Additional information

**How to cite this article:** Geering, A. D. W. *et al*. Endogenous florendoviruses are major components of plant genomes and hallmarks of virus evolution. *Nat. Commun.* 5:5269 doi: 10.1038/ncomms6269 (2014).

## Supplementary Material

Supplementary Figures and Supplementary Tables Supplementary Figures 1-4 and Supplementary Tables 1-4

Supplementary Data 1 Sequence files in fasta format are provided for each reconstructed florendovirus genome. These sequences are consensus sequences produced by assembling endogenous virus sequences that are present in each of the plant genomes, and as they are not primary sequence files, cannot be deposited in the International Nucleotide Sequence Databases (e.g. NCBI).

Supplementary Data 2 List of primary sequences used to reconstruct florendovirus genomes.

Supplementary Data 3 Summary of the genome organisations of the different florendovirus species, including the start and ends of open reading frames (ORFs), the translational reading frames of ORF 2 relative to ORF 1, and the masses of the predicted proteins.

Supplementary Data 4 Descriptions of conserved motifs in the florendovirus genomes.

Supplementary Data 5 Survey of plant genomes for loci that contain entire or nearly entire florendovirus genome or component sequences, and records of the integrity of the open reading frames.

Supplementary Data 6 List of endogenous Oryza sativa B virus loci and their presence or absence in closely related Oryza AA genome taxa.

## Figures and Tables

**Table 1:** Contributions of endogenous florendoviruses to the genomes of various plant species.

**Species**	**Plant genome assembly size (bp)**	**Endogenous florendovirus coverage (bp)**	**% of plant genome comprising endogenous florendoviruses **
*A. trichopoda*	668,257,121	5,674,476	0.85
*A. caerulea*	301,982,859	242	0.00
*Arabidopsis lyrata*	206,667,935	36,979	0.02
*A. thaliana*	119,146,348	3,438	0.00
*Brachypodium distachyon*	271,923,306	0	0.00
*Carica papaya*	342,680,090	0	0.00
*C. reinhardtii*	120,404,952	0	0.00
*C. clementina*	295,550,349	2,003,650	0.68
*C. sinensis*	319,231,331	1,272,462	0.40
*Cucumis sativus*	203,058,019	335,781	0.17
*E. grandis*	691,297,852	797,465	0.12
*Fragaria vesca*	214,219,504	339,506	0.16
*Glycine max*	973,344,380	1,889,571	0.19
*G. raimondii*	763,818,933	757,990	0.10
*J. curcas*	285,858,490	2,806,965	0.98
*Linum usitatissimum*	318,250,901	0	0.00
*Malus domestica*	881,278,625	1,016,000	0.12
*Manihot esculenta*	532,507,280	234,896	0.04
*Medicago truncatula*	307,481,907	219	0.00
*Mimulus guttatus*	321,726,589	68,534	0.02
*Oryza sativa*	373,706,981	123,914	0.03
*Panicum virga*	1,358,078,670	4,078	0.00
*Phaseolus vulgaris*	486,869,582	720	0.00
*P. patens*	479,985,347	0	0.00
*P. trichocarpa*	417,137,944	386,859	0.09
*P. persica*	227,252,106	530,315	0.23
*R. communis*	350,631,014	4,662,131	1.33
*S. moellendorffii*	212,761,159	0	0.00
*Setaria italica*	405,737,341	0	0.00
*S. lycopersicum*	781,666,411	300,953	0.04
*S. tuberosum*	727,424,546	305,991	0.04
*S. bicolor*	738,540,932	48,368	0.01
*T. cacao*	351,351,221	90,504	0.03
*Vitis vinifera*	486,198,630	3,152,021	0.65
*Zea mays*	2,065,722,704	0	0.00

**Table 2:** Variation in the contribution of endogenous Oryza sativa B virus to the genomes of a range of *Oryza* species and *Leersia perrieri*.

	**Genome**	**Hit counts**			**Genome occupancy (bp)**			**Genome fraction (%)**		
	**type**	**compAsc1**	**compBsc1**	**Total**	**compAsc1**	**compBsc1**	**Total**	**compAsc1**	**compBsc1**	**Total**
*O. s. japonica*	AA	11	39	50	25,976	98,105	124,081	0.0070	0.0263	0.0332
*O. rufipogon*	AA	39	61	100	21,429	40,579	62,008	0.0063	0.0120	0.0183
*O. s. indica*	AA	11	30	41	25,754	58,361	84,115	0.0069	0.0156	0.0225
*O. nivara*	AA	12	13	25	13,769	14,777	28,546	0.0041	0.0044	0.0084
*O. glaberrima*	AA	12	25	37	36,296	37,524	73,820	0.0127	0.0132	0.0259
*O. barthii*	AA	5	5	10	8,500	4,156	12,656	0.0028	0.0013	0.0041
*O. glumaepatula*	AA	3	6	9	2,561	5,475	8,036	0.0007	0.0015	0.0022
*O. longistaminata*	AA	19	22	41	3,231	5,579	8,810	0.0009	0.0016	0.0026
*O. meridionalis*	AA	14	13	27	6,310	5,959	12,269	0.0019	0.0018	0.0037
*O. punctata*	BB	6	17	23	19,540	39,584	59,124	0.0050	0.0101	0.0150
*O. brachyantha*	FF	—	—	—	—	—	—	—	—	—
*L. perrieri*	—	2	10	12	967	40,006	40,973	0.0004	0.0150	0.0154
Total		84	141	225	116,928	211,421	328,349	0.0486	0.1027	0.1512
