# Supplementary material for: Endogenous florendoviruses are major components of plant genomes and hallmarks of virus evolution
Source: Nat Commun. 2014 Nov 10;5:5269. doi: 10.1038/ncomms6269 (PMC4241990; doi:10.1038/ncomms6269)
Supplement: Supplementary Figures and Supplementary Tables — Supplementary Figures 1-4 and Supplementary Tables 1-4 [file ncomms6269-s1.pdf]

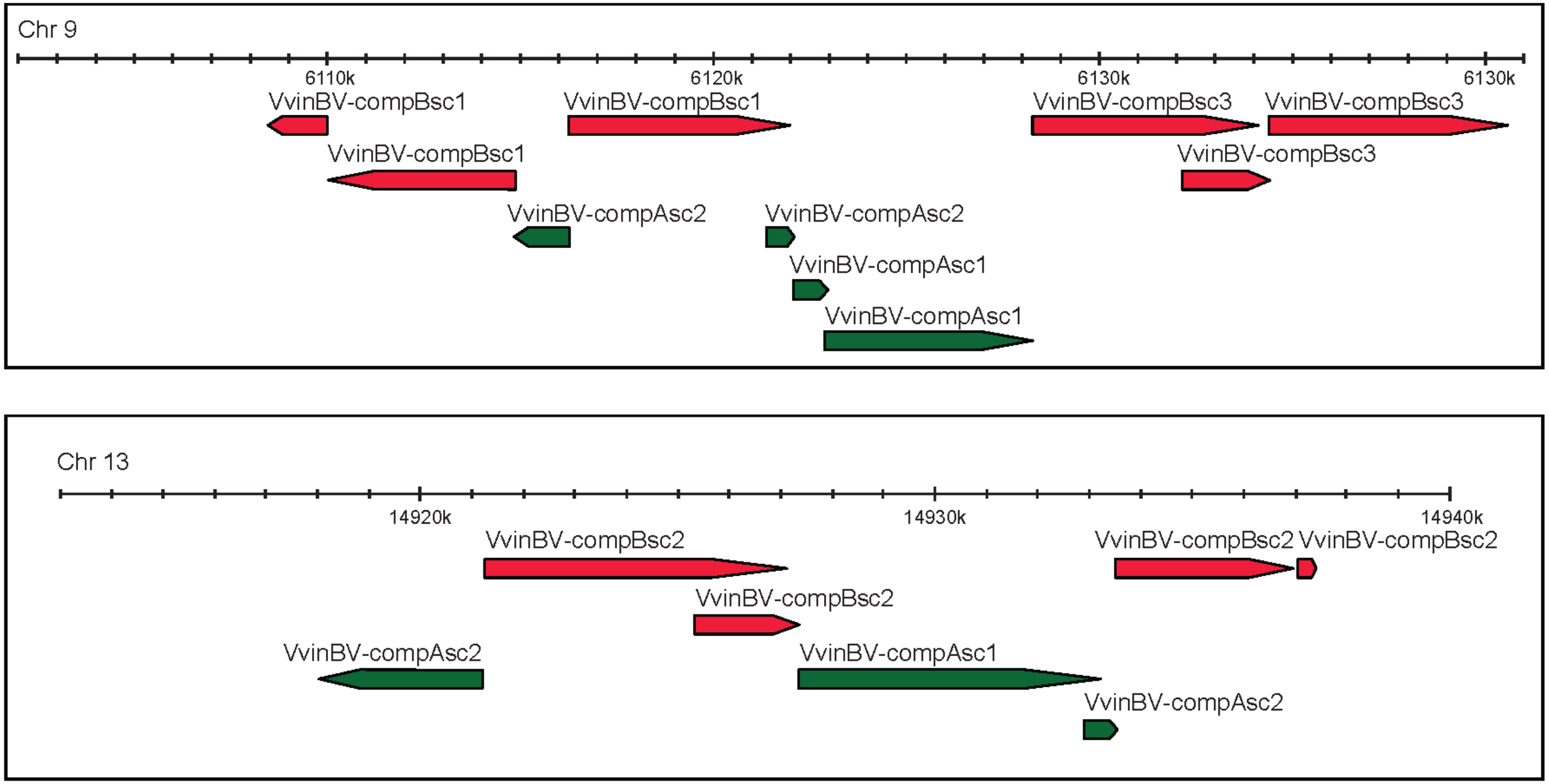

Supplementary Figure 1. Arrangement of endogenous Vitis vinifera B virus (VvinBV) genome components at two loci on the reference *Vitis vinifera* genome.

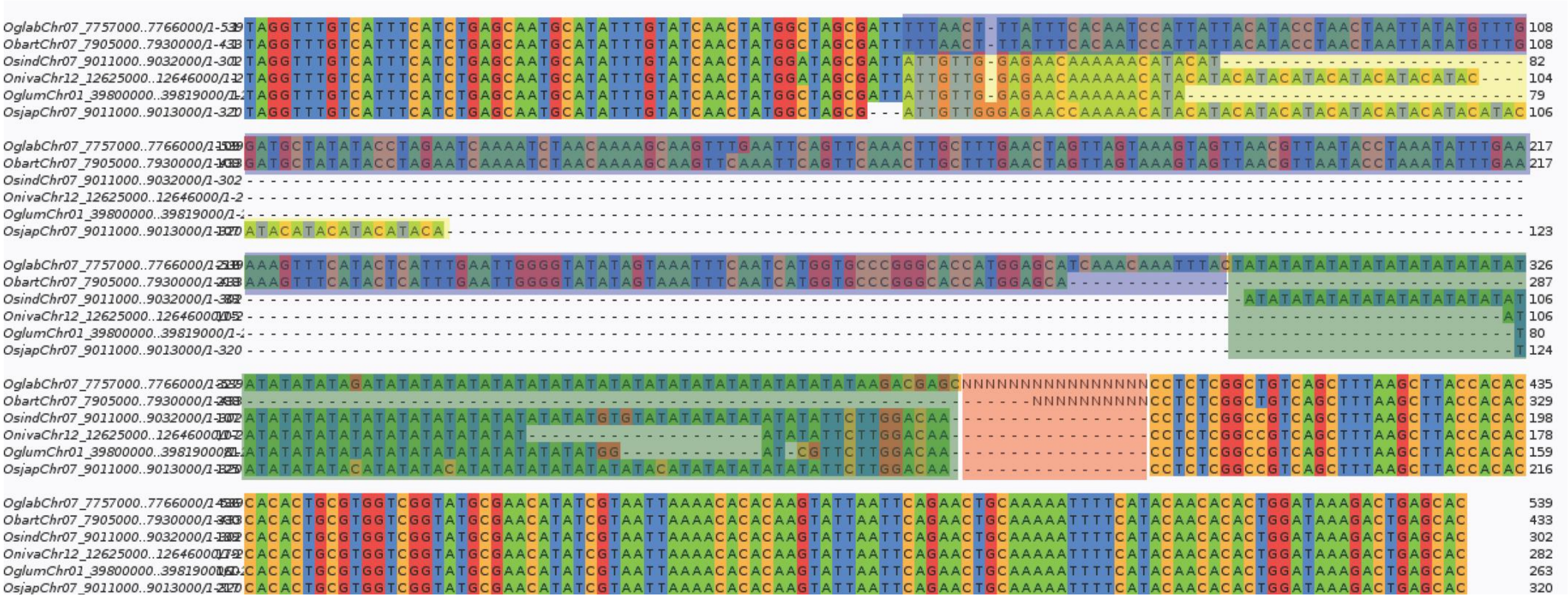

Supplementary Figure 2. Organization of the host sequence surrounding an Oryza sativa B virus (OsatBV) insertion. A complete OsatBV-compAsc1 insertion was shared between *Oryza glaberrima* and *Oryza barthii* (the florendovirus sequence has been replaced by Ns and highlighted in red – the *O. barthii* insertion was not completely assembled in its 5' end), and is located on chromosome 7. The other four species are included to describe the structure of the empty site. Upon insertion, some sequence was lost (yellow), and some was added, probably due to the DNA repair mechanism (blue). The length and sequence of the dinucleotide repeat evolved independently in each species (green).

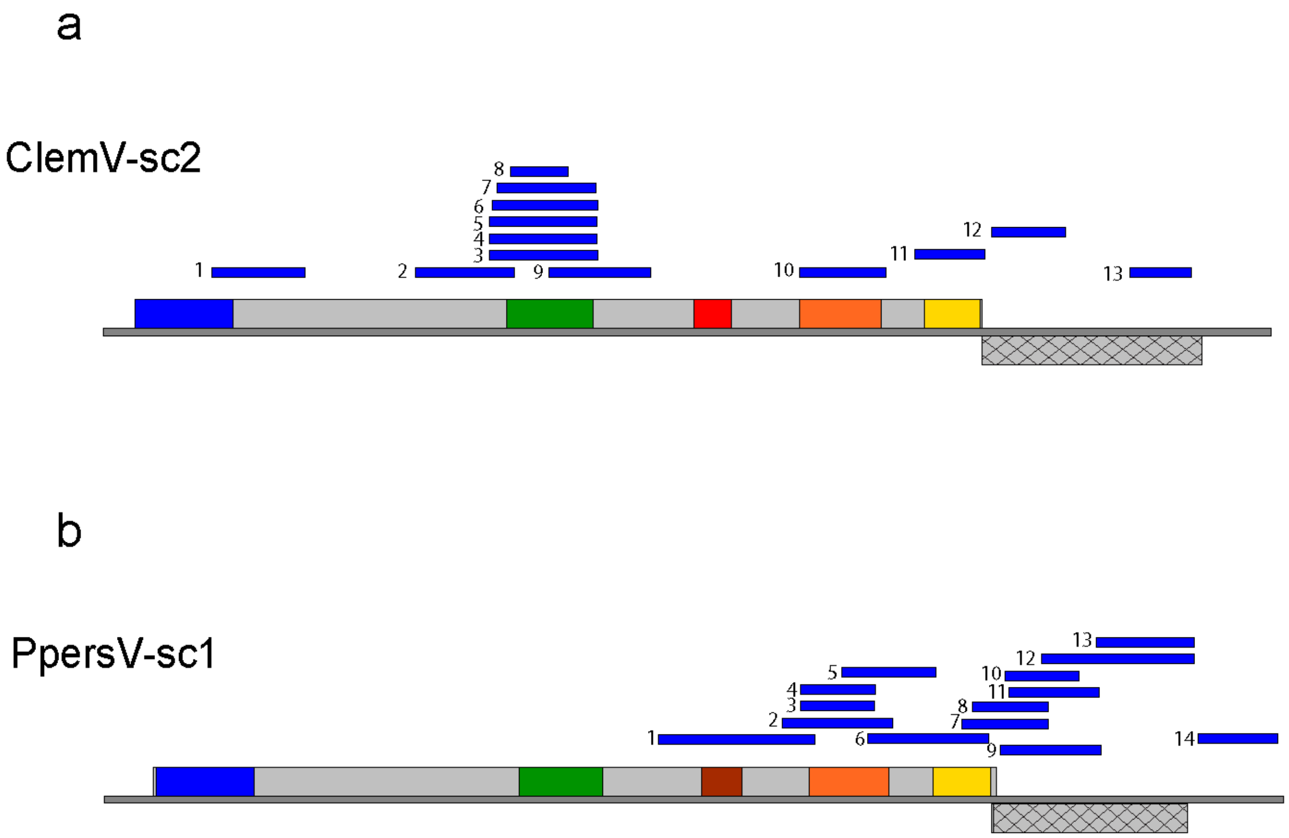

Supplementary Figure 3. Mapping of express sequence tags (ESTs) to florendovirus genomes. (a) Citrus clementina virus sequence cluster 2 (CclemV-sc2) and **(b)** Prunus persica virus sequence cluster 1 (PpersV-sc1). The GenBank accessions of the ESTs from *C. clementina* are 1) FC919332.1, 2) FC919333.1, 3) CX290102.1, 4) CX290168.1, 5) CX289814.1, 6) CX290170.1, 7) CX290502.1, 8) FC919335.1, 9) CX290711.1, 10) FC919329.1, 11) FC919330.1, 12) FC919334.1 and 13) FC919331.1. These ESTs were amplified from globular embryo tissue. The GenBank accessions of the ESTs from *P. persica* are 1) DW343673.1, 2) DW344610.1, 3) DN553877.1, 4) DN555087.1, 5) DW347536.1, 6) DW341851.1, 7) AJ873823.1, 8) AJ872920.1, 9) DW341303.1, 10) DW342036.1, 11) DW342153.1, 12) DW347617.1, 13) DW341785.1 and 14) DW342903.1. The GenBank accessions beginning with DW, DN and AJ were amplified from shoot tissue, leaf tissue and fruit mesocarp plus epidermis tissue 30 days after bloom, respectively.

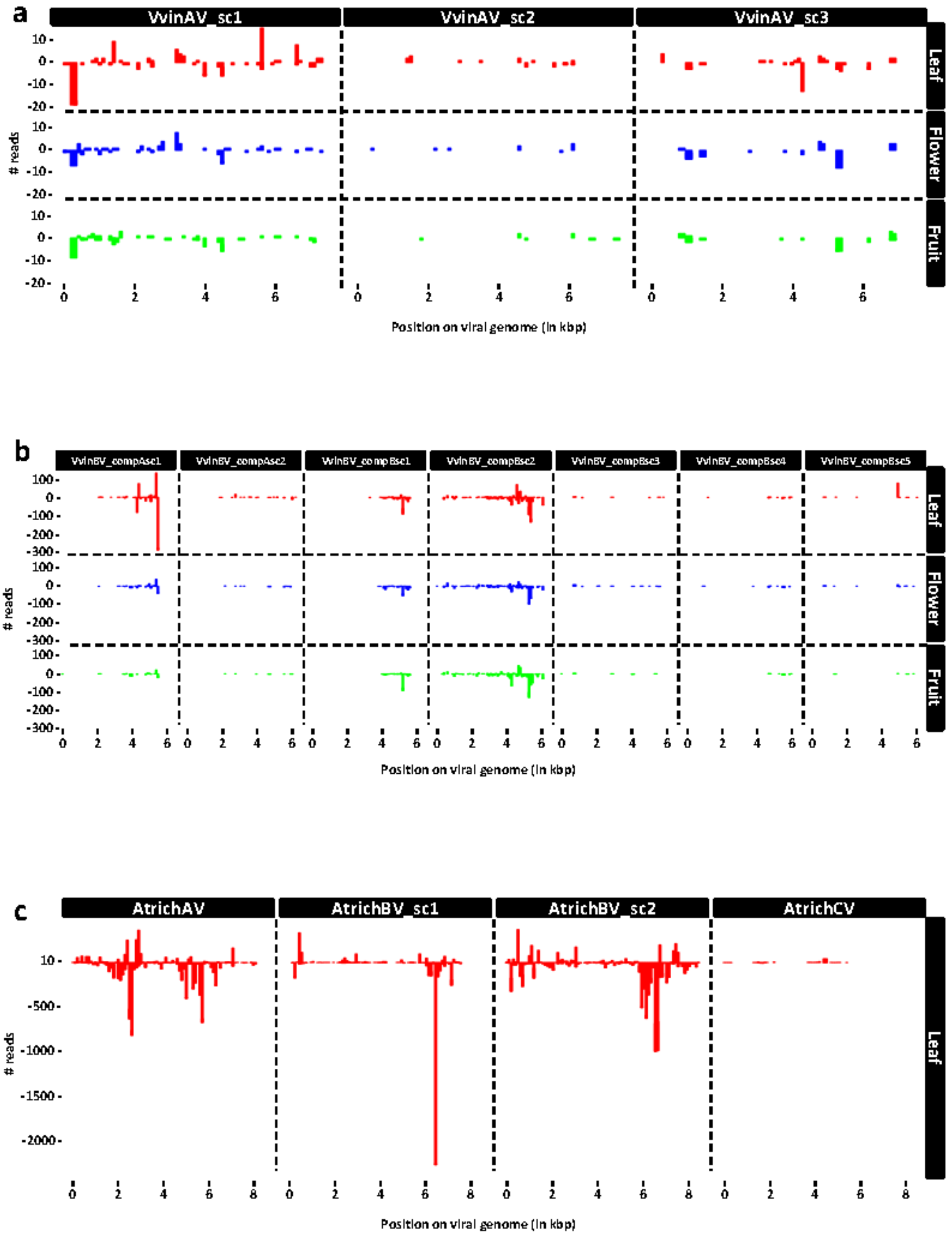

Supplementary Figure 4. Distribution of sRNAs (21-24nt) targeting VvinAV in (a) grape, (b) VvinBV in grape and (c) AtrichA, AtrichBV and AtrichCV in *A. trichopoda*. The density of the reads corresponding to positive (above the x-axis) and negative (below the x-axis) strand RNAs transcribed from leaves (in red), flowers (in blue) and fruits (in green) are shown.

Supplementary Table 1. Pairwise sequence comparisons for different regions of the florendovirus genomes in *Vitis vinifera*

Virus species	VvinAV-sc1	VvinBV-compAsc1	VvinBV-compAsc2	VvinBV-compBsc1	VvinBV-compBsc2	VvinBV-compBsc3	VvinBV-compBsc4	VvinBV-compBsc5	VvinCV-sc1	VvinDV-compAsc1	VvinDV-compAsc2	VvinDV-compBsc1
VvinBV-compAsc1	80.9/88.7/85.9<br>86.1/89.2[1]											
VvinBV-compAsc2	81.5/88.6/86.7<br>86.3/89.2	94.2/99.8/99.2<br>99.1/98.6										
VvinBV-compBsc1	79.5/87.9/80.0<br>---/69.2	95.8/98.9/73.3<br>---/67.9	89.9/98.7/73.7<br>---/67.9/									
VvinBV-compBsc2	82.1/88.9/82.4<br>---/71.7/	91.5/98.9/76.5<br>---/68.8	90.4/99.1/76.9<br>---/68.3/	93.6/97.8/96.1<br>---/76.3								
VvinBV-compBsc3	80.0/87.6/80.0<br>---/71.5	95.5/98.6/73.3<br>---/68.9	93.2/98.4/73.7<br>---/68.9	93.3/99.7/100<br>---/77.2	89.2/97.5/96.1<br>---/96.5							
VvinBV-compBsc4	78.3/87.9/80.4<br>---/70.4	93.8/98.9/73.7<br>---/67.5	90.4/98.7/74.1<br>---/67.1	90.6/100/99.2<br>---/75.4	87.5/97.8/96.5<br>---/96.3	91.7/99.7/99.2<br>---/96.9						
VvinBV-compBsc5	79.7/87.5/79.6<br>---/69.7	96.4/98.6/72.9<br>---/67.1	91.1/98.4/73.3<br>---/67.1	92.2/99.5/99.6<br>---/76.3	87.6/97.5/95.7<br>---/94.3	94.7/99.7/99.6<br>---/96.9	92.5/99.5/98.8<br>---/98.2					
VvinCV-sc1	55.5/46.2/57.3<br>69.8/67.2	51.7/46.8/56.1<br>70.4/68.0/	52.1/46.6/56.1<br>70.7/67.5/	51.6/47.1/56.5<br>---/55.6	51.3/46.8/57.3<br>---/55.8	51.7/47.1/56.5<br>---/55.3	49.7/47.1/55.7<br>---/55.0	51.0/46.9/56.9<br>---/54.8				
VvinDV-compAsc1	56.1/---/59.2<br>70.9/69.4	53.7/---/57.3<br>71.3/71.0	53.5/---/57.3<br>/71.0/	53.1/---/55.3<br>---/56.8	52.2/---/56.5<br>---/60.8	53.3/---/55.3<br>---/60.1	53.6/---/55.3<br>---/58.8	54.0/---/55.3<br>---/58.3	50.1/---/67.1<br>74.8/74.0			
VvinDV-compAsc2	54.0/---/58.0<br>71.5/69.9	51.5/---/56.9<br>71.1/71.0	51.8/---/56.9<br>73.5/71.0	51.3/---/54.5<br>---/59.4	50.8/---/55.7<br>---/62.1	51.7/---/54.5<br>---/62.7	52.2/---/54.5<br>---/60.8	51.8/---/54.9<br>---/61.0	48.3/---/66.7<br>73.7/74.0	86.8/---/95.7<br>95.4/97.3		
VvinDV-compBsc1	55.3/---/---<br>/---/---	53.4/---/---<br>/---/---	53.3/---/---<br>/---/---	53.3/---/---<br>/---/---	52.7/---/---<br>/---/---	52.9/---/---<br>/---/---	54.0/---/---<br>/---/---	53.8/---/---<br>/---/---	50.8/---/---<br>/---/---	89.6/---/---<br>/---/---	82.5/---/---<br>/---/---	
VvinDV-compBsc2	50.7/---/---<br>/---/---	49.1/---/---<br>/---/---	48.9/---/---<br>/---/---	48.2/---/---<br>/---/---	47.7/---/---<br>/---/---	48.3/---/---<br>/---/---	48.3/---/---<br>/---/---	49.0/---/---<br>/---/---	45.8/---/---<br>/---/---	74.5/---/---<br>/---/---	67.6/---/---<br>/---/---	71.2/---/---<br>/---/---

[1]Values provided in each table cell are % nucleotide sequence identities for the intergenic region (top left), movement protein (top middle), aspartic protease domain (top right), reverse transcriptase domain (bottom left) and RNase H1 domain (bottom right).

Supplementary Table 2. Size distribution and copy number of endogenous florendoviral elements in *Vitis vinifera*

Virus species[1]	Virus genome length (nts)	Covg.[2]	No. of frags[3]	No. of full lgth frags[4]	Copies	Full lgth copies	Mean lgth (bp)[7]	Mean lgth (%)[8]
VvinAV_sc1	7353	273311	398	0	318	0	860	11.70
VvinAV_sc2	7683	166175	296	1	247	2	673	8.76
VvinAV_sc3	7099	1106470	4340	0	4090	0	270	3.81
VvinBV_compAsc1	6225	217735	194	10	175	13	1244	19.99
VvinBV_compAsc2	6264	153379	182	2	163	4	942	15.03
VvinBV_compBsc1	6258	269549	257	11	206	14	1308	20.91
VvinBV_compBsc2	6194	524120	404	26	324	33	1618	26.12
VvinBV_compBsc3	6145	67223	51	7	49	7	1372	22.33
VvinBV_compBsc4	6137	96287	114	1	92	1	1046	17.05
VvinBV_compBsc5	6361	12743	44	0	40	0	318	5.01
VvinCV_sc1	7640	403571	608	1	517	1	781	10.22
VvinCV_sc2	7640	124991	284	0	255	0	490	6.42
VvinDV_compAsc1	5566	2598967	3504	3	3362	5	773	13.89
VvinDV_compAsc2	5474	2900579	3642	2	3441	2	843	15.40
VvinDV_compBsc1	5633	845502	1058	14	952	16	888	15.77
VvinDV_compBsc2	5739	1531840	2107	0	2002	0	765	13.34

[1]Acronyms of virus species are defined in Supplementary Data 3.
[2]Cumulative *Vitis vinifera* genome coverage (bp) by the virus species.
[3]Number of fragments of endogenous virus sequence before the "long join procedure".
[4]Number of full length fragments of endogenous virus sequence (≥95% of virus genome) before the 'long join procedure'.
[5]Number of fragments of endogenous virus sequence after the 'long join procedure'. The 'long join procedure' connects two parts of contiguous viral sequence that have been interrupted by other non-contiguous viral sequences that have inserted more recently.
[6]Number of full length fragments of endogenous viral sequence (≥95% of virus genome) after the "long join procedure".
[7]Mean length (bp) of all fragments of endogenous viral sequence after the 'long join procedure'.
[8]Mean length of all fragments of endogenous viral sequence after the 'long join procedure' but expressed as a percentage of the length of the reference virus genome or component sequence.

Table 3. Analysis of redundancy in sequences flanking endogenous florendovirus loci in the *Vitis vinifera* genome

	No. of joined loci with length ≥ 1 kbp[1]	No. of flanking sequences[2]	Largest cluster of flanking sequences (≥90% nt identity)[3]	Largest cluster of flanking sequences (≥80% nt identity)[3]	Annotation of the largest cluster[3]
VvinAV	103	206	6	13	VvinBV-comB
VvinBV	238	476	5	5	*Gypsy*
VvinCV	56	112	2	2	
VvinDV	232	464	3	5	*Gypsy*
Gret1	328	656	124	261	*Gret* LTR

[1]To obtain joined loci, florendovirus and Gret1 annotations obtained with RepeatMasker were joined when there was less than 1 kbp distance between one another (joined loci).
[2]Joined loci with a length >1 kbp were then selected and the flanking 500 bp sequences extracted. The flanking sequences from each of the EFE and from Gret1 were pooled and sequences were grouped into clusters on the basis of either an 80 or 90% nucleotides identity threshold.
[3]The number and identity of sequences found in the largest cluster is indicated.

Supplementary Table 4. *Oryza* species and sources of genome sequences used in this study

Species	Genome type	Accession number	
		Whole genome	Chr3S[2]
O. s. ssp. japonica	AA	http://rice.plantbiology.msu.edu/	**[3]
O. rufipogon	AA	PRJEB4137	**[3]
O. s. ssp. indica	AA	http://rise2.genomics.org.cn/page/rice/download.jsp	**[3]
O. nivara	AA	AWHD00000000.1	JNWG00000000
O. glaberrima	AA	ADWL00000000.1	ADWL01000000
O. barthii	AA	ABRL00000000.2	ABRL00000000.1
O. glumaepatula	AA	ALNU00000000.2	ALNU00000000.1
O. longistaminata	AA	*[1]	**[3]
O. meridionalis	AA	ALNW00000000.2	ALNW00000000.1
O. punctata	BB	AVCL00000000.1	JNWE00000000
O. minuta	BB	-	JJNN00000000.1
O. minuta	CC	-	JJNN00000000.1
O. officinalis	CC	-	JJMQ00000000
O. brachyantha	FF	AGAT00000000.1	JNWF00000000
O. granulata	GG	-	ALNT00000000.1
Leersia perrieri	-	ALNV00000000.2	ALNV00000000.1

[1] *O. longistaminata* genome assembly was kindly made available by W. Wang.
[2] Sequences of the short arm of chromosome 3.
[3] The genes on the short arm of Chromosome 3 (Chr3S) of these species were extracted from the whole genome assembly.
