# Supplementary material for: Endogenous florendoviruses are major components of plant genomes and hallmarks of virus evolution
Source: Nat Commun. 2014 Nov 10;5:5269. doi: 10.1038/ncomms6269 (PMC4241990; doi:10.1038/ncomms6269)
Supplement: Supplementary Data 1 — Sequence files in fasta format are provided for each reconstructed florendovirus genome. These sequences are consensus sequences produced by assembling endogenous virus sequences that are present in each of the plant genomes, and as they are not primary sequence files, cannot be deposited in the International Nucleotide Sequence Databases (e.g. NCBI). [file ncomms6269-s2.doc]

**Supplementary Data 1. Reconstructed florendovirus genomes**

>AtrichAV [endogenous-virus-name=Amborella trichopoda A virus] [host=Amborella trichopoda] [moltype=genomic DNA] [note=complete genome] 8241 bp

TGGTATCAAAGCCAACCCTGTTTTAGAAATCTAAAAGTTAATACTAGTTCTTCTATCTTA

AGAAACTTAATAAATTAATCCATTAAAGAAAGTTGGTCCGAGAATTGAGGTTAGAGACGC

TCAGGACACCCAGTCAGTTGACCGTTTAGGCCAGGAGAGCCGCTGAGGTCTAGAAGACGT

AAGAGGTAGTCAAAGAGGACTGTCCAGATAACACTCAACCGCAACCTAATAATGGAAAAA

TTTATTAAGAATCTGACGGATAAAACAACTAGTACAACTAATAGAACTCTAAGGATAAAA

AACAAAGAAGAAATTGTTATCAATGATATTGAAAACCAAATACAAAATTGGAACATACCC

AAAGTTCCAATATCAGATGTCTATAAGAAAGGCACATTCGATCTAAAAATCGGATATGCT

ATTAAAACAGTAGAAGAAACTTTATCTGTTACAAATAATCAAGAAGACTTTCAATTATTA

TCTCAAAAGATATTACAAGTCCATAAAACAAAAAACTATAAATATTTACATATAGGATTA

ATTCAAATAGCTGTAAAACCTTTGACTCGTTTAGGACTAAATAATTCTATCTTAGTCTGT

TTACGTGATACTAGGCATAATAGATTTTCTGATTCACTACTAGGAATGGTTGAATCTAGT

TTATGTAGTGGACCAATATATTTCAACTGTTTTCCAAATTATTCTGTATCTCTTACAGAT

CCAAATATTTTAAATGTTTTATCTTTAAATCTCAAAACTCAAGGATTTAATATGATGACA

GGTTCTCAAAATATAGCCATTGTTTATCGAATCTATTACAAAGTAATGACTACATTATGT

CCAAATGTACTCCAAATAGATACGAGAGGACAAACAATATTTTTTCAAACCAATATGGAA

AAATCAAATACAATTGTACCAAAAACAATATCATGGTCAGATATTGTTCTACCTCAAACA

TGGTCTATCCATAATGAAAACCAACCATCACAAAATCCCACTCAAAATATTCCAGACAAA

ATTATTGAATATACAGACGGAAATATTGAAATACAATTCACCACAAATAATAGAATTATG

GATAATGCATCCATCTCAGGTACTTCAAGTACTCATTCTTTTCGAGATCCAGAACCTCCA

AGAAAATCCATTACTACTTATAATCGAATTCCTCCTATAATAAATATCCCATCAACACCA

GTAAAACCACAAATTGATGAACTCCTAAGGACACCTGATAATATCATTATTCCCCAATAC

AAAAGAGCTCAAGAAGAACCTGCTGAAGCAGAATTCATTCAAGTATTACAACTTGAAGAA

ACATTCGATAAAGAATACATTATAAAAGATTTTTTATCTCCAGAAAATAAAAATATTAGA

GAATGGTATGTAAAAGAATTTGAACCTAGATTAGTAGAATTGTTTCACTCAAGATTTAAC

AAATGGAGAAAAAAGGGAAATAAAGGAACATGTTTTTTCGATTGGCTAAGAGACACCTTA

AGCCAACACAATATCCCTATTCCTGATTTTGAAATAAATACACTAGAAACTCAATATACC

GTATTTAATACTACTGAAGGAAATACAATTAAAGCCATACATCCTCCTGAACAAAGCTTG

AAAATACAAGGAGTTGCTACTATGGTATCAGCTACTAAAACTCCTGCTACTCCAGAACAA

AATATAGGATCCTCTTCTTCATCTTCAACCCAAATGGTTGTATATGAAACAGTAGCTACA

CCATATAAACTCAATAATGCAAATATAGCTCGTATAGAATTACTTCCACATATAATTGAA

CAAAATAATTATACAAATCTCTGCCTCCATACACTTGGACAACAAACTAATAGAATAGAA

AACTTATTACATCCTCCCATTTATAAGGAGAAAAAACAATTATTTTTCAAACCTCCTCCT

GATTTGAATAAAGAAAAATTTCATATAGGTGATGATAATAAAACACTTATGGATCAATTA

ATCTCCAAATTATCCAAATTAGAAATAAAAGATCCCAAACAAACTATTGTTTTACCAGAT

AATGATAAAGGAAAAGCAAAAATCCCTGAACATGATAATATGACTCTACAAGAAGAAATA

CTTTTCCAAACCAATGAAACCAAGGAACCACCAATTGTCAACACAATAGAAAATATGCAA

ACAGCGTTTTCTGAAATTCAAATTACTCCAGATACGATAAATCGAATTAATTACAGTAAA

AATTGGCAACCAATAAATGCCCGACCATATTATAAAAGACCAACCCCAGTTGATATCCAA

TTAGAAGAAGACCTCACTAGTCATACTTCTACATATACGGGTCGAACGATTATAGATTGG

AATATTGATGGAATTTCTGATCACCGAATATTAGATACAATGAAACATATGACTATGTAT

GCTTCGGCATGTAAAATGGCTGGTAGTCATGATCAAGAAATAGCAAAAGCTATAGTTTCA

GGATTCACCGGATGGTTAAGAGGATGGTGGGATTCCTATCTCAATGAACAAACAAAACAA

CAAATATTAACTAATACTCGACAATGGACAGCAAAAAATGAGCACAACCAGGATATGGTA

TATTCTGAAAGTGATGCTGTCAATTCCTTAATCTATACAATTGCCCTTCATTTTATAGGA

AATACTTCAATACTTGCAGCACGAACTGCTGATCTTTTATCTAATCTACGATGTCCTACA

CTATCACACTATAAATGGTATAAAGACGTATTTTTCTCTCGCTTACTCATACGTGAAGAT

AGTTATCTTGACCACTGGAAAGAAAAATTTGTAGCAGGATTACCTTATCTATTTGCTGAA

AAGGTTAAAGAACGTTTAAAAAATAAAAATAGTGGTGTCATCAATTACTCTCAATATACT

TTAGGAGAATTATCTTCAGAAATCTGTGCTGAAGGCTTAGCCCTATGTACAGACCTGAAA

CTTAAAAAGCAACTTGATAAACAAAATTTAAGTGGTAAAAAAGAGTTAGGAGATTTCTGC

CAACAATTTGGCTATCATATGGATCAAACTCATATAGATAAAACAAAACCACATAAAATA

CATAAAGTATATAAAAAGAAAAAACATCGTAATAAACCTGAAAAAACATATAAGTCTTTT

CCAACCAAACAGAAAAAATATAGAAAATTTACGAAACGTTTTGCTTCCAAATCATCCCAA

CAGAATAAAAAGAAATTACCCGTTTGTTATAAATGTGGCAAAGTCGGCCATTATAAAAGC

CAATGCAAAGTCAAACAAAAACTAGACTCCATTACATTAGATGAGTCTATCAGAAATAAA

ATCTATGATATTATTCAACTATATGATTCGTCTGATAGTTCTGATACTGACACTACTTCC

TCAAGTAGTGATACTTCTTTCATAGGAACAATACATAATGATGATTTTTATTGGTCAGAT

ATTAGTTCTGATACAGAAGACGAAATATGCCAATGTCCTACAACTTCTGGTATTCATACA

TTAACAAAAGAAGACAATCTTATTTTAGATTTAATAGAAAAAATATCTGATCCGGATGAA

AAAAGAGAAATAATTACTAAGTTTATCAATTTAACTAAAACAAAAACAAAACATATGCCA

TTAGTTAAACCTCCTGATAATTATAATTTTTCTGATATCATGAAACGTATCAGTCAGCAT

GAAGCCACCCAAAAAGAACCTACTATTGCTGAACTCAAAGCCGAAGTCAATATAGTTAAG

GTAGAATTATCCCAATTAAAAAATAGAATTCAAATCCTTGAACTCCATGACAATACCTCT

GAAATAGATACTGATACAGATGTCATAGAATTTAACCATTTACTCAAATCATATCATCCC

AATGATTATCTAACTAAATGCGAATCATCCACCAGACATTCAGTAAATGTTTTAAACCGA

GTAATCACACAAAAATGGTTTGTGCTAATAACCATTGTCATAAATGATGAATATGAATTC

CAATCTCTAGCCTTGGTAGATTCAGGTGCTGATCTGAATTGCCTCAATGAAGGATTAGTC

CCCACAAAATATTTTGATAAGACTACCCAAATCCTTAATACTGCCGATGGTAGTAGACTA

TTCATAAAATACAAGTTATCTAATGCCAAAATTTGCACTGAAGGACATTGTTTTCCTACT

CCATTTATCATGGTTAAAGGACTCAGCCAAGCTGTAATCCTTGGCGTACCTTTCCTCACA

TTATTATATCCAGTTACTATTTCCCATACAGGCATTGAAACAAATACTCATGGTACCCCT

ATAAAATTTGAATTTCTTGATAAACCCAAAATTAAAGAAATCAATACTGTCAAACAAAAT

ATAAAACAAAAAGAAAATTTTATAGGATCATTACAACTTGAAATCAAACATAAAATTATC

AGTGAAAAACTCCAAACTAATGATATGATACAAAAGATACAACAACTCAAAAATTTTATT

ATAAGCGAAATATGTAATGAATTACCAAACGCCTTTTGGCATAGAAAACAACATATTGTT

GGACTACCATATGAACCAAATTTTAATGAAACAAAAATACCGACAAAAGCTAGACCTATC

CAAATGAATACAGAATTATTACAAACATGTCATCATGAAATACAAGATTTACTCCAAAAG

AAACTTATACGTCCTAGTTCATCTCCTTGGAGCTGCTCTGCTTTCTATGTCAATAAAAAT

GCTGAGCAAGAACGAGGAGTTCCACGATTGGTCATTAATTATAAACCCCTCAATAAAGTC

TTACAATGGATTAGATATCCCATTCCAAATAAAAAAGATTTACTCGATAGACTCTTACAT

GCTGTAATCTTCTCCAAATTCGATTTAAAATCTGGTTTTTGGCAAATACAGATAAAAGAA

GAAGATAAATATAAAACAGCTTTTAATGTCCCTTTTGGACAATATGAATGGAATGTTATG

CCTTTTGGTCTTAAGAATGCTCCATCTGAATTCCAAAAAATAATGAATGATATCTTTAAT

CCCTTTTATAAATTCATGATTGTTTATATTGATGATGTCCTCGTATTTTCAGAATCTCTT

GAACAACATTTTAAACATCTTCATACTTTCATCCATACAGTTAAAAATGCTGGATTAGTA

GTTTCTGCTAAAAAGTTGAATCTTTTTCAAACAAAAATTAGATTTTTAGGACATAATATA

AATCAAGGGACCATTATTCCAATTGATCGATCTATTCTCTTTGCTGATAAATTTCCTGAC

AAAATATTAGACAAAACTGAATTACAACGATTTTTAGGATGTCTAAATTATATAAGTGAT

TTTTACCAAAATTTAGCTCTTGATGCTAAACCTTTATATGACCGATTAAAAAAGAATCCT

CCACCTTGGACTGATGTTCATACTTTGGCTGTACAACGACTTAAACTTAAAGCCAAAGAA

CTTCCATGTTTAACTTTGTCTGATCCCACAGCTTCTAAAATCATTGAATGCGATGCTTCT

GAATTAGGACTAGGAGGTATATTAAAACAATCTATTAATTCTAAAGAACAATTAATACGG

TTTACTTCCAAAGCCTGGAATCCAACCCAAAAAAATTATTCTACTGTCAAAAAAGAATTA

CTTGCCATTATTCACTGTGTTTGTAAATTCCAAACTGATATTTTGAATCAACACTTTCTC

ATCCGCACCGATTGTAAAGCCGCTAAAGACATTCTGCAAAAAGATGTTCAAAACCTTGTA

TCCAAACAAATATTTGCCAGATGGCAAGCTATTTTATCTGCTTTTGATTTCTCTATTGAG

CATCTTCACGGTTCTACTAATTCTTTACCTGACTTTTTAACCCGTGAATTTTTGCAGGGG

GGACCGCACATACCCATTACACCCTAAAGACGATACGGTTTCATCCTACGGATTTTCATC

CTACGGTTTGCAACTCTTTCTTAATAATAAGAATATCAATATGAATAATCCTCCGTCTTC

AAATACCAACAAAGGAAAACAACCAATGGACTTAGTACCAAGCAACAGATATGGGCCTCT

TATGCCCATCCAATCCTCTCTAAGTCTGACACCTATGTCACCTCTCAGATCCTCAAATGT

ACCTGAATACTTGCTACATACTCCTCCACAAAGTCCAATACAAACCCCAAGTCCGGCGTT

TTCCCCGGCAACATACCCAAATAAACAAACCTCGACCTCTGCCAAATCAGTCTATATAGA

CACAGCCTTCTTATCAGATATACCCATTTCTGATACTCATGCACAACTTGACCCAAATAA

AATAGCTCCCCTTTATCTTGTAAACCATTTGAATCCATCATACGGAGTCAAAACCCGTGA

ATGGTATGAAGCTATTCTTATGGAAACCGGTTCGGTTTCAATCGTTCATTTTTATCAAGG

TACAACTAAAGTATATGCTTATTCTAAATTTCAAATCAAAAAATTACTTTCTTATGATGA

TTGGGGATGCAATCCTCTTCAAGGAAAAAAGTTATTGAATCCAGAATTCCAATATAAATA

TTATAAATATTCTGAGTATATTGATGCATGGCACAAGGTATTCTCCTACCAAAATCCAAA

TAATACGCATTCCTGGTATTTGCAATTCAAACTCCCTATTCTCGAAAGATTCCCAAATTG

GTTCCGCCATTGGTTCTTATTATGGGGATCACAGACCCAAATTTTACCCGATGCTTTCCA

AGCCTTACAGACAAAATTTGCAGAATATAATACTTGTACTGACCAAGATGATTTCCATCT

CTACTTCATGGCTATCTACCAAATACCCTGGATCCTAAAATGGGAATACTCTATTTCTGA

GAATAATACTAATAATTATGAGTATATTCCCAAACTTGGCAAAACCCTGAAAATCAAATG

GTGGGATATTTTCAGTACTAATCCTGCCTCCTTACCCCTTAAACGAATACCGCCTCAAAC

TAATGTCTTCAACCCACCAGCATATAATACTGTCAGTTTACCCACATTTTCGGCCTATCT

GAAGAAAATGAAACAAGCACATCCTAATGCTACCTTAGAAGAAATAGAGCAGAATCTTCT

TCATATATTTGCTCCTGATACTATTGCACCAACAACTTCAGAAGAAACTGTCCAATCAGG

CAATCCCTTATTTGACGACTTCAGCCAAAGACCCGATGCTTAACACGGCCCGACAGTCCA

GCCGGCCCGATAGTCCAGCCCGAGATGATCCAGATAAATAACTCCACTGTTCGCTGATAT

GACCTCACCATCATAAGAAACAAGTAAAGATTCCCTTTTCATAAAGTGACAAGACATCTC

AATAGTACTTCATAAAGAGACAAGACATCTCAATAGTAAAGATTCCCTTCTTCATAAAGT

GACAAGACATTTCAATAGTAAAGATTCCTTCTTTACCAATGACAAGACACCTCCAAATAG

TAAAGATGCTCTTTGATCGATGTGACTAATTTGGGGTCAAATCATAGTCACGCTTCTACC

AACGTGGCATCGTCTCCTTCTTCGTTGCTCACACGTATCGCCACTATTCCGACAAACAAA

AGATTCGCCATCATGTCAATCACCATCCCAACGACTGGCTACTCCCTGCACCTGCCACGT

ATCCCTCTTCATACGACAACCTTATCTCCAGGTTCCTTCGTCACTACGCTAACGAACCTC

AGCCACAAGCTTCCCTTCACCTTATCCCCAGGTTCCTTCGTCGTCACGCTATCATCCACA

GCCACAAGTTTTCCTGGGTCATTCCTCTATAAATACCCACTCGACCAGTTGAGAAAGGCA

GACGGCATCAAGGACGCAGCAGCAGCAGCAGCAGAAATTCTCAAGCCCTCATATGCTCTC

TATGCTCTCTCATATTCTCTCTTCTTATTCTTCTTCTTCTTCTTCTCTCTTTCTGTAGAC

AGTAGGTTAGTTACTGCATTCAGTCACCTGTACATCGACAAAGTCGGTAACTTCCATATG

ACTTCTTTATTTTAGAATGTGTGATTGTAATCTATCTTCCTTACCTTCCTACTATCCTTC

TGAGTGGAAAAAGTTTGCCAGTCTCTGTATATGTAATCAGTAAGTTATGCAATTTAAATT

TGTTTTTACATTTAAAATTTGTGACTATACTCATACTTCGTGGATCATATTTGATATATC

CTTGCTTCCTGTTTTAATTATCTTATTTGTGTTTCTATATTATGTTACTATTCTGCAACA

ATTAATTACTTATAACTCTGCAATCTTTTCATATTTAAGATAAATAGTGGTAACTGCTTA

GGGTAGATCTTGCGGCGAAAA

>AtrichBV_sc1 [endogenous-virus-name=Amborella trichopoda B virus] [strain=sequence cluster 1] [host=Amborella trichopoda] [moltype=genomic DNA] [note=complete genome] 7597 bp

TGGTATCAGAGCCATACAGTAAAAACTGTGCAAACAGAAATCTGTTTATACAGTACATGA

ACAGTAAACTGTCCCAATAGTTCACTAGTCCTGTTAGAGTAAAAGTTATACTATTACTAG

GGAAACCTACGTAAATTTACCACTAAGAAAGTTGGTCCGAGAGTTAAGGCAAGAGACGAA

AGAAGTCTATTGCAAAGTACCCGCCAAGGCCAGGAGAGCCGCTTAGGGGAAAACGCTGGA

CCCTTGATCGTCCAGATTGTCATTATCCGCAACCTACAGATGGCAAATTTAATTAGGAAT

CTTAAAAGTACTAGTACAACTTCATCTCAAACACTTAGAGTGAAAAATAAGGAACAAATC

ATAATAAATGATATTGAAGATCATATTCAAAATTGGAATATACCAAAGGTACCAATACAT

GACATTTATCATAAGGGATCATTTAAATTCAAGATGGGTTATGCCATCAAGACAGTAGAA

GAAAGTTTATCGGTAACTCAGAACCAAGAAGACTTTCAACTACTATCAAGAAAAGTTATT

GAAAAACATAAAGAAAATAATTACAAATATTTACACATTGGCCTCATTCAAATAGCTGTT

AAACCTTTGACCCGACTAGGGTTGAACACCTCAGTGCTTGTATGCCTAAGAGATACAAGA

CACAATAATTTCACAGACTCACTACTTGGCATGGTTGAATCAAGCTTATGTAGTGGTCCA

ATATATTTTAACTGTTTTCCAAATTTTTCGGTTTCTTTAACTGATCCAAATATTCTCGAT

ATACTAACAATAAATTTAAAAACTCATGGTTTTAATATGACACATGGTTCCCAAAATTTA

GCTATCGTATACAGAATATATTACAAAGTTATGAATACCTTATGTCCAAATATTATGAGA

ATAGATAAAAAAGATCAAACTATATTATTTCAAACAAATATGGAAAAATCTCATACAATT

GTACCCAAAACCATAAAATGGTCAGACATTGTTCTACCACAGTCATGGGCATTGCAACAT

GCAAATCAACCCCAAGAAATTCCAAGAAATAGTATTCCAAATCAAATAATAGAACATCCA

GATGGAAATATTGAAATAAAATTCCAACAATTAAAAGTACAGGATGATATAAATATTAAT

CGAAAAAAGAATAAAAAACCATTAATACCTAGTAGAAAATCTTATTCTACTACCCCTGAT

GTTATTATACTACCTCCTCCTTCCCAATCTTCTTCACCATCAATACCTCATTCAGATTTA

CAATTACAAGGAATAAGAAGAACAACTGACCATGTTGTTATTCCCACATATACCCCTGAA

TCAAATATAGCATACACTCCTGAACCTAGTATAGAAGAATTTCAAATATTCACATTACAA

ATTGAAGATGATATAGAAGAAGAAATAAATCCATATGATCAATATTCTGATGAATATATG

GCAGCATACTTAGCAGCCAAATTGGAAAGGAAAAGGAGAAAAATAAATAAAGAGTATATC

AGACAAGATTATTTATCTGATGAAAATAAACATATTCGAGAATGGTATTTGAAAACATTT

CAACCTAGATTAATAGACTTAATGCATATAAAATATAATAAATTTCTTAAAAAAGAAAGA

GTAACCAAAGGATTTTTTATATGGTTAAAAGAAATGTGTAGTCAATACAATATTACTATT

CCTTCATTTGACAACATTCAAACAATAGAAGAAAAAACCTATATCAGTACGGATGGTACT

ACCACTGCTAGTACACACCCTCCTGAAAAAAGCATAATACTCAAAGCAGTCATCCCATCT

AAGGATGAACCACCACATCCACAATCAATATATGATATAATAGCTACTCCTTATAAGGTC

TTACCAAAAGAACTTGGCAAGAAACAGGAATTAATTCCTCATATTGTAGAACAAAATAAT

TATACAAATTTGTATATGCAATCTTTAGGTAAACAGGCAGAACGAATAGAAAGACTTGTT

TCAAAAGAACAACCTTCTGCTACACCCATACCTAAAGCTGTTCTGTTCAAACCACCTCCA

GAATTAAATAAAAATAAATTCCATATTGGTGATAACAATAAATCCTTAATGGATCAATTA

ATAGCAAAATTATCCCAATTAGAAATTAAAGGAATACCTTCTCCCAAAATAAATATTCCT

TCACCCACTATTACCAGTTCATTACCTGAATCTTCAGATAAAGGAAAAACAAAGCTACCT

GAGCATGAAACAATGATGCTCCAAGAAGAACCAATCATAGAAGATACTATCCAAAATCAA

GAAAATGCATTTATGGATACCACTCCCATAGATCTCAATAGAATAGCCTATGGAAAAAAT

TGGAAACCAATAACAGCCTTACCATATCATAAAAGACCTACACCTGTTGATATTCAAATG

GAAGAAGATGCTATACAACAAGTAGTATCATATTCAGGAAGATCAATAGTAAACTGGACA

ATAGATGGATTATCTGAATTAAAAATATTAGATGTCATGAGACATATGACAATGTTTGCC

TCTGCAAATAAAATGGCAGGCAACCCAGATCAAGAGGTTGCAAAAGCAATAGTAAATGGA

TTTACTGGCTGGTTAAGAGGTTGGTGGGATTTCTATGTAGATGATGAAACCAAGAATAGA

ATCCTCACAAATACTAGACAATGGACCGCCAAAAATGAACAGAATCAAGAAATAATCTAT

ACAGAAAGTGATGCTGTCAACACTTTGATATATACCATTACACTTCATTTTATAGGCAGT

ACAGCCCTATTAGCAGCAAGATCCGAAGATCTGCTTTCCAATCTTCGCTGTCCTACACTA

TCTCATTATAAATGGTACAAAGATGTATTTTTCTCAAGATTACTAATAAGAGAAGATTGC

CAGTTAGACCACTGGAAAGAAAAATTTATAGCTGGTTTACCATATTTATTCGCAGAAAAA

GTCAAAGAGAGACTGCGAAAGAAACATAATGGAATCATAATATATTCATCATATACACTT

GGAGAGTTATCAGCAGAAATATGTGCTGAGGGATTAGCCTTATGCACAGATATGAAACTC

AAAAGGCAATTAGATAAACAAAAAATCACTAAGCGTAAAGAACTTGGTGACTTTTGTGAA

CAATTTGGATATTATCTTGATAGAAAAAATATGCCTGACCAATTATATAAGAAAAAGAAA

AAATATAAAAAGGAAAAATATAAATCTAAAGATAAAGAAAAGAAAAAATATTATAAAAAA

TCTAAGAGAAAACTGATCAATAAATTTTCTCCCAATACAAAAGGTAAGAGAAAACTACCA

GTATGTTATAAGTGTGGTAAAGTAGGACATTATAAACATCAATGTAAATTAAAAAAGAAA

ATAGAATCATTACATTTAGATGAAACAATAAAATCAAAATTATATAATATAATCAATTAT

GATTCAACTTCTTCTACCTCTACTGATTCCCATATAGACCAAATTCAGGATGATGATCTT

ATATGGACAGAATCCTCTGATTCTTCCAATTCTTCTGATACAGAATCATCTAGTGCAATC

AAAGATAATGATTGTAGTTGTCCTATACCTTCACACAATCTTCATACACTTACCCAAGAA

GATAATTTCATCCTAGACATTATCGATAAAATAACAGATCCAGATGAAAAAAGATCTGTC

ATTACTCAATTCATTTCTATAACAAAAAATAAAGCAAAAACACTTCCCATCAATAAATCT

CCTGATGAATATAATTTTGCTTCAATAATGAAGAGAGTAAGTGAACAACATACTTCTCAA

CAAAAAGAACCTACAATTTCAGACCTGAAAAGAGAAATTAAAGAAATAAAAAATGAAGTA

TTCCAATTAAAAAATAGGATTAAATCTTTAGAATTACAACAACTATCTCAACAAGATACT

GATACTGATGAAATCGAATTTGATAAACTTATGACATATGAACCAGATCCACCATCAGAT

ATTCCAGAAACCTCAGAAGCCAAACCACATATAATAACCATTCCCTCTGTCAATTTAATA

GACAAAATAGTAATTCAAAAATGGTTTGTCCAAGTCAAAATTATTGTTGCAAAAGATTAT

AGTTTTGAAACTTCAGCCCTAATTGACTCAGGAGCTGACTTGAATTGTATCAATGAAGGG

TTAGTACCTTCAAAATATTTTGATAAGACTACTCAAATATTAAATACAGCAGATGGAAGT

AGATTATTTATCAAGTATAAATTATCCAATGCTTCTGTTTGTAATAATCATCAATGCCTT

GATACACCATTTATTATGGTAAAAGGATTAAGCCAAGCGGTAATACTTGGAGTTCCATTC

CTTACTTTGCTATATCCTTTGACCATAGATCAAACAGGAATATCTTCAACTATTCAAAAT

ATACCAATACATTTTCATTTTATCCATAAACCAAAATTAAAAGAACTAAATAATGTCAAG

AGTCAGTTACAGAATAAAGAAAAATTTCTTTGCACTCTAAAACAAGAACTCAAATATAAG

TCTATTGAAGAACAAATAAATACTCCTTCAATTCAACAAAAAATCCAATCAATACAACAC

CATATAGAAACTTCTATATGCAGCGAGCATCCCAATGCTTTTTGGGAAAGGAAAAAGCAT

AACATTAGTCTACCTTACGAACAAGGATTTGATGAAAAACAAATACCTACAAAAGCTAGA

CCAATACAAATGAATACTATCTTATTAGATATGTGTCGCAAAGAGATTACTGATCTCCAA

AATAAAAAGCTCATAAGACCAAGCTCCTCACCTTGGAGCTGTGCAGCCTTTTATGTTAAT

AAGAATGCTGAAAAGGAACGAGGAGTTCCTAGGTTAGTTATAAATTATAAACCTTTAAAT

AAAGTTTTAAAATGGATCAGATATCCTATACCTAATAAAAAAGATTTATTGGATAGAGTT

GCCCAAGCTTGTATCTTTTCAAAATTTGATATGAAATCTGGATATTGGCAGATACAAATC

CAAGAACAAGATAGATATAAAACGGCATTTAATGTCCCATTCGGACAATATGAATGGAAT

GTTATGCCATTCGGCTTAAAGAATGCTCCATCAGAATTTCAAAAAATAATGAATGATGTT

TTTAATTCATACCATCAATTTATGATAGTATACATAGATGATGTTTTGATTTTCTCTGAA

TCCATAGATCAACATATTAAACACATTAATACTTTTATACATGCAGTCCAGAATGCTGGA

TTGGTAGTCTCAGCAAAAAAGATTACTTTATTCAAAACCAATATTAGGTTTCTTGGCCAT

ATGATTCATCATGGAACAATTATTCCCATAGATAGATCAATACAATTTGCTGATAAATTC

CCTGATCAGATACTTGATAAAAAAGAACTCCAAAGGTTCTTGGGATGTTTAAACTATGTT

AGTGACTTTTATCCTAATCTTGCTATCGATTCTAAGCCTCTATATGATAGACTCAAAAAG

AATCCTCCACCATGGACCAATATTCATACTCAGGCAGTACAAGTAATCAAACGAAAGGTA

AAGACTCTTGACTGTCTTTCTCTTCCACATCCAACTGCTTTCAAAATCATAGAATGTGAT

GCTTCAGATATCGGTCTCGGTGGTATTCTCAAACAAAAGATTGATAATAAAGAACAATTA

CTCAGATATACTTCTAAGGTTTGGAATTCCACACAAAAGAACTATTCAATGGTCAAAAAA

GAACTTTTAGCCATTGTCCACTGTGTTTCTAAATTCCAAAGTGATGTTCTTAATCAACCA

TTTCTTATACGAACTGACTGCAAAGCAGCCAAAGATATTTTACAAAAAGATGTTCAAAAT

TTGGTTTCCAAACAAATCTTTGCTCGATGGCAGGCTATATTATCCGCCTTTGATTTTTCC

ATCGAACACCTTCCCGGGTCTTCCAACTCACTACCTGACTTTTTAACCCGTGAATTTTTG

CAGGGGGGCAACACTGACACAAACGACGAATCACCACTGTCCCTGGCATTACAGCTGTTC

GAGGAGAATAGAAATAATAATATTTCATTTTTACCTTCACCTAGTCCAATGGGTTCTCGA

GGCACTTCTCGCCGAGACAAACAACCCATGTCAAATATAATACCATTTGAAGGGGAACCT

TCGCCTTTGGCAATTCGTTCACCTTCCCATTCTCCTGTTCACCAAATCACCTATCCCGAA

GCAACTGCTTCAAAACCCATATATTCTCCAGCTCAGACCAAGTCTGCTTCACAACCCAAT

ATACCCAGGTCTTCTTATATAGAAAGAGCTTATCTCATGGATATTACTATCGATGAGAAA

CATGTTAAGACAGAACCAATAAAACTTGCTCCTTTATATTTGCTCAATCATCTCAACCCA

TCCAATGGTGTCAAAACCAGAGAATACTTTGAAGCCATCCTAATGGAAACGGGATCCATT

TCCGTTGTCCACTATTATCAACAGAATACCAGGACCATTGCATACTCCAAGGTCCAAATT

AAAAACTTACTTTCCTATGAAGAGTGGGGTTGCAATCCCGCGCAAGGAAAGAAACTTCAC

AACCCAGATTTTTCTTATAAATATTATAAGTATGCCGAATACATTGAAGCTTGGCATAAG

TTTTTCTATTACCAAAATCCAAATAACACACACAGCTGGTACATTCAGTTCCGGCTCCCT

ATTTTCGAAAGATTCCCCACATGGTTTAAAAATTGGTTTTTTGTGTGGGGCTCAACATCT

CAAATATTACCTCCTGCTTTCCAAACAGTGCAGGAACAATTCAAAGAATATAACCATGCT

GATAAAACCATTGATATCCATTTATATTTCATGGCCCTATACCAAATCCCTTGGATTATG

AAATGGGATTATTCAATCTCAGTTGATACCACTGCTGACTATTCCTATATTCCCAGACTT

GGAAAAACTATCTCTGTAAAGTGGTGGGACATATTCAATATGGACCCTACAACACTCACT

CCCAAAAGAATACCTCAGCAAACCTCTGAGAAACAACCAAGCCCTCATACTGCTAATACC

TCCTTTGATTTCCCAACCTATTTAAGAAATATGCTCCAGCAAAACCCCAATGCTTCAAAA

GAGACCTTAGAAAACCAAATGAGAGAAATATTTGCTGAAGCTGGGAAGAAACCAGTTTCT

TCTGAAGAAACTGTACAATCATCTCCTTTACACACCAACCAATATCTGAATCTCCCTGAA

GAAGATCCGGATGAAGAAACAGAACCAGAGGAAGACGATCCGGATGAATTCAGTCAAAGA

CCATGACCAGGAAAAATCAATAATTGAAGAAAGACTCATCAATCATCATCACCGACAAAG

TATAAAGCATCACCGACAAAGTATAAAGCATCACCGACAAAAAGCACCATCAACAGTCAT

CACCGACATATAAAGCCAAAAGAAGATGCTGTCACTGTAGCCATTTGTATATACGCCAAA

AATGTCACTGTAGCCATTTATATATACGCCAAAATGACACTGTAGCCACGATAGGAAGCG

ACAAAGGGAAACACTGTTCCCAGATGCTTCTTTTTGTCTTCTTTTGTTTTTGTTTTACTT

CTTTACGTCCTCTCATCTCGCTTTATGTCTTCTC

>AtrichBV_sc2 [endogenous-virus-name=Amborella trichopoda B virus] [strain=sequence cluster 2] [host=Amborella trichopoda] [moltype=genomic DNA] [note=complete genome] 8530 bp

TGGTATCAGAGCCATACAGTAAAACTGTCACAGTTACAACAGTAGAACTGTCCAACAGTG

AAACTGTGTAAACAGTTAAACTGTCAAAACAGTAAACCTGTTCTTTTTACATCAATAAGT

TGTATGAGATAAAAGTTACATCTTGACTAGCGAAACTAAGTTAAATTTACATCCTAGAAA

GTTGGTCCGAGAGTTAAGGCAAGAGACAAGTGAACGCCTATTGCAAAGTACCGCCAAGGC

CAGGAAGAGCCGCTTAGGGAAAACGCTGGGCCACTGAATGTCCAGATTGTCATTATCCGC

AACCTACGGATGGCAAATTTAATAAGGAATCTAACTAGTAAAAATGCAACTTCATCTCAA

ACACTTAGAGTAAAAAATAAAGAACAAATAGTCATTAATGACATCGAACATAATATTCAA

AATTGGAACATACCTAAGGTACCAATCCATGACGTTTACAATAAAGGATCATTCAAATTC

AAGATGGGTTATGCCATCAAGACTGTAGAAGAAAGTTTATCAGTTACTCAAAACCAAGAA

GACTTTCACCTACTCTCAAAGAAAATCATTGAAAAACATAAAGAACATAATTACAAATAT

TTACATATTGGATTAATTCAAATAGCCGTGAAACCTTTGACCCGATTAGGGTTGAACACC

TCTGTGCTTGTATGCCTAAGAGATACAAGACACAATATTTTCACAGACTCGCTACTTGGC

ATGGTTGAATCCAGCCTCTGTAGTGGTCCCATATATTTTAATTGTTTTCCAAATTTTTCT

GTTTCTTTGACTGACCCAAATATTTTAGATATATTAACAATTAATTTAAAAACACATGGT

TTTAATATGATCCAAGGTTCTCAAAACTTAGCAATAGTTTATAGAATATATTACAAAGTC

ATGAATACCTTATGTCCAAATATTATGCAAATTGATCAAAAAGATCAAACTATATTATTC

CAAACAAATATGGAAAAATCAAATATGATTGTTCCCAAAACAATAAAATGGTCAGATATT

AGACTACCACAGTCATGGGCATTACAACATGCCAATCAACCTCAAGAAGTTCCCCAACAT

CATATTCCTAATCAAATAATAGAACATCCTGATGGTAATATTGAAATAAAATTTCAACAA

TTACAAGTTCAAGATGATATTAATATTAATAGAAAAAGGAATATTAAAAATAAAATACCA

AGTAGAAAATCTTATTCTACTAATCCGAATGTCATAATACTACCACCTCCTACTCAGTCT

TCTTCAATACCTAGTTCAGACCTACAATTTCAAGGAACAAGGAGAACAACAGATCATGTT

GTTATTCCCACATATACTCCTGAATCTAGTACTACAAATACTACTGAACCTAGTATAGAA

GAATTACAAGACTTTCAAATATTAACTTTACAAGTAGAAGAATATGACAATTCACATGAT

AAATATTCAGATGAATATATAGCAGCATATTTAGCCGCTAAACTAGAAAGAAAAAAGAAA

AAAATTGATCATAATTATATCAAGCAAGATTTTATGTCAGAAGAAAATAAACATATTAGA

CAATGGTATCTTACAAAAGTACCATTACAATTAAGAGAAATATTACAGGCTAAATATAAT

AAATTCCTTAAAAAGGAAAAGGAAACAGAAGGATATTTTCTATGGTTAATAAGTAATTAT

CAACATAAACTTCATATCCCTTCATTTGATGAAATTCAAACAATAGAAGAGAAAACTTAT

ATTAGTACGGATGGTACTACAACTGCTAGTACTCACCCTCCTGAAAAAAGTCTAATACTC

AAAACATCTATCCCAATGAAAGATGAACCTGCCAAATCTCCATCAGTATATGATATAATT

GCTACTCCATATAAAATCTTACCCAAAGAACTTGGTAAGAAACAAGAATTGATTCCTCAT

ATTGTGGAACAAAATAATTATACCAATCTTTATATGCAATCTTTAGGAAGACAAGCAGAA

AGAATAGAAAGACTTGTCACCAAAGAACAACCTTCTGCTACAATCATACCTAAATCAGTT

TTGTTCAAACCACCTCCAGAATTAAATAAGAATAAATTTCATATTGGAGATAATAATAAA

TCCCTTATGGATCAATTGATATCAAAATTATCTCAATTAGATATTAAAGGAATACCTTCT

CCAAAAATAACTATTCCTCCTCCACCTATTGTCAATATACCACCTGAATCCTCTGATAAA

GGCAAAGCAAAAATACCTGAACATGAATCAATGATGCTTCAGGAAGAAGAACCAATCATA

GAAGATACTATCCAAAATCAAGAAAAAGCATTTATGGATACTACCCCTATTTCTGAAACA

ATAAACAGAATAGCCTATGGAAAATATTGGAAACCAATAACAGCTTTACCATATCATAAG

AGACCTACACCTGTTGATATACAAATGGAAGAAGATGCCATACAACAGGCAGTATCATAT

TCAGGAAGATCAATAGTAAATTGGCAAATAGATGGATTATCTGAATTAAAAATATTGGAT

GTCATGAGACATATGACAATGTATGCTTCTGCAAATAAAATGGCAGGCAACCCTGATCAA

GAGGTTGCAAAAGCAATAGTAAATGGATTTACTGGCTGGTTACGAGGATGGTGGGATTTC

TATGTAGATGATGAAACCAAACAAAGAATCCTCAATAATACTAGGCAATGGACCGCCAGA

AATGAACAAAACCAAGAAATAATATACACAGAGAGTGATGCTGTCAACACTCTAATATAT

ACCATTACACTTCATTTTGTAGGCAGTACAGCTTTACTAGCAGCAAGATCGGAAGATCTG

CTATCTAATCTACGCTGTCCTACCCTATCTCATTATAAGTGGTATAAAGATGTATTTTTC

TCAAGATTATTAATAAGAGAAGACTGTCAGCTAGACCACTGGAAAGAGAAGTTTATAGCC

GGTCTACCATATTTATTTGCTGAAAAAGTCAAGGAAAGACTAAGGAAAAAACATAATGGA

ATTATTGTCTATTCATCATATACACTCGGAGAACTCTCTGCAGAAATATGTGCTGAAGGT

TTAGCCTTATGCACAGATATGAAATTAAAAAGACAATTAGATAAACAAAAAATCACTAAG

CGAAAAGAGCTTGGTGACTTCTGTGAACAATTTGGTTATTACCTTGATAAGAAAAATATG

CCTGACAATCTATACAAGAAAAAGAAGAAATACAAAAAAGAAAAATATAAATCCAAAAAT

AAGGATAAAGATAAGAAATATTATAAAAAATCTAAAAGGAAATTAATCAATAAATTTTCT

CCCAATACAAAGAAAAAGAGAAAGTTGCCAGTATGCTATAAGTGTGGTAAAGTAGGACAC

TATAAACATCAGTGTAAATTAAAAAAGAAAATAGAATCATTAAATCTAGATGAATCAATA

AAATCTAAATTATATAACATATTCAATTATGATTCTTCATCTTCTACTTCTTCTGATTCT

CATATCGATCAAATCCAAGATGATGATCTCATGTGGACAGACTCCTCTGACTCCACTAAT

GAGTCAGATACAAATTCATCCAGCTCAAAGGATGATAATTGTAGTTGTCCAATATCAACA

CATAATGTTCACACACTTACACAAGAAGATAATTTCATTCTAGATATTATTGATAAAATT

GTTGATCCAGAGGAAAAAAGAGCTGTCATTACTCAATTTATTAATATTACAAAAAATAAA

GCAAAACCAATTCCTCTGAATAAAACCCCTGATGAATATAACTTTGCTTCAATAATGAAA

AGAGTAAGTGATCAACACACTTCTCAACAAAAAGAACCAACCATTTCAGATCTCAAACAA

GAAATCAAAGAAATAAAGACTGAAATAGTTCAATTAAAAAATAGAATTAAATCATTAGAA

TTACAACAAATGTCTCAACAAGATTCAGATACAGATGAAATAGACTTTGATGAATTAATG

AAAATTAATCCATCATTAGATATCCCTGAATCATCAGAAGCCAAAATAATACCTCTTCCT

TCTGTGAATCTAATAGACAAAATAGTAGTTCAAAAATGGTTTGTCTACGTTAAGATAATT

GTTGCTCAAGATTATAGTTTTGAAACATCAGCTTTAATAGATTCTGGAGCTGACTTAAAT

TGTATCAATGAAGGACTCGTACCTTCAAAATATTTTGACAAAACTACTCAAATATTGAAT

ACAGCAGATGGAAGTAGATTATTTATTAAATATAAATTATCTAATGCTTCTGTTTGTAAT

AATCACCAATGTATAGATACACCATTCATAATGGTAAAAGGATTAAGCCAAGCAGTAATA

CTTGGAGTTCCATTCCTTACTTTGTTATATCCTTTAACCATAAATGAAACAGGAATATCT

TCAGTTATTCAAAATATTCCAATACACTTTAATTTCATACATAAACCAAAATTGAAAGAA

CTCAATAGTGTCAAAAGTCAATTGAATAACAAAGAAAAATTTCTTTGTACCCTTAAACAA

GAAATTAAATATAAAACTATTGAAGAACAATTACAAACTTCTTCAATACAACAAAAGATT

CAAGCATTACAACAACATATAGAAACTTCAATATGTAGTGAGCATCCCAATGCTTTTTGG

GAAAGGAAAAAGCATAGTATAAGTTTACCATATGAACAAGGATTTGATGAAAAACAAATA

CCTACAAAAGCTAGACCAATACAAATGAATACTATCTTATTAGATATGTGCCGAAAAGAG

ATTGATGATCTCCAACAGAAAAAACTAATAAGACCAAGCTCCTCACCTTGGAGCTGTGCA

GCCTTTTATGTTAATAAAAATGCTGAAAAGGAACGAGGAGTTCCTAGACTTGTTATTAAT

TATAAACCTTTGAATAAAGTCTTAAAATGGATAAGATATCCAATTCCCAATAAAAAAGAT

TTATTAGATAGAGTTGCTCAAGCATGTATCTTTTCCAAATTTGATATGAAATCTGGTTAT

TGGCAAATACAAATCCAAGAACAAGATAAATATAAAACTGCATTTAATGTTCCATTTGGA

CAATACGAATGGAATGTTATGCCATTTGGTTTGAAAAATGCTCCATCAGAATTCCAGAAA

ATAATGAATGATGTTTTTAATGCTTACCATCATTTTATGATAGTATATATTGATGATGTA

CTAATATTCTCGGAATCGATAGATCAACATATCAAACACATTAACACATTTATACATGCA

GTCCGGAATGCTGGATTAGTTGTTTCAGCCAAAAAGATAACACTATTCAAAACCAATATA

AGATTTCTAGGACATATGATTCATCATGGAACTATCATTCCCATAGATAGATCCATACAA

TTCGCTGATAAATTCCCTGATCAAATACTTGATAAAAAGGAACTTCAAAGGTTCCTAGGT

TGTTTAAATTATGTTAGTGATTTCTATCCTAACCTTGCTTTAGATTCTAAACCTTTATAT

GATAGACTCAAAAAGAATCCTCCACCATGGACCAATATCCATACCAAGGCAGTACAGGTA

ATTAAAAGCAAGGTAAAGACACTAGACTGTCTCTCCCTACCTCATCCAACTGCTTTTAAA

ATCATAGAATGTGATGCTTCTGACAAAGGCCTCGGTGGTATTCTCAAACAAAGAATTGAT

TCTCAAGAACAATTACTTCGTTATACTTCCAAAGCTTGGAATACTACTCAATTAAATTAT

TCCATGGTTAAGAAAGAACTCTTAGCAATTGTCCATTGTGTTTCAAAATTCCAAAGTGAT

ATTTTAAACCAACATTTTCTCATACGAACCGACTGCAAAGCAGCCAAGGATATTCTGCAA

AAAGATGTTCAAAATTTAGTTTCTAAACAAATATTTGCTCGATGGCAGGCTATATTATCC

GCCTTTGATTTTTCTATCGAACACCTTCCCGGGTCTTCCAATTCATTACCTGATTTTTTA

ACCCGTGAATTTTTGCAGGGGGGCAACACTGACACAAACGACGAAGAATCACCCTTATCC

CTTGCTCTACAATTGTTCGCAGAAAATCAGAATAATACTAATATTATACCTTCACCTTAT

TATCCAATGGGTTCTCGAGGCACTTCTCGCCGAGATAAACAACCCATGCAAAATATTGTT

CCATATGAAAGGGAACCATCGCCTTTGGCAATACGGTCACCTTCACAGTCACCCATTCAC

CAAATTACCTATCCAGAAGCACATGCTTCTACTCCTATATATTCACCAGCTCAATCCAAG

ACCACCTCCATACCTAATACATCTAGATCTGCATACATCGAAAGAGCCTATCTAATGGAT

ATCACCATAGATGAACAACATATTCATACAGATCCCATTAAACTTGCACCTTTGTATCTG

ATAAACCATCTCAACCCTACCAATGGGGTAAAGACCAGAGAATATTACGAAGCTATACTC

ATGGAAACAGGATCTATCTCTGTTGTCCATTACTATCAGCAAAATACCAGAATAATTGCC

TATTCCAAGGTTCAAATAAAAAAGCTTCTTTCTTATGATGAGTGGGGTTGCAATCCCGCA

CAGGGAAAGAAATTGCACAACCCAGATTTCTCATACAAATATTATAAATATGCCGAGTAT

ATAGAAGCCTGGCACAAATTCTTTTATTATCAAAATCCGAACAACACACATAGCTGGTAT

CTTCAATTCCGGCTACCTATTTTCGAAAGATTCCCCACATGGTTTCAAAATTGGTTCTTA

GTGTGGGGTTCAACTTCTTTGATACTACCTCCTGCTTTCAAAAACCTGCAGGAACAATTT

GTGCAATATAATAATAGTGACAATACAATTGACACTCACCTATACTTCATGGCCTTATAT

CAGCTCCCATGGATAATGAAATGGGATTACACCATCTCAGTCAATAATACCAATGACTAT

ACCTATATTCCTCGATTGGGGAAAACCATCTCTGTTAAGTGGTGGGACATTTTCAAAATG

GATCTCACTGCTACCACACCAAAAAGAATACCCCCACCAATACCTAAATACCAGCCAAAC

CCAAATACAACAAACACCTCTTTTGACTTTCCAACCTACCTTCGCAACATGTTGCAAACC

AATCCCAATGCATCCAAAGAAGAATTAGAAAAACAAATGAAGGAAATCTTCGCTGAAGCC

GGAAGAAAACCAGTCTCCTCCGAAGAAACAGTACAATCTTCCCCACTTCACACCAATAGA

TACCTCCAATTGAGTGAAGATGATCCAGAAGAAACGGATCCAGAAGAAGATGATCCAGAA

GAGTTCAGTCAAAGACCATAAAAGTCTCTATCATCATCACCACCGACAATACCACCAATA

ATCCAAAAGATAATAGTCTCCACCGACAAAGCTCCATAAAAGACAAAAGCTCACCGACAG

AAAAAGACAAGAAAGACTGTTCCAGATTCCAAGCACTGTACCCACCGACATACACGACAA

AAGACATGGCCACATCACCGACAAGAAGTACTATTCCAGATGCTTCACTGTAGCCACCAG

CATAAAAACCAAAAGTGTTACTGTAGCAATAATCAGATACGACAAAGAGGAATACTGTTC

CCAGATGCCTCTTTTTAATTTTTTGTTTTCTTCTTCTTTTGTCCTACTCCTTTGGGTCAC

CTCAGTCTCCTTTACGTCTTTTCCCTTTCCTTCTGCTTTATCCTTTGCTTCGAAGGGAAG

CAAACTCCTTCGTCTATAAAAGGGCCAAAGGCCTCTCATTTGAGGCAAGGTCTTCCGCTT

CCCCTCTCAGCTCATAAACTCTTCTGCTCTCTCCCACCTCTCCCACCTTCTCCTCTTTAC

TCAGAAGCTCCTTCACTTCTCCTTCTTTCTCTCAAGACTTCTACTCTGATCTTCGACCTC

CTCTCCTACTTCCTTTCTCTCTCAGGCCTTCTTCAGATAAGATTCCAGTTCTTTCTAATC

TCTTTTCTCTCACTGTTCCTCTGAGCCCGAAGTGTTATACCAACCAGAGCTATCCTGACC

TTCATAGCTCTCGTAGTTCAAACATCTCACCAGGTTTTATCTTATACTTTCACCTTTTAT

TTTTATATCCATTATGTGTGACTGCAATCTAAATTCTCTCCCTTCCTACTGTCCACAACA

CTGGAAACTGTTTTCCAGTTATTGTATTTGCAAACAGTAAGTTTTTTCAAGTGTGCCTCT

TATCTGTAATTTCTCAATTTCAGTGTGCCTCTTATCTGTAATAAGTTGTAATCTGATTAT

CTGTAAAATATACAGTTGTTATACAGTTACTTTATGATGTGAATAAAATGCAGTTTAAGA

TTTTATTTAGTATAAAAGTTGGTGAGTTTAATTATGCCCAATGGTTTGTAATTGATATTT

CTATTATTCCTTTATTGTGCATAATCCTGTTTATCCTTAATTATTATAAGATTTTACAAG

AACTAGTTAAATACAATAGCTTATTGTTTACCTATTTACTATAAAAAATTCCTGCATACA

ATCCCTGCATAAAATTAGATTATAAAATTAAAATATCTTTGCATAACAAAATCAATTAGA

TTCAACCTAT

>AtrichCV [endogenous-virus-name=Amborella trichopoda C virus] [host=Amborella trichopoda] [moltype=genomic DNA] [note=incomplete at 5’ and 3’ ends, mutations in ORFs 1 and 2] 6022 bp

TTACGGATAATAGCTATAATCAGTTTACTGATTCACTATTGAGAATAGTAGAATCAAGTC

TATGTAATGGTCCAATTTATTTTAACTGTTTTCCCAATTTTTCTGTATCTTTAACATACC

CAAATCTATTAAAAGTTCTCACGTTAAACTTAAAAACCCAGGGTTTCGACATGAACCCAG

GATCTCATAATATAGCATTAATATATCGAATATGCTATAAAGTAATGACTACTCTTAATC

CAAATGTAATACAGAGAGATACTAAAGGTGAGACAATCTACTTTCAAACAAACACTGAAA

AGTCTAACACTATCATACCTAAAGCAGTCAATTGGTCTCAAATAGAATTACCTAAAAATT

GGTCTTTACAGAACGAAAATCAGCAATTCAAAACTCATAAAAATAATACCGTTAAACAAA

TTATCGAATATCCAAACGAAGAAGTAGAAATACAATTTAATACTAATAGAAATACTAGAC

TTAGAATCTTAGATGACAATGAAATTAGACCTATTCCAAGGAGAATTATTCCTTTTAGAA

ATTCTACTTCATCAATTAATGAACCTATAATACAGGGATTATCCAGAACTCAAAGTAATA

TACAAAGACCTGTATATAAACAAATAGATCATTCCAAATCTAAATCGTCTTTACCCGACC

TTGATTTTAATATTATGACTCTACAGAAGGAAGAGAAGGTAAACATAGAATATTTAAAGA

ATGATTATTTATCTGAAAAAAATACTACCCTAAAAAATTCGTATTTAAAATTTTTTAAGA

AAGACTTAATAGATGCATGTCATAAAAAATACAATAAATTTCTAAAAATAAATAGAAAAA

ATATAGGTTTTTTCGACTGGCTAGTTGCTACCCTTAAGGAAATAGGTTATCAAACACCAG

AATCAGAACTAAATCCTGAACTTAACACGTTAGAAAATCAGTATAAAACTTACCTTACTA

CTGAAGGAAATGAAATACAACATATTCATCCCCCTGAACAAACTCTTGTAATAAAAGGAA

AATTATAAACAAAAGCTACACCCTATAAATTAATCCCTGGTGATCATACAAATAAAGTTG

AAACTATTCCTCACATAGTAGAACAAAATAATTATACTAACCTATATCTGAAATCTTTTG

GCAAGCAGGCCACACGAATAGAAAAACTGATTAGTCAACCCCAAAAAATACAAGAAGAAA

AGAAAAAGGCAGTTATGTTCAAACCACCTCCTACTATAAATAAAGATAGATTTGTCTTAA

AAGAAGACACTAAAGAATTAATGAATGAACCGATAAAAAGATTATCTCAATTAAATAAAG

ACCCAAAAGGAAAAACAAAAATAGTATAATCAGAAACCATGGTTCTACAAGAAGAATCAA

TAAATACTGATAATACTGATATAGAGATATCACATATAGAACAAGCCATTAAAGAAGAAA

GTTTAAATATTAATAGGATAGACTATGGAAAAAAATGGAGACCAGTGTCTCAAGCAAGAA

CGTATCATACTCTCAGCCCACGCCAGTAGATATACAATTCGAAGAAGATTTAATATCTCA

TACAGTTTTCTACTCAGGACGAACTATAGTAAATTGGAACGTAGATGGAATGTCAGACCA

CCAGATTTTAGATACATTGAGGCATATGACGATGTATGCCACAGTATGTAAAGTCTCTAG

TAGCCACGATCAAGATGTTGCTAAAGCAATTGTCTGCGGATTTACAGGCTGGCTTCGTGG

CTGGTGGGATTTTCATATGTTTGAAAATACAAAAAACCAATTATTAAATGCAACTCGACA

ATGGGTTGTAAAAAATGAACTAAATTACCATATGGCGAAAGCGATGCGGTAAATACACTT

ATCTATAAATAACCTTACATTTTAGAGGTAACACACTGTTATTAAAGGCTAGATCTGAAG

ATTTGCTTTTTAATTTGAGATGTCCTGCGCTTTCATATTATAAATGGTATAAAGATGTTC

TTTTCTCTAAACTATTATTAAGAGAGGCTTCCAGTGCCGATCACTGGAAAGAAAAATTTA

TTGCTGGTCTACCATACCTTTTCGCTAAAAAGGTCAAAAATAGGTTAAAAAACAAAAATG

GCAGAATGATACCACATTCACAATATACTTTAGGAGAACTTTCTTTAGAAATATGTGCTG

AAGGATTATCCCTTTGCACAGATATAAAATTAAAAAGACAATTAGATAAAATACGAATGA

CTGGAAAGAAAGAACTAGGAGATTTTTGTGAACAATTTGGTTTTAGTATTGTTCCTTCTT

CTAGTAAAAAGCACAAGAAAAAAGTATATAAACAATATCCAAAGATAGAACTTCAAAAAA

TTTCAAAAACATAAAAAAGAAAAACGAAATAAGTTCATACAAAAATTTGCTCCAAAAGAA

AAATTTAAAAGAAAACTCCCAGTATGCTATAAATATAGCAAAGTTGGCCATTGCAAAAGC

AATTGCCGTGTGAAAAAGAAAATAGATACCCTAATGCTTGATGAAAATATACGAAATCAA

TTATATGATATAATTCAAGTAGTTGATACTTCTGATAGCTCTTCTGACGACACAGATACT

GAAACATTAATAGGAAAAATTGAAGATGAAAACTTGATTTGGTACACTTCTGATTCAAGT

AAAGATTTAGATTAAGATATAAAAATGCATGATTGTTTCTGTAATGTACTATCAAAAGAA

GAAAATCTAATACTTGATTTAATAGACAAAATTTCTGACTCTGATGAAACAAGAGATGCT

ATAACCCAATATATCCAATTAGCTAAAGGAAAAAATAAAACTCCTCAATTAATAAAAACC

CAAGAAGAGTATAGTTTATTTGAAATATTAAGAAGAATAAATAAAAGAGAAACTATACAA

AAAGAACCTACCATTGTTGAATTAAAACAAGAAATAAACGAAGTAAAAACTGAAGTTCAG

CAATTAAAAAATAGAATAAAAATATTAGAAATGGCCACAGATTTTCCTGAAAACGAAACT

GATGATGAACTTCAATTCAATGACCTAATAGAAATAATAAAATAATAGCATCCTCAGATC

GTCCTGAATCTTCTGGACTTAATCCTGAATCTTCTGGACTTAAAATTGAAGAACTAGAAA

ACCATTTGAATTTAATAAATAAAGTAATCACTCAAAAATGGTATACGTTAATAAGAATAG

TCATTCAAAATGAATATAGTTTCCAATCACTTGCTCTAATAGACTCTAGAGCCGATCTAA

ATTGTATCAATGAAGGACTAGTTCCTACAAAATATTTTTGACAAAACCACTCAAATTCTA

AATACTGCCAATGGTAGTAAACTCATAATAAATTATAAACTATCTGATGCAGTTGTTTGT

AACAATAGAAATTGCTTCGATATGGCATTTATTATGGTAAAAGGACTGAGTCAAACAGTA

ATATTAGGAACACCATTCTTAAGCTCACTCTATCCCTTAAAAATAGATAATGCGGGCATA

TAAGCCAATTTAGATGGTCATCCTGTTAAATTCAATTTTATTGACAAACCAAAAGTTAAA

GAAATAAATAATGTTAAACAAACTATTTATAATAAAGAAAGATTTCTTTATCATCTTAAA

ACAGAAATCAAATATAAAACATTAGAGCAGCATCTTTTGCTTCCATAAATCAAAAAGAAA

ATTGAGCAATTAAAAATAAAAATAGAACGAGAAATATGTAGTAAATTACCAAACGCTTTC

TGGAATAGAAAGAAGCACATGGTAAGTTTACCCTATAAAGAAGGATTCTCAGAAAGAAAA

ATACCTACTAAAGCTAGGCCAATACAAATGAATACAAGATTACTAAAAATTTATAAAAAT

GAAATAGATGAACTCCTTAAAAAGAAGTTAATTAGGTCAAGCTCTTCTCCTTGAAGCTGT

GCTGCCTTCTATGTAGAAAATGCAGCTGAAAAGGAAAGAGGAGTTCCAAGATTAGATATA

AATTATAAACCTCTTAATAAAGTTTTAAAATGGATTCGATATCCAATACCTAATACAAAG

GATTTATTAGACAGATTAATTAATGCTTGTATCTTTTCAAAATTTGACTTTAAATCAGGA

TACTGGTAAATACAAATAAATGAAAAGGACAGGTATAAAACAGCCCTTAATGTTCCATTT

GAACAGTACGAATGGAATGTTATGCCATTCGGATTAAAAAATGCTCCTTTTGAATTTCAA

AAAATTATAAATGATGTATTCAACCGTTATATAAAATTTATGATTGTATATATAGATGAT

GTGTTAATTTTTTCAGAAGATATCGATCAGCATTTTAAACATCTGAATACTTTTCTGACT

GCAGTAAAAAATGCAGGTTTAGTAGTCTCTGCTAAAAAGGTAAATCTATGTCAAACAAAG

ATCAGATTTTTAGGTCACAATATATTTCAAGGAACTATTGTCCCAATAGACAGAGCAATT

GAATTTTCAAATAAATTTCCTGATATAATTCTTAATAAAACAGAATTACAAAGATTTTTA

GGATGTTTAAATTACATTGCCGATTTTATAAAAGATCTAGCTATTATCGCTAAACCTTTT

TATGAAAGACTTAAAAAGCATCCTCCAAAATGGACTGATCAGCATACAAATGCTATTAAA

ATACTTAAGAGCAAAGCAAAGGAACTCCCTTGCTTAGTTCTTTCTGATCCAAATGCTTTT

AAGATCATTGAATGTGATGCTCCTGAACTTGGACTAGGTGGCATATTAAAACAAAAAATC

AATGAGCATGAACAATTAGTTCGGTATACTTCTAAAGCATGGAATAGTACTCAACAGAAC

TATTCTACTGTAAAAAAAGAAATACTCTCTATTATTCATTGTGTCACTAAATTTCAAAGT

GATATTCTTAATACTCCTTTTTTAATTCGAACCGATTGCAAATCTGCACGTGATATTTTA

GTCAAAGATGTCCAAAATCTTGTTTCCAAACAAATTTTTACCCATTGGCAAGCCATCTTA

TCCTCTTTCGATTTTACTATTGAACATCTTAAAGGTTCTACTAACCATCTTCCTGACTTT

CTCACGCGTGAGTTTTTTGCATAGGGGAAGGGCTCCTCCAGAACTTGAGATAGATCAGCA

GCCATTAGTTTTAATACTTTTCAATAAAAATAAAAATATGACTTATACAAATAAAGGCAA

AGCCCCTATGGATATTGTTCCATCATCTCCCGGATCTCCATTAACCTGTTATCCACCAGT

TTCCCCAAATAGACCATTAGTAATATATCCTTATTATTCTACCCAAATAAGTCAAAGCCA

GACCCCAGTTTTTATACAATCTCCAATCCTAAATAAAAAATCTAGTACTAGGAAATCTCA

ATATGAAGAAACTGCTTTCCTAGCAGATTGTATCATTGAACCTGAACACTTGACTCTAGA

ACCTGAAAGAATAGCCAATCTCTACCTTTTAAATCATCTTAATCCTTCTAATGGAAATAA

GACAAGAGAATGATATGAGGCCATTTTTTTTTAGAAACCAATTCTATCTCTCTGGTACAT

TTTTACCAGAAAAATACAAATATCATTGCTTATTCGAAATTCCAAATACGCAAAATATTA

TCATACAAAGATTGGGGTATTGACCCTCTACGTGGGAAAAAGTTACAGGCACCAGAATTC

CAAAATAAATACTTCAAATACTCGGAATATATAGATGCCTGGAATAAAGTTTTTACCTAC

CACAATAATAACAATTCTCATATCTGGTTTGCTCAGTTCAAGATGCCTATCCTTGAGGTA

TTTCCTAATTGGTTACGAGAATGGTTCTTAACCAGGGGATCAGGTTCTCAGATCCTCTCA

GAAACTCTACAAGTCTTGCAAGCGACATTTGTAGAATTTAATGGAAAAGATCAAATACCG

ATAACCATTTATATTTCATGGCGATTTACCAAATCCCATGGATTATAAAGTGGCAATACG

GAATATCCGAAAATACTGTCAATAATTATGAATATGTCCCAAAACTTGGGAAAAGAATAT

TTATTAAGTGGTGGGATATTTT

>AlyrV [endogenous-virus-name=Arabidopsis lyrata virus] [host= Arabidopsis lyrata] [moltype=genomic DNA] [note=incomplete at 3’ end, mutations in ORFs 1 and 2] 7445 bp

TGGTATCAGTCGCTAAAACGATCGATTTTCAGTTTTCAAAAATAAAGATTTCATTTTAAA

TTTTAAAATCAACTTTTAGAAAGAAACGGACCTCTTCTACCGAGACATTGAAGCCAAAGG

TGTTCCAGGCAGCAAGTCCTCAGAAGGAAGGCAGTAAGACCGTTGGTAAACCGCAGCCGT

GTCAAAGTAAAACAGGTTAGTCTCTTGCTAAGATGTATTTGATTTAAAAAATTTAAAATG

GCATCCTCATTTAGAAAACTGTTAAAATCCGGCCAATCATCAACTTCTAAATCAACAATA

AATTCAAGAGAAATAATATCAAAAATATCAACATTTAATAAAGAAATATCAAGGGATTCC

TTACCACATATTAATGCTGAATATATATATCAAATCGGAACATTTGATTTTAAAACAGCT

TATTCAATAAAAGAACATGAACAAACAATATCTCTACAACATGAATTCGAAGAAATTAAA

TTATTATCATCAATAGCTCTAGATAAATATAAGCAAAAAGGATTCAACTATCTTCATTTC

GGATTAGTTCAAATAGCAATAAAACCATTATCAAGAACATGAATTGATTCACCCATTTTA

ATGTTATTAAGAGATAAAACCCTTCTAAAATTTGAAGACTCACTTCTTGGTGTAGTACAA

TCAAATTTATGTAATGGGGCAGTCTACTTTAATTGCGCACCTAATTTTCAAGTTAGTCTT

CATGATCCGACCATACTCAACACATTAGTGCTAAACGTTCATTTACCAGAAGTCAAATTC

CAAAATGAACGGCATGGTTATCTACTCTTATACCGAATTTATTTTAAACAATCCAATTCA

GAATTTAATCCAAGATGTTTACTACAAGATGATAAAGGAGAAACAACAATACTAGCTATG

CATAGTAAAGATACTCCTTCATCTACATATGCACCTAAACAATTAAAATGGAACAAAATT

ACTATTCCAGATCAATGGAAAATAGATATTACACAACCTCCAAAAAATTTTGAACAAAGA

AATATCTCAAAAATATTTGAACAGAATGATGGAAAAATATTATTAAAATTTGAATCTTTT

AGAGAACCTATTCCACCGAGAATTAGTTCATATTATTCAAATAACTCAATATATAGAACT

CCTGCAAGAGCTTCCTTTTCCGAATAATATCCTGGTCAAACTAGTAAAAAAAAAGATTCG

GAAAATATAAATCAAAATGAACCAATTAAATTTAAAGCACCAATAGCAGAACCTGAACCT

AATATATATCCAACGTCCCCTACACAATCTGATTTTCAATCAATAAATGTTATTATAAAA

GATTTTGAAATAAACAAAGAATACATCAAAGAAGAATTTTACTCACTAAAAAATAAAGAA

AAAAGAGAATGGTACTTCAAAACTTTTAATAAACAACAAACAATAGATTATAGAACAAAA

TGGTATGATTTTATGCTAGAAAAAGAAATAGATATTCCATTTTTCATATGGTTTGAAGAC

TATTCAACAAATAATAAAATAAATTATCCATTTAAAGAAATTAATGTAAATTCTTCACTA

AGCCAAACATGGGAACAATTAGATGGTAAAAAAACTAATTCAATACATCCACCACTAACC

GATTTAAAAATACAAACAAGTAATAGACAAATTATAGCGGTTCCATTCAAGACAGGAGCT

AACGAAAATGATAATGATACTGTAACCAAAAAAGAAATAAAAAATATTTATCAACAAAAT

AATTTTCAAAGTCAAATACTACATACGATAGCTAAACAAATTGATAATATAGATAATAAA

TTGGATACAATTAACGAAGAAAATTCAATACCAAAACCGTTTTGTTCAGTATCAACACCT

ATGTTTAAACCATTTAAAAGAGAAATAAAATTTGCAGACAATGAAATATTAAAATCAATC

TCCACCAGATTAGACAATATAGATAAAGGGAAAAATGCTATTAACAATATAGACGAAGCT

GACGATAGTCAATCTTCGGAAATTGAAGAACAAGAAATAAACAGAATAAAAAACCGAAAT

ATCAAAAGTAATAAACCATATTACTCAAGGCACTCACCACCAGATATTCTATTTGAAGAA

CATTATAAATACAATGCAAGTATGTATACTGGAGATGGTCTATATGAATGGAATATAGAT

GGTAAATCAGAATACGAAATAATGAGCCTATTACAAAAAATGGGTATCGCTGCTCTAGCC

TACAAAGCTAGGGGAAAAACTGATAGACAAACTTGCATTATGCTACTAGCTGGTTTTACT

GACGCATTAAAATTTTGGTGGGATAACTCAATAGACTTAGACGTTCAAGAAAAAATTATT

AATCATATCGAAAAAATAAAAACTTATAACCCCGATAATTCCTGGGAAGAAATAGAAAAA

GAAAATGCCGTAGAAGTTTTAATCCATACAATAACTATGCATTTCATTGGTAATCCAGAA

GAAGAATTAGAAAGCAAAAAATTAATATTAACCAATTTAAGATGTCCAACATTAAGTGAT

TTTAAATGGTATAAAGACATTTTTATTACAAACATATTTCAAAGAAATGATTGTAATCAA

GCATTTTGGAAAGAAAGATTTGTCTCTGGACTACCATCTTTCTTCTCTGAAAGAGTATTT

AACAAATTAAGAGAATATTCAGGAGGACAACCAATCCCCTGGGATACAATAACTTACGGT

CAATTATTTGCATTTACTAAAAAACAAGGGTTAGCAATATGTCAAGAACAACGTGATAAA

AAACGCAATGATAAATTCAAATTTAAAATTTCAATGGGATCGTTTTGTGAACAATACGGC

TTCCCTCAACTTAATCCACCATCTAGAGAAAAAAGAATTAAGCATAGACAAAGAAATAAG

CAATTCCATAGAAATAGAAATTATAATAAACCAATGAATAAATTTTACTCTAAAAATAGA

AATTACACAAAAAACAAAATTTATTCAAAAAATAAAAAATACTCTAAAAATACTAAAAAT

GAAATAATATGCTGGAATTGCAAAAAACCTGGACATAGATCCCCGGATTGTAAAATGAAG

AAAAAAATAAATGAAATATTTCATGATCAACCCGATATCCAAGATAAACTAACCAAATTA

TTATTAACCTCCGAATCAGAAAAAACAGAAGAAACCAATAGCAATAATACAATAAATGAT

ATTGAAGATTTAAGTGATTCTTCTTCAGAATCACAAGAATCATCAAATCACACTTATAAC

GGAATATGCAATTGAAAATTAATTAATGTAATATCTGATAAAATTAAAAGACATAATAAC

TAGAGAAAATGGATTCGATACTCAAGAATCATTTAGTTTAAACAAAATATACGAAAAATA

TCCTACACTGAGTTTATTTAAACAGGTCACCACTGGTGAATTACAAAATGAAATTAAGCA

ATTAAAAACACAAATAAAAGAGTTAAAGATGACGGTAAATAAACAAGAATTAAATCAATT

AAAAATAGATGCAAAATTATCAATATTAGAAAATAATATTTCTATATATCAACCTTCATC

AGGCAATCTAATAATAAAAGAAAATCATGAAGATAATCAAGACTACATCCAGATAATTAA

TAAAATTAATTATCAAAAATGGTATACAAAAATAAAATTGGGAAAAATGTCGAAAAATGC

GCCAACTTTCAAATTTGGGACGATTAAAACATGAACTCTCGAAATGTCTCTTTAATCACA

AAGTTTCCGTTGACCTGTCTGAAAATAGCCAAGTTTTCGTTGACCTAGCCAAATTAGGCA

TCGCGTTAAGTCTCCGTTAGAACGATGCTGACGCTGTTAACAATCCGTTAGATTCTCCGT

TCGATCAAAACGACGTCGTTTTGAAATTAAAAAGGAAAAATGGCATCTCCAAAGAATCGA

ACTGATGACCTGTTACACTAATAAGAGACACTCAACCATTGGGCTACACGAACATTTTCA

TGTTAGTTCAAACAGAACATGTATATATACTAAAAACTGGTAACATAAATTAGGTAAAAA

TATATGCAATTAATTAAAAATAACAAAACTAAAAAATAATAATAACCAAAAATACACATA

TTAAATATACTTATCATAGTATATGTATCAAAATCTCAATATTATTTTATAAATAAAGAT

TTCTTCCATATTTGGAAAATTATTACTTCCGAAATTCCTTCTAATATAAAAAAAAAAAAT

ATATGACATAAAATTAATCATATAATTATGCGTTATATGCACCAATAGAATAAAATCTTT

ATATATATAATTAAATGTTTAAAATTTTGAAATATATATTTAAGGTAAGTATATTTAATA

TGTGTATATGTTTGGTTATAAATTTGTTTTTAATATTGTTATTTTAAATTAGTTGCATAT

ATTTTTACCTAATTTATGCTACCAATTTTTAGTATATATACACGTTCTGTTTGTACAAAC

ATTAAAATGTTCCCGTAGCCCAATGGTTGAGTGTCTCTTATAAGTGTAACAGATCAGCAG

TTCGATTCTCTAGAGATGCCATTTTTCCTTCTTATTTTCTGGTGAAACGACGTCGTTTGA

AAACTTCAAAACGACGTCGTTTTGATCGAACGGAGAATCTAACGGATTGTTAACAGCGTC

AGCGTCGTTCTAACGGAGACTTAACACGATGCCTAATTTGGCCAGGTCAACGAAAACTTA

GCTATTTTTAGACAGGTCAACAGAAACTTTGTGATTAAAGAGACATTTCGAGAGTTCATG

CTTTAATCGTCCCAAATTTGAAAGTTGGCGCATTTTTCGACATTTTTCCCATATAATTAC

ACATAGGATATGATTTCCAAATAGAAATAATAGCCTTAATGGATTCTGGTGCTGATTACA

ACTGTATCCGTGAAGGAATAATACCAACAAAATACTATGAAAAAACTACTGAAAAACTAA

GTGGAGCCAATGGCACTAACTTAAAAGTACAATACAAATTACCCTGTGCAAAAGTTTGTA

ATGAAGGATACTGTTTTACAAATCAATTTATATTAGTAAAGAATTTATCCCAAGAAATAA

TTTTAGGAACTCCCTTCTTTACACAAATTTATCCTTTTAAAGTATCCGAATTAGGGGTAA

CAACTAAAGTAGTAGGCACTAAGCTACTATTTAAATTTCTATCACCAATAAAATTAAAAG

AAGTCTTAACATTACAACAAAACACTATTCAAAAAACAATAAATCTCATTAAAAATAAAA

AAGAACATATTCAATCTCTACAAGAAGAAATAACTTACAAAAAAATAGAAGAACAACTTA

AAACACCTTATGTTTTAAGCAAAATAAAATCAATAGAAAACGATTTATTAAATCAAGTAT

GTTCAGATATTCCAAACGCTTTTTGGGAAAGAAAACAACATACTGTTGATCTTCCTTATA

TAAAAAATTTTAATGAGAAAAATATTCCAACTAAAGCAAGACTTATTCAAATGAATAAAG

AACAATTAGAACATTGTAAAAAAGAAATAGATGATTTACTAAAGAAAAAATTGATAAGAC

CGTCAAAATCTCCTTGGAGTTGTTCTGCCTTTTATGTTAATAATCAAGCTGAAAAAGAGC

GTGGCGTACCACGTCTAGTAATTAATTATAAACCATTAAACGATGTATTACAATGGATAA

GATATCCAATACCTAATAAGAGAGACTTATTAAAAAGGTTATACAGTGCAAAAATATATT

CAAAATTTGATATGAAAAGTGGTTTTTGGCAAATACAAATTGCCGAAAAAGATAAATATA

AAACAGCATTTATTGTACCATTTGGACATTATGAATGGAATGTAATGCCATTCGGACTAA

AAAATGCACCGTCCGAATTTCAACATATAATGAATGATATATTTAATAATTACTCAAAAT

TCACAATAGTATATATTGATGATGTATTAGTATACTCAACATCAATAGACCAACATATCA

GTCACCTAAATACATTTTTAAACGTAGTTAAAAAACATGGATTAGTACTATCCGCTAAAA

AAATGTCCCTATTTCAAACAAAAATAAGATTTTTAGGTCACAACATTTATCAAGGAACAA

TAACACCAATTTCAAGATCTATAGAATTCGCTGATAAATTTTCAAACGAATTAAGAGATA

AAACACAATTACAAAGATTCTTAGGATGTTTGAATTATGTTTCAGACTTCCTTCCGGATC

TCAGGAAAACTATTCAACCGCTATATCAACGATTACAAAAAAAAACCTAAACCTTGGACT

AATCAACACACTAATCTTGTTAAACAAATTAAAGAAAAAGTTAAAACACTACCATGTTTA

ACACTTCCTAACCCTGATGCATTTATGATAGTAGAAACAGATGCCTCAGAATTAGGATAC

GGAGGTATTCTTAAGCAGAAACTTCCAGATTTTAAACAAGAATCCATAATTAGATTTCAT

TCTGGAGTCTGGATTGGACCCCAGACAAATTATTCAACAGTTAAAAAGGAAGTTTTATCT

ATAGTAAATTGTATCTCAAAATTTCAAGATGACTTAATCAATAAGCAATTTCTTTTAAGA

GTTGATTGTAAATCAGCCAAAGAAATACTTCAAAAAGATGTTAAAAATCTTGTATCCAAA

CAAATGTTTGCTAGATGGCAAGCAATCTTATCTGCATTTGATTTTGATATTCAATATATT

AAAGGAGAAAATAATTCTTTACCAGACTTTCTAACTAGAGAATATTTACAGGGAAAAACA

AGTACTCAACAGATAGATAATCAACATGAGTGACAAGAAGAGACCGATGACCGCATTGGA

TTATATCAAGAATCAACCATCTATGCAAGGAAAGAATTTAAGTATTCAAAATACTTTCTC

GGCTCTTGCCGAATACCCACCTCTTTCTTATTCAAAGGCAGTATCTAATGATGCAAACCC

CCAGACATCCACCAAAGATAAACAACCAATCCAATCTTCTACATAAAAAGCTGCTTATTT

TCTCAAATCACAAAAACAACACCTTGTAACCACTTCTTATTCAAAACCCATTTCCCTAAA

AGAACTACAAAACTTTACCAGACGAATCTTCTATGAAGATTGTCAATATAAAACCGATAA

CATAACCAAAAACCGTACTTTTTATGAGTACATTCTAGTAGACTCTAAGTCTGCTGAGAT

TACTCATTATCCAAACAAATATGAACCTACTCAAATAGCCTACACTACTTCCAAAATACT

ACGTATCCAATCCCCACACGACTATGGATTCTTAAATCTCCATACCACAAAACCCTTTTC

ACAATCCGGTTACCATATTCAAGGATACACCTACACCGACTACCAAAATGCCTTCTTCAG

GACACTCTGCTTAAGGGCATTTGACCACTCCTGGTTCCTATCCTTCGACTCTAGATGTCC

TAACCAAGTCCCTGGCTGGTTCCACGAATGGTGGTACTGGTTTGGTCCAACCAATGAAAT

TTACC

>AalpV_sc1 [endogenous-virus-name=Arabis alpina virus] [host=Arabis alpina] [moltype=genomic DNA] [note=incomplete at 3’ end, mutations in ORFs 1 and 2] 7464 bp

TGGTATCAAAGCCGAAACGTTTTCCAAATAAGACCTAGAGTCCCCAATCTAAACTGAAAC

CTCTTCTACCCTGATACTCACCTATCTACTAAAACACTGTCACAGTAGGTGTTCCAGGCT

GCAAGTCCTCGAAGGCAGTAAGACCGTTGGCAAGCCTCTGTTGTGTCTCCCTAGATCTGG

TTAGTATCTCTCTAGTATGAGTTTAAGTCTAGATTGAGTCATGGCGTCTTCATTTAGAAA

GCTTTTTAAATCAAAACATACTGCATCTACATCACATACAACAATAAATTCAAGAGAGAT

TATGTAAAAAATAACATCTTTTAATCAAGAAATATCTAAAAATCAATTACCACATATTAA

TCCGGAAAATATTTATCAAATTGATACTTTTGATTTCAAAACAGCTTATGCAATAAAAGA

GCACGAACAAACTTTATCTTTACAGAATGAGTTCGAAGAAATAAAAAATTTAGCATCAAT

AGCTTTAGATAAATATAAACAAAAATGATTTAACTATTTACATTTTGGATTAGTCCAAAT

AGCAATAAAACCCCTATCAAGAACTGGTATTGATTCCCCAATATTAATGCTATTGAGAGA

CAAAACCTTACTAAAGTTTGAAGACTCACTCCTTGGAGCAGTCCAATCAAACTTATGTAA

TGGACCAATCTATTTTAATTGCGCTCCTAACTTTCAAGTAAGCTTGCATGATCCAACAAT

ACTCAATACTTTAGTATTAAATGTTCATTTACCAGAAATAAAATTCCAAAATGAACGACA

TGAGTATTTATTACTTTATCGTATCTATTTTAAACAATCAACATATGAGTTTAATCCACG

ATGCTTACTACAAAATGATAAAGGAATAACAACGATATTAGCAGTTCATAGTAAAGATAC

ACCAAACGCTACTTATATACCTAGACAATTAAAATGGAACGAAATAACAATACCAGACCA

ATGGAAAATTGAGATAACTCAACCAATAAGAAATTTTGAACAAAAAAATATTTCAAAAAT

AATCGAACAAAGGGATGGACGAATATTATTAAAATTTGGATCTATGAGAGAACCACTTCC

ACCCAGAATAAGTTCCTATCACTCAAGAGCTTCTTTTTCTGAATATCGTACTGGCCAAAC

CAGTACAGGCTCAGAAACTGAAAGCATAAATCAAAATACTATACAAGATTTAGAAACAGA

AAGCATAAATATTAAACATAATAATCCAATCCGATTTAAAGCCCCAATGGCTGAACCAGA

AATGGATATGTATCCAACCTCTCCTACAACCTCAGATTTCAAATCTATAAATGTTATAAT

AAAAAATTATGAAATCAACAAACAATACATAAAAGAGGATTTTTATTCAAAAGAAAATAC

AGTAAAAAGAAACTGGTATTTCAGAGTATTTACTACAGAAAAAACAATTGAAATTAGAAA

AATCTGGTACAATTACATGATCTTAAAAGAAACGGATATACCATTTTTCACTTGGTTTGA

AATATATTCAAATGAAAACAACATTACTTATCCTTTGGGATCAATTAATACTAACTCATC

TTTAATACAACAATGGGAGCAAGTAAACGGTGAAAAAAGTAAATCAGTACACCCACCGTT

AAAAGATCTAAAAATAAATACTACTAACAGACAAATTATAGCCACACCCTTTAAAACTGG

AGCCAATAGGATCGATAAAGATCCAGTAGTTGTACAAGAAATTAAAGCAATATATGAACA

AAATAATTATCAAAGTCAATTATTATACACTGTTTCAAGACAAGTAGATGATACAGATAA

CAAATTAAATAACAATGAAAATAATATGATAAATCAACAAAATCCTATTACTCAACATCC

ATTTTCATCAACGTCTACCCCAATCTTTAAACCTATAACCAGACAAGTCAAATTTCCAGA

AAGTGAAGTCTTAAAGGCAATCGCTAATAGATTAGATAAATTAGATAAAGCAAAAGGGAT

CAATATAATTGACGAAAATGATAACGAATCTATTCAAACATTAGAACAATCAGAAAATGA

TCAAGAGATAAATCGAATAAAAAATAACATGATGAGATTTAATAAACCTTATTACAAAAA

AATTCTCCGCCATATTTATTATATGAAGAACACCATAAATATTCCAATAAAGTATATACC

GGTGACGGCATATACGAATGGAACATTGATGGACGTTCCGAATATGAAATAATGAATTTA

TTTCAAGAAATAGGAATGACGGCTATAGCATATAAAGCTAGGGGTCATACTGATAAACAA

GCATGCACATTACTAGTAACAGGATTTAATGGAGCATTAAAATATTGGTGGGATAATTCA

ATATATTCAATCACACAAGATTCAATACTTAATCATATTGAAATAATAAGAGTAGAAAAT

GAAGATGGTCTCTATGAAGAAGAAGAGAAACAAAATGCAGTAGAAGTATTAATTCATACA

TTAACAATGCATTTTATTGGTAATCCAACTGAAGTATTACAAAGTAAAAAATTAATATTA

ACTAATTTAAGATGTCCTACATTAGGAGATTTTAAATGGTATAAAGGCATATTCGTAACA

AACATATTTCAACGAAGTGATTGTAACCAACCCTTTTGGAAAGAACGGTTTATCTCAGGA

TTACCAACATTCTTTGCAGAAAAAATAATCAACAAATTAAAAGACTATTCTGGAGGACAG

CCAATCCCTTGGAATACAATAACATATGGTCAATTATTTTCATTCATTAGAAAAGAAGGA

TTAACATTATGTCAAGAACATAAAGATAAAAAACGAAATGAAAAATTCAAATTCAAAATA

ACCATGGGATCATTATGTGAACAGTATGGTTATAATAATACTCTTAATCCACCTTCCAGA

AATAAACGATTAAAATATCGACAACGAAATAATAATAAACATTTTCAAAGATATAACAAT

AAAAATCAAAACAATCAATATTATTCAAAGAATAAGAAATACGCAAACAATAAACCCTAA

ACAAAGACGAATAATTATTCAAAAAATAATTTTAACAAACAAAAAGTAACATGTTAGAAC

TGCAAAAGACCAGGACATAAATCAACAGAATGCAAAATGAAAAAGAAAATAAATGAAATA

TTTTATGATCAACCTGATATCCAAGAAAAACTATCTAAATTATTACTAACTTCAGAATCT

GAACAATCTGAAGAAGAAAGTGACAGTGATTACTCAATAAATGAAATAGAGAATTCAAGT

GATTCTTCTACAGGATCACATGAATCATCAGATAATTTATGTAACTGTAAAATAATAAAT

GTAATAACCAAAGATAGTAATAAGCAATTCTTACTAGATATAATAGACAAAATTGACGAC

CCAAATACAAAAAAAGAATATTTGACAAAATTAAAAGAAATAATATACCAAGAACAGCAA

TTATCTACTCCACAACCTTTTAGTTTAAATAGAATTTTTGAAAAGTATCCATCTCAAAGT

TTATTTAAACAAGTAACTACAGGAGAATTACAAAATGAGGTTAAGCAATTAAAAACTCAA

ATTAAAGATTTACAATTAATAGTTAATCAACAAGAATTGAAACAATTACAAATAAATGCA

AGATTATCAGTAATTGAAGCACAAAATAATCAAGCATCAACTAGTAATCAAAAACTAAAA

GAAATAGTCATGACACAAGATAATGAAGAATATATACAAATAATAAATAAAATCAATTAT

CAAAAATGGTACACCAATATCACCTTACACATAGGACATGATTTTAAAATAAACATAATT

GCATTAATGGATATAGGAGATGACTACAATTGTATTCGCGAAGGAATAATTCCCACCAAA

TATTATGAGAAAACTATTGAACGCCTGAGTGGCGCAAATGGTAGCAATCTAAAGGTTCAA

TACAAATTACCCTGTGCAAAAATATGTAATCAAGGATACTGTTTCAAAAATCAATTTATC

TTAGTTAAAAACCTAACCCAGGAAGTTATTTTAGGTACTCCATTCTTCACTCAAATTTAC

CCGTTTAAAGTAACAGAAATAGGAGTAACTACAAAAGTAGTCGGAACAAAATTATTATTT

AACTTCCTTTCGCCAATGAAAACAAAAGAAGTATTATGTTTACAACAAAATACAATCCAA

AAAACAATAAACTTAATTAAAAGTAAACAAAATCATATTCAATATCTACAAGACGAAATA

TCATATAAGAAAATAGAAGAACAATTAAAAACACCTTATATAACAGAAAAAATAAAAGAA

ATAGAAAACGATTTACTAAATCAAGTATGTTCCGATCTACCAAATGCATATTGGGAAAGA

AAACAACATGTTGTCGAACTCCCTTATATAAAAGAATTTAATGAAAAAATAATACCAACA

AAAACACGACCTATTCAAATGAATAAAAAATTATTAGAAACATGTAAACAAGAAATAAAT

GATTTATTACAAAAGAAACTAATAAGACCTTCTAAATCTCCTTGGAGTTGTTCTGCTTTT

TATGTAAACAACCAAGCCGAAAAGGAACGAGGTGTTCCACGACTTGTAATAAATTACAAA

CCGCTTAATGAAGTATTACAATGGATACGATATCCCATACCTAATAAGCGAGATTTACTA

AAACGACTATATAATGCAAAAATCTATTCAAAATTCGATATGAAAAGTGGATTCTGGCAA

ATACAAATTGCTGAACAAGATAAGTATAAAACAGCTTTTACAGTACCATTCGGACATTAC

GAATGGAATGTAATGCCCTTTGGACTTAAAAATGCACCATCTGAATTTCAACATATAATG

AATGATATATTCAATAACTATTCAAATTTCACGATAGTTTATATTGATGATGTTTTAGTA

TATTCAAACTCAATAGACCAGCATATTGCTCATTTAAAAACTTTTATAAACATCGTTAAA

AAACAAGGCTTAGTAATATCTGCTAAAAAAATGCACTTATTTCAAACTAAAATAAGATTT

TTAGGCCATAACATATACCAAGGAACGATTACACCAATTGTAAGATCAATTGCATTTGCA

GATAAATTCCCAGATGAATTAAGAGAAAAAACGCAATTACAAAGATTTTTAGGATGTTTA

AATTATGTATCCGATTTTTTACCCAATCTGAGAAAAACAATTCAACCTTTATTTCAGCGA

TTACAAAAAATCCAAAACCCTGGTCTAGTATACACACAAATTTAGTCAAACAAATTAAGG

CGAAAGTCAAAACTCTCCCTTGTCTATCTTTACCAAATCCTGAAGCCCATATGATAGTCG

AAACCGATACTTCCGAAATAGGATTCGGAGGAATATTAAAACAGAAATTCCCTGAGTCAA

ACCAGGAGTCTATAGTTAGATTCCATTCAGGAGTTTGGATAGGACGCCAAAAATCTTATT

CTACCGTTAAAAAAAGGTCATTTCTATTGTCAACTGTATTTCAAAATTTCAAGATGATTT

AATAAACAAACATTTTTTGTTAAGAGTCGATTGCAAATCAGCAAAAAAAAATTTACAAAA

AGATGTCAAAAACTTAGTTTCTAAACAAATCTTTACCAGATGGCAAGCTATTTTATCTGT

TTTTGATTTCGATATACAATATATTAAAGGAGAACATAATTCTTTACCCGATTTCCTAAC

AAGAGAATATTTACAGGGCAAATCAACTAGTATTCAATCACAAAATACACAAGATGGCTG

ATCAAAAACAAAAGAAACCGATGACTGCATCAGATTATATCAAGAAACAACCATCTCTTC

AAGGAAAACCCTTACCTATCCAAAACATCTTTTCGGCTCTTGCTGAATTCCCACCTTTAT

TCTATTCAAAAGCTGTTACTAGTGAAGCTACAACCCAAGCTTCCACCGCTACTCAAAAAC

AAACTCCACCCACTTCAGAAAAATCCGCCTATTTTGTTAAACCACACAAACAGCACCTCC

TAACCACCCAATTCCTCAAACCTGTTACCCTTAGAGAACTTCAAGCTTTTACCAAACGTA

TCTTCTATGAAGATAGCCTATACCTAACAGATAATGTTACCAAAAACTGCACCTTTTATG

AGTATATTCTAGTAGACTCTAAATCTGCAGAAATTACTCATTATCCAGATAAGACCAACC

CAAATCAAATTGCCTATTCTACTTGCAAAATCCTTCGTGTTCACTGTATCCAAGATCTTG

GTTTCTTCAATCTCCATACTACCAAATCTTTTTCCTCTCCAGGCTTTCATATCCAAGGAT

ACTCCTACATGGATTATCAATACGCCTTTTTCCGGGCATTATACCTTCGGGCCTTTTTGA

CCATTCATGGTTTTTATCCTTCGATACTCGATGCCCAAAAGGAATACCTGGATGGTTCTG

TGAATGGTGGTATTGGTTCGGACCTACCGACCAAATCTATCCTTCAGAAATAATCAAAAC

AAGCCTCCCCTTTTATTCCAAACACGTAACAAAACAAGATGTTGGCCCTTTTACCACAAT

AGCCTTCCACATTGAGATGGGTCTCCCATGGATATGTTCTTGGCACTTTAACCTTGATAT

CGTCCTACCTGGTATGCCTTTCTCCCTAATCAAAGAATACCGTGTCAAATGGTGGGACAA

GTATAACCTTGATCGTTGTTCCATCTCCAACATAGCCAAACACTTTCAAATCCTTTCTCA

AACCCAAACCGCCATACAATCCGTTTCCAAATCCATACCTCTCCTTACTCCAAAACAACT

TATCCAAAAATTTGGCTCTGATCCGAAATCATCTCAGGCCACTCCCACTCCACACCAGCC

GCCTAGTCCGCTAACTCGACCGGCTCCTCCACCTCTAAATCACAAAAAAAAGCAAAACTT

CAACAACTTCTAGCCAACTTGGTCCACCAACTCGACTCCTCCGACGAGGACGATGACGTT

ATGCAAGACTCCGATCCTTTCGGTGGTCCCTTCGCACAAGACCCCTTCGCCGAATAATAA

TAAAAAAAAAAAAAAAAAAAAAACCCAATAGTATTGTATAATTTTCTGTTTTTCTGTTCA

TTCTAGTAGACTCTGTATATTATCTTCCTATATAAGACTCTGTATACTATCTTCCGATAA

GGTATGTCGAATAACATACAGAGTATATCAACAAAGAAGACAACAGCAACAACGACATTA

ATAACAAGCAATGACGTAAGCAATGACGAATGCCATGACGTATGCAATGACATAAGCAAT

GACGCACACAAAGTATCGGCACCACTACAAGACGACACGTGTCCTCCCAACTCACGCAAA

GTGTCGGCACCACTACGAGACGACACGTAGCAAGACCAGCAACTCCCTTCTTCCGCTATA

AATACTTACCTCTACCAACGAAGAAGGGCAAGCGATTCTCGCACTACTTTCTCACTCTAA

AAAATCCTCTCCAAAACATTCAAACGAATTTTTAAGTTTTAATATTTTCTCTTCGATCAA

ATCTTGTAATCAAATCTGTAAGTT

>BrapV [endogenous-virus-name=Brassica rapa virus] [host=Brassica rapa cv. Chiifu-401-42] [moltype=genomic DNA] [note=incomplete at 3’ end, mutations in ORF 1] 7881 bp

TGGTATCAGAGCCATGGTTGGGTAGGCGTCTAAAGTTTTGGGTGTTGGTGATCTATTAAT

TTTTCCTCCCTTTGGTTCTTATAGGTTAAAATCTTAGTCCTCCTGAGTGTTTTTGCCGGT

CTAGACAAGGTCCTAAGTCCCGTAGGTGTTGGGAAAACAAAACCTCTCTTTAGACAGGAT

TGTAAGGAGTCTTAGATTTTCACCTTTATCATCAAAAATGGAATCATATTTTAGATCTTC

ATCATACAAATCCCAATCCGCCTCTTCTTCTTCCAGATCGGAAAAGAAAGCTATAAAAGA

TTCAAAAAAGATAGTAACTAGTCAAGAAACAACTATAGATGATTTCCAAGGATCAATTGA

TAATTGGAAGATTCCAAGAGTTCATAAAGATCAAATTTACCAAATCTCGAAAATGAATTT

TCTAAGAACAAATTTTTCTATTAAAACTGAGGAACGTGATATTCAACTTACAAAACCTTT

TGAGACAATTCATTTATTTTCTGAAAATGCTTTACAAAGGCATAGAGAAAAAAACTTTAA

ATATATTCATATTGGCTGGATTCAAGTCGGCATAAAACCTCTTTCAAAAGAAGGGTTAAA

TACTTCAATATTAGCTATTTTAAGAGACACACGATTTAAAGTATTTGAAGATTCTTTGTT

AAGCTCGGTTGAATCTAGTTTATGTACTGGACCAATTTCATTTGATTGCTATCCAAACTT

TACTGTTTCTCTAAATGACAAAAATATCTTAAAATCTTTGGTGTTACAAGTTAAAACCCA

CAACTATGAAATGATTGAAGGATCAATTCCAATTGCTTTGATCTTTAGAGTTCATTACAA

AGCTATGACAAGTGCATTTTCTTCAAAGGTAAAACTTTCCAGCAAAAAAGGGGAGACCCT

GTTGCTTCAAACTGATCTTTCAAGATCCAACTCCGTTATTCCGAGATCCATCCAATGGAA

AGATATAAATCTTCCAGACGAATGGGTCCTCGAAGGAGCGGTCCAACCAAAAATTCCTGC

ACCCCAAGAATCTATAGAACCAAATACACGTTTAAAACATATTGAGCAATTTCGTGATGG

AAAAGTAAAACTGTCTTTTATCAGAAATAACCAAGATAGGATTGATGAATATTTGTGTGG

ATCTTCTTCTAACATGTGTGAATCTTCTTCTAATCCAGAAACTATTGATTTAGGAAGAGT

CTCACAATTAGGTCATGATTTTCCTAGAGAAGAAAAGAGTATGAAATTCCGTCCCCGGTT

TTCAACCTCGGATATTCCAAATTCTGTTTTAAGAAATGTTAATTTTCAGTCACAGATTCC

AAAACCGGTTTACTCTGCGGAAAACGATCTTTCACAAAATGAATTTATTACAAAATCTGA

GCCAACAAGTCCAACTTTTTCAGCCATTACAGATAACATGTTAAATGAATTAAATGTTTT

AGAAAAAGAATTTTCAATTGACAAAGAGTTTTTAAGAGATGATTTTTACTCTAAAAATAA

TAGTCAAAAAAGGCTTTGGTTCTTCAAGAACTTTTTAAAAGAAAGAAATAACATTCAGAA

TGAATTTTATGATTATCTTCAAACACATAAGGTACAAATTTTATTTTTTAATTGGTTTGA

AGATTATGCTTCTAAGAACAGTATAAATTTTCCTTTTTCTAAAACAGTGAATCCAATTAC

CAATCGCAATAAAACTCCTGAATGGGAAACACTTGCTAATGGTAAAATTATTCGCTCAAA

TCATCCTCCTCCCCAAGGGATAAAGCTAGATTGGTTAGGAACCCCAATTGAAGCAATTCC

TTTTAAACGACCCCAAGAAGATGATAAAGCTAATATAAAAAATATCATTGTTCAAAACAA

TTTTTCAAACACTAATCTTGGTACAATAAGCAAACAACTTGATAGGATTGAAAAATCAAT

CCAAAATCAAAACCCCATTCCAATGGAGTCTCCTTCTGAAAAGAAAAACAAAAGTCCCAT

GTTTAAACCTTTCCAAATTTCCAATTCAAGTGTCAAAACTTACCAAGACACAAACCTTGA

ATTCATTCGTGCCCTCCAAAGTCAATTAAGTAAAGCTGAAATAGGAGATAGTAGCATGCC

ACATATTTCTGATATCCCCGATACTCCTACATCCAAAATCCAAATAAACACTCTAGCCAA

TGATTCAGACATTAGTGAATGTTCTGATAAAAATTCTGTCTCAAAAATAAACCGTCTTGA

TAGGCAAAACCATAAAGATCGTCAATTAATCTCAGCCCCTGATCTAGGACAAATCCAACC

AATAGAGCAATCTCGATTTAACTCATCATCAGTCTATGACTGGAATATTGATGGTATATC

TGAATATAACATTTTAAGTTTCCTCCAAAAAATGACAATGGCCGCAAACGCTTATAGAAC

CCAAATAGGAAATGAGGATAAAACCGTAGCTGAACTCCTAATAGCTGGCTTTTCGGGACA

GCTAAAAGGATGGTGGGATAATTACCTAACCAATCAGCAACGAACAGAAATCCTAGATTC

AATTAAAACGGATGAAGATAATGTACCAATCCTAGATAATCTCGGAAATCCTCAACAAGA

TGCTGTTGCTACTTTAGTCATAGCCATAACCTTGCATTTTATAGGAGACCCCTCGGTCCT

TAGAGATAAAAATGCTGAACTCCTTTCAAACCTTAAATGCAAAAAACTAAGTGATTTCCA

ATGGTACAAAAACACATTTCTTACTAGAGTCTTACTCCGTCAGGATTCTAACCAACCTTT

TTGGAAAGAAAAATTCCTAGCTGGCCTTCCTACTCTCTTAGGAGACAAAGTTAGAAACAA

AATTAGAGATTCCATGGGAACCCAAATAATAGATTATGATGATTTCACTTATGGAGAGTT

GGTCAGTATTGTTCAACAAGAAGGTCTTAGGATATGCCAAGACCTGAAACTCCAGAAACA

TCTTAAATGGGAACTCAAAAGAACGAAAGTAGAATTAGGATCATTCTGTAAACAATTTGA

AATAGACCCCAATCAAGGTAGTAAATCTTGTGTAGGAGATTGCAACAAAAACTACTACTC

TAAAAATAAGCCTAGAAAACATTCTAGAAACTATAAAAGTTTTAGAAGTCGTGAAAATTC

ATGGAAAAAACCCCAAAACAAATTCCCTTCAAAGGAAACCCCAGCCAAAAAACCTTTCTA

TAAAGATCTTACATGCTTCAAATGCAATAAGAAAGGTCACACTTCAAAGTTTTGCCGTTT

TAATCGCAAAATCCAGGAACTAAACCTCGGAGAAGAAATTTCAAATAAAATAGATAATCT

TTTACTAAATGATTCCTCTTCGTCATATGACTCTGACTCATCCGTGGCTTCTGAAAAGGC

CCTTCAGGTTGATGAACTAAATAGTTCATCATCTAGTTCAGAGTCAAATGAAAAGAATAT

CTATGTCCTCACTAAAGATCAAGAATTACAACTTGAAATATTAAATTCCATTTCTGATCC

AAGCCTAAAACAGAAATTTCTTGAGAAAATCATTTCCTCTTTAAACGAAAAAGATGATAA

AAACATAGATAATTCCCCAAAATCTCATGGTCCAAGTTCCTCCAAATCCTCTTATGATCT

CACCACTATTCTCAACAAAAGAAAGAAAGACACCTCGAAAACAACAATACAAGATCTTAG

GATTGAAATCAAAGAAGTAAAAAATGATCTAAAAATCCTCAAAGAAAAACAAAGACAAGA

TTCTGAATATTTTCACTCGATCATATCTTCCATCAAAGATCCTCATGATTCTAGTTCGGA

AGAACAAGGAAATGATTGCCTAAATAATCAAGATGAAAATCTCAAAGAACAAATTCAATC

TCTAGATATGGCCCCTAATGATTTCCTTTTCGTTCTACGAGAAATAACTTCTCGAAAATA

TAAAATTAGAGTCACCATTGTTTTTTCTGAAAATTACAAAATTGACACAATTGCTTTGTT

CGACACTGGTGCAGATCTTAACTGCATCAAGTCTGGGCTTGTTCCAAAATGTTTTCATCT

AGAAACTAAAGAAAAACTATCTGCTGCAAATAACTCTAAACTTCGTATAACATCTAAAGC

AGAAGCTTCAATATTAAAAGAAAATATTCTTATTAAAACTGCATTCGTTCTCACAGATGA

TATATATCAAAATGTTATTTTAGGAACACCATTTATTAATTTGATCACACCATATAAAGT

TATGGATGATAAAATTTCCTTCAAAAATCAAAATATAAAATTATCTTTTAAATTTCTCGA

AACACCAAAAAAGCGAAGTCTAAACTTAATAAAAGCTTGTTCTATACAAGAAAACTTTCT

AAACTCTTTAATCCATAGTAAAACCTTCCACCTAGAACATTTAAAATCAGAAGTTTTACT

TCAAAAAACCTTAGAAACCTTAAATTCAAAAGATATAAAAGAAAAAATTTCAAACTTAAA

TAAATTATTTGAGAAAGAAATTTGTTCAGATTTACCAACTGCTTTTTGGAATCGAAAACA

ACATATTGTTGATTTACCTTATGATGAAAATTTTCTTGAAAAAGAGATTCCCACAAAAGC

TCGTCCAATTCAAATGAACTCAGAATTAGAAAAACATTGTAGAAAAAAAATTTCGGATCT

TCTAGAAAAATGATTAATTTCAAAATCAAGATCCCCTAGGTCTCGTGCTGATTTTTATAT

AAATAAAAATTCTGAAATTGAAAGAAGAACCCCAAGATTAGTAACTAATTACAAACCTTT

AAACACTGCTTTAAAATGGATTGGATATCCAATACCAAATAAAAATATTTACTACAAAGA

CTTTACTCTGCCCACATATTCTCAAAGTTTGATCTAAAATCAGGATTTTGGCAAATTCAG

ATATCTGAAAATGATCGTTATAAAACAGCATTTACAGTCCCTTTTGGTCAGTACCAATGG

AATGTAATGCCATTTGGTTTAAAAAATGCACCTTCTGAGTTTCAAAGAATCATGAATGAT

ATATTTTCTGATTATTCAAAATTTTGCATTGTCTACATTGATGATATACTAATATTTTCA

CATTCTATTGATGAACATTTCAAAAATCTCAAAACATTCTATTACGTTGCTAGAAAAAAT

GGTCTTGTTGTTTCCAAAACAAAAATGTCCCTATTTCAAACAAAGATCAGATTTTTAGGT

CATTACATATCACAAGGTATGATAACCCCTATTGAAAGATCTTTAGAATTTGCAAGTAAA

TTCCCAGATAAAATTATTGATAAAACTCAATTACAAAGGTTCCTTGGAAGTCTCAATTAT

GTTTTAGATTTCTGTCCTAATATAAATAGAATAGCTAAACCGTTGCACAATCGTCTAAAG

AAAAATCCAGTTCCTTGGACAAAAGAACATACTGAAATAGTTCAAAGGATTAAAAAACAG

GTCTTAGAAATCCCATGTCTGCACATAGCAGATCCGGATTTAGAGAAAATCGTAGAAACA

GATGCTTCTGATATAGGATATGGGGGTATTCTTAAACAAAAGAAGTTAGATGGTCACGAA

AGTATTGTGCAATTTACATCATTACACTGGAACGACACTCAAAAGAACTATTCTACTATC

AAAAAAGAAATCTTGAGTATAGTTTTGTGTATTCAAAAATTTCAAAGCGATCTTTTAAAT

AAAAAATTTCTTTTAAGAATTGATTGCAAAAGTGCTAAAGATGTTTTACAAAAGGATGTG

AAAAACCTTGCATCAAAACAAATTTTTGCGCGATGGCAAGCGATTTTGAGTATTTTTTAT

TTTGAAATTGAATTTATAAAAGGAGATTCAAACTTTGTCCCAGATTTCTTGACAAGAGAA

TTTCTCCAAAACAGGTACAATGCCTCCGAAGAAGATGATTGACAAAGGCAAGGGAATTTC

CCTGGAACCAATCTCAGAAAAACCCGAACCTACAAACACTAACCCAAAAACAGAACCCTG

GATCTCAGTAACAAAAAAGAGTGCTAAAAAGGAAGAAATACCAACCACCCCAAAATCTCT

CCAATCTCCAGAAGCTATCATGAATCAAATGTTACAATTGCAAAAAGCTTTCGAACTTAG

TTTTTCAAAGCAAGAGTCGGGAGAATCTTCCAAAAACCTTCCTTCTCAAGCTATTTCAAA

AAATAACGAGGTCCCTTCAAAAATGATTTCAAGAGAAAACCAGACCTCAAAAATTGTTTC

AAAACCTTCAAATAATTCACAAATTATTTTGAAGGAGATTTCCCAAACCACTCCATCGGA

GTATTTTGAAAAACACACATACCAAAATATTATAAATATCGAAAAGGGATTTTTTGTGAA

CAATGATCCGTTTTTAACAATCGAAAAATATTTTGGCAAAAACAATTTCTTTAAACCACA

TAATACAGATAAAACTGTGTTATTCTTTCAAACCATTTTGGAAATTACAGATTCAGTGTC

CTTCAAACATTTTTATCTAAACGAAAACCACACTGATGCTGCTTATTCCACATTCAAAAT

CCATAAAATCATTGCGCCATGTGATTGGGAATATGATCTTAACGACAATCTAAATTTTCC

AGAAGATCTTAAAAATCTCCCTTGTTACAATGTCCCCTTTAACTATTGGGATTATTGCCA

AGCATGGTACAATTCTTTTTTGATCCAATCTCCTAAACACAAACACACGTGGTTGATCTT

CTTTGACACCACCTGTAACTTATCCAAATCTCCCTATTGGTTCATACCATGGTGGAACTA

CTTTGGTTCTGTCACAGAAATATTTAAACCCAATATCCAAAAATCATTCCAAATCTTCAA

GACAAATTTCATTCCGTCTGAAGAAGAAAAGAAATTTCCCCCACTAGCCCTCTTCCACTC

AAAATTTCATTTTCCTTGGGTTTTTTCATGGACCCTAGAATTTACTTCTGATCCAATTCC

TGTTATCCGAAGAAAATTCAGAGTCAAGTGGTGGGATAAATACAAAATTCCCTCAAACCT

TTACCCGTCAAATATTCTTACCTGGATAAAAGAACAAAACTCAAAAATCCAAACACTTAA

CCAAACACAGAACCAAACACCAGAGACAACCTTTTTCTTGGCTCAAAAATCCAAAATGTT

AGCAATGTTGGCAGGTGCAAAATCTGAAGAAGAAATTAAGGCTATCTCCTTACAATTTCT

ACCGTCAATGTCAGATCAAATCTCTCGTACTTCAGGGAGTGATTCAGGGTCCCAAGTGCC

AGATATCGATGAAGAAATTTTTGGCAACAGTGGAGTTTAAATCTCTCTCCCCAAGAAATG

CAAATCTCCTCAAAATCCAAAACGTATCTTTGTTGCAACTCTGTCAGATACGGTTACATT

GTTACGAATATTTTCCACGATCTCTTGTCGCAATACCCGATAAAAATCCAAAACATCAAA

TAAGTGCTATCTCCTGTAGCCATGAAGACCAAGGTCCACTTCCAGCAGACAGCAAACAAT

TATGGAGCTTAATGGAGCAAGCTTTTGCTAGTGGAGTTATCCATATATGCCACTTTTATT

TTTATTTTTATTTTTATTTTCATAATTTCACCCTTGTAAACTCCTATATAAAGGAGTCTT

TGCTTCAGTTGTAAACCAAGTTCTCATTCTCTCATTCTCCTACTCTTTCATTCATATCCT

CTCTTACATTCTCTCCAGTCCTTGTAATCGAGAACATTGCTACAATCTCTTTTTAAGTAA

GTTTTTAATAAATAAAGTTTCAATTATATTTTTCTTGTTTCATTTTCTCGTTTTAGTATT

ATGCTTTATGTTTAAGTGGCTGGTCTAACCGCCCATATTTCTGTTTTATTTTTAAGTTAT

ATTTCAATCTGTTTCATATATGTCTTAATTTACTTTTTATTATAAATGTTTAATTATGCT

TTCTCCAAGACCGTTGTCTGA

>CrubV [endogenous-virus-name=Capsella rubella virus] [strain=sequence cluster 1] [host= Capsella bursa-pastoris] [moltype=genomic DNA] [note=incomplete at 5’ and 3’ ends, mutations in ORFs 1 and 2] 6232 bp

CATAGGTGTTCCACGCTGTAAGTCCCGAAGGCAGTGAGTCCGTTGGTAAGCCCGTGCATC

ACCCGTCGTCGATAAGATCGGTTAGTTTGCATTTTAGCATGAGTTTAAGAATATTTTAAA

CATGGCATCTACGGTTAGAAAGTTGTTTAAGTCTAAACATGCATCATCCTCTACATCTCA

AAAAACAATTAATTCAAGAGAAATCATGTCAAAAATTACAACTTTTAATCAAGAAATATC

AAATGACCAACTACCACATATTAGTCCAGAAAATATATATCAAATAGGAACTTTTGATTT

TAAAACAGCGTATTCAATAAAAGAACATGAACAAACTTTATCTTTACAAAATGAATTTGA

AGAAATTAAGTTACTTTCTTCTATGGCTTTAGAAAACTATAAAAGAAAGAAATTTAATTA

TATTCATTTCGGTCTAATACAAATAGCAATAAAACCTTTACCCAGAACAGGGATAGATTC

TCCAATACTTATGTTATTAAGAGATAAAACTCTTTTAAAGTTTAAGGACTCCTTGTTAGG

AGCTGTACAATCAAACTTATGTAACGGAGCTATATACTTTAATTGTGCTCCAAACTTTCA

AGTAAGTTTACATGACCCAACAATATTCAATACTTTAGTTTTAAACGTACACTTGCCTGA

AATTAAATTTCAGCAAGAAAGACACGGTTATTTATTGTTGTATCGAATTTAGTTTAAGCA

ATCTACATCTGAGTTTAATCCTCGATGTTTGTTGCAAGATGATAAAGGCGAAACAACAAT

ATTAGCTGTACATAGTAAAGATACTCCAACCTCTACATATACACCCAAACAATTAAAATG

GACAGAAATAACAATCCCTGATCAATGGAAAATTAATATTACTCAACCACCACGAAACTT

TGAACAAAGAACTCTTACAAGTATAACAGAACAATACGATGGAAAAGTATTATTAAAATT

TGGTTCTTTCAGAGATTCACCATCATTAAAAATAAGCTCTTTTTACCCACGTTCATCTTT

CTCTGACTATAGAACTCGCCAAGATAGTACTGAATCCGAAGAAAGTATAGAACAAATTTT

AACAAAAAACCAAGAGAAAAAACCTATTAACTTTCAAGCACCAATAGCTGAACCAATTCA

ACCATCAGAGGCAGGATCGTCTAACTTTCCTACTTCTCCAACTCCTTCAGATTTTAAATC

AATAAATGTTATTTCGAAAGTATTTACGATAGATAAGCACTATCTTAAAGAAGAATTTTT

ATCACATCAAAATAGAGATAAAAGAAAATGGTACTTTGAAACTTTTACTAAAGAAAAACT

TACAAGTTTTAGAGAAATATGGTATAGTTACATGGAAATTATAGAAATAAATATTCCATT

TTTTACATGGTTTGAAACCTACGCTTTAGAAAACAAAATAGACTACCCATATAAAGATAA

ATCAATAAACACTAGTTCAACCTTATCTCATACGTGGTAAATGACTAATGGGGAAACACA

TAAATCAGTCCATCCACCGTTAAAGGATATAAAAATTCACACTCCTCAAAGACAAGTAAT

AGCTACACCTTTTAGAACCGGAGCAAATAGAGATGATAGAGATTCAATAAATATGGCTGA

CATTAAAATAGTATATGAACAAAATAATTACCAAAGTCAAATACTACATACTATCTCACG

GCAAATTGATCATATAGATAACAAATTAGATAAAGACGAAAATGTTTTATTAACAAACTA

TCAACCATTGTTACCACCTTTTAACACAATATCACCACCCCTCTTTAAACCATTAACTAA

ACCCATCAAATTCCCAGAAAATGAAATTTTAAAATCAATTTCAAGTAGACTAGATAGACT

AGACAAAGGTAAGGGTATAAACAATATTGATGAAAACATTCAGTCTGAAACAGATATAGA

AATAAATCGAATAAAAAGAAACTTCCAGCAATCCAAAAAACCATATTATCCAAGACACTC

TCCACCCGACCTATTATTTGAAGAGCATAATAAATTTCCCTCCACAAACTTTTATAACGG

GGAAGGGATATATGAATGGAACATAGATGGAAGGTCCGAATATGAAATGATGAACTTATT

TCAAGAAATGGGAATGGCTACGTTAGCTTATAAATCTAAAGGAATGTCAGATAAACAAGC

ATGTCTACTCTTAATCTCAGGGTTTAATGGTGCCTTAAAATTTTGGTGGGATAATGCCTT

AGATATAACATCACAAGATTCTATTATAAACCACACTGAAACTAAAAGAGTAGAAGGGGA

TGATGGTTTTTATGAAGAAGAACAAGTTCAAAACGCTGTAGAAGTATTACTTCATACCAT

CACCATGCATTTCGTAGGAAATCCTTCTGAAGAACTTACAAGTAAAAAACTTATTTTAAC

AAACCTTCGATGTCCAACACTAGGAGACTTTAAATGGTATAAAGACATATTTATAACAAA

CATATTTCAAAGAAACGACTGCAACCAACCCTTCTGGAAAGAAAGGTTCATTGCAGGATT

ACCTTCGTTCTTCGCAGAACGATTAATTAGTAAACTAAAAGATTTTTCTGGCGGACAACC

GGTACCTTGGGATACGATAACCTATGGTCAGTTGTTTGCTTTTGTTAAAAAAGAAGGATT

ACAAATATGTCAAGAACATAAAGATAAACAAAAAACTAATAAATTTAAATTCGGTGAAAC

TATGGGCTCATTTTGCGAGCAATATGGTTATCATCGTCTCACTCCACCATCTAGAGAAAG

ACAAAAAACACGTAAACAAAATAAATCATTTTACCCACAACCTTACAATAAAAGGAAAAG

ATTTTCAAAAAGATTTTCTAACTCGTACTATACAAAAAATAAAAATCCAAAACAACCAAA

TAAAAAAGAAAAAATTATATGTTGGAACTGTAAACGACCAGGACATAAATCTACTGATTG

CAAAATGAAAAGAAAAATTACTGAAATATTCCACGATCAACAAGACATTAAAGAAAAATT

AGAAAAGTTACTTCTTTCGGAAACAGAAAATTCAGAAGAACTTAGTAGCGATCCTTCTCT

AGACATAATAGAAAGTGAAAGCGATACATCAACTTAAAACTCTGGGAATTACTCAGATGA

TATATGCAACTGTAAAACAATAAATGTAATAACTAAAGACATCGATAAACAATTTTTAAT

AGATATTATAGACAAAATAGATGATCAAGAGACTAAAAAGGAGTATTTATTAAAATTAAA

AGATTTAGTGCAAAGGGAAAATCAGTTGCTAACCCCACAACCATTTAGTCTTAATAGAAT

AATAGAAAAGTATCCTTCCCCAACACTATTTAAACAAGTAACTACCGGTGAATTACAAGG

AGAAGTAAAAAATTTAAAAGCACAAATAAAAGAATTATAACAAATAGTTCACCAACATCA

ACTTCACCAACTACAAGTAGACGCTAGACTAGCTCTTATAGAACCCAAAATCCGCCAACC

TCAACCCATAACAACGGATGAACCCTCCACAAGCAACCCTGATAAACATGAGAAAACAGA

AGAAGAAGAATATATCCAAATAATAAATAAAATAAACTATCAAAAATGGTATACGAAAAT

AACATTACATTGGGTATGATTTTAAACTAGAAACCATAGCACTTTTAGATACAGGAGCTG

ATTATAACTGTATTAGAGAAGGAATAATTCCTACAAAATTCTACAAGAAAACATACGAAA

AACTTAGTGGTGCAAACGGAAGCAGTTTAAAGGTCAATTATAAATTACCTAAAGCAAAAA

TTTGTAATAAAAACTATTGCTTTAAAACACAATTTATCCTTGTAAAAAATTTAAGTCAAG

AAGTTATACTAGGAACTCCATTTTTTACACAAATTTATCCATTTAAAGTTACAGAGCGAG

GAATTACTACAAAAATTGTAGGAGAAAAGATAATATTTGAATTTCTATCACCTATGAAAA

CAAAAGAAATTTTAGCTTTACAGAATTCCACAATAGAAAAATCTATAAACTTAATAGATA

GAAAACAGAAACATATTCTATACTTACAACAAGAAATAAAATACCAACAAATAGAACAAC

AATTACATGATCCACAAATAATAAATAAAATAAAAAAATTAGAAACTGAACTTTTAGCCC

AAGTATGCTCTGAACTTCCAAATGCATTCTGGGAAAGGAAAAAACATATTGTTGAAATAC

CCTACGAAAAAGACTTCAACGAAAAGCATATTCCAACCAAAGCTAGGCCCATTCAAATGA

ACCAAGAACTATTAGAACATTGCAAAAAAGAAATACAAGAATTGCTTGATAAAAATTTAA

TAAGAAAATCTAAATCTCCTTGGAGTTGTTCAGCATTCTATGTGAACAACCAAGCTGAAA

AAGAAAGAGGAGTACCTCGCTTAGTAATCAATTATAAACCCTTAAACAATGTAATACAAT

GGATAAGATACCCAATACCCAATAAAAGAGATCTACTAAAAAGATTATATGAGGCCTGTA

TATTTTCAAAATTCGATATGAAAAGTGGATTTTGGCAAATACAAATTGCCGAAAAGGATA

AATACAAGACAGCATTCACAGTGCCTTTTGGGCATTACGAATGGAACGTCATGCCTTTTG

GCTTAAAAAATGCACCTTCAGAATTCCAAAACATTATGAATGAAATTTTTAACCCTTACA

CAACTTTTTCTATCGTATATATAGATGATGTTTTAATATTTTCCAAATCTATTGACCAAC

ACTTTAAACACTTATACACCTTTTTAAACATTGTTAAAAAGCATGGACTAGTAGTATCAG

CCAAAAAAATGCAAATATTTCAAACAAAAATACGATTTTTAGGCCATAATATTTATCAAG

GAACTATAACACCAATATCTAGATCAATAGAATTTGTAAGCAAATTTCCAGATGAAATCA

AAGAGAAAACCCAATTACAAAGATTTTTAGGGTGTTTAAACTATATATCAGATTTTCTAC

CAAACCTTAGAAAAACAATTCAACCACTATTTCAAAGATTGCAAAAAAATCCCATACCAT

GGTCTAGTCTGCATACTAGTCTAGTTCGTGACATTAAGAAGAAAGTTACAACACTACCAT

GTCTAGTCATCCCACACCCTAATGCATTCATGATAGTTGAGACAGATGCATCAGAAATAG

GTTACGGGGGGATCCTTAAACAAAACTGCCAGAGTCTACCCAAGAGTCTATAGTCCGTTT

TCATTCAGGGGTTTGGCTTGGACCCCAAAAGAATTATTCTACTGTTAAAAAAGAAGTTTC

ATCTATAGTAAACTGTATTTCTAAATTTCAAGATGATTTAATAAACAAAAAGTTCTTACT

ACGTGTCGACTGTAAGTCAGCTAAAGAGATTTTACAAAAAGATGTTAAAAATTTAGTTTC

TAAACAAATATTTGCTAGATGGCAAGCTATTTTATCCGTATTCGACTTCGATATACAATA

TATAAAAGGAGAAAATAATTCATTACCTGATTTTCTAACAAGAGAGTGTTTACAGGGAAA

ATCTCAGGACTATCAAAATGAGTGAAACACAAAAGAAGCCAATGTGTGCATCAGACTATG

TCAACAATCAACCGCACCTACAAGGAAAGCAAATAACTACTGGTAACCAATTCTCTGCTT

TAGCAGAATTCCCTCCTTTATCTTATGCAAAAGCTGTCAACCCCCAACCACAAAAAATAT

CAACACCATCCTCCTCTCAAAAAACCTCAGACCAAAAACCATCATACTTCCTAAAACCAC

AACGCCAACATCTTATTACAACCTCATTTACTAAACCAATATCTCTCAAAGACCTTACCC

AATATACCAACTGTATTTTTTACGAGGATAGTCAATACCTTACCGACAACCTTACCAAAA

ACCATACATTCTACGAATTCATTCTCGTAGATACAAAGTCAGTTGAAATAACCCACCATA

CCGACAAAAATAACCCAAACCTCATCTCCTACTCCACTTGCAAAATACTCAAAATATGCA

ACCCCCAAGATCTCGGCTTTATTAACCTCCACACATCAAAAGATTTCTCTATACCAGGTT

ACCATATACCAAGATTTACCTACATAGATTACCAAAAAGCCTTTCTCCATACCTTTTACC

TACGAACCTATGATCACTCCTGGTTCCTCTCCTTTGACTTCCATTGCCCCAAGACAATAC

CAGGATGGTTCTATGAATGGTGATACTGGTTCGGCCCCTCCGATGACATATA

>CclemV_scCc1 [endogenous-virus-name=Citrus clementina virus] [strain=sequence cluster Cc1] [host=Citrus clementina] [moltype=genomic DNA] [note=complete genome, mutations in ORF2] 7597 bp

TGGTATCAGAGCCAAGGGGAGGAAAGTGTTTTATTGTTTAGAGTTTCGTGTTTAAAGATC

TTGTGTTTTATTTTGAAGTTTTGTTTTGTGGTGAATTTTTACTGGTTGTAAGTCCCGTTT

TAGGATAAGGATTCCGCTGAGGGCTGTAAATCCATACCTGTAGCCATATTGACCAAACCA

AAAATAGACCATAAGGTCTTGACCATGGATCCTCCAATCTGTAGAACATCGTCCTTTTCT

AGCTCTTCTTCCGGGAAGACTAGTGAGTCAAAGCATGTTGTGAGTTCAGAAGAATTCATT

ATTGAGAACTTTGATAAAGCAATTGATTGTTGGGAACTTCCAAAAATTTCCAAAGAAAAG

ATTTACAAAACAAAAATGCTTGATCTTTTAAAGCATGATTATATTATAAAAACTGAAGAA

CGTGATATAACTCTTTCAGAGCCTTTTGAAACAATTTATTTGTTTTCAGAAAAATCTTTA

AAGAAATTAAAAGAAAAGAATTTTAAATATATTCACATTGGTTTAATCCAAGTAGGAATA

AAACCTTTGACCAAAGAAGGCCTGGATACTTCTATCCTTGCTGTCCTTAGAGATGGGCGA

TTCATCTCTTTTGATGATTCTTTACTAAGTAGTATCGAATCTAGTCTATGTAAAGGTCCC

ATATCTTTTGATTGTTATCCAAATATAACAATTTCTCTTAAAGACAAAAATATTTTAAAA

AGCATGATTTTACAAATCAAAACCCATAATTATCATATGATTAAGGGATCCATCCCAGTT

GCATTAATTTTTAAAATTTCTTACAAAGCCATGATTTCTGCATTTAGCACACAGCATAAA

TACCAGTCAAAAAGAGATGAGACCCTTCTCTTGCAGACTGATTTGTCCAAAGCAAACACA

GTCATTCCTAAACCAATCCAATGGAAAGATGTCAACCTTCCAGAGGAATGGATCCTTGAA

GGAGCCGCTCCACCAGCGATTCCGAAACAACTTGAGCCCAATACAGAGTTGCAAAATGTG

ACTCAGTATTCCGATGGTAAAGTCAAACTATCATTCAGGAGATCTAACTCATCCAGATTT

TCTGATAAAGCGTCGTGCTCAAGCATTCCTTCATTAGAAAGGAAATTTACAAAAATACCC

TCTGTTATAAATCTCCCTTTTCAGCCAATAAAAAGTCAACCTAGGTTTTCTACTTCAGAT

ATACCTAGTACCTCCATTCGATCAGTTGATTACACCACTAGTGTTCCTCATCCGATCTAC

ACTAGTAATCAACATGTACAAAGTCAGGAAGAGAAGGAACCTTCTCCTCCAACTTCCCCT

ACGTTTTCTGCTGTTACAGAAAATGTTATTAATGTCATTGAAAAAGAATTTGAATTAGAT

AAAACTTCTCTGCATAATGATTTTTATTCTGATTTAAACAAAGAAAAAAGACTTTGGTTT

TTCAAACATTTTTTAAACCAAAGAAAAGAAATCCAACAAACTTATTATGAATTTGTGAAT

TTTCATAAAGTTCATATATTGTTTTTTGATTGGTTCGAAATATATTCTTCTGATAACAAC

ATCTCTTATCCTTTTAAAGAGTCAAACCCTATTACTATTAGGAAGAAAGTTCCTGAATGG

AAACTTCTTGATAGTGATAGAACTATAGAATCTGAACACCCACCTCTTCGGAGTGTAACC

GTTGATCACGGTGAACCTCCTGTCCAAATTCGAGCTTCACCTTACAAAATTCCAAAACCA

AATGATTCTGAGGCAAATTTAAGTAGTATTATCCAACAGAATAATTTCTGTAATACTAAC

TTAAATACAATCGGAAAGCAGTTGACTAGGATAGAAAACCAAATCCAGAAGTCAACTATT

ACTGTTCCATCAATTTCTCCTATTCCAACAAAATCGGATTCTGACAAAAAGCTTAAGGAA

CCTATTTTCAAACCCTTTCAGGTTTCGAAAACTAGCCAAAAGCTTGTTCAAGAGTCAAAA

TCAGATTTTGCTAAAGCCATTAGAGAACAATTAGATAGGATAGAAGCTGCTTCTTCCTCA

TCTAGCAAAGTTCAGATAGCCCCTGATACTCCTCAATCTAGCAAGATTGGAGTATTAGAA

CATGACCAAATGTCTATAGCCTCTTCTGATATAGAAGCCTTCAAAGAAGAACCCACTCCT

AAAGCCAACAAAATTCATTGGGAATTAGCCCTTCCCACTGTCAAGACTCCACCGGATCTA

GCAATAGATAATAGACCAAGTGCATTAAATCAATCCCGATATAATGCATCTTCAGTCTAT

GAGTGGAATATTGACGGCATGTCCGAATACAATATCCTAGGATTGTTGCAACAAATGACA

ATGGCAGCCAATGCCTATAAAACCCAAGCAGGAACTTCTGATCGTGCTATATCAGAAATC

CTCATTGCCGGTTTTACTGGTCAATTAAAAGGTTGGTGGGATCATCTTCTCACTAATCAG

CAACAATTAGACATTCTAAATTCCATTCAAGTCGATGAAAATGGAGTCCCTATTTTTGAT

GAGTTCAACAATCCAATTCAGGATGCTGTTGCTACTCTAATTTTAACCATTTCCCTCCAT

TTCATTGGTGACCCTTCACACCTTCGTGATAAAAACGCTGAGTTACTACATAATTTAAGA

TGTAGGAAACTTAGTGAATTTCAAAGTTACAAAACCTCTTTTTTTACCAGACTATTTCTA

AGGGATGATGCAAATCATATAACTTGGAAAGAAAAATTTCTCGCTGGATTACCTACTCTT

CTAGGTGAAAAGGTAAGAAATTCCATCAAAGCCCTTTATGATAACCGTATTCCTTATGAC

GAGCTCACCTATGGTGAACTCGTCAGTTTTGTCAATAAAGAAGGATTAAAGATTTGTCAA

GATTTGAAATTACAGAAACGACTTAAGTGGGAGCTTAAGAAGTCTAAACAAGAATTAGGC

AGTTTCTGTAAACAATTCAATTATGACCCCTTTAAAACTTCTAACTCCAAAGATTGTAAT

GGCGAGTGTTCTACTAAACCCCGCAGGAAACATTACAAATCAAAAAATTTTAGGAAACCC

TTTCGTAATTTTAGAGAACTTCCTTATAAGAAACCTTCGAGGCCTTATAAGAAACCCAAA

TTCTCTAAAAGAAAAGAGTTTAGAGCCAAACCAAAGACCCCTTTCAATTACAAAGAAGCT

ATATGTCATAAATGTGGCATGAAAGGTCATACTGCAAATTATTGCAGAATGAACAAAAAG

CTTCATGAACTTGACCTCGATGAAGAGATCCTTTCCAAATTAGCCCCCCTTCTTGTCGAA

TCTTCTGATTCCGAATCTTCCATGTCGGGAGACAGTGATCCATATCAAGTTGATGAACTT

TTTGACTCAGATGACTCTGTATCTAGCAGTAGTGAATCTGAATCTGATTCATATTTAAAG

AAAATCAATGTTTTGACTAAAGACCAAGAAACTTTTCTTGAACTTGTAAAGCATATTTCT

GATCCAAATCTTCAAAAAGAATATCTTGATAAACTTTTGAAAACTTTGGATTTTAATAAA

GCCGAGACTTCTAAAGTCCCAATTGTTAAAAAGAATTCTTATGATCTTACTCAAATTTTG

GATAAAAAGAAAACAAAGAAGACGGTTCCTAATATCCAAGATCTTCAGAAAGAGATAAAA

GAAATCAAATGTGAAATCAGAGATTTAAAAGAAAAACAAAAAAGTGATTCTGATACTATC

CAACTTCTTTTACAAAAACAATTGCAGGATAATTCAGACAATGAATCTAATCCTGATGAT

GGTGATGATATTAAAGTAGAAAACATTGAGTCAGTACCCAATGATTTTCTATTTGTTTTA

AAGCAAATCACCACAAGAAAGTATTTGATTAAAGTCACTTTAATCTTTTCTGATGATTTT

GCAATGGACGCCATTGCCCTTTTTGATACTGGCGCTGATTTAAATTGCATTAGAGAAGAC

ATCGTCCCCAAAAGATTTCATGAAAAAACAAAAGAAAGACTTTCTGCCGCTAATAATTCA

AAACTGAATGTCAGTTCCAAGGTTGAAGCCTCAATCCATAATAATGGTTTTGATTTTAAA

ACTTCTTTTGTCCTCACAAATGATATTCATCATGCCATCATTTTGGGAACTCCTTTTATA

AATCTTATAACTCCATATACTGTCAATTATGATAGTATATCCTTCAAAGCAAAAAACAAG

AAACTTGTTTTCCCTTTTATTGAAAAACCTAAAACAAGGAATTTGAATATTGTTAAAGCC

TGTTCTGTTTATCAAAACAAAATTAATAACATACTTAAATCAAAACAATATGATTTAATG

TGTTTACAAAAGGATTTAAACTTACAAAGAATTGAAAGCCAATTACAAAATGATTTCATA

AAAAGAAAAATCTCTGATTTCAAAACTCTCATTGAAAAAGAAATTTGTGCTGATCTACCA

TCTGCTTTTTGGAACAGAAAACAACACATGGTAGATCTACCTTATGAAACTTCTTTTAAT

GAAAGACAAATCCCCACTAAAGCACGTCCAATCCAGATGAACATGGAATTGGAACAACAT

TGCAAAAATGAAATCAAAGATTTAGAGTCAAAAGGACTCATTGTTAAATCAAGGTCCCCA

TGGTCTTGTGCCGCTTTTTATGTTAATAAAAATTCTGAAATTGAAAGAGGCACTCCTAGA

TTAGTTATAAACTACAAACCTCTTAATAACGCTCTAAAATGGATTAGGTATCCAATACCT

AATAAAAAGGATTTACTCCAAAAACTATGTTCTGCTCTTATTTTTTCAAAATTCGACATG

AAATCTGGTTTTTGGCAAATTCAAATTCATCCTAATGACCGTTACAAAACTGCTTTTACT

GTTCCATTTGGACAGTATGAGTGGACTGTTATGCCCTTTGGTTTAAAAAATGCACCCTCA

GAATTTCAAAGAATCATGAACGATATTTATAATCCTTATTCTGATTTTTGCATCGTTTAC

ATTGACGATGTATTGATATTTTCAAGAACTATCGATCAACATTTCAAACATTTAAAGACT

TTTTATTTTGCCACAAAAAAGGCTGGATTAGCAATTTCCAGTTCAAAAATTTCTTTATTC

CAAACAAAAGTCAGATTCCTTGGTCATCATATTTCCAAAGGAACTATCACTCCAATAGAG

AGATCACTTTTGTTTGCTGATAAATTCCCAGACAAAATTCTGGACAAAACCCAATTACAA

AGATTTCTTGGAAGTTTAAATTATGTTCTCGATTTCTGCCCTAACATTAATAGGATGTCT

AAACCTTTGCATGATAGGTTGAAAAAGAATCATGTTGCATGGACAGAAGAACATACCAAA

GTTGTAAAACTAATAAAGAAATCTGTGAAAAACATCCCATGCTTATATCTTGCAAATCCT

GCATTACCTAAAATTGTTGAAACTGATGCATCTGATATAGGTTATGGAGGCATATTAAAA

CAAAAAGAAAATGACAAAGAACAGATAGTACAATATGTTTCTGCACATTGGAATGATTGC

CAGAAAAATTATTCTACTATCAAAAAAGAAATCCTTTCCATTGTTTTATGCATAACAAAA

TTTCAAAGTGATTTACTAAATCAAAAATTTCTACTTAGAATTGATTGCAAAGCTGCAAAA

CATGTTTTAGAAAAAGATGTTCAAAACATTGCATCAAAACAGATTTTTGCACGATGGCAA

GCCATTTTAAGTGTTTTTTATTTTGATATTGAATATATTAAAGGTGACAAAAATTCTATT

CCTGATTTTCTAACCAGAGAATTTCTTCAAAACAGATAATGCCGCCAAAGAAGAAAGACA

AAGGTAAAGCTGTTCTCAAAGATTCTGAATCTAAAGCAACCTCTAAAGAACCCCAAGCTA

CCCCTTCTAAAGACAAATTACTTTCCTCAGCCATGCCTATTAAATCTTGGATAGAAATGG

TTGAAGAACATGGAGCCCAATACAAAACCACTTCTTCTGATGACCAAGTAAAACAATGGA

TGAGTTCCATTACAAAATCCCCAGAGCTCATGCTCGCCCTACAAAACCTTTCCCAAAGCC

AAACTTCCCCAAAAGAGAAAAGAAAACCCTATCTCCAAAGAAATCGAAAAACCCTCTTCT

CAAAATATGATTGTCTCTGCTGAAAGCTCATCTTCCCAAATTGTGCTTTCCCAACCAACA

CCTTCAAAGAAAACCTCCGATTGGTTTGATAAAACCCATTTTCAAAACATTCTTTCTATA

GAAGATGGATTTTACCATACTGATCCTTTCCAAACAATTTCAAAGTTTTTCCCTAAGGGC

TGGTTTTTCAAACCATGGGATTTAACAAAACCCCAGTCCTACTATCAAAGCATTTTAGAA

ATAACTGAATCAGTTAAATTCAAACATTTCTTTCTTGATAAAACCCATTCTGAGCCGGCT

TATTCCACGGCCACAATCTTAAAAGTTTTGAGCCCAAACCAGTGGGGTGATCTACTCCAC

AATTTCAAAACCTTTCCTTTAAATTTTGAGACCCGTTTACCACATTGTCGGACTTTTTCC

TACTGGGATTACCAACAAGCCTGGTATAACACCTTTTTTATCCAAAACCCTAAAAAATCT

CATTCTTGGTTATTTTTCTTTAATTCAAAAATAACCGTCCAAAGCCTACCTAATTGGTTC

CATCATTGGTGGAACTTTTTCGGACCAATACCTCAAATCCTAACTCCAAATGCCACAAAT

TGTTTAAACCTCTTTAAAACCCATTATAATCCTTCTGACTCAGAGAAAAGATTTCCCCCC

TTTCTCTGTTTTTGTACAAACTTCTTCCTACCATGGGTCTGGATGTGGAACCTCCGATTT

CATACCCATGAGTCCCAAGTCATCCTTCAAAGAACCTTCAAAGTCAAATGGTGGTCAAAA

TTTGATGAACAGTCCAAATTGACTGTTAACATGATCAAAGATTGGCTCTCCTCTAAAAAC

CTCCTACAACCAACCCTAGAAGCCTCAAAAGCCCAACAAACCTTCCTAACCCAAAAATCC

AAAGCCCAATCTCTATTGGCAAGCGCCAAAACTGAAGATGAATATTTTAAAGCCATGGAA

CAGCTCCTCGCCTCAAGATCAAAATCCTCAGTTGCAGACTCCTCTGAAGATGAAGATGAT

GATGAAGACGAAGAGCCATTCATTTCCCTTGGAGACGAAAACGAAGATGATTGTTTCGGC

ATTTTCTTCTCCAGTAAAGCATTGTAAATAATTATTTGATTATTTACTTAAAAAAAAAAT

TTATGTATTTTCGGGTGGTCCCCTGGACACAAGTGAGAGCGCACATGTCTCTTCACCCGT

ATTGTATGTCAGTCTGTATTTCAGTATTTGTACAAAGCATCTACTATTCAAAGATACGAT

CCAATTGTTACTGTGCACTTAAAATCCCGCTACAGTGAACAGTGCAGATTTCCAGATGTT

ACTGTGCACTTAAAATCCCGCTACAGTGAACAGTTTAAAGATCTTTGTATAAAAACCGAG

AGGAAGACTCAGACTCCTCAGGTTTTTCATTTCTCATTTTTAGAGCTTCTCTCTCTACTT

CTCTTCCTTCTCTCTAAAACTCTCTCTCTCTCTCTCT

>CclemV_scCc2 [endogenous-virus-name=Citrus clementina virus] [strain=sequence cluster Cc2] [host=Citrus clementina] [moltype=genomic DNA] [note=complete genome, mutations in ORF1] 7718 bp

TGGTATCAGAGCCAAGGGGAGGATAGTGTAATACAGTTTAGAGTTTTTGTGTTTAAAGAC

TTGTGTTTTATTTAAATTTTGTTTGTGTGGTGAATTTTTACTGGTAGTAAGTCCCGTTTT

AGGATAAGGATTTCCGCTTAGGGCTGTAAAACCATACCTGTAGCCATTCAACCAAGACCT

TAGATTGACCATAAGTTCTTTCTTCATGGATCCTCTGCTCTGTAGAGCATCGTCCTTTTC

TAGCTCTTCTTCCGGGAAGACTAGTGATTCAAAGCATGTTGTGAGTTCAGAAGAATTCAT

TATTGAGAACTTTGATAAAGCAATTGATTGTTGGGAACTTCCAAAAATTTCCAAAGAAAA

GATTTACAAAACAAAAAAGCTTGATTTTTTAAAAAATGATTATGTTATAAAGACTGAAGA

ACGTGACATAACTCTTTCAGAACCATTTGAAACAATTCATTTGTTTTCAGAAAAGTCTTT

AAAGAAATTAAAAGAAAAGAATTTTAATTATATTCACATTGGTCTTATTCAAGTAGGAAT

AAAACCTTTAACAAAAGAAGGCCTGGATACTTCTATCCTCGCTGTCCTAAGAGATGGGCG

ATTCATCTCTTTTGATGATTCATTGCTAAGTAGCATCGAATCCAGTCTCTGTAAAGGTCC

GATTTCTTTTGATTGCTATCCAAACATAACAATTTCTCTTAAAGACAAAAATATTTTGAA

AAGCATGATTTTACAAATCAAAACCCATAATTATAATATGATTAAAGGATCTGTCCCAGT

TGCTTTAATTTTTAAAATTTCATATAAAGCTATGATTTCTGCGTTCAGTACGCAACATAA

ATTCCAGTAAAAAAGAGATGAGACTCTTCTCTTGCAGACTGATTTGTCTAGAGCCAACAC

TGTTATTCCAAAACCAATCCAATGGAAAGATGTCAATCTTCCAGAAGAATGGATTCTTGA

AGGAGCTGCTCCACCAGCAATTCCAAAACAACTTGAGCCTAATACAGAGTTGCAAAATGT

GACTCAGTATTCTGATGGTAAAGTCAAACTATCTTTCAGAAGATCTAACTCAACCAGATT

TTCTGATAAAGAGTCGTGCTCAAGCATTCCTTCATTAGAAAGGAAATTTACAAAAATCCC

TTCAGTTATAAATCTTCCTTTCCAACCTGTAAAGAGTCAACCTAGGTTTTCAACCTCAGA

TATTCCTAGTTCCTCTATTCGTTCTGTTGACTATACCACAAATATTCCTCATCCAATTTA

TACTAGCAGTCAACATGAACAAAAACAGGAAGAGAAGGAACCTTCTCCTCCAACTTCCCC

TACTTTTTCTGCTATCACAGAAAATGTTATAAATGTTATTGAAAAGAATTTTGAACTTGA

TAAAAATCTTCTCCATAATGATTTTTATTCAGATAATAATAAAGAAAAAAGACTTTGGTT

TTTCAAACATTTTTTAAACCAAAGAAAAGAAATCCAACAAATCTATTATGAATTTGTGAA

TTTTCATCAAGTTCATATATTGTTTTTTGATTGGTTCGAAATATATTCTTCTGAAAACAA

CATTTCTTATCCTTTTAAAGAGTCAAACCCTATTACTATTAGGAAGAAAATTCCTGAGTG

GAAACTTTCTGATAGTGATAGAACCATAGAATCTGAGCATCCACCTCTTCGGAGTATAAC

CGTTGATCACGGTGAACCTCCTGTCCAAATTAGAGCCTCACCTTACAAAATCCCAAAACC

TAATGATTCTGATGCCAATTTAAGTAGTATTATCCAACAGAATAATTTCTGTAATACTAA

CTTAAATACAATCGGAAAACAGTTGACTAGGATAGAAAACCAATTCCAAAAGTCAACCAT

TACTGTTTCTTCGATTTCTCCTATTCCATCAAAATCGGATTCTGACAAAAAGCTTAAAGA

ACCTATTTTCAAACCTTTTCAGGTTTCGAAAACTAGCCAAAAGCTTGTTCAAGAGTCAAA

ATCAGATTTTGCTAAAGCCATTAGAGAACAACTAGATAGGATAGAAGCTGCTTCTTCCTC

ATCTAGCAAAGTTCAGATAGCCCCTGATACTCCTCAATCTAGCAAGATTGGAGTATTAGA

ACAAGACCAAATGTCTATAGCCTCTTCTGATATAGAAGCCTTCAAAGAAGAACCTTCTAC

TCCTAAAGCCAACAAAATTCATTGGGAATTAGCCCTTCCCACTGTCAAGACTCCACCGGA

TCTAGCAATAGATAATAGACCAAGTGCATTAAATCAATCCCGATACAATGCATCTTCAGT

CTATGAGTGGAATATTGATGGTATGTCCGAATACAATATCCTAGGATTGTTGCAACAAAT

GACCATGGCAGCCAATGCCTATAAAACCCAATCAGGAACTTCTGACAATGCCATTGCAGA

AATCCTTATTGCCGGTTTTACTGGTCAACTTAAAGGTTGGTGGGATCATCTTCTCACTAA

TCAGCAACAATTAGACATTCTAAATTCTATCCAAATAGATGAAAATGGAGCCCCCATTCT

TGATGAGTTCAATAACCCTATTCAGGATGCTGTAGCCACCCTAATTCTAACCATTTCCCT

CCATTTCATAGGTGACCCTTCTCACCTTAGAGATAAAAACGCTGAGTTACTACATAATTT

AAGATGTAGGAAACTTAGTGAATTCCAAAGCTACAAAACCACATTCTTTACCAGACTATT

TCTAAGAGATGATGCAAATCATATAACTTGGAAAGAAAAATTCCTTGCAGGATTACCTAC

CCTTTTAGGTGAAAAGGTAAGAAATTCCATCAAAGCCCTTTATGATAACCGTATTCCTTA

TGATGAGCTCACCTATGGTGAACTAGTCAGTTTTGTCAATAAAGAAGGTTTAAAGATCTG

TCAAGATTTGAAATTACAGAAACGACTTAAATGGGAGCTTAAGAAGTCTAAGCAAGAATT

AGGCGGTTTCTGTAAACAATTCAATTATGACCCCTTTAAAACTTCTATCTCCAAAGATTG

TAATGGTGAGTGTTCTTCTAGACCTCACAGGAAACATTACAAATCCAAAAGCTATAGGAA

ACCATTCCATAATTTTAGAGAATCTTCTTATAAGAAACCTTCGAGGCCTTATAAGAAACC

CAAATTCTCTAAAAAAAAGAGTTTAAAGCCAAACCAAAGACTCCTTTCAATTACAAAGAA

GCCATATGTCACAAGTGTGGCATAAAAGGCCATACTGCAAAATATTGCAGAATGAACAAA

AAACTTCATGAGCTTGACCTTGATGACGAAATCCTTTCCAAATTAGCCCCTCTTCTTATA

GAGTCTTCTGATTCCGAGTCTTCCATGTCAGGAGACAGTGATCCTCATCAAGTCGATGAG

CTTTTTGACTCAGATAACTCTGCATCTAGCAGTAGTGAATCTGAATCTGATTCATTTTTA

AAGAAAATCAATGTTTTGACTAAAGACCAAGAAACTTTTCTTGAACTTGTAAAGCATATT

TCTGATCCAAATCTTCAAAAGGAATATCTTGATAAACTTTTGAAAACTCTGGATTTTAAT

AAAGCCGAAACTTCTAAAGTTCCAATTGTTAAAAAGAATTCTTATGATCTTACTCAGATT

TTGGATAAAAAGAAAACAAAGAAAATTGTCCCTAATATCCAAGATCTTCAAAAAGAAATC

AAAGATATTAAACTTGAGATTAAGGATTTGAAAGAAAAACAAAAGCATGATTCCGAGACT

ATTCAACTTCTTTTACAGAAGCAATTGCAAGATAATTCAGATAATGAATCAAATCCTGAT

GATGGTGATGACAATGACCAAAATTTAGAAAATATTGAGTCTGTACCCAATGATTTTCTA

TTTGTTTTAAAACAGATCACCATGAGAAAGTATTTGATTAAAATCACTTTGATTTTTTCT

AATGATTTTGCAATTGACGCCATTGCCCTTTTTGACACTGGTGCCGATTTAAATTGCATA

AGAGAGGACATTGTCCCCAAAAGATTTCATGAGAAAACAAAAGAAAGACTTTCTGCCGCC

AACAATTCAAAGCTTAATGTAAATTCTAAAGTCGAAGCTTCAATTCATAACGATGGTTTT

GAATTTAAAACTTCTTTTGTTCTTACAAATGACATACACCATGCTGTCATTTTAGGAACC

CCTTTTATAAATCTTATCACACCTTATACTGTCAATTATGATAGTATATCTTTTAAAGCA

AAAGACAAAAAGTTTGTTTTCCCATTTATTGAAAAGCCAAAAACAAGAAATTTAAATATT

GTTAAAGCATGTTCTGTTTATCAGAATCAAATCAACAATCTTCTCCGATCAAGACAAAGT

GATTTAATGTTTTTACAAAAGGATTTAAGTTTAAAAAGAATTGAAAGCCAATTACAAAAT

GAGTTCATCCAAAAGAAAATCTCTGATTTTAAAGATCTAATTGAGAAAGAAATTTGTGCC

GATCTACCTTCAGCCTTTTGGAATAGGAAACAACATTTGGTAGATTTGCCTTACGAGAAT

TCTTTTGATGAAAGACAGATACCTACTAAAGCTCGTCCAATCCAAATGAATATGGATTTG

GAACAACATTGTAGAACTGAAATAAAAGACTTGGAGTCTAAAGGACTCATTGTAAAATCT

AGATCCCCATGGTCTTGTGCCGCTTTTTATGTAAACAAAAATTCTGAAATTGAAAGAGGA

GTACCAAGATTAGTCATAAATTATAAACCCCTTAATAAAGCATTAAAATGGATTAGGTAC

CCAATACCTAACAAAAAGGATTTGCTTCAAAAACTGCATTCTGCTTTCATATTTTCCAAA

TTTGACATGAAATCGGGTTTTTGGCAAATTCAAATTCATCCAAAAGACCGTTATAAAACC

GCTTTTACTGTTCCTTTTGGACAGTATGAGTGGACTGTTATGCCATTTGGGTTGAAAAAT

GCGCCTTCAGAATTTCAAAGAATTATGAATGACATTTATAACCCTTATTCTGATTTTTGC

ATTGTTTATATCGATGATGTTTTGATTTTTTCTCAAACCATCGACCAACATTTTAAACAT

CTAAAGACCTTTTACCTTGCTACCAGAAAAGCTGGTTTAGCAATCTCTAAGTCTAAAGTC

TCTTTGTTTCATACAAAGATCAGGTTCCTAGGTCACCATATTTCCAAAGGAACCATTACC

CCTATTGAGCGTTCTCTTGCCTTTGCAGACAAGTTTCCTGATAAAATCCTTGATAAAACT

CAATTACAAAGATTTCTAGGAAGTCTAAATTATGTTCTTGATTTCTGTCCAAATATCAAT

AGGATGTCCAAACCTTTGCATGATAGGTTAAAAAAGAATCCTGTTGCCTGGACCGATGAG

CATACTAAAGTAGTTAGACTTATAAAGAATTCTGTAAAAAGCATCCCATGTTTATTTCTT

GCAAATCCTGCATTACCTAAAATTGTTGAAACTGATGCATCTGATTTAGGTTATGGAGGA

ATATTAAAGCAAAAAGATAATGATAAGGAACAGATAGTTCAATATGTTTCTGCACATTGG

AATGAATGCCAAAAGAATTATTCAACCATCAAAAAAGAAATTCTTTCCATTGTTTTATGC

ATTTCAAAATTCCAACATGATTTATTAAATCAAAAATTTCTACTTAGAATTGATTGCAAA

GCTGCAAAACATGTTTTAGAAAAAGATGTTCAAAACATTGCATCAAAACAAATTTTTGCA

CGATGGCAAGCCATTTTAAGTGTTTTTGATTTTGATATTGAGTTCATTAAAGGAGATACA

AATTTTATTCCTGATTTTCTAACCAGAGAATTTTTGCAAAGCAGATAATGCCGCCGAAGA

AAAAAGACAAAGGTAAAGCTGTTCTCAAAGATACCGAACCTACAAAAACCTCCAAAGAAC

CTCAATCTACTCCCTCTAAAGAAAAATTACTTTCTTCAGCCATACCCATTAAATCCTGGA

TTGAATTGGTTGAAGAAAATCAAGGAACCCAATACAAATCCATCTCCTCTGAACAACAAG

TCAAAGAGTGGATGGAATCTATTACAAAGTCCCCTGAGCTTATGCTTGCCTTACAAGGCA

TCTCAAAATCTAAAGCCCTTTCTCAGATCCCTGAGGAAGAAAAACCAATTTCTAAAGAAA

TTACAAAGTCTTCTTCCCAAAGCCAAACTGTTGTTCTTTCTGGTGAAAGTTCATCTTCTC

AGATTGTTCTTTCCCAGCCAACACCTTCAAAGAAAACTTCCGATTGGTATGATAAAACCC

ATTTTCAAAATGTTTTAACTATGGAAGATGGATTTTACCACACTGATCCTTTCCAAGCAA

TCTCAAAGTTTTTTCCTAAAGGCTGGTTTTTCAAACCATGGGATTTAACAAAACCCCAGC

CTTATTATCAAAGCATTTTAGAAGCCACTGAGTCTGTAAAATTTAAACATTTCTTTCTCA

GTGAAACCCATTCAGAGCCGGCCTACTCCACGGCCACTATTTTAAAAGTTTTAAGCCCAA

ACCAGTGGGGTGACCAACTCCACAAATTCAAAACTTTCCCTCCAAGTTTTCAAATGCGTT

TACCACACTGCCTGGCCTATTCCTATTGGGACTACCAGCAAGCTTGGTTCAACACATTTT

TCTTACAAAACCCAAAAAAGTCCCATTCATGGTTATTTTTCTTCAATTCAAAAATAACCG

TCCAAAGTCTTCCAAATTGGTTCCAACAGTGGTGGAATTTTTTTGGTCCAACACCACAAA

TCCTAACTCCAAATGCCACTCATTGTTTAAACCTTTTCAAAACCCATTATTCCCCTTCAG

ATTCAGAGAAACGGTTTTCTCCCTTCCTCTGTTTTTGTACCAATTTCTTCCTCCCATGGG

TATGGATGTGGAATTTCAGATTTCATACTCATGAAAAACAACTCATCATACAAAGAACTT

TCAAAGTCAAGTGGTGGTCAAAATTCGATGAACAAACCAAATTGACTGAATCCCTCATCA

AAAATTGGCTTTGTTCAAAAGGCTTTCTACCACAAACCATTAAAGACTCCAAAGCCCAAC

AAACCTTCCTAACCCAAAAATCCAAAGCCCAATCTCTCCTGGCTAGTGCCAAAACTGAAG

ATGAATACTTCAAAGTCATGGAACAACTCCTCACCTCCAGATCAAAGTCTTCAGTAGCTG

ACACCTCTGAAGAAGAAGAAGAAGATGAAGAGCCATTTGATGAAGAACCATTCATTTCTC

TTGGAGACGATAATGAAGATGACTGTTTCGGAATTTTCTCTCCAGTAAAGCATTGTAAAT

AATTATTTGATTATTTACTTAAAAAAAAAGAATTTTTATTCGGGTGGTCCCCTGGACACA

AGTGAGAGCGCACATGTCTCTTCACCCGGGGTGTATTTTGTATTTTGTATTTTGTATTTT

GTATTTTGTACAAAGCAACAGCACCGCTACAGTGCCAATTTCCAAAGTTTACTGTGCACT

TAAAGCTCCGCTACAGTCTACAGTGCCAATTTCCAAAGATTACTGTGCACTTAAAGCTCC

GCTACAGTGAACAGTTTAAAATGTTTCTATAAAAACCGAGAGGAAGACTTAGACTCCTCA

GGTTTTTCATTTCTCTTCTTCTCTCCCTTCTCTCTAAAAAACTTCTTCTCTCTTCTTCTT

CTCTTACTACTCTCTCTCTCTCTCTCTTCTTTCTCTCCTGAGACAAAAGAAGTTTTCTGT

ATTCAAGGAATTTTCCTATCATCGATCCTATTTCCTTC

>CclemV_scCc3 [endogenous-virus-name=Citrus clementina virus] [strain=sequence cluster Cc3] [host=Citrus clementina] [moltype=genomic DNA] [note=incomplete genome, mutations in ORF1] 7592 bp

TGGTATCAGAGCCAACGGGAGAAAAGTGTTTTATTGTTTAGAGTTTTCGTGTTTTAAAAT

CTTGTGTTTTATTTTGAAGTTTTGTTTTTGTGGTGAATTTTTACTGGTTGTAAGTCCCGT

TTTAGGATAAGGATTCCGCTGAGGGCTGTAAATCCATACCTGTAGCCATTCTAACCAAAC

CAAAAATAGACCATAAGGTCTTGACCATGGACCCTCTACTCGGTAGAACATCGTCCTTTT

CTAGCTCTTCTTCCGGGAAGACTAGTGATTCAAAGCATGTTGTGAGTTCAGAAGAATTCA

TTATTGAGAACTTTGATAAAGCAATTGATTGTTGGGAACTTCCAAAAATTTCCAAAGAAA

AGATTTACAAAACAAAAATGCTTGATATTTTAAAACATGATTATATTATAAAGACTGAAG

AACGTGACATAACTCTTTCAGAGCCTTTTGAAACAATTTATTTGTTTTCAGAAAAATCTT

TAAAGAAATTAAAAGAAAAGAATTTCAAATACATTCACATTGGTCTTATCCAAGTCGGAA

TAAAGCCTTTAACAAAAGAAGGTCTAGACACTTCTATTTTAGCTGTCTTAAGAGATGGGC

GATTCATCTCTTTTGATGATTCATTGCTAAGTAGCATCGAATCTAGTCTCTGTAAAGGTC

CAATTTCTTTTGATTGTTATCCTAATATAACAATTTCTCTTAAAGACAAAAATATTTTAA

AAAGCATGATTTTACAAATCAAAACCCATAATTATAATATGGTCAAAGGGTCTATCCCAG

TTGCTTTAATTTTTAAAATTTCATACAAAGCTATGGTCTCTGCGTTTAGTACGCAACACA

AATTCCAGTCAAAAAGAGATGAAACTCTCCTCTTGCAGACTGACTTGTCTAAAGCAAACA

CTGTCATTCCGAAGCCAATCCAATGGAAAGATGTCAATCTTCCAGAGGAATGGATTCTTG

AAGGAGCCGCCCCACCGGCGATTCCGAAACAACTTGAGCCCAATACAGAGTTGCAGAATG

TGACTCAGTATTCCGATGGTAAAGTCAAACTATCATTCAGAAGGTCTACCTCATCCAGAT

TTTCTGATAAAGAGTCGTGCTCAAGCATTCCTTCACTAGAAAGGAAATTTACAAAAATAC

CCTCTGTTATAAATCTCCCTTTTCAAAATACAAAGAGTCAACCTAGGTTTTCTACTTCAG

ATATACCTAGTACCTCCATTCGATCAGTTGATTACACCACTAGTGTTCCTCATCCGATCT

ACACTAGTAATCAACATGTACAAAGTCAGGAAGAGAAGGAATCTTCTCCTCCAACATCAC

CTACGTTTTCTGCTGTTACAGAAAATGTCATTAATGTCATTGAAAAGGAATTTGAATTGG

ATAAAACTCTTCTGCATAACGATTTTTATTCTGATTTAAACAAAGAAAAAAGACTTTGGT

TTTTCAAACATTTTTTAAATCAAAGAAAAGAAATCCAACAAATCTATTATGAATTTGTGA

ATTTTCATAAAGTTCATATATTGTTTTTTGATTGGTTCGAAATATATTCTTCTGAGAACA

ACATTGATTATCCTTTTAAAGAATCAAACCCTATTACTATTAGGAAGAAAATTCCTGAAT

GGAAACTTCTTGATAGTGATAGAACTCATAGAGTCTGAACACCCACCTCTTCGGAGTGTA

ACCGTTGATCACGGTGAACCTCCTGTCCAAATTAGAGCTTCACCTTACAAAATTCCAAAA

CCAAATGATTCTGAAGCCAATTTAAGTAGTATTATCCAACAGAATAATTTCTGTAATACT

AACTTAAATACAATCGGAAAGCAGTTGACTAGGATAGAAAACCAATTCCAGAAGTCAACC

ATTACTGTTTCTTCGATTTCTCCTATTCTACCAAAATCGGATTCTGACAAAAAGCTTAAG

GAACCTATTTTCAAACCTTTTCAGGTTTCGAAAACTAGTCAAAAGCTTGTTCAAGAGTCA

AAATCAGATTTTGCTAAAGCCATTAGAGAACAACTAGATAGGATAGAAGCTGCTTCTTCC

TCATCTAGCAAAGTTCAGATAGCCCCTGATACTCCTCAATCTAGCAAGATTGGAGTATTA

GAACAAGACCAAATGTCTATAGCCTCTTCTGATATAGAAGCTTTCAAAGAAGAACCTTCT

ACTCCTAAAGCCAACAAAATTCATTGGGAATTAGCCCTTCCCACTGTCAAGACTCCACCA

GATCTAGCAATAGATAATAGACCAAGTGCATTAAATCAATCCCGATACAATGCATCTTCA

GTCTATGAGTGGAATATTGACGGTATGTCCGAATACAATATCCTAGGATTGTTGCAACAA

ATGACAATGGCAGCCAATGCCTATAAAACCCAAGCAGGAACTTCTGATCGTGCCATATCA

GAAATCCTCATTGCCGGTTTTACTGGTCAACTAAAAGGTTGGTGGGATCATCTTCTCACT

AATCAGCAACAATTAGACATTCTAAATTCCATTCAAGTCGATGAAAATGGAGCCCCTATT

CTTGATGAGTTCAACAATCTAATTCAGGATGCTGTTGCTACTCTAATTTTAACCATTTCC

CTCCATTTCATTGGTGACCCTTCACACCTTCGTGATAAAAACGCTGAGTTACTACATAAT

TTAAGATGTAGGAAACTTAGTGAATTTCAAAGCTACAAAACCTCATTTTTTACCAGACTA

TTTCTAAGAGATGATGCAAATCATATAACTTGGAAAGAAAAATTTCTTGCTGGATTACCT

ACCCTTCTAGGTGAAAAGGTAAGAAATTCTATTAAAGCCCTTTATGATAACCGTATTCCT

TATGATGAGCTCACCTACGGTGAACTTGTCAGTTTCGTCAATAAAGAAGGTTTAAAGATT

TGTCAAGATTTGAAATTACAGAAACGACTTAAGTGGGAGCTTAAGAAGTCTAAACAAGAA

TTAGGCGGTTTCTGTAAACAATTCAATTATGACCCCTTTAAAACTTCTATCTCCAAAGAT

TGTAATGGTGAGTGTTCTACTAAACCTCGCAGGAAACATTACAAATCTAAAAATTTTAGG

AAATCCTTTCGTAATTTTAGAGAACTTCCTTATAAGAAACCTTCGAGGCCTTATAAGAAA

CCCAAATTCTCTAAAAAAGAGTTTAAAGCCAAACCAAAGACTCCTTTCAATTACAAAGAA

GCTATATGTCATAAGTGTGGCATAAAAGGTCATACTGCAAAATATTGCAGGATGAACAAA

AAGCTTCATGAGCTTGACCTTGATGATGAGATCCTTTCCAAATTAGCCCCTCTTCTTATC

GAGTCTTCTGATTCCGAGTCTTCTATGTCAGGAGACAGTGATCCATATCAAGTTGATGAG

CTATTTGACTCAGATGACTCTGCATCTAGCAGTAGTGAATCTAAATCTGATTCATTTTTA

AAGAAAATCAATGTTTTGACTAAAGACCAAGAAACTTTTCTTGAACTTGTAAAGCATATT

TCTGATCCGAATCTTCAAAAAGAATATCTTGATAAACTTTTGAAAACTTTGGATTTTAAT

AAAGCCGAGACTTCTAAAGTCCCAATTGTTAAAAAGAATTCTTATGATCTTACTCAGATT

TTGGATAAAAAGAAAACAAAGAAGTCAACACCAACAATTCAAGATATTCAAAAAGAAATC

AAAGACATTAAACTTGAAATAAAGGATTTGAAAGAAAAACAAAAAAGTGATTCTGAAACT

ATCCAACTTCTTTTACAAAAGCAATTACAAGATAATTCGGATAATGAATCCAATTCTGAT

AATGGTGATAAAAATGATCAGAACTTAGAAAACATTGAGTCTGTACCCAATGATTTTCTA

TTTGTTTTAAAACAAATCACCACAAGAAAGTATTTGATTAAAATCACTTTAATTTTTTCC

AATGATTTTGCAATCGACGCCATTGCCCTTGTTTGACACTGGTGCCGATTTAAATTGCAT

AAGAGAAGACATTGTCCCCAAAAGATTTCATGAGAAAACAAAAGAAAGACTTTCTGCCGC

CAATAATTCAAAACTCAATGTCACCTCTAAAGTTGAAGCTTCAATACATAATAATGGATT

CAAATTCAGAACTTCTTTTGTTCTTACAAATGACATACATCATGCTATCATTTTAGGAAC

TCCTTTTATAAATCTTATAACCCCATATACTGTCAACTACGATAGTATATCTTTCAAAGC

AAAAAATAAAAAATTTGTTTTCCCGTTTATTGAAAAACCAAAAACAAGGAATTTGAATAT

TGTTAAAGCCTGTTCTATTTATCAAAACCAAATCAATAACATGCTTAAATCAAAACAAAA

TAATTTAATTTGTTTAAAAAAGGATTTAATTCTACAAAGAATTGAAAATCAATTACAAAA

TGATTTCATACAAAGAAAAATCTCCGATTTCAAAAATCTCATTGAAAAAAAAATCTGTGC

TGATCTACCATCTGCTTTTTGGAACAGAAAACAACACTTAGTAGATTTACCTTATGAAAC

TTCTTTTAATGAAAGACAGATCCCCACTAAAGCACGTCCAATCCAGATGAACATGGAATT

GGAACAACATTGCAAAAATGAAATAAAAGATTTAGAGTCAAAAGGACTCATTGTAAAGTC

AAGATCCCCATGGTCTTGTGCCGCGTTTTATGTTAATAAAAATTCTGAAATTGAAAGAGG

CACACCAAGACTTGTAATAAACTACAAACCTCTTAATAAAGCATTAAAATGGATTAGGTA

TCCCATACCTAATAAAAAGGATTTGCTTCAAAAACTATGTTCTGCTCTTATTTTTTCAAA

ATTTGACATGAAATCTGGTTTTTGGCAAATTCAAATTCATCCTAAAGACCGTTACAAAAC

TGCTTTTACTGTTCCATTTGGACAGTATGAATGGACTGTCATGCCCTTTGGTTTAAAGAA

TGCACCCTCAGAATTTCAAAGAATTATGAATGATATTTATAATCCTTATTCTGATTTTTG

CATTGTTTATATTGATGATGTGTTGATTTTTTCAAATTCAATCGATCAACATTTCAAACA

TTTAAAGACTTTTTACCTTGCCACCAGAAAGGCTGGATTGGCAATTTCTAGTTCTAAAGT

TTCTTTATTTCAAACAAAAGTCAGATTCCTTGGTCATCATATTTCTAAAGGAACTATCAC

TCCAATCGAGCGATCCCTTTTGTTTGCAGATAAATTCCCTGATAAAATTCTTGACAAAAC

CCAATTACAAAGATTTCTTGGAAGTCTAAATTATGTTCTTGATTTCTGTCCTAACATTAA

TAGGATGTCTAAACCCTTGCATGATAGGTTAAAAAAAAATCCTGTTCCTTGGACAGATGA

ACATACCAAAGTTGTTAAACTAATAAAGAATTCTGTAAAAAACATCCCATGTTTATATCT

TGCTAATCCTGCATTACCTAAAATTGTTGAAACTGATGCATCAGATTTAGGTTATGGAGG

CATATTAAAGCAAAAAGAAAATGATAAAGAACAAATAGTACAATATGTTTCTGCACATTG

GAATGAATGTCAGAAAAATTATTCTACTATTAAAAAAGAAATTCTTTCCATTGTTTTATG

CATTTCAAAATTTCAAAGTGATTTATTAAATCAAAAATTTTTACTTAGAATTGATTGCAA

AGCTGCAAAACATGTTTTAGAAAAAGATGTTCAAAACATTGCATCAAAACAAATTTTTGC

ACGATGGCAAGCCATTTTAAGTGTTTTTGATTTTGATATTGAATATATTAAAGGAGATAC

AAATTATATTCCTGATTTTCTAACTCGGGAATTTCTTCAAAACAGATAATGCCGCCCAAA

AGAAGAGACAAAGGAAAAGGCATAGCCAAAGATCCTGAACCTAAAGCCACTCCAAAAGAC

TCCCAATCTTCTCCTCCTAAAGAAAAACTACTATCATCTGCCATGCCAATCAAATCTTGG

ATTGACATGATCCAAGATGAAGAAGCAAAATCTAAAGCCCTCTCTACCCAAGAGCAAGTA

AATCAATGGATGAAATCCATTTCTAAATCACCTGAGCTTATGCTCGCCCTACAAAGCAGT

CTCTCCCAAAGCCAAACTCCTCCAAAAGAAATTTCTGAAAAAGAAAGCCCAAAAGAAATT

TCAAAATCTTTTTCCCAAAATGTTGTTGTTTCTGGTGAAAGCTCGTCATCTCAGATCGTT

CTTTCACAACCAACACAGAAAACTTCGGTTTGGTTTGATAAAACCCATTCCCAAAATATT

TTAACTATTGAAGAAGGGTTTTACCATTCTGATCCATTTCAAACCCTTACAAAGTTTTTC

CCTAAAGGCTGGTTTTTCAAACCATGGGATTTAACAAAACCCCAGCCTTACTATCAAAGC

ATTTTGGAAGCCACTGAATCCGTTAAATTCAAACATTTTTTCCTCAGTGACTCCCATTCT

GAGCCGGCCTATTCAACGGCTACAATTTTAAAAGTTTTGAGCCCAAAACAATGGGGTGAT

CTACTCCATAATCAAAAAAGATTTCCCTCCAATTTTCAAATGCGTTTACCACATTGTTTG

ACTTTCTCCTACTGGGATTATCAACAAGCCTGGTACAACACATTCTTCTTACAAAACCCT

AAAAAATCCCATTCATGGTTATTTTTCTTTAATTCCAAAATAACCGTACAAAGCCTTCCT

AACTGGTTTAAACAATGGTGGAACCATTTTGGCCCAACTCCTGATATCCTTACACCAAAT

GCCACCCACTGTTTAAATTTGTTCAAAGCCCATTATAATCCCTCTGAATCAGAAAAAAGG

TTTTCGCCTTTCTTCTGTTTCTGTACAAACTTCTTTCTTCCTTGGGTTTGGATGTGGAAC

CTCAAATACCACACTCAAGATGCCCAAATTATCCTACAAAGAACCTTTAAAGTAAAATGG

TGGTCAAAATTTGACGAACAGTCAAAATTGACTGACATTTTGATCCGAAATTGGCTCTCT

GCCAAAGGATTTCTTCAACCGACCATTAAACAATCCAAAGCCCACCAAACTTTCTTGAGC

CAAAAAACAAAAGCCCAATCTCTCCTTGCCAACGCCAAGACTGAAGAAGAATACTTCAAT

GTCATGCAACAACTTCTCGCCACAAGGTCAGAGACATCTGTTGCAACTTCCTCTTCAAAC

ACTTCAGGAGATGAAGAACCTTTTATTTCTCTTGGTGATGAAAATGAAGATGACTACTTC

GGCATTTTCTCTCCAATAAAGCATTAAAGCTGTATTAAAGCCAATTTAAAACTTTACTTA

TTTGAATCTATGTACAAGTTTTGTACTTAAAAAAAAAAAAGAGTTGTTATTTCCCAATTA

TTCTACTTCTGGGTGGTCCTATGGACACAAGTGAGAGCGCACATGTCTCTTCACCCGGGG

TATATTCTACATCCGCAATATTCCAAAAAACGACACAAAGACTACAGTTACAGACGATCC

AAAGATTACTGTGCACTCAAAACTCCGCTACAGTGAACAGTGCAAAGTTCCAAAGATTAC

TGTGCACTCAAAACTCCGCTACAGTGAACAGTTTAAAAAATTTTGTATAAAAACCGAGAG

GAAGACTTAGACTCCTCAGGTTTTTCATTTCT

>CclemV_scCc4 [endogenous-virus-name=Citrus clementina virus] [strain=sequence cluster Cc4] [host=Citrus clementina] [moltype=genomic DNA] [note=incomplete genome, mutations in ORF1] 7239 bp

TGGTATCAGAGCCAAGGGAAGGATAGTGTAATATAGAATAGAGACTTTATGTTTAAAGAC

TTGTATTTTAGTTAAATATTGTTTGTGTGGTGAGTGTTACCTCTGGGTGCCTTGGTAGTA

TTTGGTTGTAAGTCCCGATTTGGTTGAAGGGCAGTAAAACCAATTCTTAGCCTTGGATTG

TAGCCATACCTACAGGACTAAAACTAGAATATAGGTTTTTATCCATGGATTCTCTACTCT

GTAGAGCATCATCCTTTTCTAGCTCTTCTTCTGGAAGAACTAGTGATTCAAAGAATGTTG

TGAATTCGGAAGAATTTGTAATTGAAGACTTTAATAAAGCAATTGATAATTGGGAATTAC

CAAAGGTTTCTAAAGAAATGATTTACAAAACAAAAAAGCTTGATTTTTTAAAGAATGATT

ATGTTATAAAAACTGAAGAAAGAGACATAACCCTTTCAAAACCTTTTGAAACTATTCACT

TGTTTTCAGAACAATCTTTAAAGAAATTAAAAGAAAAGAATTTTAATTATGTTCACATCG

GTTTAATCCAAGTTGGAATAAAACCATTAACTAAAGAAGGATTAAATACTTCAATTCTTG

CTGTCCTTAGAGATGGAAGATTCATCTCCTTTGATAATTCTTTACTAAGTAGTATTGAAT

CAAGCCTCTGTAAAGGACCCATTTCATTTAGCTGTTATCCAAACATAACAGTTTCTCTAA

AAGACAAGAATATTCTGAAAAGCATGATTTTACAAATCAAAACCCATAACTATCACATGA

TTGAAGGATCTGTCCAAGTTGCCTTAATTTTTAAAATATCCTACAAAGCCATGATTTCTG

CGTTTAACACGCAACATAAATTCCAGTCAAAAAGAGATGAGACTCTTCTCTTGCAGACTG

ACTTGTCTAAAGCCAATACCGTTATTCCAAAACCAATCCAATGGAAAGATGTCAATCTTC

CAGACGAATGGATTCTAGAAGGAGCCACTGCACCAGTGATTCCAAAACAACTTGAGCCAA

ATACAGAGTTACAAAATGTGACTCAGTATTCAGATGGTAAAGTCAAACTATCCTTTAGGA

GATCTACATCATTTAGATTCTCTGATAAAGATTCATGCTCAAGTATTCCTTCATTAGAAA

GGAAATTTTCAAAAATTCCTTCTGTTATAAATCTTCCTTTCCAACCAGTAAAGAGTCAAC

CTAGGTTTTCCACATCGGATATACCTAGTTCCTCTATTCACTCTGTTGACTATACCACTA

GTGTTCCACACCCAATCTACACTAGCAGTCAACATGAACAAAGACAGGAAGAGAAGGAAC

CTTCTCCTCCAACTTCTCCTACATTTTCTGCTGTCACTGAAAATGTAATAAATGTCCTTG

AAAAAGTTTATGAATTAGATAAAACTCTTCTTCATAATGATTTTTATTCTGATACTAATA

AAGAAAAAAGACTTTGGTTTTTTAAACATTTCATAAACCAAAGAAAAGAAATTCAACAAA

TTTATTATGAGTTTGTAAAATTGCATAAAGTCCATATATTATTCTTTGATTGGTTTAAAA

TGTATGCTTCGGATAACAATATCACTTATCCCTTTAAAGAATCAAATCCCATTACTGTTA

GGAAGAAAATCACTGAATGGAATCTTTCTGATAGTGATAGAACTATAGAATTTGAACATC

CTCCACTAAGGAGTTTGACCATTGACCATGGTGAACCTCCTGTTGAGATTAAAGCCTCAC

CCTATAAAATCCATAAACCAAATGATCCAGAGACAAATTTAGGTAGCATTATCCAACAGA

ATAATTTCTGTAATGCTAACCTAAATACAATAGGAAAACAGTTGACTAGGATAGAAAACC

AATTCCAGAAGTCAACCATAGCTGTTTCTCCTATTACATCAAAATCGGATTCTGACAAAA

AGCTTAAAGAGCCTATTTTCAAACCTTTTCAGGTTTCGAAAACTAGTCAAAAGTTTGTTC

AAGAGTCAAAATCAGATTTTGCTAAAGCCATTAGGGAACAACTAGATAGAATAGAAGCTT

CTTCTTCTTCTTCTAGTAAAATCCAAATAGCCCCAGATAGTGCCCAATCTAGTAAGATTG

GTGTACTAGAACAAGACAATATGTCTATAGCCTCTTCTGATATAGAAGCCTTTAAAGAAG

AACCTGTTCCTAAAACCAACAAAATCCATTGGGAATTAGCCCTACCTACTGTCAAGTCTC

CACCTGACTTAGCCATAGACAACAGACCTAGTGCATTAAACCAATCACGATTTAATGCAT

CCTCTGTCTATGAGTGGAATATTGATGGTATGTCTGAGTATAACATCCTAGGATTGTTGC

AACAGATGACCATGGCAGCTAATGCCTATAAAACCCAGTCGGGAACTTCTGACAAAGCCA

TTGCAGAAATTCTCATTGCTGGTTTTACTGGTCAACTTAAAGGTTGGTGGGATCATCTTC

TCACCAAACAGCAACAATTAGATATTCTAAATTCTATTCAAACAGATGAGAATGGAGCCC

CCATCCTTGATGAATTCAATAGCCCAATTCAGGATGCAGTTGCTACCCTGATTCTGACTA

TATCCCTTCATTTCATAGGTGATCCTTCACACCTTAGGGATAAGAATGCTGAGTTACTAC

ACAACCTAAGATGTAGGAAACTTAGTGAATTCCAAAGCTATAAAACCACATTCTTTACCA

GACTCTTCCTTAGGGATGATGCAAATCATATAACTTGGAAGGAAAAATTCCTTGCAGGTT

TACCTACCCTATTAGGAGAAAAGGTTAGGAATTCCATTAAAGCCCTTTATGACAATCGTA

TTCCTTATGACGAACTCACCTATGGTGAACTAGTCAGTTTTATTAACAAAGAAGGATTAA

AGATTTGTCAAGACTTGAAATTACAGAAACGACTAAAATGGGAACTTAGAAAATCTAAAC

AAGAATTAGGCAGTTTCTGTAAACAGTTCAATTATGACCCCTTTAAAGCCTCGACTTCTA

AGGATTGTAATGGTAAGTGTTCTTCGAAACCTTACAAGAAACATTACAAATCAAAAAGCC

ATAGGAAACCCTTCCATGACTTTAGAGAACCTTCCTATAAGAAACCCTCGAAGCCTTATA

AGAAACCAAATTTCTCTAAAAAGAAAGACTTTAAAGCCAAAACAAAAACCCCTTTTAATT

ACAAAGAAGCCACATGTTTCAAATGTGGAAAGAAAGGCCATACTGCAAAATTTTGTAGAA

TGAACAGAAAGCTCCATGAGCTTGGCCTTGATGAAGAAATACTTTCTAAAGTAGCCCCTT

TACTTATAGAGTCTTCTGACTCTGAGTCTTCCATGACGGGAGACAGTGAGCCTCTACAGA

TTGATGAGTTAAATGACTCTGAATCTGTATCCTCCAGTGATTCTGAAACAAGTGACACAG

AATCCTATTTAAAGAAAATCAATGTGTTGACTAAAGAACAAGAAACTTTTCTTGAACTAG

TTAAACATATTTCTGATCCAAATCTCCAAAGAGAGTACCTTGATAAACTTCTAAAAACCT

TAGAACTTAATAAAGCAGAAACTTCTAAAGTCCCTACCATTAAAAAGAATACTTATGATC

TCACTGAAATCTTGGATAAAAAGAAAACAAAGAAAACAACTCCTAATATCCAAGATCTTC

AAAAAGAAATAAAAGAAATTAAACTTGAAATAAAAGAATTAAAAGAAAAACAAAAAAATG

ATTCAGAAACTATCCAACTTTTATTACAAAAACAATTAGAAGAGAATTCTGATAAAGAAT

CAGAATCTGATGGTGATAATGAACAAAACCTTGAAAACATTGAATCTATACCCAATGATT

TTCTTTTTGTTTTAAAACAAATCACCACAAGAAAATATTTGATTAAAATCACTTTAATTT

TTTCTAAAGATTTTGAAATTGACACCATTGCCCTTTTTGACACTGGTGCTGATTTAAATT

GCATTAAAGAAGATATTGTCCCAAAAAGATTTCATGAAAAAACTAATGAAAGACTTTCAG

CCGCTAATAATTCAAAATTAAAAGTCAAATCTAAAGTTGACGCCTCAATTCACAACAATG

GTTTTGAATTCAGGACTTCCTTTCTTCTAACAAATGATATACATCATACTGTTATTTTAG

GAACTCCCTTTATAAATCTTATTACTCCATATACTGTCAACTATGATAGTATATCTTTTA

AAGCAAAAAACAAAAAACTAATTTTTCCTTTTATTGAAAAACCTAAAACAAGAAATTTGA

ATATTGTTAAAGCTTGTTCTGTTTATCAGAACCAAATCAATACACTCCTCAAATCAAAAC

AAAATGACCTGATTTGTTTAAAAAAGGATTTGAGTTTACAAAGAATCGAAAACCAATTAC

AAAATGAATTTGTAAAAAGAAAAATCTCTGATTTTAAAGACCTCATTGAAAAAGAAATAT

GTGCGGATCTACCATCTGCTTTTTGGAACAGAAAACAACATTTGGTTGATTTGCCTTATG

AAAACTCTTTTGATGAAAAACAAATACCTACCAAAGCCCGTCCAATCCAAATGAACATGG

AATTGGAACAACATTGCAAAAATGAGATAAAAGATTTAGAATCTAAAGGACTCATTGTCA

AATCTAGGTCTCCATGGTCTTGTGCCGCTTTTTATGTCAACAAAAATTCTGAGATAGAAA

GAGGAACACCTAGATTGGTAATAAATTATAAACCCCTTAATAAAGCATTAAAATGGATTA

GGTATCCTATACCTAATAAAAAGGATTTGCTCCAGAAACTACATTCTGCATTCATTTTTT

CAAAATTTGATATGAAATCTGGATTTTGGCAAATTCAAATTCATCCCAAAGACCGTTATA

AAACTGCTTTTACTGTTCCTTTTGGACAGTATGAATGGACTGTAATGCCATTCGGATTAA

AGAATGCACCTTCAGAATTTCAAAGAATTATGAATGATATTTATAATCCTTTCTCTGAAT

TTTGCATTGTTTATATTGATGATGTGTTGATCTTTTTCCAAACAATTGATCAACATTTCA

AACATTTAAAGACTTTTTATCTTGCCACTAGAAAGGCTGGTCTAGCCATTTCTAGTTCTA

AAGTTTCCTTGTTCCAAACAAGAATTAGGTTCCTTGGTCATTACATTTCCAAAGGAACCA

TAACTCCTATTGAAAGATCTCTTGCATTTGCAGACAAATTTCCTGATAAAATCCTTGATA

AAACCCAATTGCAAAGATTTCTAGGTAGTTTAAATTATGTTCTTGATTTTTGTCCTAATA

TCAATAGGATCTCAAAACCATTGCATGATAGATTAAAGAAAAATCCTGTTGCATGGACTG

ATGAACACACTAAAGCTGTTAAACAAATAAAGAATTCTGTTAAAAATATTCCATGTTTAT

TTCTTGCAAATCCTGCATTACCTAAAATTGTTGAAACTGATGCATCAGATTTAGGTTATG

GAGGAATACTAAAGCAAGTTGATAATAATAAAGAACAAATAGTACAATATGTTTCTGCTC

ATTGGAATGAATGTCAAAAAAACTATTCTACTATCAAAAAAAAAATTCTATCTATAATTC

TATGCATATCGAAATTTCAAAGCGATCTGTTAAATAAAATAAAAAATACTTAGAATTGAT

TGCAAAGCTGCAAAACATGTTTTAGAAAAAGATGTTCAAAACATTGCATCAAAACAAATT

TTTGCACGATGGCAAGCCATTTTAAGGGTTTTTTATTTTGACATTGAGTTTATTAAAAGA

GATACAAATTCTATCCCTGATTTTCTAACCAGGGAATTCCTTCAAAACAGATAAATACCG

CCCAAAAGGAAAGACAAAGGGAAAGGGATCCTCAAAGACCCTGTGTCCCAGACACCTTCT

AAAGCCTCCCAATCTTCCCCTCCTAAAGAAAAATTACTTTCATCAGTCATGCCAATCAAA

TCATTGATCGATATGATTGAAGATGAAGAAGCCAAATCTAAAGCCATCGCCCTTCAAGAA

CAGGTAAATGAATGGATGAAATCCATTTCTAAATCCCCTGAACTTATGCTTGCATTACAA

AGCTTTTCTCAAAGTCAGATTTCCCCTAAAGAAGAAAAATTCATTTCTAAAGAAATTTCT

AAAGCCTCTTCACAAAATGTAGTTCTTTCTGGAGAAAGTTCATCATCCCATATTGTTCTT

TCCCAAACAAAGCTTTCAAAGAAAACTTCAGATTGGTATGATAAAGCCCATTTTCAAAAT

GTTTTATCCATGGAAGATGGTTTTTACCACACTGATCCTTTTCAAGCAATTTCAAACATT

TTCCCTATGGGATGGTTTTTCAAACCTTGGGACTTAACAAAGCCCCAGTGTTATTATCAA

AGCATTTTGGAGTCTACTGAGTCTGTAAAATTTAAACATTTCTTCCTTAGCGAAGCCCAT

ACAGAACCAGCCTACTCCACTGCCACTATCCTAAAGGTTTTGAGCCCAAAACAATAGGGA

GACCAACTCCATAAACTAAAACCCTTCCCCGCCAATTTTCAAATGCGTTTGCCACACTGC

CTGTCCTATTCTTACTGGGACTATCAACAGGCCTGGTTTAACACATTTTTTATCCAAAAC

TCAAAAAGAACCCATTCTTGGTTATTTTTCTTCAACTCAAAAATAACCGTCCAAAGCCTT

CCAAATTGGTTCTAGCAGTGGTGGAATTATTTTGGCCCATGCCCTGAAATCCTCACCCAA

AATGCCATCCAGTGCCTAAATCTTTTTAAAACCCATTATGTACCTACAGAATCTGAGAAA

CGATTTCCTCCTTTTCTTTGCTTCTGTACAAATTTCTTTCTTCCCTGGGTATGGATGTGG

AACTTCCGATACCATCCACAAGATGCCCAACTAATTTTACAAAGAACCTTTGAAGTCAAA

TGGTGGTCAAAATTTGATGAACAGTCCAAATTGACTGTTAACATGATCGAAAATTGGTTC

TCCTCTAAAGGCCTTCTACAACCGACCATCAAAGACATTAAAGCCCAAGGGACCTTCCTT

ACCCAGAAATCCAAAGCCCAATCTCTTTTGGCCAGTGCCAAAACAGAAGAAGAATATTTC

AAAGTCATGGAACAGCTTCTCGCATCGAGATCTGAACCATCAGTTGCAAGTTCCGGATCA

AGCTCTTCTGGAGAAGCCACGCCATTCATCTCCCTTGGAGATGAGAATGAAGATGATTAC

TTCGGCATCTTTCCTCCTGTAAAGCATTGTTAAAGAATT

>CclemV_scCc5 [endogenous-virus-name=Citrus clementina virus] [strain=sequence cluster Cc5] [host=Citrus clementina] [moltype=genomic DNA] 7538 bp

TGGTATCAGAGCCAAGGGGAGGATAGTGTAAAGCAGTTTATAGTTTCGTGTTTAAAAAAC

TTGTGTTTTATTTTGAAGTTTTGTTGTTTGGTGAATTTTTACTGGTAGTAAGTCCCGCCA

TAGGATAAGGACATCCGTTTAGGGCTGTAAACCCATACCTGTAGCCATTCTGACAAAGCC

ATAGATAGACCATAGGTTTCTACTCATGGACCCTATACACTGTAGAACATCATCCTTTTC

TAGCTCTTCTTCCGGGAAGACTAGTGATTCAAAGCATGTTGTGAGTTCAGAGGAATTCAT

TATTGAGAACTTTGATAAAGCAATTGATTGTTGGGAACTTCCAAAAATTTCCAAAGAAAA

GATTTACAAAACAAAAATGCTCGATCTTTTAAAACATGATTATATTATAAAGACTGAAGA

ACGTGATATAACTCTTTCAGAGCCTTTTGAAACAATTTATTTGTTTTCAGAGAAATCTTT

AAAGAAATTAAAAGAAAAGAATTTCAAATACATTCACATTGGTTTAATCCAAGTAGGAAT

AAAACCTTTGACCAAAGAAGGCTTGGATACTTCTATCCTTGCTGTCCTTAGAGATGGTCG

ATTCATCTCTTTTGATGATTCTTTACTAAGTAGTATTGAATCTAGTCTATGTAAAGGTCC

CATATCTTTTGATTGCTATCCAAATATAACAATTTCTCTTAAAGACAAAAATATTTTAAA

AAGCATGATTTTACAAATTAAAACCCATAATTATAATATGATTAAGGGATCCATCCCAGT

TGCATTAATTTTTAAAATCTCTTACAAAGCCATGATTTCTGCATTTAGCACACAGCATAA

ATACCAGTCAAAAAGAGATGAAACCCTTCTCTTGCAGACTGATCTGTCCAAAGCAAACAC

GGTCATTCCCAAACCAATCCAATGGAAAGATGTAAATCTTCCAGAGGAATGGATCCTTGA

AGGAGCCGCTCCACCAGCGATTCCGAAACAACTTGAGCCCAATACAGAGTTGCAAAATGT

GACTCAGTATTCCGATGGTAAAGTCAAACTATCGTTCAGAAGATCTACCTCATCCAGATT

TTCTGATAAAGCTTCATGCTCAAGTATTCCTTCCTTAGAAAGGAAATTTACAAAAGTCCC

TTCAGTAATAAATCTTCCTTTCCAAAGTCAACCTAGGTTCTCTACTTCAGATGTACCTAG

TACCTCCATCCGATCAGTTGATTACACCACTAGTGTTCCACATCCAATCTACACTAGTAG

TCAACATATTCAAAGTCAGGAAGAGAAGGAACCTTCTCCTCCAACATCCCCTACATTTTC

TGCAGTCACAGAAAATGTCATTAATGTCATCGAAAAAGAATTTGAATTGGATAAAACTCT

TTTACATAATGATTTTTATTCTGATTTAAACAAAGAAAAAAGACTTTGGTTTTTCAAACA

CTTTTTAAACCAAAGAAAAGAAATCCAACAATTTTATTATGAATTTGTGAATCTTCATAA

AGTTCATATATTGTTTTTTGATTGGTTCGAAATATATTCCTCTAATAACAACATTTCTTA

CCCTTTCAAAGAGTCAAACCCTATTACTATTAGGAAAAAGACCACTGAGTGGAAACTCTC

CGATAGTAATAGAACAATCGATTCTGAGCATCCACCTCTCCGGAGTATAACCGTTGACCA

CGGCGAACCTCCTATAGACATTAGAGCTTCACCTTACAAAATCCCTAAACCAAATGATAC

TGATAGTAATTTAAGTAGTATTATTCAGCAGAATAATTTCTGCAGTACTAACTTAAATAC

TATAGGAAAACAGTTGACTAGAATAGAAAACCAATTTCAACAGTCAACCATATCTGTTTC

TCCTAGTCAAAAGCCTATCCCTTCAAAATCAGATTCTGATAAAAAGCTTAAGGAACCTAT

TTTCAAACCCTTCCAGGTTTCGAAAATTAGCCAAAAGCTTGTCCATGAATCAAAATCGGA

TTTTGCCAAAGCCATTAGAGAACAACTAGATAGGATAGAAGCTGCTTCTTCCTCATCTAG

TAAAGTTCAAATAGCTCCTGATACTCCTCAATCTAGCAAGATTGGAGTATTAGAACAAGA

CCAGGTTTCTATTGCCTCTTCTGACTTAGAAGCCTTTACCGAAGAACCTGTTTCTAAAGC

CAATAAAATCCATTGGGAACTTGCTATCCCTACTTCCAAATCTCCACCTGATTTGACTAT

AGACAACAGGCCTAGTGCATTAAACCAAGCTCGATATAATGCATCCTCTGTCTATGAGTG

GAATATAGATGGAATGTCTGAGTATAACATTTTAGGTGTATTGCAACAAATGACCATGGC

AGCCAATGCCTATAAAACCCAATCAGGAACTTCTGACAAAGCCATTGCAGAAATCCTAAT

TGCAGGTTTTACTGGTCAACTCAAAGGATGGTGGGATCATCTTCTCACTAAATTGCAGCA

ATTAGACATTTTTAATGCCATCCAAACTGATGAGAATGGTGCTCCCATTCTTGATGAGTT

TAATAACCCTATTCAGGATGCAGTTGCTACCCTGATTTTAACCATTTCCCTTCACTTCAT

AGGTGACCCTTCTCACCTTAGAGACAAAAACGCCGAGTTACTACACAACTTAAGATGTAG

GAAACTCAGTGATTTCCAAGATTACAAAACCACTTTCTTTACTAGACTCTTCCTTAGGGA

TGATGCAAATCATGTAACTTGGAAGGAAAAATTCCTTGCAGGTTTACCTACCCTATTGGG

AGAAAAGGTTAGAAATTCCATCAAAGCTCTCTATGACAATCGTATTCCTTATGACGAGCT

CACTTATGGTGAACTAGTCAGTTTTGTCAATAAAGAAGGTTTAAAGATCTGTCAAGATTT

GAAATTACAGAAACGTCTGAAGCAGGAGCTTAGGAGATCTAAGCAAGAATTAGGCGGTTT

TTGTAAACAATTCAATTATGACCCCTTTAAAGCTTCTACTTCCAAAGATTGTAATGGTAA

GTGTTCTTCAAAACCTTACAAGAAATATTACAAATCTAAAAGCCATAGGAAACCCTTTCA

CGAGTTTAGAAAACTTCCTTATAAGAAACCTTCGAGGCCTTATAAGAAACATAGTTTCTC

TAAAAAGAAAGAGTTTAAAGCCAAACCTAAAACCCCATTCAACTTCAAAGATGCCACTTG

TTTCAAATGTGGTATGAAAGGTCATACTGCAAAGTTTTGCAGAATGAACAAGAGACTCCA

AGAGCTTGACCTCAATGAGGACATCCTTTCCAAAATAGCCCCTCTTCTTGTAGAGTCTTC

TGACTCTGAGTCTTCCATGTCAGGAGACAGTGATCCTCTTCAGGTCGATGAGTTATTTGA

CTCAGATACCTCTGCATCTAGCAGTAGCGATTCTGATTCGGATTCCTATTTAAAGAAAAT

CAATGTTTTGACTAAAGACCAAGAAATTTTTCTTGAACTTGTAAAGCATATTTCAGATCC

AAATCTTCAAAAAGAATATCTTGATAAACTTTTGAAAACCATGGATTCTGACAAAGCCGG

AACCTCAACTGAAATCTCTAAGGTTCCAATTATTAAGAAGAATTCTTATGATCTTACTCA

AATTTTGGATAAAAAGAAAACAAAAAAGGCAGTTCCTAATATCCAAGATCTTCAGAAAGA

GATAAAAGAAATCAAATGTGAAATCAGAGATTTAAAAGAAAAACAAAAAAGTGATTCTGA

GACTATCCAACTTCTTTTACAAAAACAATTGCAAGAAAATTCAGACAATGAATCCAATCC

TGATGATGGTGAGGATATTAAAGTAGAAAACATAGAGTCTGTACCCAATGATTTTCTATT

TGTTTTAAAGCAAATCACCACAAGAAAGTATTTGATTAAAGTCACTCTCATCTTTTCTGA

TGATTTTGCTATGGACGCCATTGCCCTTTTTGACACTGGCGCTGATTTAAATTGCATTAG

GGAAGATATCGTCCCCAAAAGATTTCATGAAAAAACAAAAGAAAGACTTTCTGCCGCTAA

TAATTCAAAATTGAATGTTAGTTCCAAAGTTGAAGCCTCGGTTTACAATGATAAAATTGA

ATTTAAAACTTCTTTTGTTCTTACAAATGATATTCACCATGCTATCATTTTAGGAACCCC

TTTTATAAATCTTATTACCCCTTATGCTGTCAACTATGATGGTATATATTTTAAAGCAAA

ATCTAAAGACCTTGTTTTCCCGTTTATTGAGAAACCAAAAACTAGAAACCTAAACATTGT

CAAAGCTTGTTCTGTTTACCAAAAGAAAATTAATGCCATTCTTAAATCCAAACAAGATGA

TTTATTTTGTTTAAAAAAGGATTTAAATTTTCGTAGAATTGAGAGTCAACTCCAAAGTAA

TTTTTTACAAAGAAAAATTTCCGATTTCAAAAGTCTTATTGAAAGAGAAATATGTGCCGA

TTTACCATCTGCTTTTTGGGATAGGAAACAACATTTGGTAGATTTGCCTTATGAAAAGTC

GTTTGATGAAAAACAAATACCCACCAAAGCTCGTCCAATCCAAATGAATATGGAATTGGA

ACAGCATTGTAAAGATGAGATAAATGATCTTATTAACAAAAAGCTCATTGTAAAATCCAG

ATCACCTTGGTCTTGCGCCGCTTTTTATGTTAATAAAAATTCTGAAATTGAAAGAGGCGT

ACCTAGACTTGTAATAAATTACAAACCCTTGAATAAAGCTTTAAAATGGATTAGGTACCC

AATACCTAACAAAAAGGATTTGCTCCAAAAACTCTGTTCAGCTTTCATTTTTTCAAAATT

TGACATGAAGTCTGGTTTTTGGCAGATTCAAATTCATCCAAAAGATCGTTATAAAACTGC

TTTTACTGTTCCATTTGGACAATACGAATGGACTGTTATGCCCTTTGGTTTAAAGAATGC

ACCCTCGGAATTTCAAAGAATCATGAATGACATTTATAACCCCTACTCCGAGTTTTGTAT

AGTTTATATTGATGATGTGTTGATTTTTTCTCAAAGTATTGATCAACACTTCAAACATTT

AAAGACTTTTCATCTTGTTACAAAAAAGGCTGGCCTGGCTCTTTCAAGTACAAAGATTTC

ATTATTTCAAACAAAAGTCAGGTTCCTTGGTCACCATATTTCCAAAGGCACTATTACCCC

TATTGAGAGATCACTTTTGTTTGCGGATAAATTCCCAGACAAAATTCTTGACAAAACCCA

ATTACAAAGATTTCTTGGTAGTTTAAATTATGTTCTTGATTTCTGCCCTAATATCAATAG

GATGTCTAAACCTTTGCATGATAGGTTAAAAAAGAATCCTGTTGCATGGACAGAAGAACA

TACCAAAGTGGTTAGACTAATAAAGCAATCTGTAAAAAACATTCCTTGTTTATTTCTTGC

AAATCCTGCATTACCTAAAATTGTTGAAACCGATGCATCTGACATAGGTTATGGAGGAAT

TTTGAAACAAAGGGATAATGATAAAGAACAGATAGTTCAATATGTTTCTGCTCATTGGAA

TGACTGCCAGAAAAACTATTCTACTATCAAAAAAGAAATCCTTTCCATTGTTTTATGCAT

TACAAAATTTCAAAGTGATTTATTAAATCAAAAATTTTTACTTAGAATTGACTGCAAAGC

TGCAAAACATGTTTTAGAAAAAGATGTTCAAAACATTGCATCAAAACAAATTTTTGCACG

TTGGCAAGCTATTTTAAGTGTTTTTGATTTTGATATTGAATTTATTAAAGGTGACAAAAA

TTCTGTTCCTGATTTTCTAACTCGGGAATTTCTTCAAAACAGATAATGCCGCCAAAGAAG

AATAAAGGTAAAGCAGTTCTCAAAGACACTGGTTCCCAAGAACCTTCCAAAGAACCCCAG

TCAACCCCTTCTAAAGAAAAATTACTTTCTTCTGCCATGCCCATTAAATCTTGGATTGAA

ATGGTTGAAGAACATGGAGCCCAATACAAATCTATTTCTTCTGATGACCAAGTAAAGCAA

TGGATGAGTTCCATTACAAAATCACCTGAGCTCATGCTCGCCCTCCAAAACCTTTCTCAA

AGCCAAATTTCTCCCCAAAAAGAAATTGAAATTACAAAACCCTCTTCCCAAAATGTTGTT

GTCTCTGCTGAAAGCTCATCCTCCCAAATTGTGCTTTCTCAGCCAACACCTTCAAAGAAA

ACCTCCGATTGGTTTGATAAAACCCATTTTCAAAACATTCTTTCTCTAGAAGATGGATTT

TACCATTCTGATCCTTTTCAAAGCATTTCAAAGTTTTTTCCCAAAGGCTGGTTTTTCAAA

CCATGGGATTTGACAAAACCCCAGTCTTATTATCAAAGCATTTTGGAAATAACTGAATCA

GTTAAATTCAAACATTTCTTTCTTGACAAAGCCCATCCAGAACCGGCTTATTCAACGGCC

ACAATCCTCAAAGTATTGAGCCCAAATCAGTGGGGTGACCTACTCCACAATTGTAAAACC

TTCCCTCTAAATTTTGAAACCCGTTTACCACATTGTCGGACTTTTTCCTACTGGGATTAC

CAACAAGCATGGTATAACACCTTCTTTATCCAAAACCCTAAAAAATCCCACTCTTGGTTA

TTTTTCTTCAACACAAAGATAACCGTTCAAAGCCTTCCAAATTGGTTTCAGCATTGGTGG

AATCATTTTGGCCCAATTCCACAAATCTTATCACCAAACGCTACAAATTGCCTAAACCTC

TTCAAAGCCCATTATTTACCCTCTGTCTCAGAAAAAAGATTTCCCCCCTTTCTTTGCTTT

TGTGCAAATTTCTTTCTTCCTTGGGTTTGTTCGTGGAACCTACGTTTCCACAACCAAGAT

TCCCAAGTCATCCTTCAAAGAACCTTTAAAGTCAAATGGTGGTCAAAATTTGATGAACAG

TCCAAATTGACTGTCAACATGATCAAAGATTGGCTCTCCTCTAAAAACCTCCTACAACCA

ACCCTGGAAGCCTCTAAAGCCCAACAGACCTTCCTAACCCAAAAATCCAAAGCCCAGTCT

CTTTTGGCAAGCGCCAAAACGGAAGATGAGTATTTCAAAGCCATGGAACAGCTCCTCGCC

TCTCGATCAAAGTCCTCAGCCGCAGATTCCTCTGAAGATGAAGATGAAGATGTTGATGAT

GATGAAGAGCCTTTCATTTCTCTCGGAGACGATAATGAAGATGACTGTTTCGGCATTTTC

TCTCCGATAAAGCATTGTAAATAATTATTTGATTATTTACTTAAAAAAAAAAAAAAAAAA

AGAAAAAGAAAAAGAAAAAAGAGATGTATTTTCGGGTGGTCCCCTGGACACAAGTGAGAG

CGCACATGTCTCTTCACCCGTATTGTATTTTTCTACAAAGAGTCTACTATTCAAAGCTAC

GATCCAATTGTTACTGTGCACTTAAAATCCCGCTACAGTGAACAGTGCAACTTTCCAGAG

TTTACTGTGCACTTAAAATCCCGCTACAGTGAACAGTTTAAAGATTTTGTATATAAACCG

AGAGGAAGACTTAGACTCCTCAGGTTTTTCATTCCTCT

>CclemV_scCs1 [endogenous-virus-name=Citrus clementina virus] [strain=sequence cluster Cs1] [host=Citrus sinensis] [moltype=genomic DNA] [note=complete genome, mutations in ORF1] 7595 bp

TGGTATCAGAGCCAAGGGGAGGATAGTGTAATATAGTGTAGAGATTTTGTGTTTAAAAAC

TTGTGTTTTATTTTGAAGTTTTGTCGTGTGGTGAATCCTTTACTGGTAGTAAGTCCCGCT

TTAGGATAAGGATTTCCGCTTAGGGCTGTAAAACCATACCTGTAGCCATTCTGACAAGAC

CATAAATAGACCATAGGGTTTTACCCATGGACCCTCTACTCTGTAGAACATCGTCCTTTT

CTAGCTCTTCTTCCGGGAAGACTAGTGATTCAAAGCATGTTGTGAGTTCAGAAGAATTCA

TTATTGAGAACTTTGATAAAGCAATTGATTGTTGGGAACTTCCAAAAGTTTCTAAAGAGA

AAATTTACAAAACAAAAAAGCTTGATTTTTTAAAAAATGATTATGTTATAAAAACTGAAG

AAAGAGACATAACTCTTTCAGAGCCTTTTGAAACAATTCATTTGTTTTCAGAAAAATCTT

TAAAGAAATTGAAAGAAAAGAATTTTAATTACATTCATATAGGTTTAATCCAAATTGGAA

TAAAACCTTTGACCAAAGAAGGCCTGGATACTTCTATCCTCGCTGTCCTAAGAGATAGGC

GATTCATCTCTTTTGACGATTCATTGCTAAGTAGCATCGAATCCAGTCTATGCAAAGGCC

CAATTTCTTTTGATTGCTATCCAAACATAACAATTTCCCTTAAAAGACAAAAACATTCTA

AAAAGCATGATTTTACAAATTAAAACACATAATTATAAAATGATAAAGGGATCTATCCCA

GTTGCTTTAATCTTCAAAATTTCATATAAAGCCATGATCTATGCGTTTAGTACGCAACAT

AAATTCCAGTAAAAAAGAGATGAGACTCTTCTCTTACAGACTGATCTGTCCAAAGCCAAC

ACTATCATCCCAAAACCAATCCAATGGAAAGACGTCAATCTTCCAGAAGAATGGATTCTT

GAAGGAGCCGCTCCACCAGCAATTCCAAAACAACTTGAACCAAATACAGAGTTGCAAAAT

GTGACTCAGTATTCTGATGGAAAAGTCAAACTATCTTTCAGAAGATCTAATTCAACCAGA

TTTTCTAATAAAGAATCGTGCCCAAATACTCCTTCATTAGAAAGGAAATTTACAAAAATC

CCCTCAGTTATAAACCTTTCTTTTCAAAGTCAACCTAGGTTTTCAACCTCAGACATACAC

AGTACCTCTATACGCTCAGTTGACTACACCACAAAAGTTCCACACCCAATCTACACTAGC

AGTCAACATGAACAAAGACAGGAAGAGAAGGAACCTTCTCCCCCAACTTCCCCTACATTC

TCTGCTATCACAGAAAACATTATAAACGTTATCAAAAAAAATTTTGAACTAGACAAAAAT

CTCCTTCACAATAATTTTTATTCAAAAAAAAACAAAGAAAAAAAAATTTGGTTCTTCAAA

CACTTTTTGAACCAAAGAAAAGAAATCCAACAAACTTACTATGAATTTGTGAATCTGCAT

CAAGTTCATATACTGTTTTTTGATTGGTTCGAAATATATTCTTCTGACAACAACATCGAT

TACCCCTTCAAAGAGTCAAACCATATTACTATTAGAAAGAAGACTCCTGAGTGGAAACTT

TCTGATAATGATAGAACCATAGAATCTGAGCATCCACCTCTCCGGAGTATAACCGTTGAT

CACGGCGAACCTCCTGTCCAAATTAGAGCCTCACCGTATAAAATCCCAAAACCAAATGAT

TCTGATACGAATTTAAGTAGTATTATCCAACAGAATAATTTCTGTAATACTAACTTAAAT

ACCATCGGAAAACAATTGACCAGGATAGAAAACCAATTCCAAAAGTCAACCATAGCTACT

TCCTCCGTTTCTCATGTTTAATCAAAATCGGATTCTAACAAAAAGCTTAAAGAACCTATT

TTCAAACATTTTCAGGTTTCAAAAACTAGTCAAAAACTTGTCCAAGAGTCAAAATCAGAT

TTTGCTAAAGCCATTAGAGAACAACTAGATAGGATAGAAGCTACTTCTTCCTCATCTAGC

AAAGTTCAAATAGCCCCTGATACTCCTCAATCTAGCAAGATTGGAGTATTAGAACATGAC

CAAATGTCTATAGCCTCTTCAGATATAGAAGCCTTCAAAGAAGAACATATTCCTAAAGCA

AACAAAATCAATTAGGAACTAGCCCTTCCCACTGTCAAGCCTCCACCTGATTTGACCATA

GACAACAGGCCTAGTGCATTAAATCAGACTCGATAGAATGCATCCTCTGTCTATGAGTGG

AATATAGATGGAATGTCTGAATACAACATCCTAGGAGTGTTGCAACAAATGACCATGGCA

GCCAATGCCTATAAAACCCAATCAAGAACTTCTGACAAAACCATTGCAGAAATCCTTATT

CCAGGTTTTACTGGTCAACTTAAAGGTTGGTGGGATCATCTTCTCACCAAACAGCAACAA

TTAGATATCCTAAATTCTATCCAAGTTGATGAGAATGGTGCCCCCATTCTTGATGAATTT

AATAGTCCAATTCAGGATGCTGTTGCTACCCTGATTTTGACTATATCCCTTCATTTCATA

GGTGACCCTTCTCACCTTAGAGATAAGAACGCTGAGTTACTACATAATTTGAGATGTAGA

AAACTTAGTGAATTCCAAAGCTATAAAACCACTTTCTTCACCAGACTCTTCCTTAGGGAT

GATGCAAATCATATAACTTGGAAGGAGAAATTCCTCGCAGGTTTACCAACCCTTCTAGGA

GAAAATGTTAGGAATTCCATTAAAGCCATTTATGATAACCGCATTCCTTATGATGAGCTC

ACCTACGGTGAACTTGTCAGTTTTGTTAACAAGGAAGGATTAAAGATCTGTCAAGATTTG

AAATTACAGAAACGGCTTAAATGGGAGCTTAGGAAATCTAAGCAAGAATTAGGCAGTTTC

TGTAAACAATTCAATTATGACCCCTTTAAAACTTCTACATTCAAAGATTGTAATGGTAAG

TGTTCTTCAAAACCTTACAAGAAACATTACAAATCCAAAAGCCATAGAAAACCCTTCCAA

GATTTTAGAGAACTTTCTTATAAGAAACCTTCGAGGCCTTATAAGAAACCAAATTTCTCT

AAAAAGAAAGACTTTAAAACCAAACCAAAAACCCCTTTTAACTACAAAGAAGCCATATGT

CATAAATGTGGTATGAAAGGTCATACTGCAAAATACTGCAGGATGAACAGAAAGCTCCAT

GAGCTTGGCCTTGATGAAGAAACCCTTTCTAAAGTTACCTCTCTCCTAATAGAGTCTTCT

GACTCTGAGTCTTCCATGTCGGGAGATAGTGACCCTCTTCAGGTCGATGAGTTAATTGAC

TCAGATACCTCTGCATCTAGCAGTAGTGATTCTGAAACAGATTCTTATTTAAAGAAAATC

AATGTTTTGACTAAAGACCAAGAAATTTTTCTTAAACTTGTTAAACATATTTCTAATCCA

AATCTTCAAAAAGAATACCTTGATAAACTTTTAAAAACTCTAGATTCTAATAAAGCAGAA

ACTTCTAAAGTTCCAATTGTTAAAAAGAATTCTTATGATCTCATTGAAATTTTGGATAAA

AAGAAAACAAAGAAAACAATCCCCAATATCCAAGATCTTCAAAAAGAAATCAAAGAAATC

AAACTTGAGATTAAAGAATTAAAAGAAAAACAAAAGAATGATTCTGAAACTATTCAACTT

CTTATACAAAAACAATTAGAAGATAATTCAGACAAAGAAGTTGAATCCGATGGTGATAAT

GAACAAAATGTTGAAAACATTGAATCTGTACCCAATGATTTTCTGTTTGTTTTAAAACAA

ATCACCACCAGAAAATACTTAATTAAAATCACTTTAGTTTTTTCCAAAGATTTTGCAATT

GACGCCATTGCCCTTTTTGACACTGGTGCCGATTTAAATTGCATAAGAGAAGACATTGTT

CCCAAAAGATTTCATGAAAAAACAAAAGAAAGACTTTCTGCCGCCAATAATTCAAAACTT

AAAGTCAATTCTAAAGTTGAAGCCTCAATTCATAACAATGGTTTTGAATTTAGAACTTCT

TTTCTTCTTACAAATGACATACACCATACTGTCATTTTAGGAACCCCTTTTATAAATCTT

ATTACCCCATATACTGTCAACTATGATAGTATATCTTTTAAAGCAAAAAATAAAAAACTT

GTTTTCCCTTTTATTGAGAAACCAAAAACAAGAAATTTAAATATTGTTAAAGCTTGTTCT

GTTTATCAGAATCAAATCAATGCCATTCTTAAGTCTAAACAAAATGACTTGTTTTGTTTA

AAAAAGGATTTAAGTTTACAAAGAATTGAAAGCCAATTACAAAATGATTTTATACAAAGA

AAAATCTCTGATTTCAAAAACCTCATTGAAAAGGAAATATGTGCAGATCTACCTTCTGCT

TTTTGGAATAGAAAACAACATTTGGTAGATTTGCCTTATGAAAATTCTTTTGATGAAAAA

CAAATACCCACTAAAGCCCGTCCAATCCAAATGAACATGGAATTGGAACAACATTGTAAA

AATGAGATAAAAGATTTGGAGACTAAAGGACTCATTGTAAAATCTAGATCTCCATGGTCT

TGTGCCGCTTTTTATGTAAATAAAAATTCTGAGATTGAAAGAGGAACACCAAGACTGGTA

ATAAATTACAAACCCTTAAATAAAGCATTAAAATGGATTAGGTATCCTATACCTAATAAA

AAAGATTTGCTCCAGAAACTGTATTCTGCTTTTATTTTTTCAAAATTTGACATGAAATCT

GGTTTTTGGCAAATTCAAATTCACCCTAAAGACCGTTACAAAACTGCTTTTACTGTTCCT

TTCGGACAGTATGAATGGACTGTTATGCCTTTTGGTTTGAAAAATGCACCCTCAGAATTT

CAAAGAATCATGAATGACATTTACAACCCTTATTCTGAGTTTTGCATTGTTTATATTGAT

GACGTGTTGATTTTTTCTCAAACTATTGATCAACATTTCAAGCATTTAAAGACTTTTTAC

TTTGCCACTAGAAAGGCTGGATTAGCCATTTCTAGTTCTAAAGTCTCTTTGTTCCAAACA

AAAATCAGGTTCCTTGGTCATTATATTTCCAAAGGAACTATTACTCCTATTGAGCGATCT

CTTGTGTTTGCAGATAAATTCCCAGATAAAATTCTTGATAAAACTCAATTACAAAGATTT

CTAGGAAGTTTAAATTATGTTCTTGATTTCTGCCCTAATATCAATAGGATGTCCAAACCT

TTGCATGATAGGTTAAAAAAGAATCATGTTGCATGGACTGATGAACATACCAAAGTTGTT

AAACTAATAAAGAATTCTGTTAAAAATATTCCATGTTTATTTCTTGCAAATCCTGCATTA

CCTAAAATCGTTGAAACTGATGCATCTGATTTAGGTTATGGAGGTATATTAAAACAAAAA

GAAAATGATAAAGAACAGATAGTACAATATGTTTCTGCACATTGGAATGAATGCCAGAAA

AATTATTCTACTATCAAAAAAGAAATTCTTTCCATTGTTTTATGCATTTCTAAATTCCAA

AGTGATTTACTAAATCAAAAATTTTTACTTAGAATTGATTGCAAAGCTGCAAAACATGTT

TTAGAAAAAGATGTTCAAAACATTGCATCAAAACAGATTTTTGCACGATGGCAAGCCATT

TTAAGTGTTTTTGATTTTGATATTGAATTTATTAAAGGTGATACAAATTCTGTTCCTGAT

TTTCTAACCAGAGAATTTCTTCAAAACAGATAATGCCGCCAAAAAAAAAAGACAAAGGTA

AAGCTGTTCTCAAAGACACTGAGTCTCAAAAAACCTCTAAAGAACCCCAATCAACCCCTT

CTAAAGAAAAATTACTTTCTTCTGCCATGCCCATCAAATCCTGGATTGAAATGGTTGAAG

AACAAGAAGCCCAGTATAAAAACATTACCTCTGATGACCAAGTAAAACAATGGATGAGTT

CCATTACAAAATCCCCTGAGCTTATGCTCGCTTTACAAAGCTTTTCTCAAAGCCAAATTT

CTCCTAAAGAAGAAATTGAAAAAGAAAAACCTATTTCCAAAGAGATTTCAAAACCCTCTT

CTCAAAATATAGTTGTTTCTGGTGAAAGTTCATCTTCTCAAATTGTTCTTTCCCAACCAA

CACTTTCAAAGAAATCTTCCGATTGGTTTGATAAAACCCATTTCCAAAACATTTTAACTA

TGGAAGATGGATTTTACCACACTGATCCTTTTCAAGCAATTTCAAAGTTTTTCCCCAAAG

GCTGGTTTTTTAAACCATGGGATTTGACAAAACCCCAGTCTTATTATCAAAGCATTTTAG

AAGCCACTGAGTCAGTAAAATTCAAACATTTCTTTCTCAGTGAAACCCATTCTGAGCCAG

CCTATTCTACGGCTACTATCCTAAAAGTCTTGAGCCCAAACCAATGGGGTGATCAACTTC

ACAAACATAAAACCTTCCCTCCAAACTTTCAAATGCGTTTACCACATTGTTTGACTTATT

CCTACTGGGATTATCAACAAGCCTGGTATAACACATTTTTTATCCAAAACCCAAAAAAAT

CCCACTCTTGGTTATTTTTCTTCAATTCAAAAATAACCGTCCAAAGCCTCCCAAACTGGT

TTCAACAATGGTGGAACTATTTTGGCCCAATACCTCAAATTCTAACTCCTAATGCCACCC

ATTGTTTAAACCTCTTTAAAGCCCATTATATTCCTTCTGAATCCGAGAAAAGGTTTCCCC

CATTTCTATGTTTCTGTACAAATTTCTTCCTTCCATGGGTATGGATGTGGAACCTCAGAT

TTCATACCCAAGAAGCCCAACTTATCCTACAAAGAACCTTCAAAGTAAAATGGTGGTCAA

AGTTTGATGAACAGACTAAATTGACTGACACCCTAATCCGAAATTGGCTCTGTTCCAAAG

GCTTCCTACAACCAACCATAACAGGCTCCAAAGCCCAACAAACCTTCCTTACCCAAAAAT

CCAAAGCCCAATCTCTTTTGGCAAGCGCCAAAACTGAAGAAGAATATTTCAAAGTTATGG

AACAACTCCTCACCTCAAGATCAAAGTCATCAGTGGCAAACTCCTCTGAAGATGAAGACC

AAGATGAAGAAGAACCGTTCATTTCTCTTGGGGATGAAAATGAAGACGACTGCTTCGGAA

TTTTCTCTCCAATAAAACATTGTAAATAATTGCCATGTAAAAAAAAAAAGTATTCTAATA

TTTTCATTTTTTATATTCTGTACCCTTGGGTGGTCCCCTGGATACAAGTGAGAGCGCACA

TGTCTCTTCACCCGAATTAAAATTAGAAAAGCTTGTGCATTCCAAAGACTACTGTATACT

CAAGCTGCACTACAGTCTACAGTGAGCCTGTGCATTCCAAAGACTACTGTATACTCAAGC

TCCGCTACAGTCTATAGTGAAGATTTCCAAAGATTACTGTATGCTACAGCCCCGCTACAG

TCTACAGTTTAAAATTTCTTTGTACAAAAACTCAGAGGAAGACTTAGACTCCTCAGGTTT

TTCATTTGAGTTTTTAAAAGTTCTCTCTTCTCTCT

>CclemV_scPt1 [endogenous-virus-name=Citrus clementina virus] [strain=sequence cluster Pt1] [host=Poncirus trifoliata cv. Pomeroy] [moltype=genomic DNA] [note=incomplete at 3’ end] 7708 bp

TGGTATCAGAGCCAAGTTAGGGAAAAGTGTTTTATTGTTTAGAGTCTTCGTTTTTAAAGT

CTTGTGTTTTATTTTGAAGTTTTGTTTTTGTGGTGAATTTTTACTGGTCGTAAGTCCCGT

TTTAGGATAAGAATTCCGCTGAGGGCTGTAATCCCATACCTGTAGCCATTCTGACCAAAC

CAAAAATAAGCCATAAGGCTTTGACTATGGATTCTCTCCTCCATAGAACATCATCCTTTT

CTAGCTCTTCTTCCGGGAAGACTAGTGATTTAAAGCATGTTGTGAGTTCAGAAGAATTCG

TTATTGAGAACTTTGATAAAGCAATTGATTGTTGGGAACTTCCAAAGATTTCAAAAGAAA

AAATTTACAAAACAAAAATGCTTGACGTTTTAAAAAATGATTATATTATAAAGACTGAAG

AACGTGACATAACTCTTTCAGAGCCTTTTGAAACAATTCATTTGTTTTCAGAAAAGTCTT

TAAAGAAATTAATAGAAAAGAATTTCAAGTATATACACATTGGTCTCATCCAAGTAGGAA

TAAAGCCTTTAACGAAAGAAGGTCTAGATACCTCTATACTGGTCGTCCTAAGAGATGGGC

GATTCATCTCTTTTGATGATTCTTTGCTAAGTAGCATCGAATCTAGTCTGTGTAAAGGTC

CAATTTCTTTTGACTGTTATCCAAATATAACAATTTCTCTTAAAGACAAAAATGTTTTAA

AAAGCATGATTTTGCAGATCAAAACCCATAATTACAAAATGATTAAAGGGTCAATTCCAG

TAGCCTTAATTTTTAAAATTTCATATAAGGCTATGGTTTCTGCATTTAGTACACAACATA

GATTCCAGTCAAAGAGGGATGAAACACTTCTCTTGCAGACTGACCTGTCTAGAGCAAACA

CTGTCATTCCAAAGCCAATCCAATGGAAAGATATCAATCTTCCAGAGGAATGGATCTTAG

AGGGAGCTGCTCCCCCAACGATCCCAAAACAACTTGAGCCAAATACAGAGTTGCAAAATG

TGACTCAGTATTCCGATGGTAAAGTTAAACTATCATTCAGAAGGTCTATGTCATCCAGAT

TTTCTGATAAAGAGTCGTGCTCAAGTATCCCTACTTTAGAAAGGAAATTTACAAAAATCC

CCTCTGTCATAAATCTCCCATACCAATCTACAAAGAGTCAACCGAGGTTTTCCACCTCAG

ATATACCTAGTTCTTCTATACATTCTGTTGACTATACTACAAATGTTCCACACCCTATCT

ACACTAGTAGTCAACATGTACAAAGTCAGGAAGAGAAGGAACCTTCTCTTCCAACATCTC

CTACATTTTCTGCTGTCACAGAAAATGTCATTAATGTCATAGAGAAAGAATTTGAATTAG

ATAAAACATTACTGCATAATGATTTTTATTCTGATTTAAACAAAGAAAAAAAAATTTGGT

TTTTCAAACAATTTTTAAATCAAAGAAAAGAAATCCAACAAATTTATTATGAATTTGTGA

ATTTTCATAAAGTTCATATATTGTTTTTTGATTGGTTCAAAATATATTCTTCTGAGAACA

ACATAACCTATCCTTTCAAAGAGTCAAACCCCATTACTATTAGAAAAAAGATCCCTGAGT

GGAAACTTTCTGATAGTGATAAAACCATCGAATCTGAGCATCCACCTCTTCGGAGTCTAA

CCATTGATCATGGCGAACCTCCTATCCAAATTAGAGCCTCACCTTACAAAATCCCAAAAC

CAAATGATTCTGATTCAAATTTAAGTAGTATTATCCAACAGAATAATTTCTGTAATACTA

ACTTAAATACAATTGGAAAGCAGTTGACTAGGATAGAAAACCAATTCCAGAAGTCAACCA

TCACTGTTTCATCCACTTCTCCTATTCCATCAAAATCGGATTATGACAAAAAGCTTAAGG

AACCTATTTTCAAACATTTCCAGGTTTCGAAAACTAGCCAAAAGCTTGTTCAAGAGTCAA

AATCAGATTTTGCTAAAGCCATTAGAGAACAATTAGATAGGATTGAATCTGCTTCGTCCT

CTTCTAATAAAGTTCAAATAGCTCCTGATACTCCTCAATCTAGCAAGATTGGAGTATTAG

AACAAGATGACCAAACTTCTATAGCCTCTTCTGACTTAGAAGCCTTCAAAGATGAAGCAC

CTACTCCTAGAGCCAACAAAATCCATTGGGAACTTGCCCCTCCTACAGTCAAATCCCCTC

CTGATTTGGCTATAGACAACAGGCCTAGTGCATTAAATCAATCTCGATATAATGCATCCT

CTGTCTATGAATGGAATATTGATGGCATGTCCGAATACAATATCTTAGGAGTCTTGCAAC

AAATGACCATGGCAGCTAATGCCTATAAAACTCAATCCGGAACCTCTGACATGGCTATTG

CAGAGATTCTTATCGCCGGTTTTACTGGTCAATTAAAAGGTTGGTGGGATCATCTTCTCA

CTAGGCAGCAACAAATGGATATTCTTAATGCTATCCAAATTGATGAAAATAGGATACCTA

TTCTCGATCAATTCAACAATCCTATCCAGGATGCTGTTGCTACCTTAATCCTTACCATTT

CCCTCCATTTTATAGGTGACCCTTCTCACCTCAGAGACAAAAATGCTGAGTTACTACATA

ATTTAAGATGTAGGAAACTTAGTGATTTCCAATTATACAAAACCACCTTCTTCACCAGAC

TCTTTCTTAGAGATGATGCGAATCATACTACTTGGAAAGAGAAATTCTTAGCAGGTCTAC

CTACCCTTTTAGGTGAAAAGGTTAGAAATTCCATCAAAGCCCTTTATGACAATCGAATTC

CTTATGATGAGCTCACCTATGGTGAACTTGTCAGTTTCGTTAATAAGGAAGGTTTAAAGA

TTTGTCAAGATTTGAAGTTACAGAAACGACTAAAACAAGAGCTTAGGCAGTCTAAGCGAG

AACTTGGTAGTTTCTGTAAACAATTCAATTATGATCCTTTTAAAGCTTCCACTTCCAAAG

ATTGTAATGGTAAGTGCTCTTCGAGACCTTACAAAAAACATTACAAGTCAAAAAGCCATA

GGAAACCCTTTCTTGGACATAGGGAAAATTTTTATAAGAAACCTACTAGGCCTTATAAAA

AGTCCAGATTCCCTAGAAAGAAAGATTTTAAAGCCACACCAAAAACTCCTTTCAATTTCA

AGGAAGCCATTTGTCATCGATGTGGTATAAAAGGCCATACTGCAAAATATTGCAAAATGA

ACAGAAAGCTTCATGAACTTGGTCTTGATGACGACATCCTTTCCAAAATTGCCCCCCTTA

TGATTGAGTCTTCGAATTCTGAATCCTCTATGTCAGGGGATAGTGATTCTTTACAAATTG

ATGAGTTAATTGATTCAGATACTTCTGTATCTAACAGTAGTGATTCTGAATCAGAGTCTT

ATTTAAAGAAAATTAATGTTTTGACTAAAGACCAAGAGACTTTTCTTGAACTTGTAAAGC

ATATTTCTGATCCAAATCTCCAAAAAGAATATCTTGAAAAGCTTTTAAAAACAATGGATT

TCAACAAAGCTGAGACTTCCAAAGTTCCAATCGTAAAAAAGAATTCTTATGATCTTACTG

AAATTTTAGATAAAAAGAAAACCAAAAAATCGGTCCCTAATATCCAAGACCTCCAAAAAG

AGATTAAGGATATTAAGTCCGAAATTAAAGATTTGAAAGAAAAACAAAAAAGTGATTCTG

AAACCATCCAACTTCTTTTACAAAAACATTTACAGGATGATTCAGATAACGAGTCTACTC

ATAGTGAAAATCATATTGAGCAAAATGTTGATAACATTGAGTCTGTCCCACATGATTTTC

TCTTCGTTTTAAAGCAAGTTACCACTCGAAAATATTTGATTAAAGCCACCTTAATCTTTT

CAAATGATTTTGCAATTGATGCCATTGCCCTTTTTGATACTGGTGCAGATTTAAATTGCA

TAAGACAAGATATTGTTCCAAAACGATTTCATGAAAAGACAAAAGAAAGGCTTTCTGCCG

CCAACAATTCAAAATTAAAAGTTGATTCTAAAGTCGAAGCCTCTATTCATAACAATGGTT

TCGAATTTAAAACCTCTTTTATTCTTACAAATGATATTCATCATGCTGTAATTTTAGGAA

CTCCTTTTATAAATCTTATAACTCCGTATACTGTTAATTATGACAGTATATCTTTCAAAG

TAAAAAATAAAAAGATTATTTTTCCTTTTATCGAAAAACCAAAGACAAGAAATTTGAATA

TTGTTAAAGCCTGTTCCATTTACCAAAACCGAATTAATAATCTAATTAATTCGAAACAAA

ATGATTTAACCTTTCTCCAAAAAGATTTGAGTTTACAAAGAATTGAAAATGATTTACAAA

GAGATTTCATTAAAAGAAAAATTTCTGATTTCAAAACCCTAATTGAAAAAGAAATTTGTG

CTGATCTGCCTTCTGCTTTTTGGAATAGAAAACAACATATGGTAGATTTGCCTTATGAAA

ATTCTTTTGATGAAAAACAGATACCCACTAAAGCTCGACCAATTCAAATGAATATGGATT

TAGAACGACATTGTAAAGAAGAAATTAATGATCTTGTAAAAAAAGGACTTATTGTAAAAT

CAAGGTCACCTTGGTCATGTGCCGCTTTTTATGTTAATAAAAATTCTGAAATTGAGAGAG

GCACACCAAGACTTGTCATAAATTACAAGCCTTTAAATAAAGCTTTAAAATGGATTAGGT

ACCCGATACCTAATAAAAAGGATTTGTTACAAAAACTGCATTCTGCTTTCATATTTTCAA

AATTTGACATGAAATCAGGATTTTGGCAAATCCAAATCCATCCAAAAGAACGTTATAAAA

CCGCTTTTACTGTTCCATTTGGACAGTATGAGTGGACTGTCATGCCTTTTGGTTTAAAAA

ATGCACCTTCAGAATTTCAAAGAATTATGAATGACATTTATAATCCTTATTCTGATTTTT

GTATTGTTTATATTGATGACGTGTTGATTTTTTCAAATTCAATTGATCAACATTTCAAAC

ATTTAAAGACCTTTTATTTTGCCACCAGAAAGGCTGGATTGGCAATTTCAAATTCCAAAG

TCTCCTTATTTCAAACAAAAATCCGGTTCTTAGGTCATCATATTTCAAAAGGAACCATTA

CTCCAATTGAGCGATCACTCGCTTTTGCTGATAAATTTCCTGACAAAATTTTGGATAAAA

CCCAACTACAAAGATTTTTAGGCAGCTTAAATTATGTTCTTGATTTCTGTCCAAATATTA

GTAGGTTATCTAAACCTTTGCATGATAGGCTAAAAAAGAAACCTGCTGCATGGACTGATG

AGCATACAAAGACTGTTAGATTAATAAAGAATTCTGTTAAGAGCATTCCATGTTTATATC

TTGCAAATCCTGCATTGCCTAAGATAGTTGAAACTGATGCATCTGATTTAGGTTATGGAG

GTATTTTAAAGCAAAAGGAAAATGATAAAGAACAAATTATTCAATATGTTTCTGCACATT

GGAATGATTGTCAAAAGAACTATTCTACTATTAAAAAAGAAATTCTTTCCATTGTTTTGT

GCATATCAAAATTCCAAAGTGATTTATTAAATCAAAAATTTTTACTTAGAATAGATTGCA

AAGCTGCAAAACATGTTTTAGAAAAAGATGTTCAAAACATTGCATCAAAACAAATTTTTG

CACGATGGCAAGCCATTTTAAGTGTTTTTGATTTTGATATTGAATTTATTAAAGGCGATA

AAAATTCTGTTCCAGATTTTCTAACTCGAGAATTTCTTCAAAACAGATAATGCCGCCAAA

GCGTCGAGACAAAGGAAAAGGCATAGCCAAAGACACTGATTCCCTAAAGCCCTCCAAAGA

ATCCCAATCAACTCCTTCTAAAGAAAAATTACTATCCTCAGCCATGCCAATCAAATCCTG

GATTGAAATGGTCGAAGATGAAGAACAAAAAGCCCTTTCTAAATCCATCTCCTCTGACCA

ACAAGTCAAAGAATGGATGGAATCCATTACAAAGTCCCCTGAGCTTATGCTTGCCTTACA

AGGCATTTCAAAATCTAAAACCCTTTCTCAGATCCCTGAGGAAGAAAAACCCATTTCTAA

AGAAATTACAAAACTATCTTCCCAAAGCCAAAATGTTGTCATTTCTGGTGAAAGCTCATC

TTCTCAGATTGTTCTTTCCCAGCCAACACCTTCAAAGAAAACCTCCGATTGGTTTGATAA

ATCCCATTTTCAAAATGTTTTAACTATGGAACATGGGTTTTACCATTCTGATCCTTTTCA

AGCAATTTCAAAGTTTTTTCCTCAAAGCTGGTTTTTCAAACCATGGGATTTAACAAAACC

CCAGTCATATTATCAAAGCATCCTTGAAGCCACTGAGTATGTAAAATTCAAACACTTCTT

TCTCAGTGAAACCCATTCTGAGCCGGCCTACTCCACGGCCACGATTTTAAAAGTTTTGAG

TCCAAATCAGTGGGGCGACCAACTCCATAAATACAAATCTTTCCCTCCAAATTTTCAAAT

GCGTTTACCACACTGTTTGGTGTATTCCTATTGGGATTACCAGCAGGCTTGGTTCAATAC

CTTTTTTATACAAAACCCTAAAAGGTCTCATTCATGGTTATTTTTCTTTAATTCTAAAAT

AACCGTACAAAGCCTTCCAAACTGGTTCCAACAATGGTGGAATTATTTTGGTTCAACACC

ACAAATCTTAACTACCAATGCCACCCATTGTCTAAACCTTTTTAAAGCCCATTATACCCC

ATCTGACTCAGAGAAAAGATTTTCTCCATTTCTCTGTTTCTGTACAAATTTCTTCCTCCC

ATGGGTATGGATGTGGAACTTCCGGTATCATACCCAAGAAAAACAACTACTTATCCAAAG

AACCTTCAAAGTCAAATGGTGGTCAAAATTTGATGAACAAACCAAGCTCACCGAAACCCT

TGTCCAAAATTGGCTTGGTTCAAAAGGTTTCCTACCGCCAACCATTAAAGAATCAAAAGC

CCAACAAATTTTCCTTACCCAAAAATCAAAAGCCCAATCTCTCTTGGCCAGTGCCAAAAC

TGAAGCAGAATACTTCAAAGTCATGCAACAGCTTCTCGCTACACGATCAGAGACATCAGT

TGCAAGTTCCTCCTCCAGCACCTCGGCAGATGAAGAACCCTTTATCTCTCTGGGTGATGA

GAATGAAGATGACTGCTTCGGCATTTTCTCTCCAATAAAGCATTAAAGCTATATTACGAT

AGAATCTTGGTTTTACCTACTTCTATGTATGTACAGTTTTGTACTTAAAAAAAAAATGAT

GACAAAAACATTTCTTTCGGGTGGTCCACTGGACACAAGTGAGAGCGCACATGTCTCTTC

ACCCGTATTTCTGTATGTATTTTGTACTTTATATTTTGTAACTTCTTGTACAAAGCCTAC

TATTCAAAGATACCATCCAAAGATTACTGTGCACTTGAAGCTCCGCTACAGTGAACAGTG

CCAAAGACTACTGTGCACTTACAGTTCGTTTGTATAAAAACCGGGAGGAAGACTTAGACT

CCTCAGGTTTTCATTTTCCAATTTCAGAGCTTCTCTCTCTTCTTCTCCTCCTTCTCTCTA

CATCTCTCTCTCTAGGAATCTGAAATTCAAAGACACTAAGGAAATTTTCCTCCGGGTTAG

TTCCTTGTCTGCGAATTAATTTCCTTTC

>CsatAV [endogenous-virus-name=Cucumis sativus A virus] [host=Cucumis sativus cv. 9930] [moltype=genomic DNA] [note=incomplete at 5’ and 3’ ends, mutations in ORFs 1 and 2] 6497 bp

ATAGAAAAAAGTCTCCATAATTATACCATACCAAAATTAGATGTTGAAACTATATATGAA

ATAGGAAAATTTGATTTTGTTCAAGATTATTGAATCAAAATTGCAGAACATTTAGTTCCA

ATAAATAAACTTTCTAAAACTCTTTCTTTATTTTCTAAACAATCAATTCTTCAACATCAA

GAAAAATACAAGTTTCTTCACATGGGCTTAGTTCAAGTTGCTATAAAACCTCTATACAGG

ATTGTGTTAGATACTCCTATGCTTTTAATTTTACGAGATAAAAGACATAGAAACTTTCCA

ACCTCTGTACTTTCAATAGTTGAATCAAATCTTGAAAAAGAACCAGTGTCGTACATTAAC

TGTTATCCAAATATTACTTGTCTCTTTAAGTGATCCAAATATAATTCGTTCTTTAATGCT

TGATATTAAAATCCTAAATATGGATTTTAGGAACCACAAACCAAATCCATCGTAGGAATT

TTTCGAATACATTATAAATTAATGCACACAAATTTATCTCCTAAAGCACTTAGAACTTCT

CCTAAAGATCAAACTCTTCTTATGGAAGCCAATACAGAATATTCAAATACCTTCACTCCA

AAAATGTTATCTTGGAATCAAATTACTCATAATCCTAGATGGAATCTAGAAGATGCTTTT

AAACCATCAATTGAAAATTCAAAATTAACTCAAATTGTAGAAAATAAGGATGGAAGCGTA

GAAATACAATTTCAATCTCAATCCGCAAGATTAGAACTTCCTAAATTTTCTACCTATGAA

CCCGCTAGTTCAAGTAATTCTTTAAACCTTCAAGGAATACAATTTGATAATACAATACCA

CAACCCTTTTATACAAAAGAAAAAGACCCAACCTTATCTCCAACGCAGTTCCGATATGCA

AAATATTAATCAATTAAATGTTAGCTCTAAACCCTTTTCAATAAATAAAATAAATCTAGA

TAATGAATTTTGGTCAAATAATAATAAACTTTTAAGAAATAAATTTTTAAAAATCCTTTA

ACAAAATTGAACAAAATGAAATACGTGAAGAATTAATAATACAAAACTATAGAAATGGAA

AAATTAAAAACAAATATATACTTCTTCCAATGGATAAAGCATAAGACCTTTTATCAAAAT

CTTACATTTCTAATACATCAAACTATTAATACAAATTCAACTTTACCCACTTCGAAAAAC

TACAAATTCAAAAAGACAAATAACTTCTTCACATCCCTCTTCAGAAGAAATCAAGTTCCA

AAGTAGCAAATTAGATCAAGAAGTTATAGCTTCTCCTTTCAAAATGATAGATTCCCAAGA

AATTGAAACAAAACCACCAACTTTAAAAGATATAAAAAACATACAATTACAAAATAATTA

TTCCAATAAAATTTTACATACAATATCCAACCAAGTAGAAAGAATAGAATCTAAACTTGA

ACCTCATCATTACTCTATTAAATCACAACCTCAACCTATAGATAAAACAAAACCTTTATT

CAAACCTTTTGAGATTTCTGATAAATTTAAAATTAATTAATTTCTCAAGTAAAGAATCTA

ATAGCATTTTTGGACAAAATTAATCTCCAATTACAACACTTAAATCTAAATACTTCTAAA

AATCCATCCACGTCAAAAATCAATACTATAAATGAAACCCCTATTGAAGATCATAATTTC

GAAGATAACAATCTTGAAGAACAATTCCAAAATCTTACTCTTCAAAAAATAACAAATCAT

AGATATCCAAAACTAAAAAATTATTATTCTCGACCTTCCTTTCCTGATATGCAATACAGA

AAATGGCATCTATGAATGGAACATAGATGGAATGACCGCACATCAAATTTTAAATTTAAT

GCATGAAATGACTTTAGCAACCACTGCCTATAAAATACACCAAAATTCAGACCATCAAAT

TGTCCAGATTCTCATATCGGGATTTACCGAAAATCTAAAAGAATGGTGGGATAATTGTGT

ACCCACAAGTGACAAAGCACGAACTCTGACCACTGTTAAAGTTGAACAAAGTGGTACGGG

CATCAATCAAACCCAAGATGTTATAGCAACCTTAATCTACACTATTATGAAAAACTTTAT

AGGAGAACTCGTTTAATTCCAAGACGAAGTTCTGAGAGCCTTTCAAATCCTACCTGTCAA

AAACTTCAAGATTTTCAATGGTACAAAGATGCTTTCCCAAGCAAAGTTTTTACCAGAACA

GATTGCAATAATCAATACTGGAAAGAAAAATTCCTTTCAGGATTACCAAAATTATTCACG

GAAAAAGTACGCCAAACTATTAGACAAACCTTTCAAGGAACAATCTCATTCGATAATTTA

ACTTACGATGAACTAATTAACTTTATAAATAATGAAGGATTAACTTTAAACAAGGATCTC

AAATTAAAAGCAAAAATAAAAAATGAATTCCACAAACAACGACAAGAACTTGGATCCTTT

TATGCCCAATACGGTTATGAAAAAATACAAGCTCCTTCAACACTAAAACAACCCAAGAAA

TATAAGAGAAACTTCTTACGAAGAAAACCTTACAGGAATCAAAGTTATTATCAAAACAAT

CAAAATAAACAAAATTATAAATCTAGACCAAAAAAGAAATATAAAAACAACAGACCCACT

AAAAAACAAAAAGAAATTATCTATTACAAATGTAGACAAAAAGGACATATAACCCCTAAC

CATCCAATAAAAGGAAAAATTAATAATTTGGAAATAGAAGAAGATTTGAAAATCCAATTA

CTAAATTTACAAAACCCTTCAGATGCTTCATCATCTTCCGATCAAAGTGAAGGATCTTTT

TGTGACCAATTTTTTTAAATTCAAGGAAATTCTTATAATACCGACTCTGAAAAGTCTTTT

TATTCTAGTGACAATCAAATAGAAATTTGTACATGCAATAAGGGCATAAATGACATTTCA

AGAAATCAAGAGATTCTTTTTTAGGCCACTAGTCAAATTGAGGATTTAGAAACCAAAAAG

AAATACTTTTAGAAACTGAAGAACCTAATTATACAAGAACCTAAACAAGAAAATATTCAA

TACTACAACCTTAATGATATATTTTCTCGGTTTGAACTCAAAACAAAACTAGTTACAATT

TCAGACCTTCAAAAAGAAATAAATACAATAAGAGAAGAAATTACTAACATCAAAAAAAAG

AAAATTCTCAAATTAGATTTGAACTTCTCTCCCTCCAAACCCAACAAATAATTGATCAAC

CTTCCACTTCTAAAGATCACCCTGGAAATATTCTCCATGATTCCTTTATTAATTTCGTCA

ACAAGGTTCAATTACAAAAATAGTATTCAGTCATTAATCTTGTTATTGACAAGGAATATC

AAGTGACTTCAAAAGTTTTGATTGATTTGGGAGCCGATCAAAATTGCATACGAGAAGGAC

TCATACCTACCAAATATTTTGAAAAAACATCAAAAAAAAAAAAAATTACATGGTGCTCAT

GGTCAAAGGTTAAAAATTCGCTATAAATTATCAAATGCACACATTTGTAACCAAGGTTAT

TGCTTTAAAAATACATTTATTTTTGTAAAAGATTTAGACAAATAAATAATTCTAGGAACC

CCTTTTCTTTTCCAACTATACCCTTTCTCAATAATGAAAAATAAATAGTCTCAAAATTCT

TAGATCAAGAAATTATTTTTAATTTCGTAAACCCTATATCTACTAAAACAATACATTCTA

TTCAAAATTCTTCGATCGCTATGAAGCTATTAATTCTATCCACAGAAAAACACAACAAAT

ACAATTTTTAAAGGAAGAAATAAGCCATAAAAGAATTGAACAACAATTAAAAAATTCGTA

TATTCAAAATCAAATTAATCAGTTTCAAGAATTAATTGAAAAGGATATTTGTGCATCTAT

TCTCAATGCCTTTTGTGAACAAAAACAACATATTGTTAACTTACCTTACATAAAAACTTT

CATGAATAAAAAATTCCTACTAAAGCTAGACCCATACAAATGAATGTTGAGTTATTAGAA

TATTGTCATAAAGAAATCCAAAAACTTCTAGAAAAGAGACTTACAAGACCAAGTAAATCA

CCTTGGAGTTGCGCCGCTTTTTATGTCAATAATCAAGCAGAAATTGAACGAGGAGTACCT

CGCCTTGTAATCAACTATAAACCTCCAAATTATGTTTTAGAATAGATTCGTTATCCTATA

CCCAACAAAAAGGATTTAATAAATCGAATACAAAATGTCTTACTTTTCTCAAAATTTGAT

ATAAAATCAACATTTAATGTTCCATTCGAACAATATAAATGGAATGTAATGCCATTTGGT

CTAAAAAAATGCCCCCTCTGAATTCCAAAATATAATGAATGATATTTTTAATCCATACCA

AGAATTTACAATTGTTTACATTGATGATGTCTTAATATTTTCTCAATCCATAGAACAACA

CTTTAAACATTTAAGAATTTTTCACAAGATAATTAAACAAAATGATTTGGCCGTCACTCT

ACCCAAAATAAAATTATTTCAACAAAAAATTAGATTCTTAGGCTACGACATCTTCCAAGG

AAAAATAAAACAAATTCAAAGATCAATCAAATTTGCTAACAAATTCCCCAATGAAATCAC

CAATAAAAATCAATTGCAAAGATTTCTAGGATGTTTAAATTACATTACAGATTTTATTCC

AAATCTAAGACAAACTTGTGAACCACTATATCAACGACCTAGAAAAACACCTCCTCCATG

GACTCATGAGCACACACGCATTACTAAAACCATAAAAAACCTTGTCAAAAGCCTTCCTTG

TTTACCCATTATCAATCCACATGCACCACTTATTGTAGAAACAGATGCATCAAATATAGG

AAATGGTGGCATTCTCAAACAATCCATCAATAGTCAAGAGCAATTAATTAGAGATCATTC

GCGTATCTAACTAGAAGCTCAAAAAAATTATTCAACTGTTAAAAAAAGAAAGGTTATCAA

TTGTTTTATGCACAAACAAATGTCAAAGCGATTTGATAAACAAAAGCTTTTTAATACGTG

TTGATTGCAAAGCAGCAACAAATGTTTTACAAAAGGATGTTAAAAACTTAGTTTCTAGAC

AAATTTTTGCCAATGGCAAGCAATACTTTCTTGTTTTGATTTCAAAATTGAGGCTATAAA

AGGTTGTACTAATTCTCTTCCTGATTATCTCACTAGAGAATTCCTACAAAGAAAAAGATA

GAAAGATAGTGAGAAGAAAAATAAAGAACCATAAAAGGAGAAAGAAAAGAAAAGGATGAG

AATGAGAAAAAACAACAAGCAACTCTCCCTCAAACTAAATCTTCTTACTCCTAAGCATAT

CAATTTACACAAGCGGAATCCTCACAATCAATGGAGGATCAAAAAGGGAAAAAACCTCAA

CAATTACCATTATCCAACAAGTTTCAAAATTTGGGATCTTCCATCCCTTTAAAGCCTTCT

ACCTTAATTATCAACCCTTCTCCGATAAAGCTTTTGGGATCTTCCATCCCTTCAAGACCT

TGTACGATAACAATTAATCCCTCTACAATCAAAACAGATAAGTCATCTACTCCTTCCACT

ATGTATGCTCAAGCTATCACTAAACCAAATTCCCAAAACTCTGAAATTATTCCATCAAGA

ACAATATATTACCATACTTCATCTTCCCACAATAAACCCCAAACTTCTTTTATCACAATC

CTAGAACCTGAATATTTGGAAAATTTCATATTAGAAACAATCCAAAAAACATTTCCATCA

AATTTTCACTTCATTCCAAACCATCCTCAAAAAACTCATCTATTCTATGAATATATTACA

ATGGAAACCGATTCAATTAAAATAACCCACACCAGAAATAAAGAAGACCCTACCAAAATA

GCTTTTTCAAAAATCAAAATCTTGGGAGTCCTAACTCCAACCAAATGGAATCAAAGTATT

TACACTGAAAAAAATTCTTAACATCAATTCCTTCCTCAAAACTATTCATATATCGATTAC

CAAAAAGCCTGATACAATGTATTTTGGCTAAATAATTTTAATCATTTTTGGTTCATTATG

TTTGACAAAACTTGTATTCAAAAATTTTCATTATGGTTTAATGATTGGTGGGATATTTTT

GGCCTAAATGAGGCTATTCTACCATATCCAATTTTAGAAAGTTTCAAGTTATTCTCCAAC

AATATTTTTCCAAATGCTCTTGATCAAATCCTACGATCTTCAAATTATTTTCATATCGCC

TGAATTTTCTATTGGAATTACTCAATCACAGAAACCAACGACACCTCCCACCTTTTCCGC

ACACATCGAATTAAATGGTAGAAAAAATTCAACACAACAATCGCCTAAAAAAATATAGTG

AAAAAAAAGGTTTCCATATATCCTCAGAATATGAAGCCAAATCTACAAAAAAAACTCATC

AATTTTTAGCTCATAAAACAAAGTTAATAGCTGTCATGACTCAAGTTATAAATGAAGACA

AACTCAATATTATTATT

>CsatBV [endogenous-virus-name=Cucumis sativus B virus] [host=Cucumis sativus cv. 9930] [moltype=genomic DNA] [note=incomplete at 5’ and 3’ ends, mutations in ORFs 1 and 2] 6043 bp

CTTTGAAGTGTAGATCGAAGCCCAGATCCAACGACGCCTCCATTCTGTTTTTGTTCAGAT

CCGCTAAGCCTCTCTACTCCATTCAGTTCAGTTTCCGTTCTGTTCGCCCCTGATTTCCCG

TTTAATTTGATTCTGACTTGCTTTAGTTTAGTTCGTTCAGTTCCATTCTGATGACGAAAC

GCTCCTGCCCAACTTATGATGGTCAATCACTCATCACCTGGAATATCGACGGATATTCAG

AAACACAAATGATGAATACTTTTCAAGAAATGTTATAGGCAGCCTATGCATTTAGCGCCA

GAAAATCAATCTACGAAACGGCTCAAATACTCATATTAGGCTTCAATGGAAACCTAAGAA

GATGGTGGCATAATCAGCTAACTGAACAAGATCGACAAAGGATCCTGACTACAACAAAAA

CGATTGTAAAAACTAAAAACTCTTCAGTTCCAGTACAAACCGAAGAACCAGATATGATAA

ATCAATTACTCTACACAATGACTAAACATTTCATTGGAAGTACCCAGATTCATTTCAATC

TAGCAACAGAAGCTCTTCTTGGACTAAAATGTCATAAGATGAGCAGATACAAATGGTATA

AAGACACCTTCATGGCACGAATGTATACCATAACAACATGCGACACTAATATCTAAAAAC

AAAAATTCGTTGAAGGATTACCATATTATATTTCTCAAAAGTTCTATCAAACTATGACAG

CAAATTCTGTCAACCAACAAATTAATTGGGCAAATCTAACTTACGGGGATATATCATCCA

CAGTTCAAATGATATGTGTTAATCTCTGTACAGAGAATAAACACACAACGAAGGTGAATA

AAGATTTCCGACTACCGGAAGGAATTGGGCACCTTCTACAAACAATATGGTCTGTCCAAC

AGACCAAAGGAGGAAAAGGAAAAGAAGAAAAAGAAGAATTATCCTTCTCGAAACATTATC

TTCAGAAAAAAAAAATCTAAAAATCAAGAGCCTCCTCGAAGAAGGAAGCACAATTACAAG

AAGGGTAAAAGCAAGAAGCATTACTCTTCAAAAACAAAAACAATTTGCTACAAATGCAAT

CAAAGTGGTCATTATGCTAATCACTGTCCTCTAAGAGACAGAATCAATGCCCTCAAAATA

GACGAAGAAACGAAGCAATCCCTCCTCTATGCTATAAGGTCGGATGATGACAACTCCTCT

CAAACAGAATCTTCATCAAAAGAAGATATCATCAACGTTTTACAAGAAGAAAGATCGTCC

TCTAAAGAAGAATTCTTCTCTCAAAGTGATTCAAGTGATGACGAAGGGGCTATTCCCTGC

ACCAGACAATGTGCTGGACACATCAATGTCATCACTAAAGATCAAGAAACTCTTTTCGAT

CTCATCGAACAAATACCAGATGAAACAGCAAAAAAAACCTGTCTACTCAAATTGAGGCAA

AGTATAGAAGAGCAAACGCCTCGACAGACGGTTCATAGTCCGATAATGTACTCATATCAG

AACATATTGAACCGAATAAAAGGGCAAAACAAAAATGCCCATTCAAGTTAATGATCTTCA

CCATGAAGTAAAGATTCTCAAAAAAGAAGTTGCTAATAACAAGCAACGACTCACCTATCT

TGAAAATGCTTTCCAAGCATTCCAAGAATCTCCAATCAGGGAAGAATATCCGGAAACTTC

AACAAACAATTTTGAAAGAAAAACCAAGGAAATGACACTATTGATAGAAGAACCAAACTT

TATCAATAGTATAAGCAAAGTTCACAATCAGAAGTGGATATCAAAGATAGTCTTTAAAGT

TAAAGACTTTCAACTAGAAACATTAACCCTCTTCGACTCCGGGGCTGATCAGAATGTTAT

TCAAGAAGGCCTCGTCCATTCGAAATATTTCGAAAAGACGAAAGAATCCTTAACTGGAGC

AGGAGGAAATCCTCTCAATATCCAGTTCAAATTATCCAAAGCGCACATTTGCAAAGACAA

TGTGTGCCTGGTTAATACTTTCATTCCGGTCAAAAACCTTAATGAAGGTATCATTCTAGG

AACACCGTTCCTAACTCAAATATATCATTTTCATGTCACTAAAGAGGGTATAATGTCGAA

GAAATTCGACAAAGAAATTACTTTCGAGTTTACTCAACCAGTAACTCCCAAGTACATCTC

AAATATTGAGGAAGAAATTCGTCAATTCATCAATAGAATCGCCAAAAAGGAGAAACAAAT

AGAGTTTCTTCAAGACGATATCAAAGGTTGTAAAGTGACTTCAGAAATAAGTAAGCCATT

AATCCAACAAAAAATCCAGAACTTCCAGCAACGACTTGAAAAGGAAGTCTGCTCAAATCT

CCCAAATGCCTTTTGGGATAGAAAAAAACATATGGTAACTCTACCATATAAAGAAGGATT

CAAGGAATCACAAATTCCCACAAAAGCCCGACCTATTCAAATGAACAAAGATCTAGTCAA

AGTCTGTACAAACGAGATAAAGGATCTACTCAAGAAGGGCCTTATAAGCCCATCAAAAAG

CCCATGGTCGTGTTCGACCTTTTATGTCAATAACCAGGCCGAAAAAGAACGTGGTGTACC

AAGGCTTGTCATTAATTACAAGCCCCTCAACAAAGTTCTCAAATGGATTAGATATCCAAT

TCCTAATCGGCAAGACTTGCTAAAAAGAATTACTCTAGCAAAAGTCTTCTCAAAATTTGA

TATGAAATCTGGATTTTGGCAAATCCAGATACACCCAACTCAACATTACAAGACGACTTT

CAATGTTCCATTCGGAAAATTCCAATGGAATGTCATGCCATTCGGATTGAAGAATGCTCC

GTCCGAATTTCAGAAGATAATGAACGATATCTTCAACCCGTATCAAGATTTCACAATAGT

ATACATTGATGATATCTTGGTATTCTCAAACACTGTTGATCAACATTTTAAACATCTTCG

GGTGTTTCTAAATGTGATTAAAACAAATGGTCTTGTGGTTTCCCAACCAAAGATCAAATT

ATTTCAAGTAAAGATTAGATTTCTTGGTTATGAAATTAATCAAGGAATCATCAAACCAAT

TCAAAGGTCTCTGGATTTTGCAGATAAATTTCCAGATGTAATACAAGACAAAACTCAGCT

ACAGAGGTTTCTTGGCTGTGTTAATTACATCGGAGATTTTATCCGCGATCTTCGCTCAAT

TTGTCTACCATTATATGACAGACTGAAAAGGAATCCAAAGCCATGGACGGACGAGCATAC

TCGAGCAGTCCAATCAATCAAGTCCCTGGCCAAAAGCATCCCATGCTTATCTCTTGTAGA

TGAACAGGCCAAACTCATTATTGATACAGATGCATCCGATATTGGTTACGGAGGCATCCT

CAAATAGGAACTTAACGGAAAAATCTCCATTGTCCGTTATCATTCAGGAATCTGGAATAG

TGCACAAAAGAACTATTCAACAGTAAAAAAGGAAATACTAGCAATAGTACTTTCCGTCCA

GAAATTTCAAGGAGATCTGATTAACAAGGAATTCCTTGTTAGAACAGATTCAAAAGCAAG

TAAATACATCTTTGAAAAAGATGTAAAGAATCTTATCTCAAAGCAAATCTTTGCAAGATG

GCAGGCAATCCTATCTTGCTTCGATTTCAAAATTGAGCCTATAAAAGGCAGTGAGAATTC

GCTTGCAGATTACCTCTCAAGAGAACATCTCTTGAAGAACCAAAAATCAGCCTTGAAGTC

TCTCTCAAATGGAACCTCCTTCCGGCCGGCAACGGCCACCCAACCAGCGGCAACCGCCGC

TAAATAATCCAAACAAAAATCCTCAAAGACCACCTCAAACAGTAGCTTCCTCTTCAGGAA

CTACTTCTCCTAGGGGAAAATCCACGGCTCAATCATCTACACCGTCACCGATGAGTGCAG

AAAACTATGCCATGGATTTACAATTTGAGCAAGTATCCAGGCGTCGTCAAGGCTCTGCTA

ACAGAGCATTGACAATCCAAACAGACACTTCGAGTCTTCCCCCTCATACCGTCAAGCACG

TTGTTGCGACCCTCTGGGCCGACAACTCCAAACAAGTGTGTCGCATCACCGGCCGCTTCC

TCCTCAAGGTCTGCAATTCCGCGGAATCCATCCTCGTATTCCCAGATTGTACGACCAAAA

GTCTTCCAGCCAAGACCACCAATTACTGGTTATTTCACCAAAACAACAATGGTGGATTTG

ACCATTGAACCAGAATTCGACGGACCTTCTGTCCTCGAAGTCTGCAAACAAATATTTCCT

CATGGATTCAATTTTCTTCCAGAGGATCTAGCAAAAACTAGGACGTTTTATGAGTTCATT

TTAGTTGATTCTAAATCGGCTGAAATCACACATGTTCCTAACAAAAATGATCCCTCGAAG

ATCATTTACTCAAAGCTGAGAATTTTTCGAGTACTCACTCCATCCTACTGGAAACAGGGG

ATGTTCGTGGGAAAGAGATTTTCTCAACCCTTCAAACCACCATCATACAATTACCGCGAT

TACACAAAAGCATGGTATATCGTATTTTGGCTTCAAGCACATAACCATTCATGGTTTGTG

ACATTTTGCAAGCAAGCTTACAAAATGCACTTTCCCCAATGGTTTCAAAACTGGTGGATG

TATTTTGGACTCTCTGAGGAAATTTTCCCAGTAGAAGTTCAACGTTCATACCATCTCTTC

CAACAAAGTATCTACTCGTCTCCTCTCTCAAAGACGTTTAGGTTTGCTTTGTATTTTCAA

ATACCCTGGATCTTTTGCTGGAATTTCCAACTCGGACCCTCAGGGAATTTCAAAGCATTG

AGCAAATCACTCAGGATCAAGTGGTGGGAAAAATTCAGCTATTCCCATCTAGAAGTAAGC

AAAATAAAGGACTGGTTTAAAGCCAACGTCCATCTGCAAGATATGACTAGGCAAGAGGAT

GAAGCCTTCCTCCTCACCAAAAATGCAGTCATGACAACTCTTGCTGGCACAAGTACTCAA

CAGGAATTCAATTCCATCGTCAACAACATCGTCGTAGACCTCTCAGACGACCACTCATTT

ACTTACCCTAAAGTTGGGAAGGAGTGATTATGTTTTCAGCTCTCCACTCGATTTTGTCCC

CAAAATGTAAGCATATTGAGTTGACAATCTTGCCACTCTCACTCGTACAAATCAAAGGAC

AATCCTTCGCGAACATGAGTTCATAATACACTCAGGATTAAGACTAAGTTACCTAAGTCA

TCCTAATGAAATAGAATTCCAACTAGTTAACGGAGTTACATCTAATGGTTACTATTTCGT

GGTCCAGTCTTATACTAACTCATTGTATAGTACCCCTTACTCGCATGTCAACTGCACGAA

CACGTTAGATCATTGCGTTTGTATCAAGTACAAAGTGAACCGTATCTATAGTGTTACCAG

GATAAGGTATCCGAACTTATCCCTATACTACAAGCTCTTTAAGCTGATCTCGGCATTGAT

TCATGTATATTGGATTTAGGTTATTAAGAAAAAACTAATAATCAATAACACTTATTGAAA

TTATAATAACAACACTTTATTAATAACGATCAATGAATTATATTTACAATCTACGAGTTT

TAGGACATAAATCCCAACGAGAAGTATTTATAATACTTTTTCGCTGTACATTTTTGGAAA

ATAGAAAACACAACAAATAATAACTAATTCTAAAACAGAGTTTATACATGATAATATCCT

CCCTCAAATTGATGCACTTTAGACTTAAGGCATAAATTTGGATCAAAGAGTGTTAAAGCG

ATCGGTGGTGAGAGGTTTTGTGAATATATAAGCAGGTTGTTTTGTTGAGGGAATGTACAA

TAGTGTAATGTAATTTTGGACAACTTTTTCCCTTATAAAGTGAAAGTCTACTTCCATGTG

TTTGGCTCGACCATGAAAGACAGGGTTATGAGCAAGTTGGATGGCAAACTCTTTGTTACA

CCATAAAAAAGGAGGTTCTTTTCTAAATATATGTAAGTCTCGAAGGAGTTGTCAAATCTA

AAAAAACTTAAGAAGTTGTTGTTGCAAGGGCTCTATACTAAGC

>EgranV_sc1 [endogenous-virus-name=Eucalyptus grandis virus] [strain=sequence cluster 1] [host= Eucalyptus grandis accession BRASUZ1] [moltype=genomic DNA] [note=complete genome] 7803 bp

TGGTATCAGAGCCAGTAGGAAGGTATAGATAGTCTTTGTAAAAACATACTCTTAGATCGA

GAGCCATTCGTTGGCATCCTTCTTGTTTCTACAGAGTGGTCCAGGCCGTAAGTCCCTGAG

TGGCTGTTAGACCGTTGGTTTAGCTCTGTAACGGTTCTGAAGAAGGAATAGTTCCGATCT

TAAGTATGAGTTTACATAGAAGATCTACATCTTCCTCTAGTTCTCTATCACAAATGGACT

CAGATCCAAGTCCTATACATAGAGAAGAAGTAAGGATAGAAGATTTCAACAAATCTATAG

AAAATTGGGAAATTCCTAAAATACAGCTTAAGGAAATATACAAGCAATCAAAATTGAAGT

TTTTCAAAAAATCAGACTATGTCATAACTACTGAAGAAAGAGATGTTCCTCTTTCTAATT

CTTATGAAAAAATACATCTTTTGAACAAAGAGTCCATCCAAAAGCATAAACAAAAATACA

ATTACATTCATATAGGACTAGTCCAAGTAGGAGTCAAACCTCTTACTAAAGAAGGCCTTA

ATACTTCCATTCTCTTAGTTTTACGAGATTCACGTCTTTTAAACTATGATGAATCCATTC

TCGGTACTGTAGAGACAAGTCTCTGCAAAGGACCCATACATTTTGACTGTTATCCTAATT

TCTCTGTATCCCTCAAAGATAAGAACATTCTTAGAGCCCTAACACTCCAAATCAAAACCC

ATAATTACAAAGTGGCAGATGGATCTATTCCTTTAGCCCTTGTCTATAGAATTCATTATA

AAGCCATGTCCTCCGCCTTTGGAGAAAATGCTTTCAAACATAGTCCACGAGGAGAAACAC

TTCTCCTCCAAACTGACATTTCTAGAGCAAATACAACAATTCCTAGGTCTATTAATTGGA

ACCAAGTTACTTTACCAGAACAATGGGAATTAGAAAACCAAATTCCCCAACAAGTTGTTC

AAAATACAGATCCGACTAATGTCATTCAACATCCAGAAGGAAGAGTGACCATTAGATTTC

CTAGAAGATCTTTCGATTCAAGATCTAGTCCTTCTTTTCATAATTCTTATACAAGGTCCA

TAGATCTTAATCCTAATCTTCCTCCTATAATCACCATTCCTCCTAGTACGCATAGAAACA

CTCCTCCTCCTAGTATACATAGAGATACTCCCACACACGATACAACACCTTTACCTCCTT

CCACCAGTAGAATTAATCAAGAGACACCTCCTGAACCAGAAATTAGTGGTTTAGATAATA

GATCAGGGATTTCCAGGCCAGTTTATCAACAAAACCAGCCTGAACCAGTCCTAGAAAGTA

CAGATTCCCCTCATCATTCTCCTACTTATTCTCAAATGATAGATGCTGAGCTTAATGTTT

TAGAAAAACATTTTGAACCAAACAAAACTTTTCTTAATAAAGAATTTAATTCTGAAGACA

ACAAACAAATCAGAGAATGGTTCTTCAAAACCTTTGCTGATAAAGCAAAAGACATCAAAC

AAAAATATTACGACTACATATACGAATATGAAGTCCATATATTTTTCTTTGATTGGTTAG

AAGAACAATTCCTTGAACCAAAAGAAATACTTGTTTTAGATCATCATTATAAGTGGAAAC

TTGATAATGGTGAAACCATAGAATCCAATCATCCACCCCTTAGAAGAATAAAAATACAAC

ATGGAGAAAGTGAGGTTACAGCTACTCCTTTTAGGCTTCCAGATCAAGGAGACTCTCTTT

ATACTCACAAAGTCATTGAACAAAACAATTACACTAATCAACATTTGGCGACCATAGGAA

AACAGTTGAATAGGATAGAATCCACCATCCAGAATCCTACTCAACTCATCACACCACAAG

TAGACACTTTCAAAAAACCCATTTTCAAAGCCTTTGAATTCCCAAAAAACCTTAGCCCGG

AGTTTGCCAAAGCCATAGAACAAAAGAAAAAACTCCTAGGAAAATCAAAGCTAGAACCTA

CTCCTATTATAGACACCCCTAAATTCCAGAATCTTATCATTAAAGACACCCCTGACCAAC

CCACTATCAGTACATTGACCAAACAATCTGGTGACTCTGATAATGAATCGGTCGCTGAGA

TTAACAGACTCAATTGGAGACAACCTCAGAAGCTTTATTATAGTAGAGCTACCCCACCTG

ACATTGCCCAAGAAGAATCATCACAAAAAATACATAATAAATACTCTCCTGATGCCATTT

ATGAATGGAATATAGATGGTGTTGCCGAAAGCAATATCATGAATATTCTTAATAACATGG

TAATGCTTGCCAATGCCTACAAAACCCAGCAAGATGTTTCTGATCATGCTGTTGCTAATC

TTTTAGTAGCCGGATTTACTGGACAACTAAAAGGTTGGTGGGATAATTATTTGACTGATG

AACAAAGAATACAAATTTTAACTGCCTATAAAATAGATGATTTTGGCCAACGCATGGATG

ATGATCAAGGACATCTCATCCAAGATGCGGTGAATACTCTTATCTATTCCATTTCACAAC

ACTTCGTAGGAGACCCTTCACATATTAGAGATAGGAACCAAGATCTCTTATCCAATATGC

GTTGCAAATCCTTAGGAAGATTTAGAGAATACAAAGAACTTTTCCTTCAAAGGGTTCTTA

TCAGACCTGATTGCAATCAACCCTTTTGGAAAGAAAAATTCTTAGCAGGTTTACCCCAAA

TTATAGGTGACCAAGTTAGAGATAAAATTAGAGAAGAATACAATGGCCAAATACCTTATG

ATCTTCTTTCTTATGGAGAAATAATCTCTTATGTTTACAGACAAGGAACACAAATGTGTA

ATACTATCAAATTACAAAGACAGCTCAAGGAAGAACAATCTCTCACCAATAGAAGTCTAG

GAGATTTCTGTGCTCAATACGACCCCTCTTATAATGAAAAAACAAAACCAAAATGTAAGG

GAACTTGTCCTTCTGAGAAAGAAACAAAGAAACATCACAACAAATCTCAACCTCGTAGGA

ATAAACAAAGACACCAGAATAACCAAAATAGACCTCATAATGATAGGAAAATTTTTAAAG

ACATCAAATGCTATTCTTGTGGTAAAAAAGGTCATATTTCAAAATACTGTAGGATTAATA

AAAAATTAAATGAACTCTCTCTTGATGACAATACTCTTAACCAGTTAAATAACATCTTCA

TAGAAAATGGTGATACTTCTTCTGATGAACTTCTTGAGGAAGAAGGTCTTCAGATTAATG

AAATAGATTCCTCTTCTGATTCTGATTATGAAGACGCCTCTCCTGAAAAACCCATTCCTG

AAATAAACATGTTAACAAAAGAAGAAGAATTTCTCCTTAGCACTGCTGATAAGATTACTG

ACCCAGAAGCCAAGAAAGAATACTTAGATAGACTTCATTCTTCCCTAAATATTACCTCCA

AGCCCAATACTTCTTACAATTTAAAAGACATTTTGAATAGAAACAAAAAACCTCAGTCTA

GACCCGTTAATATAAATGACCTCCAAAATGAAATAAAACAACAAAAATTAGAAATTCAAG

CCTTGAAAGCAACACAGTTAGATATGAAAAAAGAAATTTCTGATATAAAATCTCTTGTCA

TAAAAGGAAAAACAAAATTAGAAGACCAAAACGAAATCACCAATACATCTGATACTCCAG

ATCCAGCCTTCCTTATGTTATTAACAGAAATAACATCTAGAAAATGGATAATAAATATTA

TGATAAAAATCAACAATGAGTTTATTATTGAAACAACAGCACTCTTTGATACAGGAGCTG

ACCTCAATTGTATCAAAGAAGGCCTTGTTCCAACAATATACTTTGATAAAACTTCTGAAT

CCTTAAAATCAGCCTCAGGAGACAAACTAAATATACAATACAAATTACCAGAAGCCCTAA

TAATAAAAGACCAAATGTCTTACAAAACATCTTTTCTTTTAGTTAAAAATATGAGGCAAC

AGGTAATTCTTGGAACACCTTTTATACAATTAATCCAACCTTTTACAGTTACTAATGAAG

GTATTGAAACTCATGCTCTAAACAGAAAAATCACTTTCAACTTCATAACAAAACCACAAA

AGAGAAGCCTAAATATTTTACAAGAACATTCTATTTCAGAAATAAATTGCCTTATAAAAG

ACAAAGAAAACCAGCTTGAATTCTTAAAAGAAGATTTAGAATTCAAAAGAATAGAAGAAA

ATCTTATAAAACCAAACATCATAGAAAAAATAGCTTCCCTACAAAAACACATAGAAAAAA

CTATATGCTCTACTCTACCCAATGCCTTTTGGGAAAGAAAAAAGCATATCGTTGATTTAC

CTTATGAACATGATTTTTCTGAAAACAAAATACCTACCAAAGCCCGCCCAACACAAATGA

ATCAAGAAGTTTTAAAATTCTGTCAAGCAGAAATACAAGATCTCTTAAATAAAAAACTCA

TAAGAAAAAGCCAATCACCTTGGAGTTGTTCTGCTTTTTATGTCAATAAAAATGCTGAAC

TAGAAAGAGGAGTTCCCAGGTTAGTTATTAATTATAAACCTCTTAATACAGCCCTTAGAT

GGATAAGATATCCTATACCCAATAAAAAAGATTTGCTAAACAGACTTTGTAGATCAAAAA

TATTTTCAAAATTTGACATGAAATCAGGATTTTGGCAAATACAAATCAGTGAAAAGGATA

AATACAAGACAGCCTTTACAGTCCCTTTTGGCCAATATGAATGGAATGTTATGCCATTCG

GCCTAAAAAATGCCCCTTCAGAATTCCAAAGAATTATGAATGAGATTTTCAATCCTTATT

CAGAATACATCATAGTTTATATAGATGATGTTCTAGTATTCTCTTCCAATATAGAACAAC

ATTTCAAACATTTATCTACTTTCATAAAAATAATCATACAAAATGGTCTTGTAGTCTCTC

CTACTAAAATTGCACTCTTCAAAACCAAGATTAGATTTTTAGGTCATTACATCCATTTAA

GCACCATAACTCCCATAGAAAGATCAATCTCTTTTACTGATAAATTCCCAGATGAGATTA

AAGACAAAACTCAATTACAAAGGTTTTTGGGTAGTCTCAATTATATTTTAGATTTCTTTC

CCAATATAAATATCCTTTGTAAACCTTTACACCAAAGGTTACGAAAGGATCCTCCACCCT

GGACCTCTGTCCATACAGACATTGTCAAACAAATAAAAATGCATGTAAAAGATATACCAT

GCTTACACCTTGCTGACCCTTCCACTTTCAAAATAGTTGAAACAGATGCCTCAGAATTAG

GATATGGTGGCATTCTCAAACAAGTCAATAACAACAAAGAACAAGTTGTTCAGTTTATAT

CAAAACATTGGAATTCCACTCAACAAAATTATTCGACAATCAAAAAAGAAATACTTGCTA

TAGTTTTATCTATATCAAAATTCCAAGGAGATTTATTAAATCAAAAATTTCTCTTGAGAA

TTGATTGCAAATCAGCCAAAGACATTTTACAAAAAGACGTTTTAAACATTGCATCAAAAC

AAATTTTTGCCAGATGGCAAGCCATACTTTCAGTATTCGATTTTGACATTGAATTTATAA

ATGGTTCTTCAAATTCATTACCTGATTTTCTCACAAGAGAATTTTTACAGGGGATACCAA

TATGCCAAGATCCAAAAAAGACAAAGATAAAGGCAAAGGCAAAGCCACTGCCGAACCCTC

CAATACACCACCAAAGCCAAAAGGCTTAACATCTGTCAAGACATGGCTCCAAATGGCAGA

AAACAACCAGACATTCTTACACCAAACACCTTTGAAAATAGAACCCATATCATCTGAACC

CATGCTAGCCTTTAATCAGCTAGCCGACATACTACCAAAAGAAACAATATTGTTACTTGC

CCAGTCTTTACAAAACGTAAAGAAAGAGCCATCCAGTGCCATAGAGGTAAGCAACAATCC

TTCCCCGCTTGCTACACAAAAACAAGCGGAATACAAAAGAATTTCGAATGAGGTGGTTAT

TAGGCCACAGGCAACCCCTCATTCTCAAAGATCAAAATTTTTCCCCAAAACATCTTTTCA

AAAAATATTGACTGTGGAAGATGGATTCTTCGATGAAGATCCAATCAAATTTGCAAAAAA

CATATTTCCAAAAAATTGGCTTTTCAAACCACACAATACAAACAAAACCCTTGCTTATTA

TGAGCAAATTCTAGTGGAAACAGAATCGGCAAGGTTCACTCACTTTGCTGACAAAAACAA

TACAGAAAATATCATATACTCCACTATATCCATCAGTAAAGTCATACACCCCTCACACTG

GGGAGCCTCACCCCATACACCCAGAAAATTCCAAACCAGACTTACCCAAAGCCTACCATA

CTCCTCCTCATATACATACTGGGACTACCAACAAGCTTGGTGGAACGTCTTCTTCAGACA

AAATACAAATTTCCAACACACCTGGTTATTATTCTTCAAAGACAACTTCCCAAGACAAAG

CCTACCAAACTGGTTTGACTCCTGGTGGGATCTCTTTGGTCCTTTGGACATAATACTACA

CCCCACAGTCAAACAAGGATACAAGATTTTCAGTGAATACTTCGTACCAACACCACATGA

TATAAGGTTCCCATGCCTTATGCATTTCTTTATAATATTCAGAATCTCTTGGGTAACAGC

CTGGGAATTTGAATACAATACATCCAATAAACCTGGTGTACCAGTACTTGTCCGAAAATA

CAAAACAAAATGGTGGGATGGATTCAAATTTCATGAACAAGGATCCGAAGCTGCGGTTAA

AACATGGCTAAATAGCCAGAAAACCCAAATAACCACAGCCCCATCAAATACATCGTTTTT

ACAGGAAAAATCCATGGCAAGTGCAGCACTTGCTGCAGTCCAGAACAGAGAGGAATACTG

CAACATCCTCAGGACAATGCTCTCAGAAGCGGAAGAATCACAATGTGGATCTTCCAAATC

TCAAGACAGTCAAGACGGATCCTCACAGTCACATCACAGTGGAAACACTAATGAAGACGA

CTGCTACGGGATCAATCTAGACAGAGATTAAATTCAAGACATCACATGCCAACACTATTA

CAAGCGGCGCAAAGGCTATACACCGTAGCATAATAGACATACACTGTTCACTATTCACTA

TTCACTTTACACTGTTCACTCAGTACTGTTCACTTTACACTGTTCAGCACTGTAGCACTT

TCACTGTAGCACCCTCTATAAATACACCCTCTCGACATAGAAGAAGGGGACTCGAAATTT

TCCAGATTTCTCTCTTCTTCTTCTCTCTCTCTCACTTGTAACCCATCATCCCTTCCATCT

CCATCCTCATCTCTTCCATCTCCAAGCTCTCTTACCTTGTATCCCCTGGTTTCAAAGTAA

GTTAGTTTTCATATACATAGTTTTATACAGGGTTACCAACTTACATGATAGTTGGTTAAC

CATTTCTTGTAATACAAACATCAATACTCCCTGGAGTGCGGATTACCGCCCTAGTGGATG

ACTTGATGCTTTAGTTTCTGCATCTTTCAATACATGTAATTTCAGCTTTTCAGCTTTATA

TAATACAAGTTCAGACCACCTTCATGGTTGGTTTTTGTTTATGCATACTCTTACTTTATA

TTTTATATATTGCCTTTGCTCTTTACATTATTTTTAATTCTCGCGATTTTATTTAAGTCA

TTTACTTTCTTGTTCTTATACATCATTTCCTTTTAGATACATCCTTCTCTGCTTTCATAT

AGATCTGTTAATCTCTTATACTTCCTTGTTCTCTCATATAGTACTCCGATCCTAACCCCT

TTA

>EgranV_sc2 [endogenous-virus-name=Eucalyptus grandis virus] [strain=sequence cluster 2] [host= Eucalyptus grandis accession BRASUZ1] [moltype=genomic DNA] [note=complete genome, mutations in ORF1 and ORF2] 7893 bp

TGGTATGAGAGCCAGTGAGAAGGTACAGATAGTCTTTTTAAGTCCATACTCATAGATCAA

GAGCCATTCGTTGGCATCCTTATTGTCTCTACAGAGTGGTCTAAGCCATAAGTCCCTAAG

AGGCAGTGAAACCATTGGTTTATCTCTGTAATGGTTCTGAAGAAGGAACAGTTCCGATCT

TAAGTATGAGCTTACTTAGAAGATCTACATCTTCCTGTAGTTTTTTTTTTTTTATCACAA

ATGGACTTAGATCTAGGTCATATACACAGAGAAGAAGTAAGGATAGAAGATTTCAACAAA

TCTACAGAAAATTGGGAAATTTCTAAAGTCTAGCTTAAGGAAATATACAAACAATCAAAA

TTGAAATTTTTCAAAAAATCATATTATGTCATGACTACTAAAGAAAGAGATATTCCTCTT

TCTAACTCTTATAAAAAAAAAATACATCTTTTGAATCAAGAGTCTATCAAAAAGCATAAA

GAAAAATACAATTATATTCATATAGGACTAGTCCATGTTGGAATTAAACTGCTTACTAAA

GAAGGTCTTAATACGTCCCTTCTCCTAATTTTACAAGATTCACGTCTCTTAAACTATGAT

GAATCCATTTTCGATGCAATAGAGACAAGTCTCTACAAAGGATCAATACATTATGACTAT

TACCCAAATTTCTCAGTATCCCTTAACGATAAAAATATTCTTAGAGCTCTCACACTCCAA

CTAAAAACCCATAATTACAAAATAGCATATGGATTTATCCCTTTAGCTCTTGCCTATAGG

ATCCATTACAAAGCCATGGCTTCTGCATTTGGAGAAAATGCTTTTAAACATAGTTCACGA

GGAGAGATGCTTCTCCTCTAGATTGACATTTCTAGAGCAAATACAACAATTCCCAGATCT

ATTAGTTGGAATCAAGTCACTCTACCATAACAATGAATTTAGAAAACCAAGTTCCGCAAC

AAGTTGTTCAAAATACAAATCCGACCAATGTTATTCAACATGAGAAGGAAGGGTGACTAT

TAAGTTTCCTAGAAGATCTTTTGATTCAAGATCAAGTCCTTCATTACATACATCCTATTC

AAGGTCAATAGACCTTAACCCTAATCTTCCACCTATAATCACCATTCCTCCAAGTGCTCA

TAGAAATACTTCCATGCGATATACAACACCTTTACCTCATAATACCAATCGAAATAATCC

TGAGACACCACCTAAACCAGAAATTAGTGGTCTAGATAATAGATCAGGGATTTCCAAGCT

AGTTTATCAACAGAATCAGCCTGAACCAGTCTTAGAAAATACAGACTCTCCTCATCATTC

TCCTACATATTCACAAATGATAAATGCAGAACTAAATGTTTTAGAAAAACAATTTAAACC

AAATAGAGCAATCCTTAATAATGAATTTAATTCAAAAGGTAACAAACAAATCAAAGAATG

GTTCTTCAAAACCTTTGCTGATAAAGCAAAATATATCGAGCAAAAATATTACAACTACAT

ATATAAATATGAGGTCCATATATTTTTCTTTGATTGGTTAGAAGAACAATTCCTCAAATC

AAAAGAAATAGTAATTTTATATCATCATTATAAGTGGAAACTTGATAATGGTGAAACCAT

AGAATCCAATCATCCACCCCTTAGGAGAATAAAAATATACCATGGAGAAAGTGAGGTAAC

AGCCACTCCTTTTAGGGTTTCAAACAAAACATATTCCCTTTACACTTATAAGGTTATTGA

GCAAAACAATTATACTAATCAACATTTGGTAACCATAGGAAAACGGTTAAATATGATAGA

ATCTACCATACAAAATCCTACCCAACTAATCACACCACAAGTAGAAACACTCAAAAACCC

CCATTTTTAAAGCTTTTGAATTCCCAAAAAATCTTAGCCCGGAGTTTGCCAAAGCCATAG

AACAAAAGAAAAGACTTTTAGGGAAATCAAAACTAGAACTTGCTCCTATACTAGACACCC

CTAAATTTCAAGGGTTAACTATTAGGGATACCCCAGATCAACCCACTATTAATACTATAA

CAAAACCCTCTAGTGATTCTGGTAATGAATCAATCGTTGAGGTAAACAAACTCAAGTGGA

GACAACCCCAAAAACTTCATTATAGCAGAGCCACCCTACCAGACATCACTCAAAAAGAAT

CATCGCAAAAAATACAAAATAAATACTCTCTAGATGCCATTTACAAATGGAATATAGATG

GTGTAGACGAAAGCAATATTATGGACAAATTAAACAACATGATAATGCTTGCTAATGCCA

ATAAAACACGGCAAGATGTTTCAAACCATACTGTTGCTAATCTTTTAGTAGTAGGATTCA

CTGGACAACTAAAAGGTTGGTGGGATAACTATTTAACTGATGAACAAAGAATACAAATTC

TAACTGCTTATAAAATAGACAACTTTGGACAACGAATGGATGATGAACAAGGACATATCA

TCCAAGACGTGGTGAATACACTCATTTACTCTATTTCCCAACACTTCATAGGAGATCCCT

CATATATTAGAAATAGGAACCAAGACCTCCTATCCAACATGACATGCAAATCCTTAAGGA

GATTTTGAGAATGCAAAGAACTTTTCCTTCAAAGGGTTCTTGTTAAACCAGATTGCAACC

AACCTTTTTGGAAAGAAAATTCTTAGATTTTTTACCCCAAATAATAGGTGACAAAGTTAG

AGATAGAATTAAAGAAGAATACAGTGGTCAAATACCTTATGATCTACTCTCATATAGAGA

AATAGTCTCCTACGTTTACAAGCAAAGAATACAAATGTGTAATACAATCAAATTACAAAG

ACAACTCAAGGAAGAACAATCTCTCACCAACAGAAGTTTGCGAGATTTTTGTGCTCAATA

CGAGCCCTCTTATAATGAAAAAAAAAAATCAAAATGTAAAGGAACTTGCCCTTCTGAAAA

AGGAACAAGGAAACATCACAACAAATCACAACCTCATAAAAATAAATGAAGACACCAAAA

TAGATCTCACACTAATAAGAAAATTTTTAAAGACATTAAATGTTACTCTTGTGGTAGAAA

ATGACATATATCAAAATATTGTAAGATTAATAAAAGATTGAATGAACTATCTTTAGATGA

TAATACTTTATACAAGTTAAACAACATTCTTGTAGAAATTGATGATACTTCTTTTGATGA

ACTTCTTGAGGAAGAAGGTCTTCAAATAAATGAAATAGAAACCTCTTCAGAATTAGAATA

TGAAGACGCCTCTCTTGAAAAATCTATCCCTGAAGTAAATATGTTAACAAAAGAAGAAGA

ATTCCTTCTCGGTACTGCTAATAAAATAACCGACCCAGAAGCCAAAATAGAATACTTAGA

TAGACTACAATCTTCTATGAATGTTACTTCTAGAACCAATACTTCTTACAATCTAAAAGG

CATTCTAAATAGAGAAAAAAAAAAAAAAAAACCCAGTCGAGGCCAATAAATATTAATGAT

CTCCAAAATAAAATAAAACAACAAAAATTAGAAATACAAGCCTTGAAGGCAACACAAATA

GAAATGAAACCAGAAATCTCCAGTATAAAATCTCTTGTCATAAAGGGAAAAGCAAAATTA

GGAGATCAAAATGAAATCACTAATACTTCCGATACCTTTAACCCAAGCTTCCTCATGTTA

TTAACAGAAATAACCTTTAGGAAATGGATCATAAACATAATGATAAAAATCAATAATGAA

TTTATTATTGAAACAACAGCACTCTTTGATACAGGAGTACATCTCAATTGTATCAAAGAA

GGCCTTGTTCCAACTAGATACTTTAATAAAATTTCCAAATCCTTAAAATCAATCTCAGGA

GACAAATTAAATATCCAATACAAATTGCCAGAAGCATTAATAATAAAAAAAATCAAATGT

CTTACAAAACCTCTTTTCTTTTAGTCAAAAATATGAGACAAGAAATAATCCTTGGAACAC

CTTTCATACAATTAATTCGTCCTTTTACAGTAACAAATGAAGGAATTGTAACTCATGCTC

TAAATAGAAAAATCACTTTTAACTTCATAACAAAACCTCAAAAGAGAAGCTTAAATATCT

TATAAGAACATTCTATTTCAAAAATAAATTGCCTTATAAAAGACCAAAAACAGAAAAAAA

AAAAAAACAACTTGAATTTTTAAAAGAAGACTTAGATTTCAAAAGAATAGAAGAAAATCT

TACAAAACCTAACATCATAGAAAAAATAGGAACTTTACAAAAACACATAAAGAAAACTAT

ATGCTCTACTTTACCCAATGCTTTTTGGGAAAATAAGAAGCATATTGTTGATTTACGTTA

TGAACAAGATTTTTCTGAAAACCAAATACCTACAAAAGCTTGTCTTACACAAATGAATCA

AGAAGTTCTAAAGTTTTGCCAAACAAAAATACAAGACCTCTTGAATAAAAAAACTCATAA

GAGAAAGTCAATCACCATGGAGCTGTTCTGCTTTCTATGTTAATAAAAATGCTCAACAAG

AAAGAGGAGTTCCCAAATTAGTTATTAATTATAAACCTTTAAATACAGCTCTTAGATGGA

TAAGATATTAAATACCCAATAAAAAAGACTTGCTAAATAGATTATGTAGATCAAAAATAT

TTTCAAAATTTGACATGAAATTAGGATTTTGGTAAATACAAATTAGTGAAAAAGATAAAT

ATAAGACATCTTTTACTATCCCTTTCAGCCAATATGAATGGAATGTTATGTCATTTGGTC

TCAAGAATCTTTAATGAAATTTTCATAGTCTATATAGAATACATTATAGTCTATATAGAT

GATGTTGTGGTCTTCTCCTCTAATATAGAACATCATTTCAAACATTTATTTACTTTCATA

AGAATAATCATACAAAATGATTTAGTAGTTTCTCCTACTAAAATTGCACTCTTCTAGACC

AAAATTAGATTTCTAGGTCATTACATTCATTTAGGTACCATAACTCCCATAGAAAGATTA

ATCTCATTTACTAATAAATTCCCCAATGAGATTAAAGACAAAACTCAATTACAAAAGTTC

TTAAGTAGCCTCAATTATATTATAGATTTCTTTCCCAATATAAATATTCTTTACAAACCC

TTACACCAAAGGTTATAAAAGGATCCTCCGCCATGGACTTCGGTTCATACAAACATTGTC

AAACAAATAAAGATTCATGTTAAATAAATACCATGCTTACATCTAGCTGACCCATCTACT

TTCAAAATAATTGAAACAGATGCCTCAAAAATAGGATATGATGGCATTCTTAAATAGGTC

AAGAGTAGCAATTCATCTCAAAACATTGGAATTCCACCCAAAATTATTTAACAATCAAAA

AAGAAATACTTGCTATAGTTTTGTCAATATCAAAATTCCAACGAGATTTATTAAACCAAA

AATTTCTCTTGCGAATTGATTGCTAATTAGCCAAAAATGTTTTACAAAAAGACGTATTAA

ATATTGCATCAAAACAAATTTTTGCCAAATGACAAGCCATACTTTCAGTATTTGATTTTG

ATATTGAATTTGTAAATGATTCTTCAAATTCATTACTCGATTTTCTCACAAGAGAAGTTT

TACAGGGGATACCATTATGCCAAGACCCAAAAAAGACAAAGGTAAAGCCATTGCCGAAAC

CTCCAATACACCACCAAAGCCAAAAAACTTCTCATCAATCAAGTCATGACTTCAAATAGT

AGAAAATCAATCATTCCTACAACAACATCCAATACCAAATACCTTTCCAAAAGAATAATT

CATACTAGCTCTAGCTCAGCTAGCTGCCATACTACCCAAAGAAACACTGGAAATACTTGC

TCAATCTTTACAAAAAAATAGTAAAGAATGAGCCGTCTAATGCCATTGAGGTAAGCAACA

ATCCTTCCTCGCTTGCTACACAAAGATAAACAGAATACAAAAGAATTTCGAATGAGGTAG

TCATTAGGTCACAGGCAACCCCTCATTCTCAAATATTAAAGTTCTTTCCAAAAACATCAT

TTCAAAAAATACTGACTATAAAAGATGAATTTTTTGACAAAAATCCAATCAAATTTGCAA

AAGATATGTTTATAAAAAATTGGTTTTTCAAACCACACAATACAAATAAAATCATTGCTA

TTTATGAGCATATTTTAGTAGAAACAGAATTAGTAAGGTCCACTCACTTTACTAACAAAA

TTGATACGGAAAACATCTTATACTCCATTATATCCATCAGCAAAGTCATACACCCCTCAC

AATGGAAAGTCTCACCTTATACATCCAGAAAAATTCAAGCCAGGATTACCCAAAACCTAC

CATACTCCTCCTCATATACTTATTAGGACTACCAACAAGCCTGATGGAATGTCTTTTTCT

GACGAAATACAAATTTCTAATACACTTGGCTAGTATTCTTTAAAGATAACTTCCCAAAAC

AAAACCTACCCAACTGGTTTGATTCCTAGTGGGATCTTTTTGGTTCTTTGGATGAAATAC

TACACCCTACAGTCAAACAAGAATACCAAATATTCTGTGAATACTTTGTACCAACACCAC

ATGATACAAGGTTCTCATGCCTTATGCACTTCTTCATAATATTCAAAATCTCATGGGTAA

CAGCATGGGAATTTGATTATAATACATCTCCCATCAAACCTAGTATACCTGTACTTGTTC

GAAAATACAAAACAAAATGGTGGGATGGATTCAAATATCATGAATAGGGATCCGAAGCTA

TGATTAGAACTTGGCTAAACTGCCAAAATACCGAAATAACTACAGTCCCATCAAATACAA

CATTTTTGTAGGAGAAATCTATTACAAGTATAGCACTTATTGCGGTCTAGAATAAAGAGG

AATACTGCAATGTCCTCAAGATAAAACTCTTAGAAGCGGAAGAATCACAATGTGAATCTT

CCAAATCACAAGACAATCGAGATGGATCCTCATAATCACATCACAATGGAAACACCAACG

AAGATGACTGCTAAAGGATCAATATTGCCCAAAATTAAGACAGGATATCACATGCCAATA

CTGTTATAGCAGCGGAAAGGCAAAACACTCTAGCAAGGCAGACATACAATGTTCACTCAG

TACTGTTCATTACATTGTTCAACACCGTAGCATTTTCACTATAGCACCTCCTATAAATAC

GCTCTTTCAACATGGAAGAAGGGGGCTCGATTTCAAATCTCATTTCTTTCTTCTTCTTCC

TCACTCCCTCTTGTAAACCTAATCCCTTCAAGCTTCAAGCTTTTTTACCTTGTACCCCCG

ATTACAAAGTAAGTTTGTTTTTAAATACATTTAGCATAGTTTATACAGGGTTACCAACTT

GCATAATAGTTGGTTAACATTTTCTTGTAATACAGATATCAATACTCCCTAGAGTACGGA

TTAACGCCCTAGTGGATGACTTGATATTCTAGTTCCTGCACTTTTCAATACATTTAATTT

TCAATTTATAGATTACAAGTTCATACCACCTTCATGATTGGTTTTTGCTTCTACATACTC

TTACGTTACATTGTATATGTTGCTTTTGCTCTTTATATCATATTTAATTCTCGAGATTTT

ATTTAGTCTTTTACATGTTTGAAATTATATACAACTTCATAGTCGTACACTTAACTATAT

AGTTAATCTCTGATATTCTACAGACATTTATAGAACCTTAGAATAATAAATTTTCTTATA

CTAGTAGAACTAGATCTATAATACATAGAAACTATACCTTTCTTAGCTCTCATATCTGGA

TCTACTCGATATCTAATAATTAGATATAATCCAAACAAACGTCCAAACAAACATAGACGT

TTATCTTAATTAGGTTTTTTTCACATCCTTAGACTTGTCAATTTCTTATACTTTCTTGTT

CTCTCATATAGTACTTCGATCCTAACCCCTTTA

>EgranV_sc3 [endogenous-virus-name=Eucalyptus grandis virus] [strain=sequence cluster 3] [host= Eucalyptus grandis accession BRASUZ1] [moltype=genomic DNA] [note=incomplete genome, mutations in ORF1 and ORF2] 7762 bp

GTGGGAAGGTATAGATAGTCTTTTTAAGTCCATACTCACAGGTCGAGAGCCATTCGTTGA

CATCTTTCTTGTTTCTATAGAGTGGTCTAGGTCGTAAGTCCCTAAGAGGCAGTGAAATTG

TTGGTTTAGCTTTGTAACGATTTTGAAGAAGGAATAGTTCTAATTTTAGGTATGAGCTTA

CTTAGAAGATCTACATCTTTCTCTAGTTCTCTATTACAAATGGACTCAGATCTAGATCCT

ATACATAGGGAAGAAGTAAGAATAGAATATTTCAACAAATTTATAGAAAATTGGGAAATT

CCTAAAGTCCAACTTGAGGAATATACAAACAATTAAAATTGAAATTTTTCAAAAAATCAT

GTTATATCATAGCTACTGAAAAAATAGATATTCATCTTTCTAATTCTTATGAAAAAAATA

CATCTTTTGAATTAAGAGTCTATCAAAAAAGCATAAACAAAAATACAATTATATTCATAT

AGGACTTGTCCAAGTAGGAATCAAACTTCTTACCAAAGAATGTCTTAAAACTATGATGAA

TCCATTCTCGGTACTATAGAGACAAGTCTCTACAAAGGACCAATACATTTTGACTATTAC

CCTAATTTCTCAGTGTCCCTTAAGGATAAGAATATTCTTAGAGCCCTCACACTTCAAATA

AAAACCCATAATTACAAAGTAGCAAATAGATCTATTCATTTAGCTCTTGTCTACAGGATC

CATTACAAAGCCATGGCCTCTGCCTTTGGAGAAAATGCTTTTAAACATAGTCCATGGGGA

GAAACACTTCTCCACCAAACTGACATTTCTAGAGCAAACACTATAATTCCTAAATCTATT

AGTTAGAATTAAGTCACTTTACCTTAACAATAGGAATTAGAAAATCAAATTCCCCAACAA

GTTGTTCAAAATACAAATCAAACCAATGTTATTCAACATCCAAAAGGAAGGTCACTATTA

GGTTCCCTAGAAGATTTTTCGATTCAAGATCTAGCCCTTCATTTCATACATTTTATTCAA

GATCTATAGACCTTAATCATAATCTTCCACCCATAATCACCATTCTTCCAAGTACTCATA

GAGATACTCCCATACGAGATACAACTCCTTTACCTTATAGTACTAATAGAAATAATCTAA

AGACACCTTCTGAACCAGAAATTAGTGGTCTAGATAATAGATTAGGGATTTCCATGCTAG

TTTACCAACAAAACCAACCCGAATCGGTCTTAGAAAACATAGACTCTCCTCATCATTCTC

CTACATATTCACAAATGATAGATGCAAAATTAAATGTTTTAGAAAAACAATTTGAACCAA

ATAGAGCATTCCTTAATATGGAATTTAATTTAGAAGACAACAAACAAATCAGAGAATGGT

TCTTCAAAACCTTTGCTGATAAAGCAAAATATATCAAACAAAAATATTACGACTACATAT

ATAAATATGAAGTGCATATATTTTTCTTTGATTGGTTAGAAGAACAATTCCTCAAACCAC

AAGAAATAGTTATTGATAATGGTGAAACCATAGAATCCAATCATCTACCCCTTAGGAGAA

TAAAAATACAACATGGAGAAAGTAAGGTAATAGTTACTCCTTTTAGGGTTCTAGACAAAA

CGGATTTCCTTTACACTCATAAGGTTATTGAACAAAACAATTATACTAATCAACATCTGG

TAATCATAGGAAAACAATTGAATAGGATGGAGTCTACCATACAAAATCCTACCTACATGA

TCACACCACAAGTAGAAACACTCAAAAAACCCATTTTTAGGCTTTTGAATTCCCAAAAAA

TCTTAGCCCAAAGTTTGCTGAAGCCATGGAATAAAAGAAAAAACTTTTAGGAAAATCAAA

GCTAGACCTTGCCCCTATAATAGATACCCCTAAATTCCAAGGACTAATTATTAGGGACAC

TCCAGACCAACCCAATATTAGTACTTTAACGAAACCCTTTAATGACTCTGAAAATGAATC

AGTCGCTGAGGTAAACAGACTCAACTGGAGACAACCTTAAAAACTTTATTATAGTAGAGC

TACCCCACCTGACATCGCTCAAGAAGAATCATTGCAAAAAATACAAAATAAATACTCTCC

ATATGCCATTTATGAATGGAATATAGATGGTGTAGCCGAAAGCAATATTATGAACATTTT

AAACAATATGGTAATGCTTGCTAATGCCTACAAAACACAATAAGATATTTCTGATCATGC

TGTTGCTAATCTTTTAGTAGCCAGATTCATTGGACAACTAAAAGGTTGGTGGGATAACTA

TTTAACTGATGAGTAGATAATTCAAATTCTAACTGCTTATAAACTAAACGACTTTGGACA

ACGCATGGATGATGATCAAGGACATCTCATCCAAGATGCTGTGAATATTCTTATTTACTC

CATTTCACAATACTTCATAGGAGACCCTTCACATATTAAAGATAGGAACCAAGATCTTCT

ATCCAACATATGATGCAAATCCTTAGGGAGATTTAGAGGATACACAGAACTTTTCCTTCA

AAGGGTTCTTGTTAGACTAGATTGCAACCAACCCTTTTGGAAAGAAAAAGTTTTAGTAGG

TTTATCCCAAATAATATGTGACCGAGTTAGAGATAGAATTAGATAAGAATACAATGGTCA

AATACCTTATGCCCTACTTTCTTATGGAGAAATGGTCTCCTACATTTATAGGCAAGGAAC

GCAAATGTGTAAATCAATCAAATTACAAAAACAATTCACGGAAGAACAATCTCTCATCAA

CAGAAGCTTGGGAGACTTTTGTGCCAAATACGACCCCTCTTATAATGATAAAACAAAACC

AAAATGTAAAGGTACTTGCCATTCTGAGAAAGAAACAAAGAAATATCGCAACAAATCACA

ACCTCATAAAAATAAAAGAAGACACCATAATAAACATCAGACTAATAAGAAATTTTTTAA

AGACATTAAATGTTACTCTTGCGGTAGAAAATGGCATATTTCAAAATATTGTAGGGTTAA

TAAAAGATTAAATGAACTTTCTTTAGATAATAATACTTTATATTACTTAAACAACATCCG

TGTAGAAACTGGTGATACTTCTTCAGATGAATTTCTTGAAGAAGAAGGTCTTCAAGTTAA

TGAAATAGAAACCTCTTCAAAATCAAAATATGAAGACGCCTCTCCTAAAAAACCTATTCC

TGAAATAAACATGCTAACAAAAGAAGAAGAATTCCTCCTTAGCACTGCTAATAAAATAAC

TAACCCATGAGCCAAAAAAAGAAATACTTAGATAGACTACAGTCTTCAATGAATATTCTT

ACTAAACCCAATACTTTCTACAACCTAAAAGACATTTTGAATAGAAACAAAAAAAAAAAA

ACAAAAAAAAACCCAGTCCAGACCCATAAATATTAATGATCTCCAAAATGAAATAAAGCA

GCAAGAATTAGAAATACAAGCTTTGAAAGCAACACAAAATGAAACAAGAAATTTCTAATA

TAAAGTCTCTTGTCATAAAAGGAAAGACAAAATCAGAAGACCAAAATGAAATCACTAATA

CTTCCATTACTTCTAACCCAGCCTTTCTTATGTTATTAACAAATAACCTCTAGGAAACGG

ATAATAAATATAATGATAAAAATCAATAATTAATTTATTATTGAAACAACAACACTCTTT

GATACAAGAACAAATCTCAATTGTATTAAAGAATGACTTGCTAACAAGATACTTCAATAA

AACTTCAGAACCTCTAAAATCAGTCTCAAGAGACAAATTAAATATCCAATACAAATTGCC

AGAAGCCTTGATAATAAAGAACCAAATGTCTTATAAAACATTTTTTCTTTTAGTCAAAAA

TATGAGGCAACAAGTAATTCTTGGAACACATTTCATACAATTAATCCAACCTTTCACAGT

TACTAATGAAGGAATTGGAGCTCATGCTCTTAATAGAAAATTTACCTTTAATTTCATAAC

AAAACCACAAAAAAGAAGCCTTGATATCTTACAAGAACATTCTATTTCACAAATAAATTG

CCTTATAAAAGACAAATAAAACTAGCTTGAATTCTTAAAAGAAGATTTAGAATTCAAAAT

AATAGAAAAAAATCTTATAAAACCAAACATCATAGAAAAAATAGCCTCCCTACAAAAACA

CAGAAAAAATAGCCTCCCAATGCTTTTTGGGAAATAAAGAAGCATATTGTTGATTTGCTT

TATGAACAAGATTTTTCTGAAAACAAAATACCTACAAAAGCTCGTCCTACACAAATGAAT

CAAGAAGTCCTAAAATTTTGCCAAACAGAAACACAAGACCTCTTGAATAAAAAAACTCAA

AAGAAAAAGCTAATCTCAATGGAGTTGTTCTGCTTTCTATGTCAATAAAAATGCTGAACT

AAAAAAAGGAGTTCCCAGATTAGTTATTAATTATAAACCTTTAAATACACCCCTTAGATG

GATAAGATATCCAATACCCAATAAAAAAGACTTTATAAACATATTATGTAGATCAAAAAT

ATTTTCAAAATTTGACATGAAGTCAAGATTTTAGCAAATACAAATTAGTAAAAAGATAAA

TATAAGACAACTTTTACTATCCCTTTCGACCAATATGAATGGAATGTTATGCAATTTGGT

TTCAAGAATGCCCCTTCAGAATTTCAAAAAATCATGAATGAAATTTTTTATCCTTATTTA

GAATACATTATAGTCTATATAGATGATGTTCTAGTTTTCTCTTCTAATATAGAACAGCAT

TTCAAACATTTATCCACTTTCATAAGAATAATCATACAAAATGTCTAGTAGTGTCTCCTA

CTAAAATTGCACTCTTCCAAACTAAGATTAGATTCCTAGGTCATTATATCCATCTAGGTA

CCATAGCTCCCATAGAAAGATCAATCAATCTTATTTACTGATAAATTCCCATATGAGATT

AAAGACAAAACTTAATTACAAAGATTCTTAGGTAGTCTCAATTATATTATAGATTTCTTT

TCCAATATAAATATTCTTTGCAAACCCCTATACCAGAGGTTACAAAAGGATCCTCTACCA

TCGACTTCAGTCCATACAAACATTATCAAACAAATAAATATTCATATTAAAGAAATACCA

TGCTTACACCTAGTTAACCCATCTACTTTCAAAATAATTGAAACAAACGCCTCAAAAATA

GGATATGGTGGCATTCTTAAACAAGTCAAAAATAGTAAATAACAAGTTTTTCAGTTTATC

TCAAAACATTGGAATCTCACCCAACAAAACTATTCAACAACCAAAAAAGAAATACTTGCT

ATAGTTTTGTCCATATCAAAATTCCAAAAAGATTTATTAAACCAGAAATTTCTCTTGCAA

ATTGATTGCAAATTGGCCAAAAACATTTTACAAAATGACATATTAAACATTGCATCAAAA

TAGATTTTTGCCATATGGCAAGCCATACTTTCAGTATTTGATTTTGACATTAAATTTATA

AATAATTCTTCAAATTCATTACTTGATTTTCTCACAATAAAATTTTTACAGGGGATATCA

TTATGCCAAGACCCAAAAAAGACAAAGATAAAGGCAAAGCCATTACCGAAACCGCCAATA

TCACCAAAGCCTAGGCTTAACACCAATCAAGTCGTGGCTTTAGATAGTAGAAAATCAATC

ATTCCTACAACAACATCCAATACCAAATACCTTTTCTAAAAGAAGAATCCATACCAATTC

TAGCCTAGCTAGCAAACATACTATCCAAAGAAACACTGGAATTACTTGCTTAATCTTTAC

AAAAAAAAAAAACAATAAAGAATGAGCCATCCGATGCCATTGAGGTAAGCAACAATCCCT

CCCCGCTTGCTGCACAAAGATAAGTGGAATACAAAAGAATTTCAAATGAGGTGGTCATTA

GGCCACAGGTAACCCCTCATTTTCAAAAATCAAAGTTCTTTCCAAAAACATCGTTTCAAA

AAATACTGACTGTAGAAGGTGGATTTTTTTTACAAAGATCCAATCAAATTTGCAAAAAAC

ATATTCCCAAAAAATTGGTTTTTCAAACCACACAATACAAATAAAACCATTGCTTTTTGT

GAGCAATTTTTTGTGGAAGCAGAATTGGCAAGGTTCACCCATTAGCAAAGTCATACACCC

CTTACAATAGGAAGCCTCACCTCATACATCTAGAAAAATCCAAGCCAGAATTACCCAAAA

CCTACCATACTCCTCATCATACACATACTGAGACTACCAACGAGCCTGGTGGAATATCTT

CTTCAGACAAAATACAAATTTCCAACACACTTGGTTAGTATTCTTTAAAGACAACTTTCC

AAGATAAAACATACCAAATTGGTTTGACTCATGGTGGGATCTCTTTGGTCCTTTAGATGT

AATACTACATCCTATAGTCAAACAAGGATACCAAATATTCCGTGAATACTTTGTACCAAC

ACCACATGATACAAGGTTCCCTGCCTTATGAACTTCTTCATAATATCTAAAATCTCATGG

GTAACAGTATGGGATTTTGATTATAATACATCTACCAAACCTGGTGTGCCTGTACTTGTC

CGAATATACAAAACAAAATGATGGAATGAATTCAAATAATATGAACAAGGATCCAAAGCT

GTAGTTAGAACTTGGCTAAATAACCAAAATAGTCAAATAACTACAATCCAATCAAATACA

TCATTCTTGTAGGAGAAATCCATTGCAAGTGCAGCACTTGCTGCAGTCCATAGTAGAGAG

GAATACTGCAATGTCCTCAGAACAATGCTCTCAAAAGTGGAAGAATCACAATGTGAATCT

TCTAAATCAGAAGACAGTCAAGATAGATCTTCACAGTCACATCATAATGGAAATACTAAT

GAAGACGATTGCTACGGGATCAATCTTGCCCGAAATTAAGACAAGATATCACATGCTGAC

ACTGTTGCAGCGGCGAAAAAGCATTACACTATAGCACGGCAAACATGCACTGTTCACTTA

GTACTATTCACAAAGTATTGTTCACTTTACATTGTTCAGCACTGTAGCACTTTCACTGTA

GCACCCCTTATAAATACGTTCTCTCGACATAGAAGGGTGGCACCAAATTTTCATATCTCA

TTTTCTCTTCATCCTCACTCTCTCTTGTAAACCCTTATCCCTTCAAGCTTCAAGTTTTCT

TACCTTGTACCCCTAGTCACAAAGTAAATTTGTTTTTCAAATACATTTAGCATAGATTAT

ACATGGTTACCAACTTGCATTATAGTTAGTTAACAATTTCTTGTAATACAAACATCAATA

CTCTTTGAAGTGCAGATTGCCGCCCAAGTGGATGACTTGATATTCTAGTTTCTGCATTCT

TCAATACATGTAAACTCCATTTTATATAATACAAGTTCATACCATCTTCATGGTTGGTTT

TTGCTTTTGCATACTTTTACTTCATATTCTATATATTACTTTTGCTCTTTATATTATCTT

TAATTTGCAATATTTTAATTAGTCTTTTACATGTTTGAAATTATATACTTATTCATAATC

ATAGTCTTAACTATACAGTTATTTTTTTATATTGTACAAACATTCACAAAAACTCTTAGA

GTAACAAATATTTTTATACCATTAGAATCAACTACGAAGACATAGAACCTATTCATTTCC

TACTTCTCATATCTGAAACCGTTTGATATTTAAGAATTAGATAAAATCCAAACAAACGTA

GACGTTTAGATTAATTTCTTATAATTTCTTAGATATGTTTGTCTATCTCTTATATACTTC

CTTGTTCTCTCATAAATTAATT

>EgranV_sc4 [endogenous-virus-name=Eucalyptus grandis virus] [strain=sequence cluster 4] [host= Eucalyptus grandis accession BRASUZ1] [moltype=genomic DNA] [note=complete genome, mutations in ORF1 and ORF2] 7731 bp

TGGTATCAGAGCGAGTGGGAAGGTATAGATAGTCTTTGTAAAAACATATTCTCATATCGA

GAGCCATTTGTTGGCATCCTTCTTGTTTCTACAGAGTGGTCCAGGCCATAAGTCCCTGAG

TGGCTGTTAGACCGTTGGTTTAGCTCTGTAACGGTTCTAAAGAAGGAACAGTTCCGATCT

TAAGTATGAGTTTACATAGAAGATCTACATCTTCCTCTAATTCTCTATCACAAATGGACT

CAGATCCAAGTCCTATACATAGAGAAGAAGTAAGGATAGAAGATTTTAACAAATCTACAG

AAAATTGGGAAATTCCTAAAATACAGCTTAAGGAAATATACAAACAATCAAAATTGAAAT

TTTTCAAAAAATCTAATTATATCATATCCACCGAAGAAAGAGATGTTCCTCTTTCTAATT

CCTATGAAAAAAATACATCTTTTAAACAAAGAGTCCATCCAAAAGCATAAACAAAAATAC

AATTACATTCATATAAGATTAGTCCAAGTAGGAGTCAAACCTCTTACAAAAGAAGGTCTT

AATACTTCTATTCTCTTAGTTCTAAGAGATTCACGTCTCTTAAATTATGACGAGTCTATT

CTAGGTACTGTAGAGACAAGTCTCTGCAAAGGACCTATACATTTTGACTGTTATCCCAAT

TTCTCCGTTTCCCTCAAAGATAAAAACATTCTTAGAGCTCTAACACTCCAAATCAAAACC

CATAATTATAAAGTAGCTGATGGATCTATTCCTTTAGCCCTTGTCTATAGAATTCATTAT

AAAGCCATGTCCTCTGCTTTTGGAGAAAATGCTTTCAAATATAGTCCTCGAGGAGAAACA

CTTCTCCTTCAAACTGACATTTCTAGAGCAAATACAACAATTCATAAGTCTATTAATTGG

AACCAAGTTACTTTACCGGAACAATGGGAATTAGAAAACCAAGTTCCCCAACAAGTTGTT

CAAAATACAGATCCTACTAATGTCATTCAACATTTAGAAGGAAGAGTGACCATTAGATTT

CCTAGAAGATCATTCGATTCACGATCCAGTCCTTATTTTCATAATTCCTATACGAGGTCC

GTAGATCTTAATCCCAACCTTCCTCCTATAATCACCATCCCTCCGAGTACTCATAGAAAC

ACTCCTCCTCCTAGTATGCATAGAGACACTCCCACACACGATACAACACCTTTACCCCCT

TCCACTAGTAGAAGTAATCATGAGACACCTCCTGAACCAGAAATTAGTGGTTTAGATAAT

AGATTAGGGATTTCCAGGCCTATTTATCAACAAAATCAGCCTGAACCAGTCCTAGAAAAT

ACAGATTCTCCTCATCATTCTCCCACTTATTCGCAAATGATAGATGCAGAGCTTAATGTT

TTAGAAAAACAATTTGAACCAAACAAGGCTTTCCTTAATAAAAAATTTAATTCTGAAAGC

AACAGACAAATCAGAGAATTGTTCTTCAAAACCTTTGCTGACAAAGCAAAAGACATCAAA

CAAAATTATTACGACTACATATATGAATATGAAGTCCATATATTTTTCTTTGATTGGCTA

GAAGAACAATTCCTTGAACCAAAAGAAATAATTGTTTTAGATCATCATTATAAGTGGAAA

CTTGATAATGGTGAAACCATATAATCCAATCATCCCCCTCTTAGACGTATAAAAATACAA

CATGGAGAAATCGAGGTTACAGCTACCCCTTTTAGGCTTCCAGATCAAGGAGACTCTCTT

TATACTCACAAAGTCATTGAACAAAATAATTTCACTAATCAACACTTAGCGACCATAGGA

AAACAGTTGAATAGGATAGAATCCACCATACAGAATCCTACCCAACTCACCACACCACAA

GTAGACACTTTTAAAAAACCCATTTTTAAAGCTTTTAAATTTTCAAAAAATCTTAGCCCA

AAGTTTGCAAAAGCCATAGAACAAAAGAAGAAACTCCTAGGGAAATCAAAGCTAGAGCCT

ACTCCTATTATAGACACCCCTAAATTTCAGAATCTTATCATTAGAGACACTACTGACCAA

CCCACTATCAGTATTTTAACCAAACAATCTGGTGACTCTGATTATGAATCGGTCGCTGAG

ATTAATAGACTCAATTGGAGACAACCTCAGAAGCTGAGATTAATAGACTCAATTGGAGAC

AACCTCAGAAGCTTTACTATAGTAGAGCCACTCCACCTGACATTGCCCAAGAAGAATCAT

CACAGAAAATACAGAATAAATACTCTCCTGATGTCATTTATGAATGGAATATAGATGGTG

TTGCCGAAAGTAATATCATGAATATTTTTAACAACATGGTAATGCTTGCCAATGCCTACA

AAACTCAGCAAGACGTTTCTGATCATGCTGTTGCTAATCTTTTAGTTGCAGGATTTACTG

GACAACTAAAAGGTTGGTGGGATAATTATTTGACTGATGAATAGAAAATACAAATTTTAA

CCGCTTATAAGATTGATGATTTTGGCCAACGCATGGATGATGATCAAGGACATCTCATCC

AAGATGCGGTGAATACTCTTATCTATTCTATTTCACAACATTTTGTAAGAGACCCTTCAC

ATATTAGAGATAGGAATCAAGATCTCTTATCCAATATGCGATGCAAATCCTTAGGTAGGT

TTAGGGAATACAAAGAACTCTTCCTTCAAAGGGTTCTTATCAGACCTGATTGCAATCAAC

CATTTTGGAAAGAAAAATTCTTAGCTGGTTTACCCCAAATTATAGGTGATCAAGTTAGAG

ATAAAATTAGAGAAGAATACAATGGCCAAATACCTTATGATCTTCTTTCTTATGGAAAAA

TAGTCTCTTATGTTTACAGACAAGGAACACAAATGTGTAATACTATCAAATTACAAAGAC

AACTCAAGGAAGAACAATCACTCACTAATAGAAGTCTAGGAGATTTCTGTGCTCAATACG

ACCCATCTTATAATGAAAAAACAAAACCAAAATGTAAGGGAACTTGTCCTTCTGAGAAAG

AAACAAAGAGACATCACAACAAACCTCAACCTCGTAGAAATAAACAAAGACACCAGAATA

ACCAAAATAAACCTCAAACTGATAGGAAAAATTTTAAAGACATCAAATGTTATTCTTGTG

ATAGAAAAGGCCATATTTCAAAATACTGTAGGATTAATAAAAAATTAAATGAACTTTCCC

TTGACGACAGTACTCTTAACCAGTTAAACAACATCTTCATAGAAAATGGTGATACTTCCT

CTGATGAACTTCTTGAAGAAGAAGGTCTTCAGATTAATGAAATAGATTCTTCTTCTGACT

CTGATTATGAAGACGCCTCTTCAGAAAAACCCGTTCCTGAAATAAACATGTTAACAAAAG

AAGAAGAATTTCTCCTTAGCATTGCTGATAAGATCACTGACCCAGAAGCCAATAAAGAGT

ACCTAGATAGACTACACTCTTCCCTGAATATTATCTCCAAACCCAATACTTCATACAATC

TAAAAGACATTTTAAATAGGAACAAAAAACCTCAGTCCAGACCCATTAATATAAATGACC

TCCAAAATGAAATAAAACAACAAAAATTAGAAATTCAAGCCTTGAAAGCAACACAGTTAG

AAATGAAGCAAGAGATTTCTGACATAAAATCTCTTGTCATAAAAGGAAAGACAAAATTAG

AAGACCAAAATGAAATCACTAACACATCTGATACTCCGGACCCAGCCTTTCTTATGTTAT

TAACAGAAATAACATCTAGAAAATGGATAATAAATATTATGATAAAAATCAATAATGAGT

TTATTATTGAAACAACATCACTCTTTGATACAGGAGCTGATCTCAATTGTATCAAAGAAG

GCCTTGTTCCAACAATATACTTTGATAAAACTTTCGAATCTTTAAAATCAACCTTAGGAG

ATAAACTTAATATCCAATACAAATTACCAGAAGCCTTAATAATAAAAGATCAAATGTCCT

ACAAAACATCTTTTCTTTTAGTAAAAAATATGAGGCAACAGGTAATTCTTGGAACACCTT

TCATACAATTAATCCAACATTTTACTGTTACTAACGAAGGAAACGAAACCTATGCTCTAA

ATAGAAAAATCACCTTCAACTTCATAACAAAACCACAGAAGAGAAGCCTAAATATCTTAC

AAGAATATTATATTTCAGAAATAAATTGCCTTATAAAAGACAAAGAAAACCAGCTTGAAT

TTTTAAAAGAAGATTTAGAATTCAAAAGAATAGAAAAAAAATCTTATAAAACCAAACATC

ATAGAAAAAATAGCTTCCCTTCAAAAACACATAGAAAAAACTATATGCTCTACTCTACCC

AATGCCTTTTGGGAAAGAAAGAAGCATATAGTCGATTTACCCTATAAACATGACTTTTCT

GAAAACAAAATACCTACCAAAGCTCGCCCAACACAAATGAATCAAGAAATTTTGAAATTC

TGTCAAGCAGAAATACAAGATCTCTTAGATAAAAAACTCATAAGAAAAAGCCAATCACCT

TGGAGTTGTTCTGCTTTTTATCTCAATAAAAATGCTGAACTTGAAAGAGGAGTTCCCATA

TTAGTCATTAATTATAAACCTCTTAATACAACCCTTAGATGGATAAGATATCCTATACCC

AATAAAAAAGATTTGTTAAACAGACTTTGTAGATCAAAAATATTTTCAAAATTTGACATG

AAGTCAGGATTTTGGTAAATACAGGTCAGTGATAAGGATAAATACAAGATAGCCTTTACA

GTCCCTTTTGGTCAATATGAATGGAATGTTATGCCATTCGGCCTCAAAAATGCCCCTTCA

GAATTCCAAAGAATTATGAATGAGATTTTCAATCCTTATTCAAAATACATCATAGTTTAT

ATAGATGATGTCATCGTATTCTCTTCCAATATAGAACAATATTTCAAACACTTATCCACT

TTCATAAAAATAATCATACAAAATGGTCTTGTAGTTTCTCCTACTAAAATTGCACTCTTC

AAAACCAAGATTAGATTCTTAGGTCATTATATCCATTTAAGTACTATAACCCCCATTGAA

AGATCAATCTCTTTTACTGATAAATTCCCTGATGAGATTAAAGACAAAACTCAATTACAA

AGATTTTTGGGTAGTCTCAATTATATTCTAGATTTCTTTCCCAATATAAATATCATTTGT

AAACCTTTACACCAAAGGTTACGTAAGAATCCTCCACCCTGGACCTCTGTCCATACAGAC

ATTGTCAAACAAATAAAAATGCATGTAAAAGATATACCATACTTATACCTTGCCGACCCT

TCCACTTTCAAAATAGTTGAAACAGATGCCTCATAATTAGGATATTGTGGTATTCTCAAA

CAGGTCAATAACAACAAAGAACAAGTTGTTTAGTTCATTTCAAAACATTGGAATTCCACT

CAACAAAATTATTCGACAATCAAAAAAGAAATACTTGCTATAGTTTTGTCTATATCAAAA

TTCCAAGGAGATTTATTAAATCAAAAATTCCTCTTGAGGATTGATTGCAAATCAGCCAAA

GACGTTTTACAAAAAGATGTTTTAAACATTGCATCAGCCATACTTTCAGTATTTGATTTT

GACATTGAATTTATAAATGGTTCTTCAAATTCATTACTTGATTTTCTCACAAGAGAATTT

TTACAGGGGATACAATCATGCCAAGATCCAAAAAAGACAAAGATAAAGGCAAAGGCAAAG

CCACTGCCGAACCCTCCAATACATCACCAAAGCCAAAAGGCTTAACATCCGTCAAGACAT

GGCTACAAATGGCAGAAAACAACCAGACATACTTACAACAAACACCTTTAAAAATAAAAC

CCATATCATCTGAACCAATGCTAGCCTTTAAACAGTTAGCCGACATGCTACCGAAAGAAA

CAATATTGCTACTTGCCTAGTCTTTACAAAAAGACGTAAAGAATGAGCCATCTAGTGCCA

TCGAGGTAAGTAACAATCCTTCCCCGCTTGCTACACAAAAACAAGCGGAATACAAAAGAA

TTTCAAATGAGGTGGTTATTAGGCCACAGGCAACCCCTCATTCTCAAAGATCAAAAATTT

TCCCCAAAACATCTTTTCAAAAAATACTGTCTGTGGAAGATGGATTTTACGATGATAATC

CAAACAAATTCGTAAAAAACATCTTCGCAAAAAACTGGTTTTTCAAATCACACAATACAA

ACAAAACCCTTGCTTATTATGAGCAAATCCTAGTAGAGACAGAATCAGTAAGGTTTGCCC

ACTTTACTGACAAAAACAATGCAGAAAACATCCTATACTCCACCATGTCCATCAGTAAAA

TCATACATCCCTTACACTGGGGAGATTCACCTCATACACCCAGAAAATTCCAAACTAGAC

TTACCCAAATTTTACCATATTCCTCCTCTTATACCTACTGGGACTACCAACAAGCCTGGT

AGAATGTTTTTTACAGACAAAATACAAATTTCCAACATACTTGGCTAGTATTCTTCAAAG

ACAACTTCCCAAGACAAAACCTACCAAACTGGTTCAGCTCCTGGTGGGATTTCTTTGATC

CTTTAGACATCATACTACACCCCACAGTCAATCAAGGATACCAAACATTTCGTAAACACT

TTGTACCAAATCAACATGATTCAAGGTTCTTATGCCTTATACATTTTTTCATAATATTCA

AAATTTCATGGGTAACAGCCTGGGAATTTGAATACATTACATCCACCAATTCTGTACCAA

TACTCGTTCGAAAATACAAGACTAAATGGTGGAATGGATTCAATAGACATGAACAAGGAT

CCGAGGCTGCGGTTAACACATGGCTAAATAGCCAGAAAAATCAAATAACTACAGTCCCAT

CAAATACATCTTTTCTACAAGAAAAATCCATTGCAAGTGCAGCATTGGCTGCAGTCCAGA

GCAAGGAGGAATACTGCAACATCCTCAGAACAATGCTCTCTGAAGCAGAAGGATCACAAT

GTGGATCTTCCAAATCTCAAGACAATACAGACGAATCCTCACGGTCACATCATAGTAGAA

ACACCAATGAAGATGACTGCTACAGGATCAACCTAGACAGAGATTAAATTCAAGACATCA

CATGCTGACACTGTTACGAGCGGCGCAAAGGCTATACATCGTAGCATACACTGTTCACTG

TTCACTATTCACTTTACACTGTTCAGCACTGTAGCACCCCCTATAAATACACTCTCCCGA

CATAGAAGAAAGGGGGCACTCTCCCGACATAGAAGAAAGGGGGCTCGAAATTTTCCAGAT

TTCTCTCTTCTTCTTCTCCCTCTCTCACTTGTAACCCATCTTGTATCCCCTAGTTTCAAA

GTAAGTTAGTTTTTCATATACATAGTTTCATACAGGGTTACCAACTTACATGATAGTTGG

TTAACCATTTCTTGTAATACAAATATCAATATTCCCTGGAGTACGGATTACCGCCCTAGT

GGATGACTTGATGCTTTAGTTTCTGCATCCTTCAATACATGTAATTTCAGCTTTTCAGCT

TTATATAATACAGGTTCATACCACCTTCATGGTTGGTTTTTGTTTATGCATATTCTTACT

TTATATTTTATATATTGCCTTTGCTTTTTATATTATTTTTAATTCACGCGATTTTATTTA

CTTGTTCTTATACTTTTCTTTTAGATACATCATTCCCTATTTTCATATAGATCTGTTAAT

CATTTATACTTCCTTGTTCTCTCATATAGTACTCCGATCCTAATCTCTTTA

>FvesV_sc1 [endogenous-virus-name= Fragaria vesca virus] [strain=sequence cluster 1] [host= Fragaria vesca virus] [moltype=genomic DNA] [note=complete genome] 7725 bp

TGGTATCAGAGCCAAACGGCCTGATATAGTTAATCATTCAAAAACCTCCAACCCCAGGTG

GCGGACTACCGCCTAGGAGTGGTTCTGGAGCTTCATTCAAGCTATATGTATTAAACTTCT

CAATCCCCAGGTGGCGGACTACCGCCTAGGAGTGGATCTTGAGTTGATCTATCTGCTTTA

CTTATTTTGTGAAATTTTGAGTAAACTCCCTTAATTTTAAAGATTTTTGGTTTTATTTTC

TTTTTCTTACTTCCTTTTAAGCCTGTTATTCTCTCCAACCAAACATTAAGTCCAGGTCCA

AACACTAAGTCCCAGTTGAGGCATGAGCGGAGAAGAGCGAGTATTAGTCTTTAGGGTCTT

AAGCCAAGGATCTTAAATCCTCCTATCTTTTTAGTTTCGGTCTTGTTTTCTCTTCTTTTC

CCAAAATGAGTCGACTATTTAGATCCAACTCAGTCACTTCTAGTTCGTCTAGGTCCTCCC

TCAATAGGATTCCAGATATAGTCAACGAAGAACAAATAGAGTATCAGTCAAACGATAACA

TAGATATGAATGACTGGAATATTCCCAGAGTTCCTAGTCATGAAATTTATAAGAAAAAAT

GGAGTCTAGCTTCATTCAAAAGTGAACACCATATTAAATCAGTAGAACAAGTTTATGCCC

TTAGTAAACAACATGAAACTTGCCAACTTTTTAGTCCAGAATCAATTAAGAAACATAAAA

AAGATGGTCATAATTTCCTCCATATTGGATTAGTTCAAATTGGTGTCAAGCCTTTAACTA

GAAAAGGTCTTAATGCCTCCATTCTTTTATGTTTAAGAGATGCAAGGTTTACTGATTTTA

ATGACAGTACCCTTGGATTAATTGAGTCAAGTCTGTTTAATGGTCCAGTTCATTTTAATT

GCTACCCAGATTTTACTATTAGCCTCAGTGATCCTCATATCCTAAAAGCTTTAACCCTCA

ATATTAAAACTTCAGGTTATCATGTCCTTGAAGGAACCCAACCTTTAGCCTTAATTTATA

GAATCCATTACAAAGTCACTGGCACAAACATGAATTTCCAAGCCATTAGTAAAAGTCCCA

AAAACCATACTCTCTTAATTCAAAGTAGTCAAGAAAACGCCAATATTAGCATTCCTCACA

CAATCAGATGGTCAGATGTCCATCTTCCTTCTGAATGGTCTCTCACTAGTGAGAATCAAC

CATCTAATGCACAACATAGTCTTTCTGATTTAGACTGTATTCAACAGTTTAATGATGGCA

CAGTAAGAATTAATTTTCAAAACTCTAGGATAAGTCAAAACCCAAGAATTCAAGAGGTAG

GAAACTCCTCTAGACATTCTTTTGCTACTGCTAGACATTCATTTGCTGGTTCCACTACAG

CTGAAACAATGTCTAGACGAGATTCTGATCTAGATAAAGAAATTAAAAATATTAGGATTA

GAGAATTAGAAGAAGAATTGCAAAAGCTCAAAATTAAAAACATCAAAACTACTTCCCAAG

TCAGTTACCCAAATTATACAGAAAATGAATCTCAAAATATTTCAACCACAGATGAAGAGC

AAACTTCTCCTACTGCTTCTGATTTTTTAGTTACTCCCCCACTAAATCCTCAATTAAGCA

CACTTACCCAACCTTTCGTAATCAACTATTCTAGATTAGATAAACATCTTGAATCAAACG

AAAATATCCTTAGGAGAAACCTCTATAGGATGAAATTCCCTTTCATGGATCAACGAAAGC

AATTATTTCATGATTGGCAAGCATTTATGCTTGAAACAAGATCTGAAATATTCTTTCTTG

ATTTTATAGATCAACTAAACAACCAAAAAATTCAAACATTAACAAAAGCAAGATGGAAAA

CCTCCACTAGCTCAGTCCTTTCCAGTCATCCCCCTGTTGAAACAATAATAATTGATCATC

TTAACAGTCCAATCACAGCTTCACCTTTTAAAATTGCTATAGAAGATCCAGACACTAAGA

AAATTAACCCAGATACTAAGAAAATTATTGAACAAAATAATTATACAAACCAAAGCCTAG

TAACCATAGGAGCCCAGTTAGATAAAATAGAAACAAAGGTTGACAACATATCCTCTAAAG

TTAATATCCTACCACAACCTAAACCAGAAACTCCTTTAGTTCATTTTAGTGAATTAAATA

AACCTACCCTAAATCCTACTTCTTCGATTCAAAAGATCGAACGAATGTTAGAAAAATTAA

AAACTGAAACACCTGAACCCAGTAGAATTGCTGTAATTCATACCCAACAAACCTCTTCAT

CCACTTCTTCAGATAGCGATTCTAGTAACACTAGCCAAATTAAAGAAATAAACCAAATAC

TTCAAACCTTAAATATCGAACCTTTAAACCTAGATCGAATAGTTCATCCAAAGAAGCGAC

CTTTTAAAACTTGGACAGAACGTCCTCTCCCCTTAGATATACAACCTACTAATCACCTTA

ATAATCAATTCTCAGTTTCTTCTGATAAAACTTATGAATGGAACATTGATAATTTATCAG

AACAAGAAATCTTAAATAAATTACATCACATGTCTATGGCAGCCAATAGCTATGTCACTA

ATCATAATTTCACCCAACCTGAAATCATTGACATACTTTCCACAGGATTTACAGGAATGC

TTCATTCCTGGTGGGAAAAACATCTCACTCCTGATTCTAGATCATCCATTAAACATGCCA

TTAAACGAGATGAAGATGGGACTCCCATCTTCGACGAACGCACAGGAATGGGAATTTCTG

ATGCTGTTAATACCTTATTTTACACAATTATCCAACATTTCATAGGAACCCCTAGTCACA

TTACTTCCAGAATTCATGATCAACTCAGTAACCTTAGGTGTCCTACCCTAAGTGATTTCA

AATGGTACAAAGATGTTTTCATTTCTCGAGTTATGCTTAGAGACGATAGTAACCAACCAT

TTTGGAAAGAAAAATTCATAAATGGTCTTCCAAAATTATTTGCTCATAAAATCAGACAAG

TCCTTAGTAATGAAACAGGTCACATAAACTATGATTCTTTAACTTATGGAAACATAATTA

CAGTTATCCAAAAAGAAGGATTAAAAATGTGTATAGATATGAAACTAAATGCTCAAATGA

AATCAGATAAGAAAACAGCTAAATACGAATTAGGAAATTTTTGTGAACAATATGGCCTAC

CTCCCTTACCTCCTTCAAGGAGAAAAAAGGCAAGCAATAAATTTATTCCTTACCATTCAA

AAAGAAAATTTACACCTTACAAAAATAACTTTGAGCCAAACAAATTTTATTCCAAAAACA

AATTTCGCAAAAATTGGTCTAAAAAACCAAATCACCAACTAAAAAGTAAACCTTTCGATA

AAAGTAAAACTAAATGTTTTAAATGTAATAAGACTGGTCACTTTGCAAACAATTGTCCAG

TTAAAAGTACTATTAATCAACTCAAAATTAGTCAAGAAGAAAAAGAAAAACTTATACAAA

CTTTAGAATTACGAAACACTGATTCAGAAAATGAAATGAGTGAAGATGACATCACATTGA

TGGAATCTTCTTCCTCTGAATCCAATGAACATTCTTCCCCAGATATTACTTTTGGGTGTA

AGGATGCTTGTAATTGTAAAACAATAAATGTTCTTTCCAAACAGGAAGAACAAGAAGAAC

TATTAATTGATTTAATAGGTAAAATAGATAACCCTGAACTTAAAGCAGAATACCTCAAAA

AATTAAAAAAATTAATTACCCAAGAAAATATAGTTAATCCTCCCTCACAAAAAATTAGCC

TTACTACAACTTTCGATAAATTCTCCACTAGTAAAAAGGAAATAACAATCCAAGATCTAC

AACATGAAGTAAAAACAATCAAGAGTCAAATACATGCTTTACAACAAACACATTTAGAAC

TTACAAATGACAATAAAGAAATTAAACAAGAAATAAACTTACTAAAAATAAATAACCAAT

TTCTTAAATTACAGGATCCCCTTGAAAATCCTTTTCCAAATCCTTTCCCAGAAAATAATG

AAGCTGAAAACTCTGAAAATAATACTATTAGTACCATACGCCAAATCCAGCTTAAAAAAT

GGTACACTACTGTCCAACTCAAAATAAAAGATTTCCAAATAACAATAACAGCCCTTATTG

ACTCTGGTGCAGATCTTAATTGCATCCAAGAAGGCTTAATACCTTCAAAATATTTTGAAA

AAACGAAAGAATCTTTACGATCAGCTAATGGAACAAAAATGGATATTAAATACGAAATCC

CTAAAGCCCATATCTGCCAAAACAATGTTTGTTTCAAAACTTCGTTTGTCTTGGTAAAAA

ATTTAACTGATAAAGTAATTTTAGGACTACCTTTCATAACTTTAATTTACCCTTTCAAAA

CTGAATATGATGGTATTATTACCTACCCTTTTGATGAACCAGTTAAATTTACTTTTCTTT

CAAACCCCGAAATCCATACTTTAAAAGCATTTCAAGAAAATAATATTTCAAAAACTCTCG

GTTGCATTCAAGCAAAAATCAAACAAATAACCTTTCTCCAAGAAGAAATTAATTTCAAAA

GAATTGAAAATCAACTCCAAAACTCTTTTCTTCAGCAAAAGATCAAAACTTTTGAAGAAA

AGATAATCAAAGAAATATGTTCTGATATTCCTACTGCCTTTTGGCACAGGAAAAAACATA

TTGTCTCACTACCCTACATCAAAGAATTCAATGAGAAAAATATCCCTACTAAAGCTCGTC

CAATCCAAATGAGTCACGAAATCATGGATTTTTGTAAAAACGAAATTAATGATTTACTCA

ACAAAGATATTATTCGCCCTAGTAAATCCCCTTGGTCTTGCCCAGCTTTCTATGTTCAGA

AAAATGCTGAACTCGAACGAGGGACTCCTCGTCTAGTTATAAATTACAAACCTCTTAACA

CAGTTTTAGAATGGATCAGGTATCCCATTCCCAATAAACGAGATCTCATTAACAGATTAA

ACAAATCTGTTATCTTTAGTAAATTTGATATGAAATCAGGATTTTGGCAAATTCAAATTA

GTGAAGCCGACAAATACAAAACTGCATTTGTCACGCCCTTTGGTCATTATGAATGGAATG

TTATGCCATTCGGATTAAAAAATGCCCCTAGCGAATTCCAAAACATAATGAATGACATAT

TCAATCCTTATAGTCACTTCTCCATTGTCTACATTGATGATGTTTTAATCTTTTCACAAT

CAATTGATCAACACTGGAAACATCTTCACCAATTCTTTTCCATAATAAAGAATAATGGTC

TAGTTATTTCTGCCTCAAAAATCAAATTATTCCAAACCAACATTCGTTTTCTTGGATTTA

ACATTCACCAACTCCAAATTAAACCTATTGATAGAGCAATACAATTCGCTGACAAATTTC

CAGATGTCATTTTAGATAAAAATCAATTACAAAGATTCCTAGGATCTTTAAATTATGTAG

CAGATTTTTACAAAAACCTTAGGAAACATTGTAAACCTCTATTCGATAGATTACAAAAAA

ATCCTTCCCCTTGGACTTCAGTACATACATCCTTAGTCAAAGAAATTAAGAGTCATGTCA

AAACCTTACCATGCTTAGGAATCCCTCTTCCCGATTCCTATAAAATAGTAGAAACTGATG

CCTCTGAAATAGGTTATGGAGGTATTCTCAAACAACAACTTTCTCCTAATCATCCTGAGC

AAATTGTTCGTTTTCATTCTGGGATTTGGAATCCAGCCCAATCAAATTATAGTACTATTA

AAAAAGAAATTTTATCAATTGTTTTATGTATCAGTAAATTTCAAGATGATTTATTAAATC

AAAAATTTTTAGTCCGCGTCGATTGTAAAAGTGCTAAAGATGTTTTAGAAAAAGATGTTC

AAAACATTGCTAGTAAACAAATCTTTGCCCGTTGGCAAGCAATTTTAAGTATTTTTGATT

TTGAAATTGCATTCATTAAAGGCAGTGACAATCATATCCCAGATTTCCTAACTCGTGAAT

TTCTACAGGGCGCCACAACATGAAGTCTAGCTCATCCAAAGGGAGAAATCCCGGAAAATC

AAAAGCTACGGCATCCTCTCCAGTTAAGCCAGAATCACCTGAGTCAAAATCAAACATAGT

TCCTTTTACAGGTCATTCTCCTGTTGCATTAGTTAACCAGTATAGTGCTCTTGGTAGCAC

TCTCAGTCAAAATAAACCCAGTTACCAATCAGCTTTAGTCAACCAGTTCGACCCTTACAA

ATCAGGCTCCTCATCTAACCCTCCAGTTAATTATAAAAAATCCTCCCCTTACATTATCAG

GGTTGAGCATGGCCTATTTTACATTGAACCCACCTTGAGTCATCTAAAAAATCCTGTTTC

TTTAGTAAAAAGTTATTTTGGTCCAACCTCTCATTACCCTCCAGGTAAACCCCCATTAGC

ATTTTATAAAGACATTCTGTATAAAACCGAGTCAATATTCATTAAACCAATCCTTGATCC

AAAAGACCAATCTAAGATATTATATCATTCCCTTTATATAATCCGAATCATGTCACTAGC

CCAATGGGGTGATCCCCCCTCTACTCCCCACACATTAGACACAATCACATTTACCTATCA

TGATTACATCGAAGCATGGGATAAAATATTCCTCCATCAAACCGAGGATTTTAGTCATTC

GTGGTTCCTCAATTTCGATAGAAGATTCAACCGCCAATTCCCTATGTGGTTTGGTAATTG

GTGGAATGACCATGGTTGCCCTTATGATCTTCTCCCATTACAACTCCAGAATGCAGTCAC

GAAATTTACTACAGTCATGACCTTTTCAGAATATGAGAAAAGGTTTTCTCCCACTCTCCA

ATTCATGGCCAAGTATAAAATTCCTTGGATTATGAAATGGCAATACAAAATTGAACCTGA

AGAATACATGGTTATAAGACAATTCCTAGTAAAATGGTGGGATAAATTCGACCCTGAAAG

AGTTTTAACTCAATTCAGTGAAGAATTCACAGTTATTCCCTCGAGTTCAAAAGCTCCGGT

TTCCATTCAGCCGGCTCCTCCAATCTCGCAGTTAACATCTCCAACTCCTCAGTCACCATC

TTCTTCCAAGAAGACAAATCAGTCACCATCTTCTTCTAAGAAGAAAAAAGCCTCCAGTTC

ATCTTCATCTTCCAAAGCAGAAGAATTAAAAGATCTAGCTAAGCAATTACTACAAGAAGC

AGCAAATTTATCTACAGATGATGAAGAAGAACATTCTCCATCAGAATCATCATCACACAG

TCATCAAAGTTCCCCCAATCAACCAAAAAGATGGGCAGATTATGAAGATAGTCAAGACCC

TTATGCTGAGTTTGATTAATTCCAGTCAAGACCATCTCAGTCAGCTTTTCCACCGAAATC

AACAGTAAATCACATGACAGACAGTCCACTATTCATCAACAGTAAATCACATGACCAACA

GTTACTCAGTGGCAGACAAGTTACTATTCACCAACAGTCACTTTTTACTATTCATCACTT

TTGTAATTTCTATTTATAGAGGCCTCCGGCTTCATTGTGAGGCAGGCTTCATTTTTCTCT

TCTTCTCCTCCCATTCTCTCTCCAGTAAAATACTCATGTAAACAGAGCCCTTGCTCTTAC

ATATGCATTGATTTAAATTTGTAAGTTTGTAACTCTGAGTTCTAATCAATCAAATGTTTT

ACTTCATCTATCAATCAAATGTTTTATTTATTTGCTTCCGTGTTT

>FvesV_sc2 [endogenous-virus-name= Fragaria vesca virus] [strain=sequence cluster 2] [host= Fragaria vesca virus] [moltype=genomic DNA] [note=complete genome] 7681 bp

TGGTATCAGAGCCAAACGGCCTGATATAGTTATTCAGTCAAAAACATCCAACCCCAAGTG

GCGGACTACCGCCTAGGAGTGGTTCTGGAGCTTCATTCAAGCTATATATATTAAACTTCT

CAATCCCCAGGTGGCGGACTACCGCCTAGGAGTGGATCTTGAGTTGATCTATCTTCTTTA

CTTATTCTGTGAAGTTTTGAGTAAACTCCCTTAATTTTAAGGATTTTTGGTTTTGTTTTC

CTTCCTTAGTCTCCTTTGAGCCTGTTATTCTCTCCAACCAAACAATAAGTCCAGGTCCAA

ACACTAAGACCCAGGTGAGGCATGAGCGGATGCGGCATGAGCGGTGAAGAGTGAGTTTTA

GTCTTTAGGATCTTAAGCAAGAATCTTAAATCCTCTTATCCTTTTAGTTTCGGTCCAGTT

TTCTCTTCTTTTCCCAATTATGAGTAGACGATTTAGATCCAACTCAGTCACTTCTAGTTT

GTCTAGGTCCTCCCTCAATAGGATTCCAGATATAGTCAATGAAGAACAAATAGAGTATCA

GTCAAACGATAACATAGATATGAATGACTGGAATATTCCAAGAGTTCCTAGTCATGAAAT

TTATAAGAAAAAATAGAGTCTAGCTTCATTCAAAAGTGAACACCATATTAAATCAGTAGA

ACAAGTTTACGCCCTTAGTAAACAACATGAAACTTGCCAACTTTTAAGTCCAGAATCAAT

TAAGAAACATAAAAAAGATGGTCATAATTTCCTCTATATTGGATTAGTTCAAATTGGTGT

CAAGCCTTTAACTAGAAAAGGTCTTAATGCCTCCATTCTTTTATGTTTAAGAGATGCAAG

GTTTACTGATTTTAATGACAGTACCCTTGGATTAATTGAGTCAAGTCTGTTTAATGGTCC

AGTTCATTTTAATTGTTACCCAGATTTTACCATTAGCCTCAGTGATCCTCATATCCTAAA

AGCTTTAACCCTCAATGTTAAAACTTCAGGTTATCATGTTCTTGAAGGAACCCAACCTTT

AGCTTTAATTTATAGGATCCATTACAAAGTCACTGGTACAAATATGAATTTCCAAGCCAT

TAGTAAAAGTCCCAAAAACCATACTCTCTTAATTCAAAGTAGTCAAGAAAACGTCAATAT

TAGCGTTCCTCACACAATCAGATGGTCAGATGTCCATCTTCCTTCTGAATGGTCTCTCAC

TAGTGAGGATCAACCATCTAATGCACAACATAGTCTTTCTGATTTAGACTGTATTCAACA

GTTTAATGATGGCACAGTAAGAATTAATTTTCAAAACTCTAAGATAAGTCAAAACCCAAG

AATTCAAGAGGTAGGAAACTCCTCTAGACATTCTTTTGCTACGGCTAGACATTCATTTGC

TGGTTCCACTACAGCTGATACAATGTCTAGACAAGATTCTGATCTAGATAAAGAAATTAA

AAATATTAGAATTAGAGAATTAGAAGAAGAACTGCAAAAGCTCAAAATTAAAAACATCAG

AACTACTTCCCAAGTTAGTTACCCAAATTATACAGAAAATGAATCTCAAAATATTTCAAC

CACAGATGAAGAGCAAACTTCTCCCATTGCTTTTGATTTTTTAGTTACTCCTCCACTAAA

TCCTCAATTAAGCATACTTACCCAACTTTTCGTAATCAACTATTCTAGATTAGATAAACA

TCTAGAATCAAAAGAAAATATCCTTAGGAGAAACCTCTATAGGATGAAGTTCCCTTCATG

GATCAACGAAATCAATTATTTCTTGATTGGCAAGCATTTATGCTTGAAACAAGATTTGAA

ATATTCTTTCTTGACTTTATAGATCAACTAAACAACCAAAAAATTCAAACATTAACAAAA

GCAAGATGGAAAACCTCCACTAACTCAGTCTTTTCCAGTCATTCCCCTGTTGAAACAAAT

AATAATTGATCATCTTAACAGTCTAATCACAGCTTCACCTTTTAAAATTGCTATAGAAGA

TCCAGACACTAAGAAAATTAACCCAGATACTAAGAAAATTATTGAACAAAATAATTACAC

AAACCAAAGCCTGGTAACCATCGGAGCCTAGTTAGATAAAATAGAAACAAAGGTTGACAA

CATATCCTCTAAAGTTAATATCCTACCACAACCTAAACCAGAAACTCCTTTAGTTCATTT

TAGTGAATTAAATAAACCACCCTTGAAGCCTACTTCATCGATTCAAAAGATCGAGCAGAT

GTTAGAAAGATTAAAAACTGAAACACTTGAACCCAGTAGGATTGCTGTAATTCATACCCA

ACAAAACTCTTCTTCCATTTCTTCAGATAGCGATTCTAGTGACACTAGCCAAATTAAAGA

AATAAAACAAATACTCCAGACCTTAAATATCGAACCTTCAAATCTAGAACGGATAATTCA

TCCAAAAAAAGACCTTTTAAAACTTGGACAGAACGTCCTCTCCCCTTAGATATACAACCT

ACTAATTACATTACTAATCAATTCTCAGTTTCTTCTGATAAAACTTATCAATGGAATATT

GATAATTTGTCAGAACAAGAAATCTTAAATAAATTACACCACATGTCTATGGCAGCAAAT

AGCTATGTCACTAATCATAATTTCAGCCAACCTGAAATTATTGACATACTTTCCATAGGA

TTTACAGGAATGCTTCATTCCTGGTGGGAAAAACATCTCACTCCTGATTCTAGATCATCC

ATTAAACATGCCATTAAACGAGATGAAGATGGGACTCCCATCTTTGACGATCGCATAGGA

ATGAGAATTTCTGATGCTGTTAATACCTTATTTTACACAATAATCCAACATTTCATAGGA

ACCCCTAGTCACATTACTTCCAGAATTCATGATCAACTCAGTAACCTTAGGTGTCCTACC

CTAAGTGATTTCAAATGGTACAAAGATGTTTTTATTTCTCGAGTTATGCTTAGAGACGAT

AGCAACCAACCATTTTGGAAAGAAAAATTCATAAATGGTCTTCCAAAATTATTCGCTCAC

AAAATCAGGCAAGTCCTTAGTAATGAAACAGGTCACATTAACTATGATTCTTTAACTTAT

GGAAATATAATTACAGTTATCCAAAAAGAAGGATTAAAAATGTGTATAGACATGAAACTA

AATGCTCAAATAAAATCAGATAAGAAAACAGCTAAATATGAATTAGGAAATTTTTGTGAA

CAATATGGCTTACTTCCCTTACCTCCTTCAAGAAGGAAAAAGGCAAGTAATAAATTTATT

CCTTACCATTCAAAAAGAAAATTTACACCTTATAAAAATAACTTTGAGCCAAACAAGTTT

TACTCCAAAAACAAATTTCGAAAAAATTGGTCTAAAAAACCAAACAGCCAACTAAAGAGT

AAATCTTTCGATAAAAGTAAAACTAAATGTTTTAAATGTCATAAGACTGGTCACTTTGCA

AATAATTGTCCAGTTAAAAATACTATTAATCAACTCAAAATCAGTCAAGAAGAAAAAGAG

AAACTTATTCAAACTTTAGAATTATGAAACACTGATTCAGAAAATGAAATGAACGATGAT

GACATCACATTGATGGAATCCTCTTCTTCTGAATCCGATGAACATTCTTCCCCAGACATT

ACTTTTGGGTGTAAGGATGCTTGTAGTTGTAAAACAATCAATGTTCTTTCCAAACAGGAA

GAGCAAGAAGAATTATTAATTGATTTAATAAGGAAAATAGATAATCTTGAGCTTAAAGCA

GAGTATCTTAAAAAATTAAAAAATTAATTACCCAAGAAAACGTAGTTAATCCTCCCTCAC

AAAAAATTAGCCTTACTACAACTTTTGATAAATTTTCTACTAGTAAAAAGGAAATCACAA

TCCAAGATCTACAGCATGAAGTAAAAACAATCAAAAGTCAAATACATGCTTTACAACAAA

CACATTTGGAACTTACAAATGACAATAAAGAAATTAAACAAGAAATAAACTTACTAAAAA

TAAATCATCAATTTCTTAATTTACAGGATCCCCCTGAAAATCATTTTCCAAATCATTTCC

CAGAAAATAATGAAGTTGAAACCTCTGAAAACAATACTATTAGTACAATTCGCCAAATCC

AACTTAAAAAATGGTACACTACTGTCCAACTTAAAATAAAAGATTTCCAAATAACAATAA

CGGCCCTTATCGACTCTGGTGCAGATCTTAACTGCATCTAAGAAGGCTTAATACCTTCGA

AATATTTTGAAAAACAAAAGAGTCTTTACGATCAGCTAACGGAACAAAAATGGATATTAA

ATACGAAATCCCTAAGGCCCATATCTGCCAAAACAATGTTTGTTTCAAAACTTCGTTTGT

CTTGGTAAAAAATCTAACTGATAAAGTTATACTAGGACTACCTTTCATTACTTTAATTTA

CCATTTCAAAACCGAATATGATGGCATTATTACCTACCCTTTTGATGAACCAGTTAAATT

TACTTTTCTTTCAAACCCCGAAATCCATACTCTAAAAGCATTTCAAGAAAATAATATTTC

AAAAACTCTCAGTTGCATTCACGCAAAAAATAAACAAATAACCTTTCTCCAAGAAGAAAT

TAATTTCAAAAGAATTGAAAATCAACTTCAAAACTCTTTTCTTCAGCAAAAGATCAAAAC

TTTTGAAGAAAAGATAATCAAAGAAATATGTTCTGATATTCCTACTGCCTTTTGGCACAG

GAAAAAACATATTGTCTCACTACCTTACATCAAAGAATTCAATAAGAAAAACATCCCTAC

TAAAACTCGTCCAATCCAAATGAGTCACGAGATTATGGATTTTTGTAAAAACGAAATTAA

TGATCTACTAAACAAAGATATTATTCACCCTAGTAAATCTCCTTGGTCTTGCCCAGCTTT

CTATGTTCAGAAAAATGTTGAGCTCGAAAGAGGGACTCCTCGATTAGTTATTAATTATAA

ACCCCTTAACACAGTCTTAGAATGGATTAGGTATCCTATTCCTAATAAACGAGATCTCAT

TAACAGGTTAAATAAATCTGTTATCTTTAGTAAATTTGATATGAAGTCAGGATTTTGGCA

AATTCAAATCAGTGAAGCTGACAAATACAAAACTGCATTTTTCACACCCTTTGGTCATTA

TGAATGGAATGTTATGCCATTTGGATTAAAAAATGCCCCCAGTGAATTCCAAAACATAAT

GAATGACATATTCAATCCTTATAGTCACTTCTCCATCGTCTACATTGATGATGTTTTAAT

TTTTTCACAATCAATTGATCAACACTGGAAACATTTACACAAATTCTTCTCCATAGTAAA

AACTAACGGATTAGTTGTTTCTGCTTCAAAAATAAAATTATTTCAAACCAATATTCGTTT

TCTCGGATTTAACATTCACCAACTCCAAATTAAACCTATTGATCGAGCAATACAATTTGC

TGATAAATTCTCATATGTCATATTAGATAAAAATCAATTACAAAGATTCCTAGGATCCTT

AAATTATATAGCTGATTTTTACAAAAATCTTAGGAAAAAATGCAAGCCCCTATTTGATAG

GTTACAAAAAAACCCTTCTCCCTGGACGTCAATTCATACATCCTTATTTAAAGAAATCAA

GAGTCATGTTAAAGCTCTACCATGCTTAGGAATCCCCCTCTCTGATTCCTATAGGATAGT

CGAAACTGATGCCTCTGAAATAGGTTATGGAAGTATCCTCAAACAACAAATTTCTCCCAA

ACATCCAGAACAAATTGTTCGTTTCCATTCTGGAATTTGGAACCCTGCTCAGTCAAATTA

TAGTACTATTAAAAAAGAAATTTTATCAATTGTTTTATGTATCAGGAAATTTCAAGATGA

TTTATTAAATCAAAAGTTCTTAGTCCGCGTCGATTGTAAAAGTGCTAAAGATGTTTTAGA

AAAAGATGTTCAAAACATTGCTAGTAAGCAAATCTTTGCCCGTTGGCAAGTAATTTTAAG

TATTTTTGATTTTGAAATTGCATTCATTAAAGGCAGTGACAATCATATCCCAGATTTCCT

AACTCGTGAATTTCTACAGGGCGCCACAACATGAAGTCTAGCTCATCCAAAGGAAGAAAT

CCCGGAAAATCAAAAGCTACGGCATCCTCTCCAGTTAAGCCAGAATCACCTGAGTCAAAA

TCAAACATAGTTCCTTTTACAGGTCATTCTCATGTTGCATTAGTTAACCAATATAGTGCT

CTTGGTAGCACTCTCAGTCAAAATATTCCCAGTTACCAATCAGCTTTAGTCAACCAGTTC

GACCCTTACAAATCAGGCTCCTCATCTAACCGTCCAGTTACTTATAAAAAAATCATCCCC

TTACATTGTCAGGACTGAGCATGGCCTATTTTATATTGAGCCTACCATGAGTCATCTAAA

AAAATCATGTTTCTTTAATAAAAAGTTATTTTGGTCCAACCTCTCATTACCCTCCAGGTA

AACCCCCATTAGTATTTTATAAAGACATTCTGTATAAAACCGAGTCAATATTTATTAAAC

CAATCCTTGATCCAAAAGACCAATCTAAGATATTATATCATTCCTTTTATATAATCTGAA

TCATGTCACTAGCCCAATGGGGTGATCCCCCCTCTACTCCCCAGACATTAGATACACTCA

CATTCACCTATATATCATGATTACATCGAAGCATGGGATAAAATATTTCTCCATCAAACA

GAGGATTTTAGTCATTCGTGGTTCCTCAATTTCGATAGAAGATTCAACCGCCAATTCCCT

ATGTGGTTTGGCAATTGGTGGAGTGACCATGGTTGCCTTTATGATCTTCTCCCATTACAA

CTCCAGAATGCAATTACGAAATTTACTACAGTTATGACCTTTTCAGAATATGAGAAAAGG

TTTTCTCCCACTCTTCAATTCATGGCCAAGTATAAAATTCCTTGGATTATGAAATGGCAA

TACAAAATTGAAACTGAAGATTATATGGTCATCAGACAATTCCTAGTAAAATGGTGGGAT

AAATTCGACCCTGAAAGAGTTTTATCTCAATTCAGTGAAGAATTCACAGTTATTCACTCG

AGTTCAAAAACTCCGGATTCCATTCAGCCAGCTCCTCCGATCTCGCAGTTAACATCTCCA

ACTCCTCAGTCACCATCTTCTTCTAAGAAGACAAACCAGTCACCATCTTCTTCTAAGAAG

AAAAAAGTCTCCAGTTCATCTTCATCTTCCAAAGCAGAAGAATTAAAAGATCTGGCTAAG

AAATTACTACAACAAGCAGCAAATTTATCCACAGATGATGAAGAAGAACATTCTCCATCA

GAATCATCTTCATCACACAGTCATCAAAGTTCCCCTAATCAACCAAAAAGATGGGCAGAT

TATGAAGATAGTCAAGACCCTTATGCTGAGTTTGATTAATTCCAGTCAAGACCACCCCAG

TCAGCTTTTCCACCGAAATCAACAGTAAATCACATGGCAGACAAGTTACTATTCACCAAC

AGTCATTTTTTACTATTCATCACTTTTGTAATTTCTATTTATAGAAGCCTCCGGCTTCAT

TGTAAGGCAGGCTTCATTTTTCTCTTCTTCTCCTCCCATTCTCTCTCTAGTAAAATACTC

ATGTAAACAGAGTCTTTGCTCTTACATATGCATTGATTTAAATTAATAAGTTTGTAACTC

TGAGTTCTCATCAATCAAATGTTTTAGTTCATCTATTAATCAAATGTTTTATTTATTTGC

T

>GmaxV_sc1 [endogenous-virus-name=Glycine max A virus] [strain=sequence cluster 1] [host=Glycine max cv. Williams 82] [moltype=genomic DNA] [note=complete genome] 7861 bp

TGGTATCAGAGCCTGATGGGGAAGTAGGAATAGTGTCCGTAAGGGCATATGTTAACTGAA

CTTATGGATCTGAACCATGTACAGTAATTTTAATTTAAACAGTATGTGTTTGAACAGTAT

TTTCTGGAAGCACAGCAGTCTGATATTTTAGTTGAATGATACTGTAGCAGTAAGTCCCGT

GGATGAGAAAGGGCAGTAAGGCTAAGGTGTATTGTTCAAATAAAATATTGAGACATAATA

TACTGGTGGCTATAGGAGTAATGTTCAAACTATATTGTCTGTAAAAAAAAAATCTTCTTT

TTCATTTATTCAGATCCAAGAAGTTCTGTAACATATAGCCTTATGGAAAGATTTCTATGG

AACCAAATGAAGGCAAAGATATTAGAGAATGTTTTAATCATGTCTAGACTATTGCGATCC

TTTTCCCGTATCTCTTTTCGCTCAAATTCCTCCTCATCATCTTCTTCAAGAAACACCAAT

CTATTAAATGATCAAGATAACCAGACCCAAGAGGTCGCCCCAAAACACAATCTCTTCGAT

GAGGAAGTCCGATTCGAAGATATTAATCAAAACATGGATGACTGGAACATTCCAGAAATC

CCTCAGGATCAACTATACGTTCCTGAAACAATTAAAGACAAACACAACTTTGATTACATA

ATCAAAACAGTAGAAAACAATATTCCTCTAGGACAAGATATAGGGGAAGAATTTCATCTA

CTTTCAAAAAATTCAATTTATGAACATAGCCGTAAGTATAAATATCTACACATCGGTTGT

GTACAAGTAGCCATCAAGCCCTTAATTGATATGGGCATAGATGCAGCAGTTTTAATGTGT

CTAAGAGATATTAGGCATAATCAATTTGAAGACTCCCTAATTGGAACAGTCGAAACCAGC

CTAGGACAAGGTCCAATATATTTTAATTGTTATCCAAACAAAACAGTGAGTCTGATGGAC

AGAAACATTCTTGACTCTCTGTTCTTAAACATCCACTTTCATGGCCTTGACATGAAAGAA

GGATCAATCCCAGCAGCCTTAATTTATAGGATCCAGTACAAGGTCATGAATACTTGTGCA

TCTAGAGTCCTGTTAAAACCTCAAAAAGGAGAAACAACTTTGTTTATCACTGATATGACA

AAGGCTAACGTTTCTCTCCCGAGAAAAATAAAATGGGATGAAGTAACTCTTCCCGAAAGA

TGGGTTATGGATAAGGCCACACCGTCTATTCCCCGACCCGCTCCAACCATAGAGCATATT

AAGCAAGATAACTCCGGAAAGGTAGAGATAACCTTCAATAGGAGAAATTCTTTTTCATCA

AGGATAGAAGCCTCTAGGTCTGAATATGAGTCAGCCAGAAGGTCTTTTTCAGTTAGAACC

CGTTCAATTCCAGTAGGACTAACCAGATCTGAGTCACATAACCAGTTCCCTACTGTAAAC

CTCCAAGGATTAGACACTACTTCTAGCATACCTAGAACAACTTACAACCAAGAACAAGAG

GACGACCAAAAGTCGATCCAATCCCCAACATACTCTTCTATGGAACCTTATGATGTTATC

TGACAACTTTAGCATTGATAAGGAAACCCTTAGGAAAGATTTTTATTCTCCGGAGAATGA

ACCTCAAAGGAGATGGTTTTTCCAACATTACAAGGGTACAAACAGAAAACAAATCCAAGA

CAAGTTCTACGAATTTGTCGAAAGAGTCAAAATTAATGTCCTTTTCTTTGATTGGTTTCA

TGCTTATGCCATCAGAAAGGATATAGATTACCCTTGGAAACAAGACATCATAGGTGATCC

AACAACAAATGTCATAACAAATTGGCAAGTGAAGGATGGTGAGTTAATCCAATCGGAATT

ACCTCCCGCAACACAATATCAACTACCAAACATCAAAGACAGTAACAATAAACCTGTCAT

GGCAATTCCATTCAAGACAAAAGATGTCAACGAGGAAGTAACCTCTAAAGACATTAAGAG

CCTAATGGAACAGGCAAATTATACCAACAAATACTTACAAGCTTTAGGAGAAACCATAAA

AACTAAGGTAGTTCCTAAACAAAAATCAATTGAGGAAGCTTCGCCAAGTATCCCCATTGA

AAAACCTTTATTCAAACCTTTCAAAGTTAGTGAGAAGGCTAAACGAAAAATTAGGGAACT

TAGAAAAACTAAATCCTTAATTGAAGGCGTAGGTGACAACCATAGTGAATTACTAAACAA

GATTGGTAGTTTACTTAAAGTCATTCCAGATACTCCCCAAGCCTCGGAAAATACTTCCAA

AATGGTAACAAGAAGTACCTCCAAATTAATTAATGTTATTAATGAAGATAGTGACCAAAA

CTCAGATAACACAACTGAGATAGGATCAGTGTCAGAAAAGAATATAAATCCAATTAATTC

CAAACACTGGAAAACACCCTCCAAATTATATTATCAACGTCCAACTGCCCCTGACCTTCT

ATTAGAAGAAAGAGGTGAAAACAATTTTAAGAGTTTTAGTGCAAACAACATCTATGAATG

GAACATAGATGCACAAACGGAGTATAACATCATGAATACACTCCAACATATGACCATGGT

AGCTACGGCTTACCAAACCTCCCATGAATGTTCAGAAGAGACCATTATAGATATCTTAGT

GGCAGGATTTTCTGGACAACTGAAAGGATGGTGGGATAATTATCTCACTAACGAAGAGAA

ATCAAAAATTTACAGCGCAGTTAAGACGGATCTCAATGGAAAAGTCATTACTAATGACGA

TGATAAAGAGATTCCTGACGCTGTTAACACATTAATTTTTACAATAGCCCAACATTTCAT

TGGAGACCCATCCTTGTGGAAAGATAGGTCTGCAGAATTATTATCAAATCTTAAGTGTAG

AACCTTAGCGGATTTTAGGTGGTATAGAGATACTTTCCTAACTAGGGTTTACACAAGAGA

AGATAGTCAACAACCTTTTTGGAAAGAAAAATTTCTAGCCGGTCTTCCCAGATCATTAGG

AGACAAGGTTAGAGATAAAATCCGTAGTCAATCTGCCAATGGAGATATTCCATATGAAAG

TTTAAGCTATGGTCAATTAATTTCTTATGTCCAAAAGGTAGCCTTAAAAATTTGTCAGGA

TGACAAAATTCAGAGGCAATTAGCCAAAGAAAAGGCTCAAACAAAGAGAGATTTAGGTTC

TTTCTGCGAACAATTTGGTCTACCGGCCTGTCCAAAACAAAAGAAAAAACAATCCTCTAG

AAAAGAAATCCATGAGAATAAACCGGTCAATACAAAGAGATTTCCAAGGAGGAGATACTC

ACACAAACCATCAACCAGTAGAGAAATGGAAAATCCTAAACAAAAAACAAAATCAAAAAT

AACGTGCTACAATTGTGGAAAACAAGGCCACATCAGCAAATACTGTAGACTAAAAAAGAA

GTTAAGAAACCTTAACCTAGAACCCGCAATTGAAGAGCAAATAAACAATTTGCTCATAGA

AACCTCGGAGGAAGAAACAGAAACTTCATCCTCCGTGCTTTCCGATGAAAACCTAAACCT

AATCCAACAAGATGACCAACTATCATCAACAGATGATGATGATGGGCAAATCAATACTCT

AACGAGAGAACAAGATCTCTTGTTTGAAGCAATCAATTCCATACCTGACCCCCAGGAAAA

GAAGGTTTTTCTGGAAAAACTCAAGAAAACATTAGAGGTAAAACCTAGACAAAAGGATTT

TATCACAAACAACAAATTTGATGTAAGTAACATACTCAAGAGATTAGAAAATTCTTCAAC

CAAACCAACGACAATCCAAGATCTCCAAACAGAGATAAACAATCTAAAAAGAGAAGTAAA

AGAACTTCGTCAACAACAAGAAATTCATCAGATCATTCTTTCTCAACTTGAGGAAGATAG

TGATTCTGAAAGTACCAACAACAGTGAGGAAAACCAACCAGAAAATTTAGAAGATGATAT

GTTTATGGGATTAATCAACAAGATTAAAATCCAAAAATTTTACATAAACATAAAAATAAT

TATTAATGATTTCGTTTTAGAAACAATGGCCCTATTTGACACAGGGGCTGATTCAAACTG

CATTTTAGAAGGATTAATTCCTACAAAATTCTTTGAGAAAACCTCAGAAAAATTAAGTAC

GGCAAACGGCTCAAAATTAAAAATTAATTTTAAGCTATCAAATGCAATCATTGAAAACCA

AGGTCTTAAGATTAACACAAACTTTCTTCTGGTAAAAAACCTTAAGAATGAAGTAATCTT

AGGAACGCCCTTCATTAGAGCCCTGTTTCCCATCCAAATATCTAATGAAGGAATAACCAC

AAATTATTTAGGAAGAAAAATTATATTTAATTTTTCTACTAAACCAATTTCCAGAAATAT

AAATCTTATAGAAAATAAGATTAATCAAATTAACTTCTTAAAAGAAGAAGTATCTTTTAA

TAATATTCAGATACAACTGGGAAAACCCCAAGTAAAAGAAAGAATTCAATCTTTATTAAA

TCATATTGAATCGACCGTTTGTTCTGAATTGCCACATGCATTTTGGGACAGAAAGAAACA

TATTGTCGATCTCCCTTATGAGAAAGACTTTAGGGAAAAACAAATTCCCACAAAAGCCAG

ACCAATCCAAATGAATGAAGAACTTCTTCAATACTGTCAAAAGGAAATCAAGGATTTGCT

TGATAAAGGCCTAATCCGGAAAAGCAAAAGCCCATGGTCTTGTGCGGCTTTTTATGTTAA

CAAACAATCTGAGATTGAGCGTGGAACACCCCGCTTAGTCATAAATTACAAACCACTAAA

TCAAGCATTACAATGGATTAGATATCCTATTCCAAACAAAAAAGATTTGCTTAACAGATT

AAATTCTGCAAAGATATTTTCAAAATTTGATATGAAATCTGGATTTTGGCAAATCCAAAT

CCAAGAATCAGATAGGTACAAAACAGCGTTTACTGTACCTTTCGGGCAATATGAATGGAA

CGTGATGCCATTCGGACTGAAGAATGCCCCTTCAGAATTTCAAAAAATTATGAATGATAT

TTTTAATCCTTATTCAAAATTTGTCATTGTCTACATAGATGATGTTTTAATCTTTTCCCA

AAACATTGACCAACATTTCAAACATCTCCAAACCTTCATACATATCATCAAACAAAATGG

TTTGGCAGTCTCCAAATCAAAAATCAATCTTTTCCAAACTCGAATTCGATTCCTTGGTCA

CAATATATATCAAGGTACAATCATTCCAATCGAAAGGTCAATAGAGTTTGCTAGCAAATT

CCCCAACCAAATCTTAGACAAAACCCAGTTACAAAGATTTCTAGGTTGTCTAAATTACGT

AGGAGAATTTGTCCCCTATTTAAATAATATTGTCAAGCCATTACATGATAGGCTGAAGAA

AAATCCGCCTCCTTGGTCTGACCTCCATACACAAACAGTTAAAGAAATCAAAGTCAAAGT

CCAATCCATTAAATGTCTGTATCTTCCTATTCCACAGGCATTTAAAATTGTCGAAACTGA

TGCTTCAGACATAGGTTATGGTGGCATCCTCAAACAAAGAGTCCATGGCCAAGATCATGT

CATTGCATATACATCAAAACATTGGAATCCTGCACAATTAAAATATTCAACTGTTAAAAA

AGAAGTTTTAGCAATTGTTTTGTGCATTTCCAAATTTCAATCGGATTTATTAAACCAAAA

ATTTTTAATCAGGGTTGATTGTAAATCAGCCAAAGATATTTTACAAAAAGATGTTAAAAA

CCTTGCCTCAAAACAAATTTTTGCACGATGGCAAGCAATTTTAAGTGTCTTTGATTTTGA

CATAGAATACATCAAGGGCACCTCAAATTCACTACCTGACTACCTTACCCGTGAATTCTT

ACAGAAAAGTGGTAATGCCTCCTAAGGCATCGGGAATCTCCCTACGGGGAGGAAGGAGTA

CAGGCAAAGGAGCCAGGCTGGCTCTTCCAGAGCCAACCATCAAGAAATCATCTACCTCTT

CTGGGTCACCAACCCAGGCTGGGTCATCTACCCAGAAAACCAATTTAGAAGGGTCATCAA

CCCAGATTGCCTCAATCAAACCTGAGCCATCCACTCAGGAATCTCCCAAACCGGCAACCG

CCAAACAAACATCGGCCGACTATGCATGGCCAATTGAAACCCTTCAGGCCCTTCAAGACA

TGGGTCTGACCAAATTTCCAAAAATAATCAAAAAATCTTGGGCAGACATTGCCTCAGAAT

CTGATGATGAATCTGAATCAAATTTACAAACAATGATCCAAAATGCGTCAATGACCAAAA

CAGCCACCAATACTAAAGGAAAACTACCTTTAGCCAAAGCTCAAACACCACCTACCAAAC

AAACTAACAATTATATTTATAAAAATAAATTGTTAACTGTTTTGCAGATGGAACCTGAAT

TTTGGGACAAAAATCCCTTCAAGGCGACGGCCAAGGCTTTCCCACCAGGATTTCACTACA

AACCAACCACCATCCTGAAGACAAGAACTTTTTATGAATTGATTTTGGTAGATTCAAACT

CAGTATCTATCAAACATTTCAAAGATCCAAAAGACCAAACCTTAAACACCCACTCAACCA

TCCAAATACTAAAAGTTCTTCAACCAAGACACTTTGGTTCGGACTTGAACAAAGGAAAGA

GATTTTCAGTTCCATTTGATCCGGTAGGTTATACCTATTGGGATTACGTTGATGCTTGGA

CTAAGGTGTTTTGGCACCAAAATACCCGCTTCAAGCATTCATGGCTGATATATTTCAAGA

CCAACACAATTTACAATTTTCCCAATTGGTTTTTGCAATGGTGGGACTTCTTTGGACCAA

TCCCAGAGATATTCCCAGAAACGGTCCAACAAGGATTTTCCCAATTTAAGAAACAATACA

ATTCGCAGGAATCACGAATTCCAGCAGATCTTAAATACTTTTCAAGCTTTGCACTGTCAT

GGATATTCTCATGGCAATATCGTTACAGCAAGACTGAAAAGACAAACCAATATCCATCAC

TACAAAGACACGCATTTGTAAAATGGTGGACACAATTTGACTCATCCAAAGCGGATCCAG

AACAAGTAAAACTATGGTTCCAATCCCATCCAGAATTCCTCAAAGCAGCTGATCCAGAGA

CTTCTGTGTTTTTGAACCAAAAGTCTCATCTGGCAGCATTTTTAGCAGGATCGACATCAA

AGGAGGTCTTAGCTAAAAATCTGAAGGAAGTTCTACAAATGTTACAACAGGAAGAAGAAG

CTTCATCCTCAAAGAAGGAAGAAACAAGTTCTGCTGAAGAAGACGAAGAAGATCCCTTTT

ACCAAAACGAAGATGATTGTTTTGGTATCTGTTTAGATTAAATTTAATTTCGGTCACAAA

ACCGTCTGTTTGTAATTAATGTGTAATTAATGCTGGACAATACTACCTGTCCTGTAGCCA

TTAAAGTCCCGGTCAAAACTACTTGTCTTGTAACAAACAGTCAAATAGTCCCGGTCAAAA

CTACCGGCAACTACCGGTCTTAGATTTTTGGCTATATAAGGAGTCCTCAAGTATGTTGTG

AGGCACCCTTTCATTTAGCCATTTTTCAGGTTTCGGAAGAGAGGTGCTCTGAAACAAGAT

TTTTGTAAGCGCTTCTTTCTAGATTCAATAAATTTCTATTTTTCATCCTTCTCTCTTCTC

CCTTCACCGATCCTGTGCGGCTCTGCCGCCGCTTCGGTTTCTTTGTTTATGAAAACCTGG

GTTTTGTAAGCCGGTCTCCGGTGTGCCCTTGATATCTGTTTTCAAGTTTATATTTTCATT

TCATTAATCATACTGCATTCTTACTTCCTATCTGTCCGTTTGATCCTACTCCGCTGACCT

C

>GmaxV_sc2 [endogenous-virus-name=Glycine max A virus] [strain=sequence cluster 2] [host=Glycine max cv. Williams 82] [moltype=genomic DNA] [note=complete genome] 7860 bp

TGGTATCAGAGCCTGATGGGGAAGTAGGAATACTGTCCGTAAGGACGTATGTTAACAAAT

TTTATGGATCTGACACCTTGTATAGTATGAGTCATATTTTATTTTAATTTGTACAGTAGA

TGTTTGAACAGTACTTCCTGGAAGTACAACAGTCTAATATTTTAGTTGAATGGTACTGTA

GCAGTAAGACCCGTGGTGGATAAAGGGCAGTAAGGCTAAGGTGTACTGTTTAACCAAAAT

ATCAAGACATAATATACTGGTGGCTATAGGATTGCCGTTCAAACTATTTTTAAAAAAAAA

GAGTTTTTCTTAAAAATAATTCAGATCCGAAAAATTTTGTTACATATTTACTTACGGAAA

TATTTCTATGGAACCAAATGAGGGCGAAGATATTAGAGGATTTTATAATCATGTCTAGAC

TACTGCGTTCTTTTTCGCGTATTTCCTTTCGGTCTAATTCCTCTTCATCAACTTCTTCCA

GAAATTCAAACTTATTAAATGATCAAGATAACCAGACCCAAGAGGTCGCCCCTAAACACA

ATCTCTTCGATGAGGAAGTCCGATTCGAAGATATCAATCAAAACATGGATGACTGGAACA

TCCCAGAAATCCCTCAGGATCAACTATACGTTCCTGAAACAATTAAAGATAAACACAACT

TTGATTACATAATCAAAACAGTAGAAAACAATATTCCTCTAGGGCAAGACATAGGGGAAG

AATTCCATTTACTTTCAAAAAATTCAATTTATGAACATAGTCGAAAGTATAAATATTTAC

ACATCGGCTGTGTACAAGTAGCCATCAAGCCCTTAATAGACATGGGCATAGATGCAGCAG

TGTTAATGTGTCTAAGAGATATTAGGCATAATAAATTTGAAGACTCCTTAATTGGAACAG

TCGAAACCAGCCTAGGACAAGGTCCAGTATATTTTAATTGTTATCCAAACAAAACAGTGA

GTCTGATGGACAGAAACATTCTCGACTCTCTGTTCTTAAACATCCACTTTCATGGCCTTG

ACATGAAAGAAGGATCAATCCCAGCGGCTTTAATCTATAGGATCCAGTACAAGGTCATGA

ACACTTGTGCCTCTAGAGTCCTGTTGCAACCACAAAAGGGAGAAACAACCTTGTTTATTA

CCGATATGACAAAAGCCAACGTCTCTTTAAAAAGAATAATAAAATGGGATGAAGTAACTC

TCCCCGAAAAATGGGTCATGGAGAAGGCCACACCGTCTGATCCCCGACCCGCTCCAATTA

TTGAAGACATTAAACAGGATAACTCCGGAAAGGTAGAAATAACCTTCAATAGGAGAAATT

CCTTCTCATCAAGGATAGAAGCCTCTAGGTCTGAATATGAGTCAGCCAGAAGGTCTTTTT

CGGTTAGAACCCGTTCAATCCCAGTAGGACTAACCAGATCTGAGTTACATAACCAGTTCC

CTACTGTAAACCTCCAAGGACTAGATACCACTTCTAGCATACCAAGAACAACCTACAACC

AAGATCAGGAGGACGACCAAAAGTCGATCCAATCCCCAACATACTCTTCTATGGAACCTT

ATGATGTTATCTGACAACTTTAGCATTGATAAGGAAACCCTTAGGAAAGATTTTTATTCT

CCGGCGAATGAGCCTCAAAGGAGATGGTTTTTCCAGAATTACAAGGGTACAAACAGAAAA

CAGATCCAAGACAAGTTCTACGAATTTGTCGAAAGGGTCAAAGTTAATGTCCTTTTCTTT

GATTGGTTCCATGCTTACACCATCAAAAAAGATATAGATTACCCATGGAAGCAAGACATC

ATAGGTGATCCATCAACAAATGTTATAACCAACTGGCAAGTAAGAGATGGTGAGATAATC

CAATCGGAATTACCTCCCACGACCAAATATCAACTCCCGAATATCAAAGATAGTAACAAC

AAACCTGTAATGGCAACCCCATTTAAAACAAAAGATATTAATGAGGAAGTAACCTCTGAA

GATATTAAAAGCCTAATGGAACAGGCAAACTACACCAATAAATATTTACAAATTTTAGGA

GAAACCATTAAAACTAAGGTAGTTCCTAAGCAAAAATCGGTTGAAGAAGCCTCTCCAAGT

ATTCCCATTGAAAAACCACTGTTCAAACCTTTCAAAGTTAGTGAGAAAGCTAAACGAAAG

ATTAGAGAACTTAGGAAAACTAAATCCTTAGTTGAAGGAGTAGGTGACAATCATAGTGAA

TTACTAAACAAGATTGGTAGTTTACTTAAGGTCATTCCGGAAACTCCCCAAACCTCGGAA

AATACTTCCAAAATGGTAACAAGAAGTACCTCCAAATTAATTAATGTTATTAATGAAGAT

AGTGACCAAAACTCAGATAACACAACTGAGATAGGATCAGTGTCAGAAAAGAATATAAAT

CCAATTAATTCCAAACATTGGAAAACACCTTCCAAATTATATTATCAACGTCCAACTGCC

CCTGACCTTCTATTAGAAGAAAGAGGAGAAAACAATTTCAAGAGTTTTAGTGCAAACAAC

ATCTATGAATGGAACATAGATGCACAAACGGAGTATAATATCATGAATACACTCCAACAT

ATGACCATGGTAGCTACGGCTTACCAAACCTCCCACGAATGTTCAGAAGAGACCATTATA

GACATCTTAGTGGCAGGATTTTCTGGACAACTGAAAGGATGGTGGGATAATTATCTCACT

AATGAGGAGAAATCAAAAATTTACAGCGCAGTCAAAACGGATCTCAACGGAAAAGTCATT

ACTAATGACGATGATAAAGAGATTCCTGACGCTGTCAATACCTTAATTTTTACAATAGCT

CAACATTTTATTGGAGACCCATCCTTATGGAAAGATAGGTCTGCAGAATTATTATCAAAC

CTTAAGTGTAGAACCTTAGCAGATTTTAGGTGGTACAGAGATACTTTCCTAACTAGGGTT

TACACCAGAGAAGATAGTCAACAACCATTCTGGAAAGAAAAATTTCTAGCTGGTCTTCCC

AGATCATTAGGAGACAAGGTTAGGGATAAAATTCGTAGTCAATCTGCCAATGGAGACATT

CCATATGAAAACTTAAGCTATGGTCAATTAATTTCCTATGTCCAAAAGGTAGCCTTAAAA

ATCTGCCAAGATGACAAAATTCAGAGGCAATTAGCCAAAGAAAAAGCTCAAACAAAGAGG

GATTTAGGTTCCTTCTGTGAACAATTTGGCTTACCAGCCTGCCCAAAACAAAAGAAGAAA

CAAACCTCTAGGAAAGAAATCCAGGATCATAAACCAGCCAATAGAAGAAGATTTTCAAAA

AGAAGATACTCTCAAAAACCCTCAACTAGTAAGGAAATAGAAAATCCTAAGCAAAAAATA

AAATCAAAAATAACATGCTACAATTGTGGAAAACAAGGCCACATTAGTAAGTATTGTAGA

TTAAAGAAAAAATTAAGAAATCTTAACCTAGAACCCACAATCGAAGAACAAATAAACAAT

TTACTCATAGAAACTTCGGAGGAAGAAACAGAAACTGAAACTTCATCCTCCGTACTTTCC

GACGAAAATCTAAACTTAATCCAACAAGATGACCAATTATCATCAACGGATGATGATGAT

AAGCAAATCAATACTCTAACAAGAGAACAAGATCTCTTGTTTGAAGCAATTAATTCCATC

CCTGACCCCCAGGAAAAGAAGGTTTTTCTGGAAAAACTCAAGAAGACCTTAGAAGTCAAA

CCTAGACAAAAAGATTTTATTACAAACAACAAGTTTGATGTAAGTAACATACTCAAGAGA

TTAGAAAACTCTTCAACCAAACCAACGACTATCCAAGATCTCCAAACAGAAATAAACAAT

CTAAAAAGAGAAGTAAAAGAACTTCGTCAACAACAAGAAATTCATCAGATCATTCTTTCT

CAACTTGAGGAAGAAAGTGATTCTGAAAACACCAACAGTGAAGAAAATCAACCAGAAAAT

CTAGAAGACGATATGTTTATGGGATTAATCAACAAGATTAAAATCCAAAAATTTTACATA

AACATAAAAATAATTATTAATGATTTTGTTCTAGAAACAATGGCCCTATTCGACACAGGG

GCTGACTCAAACTGTATTTTAGAAGGATTAATTCCTACAAAATTCTTTGAGAAAACCTCG

GAGAAACTTAGTACAGCAAACGGCTCAAAATTAAAAATTAACTTTAAGTTATCAAATGCA

ATTATTGAAAACCAAGGTCTTAGGATTAACACAAACTTTCTTCTGGTAAAAAACCTTAAG

AACGAAGTAATCCTAGGAACTCCCTTCATAAGAGCTTTATTTCCCATCCAAATATCTAAT

GAAGGAATAACTACAAGTTATTTAGGAAGGAAAATTATATTTAATTTTTCTACTAAACCA

ATCTCCAGAAATATAAATCTTATAGAAAATAAGATTAATCAAATTAACTTCTTAAAAGAA

GAAGTATCGTTTAATAATATCCAAATACAACTAGGAAAACCCCAAATAAAGGAGAAAATC

CAATCTTTATTAAGTCATATAGAATCAACTGTTTGTTCTGAATTGCCACATGCATTTTGG

GACAGAAAGAAACATATTGTTGATCTTCCTTATGAGAAAGATTTTAGGGAAAAACAAATT

CCCACAAAAGCTAGACCAATCCAAATGAATGAAGAACTCCTTCAATATTGCCAAAAAGAA

ATTAAAGATTTACTTGATAAAGGTTTAATTCGGAAAAGTAAAAGTCCATGGTCTTGTGCG

GCTTTTTACGTCAACAAACAATCCGAGATTGAGCGAGGAACACCCCGCTTAGTCATAAAT

TACAAACCGTTAAATCAAGCATTACAATGGATCAGGTATCCTATTCCAAATAAAAAAGAC

CTACTTAACAGATTAAATTCTGCAAAGGTATTTTCAAAATTTGACATGAAATCCGGATTC

TGGCAAATCCAAATCCAAGAATCAGATAGGTACAAAACAGCGTTTACTGTACCTTTCGGG

CAATATGAGTGGAATGTAATGCCATTCGGACTGAAGAATGCCCCTTCAGAATTTCAAAAA

ATCATGAATGATATTTTCAATCCTTATTCAAAATTCGTCATTGTTTACATTGATGATGTT

TTAATCTTCTCCCAAGATATTGATCAACATTTCAAACACCTCCAGACTTTCATCCATATC

ATTAAACAGAATGGTTTGGCAGTCTCCAAATCAAAGATAAATCTTTTCCAGACACGGATA

CGATTCCTTGGGCACAATATATACCAAGGCACAATCATTCCAATAGAAAGATCAATAGAG

TTTGCCAGCAAATTCCCTAACCAAATCTTAGACAAGACCCAGTTACAACGATTTCTGGGG

TGCCTAAATTATGTAGGAGAATTTGTCCCCTATCTAAACAATATTGTCAAACCATTACAT

GATAGGCTGAAAAAGAATCCGCCTCCTTGGTCTGACCTCCATACTCAAACAGTCAAAGAA

ATCAAAGTCAAAGTCCAATCCATAAAATGTCTGTATCTTCCCATTCCACAGGCATTCAAA

ATTGTCGAAACTGATGCATCAGACATCGGTTACGGTGGTATCCTCAAACAGCAAATACAT

GGTCAAGAACATGTCATTGCATATACTTCAAAACATTGGAATCCTGCACAATTAAAATAT

TCAACTGTTAAAAAGGAAGTTTTAGCAATTGTTTTGTGCATTTCCAAATTTCAATCTGAT

TTATTGAATCAAAAATTTTTAGTAAGGGTTGACTGCAAATCAGCCAAAGACATTTTACAA

AAGGATGTAAAAAACCTTGCCTCAAAACAAATTTTTGCAAGATGGCAAGCAATTTTAAGC

GTCTTTGATTTTAACATAGAATATATCAAGGGCTCTTCAAACTCTCTACCTGACTACCTC

ACCCGTGAATTTTTACAGAAAATTCCCGATGCCTCCTAAGGTGTCTAGCTCCTCCGGCAG

AGGAGGAAGATCCAGTACTAAAGGAAAAGATGTTATATTGGCTCTACCAGAGCCAATAAC

CAAGAAGGCAACTACATCATCTTCTGGGTCACCTACTCAGACTGGGTCATCAACCCAGAA

AGGAAAGCTTGAAGGGTCATTGACCCAGATAACTACCATAAAACCTGAGTCATCAACTCA

GGAATCCCCAAAACCAACAACAACAAAACAGACTGTGGCTGACTATGCCTGGTCAATACA

AACTCTCCTGGCCTTAGAAGACATAGGCCTCACCAGAGTACCAAAATTGGCCAAAAAGAC

CTGGGCAGAAATGGCCTCAGAATCAGACGATGATTCTGAAACAGACCTACAAAAACAAAT

CCAAAAGGCCAAACAGACCAAAACTGTCTGTAACCAAAAACCAAGCCAACCGTTGACTCA

ACAAGAATCAACACCACAACCCAGCAATAGCTATATTTCAAAAAACAAATTCTTCAATGT

TTTACAAATGGAACCAGAATACTGGGACAAGAATCCTTTCAAGGCAACCGCCAAAGTATT

CCCCCCAGGATTCCATTATAGGCCAACAGCCACAAATAAAACCAGAAAATTCTACGAATT

CATTCTAATAGATACAAACTCAGTATCTATTAAACACTTCAAAGACCCCAAAGATCCAAA

TCTAAATACCCATTCCACAATCCAAATCCTAAAGGTTATGCAGCCACGACATTATGGCTC

AAACTTAAACCAACCCAAAAAATTTTCTGCACCTTTTGATCCTGCAGGATATAATTATTG

GGACTACATTGACGCCTGGACCAATGTGTTCTGGCATCAAAATAGTAAATTTAAGCATTC

ATGGCTAATTTACTTCAAGAATAACACTGTGTATAATTTTCCAAATTGGTTCCTCCAATG

GTGGAACTACTTCGGACCAATTCCACAGATATTTCCAGAAGAAGTCCAACAGGGATTCCA

ACAATTCACCAAGCTCTTCAATAGCAAGGAATCAAGAATTCCGGCAGATTTAAAGTACTT

TTCCAGTTTTGCCTTGTCATGGATTTTTTCATGGCAATACAGATATGGAAAAACCGAAAA

CAGCAAACAATATCCACCATTGCAGAGACATGCCTTTGTAAAGTGGTGGTCACAATTCGA

TACCTCAAAGGCTGCTCCAGAACAAGTCAAGAACTGGTTTCAGAGCAACCCTGAGTTTCT

CAAGCCTGCTGATCCAGAGACATCTTTGTTTCTGAATCAAAAGTCTCAACTTGCAGCATT

CTTAGCAAGCTCAAAATCTAAAGAGAGTCTTGCTCAGAATCTTAAGGAAGTTCTCCAACT

ACTTCAACAGGAAGAAGAAGAAGAACCCCCCAAGAAGGAAGCCGAATCATCCGAGAATAA

TGATGACCAAGAAGATGATCCATTCTACCAGAATGAGGATGACTGTTTTGGTATATCGTT

AGACGATGATTAGTTAGTTGTAGTAATCATGAATTATTGTAACTAATTATTCGGTTCCCG

ATAATAATGTAATTTGAAAAAGTTTTTGTAACAGTTCCGGTAAACATTACCGATACTGTT

GAAACAGTAAAACATTGGGTCTATTTAAAGGAGTCCTCGTTCCCTTTGTGAGGCACCCTT

TCAGATAGTCTCAAAACCAGGTTTTAGAGAGAGAAGCTCTGCCTAGGGAAAACCTCTTTG

TAAGCTTTTCTTTCGATTTCAATAAATTCAAAGTTCTATTTTTCACATCTCCCTTCTCCC

TTCACCGATCCTGTGCGGCTCTGCCGCCGCTTCGGTTTCTTTGTTTTCAAAAACCTGGGT

TTGTAAGCCGGATTCCGGTGTGCCCTTGATAACTGTTTTACAAGTTTTAAATTTCTGCAT

TTGTCATATTGCATTACTCTTACTTCCTATCTGTCCGTTTGTATCCTTTCCGCTGATCTC

>GmaxV_sc3 [endogenous-virus-name=Glycine max A virus] [strain=sequence cluster 3] [host=Glycine max cv. Williams 82] [moltype=genomic DNA] [note=incomplete at 3’ end] 7295 bp

TGGTATCAGAGCCAGATGGGGAAGTAGGAATATAGTCCGTAAGGAATATATGTATACTGA

TTTCATTGATCTGGAAGTGTGAACAATAAATGTTTTGTATAGTATCTTTAATCTAAACAA

TATGTGTTTGAATAGTATTCCTGGAAGTACAACAGTCTGATATTTTAGTTGAATGATACT

GTAACAATAAGTCCCATGGATGAGAAAGGGCACTAAGGCTAAGGTGTATTATTTAAATAA

AATATTAAGACATAATATACTGGTGACTATGGGAGTAAGATTCAAACCATATTGAAAAAA

AAAAGTTAATTTTTTTTTCCAGATCCAGGAAATTTAGGAACATATAACCTTATGGAAACA

TTCCTATGGAACCAAATGAAGGCAAAGATAATAGAGGATGAAAATTAGAATGTCTTGCCT

TTTACAATCATTTTCCCAAATCTCCTTTCGCACCAGCTCATCATCATCATCCTCAACCTC

AGACCCAAGAAACTTAAATCTGCTCACTGAGCAAGACAATCAGACCCACGAGGTCACACC

TAAACATAACCTCTTCGATGAAGAAGTACGCTTCGAGGAGATAAACCAAAACATGGATGA

CTGGAGCATTCCAGAAATCCCTCAGGATACATTATATGTTCCTAAGACAACTAAAAACAA

ACACAATTTTGACTACATAATTAAGACTGTAGAAAATAAAATTCCTCTAAGACAAGATAT

AGGGGAAGAATTCCATTTATTATAAAAAAACTCAACCTTTGAACATAGCAAAAAATACAA

ATACCTACACATAAGATGTGTCCAGGTGGCAATCAAACCATTAATTGACATGGGCATAAA

CGCCACAGTCTTGATGTGCCTTAGAGACATTAGACACAACAAATTTGAAGATTCATTAAT

AGGAACAGTAGAAACTAGCATAGGACAAGGACCAGTCTATTTCAACTGTTATCCAAAAAA

AACGGTGAGTCTCATGGATAAAAACATCCTTGACTCCCTATTCCTTAACATCCACTTTCA

TGGCCTAGACATGAAAGAGGGTTCAATTCCAGCAACCTTGATCAACAGAATCCAATACAA

GGTCATGAACACTTGAGCATCAAGAGTTCTGCTCAAACCACAAAAAGGAGAGACAACTTT

GTTTATCACTAATATGACAAAGGCCAATGTTTCTCTCCCAAGAACCATAAAATGGGATGA

GGCAACTCTTCCCAAAAAATGGGTAATAGACAAGGCAACACCTTCAATCCCCAGACCTGC

CCCCACCATAGAACAAATCAAACAAGATAATTCTGGTAAGGTAGAAATAACCTTCAATAA

GAGAAACTCATTCTCATCCAGAATAGAAGCCTCAAGACAATCTGAGTATGAATCAGCAAG

AAGGTCCTTCTCAGTAAGGACCCAATCAATTCCAGTAGGATTATCTAGATCTAAGTCACA

CAACCAGTTTTCTAACATAAACCTCCAAGGATTAGATACAACTTCTAGTATACCTAGAAC

CAAATACAACCAGGAACAAGAAGATGACCAAAAGTAAATCCAATCTCCAACATACTCTTC

TATCCATGAACCTTATGATGTTATCTGACGAATTTAACATTGATAAGGAAACCCTTAGAA

AAGACTTTTATTCTCCAGAGAATGAACCTAAAAGGAGATGGTTTTTTCAGCATTTTAAAG

GGTTAAAAAGACAACAAATCCAAGACAAATTTTATAAATTTGTCGAAAAAATCAAAACCA

ATATCCCTTTCTTTGATTGGTTTCATGCGTATACCATAAAAGAAAATATAAATTACCCTT

GGAAACAAGACATAATAGGTGATCCAACAACCAATGTCATTACCAACTGGCAAATCAAAG

ATGGTGAGTTAATCCAATCAGAATTACCTCCCACAACACAATACCAACTACCAAAGGTCA

AGGACAGCAGTGATAAACCTGTCATGGCCACACCGTTTAAAACAAAAGATGTCAACGAAG

AAATAACCCCAAAAGACATCAGAAGCCTAATGGAACAGACTAACTACACCAACAAATACC

TTCAGGTGATAGGAGAAACCATTAGTAAAGAAAAAATCTCTACTAAACCCAAGGACCACG

AATGGCCAACATCAAACGTTCAAATAGAAAAACCTTTGTTCAAACCTTTTAAGGTTAACA

AAAAAGCAAAACAAAAATTTACAAAACTTAGAAAAAAAAAATCCCCTACAAAGGAGATAA

GTGATAACAATAGTGAATTATTAAGAAAGATTAATAGTTTACTTAAAACCATCCCTGAAA

CCCCTCAACCATAAGAAGAATCATCCAAAATAAGAACTAGACACACCTCCAAACTAATTA

ATGCCATTAACAAGGATACTGACAAAAACTCAGAACAAGTAACTGAAGAAGGATCAATAT

CTGAAAAAGAAATCAATCCAATTAACACCAAACATTGGAAAACACCTTCCAAACTTTATT

ACCAAAGACCAACCGCCCCTGATAATCTACTAGAAGAAAGAGACAAAAATAACTTTAAAA

GTTTTAGTGCAAACAACATCTATGAATGGAATATAGATGCTCAAATAGAATACAACATCA

TGAATACACACCAACACATGACCATGGTAGCCACAACATACCAAACATCCCATGAGTGTA

CAAAAGAAACAATTGTAGACATCCTGGTAGCAAGATTTTATGGCCAATTAAAAGGATGGT

GGGATAATTATCTCACTAATGAGGAAAAATAAAAAAATATACAGCGCTATTAAAATGGAT

CTCAATGGAAAAGTCATTACAAATGACGATAACGAAGAGATCCCTGACGCTATTAATACC

CTCAATTTTACAAAAGCCAAACATTTTATCGGTGTGACCCATCACTTTGGAAAGATAGGT

CAGCAGAATTACTATCCAACCTTAAGTGTAGAACTTTAGTAGCAGACTTTAGATGGTACA

GAGACACCTTCCTAACTAGAGTTTACACGAGAGAAGATAGCCAACAATCTTTCTGGAAAG

AAAAATTTCTAGCCGGTCTTCCAAGATCATTAGGAGATAAGATTAGAGACAAAATCCGTA

GCCAATCCACCAATGAAGACATTCCATATGACAACGTAAGTTATGGACAACTAATTTCTT

ATATCCAAAAAGTAGCCCTAAAAATTTGCCAAGATGACAAAATCCAGAGACAACTGGCCA

AGGAAAAAGCCCAAAATAAAAGAGACTTAGGATCTTTCTGCGAAAAATTTGGATTACCAG

CCTGTCCAAAACAAAAGAAAAAACAAAACCCCAAAAAAAAATTTCATGAAAACAAAAACG

CCAATAAAAGAAGATTTCCAAATAAAAAATAATCAGACAAACCCTCAACCAACAAAGAAA

CCAATATGCCAAAAACGGTTACAAAACCCAAAACCAAAGTAGTTTGCTACAATTGTGGAA

AACAAGGCCACATAAGCAAATATTGTAGATTAAAAAAGAAACTAAGAAACCTCAACCTAG

ACCCATCCATAGAAGAGCAAATTAATAATCTGCTCATTGAAACCTCAGAAGAAGAAACTG

GGGAAGCATCCTCAGAAGAAAATCTCAACCAAATCCAACAGGATGATCAAACATCATCCA

CGGAAGAACAAGATACCCAAGAAATCAAAACTTTAACAAAAGAACAGGATCTTTTTGTTT

GAAGCAATAAATTCCATACCAAACCCTCAGGAAAAGAAGGTTTTCCTGAATAAACTTAAA

AAATCTTTGGAAAACAAACCTAAACCAAAAGATTTTATTACCAACAACAAATTTGATGTT

AGTAATATCATCAAAAGATTAGAAAACACTTATGTCAAACCAACCACAATCCAAGATCTC

CAAACAAAGATTAACAACCTTAAACAAGAAATTAAAGAACTCAAACAACAACAAGAAATT

CATCAAATCATCCTTTCTCATTTCGAAGAACAAAGTGATTCAGAAAATGAGGAAAAAGAA

GAAAAAGATGAATGTCAAGATGATGAAATGTTCATGGGATTAATCAACAAGATTAAAATC

CAAAAGTTTTATATTAATATTAGAATCATTATAAATGATTTTGTTTTAGATACAATGACT

CTATTTGACACAAGAGCTAATTCAAACTGTATTTTAGAAGGACTAATTCCTACAAAATTC

TTTTAGAAAACCTCAGAGAAATTAAGTACAGCAAGTGGCTCAAAACTAAAAATTAATTAT

AAACTATCCAGTGCAATCATAGAAAACCAAGGTCTTAGGATTGAAACAAATTTTCTTCTG

GTAAAAAACCTTAAGAATGAAGTAATCCTAGGAACACCATTCATCAGATCTTTGTTCCCC

CTCCACATATCCAAGGAAGGAATAACCACTTGGCACTTGGGAAGGAAAATCACCTTTGAT

TTCTCCACTAAACCAATTGCAAGAAACATAAACTTCATAGAAAAGAAGATTAGCCAAATC

AACTTTTTAAAAGATGAAGTTTCCTTTAGTAATATTCAAATCCAATTAGAAAAACCCCAA

TTAAAGGAGAAAATCCAATCTCTGCTAAAACACATCCAATCAACAATTTGTTCCGATCTG

CCACACACATTCTGGAATCAGAAAAAGCATATAGTTGATCTCCCTTATGAGAAAGACTTC

AGGGAAAAACAAATTCCCACAAAAGTCAGACCAATCCAAATGAATGAAGAACTCCTTCAA

TATTGCCAAAAAGAAATTAAGGATCTCCTTGATAAGGGATTAATTAGGAAGAGCAAAAGC

CCTTGGTCTTGTGTTGCTTTCTATGTCAACAAGCAAGCAAAACTTGAACGAGGAACACCT

CGTCTAGTCATAAATTACAAACCTTTAAACCAAGCCTTACAATGGATTAGGTATCCTATT

CCAAACAAGAAGGATTTACTCAATAGGTTAAACTCTGCAAAAATATTCTCAAAATTCGAC

ATGAAGTCAGGATATTGGCAGATCCAGATAAAGGAAACTGATAGGTACAAGACTGCCTTC

ACAGTTCCCTTTGGGCAATATGAATGGAATGTTATGCCATTTGGTCTAAAGAATGCCCCT

TCAGAATTCCAAAAAATTATGAATGATATTTTTAATCCATATTCAAAATTTTCCATTGTC

TATATTGATGATGTCTTAATTTTTTCTCAAACCATTGAACAACATTTTAAACATCTGCAT

ACCTTCATTAATATCATCAAACAAAATGGTCTTGCAGTTTCCCAAACCAAAATCAATCTT

TTCCAAACAAACATCCGATTCCTTGGTCACAATATTCACCAGGGAACAATTATACCAATA

GACAAGTCAATCGAGTTCGCCAACAAATTCCCAAACCAAATCTTAGACAAAACCCAGTTA

CAAAGATTTCTAGGGTGTCTAAATTATGTTGGTGAATTTGTACCATACTTAAACAATATT

GTCAAACAACTACATGATAGGTTAAGAAAAAATCCTCCTCCTTGGTCTGATATTCACACC

CAGGTTGTCAAAGAAATCAAGCTCAAAGTTCAATCTATAAAATGTTTGTATCTCCCAATT

CCGCAGGCATTCAAAATTGTTGAAACAGATGCATCTGACATAGGTTATGGTGGTATCCTC

AAACAAAGAGTCAATACCCAAGAAAATGTCATTGCATATACATCAAAGCATTGGAATTTT

GCACAACAAAAATATTCAACTGTTAAAAAAGAAGTTTTAGCAATTGTTTTGTGCATTTCC

AAATTTCAATCTGATTTGTTAAATCAAAAGTTTCTTATCAGGGTTGATTGTAAATCAACC

AAAGAAATTTTACAAAAAGATGTTAAAAATCTTGCCTCAAAACAAATATTTGCTAGATGG

CAAGCAATTTTAAGTGTTTTTGATTTTGAAATAAAGTATCTCAAGGGCACTTCAAACTCT

CTACCTGACTATCTTACCCGTGAATTCTTACAGAAAAATTATTATGCCGCCTAAGGCATC

CAGTACCTCCTTTCAGGGAGGAAGGAGTTGCAGAAAAGGTTTCAAGTTGGCTCTACTAGA

GCCATCCTCAAATAAAATTTCACCCTCAAAGACTAGGTCATTAACCCAATCTGGGTCATC

AACCCAGAAATGTAAACAACAAGATGGGTCATTTACCCTACTCGTTGAAATCAAACCTAA

GTCATCCACTCAGGAACCTTAAAAGAACCAAACATCAAAACAAGCCAAGGCTGACTATGC

CTTTCCAATCAAGACACTTTTGGCCCTTCAAGAAATTGGCCTAACAAAAATCCCAAAGAA

AACATGGGCTGACATAGCCTCTGAATCAGATGATGAGTCTGAAATTGATCTAAAAACCTT

AATCCAAAAAGCCAAAGACACAAAAATTATCTGCAATTCTCCAAAAGAAAAACAAATAAT

AACCCCAATGGCAACACCAGCCCCAAAACCCACTAACAGTTATATTTGCAAAAATAATTT

TTCAAGTGTTTTGCAAATGGAGTCGGAATATTGGGAAAAGAATCCCTTTAAAGCTATAGC

CAAGGCATTTCAACCCGGATTCCATTTCAAACCAACAACAATCAACAAAACAAGGACCTT

TTATGAATTCATTTTAATAGATTCAGACTCAGTTTCTATTAAACATTTAAAAGATCCAAA

AGAACCCCTTTAAAAAACCCACTCTACAATCCAAATTTTAAAAGTTCTGCAACCCAGACA

ATTTGGATCCAATTTGAATGAAATAAAAAAAATTTCTATACCTTTTTGATCCAAGAGGAT

ACACCTATTGGGACTATATGGATGCCTAGACCAAAGTGTATTGACATCAAAACAACAAAT

ATAAGCACTCATGGCTTATTTACTTCAAGACTAACACAGTTTATAATTTTTCAAATTGGT

TCCTTCAGTGGTGGTACTTTTTTGGACCAATCCCGAAAATATTCCCTGAGCAGGTTCAAC

AAGGATTTGCACAGTTCAACAAGCATTTTAACTCTCAGGAATCACAAATTCTAGCAGATT

TAAAATATTTTTCCAGTTTTACTTTGTCCTGGATATTTTCTTGGCAATACAGGTATAGCA

AAACAGAAAACAACAAGCAATTTCCATCCCTTCAGCGACATGGATTTGTCAAGTGGTGGA

ATCAATTTGACACATCAAAAGCACAACCAGATAAGGTGAAAATCTAGTTTAAAGCTCATC

TAGAGTTCCTGAAAGCACCCGATCAAGAGACTTCTTTGTTTCTCAATCAGAAGTCCCAAT

TAGCTGCCTTCGATTCCTAGCAAGATCTAACTCCAAGGAACATCTCACGAAAAAATTAAA

AAAAGTTTTGCAATTACTCCAAAGCCAAGAAGAAG

>GraimV [endogenous-virus-name=Gossypium raimondii virus] [strain=sequence cluster 1] [host= Gossypium raimondii] [moltype=genomic DNA] [note=complete genome] 7646 bp

TGGTATCAGAGCCAAGGAGGAGCAGGTATTTCTAAGTTTAAGAAAATATTCAATTTGTTT

TCAATATTTCCGAGTTCTGGTTTAACTGTCTTCATTAATGTATTATTATTTTGCTGAGAA

TTGATACAGTGTCCAGTTAGCCATGGGTTAGGCATGTCAAGGACAAAGTATCCCTGTAGG

CAACTTAATTAATTCAGAAATCCAGATAGTAGAACCCCCTGTAGGAAATAAGAAAACAAA

GAATGTTTCCTTTCCTTAGAAGAACCAATTCTTCTGCAAGCTTTGTTTCTAATCAATCCC

AAGAATCTGACCAAACCTCAAACAACACTGTTAATGAACAAGAAGAACATTATGAAAATA

TTCCCCAAAGATTTGATAATTGGACTCTTCCAAGAGTTCCAACAAACCAAGTTTACAAGA

AAACAACTTTTGAAAATTTAAATGCTTTTTGCAGTTATGTTATAAAAACAAAAGAAAGGA

GTTTACCTATTCAAAAAGAATATGAAACAATCCAATTATTAGATAAAGTCGTCATTAATA

AACTCAAAGAACAAAGATACAAATATATCCATTTTGGATTAGTCCAGGTTGGAGTCAAAC

CTCTTAGTGTTGAAGCCACTAAAAATACCTCAATATTAGTAGTCTTAAGAGACCAAAGAC

ACATCATGTTTAATGACTCATTATTAGGAACTATAGAAACAAGCCTATGTACAGGCCCAA

TACATTTTAATTGTTATCCAAATTTTATGGTTTCTTTGACTGATAAAAATATTTTACAAT

CTTTGACCCTCCAAATACATACCCATAACTACAAAATGCTTCCTGGTACGGAAGTATTAA

CTTTAGTTTATAGACTCCATTTCAAAGCTATGTATTCTGTTGTTAATACCAAGGCTTTAC

TCCAAAGCCCAAAAGGAGAAACCCTCTTAATAGAGACAGACACCACAAGATCTCATACCA

CAATCCCAAGAACAATACAATGGCATGAGATAAACCTTCCTGATAAGTGGAAACTTGAAG

GTGCTACAGACCCTGTGGCACCAACCCCCATAAGAAACACTTCATTAAGTGAAATTTCTC

AGCACCAAGATGGCACAGTTGAATTAAAATTCAATAGGCCACAAAGAATGCCTCCAAGAT

ACTCCTTCGAAATAGGAAGTACCAGCACAGCTTTTAGGAGGTTAAATATAGAAGAAGAGT

CAAATCCAGAAACACAAACAGTAGATTTTAGGACAGCCAGAGCCTCTGTCTCTTCTATCC

CAACAACATTTAGGACCAATTTACAAGGAATAGATAATTCCTCAAATATAGCTCAACCTA

TCTATGCCAGACAGGAAGAGAGTCCCCAAAATTCACCTAATATGTCACCAACATATTCAT

CAATGACTAATAATGCAAGACAAGGAGAAAATTCAGAAATATTTGTTTTAGAAAAAACGT

TTGAGATAAATAAAGAATGGTGTAGAAAGCACTTCTATTCCAACAAAAATAAGCAAAAAA

GAGAAGATTACTTTAAAAATTATAATGATAAAAAAGAAAGCATTCTTCAAGAATATTATG

GATTTATGAATACCCATAAAGTCCATATAAAATTCTTTGAATGGTTTGAAGAATATTATT

TAGAATCTGTTAATACAATAAGACATAATACTAGATGGCAGACTAATAAAGGAGAAGTTG

AATCTCGACATCCCCCTTTAATGGAGGTTCAATATCTTCACAAGAATACTGGAATAAAAG

CAAATCCTTTAAGAATGAGAGCTCCTGATGCAGGAGAACAAATCTCATCAAAAGATATTA

AAATGATAGTAGAACAAAACAATTATACAAATATTAATCTTCATACAATAGGAAAACAAT

TAGATTATATTGAGAATCTGGTAGAAAGTCAACCAATCAGGAAAGAACCAGTAAAAGAGA

TAACCGAAAAAAGTTCCAAAGAACCAATATTCACACCTTATGAGATTCCAAAACCTTTCC

AAAAATCCCAAAATGATTTCCTAACAGAAATCCAAAATAGACTTAATGCTTTGGAAAGCT

ACAAGTCTGAATTAATTGCCCCTGATACCCCAATACAAGCCCAATATTCAGTAAATACAT

TACACCAATCCTCCCAATCTGACTCGGATCAGTCAGATGAACAACAAATTAACAAGATGG

CTTGGAAAGAACCAAAAAGATTATATTATCCAAAAATCACTGCACCTGATCTTAACATAG

AAGAAAAACCTGTTTTCCAAAATAAATACAATGCTAATACAATCTATGAATGGAATATAG

ATGGAATGTCTGAATACAATATTCTTAGTTTATTACAACAAATGACAATGGTTTCAAATG

TTTATAAAACCCAAAATCAAAATGGATTAATCAGTGATCATGCTATAGCTAATCTTTTAG

TTGCTGGATTTACTGGTCAATTAAAAGGATGGTGGGATCATGCACTCACTAAAACCCAAC

AGGAAGAAATTTTAAAAGCAATAAAAAAAGATGACCAAGATAGAATTATTTTAGACGAAC

AAGGAAGAGAAATCCAAGATGCAGTAGCAACTTTAATTTTCTCAATCTCTAAACACTTTA

TTGGAGATCCTTCTCATCTTAAAGATAGAAATTCAGAATTATTATCAAATTTAAAATGCA

AAAAATTAACCGATTTTAAATGGTATAAAGATGTCTTTATGACTAGAGTTATGCAAAGAT

CTGATAACCAACAACCATTTTGGAAAGAAAAATTTCTAGCAGGACTTCCCACTTTATTAG

GAGAAAAAGTTAGGAATCAAATAAGAGAAAATTACAAAGGTATTATTCCATATGAAAAGC

TTACATATGGTGAACTAATTAGTTTCACCCAAAAAGAAGGATTAAAAATTTGTCAAGATT

TAAAATTACAAAAACAACTTAAGAAAGAAAGATATCAATGTAGAAAGGAATTAGGATCAT

TCTGCCACCAATTTGACATTAGGAATGAACCTTCTTCTTCAAAAACATGTTGCCCTGTAA

AGCCAAAAAATAGGAAAAAGAATATTTCAGAATATTATAAAAAACCCAAATATAGAAAAT

ACAGAAAAGGAAAGAAACAACAAAAAACAGAAAACAAAATTGATAAAACAATAAAATGTT

ATAGATGTGGAAAACCAGGACATATCTCAAAATATTGTAAAATCAAAAGAAAAATCAATA

ATTTAAATCTAGATGAAGAAATAGAACAAAAATTAAATGAAATCCTATTAGAAACAACTT

CTTCTGAAAATGATACATCTACTGAAACAGATGAATTACAAATAGACGAATTACATACAA

CATCCCAGTCTTCTGGTGATGAAAATGAACCTTCAATTAATATGTTAACCAAAGATCAAG

AATTTATGATTGAAGTTATTGATAAAATTCAAGACCTAGAGCTTAAAAGAGAATACCTTT

TGAAATTAAAATCCTCATTAAAAGATAAACCAGAAAAAGAAAAAGAAATTATTTCTAGTC

AATCACAAATGTATAATATACAAGACATAATATTTAATAAATATGAAAAAATAAAACCAA

GACAAATTACAAATTCTGAATTACAGTTGGAAATAAAACAAATTAAATTAGAACTTTCTC

AGCTTAAAATAGAACAACAAGAAATGAAAGAACAAATGAGGACCTTAAAACACGAAACCT

CAGAAAAAAGTTCGTCAGAAACTGAACCTGAACCTGAAGAAAATACACAAGAATATATGA

TGGTTCTAACTGAAGTATCTATTCAAAGATATTTAATAAAAATAAATATTGTCATAAATA

ATGAATTTCAATTAGAAACAATAGCCCTTTTTGATACAGGAGCGGACCAAAACTGCATTA

GAGAAGGAATAATTCCAACAAAATATTATAATAAAACATCAGAATCTCTTAAAGCTGCAA

ATGGTAAAAAACTAAAAATTACTTACAAAATTCCCAATGCAGAAATATCCAACAAAGGTA

TAAAATATCAAACATGTTTTTTAATGGTAAAAGATATTACCCAAGATGTCATATTAGGAA

CCCCATTCATATCCTTACTCAAACCTTATAAAGTAACAAATAATTCTATCTCCACCAAAG

TTTTAAACACTAAGGTAGAATTTCCTTTTGTAGAAAAACCGAAAATAAGAAATCTCAACC

TGTTAAAATCTTTATCCATTCATAATGGGCAAATTAATAATTTAATTAATTATAAACAGA

AGCAAATCTCTTTTCTAAAAGAAGAAATATGTTTTAAAAAATTAACTGAACAATTAAGAA

AAAGAGAAATACAAAAAAGAATAGATCAAATCAAAAAAGAAATAGAATCAACAATTTGTT

CTGACATTCCTAATGCCTTTTGGAATAGAAAAAAACACGAAGTAACATTACCATATGAAA

ACGATTTTGATGAAAGACAAATACCCACAAAAGCAAGACCCATACAAATGAACAAAGAAA

TGGAAGAATTTTGTAGAAAAGAAATACAAGACCTTCTTAATAAAAATTTAATTAGGAAGA

GCAGTTCCCCTTGGAGTTGTTCAGCATTCTATGTAATAAAAAATGCAGAACTTGAAAGAG

GAACACCAAGATTAGTAATTAATTACAAACCTCTCAATCAAGCCTTAAAATGGATTAGAT

ACCCAATACCAAATAAGAAAGATTTGCTACAAAAACTTTGTAATGCAAATATTTTCTCAA

AATTTGACATGAAATCTGGATTTTGGCAAATCCAAATAAAAGAAGAAGAAAGATATAAAA

CAACATTTACTGTACCATTTGGACAATATGAATGGAATGTAATGCCTTTTGGGTTAAAAA

ATGCTCCATCTGAGTTTCAAAGAATAATGAATGATATTTTTTACCAATATTCTCAATTCA

CAATAGTATACATCGATGATGTATTAGTATTTTCAGAAAATTTAGAAAAACACTTCAAAC

ATATATCGATCTTTATAAAAGTCATAAGAAATAATGGTTTAGTAGTTTCTAAATCCAAAA

TAAGTTTGTTCCAAACCAAAGTAAGGTTTTTAGGTCATTATATTACCCAAGGAACCATTA

CCCCAATAGAACGATCTATAGAGTTTGCTAGCAAATTCCCAGACCAAATTCTTGATAAAG

TCCAATTACAAAGATTCTTAGGAAGCCTCAATTATGTCATAGACTTTTATCCAGGTCTTA

GCAAATTATGTAAACCGTTATATGATAGGCTCAAAAAGAATCCACAACCTTGGACAAATA

ACCATACCAATATTATCACCCAAATCAAAAAACAAATCACTAAGCTTCCTTGTTTATATT

TAGCTGATCCAAATGCCCCCAAGATTGTTGAAACAGATGCTTCTGAAATAGGATATGGTG

GGATCCTAAAACAAGTTAAAGGAGGAAAAGAGCAAATAGTCCAGTTTACTTCCAGACACT

GGAATCCTACTCAGCAAAATTATAGTACTATTAAAAAAGAAATTTTATCAATTGTTTTGT

GCATTACAAAATTCCAAAGTGATTTATTAAATCAAAAATTTCTTTTACGAATCGATTGCA

AATCAGCAAAAGAAGTTTTACAAAAAGATGTTCAAAACATTGCCTCAAAACAGATTTTTG

CAAGATGGCAGGCAATTTTGAGCATATTTGATTTTGATATTGAATACATCAAAGGAGAAA

CTAATTCTTTGCCTGATTTTCTCACCAGAGAGTTTCTTCAAAAATGCCCCCCAAACTCCG

AGACAAAGGAAAAGGGAAAATAGAAGAATCATCCAAAACCCAAATCACCTCAAAACCAAT

TAAGTCATGGTATGAAATTTGCCATGAAGAAAATGAGAAATCATCATCATCGTCAAAATC

CTCCAAAAATACAATAATTCAAGATGCACAAGATCCAAATGAGATTGGTTCTCAAATCAA

AAAATGGATTGAGTCCCTATCTCAATCTCCAGAAGTAGCATTGGCCTTTTCACAAATGAA

AGAGGAAACTCCTCTCAAACAAATTGCTGCTGAAGCAGCAAAGGTTTCAAAAAATAAAGA

GATTGTTTTACACAAACCAAAATCTCTCAAAAATGTTTTAAAAGAGACTTCTCTCCAAGA

TGTTTTTCCAAAAGAGATAGCAGTGTCTTCACAGACTGCTACACAAAAATCTTCACAATA

TTTTCCAAACAAATATTTTGAAAAAATTCTTGTTATGGAGGAAGAATTTTCAGAAAAACC

TCCACATATACTTGCAAAAGAACTTTTCAATGGATGGCATTTCAAACCATTGGATTCTCA

AAAACCCCAACAGTATTATGAAAATATACTGGTCCAAACTGGATCAGTATTGTTCAAACA

TTACACAGATCCAAAAGATCCAAATTTTATTACCCATTCAACAGCCCAAATATTAAAAAT

TCTTCGACCAAGAGATTGGAGTGAAAACCCAAATTCTCCAAAGAAATTCCCAGCCAAATT

TACCACCAAAATAGACCATTACCCATATTTTACATATTGGGATTACCAAATGGCATGGTA

CAATGCCTTCTTAATGAACAACCAACACATGAGACATTCTTGGCTCATATATTTCAAATA

TGGCACCCAATTCAAATTCCCAAACTGGTTCCAAGAATGGTGGAATTGGTATGGACCATC

ATCCTTTGAGATACTACCAGAAAAAATTCAAAACTTATGGCCCAAATTCTTTGACAAATT

TCATCCTGAACCAGACCAAAAACACATTTACAGGACAATCCATTTTTTCTCAAAACTATG

CATTTCTTGGATTGTTTCATGGAATTATTCTTATGAGCAAGACCAACATACTGGAATTCC

ATTATTAGTTCGCAACTACAGGACTAAATGGTGGGACAAATTTAATGATGAAAAATATGA

TTCAAAATATTTGGATAATTTTTTCAACAAGAATCCAAGATTATGTAAGTCCGCAGCCCC

GGATCAAACCACAGCAAAATTTCTTCAAGCAAAGTCGACAGCTAGTGCAATGTTAGCTCA

AGCCAAGACCAAAAAAGAATACAAAAAACTCATGGCTGAAATGCTCAGCTCATTGGACTC

CGAATCTGAAGATGAATCTTCAGCATCCTCAATCAAGACGGTGGATCTTGCAGATGATAC

CACTTCAGTAACCATCACCAGGTCCAAGAAGAAATGATACAAGAAAAGATGGTGAACAGG

AAAGATTCCCTGCAAGAAATGATGCGTGAACAGGAAAGATTCCCTGCAAGAAAAGAGAAG

TGAACAGGAAAGATTCCCTGTGCGTGAACAGGAAAGATTCCCTGCAAGAAATGATGCGTG

AACAGGAAATATTCCCTGCAAGAAAAGACAAGTGAATAGTTCTGTAATTTGTATTTTTGT

AATCAGAGAATATATTCTCTGAAAAGTTTTAATCAAAGTCTCAAATGTAATGATTTCCCG

ATTCAAATGATGTAAAGAGAGAATAGCCCTCTATAAAAGGCTATCAGATTGAGAATGAAA

GGCAGAACTCGATATACCCATTTGAGTTGTTTCTCCAAAGTTTCTCCAAAGTTTTAAAGT

TTCTCCAAATTTTTAAGTTTTCAAAATTTAAGTTTTAAAATTTTTCAAACTAAGTTTTCA

AACCACGATTATCTCATTCCAAATTTTTAAGTTTTCAAATCACGATTATCTCATTCCAAA

CCCAATCCTCAAATCCTCGGGAGAGT

>JcurV_sc1 [endogenous-virus-name=Jatropha curcas virus] [strain=sequence cluster 1] [host= Jatropha curcas] [moltype=genomic DNA] [note=complete genome] 8268 bp

TGGTATCAGAGCCATTAGATTTAGTGCTACGTGGTAGAGATACATAAAAAGTCTTCATTT

TATGATATATAATAAAAGGTTTTGGCTTTGCCTACTGAACCCACGCCTACTGAACCCATG

AGTAAGTCCCATGTGAGTGGCATCTAACGGGTATAACCAGGAAGGGTATAACCAGGTAGT

CAAAAAATTTTATCGTATATTATAAAATGGATCCTTTCTTATGTAGATCGGCTAGTAGTA

ATAGATCTGAAGACTCCGATAACTCAGAAATTAAAAGAGTCATAAATAGAAATGAATTAA

TTTTGCAAGATTTTAGTAAAGCAATTGATGAATGGGAAATTCCTAAAGTTGATAAAGAAC

AAATTTATAAAGTTTCTAAATTTAATTTATTTAAAACAGATTATGTAATTAAAACCGAAG

AAAGAGATTTTCAATTAAGTAAACCCTTTGAAAGGATAACTCTTTTGTCTTCTAAAACTT

TGCAACATCATAAAAGTAAAAAATATAAATATATTCATATAGGATTAATTCAAGTAGGAA

TAAAACCTTTAACCAGAGAAGGATTAAATACTAGTATTCTTGCAGTAGTAAGAGATGCTA

GATTTTTAAATTTTCAAGATTCTTTATTAGGATCAGTTGAAACTAGCTTAAGTGAAGGAC

CGATCTCCTTTGAGGTCTATCCAGATATGACTGTCTCATTAAATGATTTAAATATTTTAG

AAAGTATAGTTTTGGAAATTAAAACTCATAATTATAAAATGAAGCAAGGATCAATTCCTA

TAGCTTTAATTTATAAAATTCATTATAAAGCAATGAATTCAGCTTTTGGAACTAAATATA

AATTACAACCTAAAATTGGTGAAACAAGATTTCTGCAAACAGACTTGACTAAAACAAACA

CAGTAATTCCTAAAACCATCCAATGGAAAGATATTACACTTCCAGATGAATGGATATTGG

AAGGGGTTGTACCTCCTCCTGTTCTAGATCCAGTAAAACCAAATGTCAATTTACAAAAAA

TTGAACAATTTGAAGATGGTAAAGTAACATTAAAATTTAATCGTAGTCAAAGTATGAGGT

ATACAGGGTCATCTTCCTCTGTTACAACAGATGAAAATGATAAACCTAGTTCCTCTATAC

CTTCTGTGATTTATGCTAAATATAAAGAAACTGAGCCTAAACCAATTATTCAAACCTTAG

TAAAACCAAGATATTCTACTTCGGATATTCCAAATAAAATGAAAGGTGTTGATTATAGTA

ATTCTATACCACAGGCAGTTTATAATCCTGTAGATAATTATGTAAATCAACAGTCAAACT

TAGTTAATTCACCTCCAGAATCACCAACAGCTTCAGCTATAACTGAAAATGTTAGTGATA

TAAACAGAGAATTAAATGTTCTTAGTAAAGAAGATATTGAGAAAAGTTTAATAAAAGAAA

TATCTAAAACTAAAAATTTACTTAAAATGAAAAAGATATTAAAAACTTATACTTTTGAAG

AATTAAAATATGAATATTTAGATTATAAATTAAATAACCAAACTGAAATTAAATTCTTTG

ATTGGTATAAATTAATTAAAGAAAATAAATCTGATAAAAGTGTAAATCCAATTACTATTA

GAGATAAAAGACCAGAATGGATAGTAAATGAACAAACAATAAAAAGTGATTTTCCTCCTA

AAACAATTAGTAAATTAAAACATCAAGATAATGAAATTATATGTGCACCATATAAAGTTG

CTCGAGATAATGATACAAATAGTAAGATAATTGAACAAAATAACTTTAGTAATGCAAGTT

TAATTTGTTTAAACTCACAATTAAAACGAATAGAACATATGCTAGAAGATAAACCTTTAG

TGTCAGAAACCATAAAAGGAAAAGAAACTGTAAAAACTCACATGTTTAAACCTTATCAAA

TTTCTCAATCAAGTAAACAAGAATTTGTAAATAAATTAAAAGATCAATTGAGTAAACTTG

AGGTTCATTCAGCAACTAATCAACATGTAGCTCCTGACACTACTGAAGCCTCTAGAAATA

TAAACATTATAGAAGAAAACACCAACTCTGATGACAGCGAACCTGACTCAGAAAATGCTA

AACAATTTGAATTAAATCCTATAAATCATCATCGAGAAATAGTAAATAGGGCACCAGACT

TAGGCATTATTGATAAACCTAACACTATATCTCAATTTAAATATAATGCTTCAACAATCT

ATGAATGGAACATAGATGGTTTAAGTGAATATAATATATTAAGTATTTTGCAACAAATGA

CTATGGCTGCAAATGCTTATAAAACGCAGTCAATTATTTCTGATAAAGCTATTGCAGAAA

TCCTCATTGCTGGATTTACTGGACAACTTAAAGGTTGGTGGGACTATCATCTCACTGAAC

AACAACAACTAGAAATTTTAAATTCAATTCAAGTCAATGATGAAGGTGAACCTATATATG

ATGAAAATGGTGAAGTAATTCAGGATGCTGTGTCATCTTTAATTCTCACTATTTCACTTC

ATTTTATTGGGGATCCATCTCACTTAAAAGATAAAAATGCAGAATTACTTAGTAATTTAA

AATGTAAACACTTAGGTAATTTTCAAGAATATAAAACTACTTTTCTAACTAGGTTAATGT

TAAGGGAAGACTCTAATCAAAGCTTTTGGAAAGAAAAGTTTTTGGCTGGACTACCTTATT

TCTTAGGAGAAAAAGTTAGGAACAATATTAAACAACAATATGGTCAACCTATACCATACA

GTAAATTAACTTATGGTCAATTAATTAGTATAATTCAAAAAGAAGGTTTGCAGATTTGTC

ATGACTTAAAACTTCAAAGAACAATAAAATATGAAATGCAGAAAACTAAAAAAGAATTAG

GATCATTTTGTAAACAATTTGATTATAGTTTAAAATCTCCACAGAAATGTGATGGACAAT

GCTTGCGAACACATAAATCTAAAAAACAGTATAAAAATAAATATTATAAAAGACAATACA

GGTCTACACCTAGTCAAGATAAATTTTATTCTAAAGAAAAATATAAACCCCATAGGAAGT

CTAAAAGAAAGCAACAAGCTAAAAATTCAAATAAAACCTCTTATAAAGATTTAACTTGTT

ATAATTGTGGTAAAAAGGGACATACTTCAAAATTTTGTAAATTTAATAAAAAGTTAAATG

AATTACATTTAGATGAAGAAGTGTTAAATAAAATCCACGAATTATATCTTGATTCTGATT

CTTCAGAATCTGAGGTTATAGAAAGTGAAAATCAAATTGATGAATTAGGTACAACAACTA

GTTCATCTGATTATTCTATAAATATGTTAACTAAAGATCAAAGTACGCTTTTAGAAGTAG

TAAATTCTATAGATGACCAAGACACTAAAGCTAAACTCCTTCAAAAACTCATTAAAAGTT

TTGACGAAACCCAGATAGTAAAAGAGGATATAACTCCTAAAATTCAACATAATACTTATG

ATCTCACAACTATATTAAATAAAAATAAGCATAAAAAAGTAGAAGCAGAAAATTCCACTA

GTAATTTAAAAAATGAAGTTAGAAATCTAAAGAAGGAAGTTAGTGAATTAAAACGACAAT

TTCAACTTCATGATAGACTAATAAAGGTATTAGAATCTCAACTTATTTCTAGTTCTTCTT

CTGATGAAGCAGAACCAGATAAAGATGAAATAAATCAATTAGATGAAGATGTAGATAGTA

ATTTTCTAAACTTATTACAAGAAATAACTTCAAGAAAATATTTGGTAAAAATAAACATTC

AGGTAAATGATTTTGTGATAGATGCAATTGCACTGTTTGATACAGGTGCAGATTTAAATT

GTATAAAATCAGATTTAATCCCTAAAAGATATTGGTCAAAAACTAATGAAAAACTAAGTA

CTGCAAATAAATCTAAATTAGAAATTATAGGTAAAACAGATGCACAAATAGTAAATCAAG

GTTTAGAAATAAACACAGTATTTTTGTTATCACCTCAAATAAATTATATGGTTATATTAG

GAACCCCTTTTATAAACCTAATAACACCATATTCAGTTAAAGAGGATTGTATTAAATTTA

AAATAAGAAATAGTACAGTTAAGTTAGAATTCACTGAAAAACCTAAGAAGAAGCAATTAA

ATCTCTTAAAAGCTCATTCAATTAAAATATTAGAGATCAATTCTTTAATTAAATCTAGAG

AAGAAGATTTAAAAGATGCAAGAAATAATATGTGTTTAAACAAAATTAAACAACAATTAG

AGACTAAAGAAATACAAGATAAAATCACAACATTAAAAGAATCTATGGAAAAGGAACTAT

GTGCTGATCATCCACATGCTTTTTGGGATCGTAAACAACACATAGTAGATTTACCATATG

AAAAAGATTTTGATGAAAAAACTATTAAAACTAAAGCTAGACCAATTCAGATGAATCCTG

AATTGATAGAGCATTGTAAAAATGAAATTCAGGATTTACTCAATAAAAAATTAATATCTA

AATCTAGAAGCCCATGGTCTTGTGCAGCTTTTTATGTCAATAAAAATGCTGAAATAGAAC

GTGGTGTTCCGAGATTAGTAATAAATTATAAACCATTAAATACGGCTTTAAAATGGATTA

GATATCCAATTCCTAATAAAAAAGATCTTCTAAAAAATTTATATAAAGCAAATATTTTCA

GTAAATTTGATATGAAATCTGGATATTGGCAGATTCAAATTGATCCTAAGGATAGATATA

AAACTGCATTTACAGTTCCATTTGGTCAATATGAATGGAATGTAATGCCTTTTGGACTAA

AAAATGCTCCTTCTGAATTTCAGAGGATTATGAATGATATTTTTAATCCTTATTCTAAAT

TTTGTATTGTATATATTGATGATGTACTTATTTTTAGTACAACTCTTCATGAACATTTCA

CACACTTAAAAACATTTTGTATGGTAATTCGGCAAAATGGTTTGGCCTTAAGTAAAACTA

AAATGGACTTGTTTAAAACTAAAATCAGATTTTTGGGTCATTATATTGAGCAAGGTAAAA

TTCAACCCATAGAAAGAACTTTGGTATTTGGAGAAAAATTTCCAGATGAAATTACTAATA

AACTCCAATTACAAAGGTTTCTTGGTTGTTTAAATTATGTCATTGATTTTTACCCTAATT

TAAATTGTATGTTAAAATTATTGCATGATCGATTACGAAAAAATGCTAGTCCTTGGACGG

ATAAGCATACTCAAATAGTTCAAAAGATAAAATCTTTAGTAAAAGAAATACCATATTTAT

ATATTCCTAATCCTACAGCATTTAAAATAGTTGAAACTGATGCTTCGGATATAGGATATG

GTGGTATTTTAAAGCAACAACTTGATGGTAAAGAGCGTATAATCCAATACACTTCAGGCC

ATTGGACTCCCGCTCAACTTAACTACTCCACTATTAAAAAAGAAATTTTGAGTATAGTAA

TATGTATAAATAAATTTCAGTCTGATTTATTAAACCAAAAATTTTTATTACGTATTGATT

GTCAATCTGCTAAAGATGTTTTACAAAAAGATGTTAAAAATCTTGCCTCTAAACAAATTT

TTGCACGATGGCAAGCATTATTAAGTATTTTTGATTTTGATATTGAATTCATTCCTGGTA

ATAAAAACAATATTCCAGACTTTCTTACTCGTGAATTCTTACAGAACAATGGGCAAGAAA

AAGGATAAGGGAAAAGCCATTAAAGAAGAATCAGACTCACCCACTAAAGCTCTTTCTGAG

CATTCTGAAGACCTCACTAAAACCCCAGCCTTAAACACTAAAACCTCTAAAGATCTTCAA

AAATGGATTGAACAACTTAGTCAATCTCCTGAAGTTTTAAAGGCGTTGCAAGGTATAGCA

TCTTCTTCCACAGAGGATGCAGGTATGTCTTCTAAACTTAAAACGATCGTACCCGTTCAT

GGTACGGCTAGTCTGTCTCAAACGGTAGACAGTAAAGACTTATCAAATCCGTTGATGGCT

GTGGATTTGCCAAAAATCCAGTATACTCCTCAAAGTATACATAAATGGTTTGTTAAAAAT

ATTTTTGATTATGAAATTGAGATCGAGGATGGGTATAATAATATTAATCCTTGGTCAATT

TTAAAACACTATTACCCTGAGGATTGGTATTTTACTCCAAATGATCTTTCAAAACCTCAG

GAATATTATCATAATATCTTACTTGATACTGACTCTGTAAAAATTAAACATAATTTTGAT

CCTAAAAATAAATCTATTATTTTGTATTCTTCCATAAAAATCCAAAGAGTTATTCATCCT

AAAGATTGGCCTGCTCCATCCCTTTATACTCCAGTAAAATTCAAAGTTTTATATAAGCAT

GTGCATAGTACTACTTATAATTATTTTGACTATAAAGAAGCATGGGAAAAAGTTTTCTAT

ATTCAAAATCCCACTGCTACTCATTCGTGGTTAATTTATTTTGATCAGTCTTTTCGTACT

GATTTAAAGATTCCAAACTGGTTTAAAAAATGGTGGCAAACAAGGGGTTTATCCAATGAT

ATTCTCCCTCCTCCTATCATCTCTTTATATCAGTACTTTAAAGCCCATTATAAACCTAGA

CCTGAAGAAGCCCATATTCCATCCTTAATGTACTATTGTATGGCCTTCTTTACGCCCTGG

GTATATCAATGGTATTTGGACACTCAGTATGCCATAGGAACTCAAATTCCTATGTTGGTA

AAACGACACAAGATTAAATGGTGGGGCTCTTTCAAGAACTCTACTACTGAACTTAAAATC

GATCAATGGATTCGAAATAAACTTTCTGGTACACCTGCATTAACCTATGCCAATACACTT

GCTTTAAAGGATCAACCTACATTTGGAATCCAAAAAGCACAGTGTCAAGCCAGGTTAGCA

GCGGCTAAAACACCTGATGAGTATAAAAGAATTTGCCAGGAAATGTTCCAGCAACTTTCC

ACAGATTCTCCAGTAAAAACCTCCTCAAGTAAACCGCCAGCAGGTTCTTCAGATAAATCG

TCATCCAAACCCAGTCTTAAAGGCAAAGCCAAATGGGCAGACATGGCGTCCTCCCTCTTC

GTCAACATCATCATCTTCCCTTGTTTCAAGTAAAGATAGCAATGATGATATTCTTCAATC

AATAATAGTAAAAAGTAAAGCTGACAAAAAAGAAAAAGAAAAAGCAAAGTTAAAACAAAA

AAGCAAAGTAAAAAGAAAAGAAAAGAAAAAGAAGGACACTTCGTCCTCATCTGATTAAAA

GGCAGAAGGAACCTTATTCCTTCACGTGCCTCTACAGTAAAAGGCAAGCTCCAATGGCCG

ACCAAGCACTATTCCTCTACAGTAAAATTCCGTGACAAACAGAGTAATGTGCATCAACAG

TAAAAGTAAACAGAAAAGATTCTTTGTTTTTTCTATATAAAGGGAGCCTCTCCCCTTGTG

AAGGCATAAGAAAGTTTAGTTTTAGTTAAATTAACCATAATCCCTCTCTCCTTCTTCGCT

CCTAGTTCAAGCTGTAAGCGTTCAAGTTGGAGAAAGTCTAAATCTAAAACTCTTGTAAGT

TTTAAATCTTGTATTTATATTTTGTAAATAAATAAAGTTTGTATTTTATCTTAAACTGCT

ACGAATATAAGTCCGTGTTTACGGCACCTTTCTCTTCTCCCTCCACACTCTGACTTAATT

CTGGGGATCCCAGGTTAAGGTTGCAGGAACCCTAAACCTTTGTGCTAAATTGTTGATTAA

ATTTGTTATAAGTATGCTCTGTGGATGCAGCTTAGTCTGATCTTCCACATCAGAAATATT

GTGTAAACTGTATGTTAATTCTGAAGTAAAATTATACTTAATGAATTATATAAGTATTCA

TATAAACCCAATTAATCATAGTATTTTAGTTCACAGTTATAATTTATCTTTAAATATTTG

TATCTGTATTTATTTAAATCCTAGTAATAATCTTAGCGCTCTACATATATATGACTTGTA

ATTCTTTAGTCGTTAGTCTTTATTAAAGTCTTTGGTCTACTTTAAAGTCATAAGTCATTA

GTCTTAGTTTTTGTTGGTATATATATTCTTTTAGTCTTAAAGTCTTTATCCTTAAAGTTT

ATATACGATAAAATTTAAAAAGCATAAGTCATTTGTTTTCAAAGTCTTTAGTCTTTAATT

TTTGTATATTCTTTTAGTCTTAAAGTCTTTATCCTTAAAGTTTATATACGATAAAATTTA

AAAAGCATAAGTCTTTTGTTTTCAAAGTCTTTAGTCTTTAATTTTTGT

>LjapAV [endogenous-virus-name=Lotus japonicus A virus] [host=Lotus japonicus accession Miyakojima MG-20] [moltype=genomic DNA] [note=complete genome] 8013 bp

TGGTATCAGAGCCTGATGGGGAAGTAGGAACATGCTAAATATGTTAGATATAAGTTATTT

CTCTGTTCTGTTCTTATATAATTAAGCACTATGGTCGGGATCTGGACTGGTAGTACACTT

TGAACTAAACTAAAATGAAAGCTCTTTTGAAAACCTTCTAGGATTATTACTATTCAGACT

TATGATCATCTCTGCAGCGGAGGCGTTGAAGGCAGTAAGTCCCTGTGTGGCAGTAAGTCT

ATATTGGCCATACTACAGACATGTTATTAAGTTCTGAATATTTTTAATGTCCTTGTTTAG

TTTCTTTTTGTGGTGGCTTAAGTCTGATCCAGATCTATACTGCTCAACTATTAAGAACAA

CACAGAACGTAACTTATATTTCTAATTTATTTATCTGTTTCTATAGAACCAGGTGATGGC

GATGATACTTACGGGTGAGGAAGATCTATCTTTTATGATGCTTCGCTCTTTCTCTTCCAA

GCTTTCTTTCAGATCTTCCCAACCCTCTTCTTCTTCTATCGATCATCCTCATCAATCCAA

TCAATTATCCAAATCTGTCTTCAAAGAAGAAATTCATTTTGAAGATATCAATCAAGATCT

CGACAACTGGGAAATTCCAAAAATCTCTCATGCTGAGATCTACAAACCAAACGGATCCTT

TTTCAAACGATCAGATTTCATTATCAAAACCGTTGAGCAAACCTACAAGCTCAGATCTGA

TGATGAAGAAATCCATCTGCTTTCCGAGAAAAGCATCAAAGAACATATCAGCAAGAACTT

CAACTATCTTCATATAGGTAGTGTTCAAGTTGGTCTTAAACCCCTAACCAGGAAATCTCT

TGACATCGCTGTACTCCTTTGTCTTAGGGATGTTAGACATAATCAATTCCATGACTCCCT

CTTAGGAACGGTTGAAACCAGCCTGAGTAATGGACCAATCTTTTTCAATTGCTTCCCAGA

TCTAACTGTTAGTCTGGAAGATAAAAACATCCTTGATGTCCTCTTCCTAAATATCAAGCT

TCACGGCCTTGATATGAAAGAAGATTCCATTCCAATCTCTTTGATCTACAGGGTTCAATA

CAAGGTAATGAACTCTATCAAATCTTATTTCTTGCGAACCAATTGTGAGAAATCTGGAGA

AACTACCCTCTTCCTCACAGATTATGATAAGGCCAATGTTATTGTGCCCAAAACCATCCA

ATGGAGTGATATCACCCTTCCAGAAAAATGGTCTTTGGAAAAGGCTACTCCAGCCCAACC

TGTTGAAACTCCTGAGTTTCGTGAAATCATCCAACACCAATCTGGCCAAGTAGATCTCAT

CTTTGATAGGAGAAATTCCTTCTCTATCCCTAGATCTATTAGGACGTCCTCAAATGATTT

CCAATCCGCCATGTCTAGAAGATCTTTTTCTATGACTAGAAGGAACCAGCCTGAGTCTAG

TGCTCGACCTTCCCTCTCTCATGACGGATCTACTATCCATTTAGCTGGATATGTCAATAC

CACTCCTGTCAAGACTACCTATGACAAGGGTGATGAAGATCATCAATCCACCTTATCTCC

AACGTATTCTTCTCTAGAAACCCCTGGAGAAACTTCTGATTTCATTGGAGCAATCACTAC

TGAATTTGTCATCGACAAAGGAAGTATCAAGGCAGATTTCTACTCTGACCGATGGACTCA

GCAAAGAAAATGGTTCTTTGCAAAGTATCAGGGTGAAAAGAGAAAGAAGATCCAAGAAAA

ATTCTATGGGTTCCTTGAGAAAACCCAGCAAAACATCTTCTTTTTTGAATGGTTTCAAGA

TTATGCCAACAAGAATTGGAAATCTTACCCCTTCCAAATTGACATGGTTAAATGGCAGCA

TAAGGATGGAAAAGAAACCCTTTCTAACCTGCCACCTAAAACTCCTTTCCTTCTTAAAGG

AGCTCAATCTACACCTGTCCTAGCTTCTCCCTTCAAGACTAGGAAAGAAGAAGGTGATGT

CACTGGTAAAGACATCAAAGACCTCATGGAACAATCCAATTATACCAATAAGTATCTTCA

AATTCTTGGAGAATCCCAAGTGCAGGATTCCTCTTCCAGGAAAGGAAAAGAAAAGGTCAA

GATTGAACCTTCCTCTTCTAATCTTGATATCCCTAAAGGATGTCAACTTGAAAAACCTCT

TTTCAAACCTTTTCAAATCAGTAGCCGTTCCAGACACTCGGCTCAAACTCTTCGAGCAAA

AAAGGAATCTGACAATGAGCTCCTCCAAAAGGTGGTAGAGCAACTTCAGCTTCTCAAACA

AGTTATTCCTGAGACTCCGGAACCTCCTGAGACTCCGGAACCTACTCCGGAAGCAGTTTC

TAATCCCACTATTACTGCTCCTCCTCCTCCAAAAACTCGTACTTCTACTCGTAATACCTC

TACTTCTTTAAATAATATTGAAGATGGTAAGGTTGAGTCTGATTCTGATATTCAATCTAT

GAAGTCTGACGTTCAATCTATCAAGAGTATTCCGGTCAATACTGTCATTCACAATTCTAA

GAATCAGTGGAGAAAAGAAACCAAGCTCTACTATCCTAGAGCTACTGCTCCTGATCTTCT

TTTAGAAGAATCTTCAAACTTCAAAAGCTTCAGTGCCAACAATGTCTATGAATGGAATAT

TGATGGTGAAAACGAGTATGGCATCACCAAAATCCTCCAAAATATGACTATGGTAGCCAC

TGCCTATGTTACCGCCAACAATTGTCCTGAATCTCTTATCGTTGAAGTCTTGGTTGCTGG

TTTCTGTGGCCAGCTCAAAGGTTGGTGGGATAATTATCTCACTCAAGACGAGAAGGATCA

GATCTTGACCGCTGTCAAGACTGATGAAGAAGGTAATCCTATCATGGAAGATGGCAAATT

CATCTCTGATGCTGTCAACTCCTTAATCTTTACCATAGCCCAACATTTTGTTGGAGATCC

TTCCCTCATCAAGGACAGATCTGGTGATCTTTTGTCCAATCTTAAGTGCAAATCTTTGGG

TGATTTCAGATGGTATAAGGATACCTTCCTGACCAGGGTTTACACCCGTGAAGACAGCCA

ACAAGCCTTCTGGAAAGAGAAATTCCTGGCCGGTCTTCCTAAATCTTTTGGCGACAAAGT

TCGTGAGAAACTTAGAAGCCAAAATCCGGGGGGAGAAATCCCATATCACACCCTTAGTTA

TGGACAGCTCATAGCTATCATTCAAAGAGTTGCTCTCAAAATCTGTCAGGATGACAAAAT

CCAACAGCAACTCACTAAGGAGAAGTCTCAGAATCGCAGGGATTTGGGTACTTTCTGCGA

ACAATTCGGAATTCAAGGTTGTCCCAAGAAACCTAAACCCAGAAAGCAAGATCCTCCTCC

CAAACAACAATGGAGAAAGAGATCTAGCAGGAATGATCATAGGAAACCCAAGCCTAGATC

AAAACCCCAATCTTCGCAAATTCCAAAGAACCCTCCTGAGACTAGACCCTCTCAAGGAAA

AGATGTTACCTGCTACAACTGCGGCAAGCCGGGCCATATTAGCAGGTATTGCAGACTCAA

AAGGAGAATCTCTGAGCTTCATCTTGAACCTGAGATCGAGGACAAGATCAACAATCTCCT

CATTCAAACTTCTGATGAAGAAGAATCTAATCCTTCGGATTCTGAGGTCTCTGAAGACCT

AAATCAAATTCAGAATGATGATTCTCAATCATCATCTTCCGTCAATACCTTGTCCATCAA

CACTTTGACCAATGAACAAGATCTTCTCTTCAGGGCCATTAATTCTATCCCTGATCCTGA

GGAAAAGAAAATCTATTTGGAACGCCTCAGATCTACTCTTGAAGATAGGCCTCCCAAAAG

TCCTATAACCACCAATAAATTCAATCTTAGAGATACTTTCAAGCGTCTTGAGAAATCTAC

GGTCAAACCCGTCACTATTCAAGACCTTCAATCTGAAGTCAATTCCCTCAAGACTGAGGT

TAAAAGTCTCAAACAAATCCAAAATAGTCAGCAGCTTATCTTAGAAAAACTGACTAGAAA

TTATGAGGAAGATGATTCTTCTGTACCTGATTCCAATCCTGCTCCTAACAACAATTGCGA

AGACTTTCTGGAAAATATTAACCAGGTCACCATTCAGAAATTCTTCATCCATGTCAAAAT

CCTCATAGGAGATTTTATTCTCGAAATCCCTGCCCTCTTTGACACAGGGGCAGATTCCAG

TTGTATCTCGGAGGGACTCATTCCCACCAGATATTTTGAGAAAACAACTGAGAAGCTCAG

CGCTGCTGAAGGATCTAGACTAAAGATCAAGTATAAAATCCCCTCAGCTATCATCAAAAA

TGGTAGTCTTGAAATCGAAACTCCATTTCTGTTAGTCAGAAATTTGAGTCAAAAAATCAT

TATAGGGACCCCTTTCATTAAGAAGCTCTTCCCCTATAATACTGACGAAAACGGCATCAC

TGTTCAGCATCTTGGACAACCTATCTTGTTCAAATTCTCTGAACCTCCCATAGATAAGAC

TTTGAACGTCATATCCTACAAGGAGAAGCAGATCAACTTCCTCAAGGAAGAAATCTCTTA

CAGAACCATTGAGGATCAATTGCAACAACCTTCTGTTAAATCAAGGATTGAGAATATTTT

GGAAAATATTCAATCCAGCATCTGTTCTGATCTACCCAACGCTTTTTGGGAAAGGAAGAG

CCATATGGTGGAACTTCCTTATGAAAAAGATTTTTCAGACAAACAGATTCCAACCAAGGC

TAGGCCTATTCAAATGAATGAAGAACTTCTTCATTTCTGCCAAAAGGAAATCAATGATCT

CCTTGAAAAGAAACTTATTCGCAGGAGTAAGAGCCCTTGGTCCTGTGCAGCCTTCTATGT

CAACAAACAAGCTGAGATAGAACGGGGAACCCCTAGGCTGGTTATTAACTACAAACCTCT

CAATCAAGCCCTTTGTTGGATTAGATATCCTATCCCCAACAAGAAAGATCTCTTAGCCAG

ACTGCATGATGCTAAGGTCTTTTCAAAATTCGACATGAAATCTGGATTCTGGCAAATCCA

ATTGCAAGAGAAGGATAGGTATAAAACTGCTTTTACTGTTCCTTTTGGGCAGTATGAGTG

GAACGTTATGCCTTTTGGGCTAAAGAACGCCCCATCTGAATTCCAAAGGATTATGAACGA

AATCTTCAATCCTTATTCCAAATTCACTATTGTCTACATTGACGATGTTCTAATCTTTTC

CCAAACTTTAGATCAACATTTCAAACATCTCAATACGTTCATCTCTGTAATCAAAAGGAA

TGGCTTGGCTGTATCCAAGACAAAGGTCAGTTTGTTCCAAACCAAAATCAGATTCCTTGG

TCACAACATCCACCAAGGAACCATCATTCCTATCAATAGAGCCATCGAGTTCACGGATAA

ATTCCCTGATCAAATCATTGACAAAACCCAACTACAAAGATTCCTGGGTTGTCTCAATTA

TGTTGCGGACTTCTGTCCTCAACTCAGTACCATAATCAAACCCCTTCATGATAGACTCAA

GAAGGATCCTCCACCTTGGTCCGATATTCATACCAATGTGGTCAAACAAATCAAACTTCG

TGTCAAGAATCTCCCTTGTCTTTATCTCCCTAATCCTCAAGCTTTCAAAATTATTGAAAC

TGATGCCTCTGATATTGGCTTTGGTGGTATTTTGAAACAAAAGGTTTTTGACAAGGAACA

AATTATTGCTTTTACTTCAAAACATTGGAATCCTGCTCAACAGAATTATTCTACTGTCAA

AAAAGAAGTTTTAGCAATCGTTTTATCTATTTCAAAATTTCAATCTGATTTGATTAATCA

AAAATTCTTAGTCCGTGTAGACTGCAAATCTGCGAAAGAAATCTTACAAAAAGATGTCAA

AAACTTAGCTTCAAAACATATCTTTGCCAGATGGCAAGCTATTTTAAGCGTTTTTGACTT

CGATATTGAATATATAAAAGGATCTAACAATTCTCTCCCTGATTTCCTCACTCGTGAATA

TTTGCAGGGAAGATCATAAGATGTCCTCCAGAGGAGGCTCCTCCTCCTCCCGAGGAAGAG

GAAGAAACAAAGAAAAGAGCAAAGGTATTATAATCTCTGACTCTTTAATCTTATCTGGGC

CCACTGCCCATCAATCTGGACCCACTGTCCAAAAATCTGAGCCCACTGCTCAAACCCAAT

CTTCACAACCAAAACAAACCAAAGCTGATTATGCTTTATCAATTCCAACTCTCCTTGCCT

TCAAACAAGCAGGCCTGGATAATGTTCCTCAGGGACTCACCAATTTACCAAATAAATCTT

GGGCCAGCATCGCTGATAAAGATGATGACCTAGATCTCCAATCCCTCCAATCTTTTATTG

AAAGAACAAAAAGCTTAGCAACTCATGATGGAAAGAAACCAGTTGTTGTTGCCCAATCTA

GCCCAGTTGCCCAATCTAACCCAAAATCGGAATATTTGACCAAAACTATCTCCAAATTCC

AAACCTTGATTGAACCAGAATGGTGGGACCATTCAGGAGGGAACCTCGCCAATAAAATTG

GTACTAAACTTTTCCCTGGTTATCTTGATCCAATCCACCCAAACAAAACCCAGAGATTCT

ATGAGTTTATCTTGGTTGATACAAACAGTGTGGATATTAAGCATTTTAGGGATAAAAATG

ACAACTCCCTAATTACCCACTCCACACTCCAAATTCTCCGTGTCCTTCGTCCCACTGACT

TTGGACCCAATCCAAACACCTACAGAAAATTTTCCCAGAATTTTGACCCTATAGGTTTTA

ATTATTGGGATTACATCAAAGCTTGGGAATTTACAATCCTTGGCCTGCAAAACCAAAATT

TCAAGCATTCTTGGCTCATTTACTTCAAATGGACCAACAAATATTCCTTTCCAATTTGGT

TCCATTCTTGGTGGAGCTTCTTTGGTCCAACTACTGAAATCTTTCCCCCTGAAGTTCTGG

AGGGATACAATCTTTTCCTGAAACATTGGGACAAAGAGTTTAACAATTATCCTGATTGTT

TAAACTTTTATACAATCTTTTCCCTTTCTTGGGTATTCTCTTGGAGGTACGGTCTTAAAG

CCAAACCCCAACCAATTCTCAACAAACATGCCCTTATCAAGTGGTGGAAGGCTTTTGATG

CTTCCAAAGCTCACAAGGATAAAGTAATTCAATGGTTCAAAAACAACCAGAAATATCTCA

AGCATGCAAGTCCGCAAACCAGCCTCTTGCTAAATCAAAAGGCCCACATCACAGCGGCCC

TTGCAGGAGCTTCAACAGAAGAAGACTTGCTAAGAAATCTTCAATCTGCTCTTCAATTAT

TGAAGGAACCAATTAAGCAAGCCTCATCTTCAAAGGCCCCTCAACACAAAGTCTCCGAGC

CATCCTCTCTTGGGTCGTCAGATTTTGTCCAAACCACAGAATCAGATGATGATGACTTCT

GCTAAGTTTGTCGTCTTGTAATCATTTTTATTCTGTAATAATTCAATTTTTGTACTGTAG

CAAATAGTAAAATTGACTTTTTACTGTAGCAAATAGTGCCAGTCAAATAGTACCGTCTAC

CGGAACTATTCAACCGGAACTATTCAAACAGTGCCCAATCTTTTCTTTAAATAGGCTCAT

TCTGAACCTTGTTAAGCAGAGTTTGGTATAGAGCTTAGATAGAGAGTTTAGAGAGAGAAA

CACTGAGAGAATTCCTTCTGTAAGTTCTCCTTCAGTTTTCCTTTCTTTCAATCTTTTCTG

TTTTTAAGTTTCAGTTTTTCAGTTTTTAAGTTTCAGTTTTGTAATCTATGTTTATGTAAT

ATATATCTTGTTCAAGTTTTATTTTTCTGTTTATGGAAGGCTGAGCCTCCTGTTTTAATT

CCTGTTTTTATTTTTCTGTTTATGGAAGGCTGAGCCTCCTGTTTTAATTCCTGTTTTCTG

CAATTTAATTTTCTGCAAAAAGCCCCTCCGTGGTGGCGTCCTACTTCTCTTAATCCTCTT

CTCATCCCCCTCCTTCTGCTTCCGACCCCCTCT

>LjapBV [endogenous-virus-name=Lotus japonicus B virus] [host=Lotus japonicus accession Miyakojima MG-20] [moltype=genomic DNA] [note=complete genome] 7729 bp

TGGTATCAGAGCCTGATGGGGAAGTAGGAATATGATGAGTAACTTGGATATAAGTTGTTT

ATCTTCATTGTTCTTACATGTTTAAGCTGGAAAGAACCGGGATCTATACTATGAGTACAC

TTTGGGACTTAAGTACTGTTTCGATTTAGAATTTTTATCTATAGCGGAGGCATTTGTCTG

TAAGCCCCGTTTGTTGTGGGCTTCTCTGTCTTTTTGGCCATACTATAGATAAAATTTGAC

AGATCTGAACAATATTTATTTCCTCCGTTTAAACACTTGTTTTTGAGTGGCTTTAGTATG

ATCTTGTCTTTATTTCCAGCCTAAGAATTAAGAACAACCCAGAACATAACTTATAATTCC

AATTTAATTATCTATTTCTATGAAACCAAGTGATGGCGATGATATTAATGGGTAAGGAAG

ATTTGTCTTTCATTATGCTACGCTCCTTCTCCAAGCTTTCTTTCAAATCTTCCCAACCCT

CCTCATCTTCTCTCAACGAAAATGAATCTCATAATCATTCAAAGAAAATCTTCAAAGAAG

AAATTCATTTTGAAGACATTAATCAGGATCTTGATAATTGGGAAATTCCAAGAATATCTC

ATAACGAGATCTACAAACCAAATGGATCCTTTTTCAAAAAATCAAATTACGTCATTAGAA

CTGTAGAACAAAAATACAAAATTCAAAAAGAAGATGAAGAAATTCATCTGCTCTCTGAGA

AAAGCATCAGAGAACATATCCTCAAAGGTTTTAACTACCTGCACATAGGTAGTGTTCAAA

TTGGTATGAAACCTCTTACTAGAGGTTCACTAGACGTCGCCGTTCTTCTATGTCTAAGAG

ATACTAGACATAACAATTTTCACGACTCTCTCTTAGGAACTATTGAATCTAGCTTGAGTC

ATGGACCAATCTTCTTCAACTGTTACCCGGACTTAACAGTAAGTCTGGATGACAAGAACA

TTCTTGATGTTCTATTCCTTAACATCAAGCTTCATGGATATGATATGAAGGAAGGATCTA

TCCCAATGACCTTAATCTACAGAGTTCAAGATAAGGTTATGAACTCAATCAAATCTCATT

TCCTTAAAACAGGTTGTGAAAAGTCTGGAGAAACCACTTTGTTTCTCACTGATAATAAAA

AAGCTAATATTATCGTCCCAAAAACTATTCAATGGAATGATATCACTCTACCAGAGAAAT

GGTCAATAGAAAGAGCTACACCGGCTCAACCTATTGTCACCCCTGACTTCCGTGACGTTA

TCCAACACCAAACTGGGCAGGTTGATTTAGTCTTTGATAGGAGAAACTCCTTTTCCCTAC

CTAAATCAATCAAAAATAAAGAAGACTTTAGATCTACTATGTCTAGAAGATATATTTCCA

TGTCTCGGACCAACTCCATCAGTAGAGCTCAACCTGAACCCTCCCATGATGGATCTACAA

TCCACCTTACTGGTTTTGAGACTACAACACCGGTAAAAACAACATACCAAATGGATAATG

ATGACCAGAGATCTACTTTATCCCCAACTTACTCCTCCATTGAAACCCCAAATTTCATTG

GAGCAATCAACATAGAGTTCACTATTGATAAAGAATCTCTTAGAAAAGATTTCTATTCCG

AGGAATGGACTCCTCAAAGAGAATGGTTCTTCAAAAACTACCAAGGCCAAAATAGAAAAA

ATATCCAAGAGCGATTCTATAGATTTCTTGAACTACTCCAACAAAATATCCCTTTTTTCA

ACTGGTTCCATGCTTATACCATCAAAAAAGGGATTAATTACCCTTATCAAGTCGATGTCA

TAACCTGGCAACTAAGCGATGGAAAAACAATACAATCAGATGTACCTCCCAAGGCACCCT

TTGTGGTTAAAAATGCCCAAAACCTTCGTGTCCTAGCATCCCCGTTCAAAACAAAAAGTG

AGGAAGCAGTTACATGGAAGGATATCAAAGATATCATGGAACAGGCCAACTACACAAACA

AATACCTTCAACAATTAGGTGATGAGGCATATTTCCAACTTTTGAACAAAGGAAAGGTTA

AGATGGAAACTCCCTCTACCTCAGGAATTCAAAAAGAAAATCACAAAGAAAAACCCTTGT

TTAAACCTTACCAATTAACGAAAGGTTCTAAACTAAATCTCCAAAATACCCAAGCCGGAA

AAGATGCAGATAACAAGCTTCTTCGCAAGGTAGTTGAACAACTCAAACTTCTCAATACTG

TTGTCCAAGATACACCACTAACACCAGAAGCATCTGTAAATAACATTGAGGAAGTAAATT

CAACAGGATCTGTTGAAGATTCACCTAAAATCAGTCCTGTCATGCACAATCCAGGAAACA

ATTGGAGAAAAGGAACCAAACTCTATTACAATAGGGCTACAGCTCCTGACTTATTATTAG

AAGAAAAGGATTCTTCTAATTTCAAGAGCTTCAGTGCCAACAATATCTATGAATGGAACA

TAGACGGTGAAAATGAATATGGGATCACCAAAATCCTCCAAAATATGACAATGGTTGCTA

CAACATACTCCACTTCAAACAACTGTCCAGAAACCTTAATTGTAGAAATCCTCGTAGCAG

GATTCTGCGGTCAACTCAAAGGATGGTGGGATAACTATCTCACTGACGATGAAAGACTCC

AAATTCTGACAGCCATCAAACAAGATGATGAGGATAACCCCATCAGGGGAGAAGGTGGAG

AATATACATCTGATGCTGTCAATACTCTCATCTTCACTATAGCCCAACACTTCATAGGAG

ACCCCTCCTTGATCAAAGATAGGTCTGGCGACTTATTATCCAACCTTAAGTGTAAATCTC

TAGGAGACTTTAGATGGTATAAAGACACCTTCCTGACTAGGGTTTACACTAGAGAAGACA

GTCAGCAAGCCTTCTGGAAAGAAAAATTCCTTGCAGGTCTTCCCAAATCCTTAGGCGACA

AGGTAAGGGAAAAACTTAGGAGTCAACATCCAGGCGAAGAAATCCCTTACCAAACCTTAA

GCTATGGTCAGCTTATAGCCATAATCCAAAAAGTTGCTCTAAAAATTTGCCAAGATGACA

AAATCCAGCAGCAACACTCAAAAGAAAAGGCCCAAAATAGAAGGGATTTAGGAACCTTAT

GTGAACAATTTGGATTTGGAGTTCGTTGTGAAAATAAAGACAAACCTAAACCTAGGCATG

ATCTTCCCAAACCTCAGCATAGAAGAAGACCTAGACAAACCTACAACAATAGGAAGCCTA

AACCTAATTCTAAAACTACTCAACCTAGTGAAAAGCCTTATGCGAATAATAACAATTCTC

GCAATAAAACCATTACCTGCTATAACTGTGGCAAGCCTGGTCATTATAGCAAGTACTGTA

GGCTCAAAGGAAAGATTTCTGAACTTAAACTTGACCCTGAATTAGAAGACAAACTCAACA

ATCTCCTTTTTCAATCTTCTGATGAGGAAGAATCACATTCTCCTAATTCAGACAAATCTG

AAGACCTTAATCACATCCAGGATGATGATTGGTCCCAATCTTCCTCTTCTATTAATGTCC

TAACCAATGAGCAAGATTTGCTCTTTACAGCAATTAATGTCATTCCTGACCCAGAAGAGA

AAAGGAAATATCTTGAGAAACTCAAATCTACCTTAGAAAGCAAACCTCCAAAGGGTCCTC

TCACCACCAATAAATTTAATCTTAGAGAAACTTTTAAGAGGCTTGAGAGATCCAGTATTA

AGCATGTTACCATCCAAGATCTCCAATCGGAGGTCAACAACTTAAAAATTGAAGTCAAAA

GTCTGAAGCAACAACAGGCCAGTCAGCAACTCATCTTAGAAAGTTGGATTGAAGAAAAAT

CATATGAAGATGATTCTTTAAACCACAAACCTTCAACATCTAAATTACCTAAAGATGATG

ATAACAATTCTCCTGAAAATGATATTGAAGATTTCCTGCAAATAATTAATACTGTTACAA

TCCAAAAGTTCTATATTGATGTCAAAATCATCATAGGAGATTTTAGCTTTAAAACTGCAG

CTCTTTTTGACACTGGGGCTGATTCCAGCTGCATTTTAGAAGGACTTGTCCCCACTAAAT

ATTTTGAAAAAACTACTGAAAAGCTCAGTGCTACTGAAGGTTCTAAGCATATTATCAGAT

ATAAAATCTCTTCAGCTATCATTCAAAATGAAAACCTTGAAATAGAGACATCTTTCTTGC

TTGTCAAAAATCTGAGTAGCAGGGTAATTATTGGAACACCCTTCATAAAAAAACTCTTCC

CTTATATGACAACTAAAGATGGCATTACCGTCCAACATCTAGGACAACCTATTACTTTCA

AATTCTCCAAACCCCCGATTATCAAGAATTTAAATGTCATCTCATATAAAGAAAGACAGA

TTAATTTTCTCAAAGATGAGATTTCGTTCAAAAATGTTGAAACCCAATTACAATATTCCT

CGATCCAAGCTAGGATTGAAAACCTTCTCAAAGAAATCCAATCTTCCATTTGTTCGGATC

TACCAAGCGCTTTCTGGGAAAGGAAACGCCATATGGTTGACCTCCCTTATGAAAAAGATT

TCAATGATAGAAAAATTCCCACCAAGGCTAGGCCAATCCAAATGAACGAAAAACTTCTTC

AATTTTGTCAAAAGGAAATCAATGACCTATTAAGCAAAAAATTAATCCGCAAAAGAAAGA

GTCCATGGTCTTGTTCAACTTTTTATGTTAACAAGCAAGCTGAAATAGAAAGAGGAACTC

CCAGACTTGTCATTAATTACAAACCACTTAATCAAGCCCTCGGATGGAATAGGTACCATA

TCCCTAACAAAAAAGATCTTCTTGCAAGACTACATGATGCTAAAATCTTTTCTAAGTTTG

ACACGAAATCAGAATTTTGGCAAATTCAAATCAAAGAAGAAGATAGGTATAAAACCGCTT

TTACTGTCCCTTTTGGACAATATGAGTGGAATGTTATGCCATTCAGATTAAAAAACGCCC

CTTCCAAATTTCAGAAGATCATGAATGATATATTTAATCCTTATTCAAAATTTACAATTG

TCTACATTGATGATGTTCCAGTCTTCTCCCAATCCATTGATCAACATTTCAAACATATCA

ACATTTTCATCTCCATTATCAAGAAAAATGGTCTTGCTGTTTCCAGATCAAAGATTAGTC

TCTTCCAAACCAGAATCAGATTCCTTGGACACAATATCCACCAAGGAACTATAATTCCTA

TCAATCGTGCCATTGAATTTACAGACAAATTCCCTGATCAAATTATTGATAAAACTCAAT

TACAAAGGTTTTTGGGTTGCCTCAATTACGTTGCTGATTTCTGCCCACAGATCAGCAACC

TCATAAAGCCCCTTCATGATAGACTCAAGAAGGATCCTCCTCCATGGTCGGATATCCATA

CCAATGTTGTCAAACAAATAAAGGCTCAAGTCAAAAGTCTTCCTTGTCTTTATTTACCCA

ATCCCCAAGCTTTTAAAATTGTTGAAACTGATGCTTCTGATATTGGCTTTGGAGGTATCT

TGAAACAAAGAATTTTAGACAAAGAACAAATTATTACTTTTACTTCAAAACACTGGAACC

CTGCTCAGCAGAATTATTCTACTGTCAAAAAAGAAGTTTTAGCAATTGTTTTATCCATCT

CAAAATTTCAATCGGATTTGATAAATCAAAAGTTTCTAGTCCGCGTAGATTGTAAATCTG

CCAAGGATATATTACAAAAAGATGTTAAAAATTTAGCTTCAAAACAAATTTTTGCTAGAT

GGCAAGCTATTTTAAGTGTTTTCGATTTTGACATAGAATATATCAAAGGCTCAGCAAACT

CTCTCCCTGACTATCTCACCCGTGAATATCTACAAGGAAGAGGCTAATATGGCCTCCAGA

ACCTCTTCCTCCAGAGGAAGGGGAAGCAGCAAATCCAGAGGTAAGAATATTTCTTTACAT

CAAAATGGGTCATCCACCCTAAACCAATCTGGGTTATCCACCCAAAGCTTTGTCCAATCC

AAACAAACTAAGGAAGACTATGCCTTCCCAATCCAAACCCTTCAGGCCTTCCAAGAACAA

GGTCTAACCACACTTCCAAAAAAGACTTGGGCTAGCATTGCAGATGATGATAGTTACCAA

GACCTACCATCACTCCAAACAATAATCCAAACCCAGAAATCCCAAGTCATTCAAGCCAAT

CCCAAACCAATCCAATCAACCCTTCCCAGACAAGAATATCTCAAAAAACCAATTTCCAAA

TTTGTCATGTTAATCGAACCAGAGTATTGGGACCAAACTAGTGGTGAGCTCGTAGCCGAT

AAAACTGCTTCAAAAGTTTTTCCGACATCAAGCCACCTTGAACCAATCAGCCACAACAAA

ACCCAAAACTTCTATGAGTTTATTCTTGTAGATACAGATAGTGTGGCAATCAAACACTTT

AGGGACAAAAATGATGAATCCCTAATAACCCATTCAACTTTCCAAATCATGAGAGTTCTT

AAACCTTCAGACTTTGGTAGCAATCCCAACAAAACCAAGAAATTCTCCAAGAACTTTGAC

CCAATAGGTTTTAATTATTGGGACTATGTCAGAGCCTGGGAAAATACCATCCTCGGCTTC

CAAAATAAGAATCTCAAACATTCCTGGCTTATCTATTTCAAGAAAGCCACCAATTACTCT

TTTCCAAATTGGTTCCACTCTTGGTGGGATTTCTTTGGACCTACAAAAGATATTTTTCCC

CCCGAAGTACTGGAGGGATTCAAACTTTTTTCAAAAAATTGGAATAAAGATTTAAACAAC

TATCCGGTTAGTTTAAATTTTTATACAATCTTTTCGCTGTCTTGGATTTTTGCATGGCAG

TACTTCATTAAAACCAAAAATATTCCTGAGCTCCACAAGCAAACCTCAATCAAATGGTGG

AAAACCTTTGATGCTTCGATGGTTTCAAAAGAAAATATTGATCTTTGGTTCAGAACAAAT

CAGAAATATCTGAATCATGCAAGTCCTCAATCTTGCCTCCTGCTCAACCAGAAGGCTCAC

ATCACTGCAGCACTCGCAGGTGCCTCTTCAGAGGAAGGTTTATTGAAAAGCCTCCAAGTT

GCCCTTCAAATACTACAAAATAATTCAAAAGACAATCAAGCATCTTCTTCAAAGGACCCT

TTGCAGAAAAAAGAAAAGCATCGGATACCCTCTCAATCAGAATCTGACGCCAGTTTTGTC

CCAGACAGCCTGGAAGACAGTGAAGAAGATCTCCTTTGATTTTCATCTCAATAATTATAC

TTTTTACTGTAGCAAAGTTGACTTTTCACTGTAGTTAGTACCGAACAGTTCCTGTCGATA

ACCGGAACTATTCAAACAGTGTTTGTATTTTTCCTATAAATAGAGAGCCCCTCTTATGTT

AGAGGGCACACTTTAGACGGAGAGAGATAGTCAGATCCTAGAGAGATAAACTGTAAGTCT

TATCTCTTCTTGCTTTCTCTTTTCAATCCGTTTCAGTTTTTCAATCTGTTTCTGTAATTT

CAATCCTTTTCAGTTTTCAATAATATCAATCTTCTGCAATCTTGTTTAATGGACGGCTAA

GCCTCCTGCTTTATTTTATTCTGTCAATGGAAGGCTGAGCCTCCTTTTAATTTATTTACT

TACTGCGCTTTTATTATTCCAGTCAATCTTTATATTCTGCAGTCTGGCTGCATCCTACTT

CTCTTACCAGCTTTAATTCTAACCCCTTAAATCTGCTTCCGAACCCCTC

>LjapCV_sc1 [endogenous-virus-name=Lotus japonicus C virus] [strain=sequence cluster 1] [host=Lotus japonicus accession Miyakojima MG-20] [moltype=genomic DNA] 7205 bp

TGGTATCAGAGCCTTTTCCCGTGAGGGAAGGGTTCTGGAATCAGTAGTTAGTTTTTGTTG

GATTCAATCTTTTGTTTTTCTGCAACTTTATTTCTTCTTACTGTTTTTCATTAATCACCT

TGTTATTATCTTCAAGTCCTTAAGGCTTAGTGTGAGGTATCCCTGGAGTCTAGTGGTCCC

GGCAGTAAAGCCTGAGAGGTTGTGAGACCGTTGGTTTAGCTAGATTGGGAAGAAGCGTTA

TCCTTCTTAAGTTACTTAAGTTAATAACATGGCTAGATTAATCAAAACCCTAAGTGAAAT

AAGTTTAGGAAAAAACAAACAGATCATGATTCCATCAACATCCAACTCTGGTTCTAGTAA

CCAAGATCAAGAACTCTCTAGGGTAGCCCAACAAGAGTTAGAAATAGCTTCAATAGAAAG

AGCACTTCAAAACTGGTCAATCCCGATAGTTAAAAAGAAAGAAATATATAAACAACACAC

TTTATTCAATAGAGCAGATGATTCTATCTTTACAATAGAATGTTGTCAACATAGTTCTGA

TCACACAAGTACTATAAAATTATTAAATGAAGATGTTCTAGATCAACACATTAAAAATGG

TTTTAACTTCATCCATGTAGGTTTAATACAAGTAGCTGCAAAACCTAACTTTCGTTTAGG

AATCAATTCTCCTATAATAGTAATGTTAAGAGATATGAGGCTATTAAAGCCACAAGATTC

GTTAATTGCAGTCTTAGAGTCAAATCTTCATGATGGTCCTGTTTTCTTTAACTGCTATCC

AAACTATGCTATGAATCTTCAGAATAGTTGGACCAAAAATGCCATACAATTAGATTTATT

GGCAAACAATGATATTTTTGAAGAAGAAAGTGATCCTTTCTCAATAATATATAGAGTATA

TTATAAAGTCTCAAAAATCAATTATAATTTTAAAGCTTTAAGATCTTCACCCAAACAAGA

AACCATTATGCTAGAAGCAAACCTTAAAAGATCTTCAGTACAAGTTCCAAAGAAACTTAC

TCATGAAGAAGTCATGAGTAAAGTCCCTGAGGAATGGGTATTCAAGAATTCCCTGCCTCA

ACCAAAAATTCATACTACTCAAGTAAGAGAATTGTTTCAAGAAGGAGCAAATCTCACTTT

AAGAATGAATAGATCAAACTCATTTAGTATTAGATCTCCTCAACAGTTATACAGAGTAGA

TCTCCCAAGATCTTCTGTATCATCAAAACTTAAAGGATTAGATACAACGTCTAGTCCTAA

TATAGCAACACCTATATATCAAGATGAAGAATCTAGTAATTATTCTCCAACCCCTTCGCA

AATCAACATGCTAAGTAGGGTAAAACCTAGTTTTGAAATAGACAAAGATTTCATTAAAAA

AGATTTTATGGCCGAATACAATAAAGATAAAAGATTATGGTACTTCAAGAATTACAGTAA

ACAAGAAACTGAAACTCTTAGGGCCTTTTATTATGAAGAAATGGAAGTTTCTGAAACAAA

CATCTATTTTTTTGATTGGTTCGAAAACCATTGTATTAAAAATAATCTTAATTATCCTTT

CTGGAAAAACGTCAATCCAATAACAAAAATAAATACCACTTGGAAAACTCCTGATAATAA

CACTATTACTTCAGAATACCCTCCACAAACTGGAGTGAAAATAGTCATAAAAGATGGTCA

TGAAATCGAAGCATCTCCTTATAAGGCAGAAGCTAACAAAGTTCATCAACAATTAAATTT

TGCAAACACTATGCTAACAGTCATGTCAAAACAGCTAGAAAGAATAGAGGTTCAAAAACC

CTCTATCACAACCAAAGAAGCTTCTTCATCGTTTACCTTAGGAAACCCTTTGCAAACTCC

TATTTTCAAAATTCCTCAATACACTAAAGAAGAATTTAATTCCTTTAATCTCTCAGGAAG

TGTAGAAAAATTAAAAGAAAAATTAAACAAGCTTTCAATAAATCATTTGGATAAAGATTT

AAAAATCAATAAAATTAGAAAGTTTGGGCCAGATAACAACACAAGAGCCTACTATCCAAG

ACCTTCCTATTCTGATATGAGGTTTGAAGAAAGAAAAGAATTTATTCAAAATACCTTTAG

TGGAACTAGCCTTGATGAATGGAATATCGATGGCTTTAGTGAACAAAATATATTAGATAT

TACGCATCAAATGATTATGGCTGCCACAGCCTATAAAGTCCATAATAACACCGACAGAAA

TGCAGCATTAATGATTACTCATGGCTTCACTGGTCAGTTACGAGGCTGGTGGGACAACAT

CATGACCCCTGAAGATAAATCAACTATATTAGAAAAGAAAAAAGAAAATGGAGAAGAAGA

TGCTGTAGCAACATTAATATATACTATTATACTTCATTTCATAGGAGATCCTAGCATATT

TAGAGAGAGAGCTTCAAGCCAGCTCGCTAATCTCTATTGTCCTACCATGTCCGATTACAG

ATGGTATAAGGACACCTTCTTCTCAAAGGTAACCTTAAGAGAAGATGGCAACAGCGCCTT

CTGGAAGGAGCGCTTTATTGCAGGATTACCTAGATTAATGCAATCAAAGGTATTAGATAA

TTTATCACTTTTTAATCAAGGAAATCCAGTAAATTTTGGTTCACTCTCATTTGGACAATT

GCATAACACTATAGTACACACAGGAATACAAGTTTGTACTGACTTCAAACTTCAAAACAA

AATGCAAAAAGATATGCAAATCTCAAGAAAAGAAGTAGGTAGCTTTTGTGAACAATATGG

AGTAGAACCTTTACAAGCTCCTAGTTCAAAAGCTAGAAGGATTAATAGATCCAAAACAAA

GCCTTCTCAGGGATATAAAAAGAGAAAACCATTTCAAAAGAAACCTTATGAACAAAGTCC

TACTACTCAAAAACCTATAAACAAAAAAGCCGTAAATCCTAATAAAAAGAAAAATGTCTG

CTGGAAATGTGGAAAGCCAGGACATTATGCAAACAAATGCAAAACTCAACAAAAAATTAA

TGAATTAGATTTAGACCAAAAACTCAAAGATAGTCTAATCAGTGTTCTCATTAATAGTGA

AGATCCTCACTACTCTTCAGAAAATGAATATGAAGGAGATGAAGACCAAGATGTAAATCT

GGTCGAATACTCATCTGATTCTCATTCCTCTTCAGAAGAAGAAAGAGAATGTGTTAAAGA

TAGTTCAGGATTCTGTGATTGTGCAGGATGCTTAGGACAAAACGTCAATATGCTAACCCA

AGATCAAACTATGAGTTTAATCTCTATAATAGACAAAATGGAGGATTCTCCCTTAAGGGA

CGAATTTCTACAACAACTAAATCTTTTGGTCAAGAAGGAAGAAACCTTGAAAAAGGAAAT

TAGCCCTCCAATAAATAGCATGAATGAAATCTTTAATAGATTTCGTCCTAAACCAACCAT

AACCTTATCAGATCTTCAAGAAGAAGTGAAAATATTAAAAGAAGAAGTTAACCAACTAAA

GCAAAATGATCTTAGCTTAGAGTTCAGATTAATGGAAATAGCTGGGATAAAAATTATAGA

ATCTCAAGAAACCTTAGCCAGTACCTCAGGATTAAAGCAAATACATAATGAAGAAGAAGA

AGAACCCAGTGAAAGTTATCTTAATCTCCTCAACTTGGTTATAACCCATAAATGGCACTC

TGAAATAACTTTAGTCATTAATAATGATTTTCGAATCAATATAATAGCCCTCATTGATAG

TGGCGCAGATATCAATTGTATTCAGGAAGGCTTAGTCCCCACTCAATTTTATGAGAAAAC

CAAAGAAGGAGTCAATAGTGCAAATGGATCCAAAATGAATATTCAATACAAACTATCAAA

TGCTAAAATTTGCAAAAGCCAAGTTTGTTTTAGATCTTCATTTGTCCTAGTCAAAAACAT

GACAGAAAAAATCATTTTAGGAACTCCATTCATTTGTCTTCTATACCCTATTGATAAAAT

AGATGAAAAAGGAATAGTCACAACAGTCTTAGGAAAGACTATTACTTTTCCATTTATAAA

ACCTCCTCAGGTAAAAGAATTAAATTTATTAAAGGAAATCTCCACCTCCAAAATAAATAT

AATCTCTAGAAAAAAGAATCATATCAATTCTTTACAAAAAGAAATAAAATATTATAAATT

AGAAGAACAGCTCAAGAGATCCAATATTCAAAAGATTATTAAGGATTTTCAAGATAGGAT

AGAAAAGGATCTATGTGATTTAAATCCCATGGCCTTTCATCATAGAAAAATACATACCAT

AACATTACCATACATAGACGGGTTTAATGAGAAAGACATCCCTACCAAAGCCCGGCCCAT

TCAAATGAATGAACAATATTTAAAATATTGTAGAGAAGAAATAGGAGAATATCTCAACAA

GGGCCTGATTCGCCACTCCAAATCTCCTTGGAGTTGCACAGGGTTTTATGTCATGAATGC

ATCTGAGTTAGAAAGAGGAGCCCCTAGATTAGTCATTAATTACAAACCCTTAAACAAAGT

ATTAAAATGGATAAGATATCCATTGCCTAATAAAACAGATCTCATTAAAAGATTACATAA

AGCAACAATATTCTCAAAATTTGATATGAAGTCAGGATATTATCAAATTTCGGTCAAGGA

AGAAGATCGATATAAAACCGCCTTCGTTGTCCCTTTTGGTCATTATGAATGGAATGTAAT

GCCACAAGGTTTGAAAAATGCACCTAGTGAATACCAAAACATCATGAATGATATATTCTA

TCCATACATGGACTTTACCATTGTATATCTAGATGATGTCTTAGTATTCTCAAAAGGCAT

AGATCAGCATGTTCAACATTTAGAAAAATTTATAGAAATCATTAAGAAAAATGGATTAGT

AGTTTCAGCAAAAAAGATGAAAATCTTTGAAACCAAAACCAGATTTTTAGGATATGAAAT

TCATCAGGGACAAATTACCCCAATACAAAGATCGTTAGAATTCGCCAAAAACTTTCCAAA

TGAATTAAAAGAGAAAAATCAATTACAAAGATTTCTAGGTTGTGTTAATTATGTAGCAGA

TTTTATCCCCAACATAAGGATAATTTGTGCCCCTTTGTTCAAAAGACTTAGGAAAAACTC

TCCGCCATGGTCTGAAGAAATGAGCCATAGTGTTAGAGAAGTCAAAAGATTAGTTCAAAA

CCTACCTTGTTTAGGAATACCAGATCCAGATGCTTCATTAATTATAGAAACCGATGCATC

AGAATTAGGATATGGAGGGATACTTAAGCAGGTAAAACCTCAGTCTTCAAAAGAACAAAT

AGTTAGGTACCATTCAGGTATTTGGCACCCAGCTCAACAGAAATATTCCACAGTCAAAAA

AGAAGTTCTTTCAATAGTTCTATGTGTTCAAAAATTTCAAGATGATGTTTTTAATAAAAA

AAATTTGATAAAAACTGATTGCAAAGCAGCACCTTCAGTTTTACAAAAGGATGTTCAAAA

CCTAGTTTCAAAACACATTTTTGCCAGATGGCAATCTTTACTTTCTTGCTTTGATTTTGA

AATAACTCATATTAAAGGAGATAGTAACTCCCTTCCAGATTTTCTAACCAGAGAATTTTT

ACAGGGAAAACATGAGTGATACAAAACCCAAATCAAAGGATCAATATGGACCCCCACTGG

GTTCGTCAGCATCATCATCACCATCAGGATCAAAAGCAATAGTAGTAACCCCTATCAAGG

TTGCTGAATCTTCAATACCAACCACCCCTTCAAAGCCTTCTCAAAAACTCACCACAGCTC

AAATTGTCAAAACACCTCACCAAAAAACCTCACTAGTCCCATTAACCAATAAATATACAG

TATTAGCTCAAAGCCCTTCAAAATCCCCTGCCAAACCCGAGCCAACCGAATATCTGGAAA

AACCAGAAGGTGAGCCATCTCTCATTATAGAAAGAGAACATTTCTCTTTAAGTCCTAGAG

AAATAGCCACTCAATTATTCCCTACCAACTTTCATTATGTCCCTGGACACCCCAAGAAGA

CCAGATTATTTTATGAATTCATTCTAGTAGACTCTGACTCCATAGAAGTCACACATAACC

AAGATAAACAAGGAGAAATAGCGTTCTCCAAGATCAAAATCCTTAAAGTCCTTACACCCC

AGGATTGGAATGCTCCTCTTCATTATTTTAAGCAGTTTTCTAGACAGTTCGACCCTCCTA

GTTATAACTACTTTGATTATACAGATGCCTGGTATTACGCCTTGTATCTTTATCCTTATC

AACACTCCTGGTTCATTTGGTTCAAAAAGGGAATCTCTTTAAAGTTCCCTCAATGGTTTA

AAGTTTGGTTTTGCAAGGTAGGACTCGATGAGTCTATCTTTCCCGAAGAAGTAAAACCAT

TATTCAAGTACTTTGCTCAAAAGTCAAATTTCATTATGGAAGACAAACTCCTCATGTTCA

CAGCTTCGCAAGCCATATCTTGGATTTTAACCTGGGATTGGAGAACTGTAGAAATTTATG

AGGATACAGAACTCTATCAGCTCTATCGCATTTTTAAAATAAAATGGTGGGCAAAATTCA

ATATCTCTCTAATTCAACAGACAAAACTTGAAGCTTGGATAAAGACTTATCAGCTTACTC

AACAAAGCAAGAAACAGCTGTCAACTACCCCTGTTTCTCGTCTCAATCAAGAAAGTTCAT

TTCTTCAAGAAAGATCAAGGCTCATAGCCGAATTAGCCGCAGCAAGCTCTCCCCAAGAAT

TTCAAAAGAAAATAGATATGATGAGTCAGAAGTCTGGCTCAGATACTTCATCAGTCAACA

GCCAGAACTACCTTCAAGAAAATGAAGATGACTGTTTAGGCATCAACTATCATCCATACA

CTTAAGGACAAAAGGTCATTATCAGCCCTTCTCATCATGATCAAGAAAGAATCTGCTTTC

TAATATTGGCCGACAAGGAATCCACTTTCATTATTAAAGCTACTATCCACTTTCATTATT

AAAGTTACTTTTCATTAACTGTAGTTACTTTTATGTAAACCAGAGTTACTTTCGAAAGTA

ACCATGGTCAAAACTTGTATAAATAGGTAGCTTAGTAAGAGTAAGAGGCATCGGGTTTTT

GACCTACCTCTCTCTCTCTCTCTCACCTACCTCTCTCTCTATGTGTGTAATCCCTGCAGT

TCTTTGAATAAAAGCTTTTCTGTAAGTACTTTTCCATCATGTTTTTTTTTAGCTTCCGTA

AATTC

>LjapCV_sc2 [endogenous-virus-name=Lotus japonicus C virus] [strain=sequence cluster 2] [host=Lotus japonicus accession Miyakojima MG-20] [moltype=genomic DNA] 7298 bp

TGGTATCAGAGCCTTTTCCCGTGAGGGAAGGGTTCTGGAATCAGTAGTTAGTTTTTGTTG

GATCAATCTTTTGTTTTTCTACAACTGTATTTCTTCTTACTGTTTTCATTAATCACCTTG

TTATTATCCTCAAGTTCTTAAGACTTAGTGTGAGGTATCCCTGGAGTCTAGTGGTCCCGG

CAGTAAAGCCTGAGAGGTAGTGAGACCGTTGGTTTAGCTAGATTGGGAAGAAACATTATT

ATTAAGATTCTTAAGTGTATAACATGGCTAGATTAATCAAAACCCTAAGTGAAATGAGTT

TAGGAAAGAACAAACAGATCATGATTCCATCAACATCCAACTCTGGTTCTAGTAACCAAG

ATCAAGAACTCTCTAGGATAGCCCAACAAGAGTTAGAAATAGCTTCAATAGAAAGAGCAC

TTCAAAATTGGTCAATCCCTATAGTCAAAAAGAAAGAAATTTATAAGCAACATACTTTAT

TCAATAGATCAGATGATTCTATTTTTACGATAGAATGTTGTCAACATAGTTCTGATCATA

CAAGTACTATAAAATTATTAAATGAAGATGTTTTAGATCAACACATCAAAGATGGTTTTA

ATTTCATTCATGTAGGTTTAATACAAGTAGCCGCAAAACCTAACTTTCGTTTAGGAATCA

ATTCCCCAATAATTGTAATGTTACGAGATATGAGACTATTAAAACCACAAGATTCACTAA

TCGCAGTCTTAGAGTCAAATCTTCATGATGGTCCTGTCTTTTTTAACTGCTATCCAAATT

ATGCCATAAATCTTCAAAATAGTTGGACTAAGAATGCTTTACAATTAGATTTATTAGCAA

ATAATGACATATTTGCAGAAGAAAGTGATCCTTTCTCAATAATATATAGAATATATTTTA

AAGTTTCAAAGATTAATTATAATTTTAAAGCCTTAAGATCATCACCAAAACAAGAAACCA

TAATGTTAGAAGCAAATTTAAAAAGATCTTCAGTACAGGTTCCTAAGAAGCTTACTCATG

AAGAAGTTATGAGTAAAATTCCTGAGGAATGGGTGTTCAAGAATTCACTACCTCAACCAA

AGGTTCATAATACTCAAGTAAGAGAATTGTTTCAAGAAGGAGCAAATCTCACTTTAAGAA

TGAATAGGTCAAATTCATTTAGTATTAGAACTCCTCAACAGTTATACAGAGTAGATCTTC

CAAGATCTTCTGTATCATCAAAACTTAAAGGAATAGATACAACGTCTAGTCCTAGCATAG

CAACACCTATATATCAAGATGAAGAATCTAGTAACTATTCTCCAACCCCATCTCAAATCA

ACATGTTAAGTAGGGTAAAATCTAGTTTTGAAATAAATAAAGATTTCATTAGAAAAGATT

TTATGGCTGAATACAACAAAGATAAAAGATTATGGTACTTCAAGAATTTTAGTAAGCTAG

AAACTGAAAATCTTAGAACCTTATATTATGAAGAGATGGAAGTTTCTGAAACAAACATCT

ATTTTTTTGATTGGTTCGAAAATTATTGTATTAAAAACAACCTTAATTATCCTTTTTGGA

AAAACGTCAATCCAATAACAAAAATAGATACTATCTGGAAAACTCCTGACAATAATACCA

TTACTTCAGAATACCCTCCACAAACTGGAGTCAAAATAGCAATAAAAGATGGTCAAGAAA

TCGAAGCATCTCCTTATAAGGTAGAAGCTAACAAAGTTCATCAACAATTAAATTTTGCTA

ATACCATGTTAACAGTTATGTCAAAACAATTAGGAAGAATAGAGGTTCAAAAACCTGAAC

CATCTACACCCAAAAAATCCCCTTCATCATTTACTCTAGGAAACCCTTTACAGACACCTA

TTTTCAAAATTCCTCAATACACCAAGGAAGAATTTAATTCCTTTAATCTCTCAGGGAGTG

TAGAAAAATTGAAAGAAAAGTTAAATAAGCTTTCAATAAATCATTTAGATAAAGATTTAA

ATATCAATAAAATTAGAAAGTTTGGTCCAGATAACAACACAAGAGCCTACTATCCAAGAC

CTTCCTATTCTGACATGAGATTTGAAGAAAGAAAAGAATTCATTCAAAATACCTTTAGTG

GAACTAGCCTCGACGAATGGAATATCGATGGCTTTAGTGAACAAAATATACTAGATATTA

CTCATCAAATGATTATGGCCGCCACGGCCTATAAGGTTCATAACAACACAGATAGAAATG

CAGCATTAATGATTACTCATGGCTTCACTTGTCAGTTACGTGGCTGGTGGGACAACATCA

TGACCCCTGAAGATAAATCAACCATATTAGAAAAGAAAAAAGAAAATGGAGAAGAAGATG

CTGTAGCAACATTAATATATACTATTATACTTCATTTCATAGGAGATCCCAGCATATTTA

GAGAAAGAGCTTCAAGCCAGCTCTCTAATCTCTATTGTCCTACCATGTCCGATTACAGAT

GGTATAAGGACACCTTCTTCTCAAAAGTAACCCTAAGAGAAGATGGCAACAGCGCCTTCT

GGAAGGAGCGCTTTATTGCAGGATTACCTAGATTGATGCAATCAAAAGTATTAGATAATT

TATCACTTTTCAATCAAGGGAATCCAATAAACTTTGGTTCACTATCATTTGGACAACTTC

ATAATACTATAGTACACACAGGAATACAAGTTTGTACTGACTTCAAACTTCAAAACAAAA

TGCAAAAAGATATGCAAATCTCAAGAAAAGAAGTAGGTAGCTTTTGTGAACAATATGGAG

TAGAACCTTTACAAGCTCCTAGTTCAAAAGCTAGAAGGATTAATAGATCCAAAACAAAGC

CATCTCAGGGCTATAAAAAGAGAAAACCATTTCAAAAGAAACCATATGAACAAAATCCTT

CAACTCAAAAACCTACAAACAAAAAAACTGTAAATCCTAAAAAGAAAAATGTCTGCTGGA

AATGTGGAAAGCCAGGACATTATGCAAACAAATGTAAAACTCAACAAAAAATAAATGAAT

TAGATTTAGACCAAAAACTCAAAGATAGTCTAATCAGTGTTCTCATTAATAATGAAGATC

CTCACTACTCTTCAGAATATGAATATGAAGGAGATGAAGACCAAGATGTAAATCTGGTCG

AATACTCATCAGACTCTCATTCTTCTTCAGAAGAAGAAAGAGAATGTGTCAAAGATAGTT

CAGGATTCTGTGATTGTGCAGGATGCTTAGGACAAAGTGTCAATATGCTAACACAAGATC

AAACTATGAGTTTAATCTCTATAATAGACAAAATGGAAGATTCTCCTTTGAGAGACGAAT

TCCTACAACAACTAAATCTTTTAGTCAAAAAGGAAGAAACCTTGAAAAAAGATATTAGTC

CTCCAATAAATAGTATGAATGAAATCTTTAACAGATTTCGACCTAAGCCAACAATAACTT

TATCAGATCTTCAAGAAGAAGTGAAAATATTAAAAGAAGAAATTAATCAACTAAAGCAAA

ATGATCTTAGCTTAGAATTTAGATTAATGGAACTAGCTGGAATAAAGATTATTGAAACCC

AAGAAACTTTAGCTAGTGCTTCAGGAATAAAACAAATTCATGATAAAAATAATGAAGATA

ATAATAATCAAGAACAAAATGAAAATTACCTTAATCTTCTTAATATAGTTATAACTCATA

AATGGCATTCTGAAATAACCTTAGTCATTAATGATTTTCGAATCAATATAGTAGCCTTAA

TTGATAGTGGCGCAGATATCAATTGCATTCAAGAAGGGTTAATTCCTACTCAGTTTTATG

AAAAAACTAAGGAAGGAGTTAATAGTGCAAATGGATCCAAAATGAACATTCAATACAAAC

TATCAAATGCCAAGATTTGCAAAAACCAAATCTGTTTTAAATCTTCATTTGTACTAGTCA

AAAATATGACAGAAAAGGTCATTTTAGGAACTCCGTTCATCTGCCTATTATACCCTATCG

ATAAAATAGATGAAAAAGGAATAGTTACACAAGTCTTAGGAAAGACTATTACTTTTCCAT

TTATAAAACCTCCCCAGGTAAAAGAATTAAATTTATTAAAAGAAATCTCCACCTCCAAAA

TAAATATGATCTCTAGGAAAAAGAATCATATCAATTCTTTACAAAAAGAAATAAAATATT

ACAAAATAGAGGAACAACTCAAGAGATCTAATATTCAAAAGATTATTAAGGATTTTCAAG

ATCAAATAGAAAAGGATCTATGTGATTTAAATCCCATGGCCTTTCATCATAGAAAGATAC

ATACCATAGCATTACCATATATAGACGGGTTTAATGAAAAAGATATCCCTACAAAAGCCC

GGCCCATTCAAATGAATGAACAATATTTAAAATATTGTAGAGAAGAAATAGGAGAATATC

TCAATAAGGGTCTGATTCGCCACTCCAAATCTCCTTGGAGTTGCACAGGGTTTTATGTCA

TGAATGCTTCTGAGTTAGAAAGAGGAGCTCCTAGACTAGTCATTAACTACAAACCCTTAA

ACAAAGTATTAAAATGGATAAGATATCCATTACCCAATAAAACAGATCTCATTAAAAGAT

TACATAAAGCAACAATATTCTCAAAATTTGATATGAAATCAGGATATTATCAAATTTCGG

TGAGGGAGGAAGATAGATATAAAACCGCCTTTGTCGTCCCTTTTGGTCATTATGAATGGA

ATGTCATGCCACAAGGCTTGAAAAATGCACCTAGTGAGTACCAAAACATCATGAATGATA

TATTTTATCCATATATGGATTTCACCATTGTATATCTAGATGATGTCTTAGTATTCTCGA

AAGGTATAGATCAGCACGTTCAACATTTAGAAAAGTTTATAGAAATCATTAAGAAAAATG

GATTAGTAGTTTCAGCAAAAAAGATGAAAATCTTTGAAACTAAAACTAGATTTTTAGGTT

ATGAAATTCATCAAGGGCAAATTACCCCAATACAAAGATCATTAGAATTTGCAAAAAAAT

TTCCAGACGAATTAAAAGAGAAAACTCAATTACAGAGATTTCTAGGTTGTGTTAATTATG

TAGCAGACTTTATCCCCAACATAAGGATAATTTGTGCCCCTTTGTTCAAAAGACTTAGAA

AAAACTCCCCGCCATGGTCTGAGGAAATGAGCCATAGTGTTAGGGAAGTTAAGAAATTAG

TTCAAAGTCTACCTTGTTTAGGAATACCAGATCCAGATGCCTCATTAATTATAGAAACAG

ATGCATCAGAATTAGGATATGGTGGGATACTTAAACAGGTAAAACCCCAGTCTTCAAAAG

AGCAAATTGTTAGGTACCATTCAGGTATTTGGCACCCAGCTCAACAAAAGTATTCCACAG

TCAAAAAAGAGATTCTTTCAATAGTTCTATGTGTTCAAAAATTTCAAGATGATGTTTTTA

ATAAAAGATTCTTGATAAAAACTGATTGCAAAGCAGCACCTTCAGTTTTACAAAAGGATG

TTCAAAACCTAGTTTCAAAACATATTTTTGCTAGATGGCAATCTTTACTTTCTTGCTTTG

ATTTTGAAATAACTCATATCAAAGGAGATAATAACTCCCTTCCAGATTTTCTAACAAGAG

AATTTTTACAGGGAAAACATGAGTGAAAAACCCAAGTCAAAAGATCAATATGGACCCCCA

CTGGGTTCCTCAACAACATCAAAAGAAAAAGCACTAGCAATAACCCCTCTCAAGAGTGCT

GGATCATCCGCACCAGCCACACCCTCAAAATCCTCTCAAAGACTTACTACCTCACAAATT

GTCAAAACACCTCCTCAAAATCAAAAAGCTCCATTAGTCCCACTGACTAATAAATATACT

GTATTAACTCAACCCTCTTCAAAATCCCCTGTCAAACCAGAGTCAACAGAATATTTAGAA

AAACCAGAAGGCTTGCCCTCTCTCATCATAGAAAAAGAATATTTCCATTTATATCCAAAA

GAGATAGCTACCCAATTACTTCCTCCCAACTTTCAATATGTCCCAAGACACCCCAAAAAG

ACCCGACTTTTTTATGAGTTTATCCTCGTTGATTATGACTCAATAGAGGTTACTCATAAC

CCTGACAAACAAGGAGAGATAGCCTTTTCAAAAATCAAAATCATCAAAGTCCTTTCAGCA

CAAGATTGGAATGCCCCCCTTCATTACTTCAAAAACTTTTCTAGACAGTTTGACCCCCTA

GTTATAATTACTATGACTACACAGATGCCTGGTATAACGCCCTGTATCTTAAACCCTTTC

AACACTCTTGGTTTATATGGTTTCAAAAAGGGATTTCCCTGAAATTCCCACAATGGTTCA

AAGTCTGGTTCTATCAAGTTGGTCTGGATGAATCAATTTTCCCTGAAGAAGTTAAGCCCT

TATTCAAATATTTTGTTCAAAGGACCAACTTCGCTATGGAGGAACAGCTCCTTATGTTCG

CAGCTTCCCAAGCCATCTCATGGATATTAACTTGGGACTGGAGATCTGTCCAAATCCTTG

ATGATACAGAATTATACCAACTTTATAGAATCTTCAGGATAAAATGGTGGACAAAGTTCA

ATATTTCATTAATACAACAAACCAAAATTGAAGCATGGATAAAAACCTATCAGCTTACTC

AAAAGAGCAAGAAGCAATTATCCACTTCACCGGCCCCCTGTCTCTCTCAAGAAGCCTCAT

TCCTTCAAGAAAAATCAAAAATAATGGCAGAACTAGCCTCGGCCAGCTCGCCAGTAGAAT

TTCAAAAAAGATTAGACATGATATCAGGAAGTCAGAAATCAGATTCATCAGCTGAAGACA

CCTCATCAGTCAAAATGGAAAATCACCTTCAAGAAAATGAGGATGACTGTTTTGGAATCA

GTTATCATTCATTCACCTAATCGGCCCCGGGTTTCCATTACAAAAGGACAAAAAGATCAA

CATCAAAACTCCTCATTATGGCAGACAGAGAATCTGCTACTTTTATCATAATCTACTGCT

TTTATCACGTAACCCTAGTTGCTTTTGTAAACCATAGTTACTTTCTAAAAAGTAATCATG

GTCAGAATAACCCTAGTTGCTTTCAAAAAACCAGAGTTACTTTTACAAGTAATCATGGTT

AGTTTAATGTGTATAAATAAGCATCATAGAGCTGAAGAGAAGGCATCGGGTTTTCACCTA

CCTCTCTCTCTCTCTCTCTCTCTCTCCCTCTCTCTCTCTTTCCCCTAAGTATGCTTGTAA

TTTCTGAAGTTCCTCTTTGAATAAAAGCTTGTTTCTGTAAGTACTCTGAGTTTTCATATT

TTCATATTTGCAAAATGTTTTTTATCTTCCGCACCTTC

>MdomV_scMd1 [endogenous-virus-name=Malus domestica virus] [strain=sequence cluster 1] [host=Malus x domestica cv. Golden Delicious] [moltype=genomic DNA] [note=complete genome] 7891 bp

TGGTATCAGAGCCAAGTGGTTGGTTATACCATTAGTATCTGAATACTCTGTTTATACTCT

TTAGATTTATTCAGTCTCTTTATAGTTAATCTTTTCAGTTTAATCTTCTTAGTGTCTTCT

GATCTCCATAAGGTCTGAGCAGTAAGGCGTCACCCAAACAGTAAGTCCCGGAGGATAGGG

CCTGAGCGGCGCGCATGACCCAGAGAGCTTGGTGTTTAGAAGCACTAGTAAGATTTTCTG

TTAAGGTTTCTGTATTTTGATTGTTTGAATTTATTAAGTATGAGTAGATTGTTTAGATCT

AATTCTATGGCCAGCACTAGCTCTAGATCAAATTTAGGAACTATACCTGATATTGTCAAT

GAAGAACAAAAATTTGAATTTCAAACAGACAGTAGTCTAGATTTTGCTGAATGGAATATT

CCAAAAATATCTTCAAAAAATATTTATAAAAAGAAATGGGCATTCGCCTCTTTTAAAACT

GAACACCATGTTAAAACGGTTGAACAAGCTTATGCTCTTAGTAAAGATCATGAAACTTGC

CAATTGTTTACCCCAGAACAAATTAATTCCCATAGGAAAGATGGTCATAATTATATCCAT

ATAGGTTTAGTGCAAATTGCAGCCAAACCATTAACACGTAGAGGTCTTAATACCTCTATC

CTCTTATGTCTTCGAGATGCTAGATTCACTGACTTCAGTGATAGCATACTTGGTATGGTT

GAATCCAGCCTCTGTAATGGACCTATCCACTTTGACTGCTTTCCAGATCTTACCATAAGT

CTATCCGACCCACATATGTTAAAAGCACTTACACTAAATATCAAAACTTCAGGATACAAA

GTCCTTGAAGGAACACAGCCTTTGGCCTTAATTTACAGAATTTATTATAAAGTCACTGGC

ACAAATATGAACTTTCAGGCTTTAACCAAAAGTCCGAAGGATCATACTCTCTTGATCCAG

ACTGATCAAGAGAATGCCAATATTAAAATACCTCGAACCATTAGGTGGTCAGAAATCAAT

CTCCCTTCAGATTGGTGTCTGATAAACGAGAGTCGGCCAGTAGCCATCCAAAATAGTCTA

GTTAATTTAGACAACGTAGAACAATACTTTGACGGTACGGTTAAAATTAATTTTGATCGT

CCTGCTAGGAAATCCTGTGATTCTAATCGATCACTTAAATCCTTTACTTCCAATAGGAAT

TCTTTCTCTGGATCTATGCCAAACGATAGGCAAGGTCGAGACCAAGAATTAATCAATTCC

CTAGCTAATAGGAAATTAAAATCAGTAGATACAGGCTCATCTGTCACCCAACAAGAATTA

ATAAATGACCTTTTAAATTTCAAACTCCAATCACTTGAGACAAACGCTCAAGTAGTCCAG

CCTACCTATCATACCCCATCCATTCCTCCTAAGGAAGAACAAACATCCCCAACTGCTTCT

GATTTTGATGCAGAACCAGTACATCACCAACTATTGGTTTTAACCAGACAAGAACCTTTT

AAATATGATCGTAAAAAACTCTGTAGAGACATAGAAGCCTTAGAAAATGTCCCTAGACGA

AACATCTTCAGAAGTAAATATACTACTGAAGAAAAATTAGAAATCCATAAGGCATGGAAA

GACTTCATGCAAACCAACAGACTTGAAATATTTTTCTTCGACTTCGTAGAAAAACATTAT

GAGTCTGAAAACCAAGTAAAGGTAATAACCAAAGAAAATTGGCTTAAAGAAGACAAGACA

ATTGTCTCCTCTAGTCACCCTCCACAAGAAACTATTCTTATTAGTACTGCGAATACGCAG

GTCCCAGCCTCACCGTTCAAACTTCCGAAGGAAGATGCAGGTGTCAAACCTGTTATTGAA

CAAAACAATTTTACCAACCAAAGCCTACATATCATAGGAAAACAGTTGGATAAAATTGAA

ACCAAGGTTGACAATTTGTCAATTAAACCACTTAGTAAAGAAATACCTTTAACTAGTTTT

AGAGAGTCACCCTCTTTAAAAACCTCAACATCTATTAAGATAGAACAAATGCTTAATAAG

CTAGAAAAGGACAAATCAGTTCGTGTGATTAATAATCCACATGATTCTGAAACACCTTCC

TCAATTCCAGATTCAGAAGATGAAAGTATTGATTCCATCCATCAAATGGAACAAGCCTTT

CAGAGTCTTGAATTAAAACGTCTTAACGTTAAAAGGACTAATCCTACATCCTTAACCAAA

AACTGGTATCCCAGACCAACACCTCCTGATCTTCAATTTGAAGAAAGGAATTTCCAATCC

CAATTCACTGTTTCATCTGATAAACTCTATGAATGGAATATAGATGGTTTATCAGAACAA

GAGTTATTAAACAAACTCCAACACATCTCCATGGTAGCAAATAGCTATATCACTAATCAT

GACCTTAGTCAAACCCAAATTGTTGACATCCTTGTCTCTGGATTCACAGGAATGCTTCAT

TCCTGGTGGGAAAAACATCTCACTGAAGACTCCAGAAATCTAATAAGAAATGCAGTAAAA

CAAGATGAAGAAGGAATACCTATCTTCGATGAACATCTAGGACGTGGTGTCCCAGATGCA

GTCAATACCTTACTTTATACAATCATACAACATTTCATAGGAATCCCTTCCAACATCACA

TCTAGAATCCATGACCAACTAAGTAACTTAAGGTGTCCAACCCTAAGTGATTTTAGATGG

TATAAAGATGTATTTATGTCAAGAGTTATGCTTAGAGATGATAGTAATCAACCATTTTGG

AAAGAAAAATTTATAAATGGATTACCAAACCTTTTTGCACACAAAATAAGGAATACCTTA

ATTAACGAAGAAGGTATTATACCATATAATGATCTTACTTATGGACATATCATAAGTACT

ATCCAAAAAGAAGGCCTAAAAATGTGCATAGATATGAAAATATCTAGACAAGCACAATCT

GAAAAGGCTATCGCCAAATATGAATTAGGAACCTTCTGTGAGCAATATGGTTTACCACCT

ATAGCTCCTTCCAATCTCAAAAAGAAATCAACTACCTTCAATAGATCTAAGATCTCTAAA

CCACGGACCAACTACTCCAAATATCATAAAGGCAAGTATGCCAAACCAAACCCATTTTAT

GAAAACCCCAAACAGAAAAGATGGAAAGGAAAGAGAAAATCCTCTGGAAAATTCCATTCT

AAAGACAATAGTCATAAGGGAAAATGCTACAAATGTGGCAAAGAAGGCCATTATGCCAAT

AAATGTAAAGTTAAGAAAACCATAAGTCAGTTGAAGATTTCTGAGGAAGAAAAGCTTCAA

CTTATCCAAGCCCTAGAACTCCAAAATACCGACAGTAGTCATTCATCTGAAGATGATCTA

GGTTCCACCTCATCATCCTCATACCAATCTGCCTCTAGTAAGCACTCTACCCCAAACATC

AAGCTCGGATGTCCTGATGCTTGTTGTAAAAATATATCAGTACTAACTAAACAAGAAAAA

CAAGAAGAATTACTCTTAGAGCTAATAAGCAATGTTGAGGACACTGAACTCAAAAGGTTC

TACCTCAAAAAGCTTAAAAACTTAGTCTCCCAAGAAGAAGTAGGCACTAGCCAACCTCAA

ATCCAAAAACCCCAAATCTCTCTTAGTTCTACACTAGCAAAGTTTAGTAAGACTAAGAAA

GAAATTACGGTCAGTGACCTCCAAAAAGAAATTAATCAGATAAAAGAAGAAATTAAACTT

CTAAAATCTGATAATCACGAGATTCGTACACAACTCACTAAAGTCAATACTCCTCCAAAA

GAAGAACTATTCAGTTCCTCTTCTTCCAGTAGTCACCATGAGTCAGAATCTGATACACAT

TCAGAACACTTAGTTCTGAATCTTCTTCACAAAATTAGAATCCAAAAATGGTATTCTAAA

GTAACAATAGTAATTGAAGACTTTCGTTTCGAAACAATTGCATTAATTGATTCAGGAGCA

GACTTGAATTGTATTCAAGAAGGACTAATTCCCACTAGGTATTTCCACAAATCAAAGGAA

ATCCTTAGTGCTGCTAATGAATCTCCAATGGAAATCAAGTATGAACTTCCTAAGGCGCAT

GTCTGCCAAAATGAAGTTTGTTTCAAAACACCTTTTGTTCTAATTAAGAACTTATCTGAC

CCTGTCATACTAGGACTACCCTTTATAGCACTCCTCTATCCTTTTAAAACACATCATAAT

AGGATTACCTCAAAAGTCTTAGGACAGAAAGTCACTTTTGAGTTCTGCCTGGAAACAGAC

CTCAAAAAACTTAGGCATCTCCAGAAGGATAGCACTTCGCGATCCTTAAACATAATCTCT

AATAAGACTAAACATCTTCAATTCTTGAAAGATGATATTCATCACAAAAGAACTGAAGAA

CAACTCAAACAAGAGATTATATCTCAAAAAATCCTTAAATTTGAAGAACAACTTGTTAAA

GAAGTATGTTCTGATATTCCCAATGCCTTTTGGCACCGCAAACAACATATCGTTAAATTA

CCTTATATCAAGGAATTTAATGAGTCAAAAATCCCTACTAAGGCCAGACCTATTCAAATG

AGTCAAGAAGTAATGGATTTCTGTAAGGCAGAAATACAAGACCTTCTTAATAAAGGAATT

ATCCGTAAAAGTAAATCACCTTGGTCGTGTCCTGCATTTTATGTTCAGAAAAGTGCAGAA

CTTGAAAGGGGATCTCCCCGATTAGTTATTAACTACAAACCTTTAAATGAAGTTTTGGAA

TGGATTCGATATCCAATTCCCAACAAAAGAGATTTAATAAAAAGGCTTAGTAAAGCAACT

ATTTTCTCAAAGTTTGATATGAAATCAGGTTTTTGGCAAATCCAAATTCATGAATCTGAT

AAATATAAAACTGCTTTTGTAACGCCTTTCGGCCATTATGAATGGAATGTTATGCCTTTT

GGTTTGAAAAATGCACCATCTGAATTCCAGAATATTATGAATGAGATTTTTAATCAGTAT

AGTCATTTTACAATTGTTTATATTGATGATGTTCTTGTTTTCTCTAAATCTATAGATGAA

CATTGGAAACATTTACACTTGTTTGCTAGGATCATTAGATCAAATGGATTGGTTGTGTCA

GCCACTAAGATTAAGTTGTTTCAACTTAAGGTTAGATTTTTAGGTTATAATATCCATAGA

TCTACCATCCAACCAATTGACCGTGTCATCCAATTTGCAGATAAATTTCCAGACCAGATT

ACTGAAAAAACCCAGCTTCAAAGATTCTTAGGTTCTTTGAATTATGTTTCAGAATTTTAT

CCTCATTTGCGTCAACAATGTAAACCCTTGTTTGATCGCCTTAAGGAGAATCCTCCTGCC

TGGTCTACCATTCATACTTCTATTGTCAAACAGATCAAAAATCATGTTAAGACTTTGCCC

TGTCTTGGCATTCCAACTCCCAACTCTTTTAAGATTGTTGAAACTGACGCCTCTGACGTT

GGTTATGGAGGTATCCTCAAACAGCAAACTTCCTCCGGATCACCTGAACAAATTGTTCGT

TTCCATTCAGGTGTATGGAATCCTGCTCAAACTAATTATAGTACTATTAAAAAAGAAATA

CTATCTATAGTTTTATGCATTAGTAAATTTCAAGATGATTTATTAAATCAAAAATTTCTT

GTTAGAGTCGATTGCAAATCTGCAAAACATGTTTTACAAAAAGATGTTCAAAACATTGCA

TCAAAACAGATTTTTGCACGATGGCAAGCCATATTATCAATCTTTGATTTTGAAATTGAA

TACATTAAAGGCAGCCATAACTGCATTCCAGATTTCCTAACCAGAGAATTTTTGCAGGGG

AAGAATGTCTAATAAAAACACAGCTTCTCCACAAGGCAGTAGCCAACAAAGGAGGCTTCC

CGCCTCATTACCACCACCAAAACAGGAACCTATTCCCGGTTCCTCGGCCTTAGCCATTAC

CAATAGATTTACTCCATTAGGATCAACTCTAGGAAGTATCCGACCAAATTACCAAACAGC

GTTAGTAAACCAGTATGATCCTTTCTCCTCTACCTACAGGCAATCGCCATCTCCCTCAGT

AAGTTATACCAAAACATCTCCTTATATTATTAAACCCTCCAAAGAGTATCTCTTCAGTAT

CCCCTTCAACTACAAACATCTGAAAACACCTGAGGTAATAGCCAAAAAGATGTTTCCAAC

CAATTCCCATTTTCAGCCAAGTGAATTTCACAAAACCCTCAAGTACTATACTACTATTCT

CAATGAGACTTCATCCATCACCATAAAACCTATCTTTGATAAAACCTCTGGCAATCAAAA

CAGAATCCTGTATCATTCCCTTACCATTAATAAATTCTTATTAGAATCAGAATGGCAAGT

ACCTCTTTTTCAGCCTAAACCTTTTAAAAGCAGTAGCTTTCCCAGTCACTCAAACCATTA

TACCTATTTTGATTACATTCAAGCTTGGACTAACATTTTCCTACACCAATCCGAAGATTT

TAGTCATTCATGGTTTATAACCTTTGATCGAAGTTTCAGATTGAATTTTCCTGCATGGTT

TCCCAGATGGTGGTCAGAACATGGCCTTATCTCTGACCTCATGCCGGACGAATTCAAGCA

AGTCTTATCAGGCACTTTCCAGAAGAAGTTCGATAGAACACGTTTTGATGAAGGAATCCC

TTTGTTCGCCCTGTTCATGGCAAAATACAAGGTCCCCTGGATCCTCCGCTGGAACTACGA

GATTGAATCCAATCAAGTCTTTCGACAGAGAATGATCAAATGGTGGGACAAGTTCAACTA

CCAGAAGATCATTGAAGTTGTGCTCAAGGACTTCCCCATGGGTTCTAATCCAGTACCTGC

AGTAGCAGCTCCTCCAGCAACAGCTTTACCAGAACCAACCAAGGAAGAAATCGATTCATT

ATCGGACATTAGTCCTTCTTCCTCTCTCAAGGGCAAGACTAAGTCATCCAAATCTTCGAG

CAGTTCGAAGAAAAAGAAATCATCAAAGCTCAAGGCTCTAGCCAAAATCCTCATGAAGGA

ACTGCAAGAAGAAAATGAAGACAGTTCGGACTCCGACAACTCTTCGGATGCTTCATCACA

ATGCGCAGTAGCCACACAAGGAAATATTTTCCCGCCTGATTCTCAAGACCCATACGAATT

TTAAACTTGTAATAGCATGTATAGGAATTTCTAAATTCGTATGAATGTAATCTCAGTAGC

ATACATTGTATTTAGACTCAGTAGAGTATTATGTAATAACTGCATATGAAGACAAAGCCA

GCCGCAAGTGAAAACAGCAGTAGCAGACAAGGCACTATTCATGAATAGTAAAATCACTTT

TACTATTCATCCATCATTTCCTCCACAGTATTCACCCTCGTGAAAGCCACAGTCACCGAC

AGCATCTTCTGCTTTCGGCCTCTTTACAGCAGTAGCACATGCAAAGCGGGAATCCCGCTT

CCATCCTTCAATCATTCCGGATCACCTCCCTCGTCTATATAAGGACTCTCGAGCTATCTG

GAGAAGGGACTTCGAATTTCGGAAGAAAAAAACTCTGCGTATTCATATGGAACAGAGTCT

ACAGCTTCTCAGCAAAAATATTGTAATAATTATATTTTAATTTTAAAGGATGCCTGCGAG

CTCCAAGATCAGTCTTTAAGTTTTCAAAATCGTCAATAGACGAAACTTCTGTAAGTCTTA

TTTGATTTTCAATAAATATATTTTCAATCGAATATCAGCATCATTAATTTAATCATGAAT

ACTAAATCTATTTTAAATTTAGTTTTGTTTATATATGCTCTTGTTATTAATCCTAAAAAT

TTTATATTGATATTAATCAAGTTCTTTGAATTCCCACATACTTATATCGGATATTTCCGG

CTTCATATGTTACCCTTCTTCCGCACCCATT

>MdomV_scMd2 [endogenous-virus-name=Malus domestica virus] [strain=sequence cluster 2] [host=Malus x domestica cv. Golden Delicious] [moltype=genomic DNA] [note=incomplete at 5’ and 3’ ends] 6541 bp

TTGAAAAAGCAACTGCTCTTAGCAAAGAACATGAAACTTGTCAATTATTCACACCTGAAC

AAATCAGAACTCATAGAAAAGATGGTCATAATTATATCCACATAGGTTTAGTCCAAATAG

CAGTCAAGCCTTTAACTAGAAGAGGTATTAATACCTCTATTTTGTTATATTTACGAGATG

TGCGATTCACTGACTTTAGTGATAGCATTCTTGGAATGATCGAATCAAGTCTTTACAACG

GACCAATTCATTTTGATTGTTTCCCAAACCTCACAATAAGTCTTTCAGATCCCCACATGC

TGAAGGCACTCACTTTAAACATCAAAACTTTAGGATACCAAGTTCTTGAAGGAGTTCAAC

CATTAGCCTTAATCTTTAGAGTTTACTACAAAGTCATTGGAACAAACATGAATTTTCAAG

CCTTAGTCAAGAGTCCAAGAGACCACACACTTCTAATCCAAACCAAGCAAGAAAATGCTA

ACATCAAAATACCTAGAACCATTAGGTAGTTAGATATCAATCTACCTTCAGATTGGTGTT

TGATTAATGAAAGCAAACCAGTTAATATTCAAAATAGTCTAGTCAACCAAGACAACATAG

AACAATATTTTGATGAAAAAGTTAAGATCAACTTTGACCGACCAGCTAGAAAATCTAATG

TCAACCCCCATGAGTTATCCCAGTCACATAGGTCTTTAGCCCCCTGTAGGAATTCTTTTG

CCATATTCACATCTACCAGCATGGTAGATCAAGATCAAGATCTCATTAATTCTTTAATCA

ACAAAAAATTAAAAGCAGTAGAAAATAAGTTCAACTGTCACCCAACAAGAATTAATCAAT

GATTTGATTAATATCAAACTAAAATCAATAAAAACAACCTCTCAAGTCACTCATCCCGTT

TATCAACCCAATACCCAAAATCAAGGAGAACGAACTTCTCCAACAGCATCTGACTATGAC

ACAGGTCAAGTTAATCATCAATTACTTATGTTAAATAAGCCATTTAAAATTCGATGGAAG

AAGCTCAATGTTGACATCGAAGCAACAGAGAACATTCCTCGATGAAATATTTTCATAAAC

AAATATACAAAAGAACAAAAATTGGACATTTACAATAAATGGACCAATTTCATGAACAAA

TACAGATTCAAAGTATTTTTCTTCGACTTTGTTGAAAGATATTATGAATCTAAAAACAAA

TTAAAGGTTATCACCAAAGAAAATTGGGTCAAACAAGATAAAACCATAGTTTCATCTAGT

CATCCCCCACAAGAAACTATTTTAATTTCCACTGCTAATACTCAAATACTAGCCACTCCT

TACAAACTCCCAAAAGAGGATGCAGGAGTCAAACCTGTCATTGAACAAATCAATTTCACA

AATCAGAGTCTTCATACCATGGGAAAACAACTAGATAAAATTGAAATAAAAATAGACAAA

CTTTCCCAACCAAAACCAACATAGGGAAAAACCTCTAGTCAACTTTCCAAAGTCTTCTTC

CAACATAATCGCTTTGAAAACCATCTCCACAAGCCAAAAAATTGACCAAATGTTACAAGA

ATTAAAACAAGAAAAGACAATCAATGTTTTACAACACCCGGAAGAGTCTCATGAAGAAAC

AATTTCTACAATTTGGGATTCAGAAAATGAAACTGAAATAGATTCCATTAGGAAACTCGA

AATTTTTTTCCAAAACCTTGAACTCAAAAGAATCACAAATAAATGAGTCAACCCAACACC

TCTTACAAAAAATTGGTATCCAAGACCAACGCCTCCGGATATTCAGTTTGAAGAACGAAA

CATCCAAAATCAATTCTCAGTCTCAGCTAATAAACTCTATGAATGGAATATAGATGGTTT

ATCAGAACAAGAACTTCTAAACAAATTACAACACATCTATATGGTTGCTAATAGTTATAT

TACCAATAATAATCTTAGCCAAGTACAAGTTGTTAACCTTTTGGTCATTGGATTTACAGG

AATGCTTCATTCCTGGTGAGAAAAACATCTCACTGAACAATCAAGAAATGAAATAAAAAA

TGCAGTTAAAAAGGATGAGGAAGGAATTCCCATCTTTGATGAGCATATAGGACAAAAAGT

CTCAGATGCAGTGAACACATTACTTTTCACCATTATAGAACATTTCATAGGGACATCTAG

TAATGTCACCTCCAGAATTCATGACCAACTTAGCAATTTACGATGTTCAACCTTAAGTGA

TTTTAGGTGGTATAAAGATGTCTCCATGTCTAGAGTCATGCTTAGAGAAGATAGTAATCA

ACCATTTTGGAAAGAAAAATTTATTAATGGATTACCAAACCTTTTTGCCCACAAAATTAG

AAATGTTTTAGTCAATGAAGAAGGTATCATACCATATCATACCCTTACTTATGGAAATAT

TATTAATATTATCCGAAGGAAGGATTGAAAATGTGTATAGATATGAAAATCTCAAAACAA

GTTAACAAAGATAAATCAATAGCTAAATACAGATTAGGAATATTCTATGAACAATATGGA

TTACCTCTTATTGCTCCATCCAACAAAAAGAAAAAAATCACAAGTCTACCATAAGTACCC

CAATAGGTACTCAAGTAGACAAAACCTCAGATACAACAAATACAAATCCAAAAACAAAAA

CTTTGAAACCAATAAATTTTATGAAAAACCCAAGTCTAATAAATGGAAAAGAAAACCCTC

AGAAAAATTTCACTCCAAAAAGCAAGAACAAAAAAGGAAAATGCTACAAGTGTGGCAAAT

TTGGTCATTATGCAAAAAAATGTTGTGTCAAGAAAACAATCAACCAATTGAAGATCTTTG

AGGAAGAGAAGATTCAATTAATCCAAGTCCTAGAATTAAAGGACACAGACCATAGTCAAT

CCGAGGATGATCTAAGTTCTAGTTCATCTTCCTCCAATAATTCTTCAAGTAGTCTTTCAT

CCCCAAATATTAAGATAGGATGCACTGATACTTGTTGTAACAAGATTTCAGTCCTCAATA

AACAAGAAGAACATGAAGATCTTCTTAAAGAGTTAATCAGTAAGGTCGATGACCCGGAAT

TAAAATCATATTATTTAAAGAAACTCAAAAAACTAGTCTCTCACGAAGAAACTAGTACTA

GTCAACCCTAGTCATCACAAAAAATCAGTCTAAGTACAACTTTAGAACGATTCAACAAAT

TCAAGAGGGAAATCACAGTGAAATACTTACAGAGAGAAGTCAATAAAATCAAAGATGAAA

TTAGGGTTCTCAAATCAGAAAATCTAGAAATCCGTACACATCTCAGTAGAGTTCAAATGC

AAATGATTGGCAACAACAATGATGAAGAACACATTAGTTCTTCTTCAAGTCACAATTCAG

ACTCCCAGTTTGAAGAAACGTCATCAAAAAATTTAATTTTAGGATTATTAGACAAGATAA

GAATCCAGAAATGGTATTCTAAAATCAAAATAATAATTGAAGATTTTCACTTAGAAACAA

TTGCTTTGATTGACTCAGGAGCAGACTTGAATTGTATTCAAGAAGGACTAATTCCTACTA

AGTATTTTCATAAATCAACAAAAATACTTAGTGCTGCAAACCAGTCACCAATGCAATTAA

AATATGAAATTCCAAAAGCCCATGTTTGTCGAAACAATGTTTGTTTAAAAACCCCATTCG

TTTTAATAAAAAATATCTCGGATCTAGTCATTTTAGGGCTTCCTTTCATAGCCCTTTTAT

ATCCTTTTAAGGTTCATCATAATCGTATAACTACCCGAATGTTAGGACAGAAAATTATCT

TTGAGTTCTGCACGGAAACAGACCTAAAAAAAGTAAGACAATTACAAAAGAATAGCAACT

AAAAAACCATCAATTTAGTCAGTAAGAAATCAACAAATTAATTTCTTAAAAGAAGAAATT

AATTACAAAAGAATTGACGAACAATTAACGAATCAATCTTTGTTACAAAACATTGACAAT

TTTCAAAATAAACTTGTTAATGAAATATGTTCTGAATATTCCCAATGCCTTTTGGCATCG

AAAACAACATATTGTCAAACTCCCTTACAATAAGGATTTTGATGAAAAGAAAATACCAAT

CAAAGCCAGACTAATTCAAATGAGTCAAGAAGTTATGGAATTCTACAAGGCAGAAATTCA

AGAACTTCTTAGTAAAGGAATAATTAGGAAGAGTAAATCACTATGGTCATGCCCTGCTTT

CTATGTTCAGAAGAATGCTAAATTAGAAAGAGGAACTCCCAGGTTAGTCATCAATTATAA

ACCTCTTAATGAAGTCTTGGAATGGATACGATATCCAATTCCTAACAAAAGAGATTTAAT

TAAGAGACTAAGTCAAGCTGTGATATTTTCAAAATTTGACATGATGTCTAGATTTTAGCA

AATCCAAATACATAAGGATGACAAATACAAAACAACGTTTGTCACTCCTTTTGGTCATTA

TGAATGGAATGTAATGCCCTTCGGCCTTAAGAATGCACCCAATGAATTTCAGAATATCAT

GAATAAAATATTTAATCAATACAATCATTTCTCAATTGTTTACATTGATGATGTTCTTAT

TTATTCAAAATCAATAGATGAGCATTGGAAACATTTGAATATGTTTGCCAGGACAATTAA

GTCAAATGGATTAGTTGTCTCAACAACAAAGATTAAATTATTTCAAACCAAAATTAGGTT

TTTAGGATACAATATCCACCAGTCTACCATTCAACCAATAGACAGAGTCATCCAATTTGC

AGATAAGTTTCCTGATGAAATCACTGATAAAACCCAATTACAGAGATTTCTAGGATCTTT

AAATATGTTGCAGAATTTTATCCCCATCTCAGACAACAATGCAAACCTTTATTTGATTGT

CTCAAAGAAAATCCTTCACCTTGGTCTCATACTCATACTTCTATTGTCAAACAAATCAAA

TCCCATGTTAAGACTTTACCCTKTCTTGGCATTCCAACTTCCAATTCATTCAAAATTGTC

GAAATTGATGCCTCTGAAATTGGTCACGGAAGTATCCTCAAACAGTCAATCTCATCCAGA

TCTCCTGAACAAATTGTTCGTTTTCATTCAGGAGTATGGAATCCAACACAAAGTAATTAT

AGTACTAGAGATATTATCTATTGTATTATACATTAGCAAATTTCAAGATGATTTATTAAA

TAAATTTTTTTTAGTCAAAGTCGATTGCAAATCTGCAAAATATGTTTTACAAAAAGATAT

TCAAAACATTGCATCAAAACAGATTTTTGCACGATGGCAAGCCATATTAAGTATTTTTGA

TTTTGACATTGAATATATTAAATGTAGCCAAAATTGCATTCCTAATTTCTTGACCAGAGA

ATTTTTGCAGGGGAAGAATGACGGACAAAAGAAATAATCAAAGAAGGCTTCCAACCACCC

TAACATCACCACCAGTAAAACAAGAATCAAGCCCAGATTCTTCATTAGCCTTAACCATTA

CCAACTGATTCACTCCCTTAGGTTCCACAATAGGAAACTTCAGACCAAACTACCAAACAC

CACAACCAAATTACCAAACGACATTAGTCAGCCAATATGATTCTTACTCATCTTAGCCAT

ACCATCCATCATCGTCTCAATCAGCCAACTATGCTAAAACATCCCCTTATGTCCAGAAAA

ACCCGAAGGAATACCTTTTCAGCATCCCCTTCAATATTCACCAAGATCAAGCCCCAGAAA

AAATAGCCAAAACAGTTTTTCCTCCCAATTTTCATTATCAGCTTATTGAATCTATTAAGA

CTCAAATATTATCAAGCCATTCTCAGTGAGACTGAATCCATCTCAATTAAACCAATTTAT

AGCACAACCCATGAAAACAAAATCTTTTACCATTCTCTTTATATTGGAAAGTTTTTAACT

AAATTAGAATGGGGACTCCCCCTCTACCAGACAAAACCTCTTCATACCCAGCATGTTCTC

ATCCCCAATAATCATTACAATTATTTTGATTATATTCAAGCATGGACTAACATTTTCCTC

CATCAAACAGAAGACTTTAGTCACTCATGGTTTATCAATTTTGACAAGAGTTTCAGATTA

AATTTCCCTGCATGGTTTCCAAAATAGTGGTCAGTTCATGGCCCAATAGCCAACCTCTTA

CCAGAAGAACTCATGCAAGTCCTCTCAGCTGGTTTCCAGCAAAAATTTGACAGAAGCCGA

TTCAACTACAAGATTACACTTCTCCCATTGTTTATGGCTAAGTACAAAGTCCCGTGGATA

ATGAAGTGGAATTATGAAGTCGAAACCGGCGAAGTCTACCGAACAAGATCAGTCAAGTGA

TGGGATAAGTTCAATCATTAGAAGATCATCAATCTGGTCCTCTCAAAATTTCCCGTCACT

CCAGTTCTAGTCATCCCAGTAGCCTAAGCTCATGCTATTTCAGCACAACCAAAAATGGCA

TTGCCAATCATTTCAGAATAATCTCTATCAGACATTAGTCCTTCATCATCCCTCAAAGGA

ACAACCAAGTCATCTAAATCCACAAGTTCGAAGAAGAAAGAAAAATCATCCCAGCTCAAG

G

>MdomV_scMd3 [endogenous-virus-name=Malus domestica virus] [strain=sequence cluster 3] [host=Malus x domestica cv. Golden Delicious] [moltype=genomic DNA] [note=incomplete at 5’ and 3’ ends] 6669 bp

TTTCTGTGTTTTGTTAGTCTGAATTTTGTTCAAAATGAGTAGATTATTTAGATCTAATTC

AATGGCCAGCACTAGCTCTATATCCAGTCTAGGAACCATTCCAAATGTAGTTAATGAAGA

ACAAAAATTAGAATTTCAAACAGACGACAGTGTCGACTTTGGAGATTGGAATATCCCTAA

GATTTCTAGTAAAAACATTTATAAAAAGAAACGGTCAGTAGCCTCCTTTAGAAGTGAACA

CCATATAAAAACAATTGAAAAAGCATATGCTCTTAACAAAGAACATGAAACTTGTCAATT

ATTCACGCCAGAACAAATCAAAACCCATAGGAAAGATGGTCATAATTATATCCACATAGG

ATTAGTTCAAATAGTAGTCAAGCCTTTAACAAGAAGAGGTCTTAATACCTCTATTTTGTT

ATGTCTCCGAGATGCGAGATTCATTGACTTTAGTGATAGCATTCTTGGAATGATTGACTC

AAGTCTATTTAACAGACCAATTCATTTTGATTGTTTCCCAGATCTCACAATAAGTCTTTC

AGATCCCCACATGCTAAAAGCACTCACATTAAACATCAAAATTTCAGGATACCAAGTCCT

TGAAGGAGTTCAACCATTCGCCTTAATTTTTAGGCTTTACTACAAAATCACTGGAACAAA

CATGAATTTTCAAGCCTTAGTCAGGAGTCCTAGAGACCACACACTTCTAATCCAAACCAA

CCAAGAAAATGCCAACATCAAAATACCTAGAACCATTAGGTGGTCAGATATGAGTCTACC

TTCTGACTAGTGCTTGATCCATGAAAGCAAACTAGTTAGCATCCAAAATAGTTTAGTTAA

CTTAGACAACATAGAACAATATTTTGATGGAACAGTTAAAATCAACTTTGACCAATTGGC

TAGAAAATCTAGTGTTAACCTCCATGAGTTATCTCAGTCACATAGGTCTTTACCTCCCAG

TATGAATTCTTTTGTCGGATCCACATCTGCCAGCATGGTAGATCAAGATCAAGATCTCAT

TAATTCTTTAATCAACAAAAAATTAAAAGCCATAGAAACAAGTTCTCAAGTCACTCATCC

TGTCTATCAATCCAATACCCAAAATCAAGAAGAACAAACTTCTTCAACAGCATCTGACTT

CAAAGCAGGTCAAATTAATCATCAATTACTAGTATTAAATAAACCATTTAAGATTTGATA

GAAGAAACTCAATGCTTACATCGAAGCTATAGAAAACATTCCTCGTAGGGACATTTTTCA

TAATAGGTATACAAAAGATCAAAAAATGGACATTTATAATAAATGGACCAATTTCATGAA

AAAACACAGGTTTGAAGTATTTTTCTTCGACTTTGTTGAAAAGTATTACGAATCTGAAAA

CAAGTTAAAGGTAATCACCAAAGAAAATTGGGTCAAAGAAGATAAGACATTAGTTTCCTC

TAGTCATCCCCAAAAAGAAACATTTTAATTTCCACTGCAAACACACAAGTACCAACCACT

CCTTTTAAACTTCCAAAAGAAGATGCAAGAGTTAAGCCAGTCATTGAACAAAACAATTTC

ACGAATCAGAGTCTTCATACTATTGGAAAATGGTTACATAAAATTGAAACAAAGATAGAT

AAACTATCCCAACCAAAACCAACACGTAGGGAAAAACCTTTAGTCAACTTTCTAGAGTCT

TCTTCTAGCACAATCGCTTTGAAAACAATTTCCACAAGCCAAAAAATAGACCAAATGTTA

CAAGAATTAAAACAAGAAAAAACAGTTAATGTTTTACAAAATCAAGAAGAGTCTCATGTA

GATACAATCTTTACAATTTGGGATTCGGAAAATGAAACCGAAACAGATTCCATTAGGAAA

GTAGAACAAGCTTTTCAAAACCTTGAACGCAAAAGAATCACAAATAAACGAGTCAACCCA

AAATCTCTTACCAAAAATTGGTACCCGAGACCAACACCTCCGGATATTCAGTTTGAGGAA

CGAAACATCCAAAATCAATTCTCTGTCTCATATGATAAACTCTATGAATGGAATATAGAT

GATTTATCAAAACAAGAACTTTTGAACTAATTACAACAAATTTCTATGGTTGCTAATAGT

TATATTATCAATCACAATCTTAGTCAAACACAAGTTGTCGACCTTTTGGTCACTGGATTC

ACAGGAATGCTTCATTCCTGGTGGGAAAAAACATCTCACTGAACAATCAAGAAATGAAAT

AAAAAAATGCAGTTAAAAAAGATGAAGACGAAATCCCTATATTTAATGAGCATATAGGAC

AAAGAGTCCCAGATGCAGTCAACACCTTACTCTTTACCATCATCGAACATTTCATAGGAA

CACCCAGTAATGTCACCTCCAGAATTCATGACCAACTTAGCAATTTAAGATGTCCAACCT

TAAAAGATTTTTGGTGGTATAAAGATGCTTTATGTCCAGAGTCATGCTTAGAGAAGATAG

TAATCAATCATTTTGGAAAGAAAAATTTATTAATGGATTACCAAACATTTTTGCCCATAA

AATTAGAAATGTTTTAGTCAATGAAGAACCCTTACTTATGGAAAATATTATTAATATTAT

TCAGAAGGAATGATTGAAAATGTGTATTGATATGAAAATTTCAAAACAAGTCAATAAAGA

CAAATCCATAGCCAAATACGAATTAGGGACATTCTATGAAGAATATGGATTAGATCCCAT

TGCTCCATCCAACAAAAAGAAAAAATCACAAGCCTACAATAAGTACCCCAATAGGTACTT

AAGTAGACAATGCCCCAGATACAACAAATACAAATCCAAAAACAAAAACTTTGAAACCAA

TAAGTTTTATGAAAAACCCAGGTCTAATAAATGGAAAAGAAAACCCTCAGAAAAATTTCA

CTCCAAGAAACAAGAACAAAAAGGAAAATGCTACAAGTGTGGCAAATTTGGTCATTATGC

AAATAAATGTCGAGTCAAGAAAACAATCAACCAGCTGAAAATCTCTGAAGAAGAGAAACT

ACAGCTAATCCAAGTCTTAGAATTAAGAGATACAAACCATAATCAATCCGAAGATGATTT

AAGTTCTAATTCATCTTCTTCCAACAATTCCTCAAGTGGTCTTTCATCCCCCAACGTTAA

ATTAGGATACACTGACACTTATTGTAAAAAGATCTTAGTCCTTAATAAACAAAAAGAACA

AGAAGATCTTCTTATTGAATTAATTAGCAATGTTGATGACCCTAAACTAAAATCATTTTA

TTTGAAGAAGCTCAAAAAACTGGTTTCTTACAAAGAAACTAGTACTAGTCAACCTTAGTC

ATCTCAAAAAATCAGTCTAAGCACAACTTTAGAACGATTCAACAAATCCAAGAAAGAAAT

CACAGTGACAGACTTACAGAGAGAAGTCAATAAAATCAAAGATGAAATTAGGTTTCTCAA

ATCAGAAAACTTGGACATTCGTACACATCTTAGTAGAGTTCAAACACAAATAATTGTCAA

CAATAATAATGAAGAACATAGTAGTTCTTCTTCAAGTCACAATTCAGACTCCCAGTATGA

AGAAACATCGTCAAAAACTCTAATTTTAGGATTATTAGACAAGATAAGAATCCAGAAATG

ATATTCTAAAATTAAAATAATAATTGATGACTTTCATTTCGAAACAATTGCTTTGATTGA

TTCAGGAGCAAACTTGAATTGTATTCAAGAAGGATTAACAAAAATACTTAGTGCTACGAA

CCACTCACCAATGCAATTAAAATATGAAATTCCAAAAGCCCATGTTTGTCAAAACAATGT

CTGTTTTAAAACCCCATTTGTTTTAATCAAAAATATATCAGACCCGGTCATTTTAGGTCT

TCCTTTCATAGCCCTACTATATCCTTTAAAGGTACACCATAATCATACAACTACCAGAAT

GTTAGGACAGAAAGTCGTCTTTGAGTTCTGCATGGAAACAGATCTCAGAAAATTAAGACA

GTTACAAAAGAATAGTATCTCAAAAACCATCAACTTAGTTAATAAGAAATCTCAACAAAT

TAATTACAAAAGAATTGAAGAACAATTAACAAATAAATCTTTGTTACAAAGCATTGACAA

TTTTCAAAAGAAACTTGTAAATGGAATTTGTTCTGATATTCCCAATGTCTTTTGGCACAG

AAAACAACACATTGTCGAACTTCCTTACAATAAGGATTTTGACGAAAAGAAAATACCAAC

CAAAGCCAGACCAATTTAAATGAGTCAAGAAGTTATGGAATTTTGCAAGGGAGAAATCCA

AGAACACTTAATAAAGGAATAATTAGGAAGAGTAAATCACCATGGTCATGCCCTGCTTTC

TATGTTTAATAAAATGCTGAATTAGAAAGAGAAACTCCCATGTTAGTCATGAATTATAAA

CCTCTTAATGAAGTCTTGGAATGGATACGATATCTGATCCCTAACAAAAGAGATTTAATT

AAGAGACTTAGTCAAGCTGTGATATTTTCAAAATTTGACATGAAGTCTGGATTTTGGCAA

ATCCAAATACATGAGGATGACAAATACAAAACAACGTTTGTCACTCTTTTTTGTCATTAT

GAATGGAATGTAATGCCTTTCGGCCTCAAGAATGCACCCAGTGAATTTCAGAAAATCATG

AATGAAATATTTAATCAATATAGTCATTTCTCAATTGTTTACATTGATGATGTTCTTGTT

TATTCAAAATCAATAGATGAGCATTGGAAACATTTGAATATGTTTGCCAGATAATTAAGT

CAAATGGATTAGTTGTCTCAGCAACAAAGATTAAATTATTTCATACCAAAATTAGGTTTT

TAGGATACAATATCCACCAATCTACCATTCAACCAATAGACAGAGTCATCCAATTTCCAG

ATAAGTTTCTAGATGAAATCATTGATAAAACCCAATTATAGAGATTTCTAGGATCCTTAA

ATTATGTTGTAGAATTTTATCCCCATCTCAGACAACAATGCAAACCTTTTTTTGATCGTC

TTAAAGAAACTCCTTCACCTTGGTCTCATACTCATACTTCTATTGTCAAAAAAATCAAAT

CCCATGTTAAGACTTTACCCTGTCTTGGTATTCCATCTCCCAATTCATTCAAAATTGTCG

AAACTGATGCCTCTGAAATTGGTTACGGAGGTATCCTCAAACAGTCAATCTCATCTGGAT

CTCCTTAATAAATTGTTTGTTTTCATTCAGGAGTATGGAATCTAGCACAAAGTAATTATA

GTACTATTAAGAAAAAGATATTATGTATCGTATTATGCATTAGCAAATTTCAAGATGATT

TATTAAATCAAAGGTTTTTTATCAGAGTCGATTGCAAATCTGCAAAACATGTTTTACAAA

AAGATGTCCAAAACATTGCATCAAAACAGTTTTTTGCACGATGGCAAGCCATATTAAGTA

TTTTTTATTTTGAAATTGAATATATTAAAGGCAGCCAAAATTGCATTCCTGATTTCTTGT

CCAGAGAATTTTTGCAAGGGAAGAATGACGGACAAAAGAAATAATCAAAGAAGGCTTCCA

GCCACCCTACCACCACCACCAGTAAAACAAGAATCAAGCCCAGATTCTTCATTAGCCTTA

GCCATTACCAACCGATTCACTCTTTTAGGTTCCACAATAGGAAACCTCAGACCAAACTAC

CAAACACCATGACCCAAATTACCAAACTACATTAGTCAACCAATATGATCCTTACTCATC

TCAGCCATACCATCCATCATTGTCTCAATCAGCCAGCTATGCTAAAATATCCCCTTATGT

CCAGAAAAACCCGAAGGAATACCTTTTCAGCATCCCCTTCAATATTTACCAAGAACAAGC

CCTAGAAAAAATAGCCAAAACAATTTTTCCTTCCAATTTTCATTATCAGCCTACTGAATC

ATATAAGACTCTTAAATACTACCAAGCCATTCTCAGTGAGACCGAATCCATCTCAATTAA

ACCAATTTACAGCACAACCCATGAAAACAAAATCTTTTACCATTCTTTTTATATCAGAAA

GTTTTTAACTGAATCAGAATGGAGACTCCCCCTCTACTAGACAAAACCCATTTATACCCA

GCATGTTCTCATCCCCAATAATCATTACAATTATTTTGATTATATTCAAGAATGGACTAA

CATTTTCCTCCATCAAACAGAAGACTTTAGTCACTCATGATTTATCAATTTTGACAAGAG

TTTCAGATTAAATTTTCCAGCATGGTTTCCAAAATGGTGGTCAGTTTATGCCCAACAACC

AGCCTCTTACCAGAAGAACTCATGCAAGTCCTCTCAGCTGGTTTCCAGCAAAAGTTTGAC

AGAAGCTGATTCAACTACAGGATTACACTTCTCCCATTGTTCAAGGCTAAGTACAAAGTC

CCGTGGATAATGAAGTGGAATTATGAAATCGAAACTGACGAAGTCTACCGAACAAGATCA

GTCAAGTGGTAGGATAAGTTTAATCATCAAAATATCATTAATCTAGTCCTTCCAGAATTT

CCCGTCATTCTAGTTCCAGTCATCCTATCAGCCTAAGCTCAAGCTATTTCAGCACAACCA

AAAATGGAATTGCCAATCATTCCAGAACAATCTTTATCAGACATAGTCCTTCATCATCCC

TTAAAGGAACAACCAAGTCATCTAAATCCACAAGTTCGAAGAAGAAAGAAAAATCATCTC

AGCTTAAGG

>MdomV_scPp1 [endogenous-virus-name=Malus domestica virus] [strain=sequence cluster Pp1] [host=Pyrus pyrifolia cv. Choujuuro] [moltype=genomic DNA] [note=incomplete at 3’ end, mutations in ORF1 and 2] 7473 bp

TGGTATCAGAGCTAAGTGGTTGGTAATTGCATTAGCATTTATTTGATCTGTTTCTGATTC

ATACATCCTTATATTCTATCTACATTTCTTAATCGTCGGTCCTCAGTTCTTATTTCTTCC

AGCCAAACAGTAAGGCGTGGTCCAAACATTAAGTCCCAGGAGAGGCATGAGCGGGGGAAG

AGTCTAGACATACGAGGAAGGTGAATCTTTTAAGTCTTGCATATAGCATATTTGTTTTTG

TATGCATCATGAGTAGATTATTTAGATCTAATTCAATGGCCAGCACTAGCTCTAGATCCA

ACTTAGGAACTATACCCGACATTGTCAATGAAGAACAAAAATTGGATTTTCAAACGGACG

AAAGTATTGATTTTTCTGAGTGGAACATTCCAAAGGTTTCAACCCAAAATATTTACAAGA

AAAAATGGTCTTTAACCTCTTTTAAATCAGAACATCACGTCAAAACAGTTGAGAAGGCTT

ATGCTCTTAGCAAAAAACATGAGACTTGTCAATTATTTTCTCCGGAACAAATCAAAGCGC

ATCGCAAAAATGGTCATAATTATCTTCATATTGGTTTAGTCCAAATCGCAGTTAAACCAT

TAACTCGAAAAGGTCTTAACACTTCTATCCTTTTATGTTTACGCGATGCTAGATTTACTG

ATTTCAACGATAGTATACTAGGAATGATCGAATCTAGCCTTTACAACGGACCTATCCATT

TTGATTGTTTTCCCGATCTCACAATAAGTCTTTCAGATCCACATGTTTTAAAAGCTCTTA

CTCTCAATATCAAAACCTCTGGGTACCAAGTCCTGGAAGGAACACAGCCTTTGGCACTTA

TTTTTAGAATTTATTACAAAGTCACTGGCACAAATATGAATTTTCAAGCTTTGAACAAAA

GTCCTCGCGACCACACTCTTCTGATCCAAACAACCCAAGAAAATGCAAACATAAGGGTAC

CAAGAACCATTAGGTGGTCAAACATCAGTCTCCCTTCAGATTGGTGTTTAATCAACGAAA

GTAAAATGGTTTCCGTTCAAAACAGCCTTGTTAATCTCGACAACATTGAACAATATTTTG

ATGGCACCGTAAAAATTAATTTTGACAGACCAGCCAGAAATTCTTGTGAATTAGTACAAT

CACATAAATCTTTTACTCCTAGTAGACAGTCTTTTTCTGGATCCACGTCAACCGATAGAC

TGGGTCGAGATCAAGATTTGATCAAATCATTAGTAAACAAAAAATTAAAAGAGCAGGAAG

CTACACAACAAGATTTAATCAATGATTTACTTAAATTAAAGTCTGTCGACACTTCTTCAC

AAGTTGCACACCCTGTTTATCATCCTCCAAACCAAGAAGAACCAACTTTTCCAACTGCTT

TTGATTTTGAAACAGCATCGGTGAATCACCAATTACTTGTTTTAAACAAACCACATAAAA

TCCGATGGAGGAAGCTTAAAGCTGACATCGCTTCTGAGGAAAACATCCCTAGAAGAAATA

TTTTTAGAAAAAAGTATTGTGATGATGAAAAGCTAGCCATTTATGCTAAGTGAAAAGCTT

TTGTGGAACAATACCAATTTGAAATATCTTTCTTTGATTTTATTGACAAACATTATGAAA

CCAAAAATAAAGTTACGGTTGTTACCAAAGAAAATTGGGTTAAGGAGCATAAAACTTTGA

TTTCTTCCAGTCATCCTCCCAAAGAAACTATTTTGATTTCCACTGGTAATACCAACATTC

TCGCAACTCCTTTTAAAATTCCAAAAGAAGATTCTGGTTTTAAACCTGTCATTGAACAGA

ATAATTTTACAAACCAAAGTCTTCATACTATTGGAAAACAGTTAGACAAAATAGAAACTA

AAATTGACAAATTGTCTCTTCCAAAATCTTCTCAGAAAGAAAAACCATTAGTGCATTTTA

CTGATACTTCTTCAAGCTCTAATGCTTTAAAGTCTATGACTACTTTACAAAAAATTGATC

ATATGCTTACTGAGCTTAAAAAAGAAAAAGTTGTCAATGTTTTGCAAAACAATGATGTTT

CTGTTGAAGAAGAAAACACATCTGGTCCAGTTAACCCCACTTCTCTTACAAAAAACTGGT

ATCCCAGGCCAACCCCTCCTGACCTTCAATTTGAAGAAAAAAACATTTAAAATCAATTTT

CAGTCTCTGCTGACAAACTCTATGAATGGAATATAGATGGATTGTCAGAACAAGAACTTT

TGAACAAACTCCAACACATGTCCATGGTTGCTAATAGCTATATTTCAAATCATAATTTTA

CACAAACACAAATCGTTGATCTCCTCGTCACTGGCTTCACTGGAATGCTTCATTCTTGGT

GGGAGAAGCACTTAACTTCTCAGTCCAGGGATGAAATACGCTTTGCGGTCAAAAAAGCTG

AGGAAAGACTTCCTATCTTTGATGAAACAATAGGATAGGGAATACCCGACGGAGTAAATA

CTTTACTTCATACGATTATAGAACACTTTATAGGAACACCCAGCAACGTTACTACTCGCA

TTCACGACCAACTGAGTAATTTAAGGTGTCCAACCTTAAGTGATTTCAGATGGTATAAAG

ATGTTTTTATATCCAAAGTTATGCTTCGGGAAGATAGTAATCAACCCTTTTGGAAAGAAA

AATTTATCAATGGTTTACCAAATCTTTTTGCTCACAAAATCCGAAATGTTTTAGTAGACG

AAGAAGGTGTCATACCTTATCATACCCTTACTTATGGTAACATAGTAAATGTCATACAAA

AGGAAGGATTAAAAATGTGTATTGACATGAAGATTTCAAAACAAGTTAATAAAGATAAAT

CTATTGCTAAATATGAACTTGGAACATTTTGTGAACAATATGGTTTACCTCCGATTGCTC

CTTCGAGTAAAAAGAAAAAAGCCCAACAAACCAGCAAATTTTCTAAATATCAATCCAGAT

TTTACAAAAACAAAAAATCTTCCAACAAACCTTTTCAAACAAATAAATTTTATGAAAAAC

CATCTCCAAACAAAAAACCTTTTCAAAAGTCTAAAAAGGATTTCCAAAGTAAAAAGGGGA

AATGCTACAAATGTGGCAAATTTGGTCATTATGCAAATAAATGTAGAGTTAAAAAGGTTA

TTAGCCAACTAAAGATATCTGAAGAAGAAAAGGTTCAACTTATCCAGGCTTTAGAAATTA

GAAATACCGACTCTAGTCAATTTGATAAAAATCCCGATTTTTCCTACTCTAGCAGTTCTA

GTGACGTCTCCTCGCCAAATATTAAGTTTGGATGCACGGACACTTGCTGTAATAAAATTT

CAGTGCTTAATAAGCAAGAAGAACAAGAAGAGTTTTTAATGGAACTAATTAGTAAAGTCG

ATGACCCTGATTTGAAATCTTTTTATTTGAAAAAACTCAAAAATTTGATTTCTCCTCATG

AACCTGGCACTAGTCAAACTTCTTCTCAAAAGATTTCTCTCAGCACCACTTTGGAACGGT

TTAATAAACCAAAAAAAGATTTAACCATCAAAGATTTACAAAAAGAAATTAATCAAATTA

AAGCCGAAGTTAAATTTTTAAAATCTGAGAATACAGAAATTCGTTCACTACTCAGTACGG

TACGTACGCAAGCGCTTGTAGCAGAACAAGGTAATTCTTCAAACTCCGATTCCGATGCTC

ACTCAACTAAATCTTCTTCTTCAAAAGATTTTGTTTTAAGTCTTTTAGACAAAATAAGAA

TTCGAAAATGGTATTCTAAAGTTACGATAGTAATCGAAGATTTCCATTTAGAAACCATCG

CTTTAATTGACTCAGGTGCAGATTTAAATTGCATTCAAGAAGGGTTAATACCTACTAAAT

ATTTGCACAAGTCTACAGAAACACTTAGCGCTGCAAATCAATCCCCAAGGAAATTAAACT

ATGAAATTCCCAAAGCTCACGTTTGTCAACAAAATGTTTGTTTCAAAACTCCATTTGTTT

TGATCAAAAATATCTCTGATCCAGTCATTTTGGGACTCCCTTTCATTGCATTAATTTATC

CTTTCAAAGTTCATCATAACTACATTTCTACTAAGGTTTTTGGCCAAAAAATTATTTTGA

ATTTTGCATAGAAACCAATCTTAAAAAGTTAAAACAACTTCAAAAGGATAATGTTTCAAA

AACTCTCAACCAGATTACTGCAAAATCCCAACAAATTGCTTTTTTACAATAAGAAATTCT

TCACAAAAGAATTGAAGAACAGTTAACAAACAAATCTTTATTAAATTCTATTTGCCAGTT

TTCTGATAAGCTTACTAAAGAAATTTGTTCTGATCTCCCAAATGCTTTTTGGCATAGAAA

ACAGCATATTGTTAAACTACCCTATATTAAAGATTTTATTGAGTCAAAAATCCCCACAAA

AGCACGACCTATTCAAATGAATCGACAAGTTTTAGAGTTTTGTAAAACCGAAATTAATCA

ACTTTTAGACAAAGGAATTATCCGTAAAAGCAAATCTCCATGGTCTTGCCCTGCTTTTTA

CATCCAAAAAAATGCAGAATTAGAACGCGGTGTTCCACGATTAGTTATTAACTACAAACC

TTTGAATGATGTTCTAGAATGGATCCGATATCCAATCCCCAACAAAAAAGATTTGATAAA

AAGACTTTCTCAAGCAACTGTTTTTTCCAAATTTGATATGAAATCAGTCTTTTGGCAAAT

TCAAATTCATGAATCTGATAAGTACAAAACGGCTTTTGTGACTCCTTTCAGACACTATGA

GTGGAATGTAATGCCTTTCGGCTTGAAAAACGCACCAAGTGAATTTCAAAATATCATGAA

TGAGATTTTTAATCAATACAGCCATTTTTCGATTGTTTATATTGATGATGTATTAATTTT

CTCAAAATCAATAAATGAGCATTGGAAACATTTGCATGCATTTGCTAGGATCATTAGGTC

TAATGGGTTAGTCGTCTCGGCATCTAAGATTAAGTTATTTCAAACCAAAGTTAGATTTTT

AGGATACCATATTTACAGGTCTACCATTCAACCAATTGATCGAGTCATCCAGTTTGCTGA

TAAGTTTCCAGACCAAATTATTGATAAAACCCAGCTTCAGCGATTCCTCGGATCTCTCAA

TTATGTTTCAGAATTTTATCCTCATCTTCGTCAACAATGTAAACCTTTGTTTGATCAACT

CAAGGAGAATCCTCCATCATGGTCCCAAAATCATACTTCCATTGTCCAACAGATAAAAAA

TCATGTCAAGACTTTGCCTTGCCTTGGCATTCCAACTCCCGACTCTCTCAAAATAGTCGA

AACCGACGCTTCCGACATCAGTTATGGAGGTATCCTCAAACAGAAAACTTCTTCCGAATC

TCCTGAAAAAATTGTTCGTTTCCATTCAGGAATTTGGAATCCGGCTCAAAGTAACTATAG

TTCTATTAAAAAAGAGATTTTATCAATTGTATTATGCATTAGCAAATTCCAAGATGATTT

ATTAAATCAAAAATTTTTAGTTCGTGTTGATTGCAAATCTGCAAAACATGTTTTACAAAA

AGATGTTCAAAACATTGCATCAAAACAAATTTTCGTACGCCAGCAGGCCATATTAAGTAT

TTTTGATTTTGAAATTGAATACATTAAAGGCAGCCAGAATTGCATACCAGATTTTCTGAC

CAGAGAATTTTTGCAGGGGAAAAATGAGTACTAATCCTACTCAGCAACGAAGGCTCCCAG

CCACCCTTTCCCAAACCCCAGCACCAGTAAAACAAGAGTCTTTTCCCGACTCTTCATCGG

CCCTCGCCATTAAAAACAGATTCACTCCATTAGGATCCACAGTAGGAAATATCCGACCAA

ATTACCAAACAGCTTTAGCTACTCCTTTTGATCCTTACTCTTCAGTACGTTCTCCAACAC

CTTCACAACCCAAAACACCTAGCTATGCTAAAACTTCTCCATATATCCAAAACTCCACTA

CTGAAAAGCTTTTTTGCATACCTCTTGACATTGATAAAAATCAACCTCCAGAACGCATTG

CTAAACTGCTTTTGCCCCCAAATTCTCACTATCTTCAGCAGCACCATACAAAAGCCTCAA

ATACTACCAGGCTATTCTCTCTGAAACCGAAAGCATTGCTATTAAGCCAATTTACAGCAC

CACTCACCAAAATAAAATTTTATACCATTCACTTCATATTGGAAAATTTCTCATTGAACA

AGATTGGGGTCTCCCCCTTTATCATACCAAACCCCTTCACACCCAGCATGTTCTCATACC

GAACAACAATTACAATTATTATGATTACATTCAAGCTTAGACTAATATTTTTCTTCATCA

GACGGAAGATTTTAGTCATTCATGGTTCGTGACCTTTGATAAAAGTTTCAGATTGAGATT

CCCAGCATGGTTTCCAGACTGGTGGGCTACTCATGGCCCAAATATTTCCCTTGTCCTTGA

AGATCTTCGTCAAGCACTAAACAATAGTTTCAAGACTTGTTTTGACCGCAGCCGTTTTGA

CAGCCGGGTTTCCTTATTCTCTTTATTCATGGCCAAATACAAAGTTCCATGGATCTTAAA

ATGGAATTTCCAGTTAGACAACGACGGAATTTTCAGGACCCGGTGGGTGAAATGGTGGGA

CAAATTCAACTACCAAAAGATTATTGATCTAGTTCATGCTGAATTTTCCCAGAGTTCGTC

CCCAGAAATTGCTGCATCTTCTTCGCAGGCCTTGCCAGACACTCAGCAATTTCCTGAACT

CCTGAAAGCTCAAGAACCAGTTCAATCTTTTTCCGACATTAGTCCATCCTCCTCCCTCAA

TGGAAAGACCAAGTCAGCCAAATCCTCTAGCTCGAAGAAAGAAAAATCTTCAAAGACTAC

CCAGCTTCTGGACTTGGCCCAACAGCTCATGGCCGAAGTAGCAATGCTCCAAAAAAAGAA

AGATTCCGACTCGGATTCTGACGCTTTTAATGCAGGGTCTCAGCCTCCTGCCAATTGGGC

TGATCAAGTGGACCTTCAAGACGCACAAGATCCATTCGATTTTTGAACTGCTTTATTTGC

CCATGTGATTGTGTCAGCCAAACAACTTCATCAGCAGTAGCTGCTATCATTTCTTCCGCA

TAGACAAGGCACTATTCTTCAATAGTGAAAAACAAAAAGTCACTGTTCATTCAGCTTTTG

TAAAAGTAGCAACGTGATTCCATAGTAAAGGCATCATCAACAGTAAAAAACAAAAGGGTC

ACTATTCATTCAGCTTTTGTAAAAGCAATAACGTGACTCCACAGTAAAGGATCATCAATT

AGCCAGCTTTGTCAGTTCTCTTCTCTTACCAGCGGGATTCCTGGATGAAAATGGCATTAT

TCAATCATTCTCCTCCCTCTATCAAAGAGGAATTCATCTTCAGAAGAAGGACACGGGTTT

TTAAACTCAATAGCTTAGTTCAATATCATCTCTCTGCTTATCCACAAAGGACAGAGATTA

TTTTCCAAACACTTGTAACACCTATATTATTCA

>MescV [endogenous-virus-name=Manihot esculenta virus] [host=Manihot esculenta] [moltype=genomic DNA] [complete genome] 7676 bp

TGGTATCAGAGCCTTGGTGAGAACCCATTCTTAATTAGTTCTAGTGAAACCCAAGTCGCA

TATCCATTTGCCAAATAAACCAGAAGGTATAGGCATGTTTGGAGTAAATCATTGACAGTG

ATCTAGAGCTAAGATGGAAAGGCTGTTTGAATCTCTCTCTGGTAGATCTAGAGGTTCTAG

CTCTTCTAAATCCTTGTCTAAAAAATCCACAAAAAATAAAGAAATTTTGTTAGCCCAAGA

TCTAGATACTAGTTTAAATAATTGGGAGTTACCTCCTATTTCTTCAGATCTTATTTATAA

ATCTAATTTCATGTATAAATCTGATTACGTTATTAAGACTGTAGAACAAGCAACACCAGT

ACATGGAGACATGCAGTCTCTAAGTTTGCTCTCTGAAGAATTGATCAAAAGGCATCAAGA

GAAATATTCATTCCTTCATATAGGAATGATACAGGTAGCGGTAAAACCAGCTACAAGACT

AGGGCTTAATACAACCGCTATGTTGTGCGTTCGAGACAAAAGGCACTTCAAGTTTAATGA

CTCCTTATTAGGAGTAGTAGAGTCCTCCCTTTGTGATGGACCCATATTCTTTCAGTGCTA

TCCAAATCTTACCTTGTCTCTCACAGATCCGTACATCTTACAGACTCTGATCTTAGATAT

CAAAACAATGGGATATGATATGCTGCCAGGATCAGAAAACTTAATCCTAATTTACAGGAT

TCATTATAAAGCTATGAATACTGTAGTCCCTAATTTAAGGGAAAAGGCTACTAAGTTAAT

ATCCCCAAAGGGAACAACTACTCTGTTCGTGACTAATATGTCAAAAGGGAATTTAATAAT

TCCAAAGTCCATTCAATGGGATCAGGTTAATTTGCCTGAAAGCTGGATACTTGAAGAAGC

TGCACCACCAAAGAAGGAAGAATCGACTACTGTTCAGTCAATAGTACAAAACAATAAAGG

ATCAGTAGCCATCTCTTTCGCCAGAAGCAGAAGTTTTGGAGGAAGAAGATCCTACTCCAA

TGCATCCCTACTATCAGAATCATTACCTCCAAGATCTTCAGTATCAACTCCTATTCAAAG

AAGCAACTCTGTGATTGGAGTCCAAAGGACTGAAGAACAAATAGCAAATCCAGTTTATGA

AAAGCCTCTAAGCCCAACTCCATCCGACATGGGCTATGACACTGAAAGTGTGATCTCCAG

ATCCTTCAAAATCATGATTCTTGAAAAAGAAGAAACGCCGTTCGAAAAATGGTTCAGTAT

GGAAATTTCCAACAAAGAGCAAAAGCTGTGGCGTAGCAAATACAAAGTATACAAGAATCA

GAAACAGGGGAGTAAAGACTTTCCTGAATTTATACACCACTGTTTCACTCTACTAAAAAA

ACCATACCCCAATTTTGAAGACATTTCCTTTCTGACCACCCTTGAGACCAACTACAAGAC

CTTCATCAATCCCCATGGAAAGATTCTCAAAGACATGCATCCACCCTTAACAGACCTTAT

CTACCAACCCACAGAAAACTCTAAAGTGGAGTGCATCCCCTTTTCACATGACAATAGTGA

AAATCCTGCTGCTCGGCAACAAAACTTCACCAATCTAAGCCTTCATACAATTGGGCAACA

GCTGAGCAGAGTTGAGAATCAAGTCTCAAAGATAGCAGCTCAACCATCAGGAGTAGACAT

TCTTCCACAAAAGGAAAAGGCAGAATCATCAGGCACTAAAATGGAAGAAAGGGTTTTATT

TAAACCCATGGATAGTAAAAGCATCAACATCAAGTTAGATAAAAAAGAAGAAATGCTGGA

AGAATTAACCAAAAGATTGGCTAAACTGGGACTAAAAGAAGATACAAAGAAGAAATCGAT

TGTTCCATTAACTATGGAATCTGAGAACGAGACAGAAAGAGAAGAAAGGCAAAATGAAGA

AGAACTTACTCAGCTCGAATCAATGTTACGAGAAACAGAACCAGCTGAGGTAAATAGGAT

AAAATATCCTAAAGCACAGGCCACGATGGATTTGAAGCCATACTATCCCAGGCCATCTCC

AATTAACTTGCAGTATGAAGATACAAGCTATAATCCGGTGCAAGTAGATGGATCCTCCAT

TATGGAATGGAATATTGATGGGCTATCTGATTATCAAATCAAAAATGTCCTTCAGTACAT

GACCATGCATGCTACAGCATGTCGAGCTAAGGGTAATGATGACCCTGCCGCTGCAAGAGC

CCTAATAGCAGGGTTTAGTGGCCAACTAAAAGGATGGTGGGACTTCTCAGTCTCCAATGA

AGGAAAAGCTCAAATATTCAATATGGTTAAACAAGAAGGAGCCCAACAAGTACCAGACGT

AGTCAACACCCTATTATATACAATAGGGTTACACTTCATCGGATCAGTCAGCATGTTTAC

TGACAGAGCGCAAGAGCAGTTAATAAATCTGCGATGCCCAGATCTTTCTCATTTTAAATG

GTATAAAGATACCTTCTTCTCTCTGGTCTTTACCAGAGAAGATAGCCAGAATAATGTCTG

GAAAGAAAAATTCTTGGCAGGACTTCCCACACTCTTTGCTGAACGAGTACGAGATCAAAT

ACGAAGTAAGCATAACGGTAATATACCGTACCATCAGTATACATATGGAGAACTAGCCTC

TGAAGTAGTTACTGCAGGAATCATCCTTTGCAATGAATTAAAAATTCATAAGCAAATGCA

AAAGGAACGGTTCTATGGAAAACAAATTCTTGGTAACTTCTGTGAACAGTATGGATTACC

ACCTATAAAATTTCCCTCCACGAAATTTAAGGGGGGTAGGAAAGAGGACAATCACAGACA

CCGGCACCGACGAACAAAACGCTTCTACAAAGACAAACGTCCTTACAAAGAAAAACATAA

AAGATTCCAGAAGCCAGAAAAGAGTCATAAGCCAAAAGAAAAAAGGAAGCCTAATCAAGG

CAAAGCAGAGAAAACTATAGTCTGCTACAGATGTGGAAAAGTAGGACACTATGCCAACAA

GTGTCGAGTGAAGCAGCAAATCCAAGCATTAACGATTGAAGAAGATTTAAAAGAAGCATT

AGCAAAAATTTTGCTAAATGAAACGGATTCGGAGCAAGAAGTAATGGCACTCAATGCTCT

CGACTATACAACAGAAGAAGAAGAAAGTAGTACAGAAGAAGATGAGGAACAGAAAGAAGA

ATGCGAAGGAAACTGCGATTACTATAAGTCGCTATGTGCCATGAATGGTCTCTTGGTACT

AACAAGAGAAGATAATCTCATTCTGGATCTAATTGATAACATTGAAAATCCAGAAAAGAA

AAGAGAGAAGCTGGAAACTTATATCAATCTTTATAAAGATAAAGATTCCAGTACATCCAC

ATATAATCCGATAGAAAAGAAAATTGATAGCAAGCAGTCTCCTTATGACCTAAAGGAGAT

CTTAGAAAGAGTTAAAAACTCAAAGAGACAGAAAGAACCAACAGTGGCAGAATTAAGATC

GGAACTCAACTCGGTAAAAACTGAGATCAAAGAGCTGAAAGAAAGGATAAATATCCTAGA

GCTGTTAAATGAACAGCAACAATTAGCCATAGAAGAACCAGAAGAAGAACAGTCGAAAGC

AGAAGTCAAAGATGTCAACAACCTCCATTATATCAACATGACTGACAGGGTAATTACCCA

TAAGTGGCACACAAAGATAACGATAGTGGTGCATAAGGAATACTTATTTGAGACAAATGC

ATTAATCGACTCTGGAGCTGACTTAAACTGCATTAATGAAGGATTAGTCCCTTCCAAATA

TTTCTCAAAAACAGTTGAGGAATTGCATACTGCAGACGGAAGCAAAATGACAGTAAGGTA

CAAATTGAAAGACACCGCTATCTGCAACCAAGGTATTTGTTTCGAAATCCCTTTCCTAAT

GGTAAAAGGATTATCTCATCCTGTTATCTTAGGAAATCCTTTCTTGCATATGCTGTATCC

TATTCAGCAAGTAACTAAAGAAGGAATAAGTACAAAGATAAATGGAAAGGTAATTACCTT

CCATTTCACCTCTCAACCAAAGGTAAGAGAGATAGATGTGCTAAAAAGCACTGTAGAAAG

TAAAACTAAATTTATCAACTCCTTAAAACAGGAGGTAGTCCACAAAAGTATAGAAGAAAG

GTTAAAAGAGCCTAAAGTCCAACAAAGGATTAAAATAATCCAAGAAGAGATGCTAAATAG

TATTTGTGCTGAAAGCCCAGATGCATTCTGGACTAGGAAAAAACATGTGGTAAATTTGCC

ATATGAGCCTGAGTTCACTGAAAAGGCTATCCCTACTAAAGCTAGACCAATAGCTATGGG

ACCAAGACACCTGGAGATTTGTAAAAAAGAAATTGCTGAGCTAGAAGCAAAAGGGCTAAT

AAGGAAAAGTAGTAGCCCATGGAGCTGTCCAGCGTTTTACGTTGAAAACGCTGCCGAATT

GGAAAGAGGTGTCCCTAGACTAGTCATAAACTACAAGCCCTTAAATAAGGCGCTTAGATG

GGTTCGATACCCTTTGCCTAATAAAAGAGATCTGTTAAACAGACTCTATGAGGCCACTAT

CTTCTCGAAATTTGATATGAAATCTGGATATTGGCAAATCCAAATTGCTGAAGAAGATAA

GTACAAGACGGCATTCACAGTGCCTTTTGGGCATTATGAATGGAACGTCATGCCATTTGG

GTTAAAAAATGCCCCTTCGGAGTTCCAGAAGATAATGAATGAGATCTTCAACGCTTATTC

AGCGTTTTCTATAGTCTATATAGATGATGTCTTAATTTTCTCTAAGACTATAGATCAACA

TTTCAAGCATTTAAAAATGTTTGAAAAGATCGTTAAATTAAACGGTCTAGTAGTCTCAGC

AAAGAAGATAAAAATCTTCCAAACCGAAATTCGGTTTTTAGGACACAACATTGCTAAGGG

AACTATTATTCCTATAAATAGAGCTATAGAGTTTGCTAGCAAATTTCCAGATGAAATAAA

AGAAAAAACTCAGCTGCAAAGGTTTTTAGGAAGTCTGAATTATGTAGCCGATTTCTATAA

AAACCTAGCCCAGGATGCTAAGCCTTTATTCCAAAGGTTAAAAAAGAATCCTCCTGAGTG

GACCAATGAGTGTACCTTAGCAGTAAAACGGATTAAAGATAGGGTAAAGACCCTACCATG

CTTAGCCATTCCACACCCTGAGTCATTTAAAATAGTTGAAACAGATGCGTCTGATAAAGG

GTATGGAGGAATCCTTAAACAAAGGATTAATAATAAAGAAACCCTAGTAAGATTCACTAG

TGGTATATGGGTAGGACCAAGAGTAAATTACTCTACTGTAAAGAAAGAAATTCTTTCAAT

AGTCCTATGTGTTCAAAAATTTGAATCTGATTTATTAAATCAGAAATTCCTAATCAGGAT

TGATTGCAAAAGTGCAAAAGAAATACTCCTTAAAGATGTTAAAAACATCGCCTCCAAGCA

AATCTTTGCTAGGTGGCAAGCTATTTTATCTGTCTTCGATTTTGACATTGAATTCATAAA

AGGAGAGACAAATTCATTGCCTGATTTTTTAACCAGAGAATTTCTTACAGGAAATGAGTT

CAAGGACTCCGGCCAAAAGACAGCATGAGGAGTCACCACCCCCACCGGTTAAAGAATCGG

ATCGGGTCAGAGACGATGCTTATAAAATAAGCATTAAGCCGGTAAATGATAAAGAGGTAA

ATTACCCTTATATCCCTATCAGCTATTATGTTCTAAATCCTATTGAAAAAAGATATGAGA

ACTTAAACCCATATGAAATAGCTGATTATTATACAGAACACCATTCAACAGTACCATTTC

TTCCAAAGGCTTTTGATTATTATCAAGCCATACTCACAGAAACTGGATCTGTTGAAATTG

CACCCACTAAGAAAACTGGTCATCAATGGGCTTATAGTAAGTTCATTATAAAAAGCATCA

TAACCAGTGAACAATGGGGTGATTCTCTCTTTAAACAAAGAGAGTTGAGATACGGGAAAC

CAAGGTGGTATAGTTACTTTGATTATATCCAGGCCTGGACTGGAGCTCTGTTATATGAAA

ATAGACAGAAAAAATTCAGTTGGTTTATCCAGTTCCAGCTTGAAAAGGAAATTAAAGAAT

TTCCTCCCTGGTTTCATGAATGGTTTTTCAATTGGGGAATCTTTCCAATAATTCTACCAG

ATAAGGTAAGGACTGTTTATGAACAGTTTGCAGAGATTAATAAATCTCTACCAAATCTCA

GACTTGAATTTACAATACGGTATAAAGTACCATGGATCGCCAGATGGGATTATATATTAA

CTGCCAAAAAACCCAAAAAATTTAATGAATTTACCATTAATAATGTAAAATGGTTAACAA

GGAGGGTAATGATAAAATGGTGGGACAAGTTCGAATTTTTTGGTAATTTTTCAGTATTTC

ACCATATGAGAATTATCCACCTGGTTAAACAACCAGAAGAATCTTCTGAAAAGTCAAAAA

TGACTCGATTACTCAAGGAGTTATTTCACTCCATTCCACAAGAAGAGCTGAAGAAGCGCC

TATCACTAGCGCTTGAAGAATTAAGCGACACTGCAACAACAAAAGAAAAGCCACTGGAAA

AAGGAGAAAGCTCCAGGACAAAGTTCAAGCGCACTAAAGAAAGTAGTGACTCAGATGACG

AGGACTTGCTGTCAGATTCCCTCTTTCAAGATAGCCAGGACCCAGACGAGCCAAACGAAG

CCACTTGGCCTTAGTGGACACCGCACTACTAAAAGCAGATTCCAAGAAGATAAGAAAGAA

AATAAAGACAAAAACCCTGACGAACGCCATGACGACATCAAGATTCCTTCAGGCTCTATA

AAAGGATGGTTAAGCTCAGAGGCAAACAACAGCAAAAAATCCCAAGAGAAAAAAATTTCT

CTCTGTAATACTAGTTTAGAATTCCTTTGTAATCTCTCTTGTACTTATCCTTTTCATGAA

TGAAAAATTCATTTCCTAAGTTTGATCACCGATCTCTTGAAAAGAAACTATCTCAAATCT

CTCTAAAATCTGAAACAGCAAAAATATACCGAAAAAGAAATCTTCCTGTCAACGAGTTAA

TTGGTGTGAAAGACACTCTTGGAGAACCTTCTGGAAAATTTGATTATCTGGTCTGGTATA

CTAAACTAGAATCTGCAGAGACTCCTTTCTCCGACATCATACCAACAGGTTGGGGAGAAG

AATTCCAGCAAGAAGATATTGTAAGTTATTTATTTTAATGTCAGATAATATCTTAAGTGT

CATCAAAAGATTAATTTTAATAGATCTAACAAATAGACAAGAGGTATTAAGGTTTAAAGA

AGAATTAGTTGCAGAAGGTAGAGAGTTATATAATTACCATGTTGTTAACCATAATGAACA

CTGTGACTGTAGAGGGTATCAAAGAACATTAAGATTGTTTAACTGCATCCTTAATTATTT

ATATCAGCTTTTGAGAAGTAATAATTTTTTTAAAAAAATTCTTACACCACACTTCTCTGG

TATATCGACTGGAAAACCAGACTTCCTTAACCCGGCAATAGCACTTTATGCCGTAGAGGA

GACATGGTTGACAACGTCACGTACCATTGAATTATAAAATAGTTGCCTTAATTGCATAAT

AGTCGACTGAACTAGTTGTTGCATGCGCAGGAAAAAACAAGATTTTTGTTTTCAAA

>MgutV_sc1 [endogenous-virus-name=Mimulus guttatus virus] [strain=sequence cluster 1] [host=Mimulus guttatus] [moltype=genomic DNA] [complete genome] 7228 bp

TGGTATCCGAGCCATTGAATTGGCAGGTATGTAGTACTTTATGTAGTACTTTTAATAACA

GTTTGTATTTTTTAATAACAGTTTGTATTTAATCTATTTGTGTTTATCTGCTTTCTTAAT

TTTAAATTATATCTACTTTTTAAAGTTTTATTTTCCTTTCTTTAAGAGGGTTGTGAAACC

TTAAGTCGCGTTGTCCTTGATATTCCCATTGTTGCTAGATTTAGGCATTTAGGGGATTTG

TGACGTAAGTCGTTGACAGTGATCAACAACCTTATGGAGAGGATAAGTAGATCTTTATCA

AATAGATATAAAGCAAATCATACTAATAGTAGTTCTAACAAAGAAATTATTATTTCTGAA

AATATTGATAATACTCTACAAAACTGGAAATTACCCAGCTCATCATCTGTTTATAAGCAA

AAAGGAACATTCGAATTTACTTCTGATTATATAATAAAAACTGTTGAAGAAGCTATGCCT

TTAACTGAAGGATATAATGCATTTCAACTTTTATCAAGACAATTAATTGAACAACATAAA

CGAAAATATAAATTTCTTCATATTGGAATGATACAAGTAGGATTAAAACCTGCAACCAGA

CTAGGACTAAACACGTCTGCAGTCATTTGTGTCAGAGATAAAAGACATAACAAATTTCAT

GATTCCTTATTAGGAATAGTTGAATCTAGTTTATGTGATGGACCAATTTATTTTTCATGT

TTTCCAAACTTCACTTTATCCCTCACAGACCCAACTTTAATGCATGCCTTATGCTTAGAT

ATTAAATCTGAAGGATTTAATATGATGCAAGGAGCAGAGAATATAATTCTTATTTATAGA

ATTCAATATAAAGTTATGAATACGGTAATTCCCCGTATTAAAGAAATTCCTACACAACAT

AGGGGTTATACAACTCTATTTATTACTAATTTAGCAAAATCCAATTTAAAAGTCCCAAAA

ACAATCACTTGGGACCAAGTCAATTTACCCGAAAAATGGGTACTTGAAAAAGCCTCTGAA

CCTGTTAAACAGGAAAATAGAGAATTAGAAGAAATTATTGAATACCCAGATGGAGATGTT

GAAATTAAATTCTCTAATCAGAGAATTGCTCACCTTAATCTAGGACGAAAATCTACCTCT

TCAGTTCCTATTTCACAGTTTGAAAGAGATAACTTGTTAGGAACAAAATTCACTACAGAC

GGAGTAAATCAACCAGAATATAAGGTTGAATTACCACCCTCAACATCTGGAACTTCCAGA

AATATTCCTAAAAGGCAATCACCTACTCCATCAGACATGGGGTATGATGATAGAAGTGTT

TATGGTATTAACACATTAACAGTTGAAGAAAAAATAGAATTAGAATTTCCGATTCAAGGA

CTTGCAAAAGAATTATTTTATTCTCCTGAAAATGAAATTAAAAGAAAATGGTTCTACTCC

AAAAAGAATTTTGAAGGACAGAAAAAATGGTTTATGCTATATAAAAAATTTATTAAAAAG

ACAGAAGAAATAATTGATTTCTTTGAGTTTTTAAAAATATTTTACCAAAGTGAAAAACGT

CACTTTCCTCAATTTGATGATCCAAGATGTATTTCTACAATTGAATCTACTTTTCAAAAT

TACATTACAAAAAGAGGAACACTTGTTAAAAGTATTCATCCCCCTGCAGCAAATTTAACT

GTTCAAATTGACAAAGACGAAGTTATTGCTACACCATATAGGAAAGGTAGTACTATCGAA

GCTGTTGTTGAACAGAACAATTTTACAAATCTCTCTCTTCAATCAATTGGTAATCAATTA

AACCGTATTGAGCAAAAAGTAGCTTATACTGAGTCAACATCCAAACCTTTACAAATAGAA

CAAAAGGTTTTGTTTAAACCAATGTCAACAGAAAAGACTGAGTTTAAATTACAAACTCAA

GAATCATCTGAAATGGTGGATGAAATTGTCAAAAGATTAAAAAATCTTGGCATAAAAGAA

AAAGAAATAGCTCCTCTGGAAAAGGAATCACAAAAAGGAACTGACTCAGAAGATGAGCAA

GGTACTGATGACCAAGTAAATCAATTACAACGTATGTTTGAAACAGAACAACAAACACCT

TATCAGGTGAATAAGATATCTTCTTATAAAAAGAAGTTAGTTTATAATATTCCAGCAAAA

CCCTATTATCATAGACAAACTCCAGTTGACCTTCAACTGGAACAACATGATGATTATATG

ACTGTTCAATTCGACGGTCGATCTATTAATGAATGGAATATAGATGGTATGTCAGAATAC

CAAATCCAGACAGTCATACATTATATGACTATGTTTGCCACAGCATGTAAATCAAATGGT

AATTCAGACCAAAATATTGCAAAAATTATAATACAAGGTTTTACCGGCCAACTAAAAGGT

TGGTGGGACTTTTACCTCCCTGAAGAAGCTAAAGCTCATATACTTCAGGCAGTGAAAACT

GAAATGGTTAATAACCAACCAATTAATACTAATGATAGTGTTAACACATTGTTATATACA

ATAGCCAAACATTTCATAGGCACTACATCCTTATTATTGGATAAAACTCAAGAACAATTA

ATGAATCTAAGATGCCACAATTTATCCCAATTCAAGTGGTATAAAGATGTATTCTTTTCA

AAAGTTTTTACACGAGCAGACAGCCAACAAGATTTTTGGAAAGAAAAATTTCTTTCCGGA

TTACCACACTTCTTTGCTGAAAAAATCCGTAACAGAATTAAGCAAAAACATAATGGAGTT

ATTCCTTACTCCCTTTACACTTATGGTGATCTTGCTTCTGAAATCACATCAGAAGGAATA

AATCTATGTAATGAAATAAAATTACATAGACAAATTCAAAAGGATAAAATCCTAGGGAAA

AAGATTATAGGAGATTTCTGTGAACAAATGGGACTACCACCTATTCGTTCAGAGACATCT

GAAAAGAAAAAGAAAACTAAAATTAATAAGTTTTCTAAAAATAAATATTTTCCCCTGAAA

GAAAAGAGAGAAAAGAAAAGGTCAAAAGACCACAAACATGGGACTAACAGTGCCCAACCA

TCAAAGCAGAAGACAACACCCGTCTGCTGGCGTTGTCATAAGGTTGGCCATTATGCTAAT

AAGTGTAAAATGAAAAAGAAAATTAATAGCCTATCTATTGATGAAGGGCTCAAAAAGACA

TTGGAAAAATTATTCCTTTCTGACTCTGAGGATGATAAAACTAATCTTCAAGTATCTCTA

ATAGAGAATGAAAATGAAGAATATGAATCTGATTCTAACCAATCAGAAATAGAATCAGAA

AAATCTGATTGTGAAGGAAATTGCGACTATTATAAAACCTTATGTCAGGCTAACGGATTA

TTTGTTCTGACAAAAGAAGACAATTTTATTCTTGATATTATAGATAAAATTACAGATCCT

ACAGAGAAAAGAGAAATACTTCAGAAATATCTGGAGAATTATTCCAAAAAAGGAAATATT

GATAAAGAATTTCGTTTTAAAGAACCCGAAGCTTATAACATAAAAGAAATTCTTAACAGA

GTAAAAAACTCTGAAGAAAAACCCGAAGACACATGTATTAGTGAACTAATATATGAAGTC

AAAGAACTAAAAACAGAATTACGCTCTCTTAAATCTAGAATAGCAGTATTAGAATTAAAA

AGAATTCAGAATATTTCTGACCAATCAGAAGAAGAGGAAGACTTAGAACCTAGTCCAACT

ACTTCTCAAGAAAAAGATGGATTAAATTCATTAACCTATATTAATATGATTGACAGAGTA

ATCACTCACAAATGGCATATCAAAGTCACCATAATTGTCTGGAAAGAATATTCCTTCTCT

GCAATTGCTATGGTTGACTCTGGTGCTGATCTAAATTGCATCAATGAAGGACTTATACCT

TCTAGGTACTTCTCAAAAACCTCAGAAATTCTGAATGCTGCAGATGGTAGAAAACTTATA

GTAAAATATAAGTTACAAAATACTGCAATTTGCAATAATGGAATTTGTATAGAAATGCCA

TTTATAATGGTAAAAAATTTATCCCATGATATTATATTGGGAAATCCTTTCTTACACATG

CTCTACCCTATAAAAAATGTGGATGAGAAAGGTATTACTAGTATTGTAAAATCACAAGAA

ATAACATTTCAATTTACTACACAACCTCGTTATCACGAGATTGATGTTTTACAAAAACAA

ATAAAAAGAAAAGAAAGTTTTTTAAATTCACTTACAAAAGAAATATCTTATAAAAAGATT

GCTGAGAAAATTCAGCAACCAGATATACAAAAGAAAATACAAGAAATACAAGAACATATT

GTTCAAACAATTTGCTCAGATATTCCCAATGCCTTTTGGGAAAGGAAAAAGCATATAGTT

CATTTACCATATGAACCAGACTTTAAAGAAAGTCAAATCCCAACTAAAGCTAGGCCCATA

GCAATGGGACCGGAATATTTAGAAATGTGTAAAAAAGAAATTGAAGATTTATTAAAAAAA

GGACTAATTAGAAAATCTTATAGTCCATGGAGCTGCCCAGCATTTTATGTTAACAAAAAT

GCTGAACTTGAGAGAGGAGTTCCAAGGCTTGTTATAAATTATAAGCCATTGAACAAAGCT

CTTCGATGGATAAGATATCCCATTCCTAACAAAAGAGATTTATTAAACAGACTTTATACA

GCCAAAATAATGTCAAAATTTGACATGAAATCTGGCTTCTGGCAAATTCAAATTGCAGAA

GAAGACAGATATAAAACTGCATTCACTGTTCCATTTGGACATTATGAATGGAATGTTATG

CCTTTTGGGCTAAAAAATGCCCCATCTGAATTTCAAAATATAATGAATGATATTTTTAAT

CCATTTACACATTTCTGTATAGTATACATCGATGATGTATTAATCTTTTCAGAATCAATA

GATCAACATATCAAACATCTCAAAGCATTTATTAATGCAACTACAAAAGCAGGACTTGTT

GTATCTGCAAAAAAGATAAAATTATTTCAAACCGAAACTCGATTTTTAGGGCATAATATT

AAAGAAGGGACTATTATCCCGATTGAAAGATCTATTACTTTTGCGGAAAAATTTCCGGAT

GAAATAAAAGACAAAAATCAATTACAAAGGTTTTTGGGCAGTTTAAATTATATTGCTGAT

TTTTATACTAATCTTGCATTTGATACCAAACCTTTATTTGAAAGGTTAAAAAAGAACCCT

CCAGAATGGACACATGTTCACACTGAAGCAGTTCGTAAAATTAAAGCAAAAATTAAGGAA

ATTCCATGCTTGGCTTTACCTCATCCTAATGCATTTAAAATTGTCGAAACAGATGCCTCA

GACTTGGGATATGGAGGAATCCTTAAACAAAGGATTAATAATAAAGAAATTCTTGTTAGA

TTTACATCAGGAGTTTGGAAAGGACCCCGAATAAATTATTCTACTGTTAAAAAAGAAATC

CTTTCAATTGTTCTTTGCATACAAAAATTTGAAAGTGATTTGCTAAATCAAAAATTTCTA

TTACGAATTGATTGTAAATCTGCAAAAGATATACTTCAAAAAGACATAAAGAATGTTGCA

TCAAAACAAATTTTTGCAAGATGGCAAGGCATATTGTCCGTTTTTTATTTTGATATTGAG

TTTATAAAAGGATCAGATAATTGCTTACCTGATTTCTTAACTCGTGAATTCTTGCAGGGA

CAGAATGAATAAAGACAAAGGGAAGGCAAAAGCCACTGGTGAACCTTATAAGGTTCCTTC

ATACAAAAATGTTGTGGGAAAAACCCCAGAACAATTAAGAAAAGAAGCCTTAAAGGCCAC

GCCAAAAGAAAACAAACTTAGAGATGATGCATATGCTATTGCAGTATCGTCTAATATGAC

GAATGAATTCGTTTATCCATATCTTCCTATTTCTACAATATGGATCGCACCAATAGAGCT

TAAATATGCTCATCTTGCTGCTAATGAAGCTGCTAAATTCTACCTTCAAGAATTTGCAGA

CCCATTTCTTTATCCTAAGGATTACAATTTTTATTATGCAATACTTGACTCTACTGGTTC

TGTAGAATTTGATTCTGTTAATCGAACAGGTGGTGAATGGGCTTATTCCAAAGCTTTCTT

CAAAAAGGTAATTACTCCTCAACAATGGAATGGAGACCTTTATAGATTCAGAGAACTTCC

TAAATATAGAAAGGAACAAAATAAATTTTGGTTCAACTATTTTGATTATATCAAAGCTTG

GGATGGTGCTTTCTGTTATGAGAACAGACAAAGAAAGCACAGTTGGTTTTTCCAATTTAG

AGATTTTGATCAAAGTTCTCCTTTACCACAATGGTTTTTAATGTGGTTTAATAATTGGGG

ATTTCATCCTATTACTCTTCCTGATAAAATCAGAACAATTTATGATGATTTTATAAAAAC

AAATGCATCATTAAAAACTCCAGGATTAATGTTTGCAATCCTCTATCAAGTTCCTTGGAT

TATGAAGTGGGACGTTATTATTTCCAAAATGCCCCTTCGTAAATTTGGAGAAATTGTTCA

TAATATCCCTTATCTGGGAAGAAGGATTTTAATCAAATGGTGGGACAAGTTTGATTTCTT

TGCAGAAAATTCAGTCTTCTCCCAAAAGGGATTAATTAATCTAGAGCCTCTACCACCTCC

TCCAAGTCCCAAGAAAGACATTACAGAAATTCTGAGGGAATTATTGGCATCGTCATCCAA

ATCGGATCTTAAGGCACAATTAAAAGAAATCCTACAAGAATCTGATTCGGATACAGAGGA

CACAGATATGGCCAGCCTTAAATCAGACTCAATGCAAGACTCCCAGGACCCATATGATGT

TTAGAAAATTGCATATGTCATTTTCAGGAATCCATAATAATGGAGTTACTGTGCTACCCA

CTACTGGCCACAATAATGGAGTTACTGTGCAAACCACTACTGACCACAATAATGGAGTCA

CTGTGCTACCCACTACAGAGTCCATGATGGAGGTCACAATCAGCTACTATTCATTTAAAG

AAAATGAAGAAGAATTTGAAGTCCGGAAGAAGACCTATATAAAGGCATACATCCATCACA

AGGAGGGCATCCGAAAATTTCCTAAGATCTTATTCCTTCCTTTGAAAAGATATTCTCCCC

TATCTGTTTTATACCCGCTGCTTTCTCTAAAATAATATCTAGTATTTTATTATAAGTTGT

ATAATATTGGTGTACTGAGGGACCTTA

>NbenV_sc1 [endogenous-virus-name=Nicotiana benthamiana virus] [strain=sequence cluster 1] [host= Nicotiana benthamiana] [moltype=genomic DNA] [complete genome] 7887 bp

TGGTATCAGAGCCATGATGGCTGTTTATGTGTTTAACATGAGCAACTAAACACATATACT

GTCCGGAGCTCAATGCTTTTGCAAAACAAATCCATGACTAAGTTATTATGCTAAATACTT

GAAGTAGTATGCTTAAATTATTTATAAGTGTTAAATCTTTTTATCTTGTTTTATAATTGA

TCTACTTTGTGTGAGGTACTTATTTCTCTCTAAGTGTGTGAGGTATCCGAGTAATCCCCG

GAGCATACGTTGGCAAGCCTGTCCCTGTGGTCCTTGTAAGCCGAGTTAGGCGTTTTGTTG

AGACCTTTAGGTGTTCCCGTAGCCAAGATAACTCTAGAGAGAGAAGAGGTAAAGCCAAGA

GAACATGTAGATCCTTATGAACCTTTGATAATTAGATCTAAAACTTATTAATAATTTGGC

TTTTGTAATTCTTGTATTTGCATAATCTGGATATATTGTTTGGATACGTTGTTTTGCATT

AGTATAGGTTCCAGGACAGTAATTTTTTGAAATGAATCAAAATATTTTGAATACTGTTAA

CTTAACATCTCGTGCTTCTCAGATTGCATTAACTGAGTCTAATTTGGATAACTGGAGTAT

CCCTGAAGAACCTTTTCATAAGATCTACCAGATAGGTCATTTTAATTTTATTAAGAAGCA

TAATATTAAAACATGTGAATCAACTGTTGCTATTAATAACTCTTTAGAAGTTATAAAATT

GTTAAATGAGCAAGATATAATTAGATATAAATCTAATTTTAATTACTTGCATATTGGTCT

TGTTCAAGTTGCTGTTAAGCCTTTGTTTAGAAAAGGACTTAATATTCCTGTTTGTGCACT

GTTACGTGATGCACGTTTTCTTAATTTTGATGATTCTTTATTGGGAGCTCTTGAAAGTAA

TTTAGCAGGTGGCCCTGTTTATTTCAATTGCTATCCTAATTTCTCTGTTGATATTAATGA

TCCTAATGTGTTGGATACATTAACTCTTAATATTAAAACTATAAATATGAACAGCAAATC

CGAAACTCGTGAGTTAGCTGTTATATATCGAGTCTATTACAGGTTGATGGCAACTACCCT

TGCTCCTAGAGCTAGGATAGAGAGTACCAAAGGTGTGACTATGTTAATGGAAGCTAACCA

TGAACATAGTACCGTCTTTGTTCCTCGCCTTCTCAAGTGGAATGACCTCCTGGCCAATAC

AGATTGGCATTTCGAGGCTATTACCCAACCTGTTTCCTCTCACACTGAGAAACGTCAGAT

TGAGCGTATCATTCAAAATTCCGATGGTTCTGTTGATCTTAAATTTCTGGACTCCAGACC

TCGCAGAAGTTTCACCGCTGGTTCTACTTCTTCAAAACCCCCAGAGGGAGTATTTAATCG

TTCTTATTCCTTTAGGTCAGCTGACAAAAGTCAAGTTGATGGTGTAGATTTTTCGGGAGA

AATCCCAAAAGTGTTTTATAAACCTCAGGTCCAGGAACCTTCTAGTCCCACAGCGTCTGA

TATGGAGCCTCCAAGGCAATATTCAGACCCTGGATTGGGTGTTATTACTACAGATGAGGA

TTATACCCCTGATCTTGCTTCTTTGAAAAAACTCTGGCTTGACCCTAAAAATAGGTTGAA

AGTCAAATGGTATAGTGCTACCTATTCTCAGAGTCAGCAGGCTCTTTTTGGAAAACAATG

GATTTCAGATATGAAGCGTTTAAAGTGTGATATCGAATTCTTCAGATGGTTTGAGATCAC

TGGAAAGTTAGATGACCAAAATGAGTCATTACAAGTCCTTGTTAATAAGTGGTATACTCA

GTCCAGCAAGGTCGTTGAGTCTGTCACTCCCCCGCTTGAGGGAATTAAAATTCCTCATTC

CAATATTCTTGTTCCTGCGTCTCCTTTCAAACTCAAAAGCAGTAATCCTCAGAGTTTGCC

CTCTAATGCAGATATTGATAAGATTGTCGAACAGAACAATTACACCAATAAATTGTTGCA

CACTGTTTCTTTACAGATTGAGGAAACAAATCCTAAGTTGGTTTTTTCAGCCCCTAGTTT

AAGTGCCCCTAGGCCCAGTACTAGTAGTACGATAGAGCCTAATCCTATATTCAAACCCCC

TGAATTTCGTCGTGAATTGTTCCCCAAGCTCAGCAACGATATTGCCGTCTCGGGTAAGCT

AGTTGAACAAATCACCGAAAAATTAGCCCACTTTCACGTCTCTGACAAAAGTAAGGCTAA

AGCATCGTCAGTTTCTGATTCTCCCTCAACCAAGGAAAATAAAGCTTCCTCAACCAAGGA

AAATGAAGCCTCCCTAACCAAGGAAAATAAAGCTTCTACTTCCGAGAGTGTCCAAACTCT

TTCTGATAACTCTCTTCATAAACTTTCTGACCGATATCCTAATCGGAGAAATTATTATAA

ACGTCCCTCTTTTCCTGATACTCAGTATGAGGAAGACGGTTTCCAGTCTCAATCATCTCA

TGATGGTCGAGGCATCGTTGAATGGAATATTGATGGATTAGCCGAACATCAAATCTATCG

AGTCCTTCACGAAATGGGTGTTTGCATTACCAGCTATAAGCTGCGTAATTCCTCCGATAA

AGAAGCAGCTGCTATGATCACAGCTGGCTTTACCGGAATGCTTAGGTACTGGTGGGATAA

TTATGTCACCGAAGAACTTAAGCATCACATCCTCAATGCAACCACGATCATTACCACTAT

TAAAACTAACAGTGGTATGGAAACTCAGGAATCTGAGACTCGTGAGGATGCTTGTGCCAC

TCTGCTCTACCACATTGCTAAACACTTTGTAGGTGAGCCAAAACTCTTCCAAGATAGGAG

TCTAGAAATCCTTAATAACCTTAGATGTCCTAAATTAAGTGACTTTAGGTGGTATAAGGA

TCAGTTTATGGTTAAGATTATGACCAGACCCGACTGTAATAGTGACTTCTGGAAAGACAG

ATTCATTAGCGGTCTTCCCCCTCTTTTTGCCGAAAAGGTTAGATCAAAAATAAGGGATAG

GTGCGGTGGTAAGGTGCCCTATGACCAGATGACTTATGGAGATATTATTAGTCTCATTAA

CACCACAGCTTTAGAGCTGTGTACTGACCTAAAGCTCAAGAGTCACCTTAAGAAAGATAG

GATGACATCTAGAGCCGAATTAGGCAGTTTTTGTCGTGATTTTGGGTATGGTAATATAGA

CCCTCCCCCTTCTAAGCTAGCCAAGAAGAAATCTAAGAAGTCTTCGTATAAATCTAAGAA

ACCTAGAAAGAATACTTCTAAGGATAGGCCATACCAACCCAAGAAGAAGTTCAGGAAGCA

TAGGCCTTCCCATAAAGAAGATGTATGTTGGAACTGTGGTAAGTCTGGACATAGAGCCAA

CCAGTGTAAGTCTTCTAAGAAGAAGAAGAAAATTAATCTCCTAGGATTAGATGACGATAC

TAAGGAAAAACTCTTTTCTATCCTCCAAGAAAGTGATTCTGCTTCTAATGATACATCTTC

GGACGAATATAGTGATGAAGAAAATATCAACGTCGCATATGAGACTGATGAAAGTCAGTC

CGAACAAGATGATTCCTGCGAAGAATCGTGCGAAGGTATTTTCTGTGATTGTAATTCTAA

TACCATTAGTACAATTACTGAAGACTCTAAAGAGGTTCTCTTTGATATTATTGACCATAT

CAAGGATGATGAGTCTAGAACAAAATACCTTGCTGAACTTAAGAAGATTATTCTTGGTTC

AGATCAAAAGAAACCTTCTCATTCTAAGGTCATAGAACCTTTTAGTATGAAACAAGTTTT

CTCTAGGTTCGATAAATCTGCCGAGCCCTCTGTTTCTGACTTACGGGTAGAAATGAATTC

CCTTAAGCAGGAAGTCAGAAATATGAAATCTCGTCTTGATAAGATTGAGATTGAGGCCCT

TACTTCTCAAGTTCTTAGAGAAGTTCCTGCAACAGATAAAGGAAAATCTGTTTCTATTCT

TGAACCCACCTCTAACTTTCCTGGAGAATTAATTGATCCCGGCCTCCTTGATCCCGGTCT

TCAAAAAGAAGCCAGTCCTGTCGGTCCTTCGGTATCTACCATTAAATCTATCAGTCAGCA

TATCCCTATTAAATTAGTGATTGCTAAAGACTTCGTGCTTAACAAAATCGCATTATTTGA

TAGTGGAGCCGATCGCAACTGTATTGTCAAAGGCCTCATTCCTCTCAAGTACCTTGAAAA

GGGTACTACTCGCTTATATTCTGCAACTGGAGAGCGGATGAAAATAGATTACAAATTGTC

TAAAGCGCATATTTGTAATAATAACATTTGCTTTACAAATGATTTTGTTATTACTGAAAA

TATTAATGAAGATATCATTCTTGGTTTGCCTTTCATATATCAGTTAAAACCCTATACTGA

TGATTTAGATGGCATTCATGCTTGTATCCTTGGTCAAAAGGTATTTTTCCCATTTGTTAA

ATCTCTTTCTCCTGAAGAAAGTTTGTTTGTAAAAAATAAAACTGTTTTTAAAATTAATCG

CCTCTCTAATCATATTTCTTTTCTTAAAGAAGAAATAAAGATTGAAAAAATTAAACAAGC

TTTAAAAACCCCCGGAATGGTGGCTAAAATCGATTCTCTTCAACAAAAATTTGAAAGAGA

AGTTTGTTCAAATCTGCCTACTGCTTTTTGGAATATTAAAAGGCATTCTGTTGCACTCCC

TTACATTGATGATTTTGATGAAAGGTCCATCCCTACAAAGGCAAGGCCTATTCAAATGAA

CCAAGAAATGTTGGAGACTTGCAAAACTGAGATTAACCATTTACTAGAAAATGGTATAAT

CCGTCCCTCTAACTCTCCATGGAGTTGTTCTGCCTTTTATGTAAATAAAAGTGCAGAAAA

GGAGAGGGGTGCTCCAAGGCTTGTAATAAATTATAAGCCTCTAAACTCTGTCCTAAAGTG

GATCCGTCATCCAATCCCTAACAAAAGGGATCTTCTTAAACGGACTTACAAAGCAAACCT

TTATAGTAAGTTCGATATGAAATCGGGTTTCTGGCAAATCCAGATTAAAAATGAAGATAG

GTATAAGACAGCATTCAATGTCCCTTTCGGACATTATGAATGGAATGTCATGCCATTTGG

ATTGAAAAATGCTCCTTCTGAGTTTCAGAACATTATGAATAATATTTTCAATCCTTACTC

TTATATGTCTATAGTTTATATTGATGACGTATTGATTTTCTCTGAGGACATAGATTCTCA

TTTTAAGCATCTTAATACATTTTTTAATGTGGTTAAGCATAATGGTCTGGTAGTCAGTGC

TAAAAAGATTAAGCTTTTCCAAACTACCATTAGATTTTTAGGCCATGACTTATATCAGGG

TAGTTATAAACCTATATGTCGAGCCATAGAGTTCTCCGACAAGTTTCCTAATGAAATTAA

AGATAAAACCCAGCTTCAACGGTTTCTAGGAAGCCTTAATTACGTGGCTGATTTTATCCC

TAATATTAGACAGGTCTGTGAACCTCTTTATAAACGACTTAGGAAGAATCCCTCACCTTG

GAGTGATGAGCAAACTGAAGCTGTTATAAAAATTAAAAAGATAGTTAAAAATCTTCCTTG

TCTTGGTATCCCTAACCCTGAAGCTTTCATGATTGTTGAAACTGATGCTTCTGACCTTGG

TTATGGTGGTATTCTTAAGCAATGTATTAGTCCTGAAAAAGGTGAACAATTAGTTCGATT

TACTTCTGGAGTCTGGAACTCTACTCAAAAAAATTATAGCACTGTTAAAAAAGAAGTTTT

ATCTATAGTGCTATGTATCACAAAGTTTCAAGATGATTTGGTTAACAAACCTTTTTTACT

TCGTGTAGATTGCAAATCTGCGAAAGAAATTTTATTTAAAGACGTTAAGAATTTGGTTTC

AAAACAAATATTTGCTAGATGGCAAGCTCTACTATCTAGCTTTGATTTTGAAATAGAGTA

TATTAAAGGAGAAAATAACTCCCTTCCTGATTTCTTAACCCGTGAATTCCTGCAGGGTCA

TGAAGCCCTCAAGACCTAATTCACCTTTCAAAAATGTTCGTCCTGAAAGGCAGGCCAATA

CGGTCAGACCTTTCGTTCCTCTTAACTTGACCCAATTGAGGCCTTCCTTTCCTCCTCTGC

CTAGTCCGGCCCAAAGCCCAAATAAATTTACAGTATTAGGAAATCTCCCAAAAAATACTA

GTCCAACTCCAGGCCTCAAAACCTGAAGCCTTACCAGGCCCACAACCTGTAGCATCTTCT

GGATCCACCTCTAAGGTCCAAACCAAAACCTCCTATGAAATGAAAATTCCTGAGTCCTTT

GCCCAAGCGGTTGACCCGAAAGTCCAACACAAAGAGCAAACTGGTTCATCTTCGACCCCA

GAGTCTTTTGAGTATCTAACTTCCAAGGTATTTCCTATCCTCGCCTTGGACAAAGAATAC

GAGTCCGGTAGGGTTCAAGATCTAATCAAACCCTGCTACCTTAATTCGAACTATGTAGAT

AGTTCAGACCCCCTGCATACCCGTCTGTACTATGAGGCCATTCTAGTAGATACAGATTCG

ATTGAAATTGAGCATGTATATTCTGAGAAGAACCCAGATTTCATCCTCTAATCCCAATTC

ACCATTAGGAGAATTCTTTCCCCGTTTGAATGGCAAGTAGACCATCTTCTTACTCCCATA

TCTTTGAGTATGCGCCATAAACCTCAAACTTTTAACTGGTATACCTACAAACAAGCCTGG

AATAATTTCATGTATCTCAAACCTACTCACACCTGGTTTGTTAAGTACAGTTCAGATTTG

GCTAAAGCCACTATTCCTAGGTGGTTTTACGATTGGTGGAAGATCTTCGGAGGAAAACTA

GAAAACCTCCCTCAGAGATTTTTACATAAGTTTGATGAATTCCAGACTCAGCAAGAGATC

TCAACTTTGCCTATGCATATTAAAATGTGTAAATATTATGTTCAAAAGCGAGTTTCTTAT

ATTATCAGTTGGGACTTTAAAGTCGCTGAATTTGAGCGGATAAAGTATTTGTCCAAAGAG

ATTATGGTCAAAGGATGGACGCCAAAGCCAAAGGCCCCTACTCCAAGCAAAGGAAAAGCT

CCTGTTCAATCCTCGTCTTCAGCTCACTCGAAAGCCGAACTCAAGAAAAAACTTCAACAA

GCTATTCTTGACCTCGACAAACAAGATGAAGCAGCTTTGCTACAGCTCATTGAAGCAGCT

GGATCATCAGGAGATTCCTCTAATGGAGATATGTGTGATCCACAGGGTATAGCGCAGGCC

TACCTAGAAGCCTGTTATGAATAGGATATCTTATCTCTGTGGGAAAATAGAGTTTCAAAG

AAACGGAATTATTTTCCCCAAGGACGAGAATACTATTCTAAAAGGAATCTGGTTTGGGTA

CAGCTGTAATTACCCGTGAATGGTGGGGATGCCCACTTGATGTTATGTAAGTGCTGTCAA

TAATTGTACACAGCACGCGACCTTTCTTTTCAAAAAGCAAGCCCCACTAATTAATAGTGT

CGGTGGACTTTTCTAGTTATCTAAGGACCCACATCTTTTGACAAAATTGTCTAGAGGTCC

CCACCTCCCATTTAATATTGTCGGCCGATTATGAGCCGTCCACTTGTAGGAAAGAAGTTT

CTTTTCTTTATCTTTTGAGGTCTATAAAAAGACACTCAATTTTACTGAGAAGGCATCGGA

AAATTTTAGAAAAATTCTTAAGAACTCTCCCTTAGCCTTCTATTCATCCCAGCCTTGTAC

TTCCAGTCTTACTGTAAACTTTCTCCTGTTTATCTTGGAATAAAGATAATGTTTTAAGTA

AGTTTTCATCTTTGATATTTTGTACTCTGATTTAGATATTGTATTAATTTTATCATATTT

CATTTTTATCTTGGTCCTGTTCCCCTT

>NbenV_sc2 [endogenous-virus-name=Nicotiana benthamiana virus] [strain=sequence cluster 2] [host= Nicotiana benthamiana] [moltype=genomic DNA] [complete genome] 7727 bp

TGGTATCAGAGCCATGATGGCTGTTTATGTGTTTAACATGAGCAACTAAACACATATACT

ATCCGGAGCTCAATGCTTTTGCAAAGACAAATCCATGACTAAGTTATTATGCTGAATACT

TGAAGTAATATGCTTAAATTATTTATAAGTGTTAAATCTTTTATCTTGTTTTATATTTGA

TCTACTTTGTGTGAAGTCTTATTTCTCTCTAAGTGTGTGAGGTATCCGAGTAATCCCCGG

AGCATACGTTGGCAAGCCTGTCCCTGTGGTCCTTGTAAGCCGAGTTAGGCGTTTTGTTGA

GACCTTTAGGTGTTCCCGTAGCCAAGATAACTCTAGAGAGAGAAGAGGTAAAGCCAAGAG

AACATGTAGATCCATATGAACCTTTGATAATTAGATCCAAAACTTATTAATAATTTGGCT

TTTATAATTCTTGTATTTGCATAATCTGATTATATTGTTTGGATACGTTGCTTTGCATTA

ATATTGATTCCAGGACAGTAATTTTTGAAATGAATCAAAATATTTTGAATACTGTTAACT

TAACATATCGTGCATCTCAGATTGCATTAACTGAATCTAATTTGGATAACTAGAGTATCC

CTGAAGAACCTTTTCATAAGATCTACCAAATAGGTCATTTTAATTTTATTAAGAAACATA

ATATTAAAACATGTGAATCAACTGTTGCTATTAATAACTCTTTAGAAGTTATAAAATTGT

TAAATGAGCAAGATATAATTAGATATAAATCTAATTTTAATTACTTGCATATTGGCCTTG

TTCAAGTTGCTGTTAAGCCTTTGTTTAGAAAAGGACTTAATATTCCTGTTTGTGCACTGT

TACGTGATGCACGTTTTCTTAATTTTGATGATTCTTTATTGGGAGCTCTTGAAAGTAATT

TAGCAGGTGGCCCTGTTTATTTCAATTGCTATCCTAATTTCTCTGTTGATATTAATGATC

CTAATGTGTTGGATACATTAACTCTTAATATTAAAACTATAAATATGAACAGCAAATCCG

AAACTCGTGAGTTAGCTGTTATATATCGAGTCTATTACAGGTTGATGGCAACTACCCTTG

CTCCTAGAGCTAGGATAGAGAGTACCAAAGGTGTGACTATGTTAATGGAAGCTAACCATG

AACATAGTACCGTCTTTGTTCCTCGCCTTCTCAAGTGGAATGACCTCCTGGCCAATACAG

ATTGGCATTTCGAGGCTATTACCCAACCTGTTTCCTCTCACACTGAGAAACGTCAGATTG

AGCGTATCATTCAAAATTCTGATGGTTCTGTTGATCTTAAATTTCTGGACTCCAGACCTC

GCAGAAGTTTCACCGCTGGTTCTACTTCTTCAAAACCCCCGGAGGGAGTATTTAATCGTT

CTTATTCTTTTAGGTCAGCTGACAAAAGTCAAGTTGATGGTGTAGATTTTTCGGGAGAAA

TCCCAAAAGTGTTTTATAAACCTCAGGTCCAGGAACCTTCTAGTCCCACAGCGTCTGATA

TGGAGCCTCCAAGGCAATATTCAGACCCTGGATTGGGTGTTATTACTACAGATGAGGATT

ATACCCCTGATCTTGCTTCTTTGAAAAAACTCTGGCTTGACCCTAAAAATAGGTTGAAAG

TCAAATGGTATGGTGCTACCTATTCTCAGAGTCAGCAGGCTCTTTTTGGAAAACAGTGGA

TTTCAGACATGAAGCGTTTAAAGTGTGATATCGAATTCTTCAGATGGTTTGAGATCACTG

GAAAGTTAGATGACCAAAATGAGTCATTACAAGTCCTTGTTAATAAGTGGTATACTCAGT

CCAGCAAGGTCGTTGAGTCTGTCACTCCCCCGCTTGAGGGAATTAAAATTCCTCATTCCA

ATATTCTTGTTCCTGCGTCTCCTTTCAAACTCAAAAGCAGTAATCCTCAGAGTTTGCCCT

CTAATGCAGATATTGATAAGATTGTCGAACAGAACAATTATACCAATAAATTGTTGCACA

CTGTTTCTTTACAGATTGAGGAAACAAATCCTAAGTTGGTTTTTCCGGCCCCTAGTTTAA

GTGCCCCTAGGCCCAGTACTAGTAGTACGATAGAGCCTAATCCTATATTCAAACCCCCTG

AATTCCGTCGTGAATTGTTCCCCAAGCTCAGCAATGATATTGCCGTCTCGGGTAAGCTAG

TTGAACAAATCACCGAAAAATTAGCCCACTTTCACGTCTCTGACAAAAGTAAGGCTAAAG

CATCGTCAGGTTCTGATTCTCCCTCAACCAAGGAAAATAAAGCCTCTACTTCCGAGAGTG

TCCACACTCCTTCTGGTAACTCTCTTCATAAACTTTCTGACCGATATCCTAATCGGAGAA

ATTATTATAAACGTCCCTCTTTTCCTGATACTCAGTATGAGGAAGACGGTTTCCAGTCTC

AATCTTCTCACGATGGTCGAGGCATCGTAGAATGGAATATTGATGGGTTAGCCGAACATC

AAATCTATCGAGTCCTCCACGAAATGGGTGTTTGCATTACCAGCTATAAGCTGCGTAGTT

CCTCCGATAAAGAAGCAGCTGCTATGATCACAGCTGGCTTTACCGGAATGCTTAGGTACT

GGTGGGATAATTATGTCACCGAAGAACTTAAGCAACACATTCTCAATGCAACCACGATCA

TCACCACTATTAAAACTAACAGTGGTATGGAAACTCAGGAATCTGAGATTCGTGAGGATG

CTTGTGCCACTCTGCTCTACCACATTGCTAAACACTTTGTAGGTGAGCCAAAACTCTTCC

AAGATAGGAGTCTAGAAATCCTTAATAACCTTAGATGTCCTAAATTAAGTGACTTTAGGT

GGTATAAGGATCAGTTTATGGTTAAGATTATGACCAGACCCGACTGTAATAGTGACTTCT

GGAAAGACAGATTCATTAGCGGTCTTCCCCCTCTTTTTGCCGAAAAGGTTAGATAAAAAA

TCAGAGATAGGTGCGGTGGTAAGGTGCCCTATGACCAGATGACTTATGGAGACATTATTA

GCCTCATTAATACCACAGCTTTAGAACTGTGTACTGACCTTAAGCTCAAGAGTCACCTTA

AGAAAGATAGGATGACCTCTAGAGCCGAATTAGGCAGTTTTTGCCGTGATTTTGGGTTCG

GTAATGTAGATCCTCCCCCTTCTAAGATGGCCAAGAAGAAATCTAAGAAGTCTTCGTATA

AATCTAGGAAACCTAGAAAGAATACTTCTAAGGACAGGTCAGACCAACCCAAGAAGAAAT

TCAGGAAGCATAGACCTTCCCATAAAGAAGATGTGTGTTGGAACTGTGGTAAGTCTGGAC

ATAGAGCCAACCAGTGTAAGTCTTCTAAGAAGAAGAAGAAAATTAATCTCCTAGGATTAG

ATGATGATACTAAAGAAAAACTCTTTTCTATCCTCCAAGAGAGTGATTCTGCTTCTGATG

ATACCTCTTCGGACGAATATAGTGACGAAGAGAATATCAACGTCGCATATGAGACTGATG

AGAGTCAATCAGAACAGGATGACTCTTGTGAAGGATCGTGCGAAGGTATTTTCTGTGATT

GTAATTCAAATACCATTAGTACGATTACTGAGGATTCTAAAGAAGTTCTCTTTGATATCA

TTGACCACATCAAGGATGATGAGTCTAGAACCAGGTACCTTGCTGAACTTAAGAAGATTA

TCCTTAATTCAGACCAAAAGAAACCTTCTCCTTCTAAGGTCATAGAACCTTTCAGTATGA

ACCAGGTTTTCTCTAGGTTCAATAAACCTGCTGAGCCCTCTGTTTCTGACTTGCGTAGAG

AAATGAATTCCCTTAAGCAAGAGGTCAGTGATATGAAGTCTCGTCTTGATAAGATTGAGC

TTGAGGCCCTTACTTCTCAAGTCCTTAGAGAAGTTCCTGCAACAGATAAAGAAAAATCTG

TTTCTTTTGCTGAACTAGAACCTACTTCTAACTTCCCTGGAGAATCAATTGATCCCGGTC

TTCTGAAGGAAGCCAGTCCTGCCAGTCCTTCTGTGTCCACCATTAAATCTCTCAGTCAGC

ATATCCCTATTAAATTAGTAATTGCTAAAGACTTTGTGCTTAACAAGATTGCATTATTTG

ATAGTGGAGCTGATCGCAATTGTATTGTGAAAGGCCTTATTCCTCTTAAGTACCTTGAGA

AAGGTACTACTCGTTTGTATTCCGCAACTGGAGAGCGGATGAAAATAGATTATAAATTGT

CTAAAGCGCATATTTGTAATAATAATATTTGCTTTACGAACGATTTTGTGATTACTGAAA

ATATTAATGAAGATATTATTCTTGGTCTGCCTTTCATAAATCAGTTAAAACCATATACTG

ATGATTTAGATGGCATTCATGCATGTATCCTTGGTCAAAAGGTGTTTTTCCCATTTGTTA

AATCTCTTTCTCCTGAAGAAAGTTTGTTTTTAAAAAATAAAACTGTTTTTAAAATTAATC

GCCTTTCTAATCATATTTCTTTTCTCAAAGAAGAAATAAAGATTGAACAAATTAAACAAA

CTTTAAAAACCCCGGGAATGGTGGCTAAGATCGATTCCCTCCAAAGAAAATTTGAAAATG

AAGTTTGTTCTAATTTGCCTTCTGCTTTTTGGAATGTAAAAAAGCATTCTGTTGCACTCC

CGTACATTGATGGTTTTGATGAACGGAATATCCCTACAAAGGCTAGACCGATTCAGATGA

ATCAAGAGATGTTGGAGGTTTGCAAAAATGAGATTAATCATTTGCTTGATAATGGTATAA

TCCGCCCCTCTAACTCTCCTTGGAGTTGCTCTGCATTCTATGTTAACAATAGTGCAGAGA

AAGAAAGAGGAGCACCCAGGCTTGTAATTAATTATAAGCCCCTGAACTCTGTCCTCAAAT

GGATCCGCCATCCAATCCCCAACAAAAGAGATCTTCTTAAACGAACTTATAAAGCAAACC

TTTATAGTAAGTTCGATATGAAGTCTGGATTCTGGCAAATCCAAATTAAGGATGAAGATC

GGTATAAGACTGCATTCAATGTTCCTTTTGGGCATTACGAATGGAATGTTATGCCATTTG

GGTTAAAAAATGCGCCTTCTGAATTTCAAAATATTATGAATAGCATTTTTAATCCTTATT

CTTATATGTCAATAGTTTATATTGATGATGTGTTGATTTTTTCTGAGGACATTGATTCTC

ATTTTAAGCATCTCAATACTTTCTTTAATGTTGTGAAAAACAATGGCTTGGTTGTTAGTG

CTAAAAAAATTAAGCTCTTTCAAACCTCGATTAGATTCTTAGGTCATGATCTTTATCAAG

GAACCCATAGGCCTATTTGTAGAGCCATAGAATTCTCTTCTAAATTCCCTGATGAAATTA

AGGATAAAACTCAGCTTCAGAGATTTTTAGGAAGTCTTAATTATGTTGCTGATTTCATCC

CAAAAATTAGGCAAGTCTGTGAGCCATTATACCATAGGCTTAGAAAAAACCCAGCACCTT

GGGGAGAAGAACAGACCCAGAGTGTCCAAAAAATTAAGAGATTAGTTCAAAATCTTCCTT

GTCTAGGGATACCTAATCCTGATGCATTTATGATAGTAGAAACAGATGCTTCTGATATAG

GATATGGAGGCATCCTTAAACAGCGCGTCAACCCAGATAGTTGTGAGCAACTTGTGAGGT

TTACTTCTGGTATTTGGAATTCTACCCAGAAAAATTATAGTACAGTCAAAAAGGAAGTTT

TGTCTATAGTACTATGTATTACTAAATTTCAAGATGATTTAGTAAACAAAGAATTTTTAC

TCCGAGTTGATTGCAAATCTGCAAAGGAAATTTTACAAAAAGATGTAAAAAATCTTGTTT

CAAAACAAATATTTGCTAGATGGCAAGCTCTACTATCTAGCTTTGATTTTAAAATTGAGT

TCATTAAAGGAGAAAGTAATTCTCTTCCTGATTTTCTAACCCGTGAATTTCTTCAGGGTC

ATGAGGCCAGCCAAGACTAACTCCCCGTTCAAAAATGTTAAACCCGAAAGGCAGCTTTCG

TCCGCTAGACCTTTCGTCCCTACAGGCCTAGCCCAGCTAAGGCCTTCTTTCCCGCCTTTA

CCTAGTCCGGCCCAAAGCCCATCTCAAAACAAATTTACTGTCCTTGGAGACATCCCCAAA

CTTACAGGCCCAAAACCTGAATCCTCGTCTGGATCTACTTCCAAGGTCCAAAGTAAACAG

AGCTACGAGATGAAGACCCCTGAGTCCTTCGCCCAGGCTGTGGACCCAAAGGTCCAGACC

CAAAGTCCTTCCTCTCAGGCACAAGAGCCTTTCGAGTACCTAACCACTAGAGTTTTCCCT

ATTTTGGCCCTCGATAAAGAATTCGAAACTGGTAGGGTCCAAGATTTAGTAAAACCTTGT

TACAACAATTCAAACTACGTAGATAGTCCTGATCCTTTACACACTCGCCTTTATTTTGAG

GCAATTCTAGTTGATACCGATTCTATAGAAATCGAACATGTTTATTCTGAAAGAAATTCA

GATTTTATCCTATATTCCCAATTCACCATTAAGAAGATCCTCTCCCCGTTTGAATGGCAT

GTGGACCATCTACTCACTCCTGTACCTCTGAGCATGAGTCACAAGCCTCAGACATATAAC

TGGTACATCTACAAGCAAGCATGGTACAATTTCATGTATCTCAGGCCGAGTCACACTTGG

TTTGTTAAATATAGCCCTGAGCTAGTAAAGGCCACTATTCCTAGGTGGTTTTACGATTGG

TGGAAAGCTTTCGGAGGAAAATTGGAAAACCTCCCTCAACATTTTCTGCAAAAGTTTGAC

GATTTCCAAACCAAGCAGGAGATCACTACGTTGCCTACTCATATTAAAATGTGTAAATAT

TATGTCCAAAAAAGGATATCGTATATTATCAGCTGGGACTTTAAGATCGCAGAATTTGAC

CGTATCAGGTATCTATCCAAAGAGATAAAGGTTAAAGGTTGGGTTCCAAAACCCAAAACT

CCTACTCCAAAGAAGGAGAAAGCTACTGCTCAGTCCTCTTCGTCAAAGTCATCCAAAGCC

GAGATCAAGAAGAAGCTTCAACAAGCCCTTCAAGATATCGAACATCAGGATGAAGCCACT

TTGCTGCAGCTCATTGAAGAAGCCGCCTCCTCAACCGAATCAAGTAATGATGATATGTGT

TGTCCTAAAGGCATAGCACAGGCTTACCTGGATACCTATTACGAATAGGAATTCTGGCCC

TTGAAGAAAAGTAGAGGTTCAAAGAACTGGAATTACTTTTCTCCAAAGGACGTTTTACTA

TTCCTAAAAGGAATCCGAGTAGGGCCAAGCACATGGCCTTGTCTTAAATTAATTATAGTG

GGAATGCCCACTTTTGTCTTGTATACGCTTACTTTATTAAAGAAAAGATAAGCATGAGTA

ATAAGTCAAAAGTATGCCTTCCACTCAGATGGAATCTTTGCTGTACGAGTCTAGTCTTAA

TCAGTAGGGACCCACTACTTTGTCGGCAAAATAATAGCCGTCCACCTGTAAGCACAAGAT

GCTTTTACTTTATCTGAGGTCTATAAAAGACTCTCACTTGTATTGTTGGAGGGCAGTCGC

AATTTTAGTCTCTCTTAAAATTCTCTTTACCTCTGAATCTTGTAATCTTCCAGCCTTGCC

TTCTCCTTTTAAATAAAAAGCTGGTTTTGTAAGTAACATCTTTTCATTCGTTCATATTTT

ATAAATGACTCACACTTCTTAGTGTTTAATTTCCGGTCCTGTTCCCT

>OsatBV_compAsc1 [endogenous-virus-name=Oryza sativa B virus] [strain=sequence cluster 1] [segment=component A] [host=Oryza sativa japonica cv. Nipponbare] [moltype=genomic DNA] [note=complete component sequence] 6528 bp

TGGTATCAGAGCCAAGTGCTCGTAACCTCCCCAAGCCATCTTCTCCGAAAGGGGAGGGGG

GGGAAGAGGGGTCGGGTGGCGTGCTGAAGTCGCGGTGATCCCGAACGGTGTGGTTGTGTT

CTGAAGTCGCGGAGTTCCCGAACCTTTGATCGGTTGCCCGAAGTCGCGTTGCCTTGTTGA

GCCGCAGAAGGCGTTTGTCGGCATAATTCGTTGACTGGGCACTTATCAAAGGCAAAGGTT

GAACTTAATTGTGGGAAATATTCCCTGCCGTTGACTGGAATACATCATATCCAAATAGGT

TGATCATAATTGTGGGAGATTTCCCCGCCGTTGACTGGTAACTACCTACCTATCTTCCTA

TCTTTCGTATAAGATGTCTTGGTTGGTTAGATCACGAAGAGCCACTGATCAATCTGTAGC

CTCGTGCAGTAGCGAGAAGTCTGTTGGCACTATGTCGATAAAGAATGCTTCATCTATATC

TTTTGAGGATATTGAGAAAAGTATTTCCAACTGGAAGATCCCTAAGGTTAATATTAAGGA

AATATATCATGTAGGTACTTTTTCGTTACTTAGTAACTACTATATTAAAACAATCGAAAA

AACTATTCCAATTAGTTCTTTGCATGAAACCCTGCATCTTCTTTCAGAAAGAGAAATCAA

TTCTATAAGAGCTAAACATCAATATCACTACCTTCACTTTGGTCTTATACAAGTTGCTAT

CAGATCCTTGACTAGAAAGGGTTTGAACGTCTCTGTTCTGGCTTGTCTAAGAGATTGCAG

AAATAAAAGGTTCAAAGATTCCTTACTTGGCATGGTGGAAGCCAGCCTCAGTAATGGTCC

TATTTATTTTAATACCTTTCCCGATTTTTCTGTATCCCTCTCAGACAAAAATATCCATAA

AGTTCTCACTCTCAACTTACAAACATCTAGTTATGAACTAGAACCAGGGAGTGAAAACAT

CTCCGTTACCTACAGAGTTTACTACAAAGCCATGACAACTTTAGCACCTTGTGCCAAACA

TTATACACCAAAAGGCCTAACCACTCTCCTTCAAACCAATCCTAATAACAGACATACTAC

CCCCAAAACCCTGAAATGGGATGAGATCACCCTGCCTGAGAAATGGGTTTTATCTCAAGC

TGTTGAACCTAAGTCTATGGATCAATCAGAAGTCGAATCCTTGATTGAAACACCAGACGG

AGATGTTGAGATAACATTTGCTTCAAAACAAAAAGCCTTTCTCCAGTCTAGACCTTCTGT

CAGTCTAGATTCTAGGCCTAGGACAAAACCTCAAAATGTTGTCTATGCTACCTATGAAGA

CAACTCCGACGAACCTTCAATCTCAGACTTCGATATCAATGTTATCGAGCTTGATGTTGG

CTTTGTTATTGCTATAGAAGAAGAAGAATTTGAAATTGACAAAGAATTGCTTAGGAGAGA

AATAAGACTCCCCAAAAATAGAACTAAAACTAAAAGATATCTTGAAGAGGTTGACAAATC

ATTCAGAATGAAGATAAGAGAAGTTTGGCATAACGAAATGAGAGAACAGAGGAGGAATAT

TTTCTTCTTTGACTGGTATGAGAATAGCCAGATTATATATTTTGAAGAATTTTTTAAGAC

CCAGAAGAAAGGAAAAGGGGTATGTAATGAAGAGAGAAAACAAGAACATGACAAGTCAGT

AATTAAGGTCTTTTCAGATTATGGGTCAACCAGTAAAACGTATATTACTTATTCTCCTTT

TAAACTTACTCAACAATCTCAGGATATCAGAGGAAAAAATTGTTTTCCAGAAAGCACTTA

TGAGAAATCCATAGAGAATAATTTCCTAGAATATTTAGTAGAACAACAGAAAACTCAAGA

TCTATCCCCTGAACAACAAGATTCCAAAACAATTCCTATGGAACCCATTATTCTAAGAAG

CCATGAAGAACCTTCTTCCCACTCTCAATTTGAGGTATCTAGACCTCAATCTGAAACCTA

TCTTTTGCCTTATTCTTATCCTATTACAACTATACTTATTCCAACTGCCACTCCTAAAGA

TAAGGTAGCCAAATTCTTTGATATTCCCAACAATTTTCCTAATACTAATGACCAGATGTC

TAATTCCTTGACAAAAATCCAAGCGTCATCTTACGATAAATTGACGCTCCAAAAAGAAGT

TTCTTCCTTTTATAACAAAATCGACTCTTCGGTAAACAGAAACCTCGAGAGTTATATTAC

CAGAATCATCAATACCTCTTTCTACAACATAATCAATGATCCTAAAGGTATTACCAGATC

AAAATTTAGATTGTTCCATAATGTTCTCTTCTCCAAGATCTATATCCAACCCAATCCAAA

TAAAACACTTTGTTACCATTCTCAAACCAAAAACTCCTTTGAAAGAAAATCTCAAAACCA

AATCTCGGCTGAAGCCTTATGCACAAATAATGAAATCCTAGTCCATAAGCAAGATCTTTT

TGGAAGCATATTTGTTATTTCTGATACAAATCAATTTGGCACTTTTAATCCTGAGGAAGA

AAGGACCAAAAGAAAACTTAAGATTAATGACCTCTTCCAAGAACAAAATTATACTAAAAA

GGAATTAAGAGACTTAAAGGGTAAAATTCGAAGTCTAGAATTACAAAATAATAGTTGCAT

GGGCCAAGATGAAGAGGTTGTCAACTCCATCAACAGTTATGTTAAGCAGAAATGGTATGC

AGAAGTAAGGTACAAATTCAATGATGGATTCCAATTCAGCTATAAGACTTTAATTGATTC

AGGAGCTGATGTGAACTGCATTCGTGAAGGTATTATCCCATTCAAATACTTTGAAAAATC

CTCCCATAAAATTCATGCTGCAGATGGAAATTTACTAAAAGTCAATTACAAAATCCCTTC

TATTCATGTTTGCATAAATGGAGTCTGCATTAAAACTTCCTTTTTGCTTATAAAAAATCT

AAAGCAAGGAGTTATTTTGGGAACGCCATTCTTATCCCTAATAAAACCCTTTATTGTTAC

TGAAAAAGGTATCCAGTTTAGAATCAGAGACAAAAATGTTAAGTTAAAATTTTCTTCTAA

ACCCGAGCAAACCATGTTAAACTATTTAAAGGAGATCATCACACAAAAAGAGCAATTTGT

GAGTGACATATCTTCTGAAATAAAAAATGCTAGGATTTCCCAAACCCTTAATGATCCAAG

GTTTCAAGAAAAAATTATACTGTTAGAAAAAAGGTTCATAAAAGAGATATGTTCTGATTT

CCCATCAGCTTTTTGGCATAGGAAAAAGCATGTTGTAGGTCTTCCTTATATCTCTAATTT

TAATGAAGATAAGATTCCCACTAAAGCAAGGCCCATTCAGATGAATAGTCGACTTTTAGA

AATTTGCAAGCAAGAAATAAAAAACCTGCTAGACAAAGGCTTAATAAGGAAAAGTTCCTC

TCCTTGGAGTTGTGCAGCCTTCTATGTAGAAAATGCTGCTGAAAAAGAACAAGGAGTTCC

TCGGCTAGTTATCAATTACAAACCTCTCAATAAAGTTCTACAATGGATAAGGTATCCAAT

ACCTTATAAGCATGACTTAATACGAAGGATTCAGGGTTCAAAGATATATTCTAAATTTGA

TATGAAGTCTGGGTTTTGGCAAATCCAAATAAAAGAAGAGGATAGATATAAAACTGCCTT

TACTACTCCTTTTGGTCATTATGAGTGGAACGTAATGCCGTTTGGTTTAAAAAATGCTCC

TTCTGAGTTCCAAAAAATAATGAATGAGATTTTCCTTCCCTTTACATCCTTCATTATAGT

TTATATAGATGATGTTTTAATATTTTCCCAAGATGTTGACCAACATTGGAAACACCTTAA

TATATTTTATAAAATTATAGTTCAAAACGGTTTAGTAGTCTCTGCTAAGAAAATGAAACT

TTTCCAAACTAATGTTCAGTTTCTCGGTTATAAAATTCAATATGATCAAGTCCAACCAGT

AGCAAGAGTAATAGAATTTGCAGAAAAATTTCCGGATGAGATAAAAGATAAAACACAGTT

GCAAAGATTTCTAGGGTGTTTAAACTATGTCTCCGATTTTTATAAAGATCTTGCAAAAGA

CAGAAAGATTTTAACCGAGAGGTTAAAGAAAAAGCCTCCAGCTTGGACAGCTAAACATAC

TCAAGCTGTTAAGAAGATCAAAGGTAAGGTCAAAACCTTGCCCTGTCTTTATATTCTAGA

TCAAGATGCCTTCAAAATTATCGAATCAGATGCCTCAGATCATGGTTATGGAGGAATTCT

TAAACAAAAAAAGGATTCCAAAGAGCAGCTGGTCAGGTTCACCTCTGGGACCTGGAATGA

GGCTCAAAAAAATTATTCAACCATCAAAAAAGAAATATTGGCCATAGTAAAGGTCATATC

AAAATTCCAAGGAGAATTATTGAATCAAAGATTCTTGCTGCGTATAGATTGTAAGGCAGC

AAAAGATGTCCTCCAAAAGGATGTGGAGAACTTAGTCTCCAAACAAATCTTTGCCAGATG

GCAAGCAATATTATCATGCTTTGACTTTGAAATAGAATATATCAAGGGAGAATTAAACTC

CCTACCAGATTTCTTATCTCGTGAATTTTTACAGGGCTATGGCACCTAAAAGGGACTTAT

CAGCCCAAGGGAGAACTACAGAAAGTTCACCAAATAAGCATAAATATAATGAACTAGGAA

ATATCATTCCTAGAACCCCTACAGTTCAAGAAAAGTATGGGAATAATTCATCTTATATCC

TCAATATAGAGGAAGTAATTCTGCCATTAGAATTTGGAGACTCTGATCTCAATATTATCA

AGATTATGGGAAAGTATTTCCCACAACACCAATATTTTATCCCTGAATATCCAGGGAAAG

ATCAAAATTATTATGAGACCATATTATGCGAGACAAGAAGTGCACAGATATTCCATACAA

GGAATGGTGATGAATTGGGATTCACAAAATTATTGATCCAAAAAATTATAAGTATCGATG

ATTGGGATAAATCATCAAATCCTTATGTAGCCAGGACTATATATTCTACATCCTGTGCAA

ATAAAAGATATAACTATTGGGATTATCAGAAGGCCTGGGAAAGAGTACTCCTCGTACAAA

ATTCCCAGATGAAGCATTCATGGTTTATTCGCTTCAAAGAAGGATGTGAGGAGATACCAC

TGTGGTTTTTCAGTAATTGGTGGCTGAAAGCAGGGGCCATTCCTGAAATCCTACCACAAG

AAATCATCAAAGTTATTACACAAGAATCAAAAAAGGATCTCAAGGAATATCCTTTTATCC

TTATGCAATTTTGTGCCGAAACAGGGATGCCTTGGATACTAAAATGGGACCTCAATATTC

AAAGAATGGAGTTCCCAGCAACCTTAAAGCGAAACTATTATGCAAGATGGTGGGATAAGT

TTGCAATCACACCAGTAATTGAAGGAAGAAAGTTTCGAGCCAAAAACAAGAAATCTCATG

TGGCTCAATTAAAAGAAGATATAACAAGAGAATTATTAAAGGCAAGACCCGAGTTGACAA

AGGGAGAATTGCAACTTCAAGTATATGAAACTATGTTTAAAAGATTGGAAGAATCACCAA

AATCTAGTTCTACATGCAGAAGTCTAGACGAAGATATGGTACAATGCAGCCAAATTAAGC

CATCTTCGCCAATACCTCCTTATTACTCAATTAAGCAAGATAATGATTCAGATGAAGGAA

TATCAGATTTCAATCCAACACACATTTAGTCAAGATATGACGACAGATCTTGTCACCAAG

ATAATGAATAAAGATCTTGTCATCCCCGACAAAGATCACTATTCAAGATAAGGAATCTAC

ACTATTCCAGATAAGATAAGTCCATGGTCAAAAGGCGATATTATGACATCTTTATTGAAG

GTCAAAAGCGTCATCACCAAAGATTCTCTCCATGAAGTCTAAAGGGATATCATGAACTCT

TTGAAGTCAAGAAGAGATATCATGAAGATTCTCTACCGAGCGCTATATAAGGGGCTCACT

ATCACATTTCAGAGGCATCGCATTCCACACACAACACATCTCACTCAACCATTCCTCTCA

CCGCTCCACCAGCCATCACACTCCACTCCACTCCATCTAAGTGTTAGTACTATAGTATAA

CCTAAATAAAAGACTCATGAGAGTCTAAGTGTGAGAGTCTATGTAAGAGTCCTTGTGAGA

GTCCTGTACCTGTATAATTACGAACGTGTTGTACCAAGTCCTTGGTGTAATCTTCAGAAG

GATCTACCTTCAACCTATCAAGGTATATGAATGAACACCTGGACCGTTCTATATAAACCT

ACTATGTTCTAAATATCTAATTTTATATCTTGTACATAATTCGTATTTACAGCTTGTCTT

TATTTCCTGTACTGCATTTATGTTTGCATAATAATATAAAAAAAATTAACAACCTGGATT

GCCATCCAATTGTCACATACTAATCTCACATCCAAGATTGCATATAGATAAGCATTTCTA

TCCTCATATCTGGTGACTAGACCCCTAGTCACTGCCCTGGGTTAGAGC

>OsatBV_compBsc1 [endogenous-virus-name=Oryza sativa B virus] [strain=sequence cluster 1] [segment=component B] [host=Oryza sativa japonica cv. Nipponbare] [moltype=genomic DNA] [note=complete component sequence] 6651 bp

TGGTATCAGAGCCAAGTGCTCGTAACCTCCCCAAGCCATCTTCTCCGAAAGGGGGGGGGA

AGAGGGGTCGGGTAGCGTGCTGAAGTCGCGGTGATCCCGAACGGTGTGGTGGTGTTCTGA

AGTCGCGGAGTTCCCGAACCTTTGATCGGTTGCCTGAAGTCGCGTTGCCTTGTTGAGCCG

CAGAAGGCGTTTGTCGGCGTAATTCGTTGACTGGGCACTTATCAAAGGCAAAGGTTGAAC

TTAATTGTGGGAAATATTCCCTGCCGTTGACTGGAACACATCATATCCAAATAGGTTGAT

CATAATTGTGGGAGATTTCCCCGCCGTTGTCTGGTAACTACCTACCTATCTTCCTATCTT

TCGTATAAGATGTCTTGGTTGGTTAGATCACGAAGAGCCACTGATCGATCTGTAGCCTCG

TGCAGTAGCGAGAAGTCTGTTGGCACTATGTCGGTAAAGAATGCTTCATCTATATCTTTT

GAGGATATTGAGAAAAGTATTTCCAACTGGAAGATTCCTAAGGTTAATATTAAGGAAATA

TATCATGTAGGTACTTTTTCGTTACTTAGTGACTACTATATTAAAACAATTGAAAAAACT

ATTCCAATTAGTTCTTTGCATGAAACCCTGCATCTTCTTTCAGAAAGAGAAATCAATTCT

ATAAGAGCTAAACATCAATATCACTACCTTCACTTTGGTCTTATACAAGTTGCTATCAGA

TCCTTGACTAGAAAGGGTTTGAACGTCTCTGTTCTGGCTTGTCTAAGAGATTGCAGAAAT

AAAAGGTTCAAAGATTCCTTACTTGGCATGGTGGAAGCCAGCCTCAGTAATGGTCCTGTT

TATTTTAACACCTTTCCTGATTTTTCTGTATCCCTCTCAGACACAAATATCCATAAAGTT

CTCACTCTCAACTTACAAACATCTGGTTATGAACTAGAACCAGGGAGTGAAAACATCTCC

GTTACCTACAGAGTTTACTACAAAGCCATGACAACTTTAGCACCTTGTGCCAAACATTAT

ACACCAAAAGGCCTAACCACTCTCCTTCAAACCAATCCTAATAACAGATGTACTACCCCC

AAAACCCTGAAATGGGATGAGATCACCCTGCCTGAGAAATGGGTTTTATCTCAAGCTGTT

GAACCTAAGTCTATGGATCAATCAGAAGTCGAATCCTTGATTGAAACACCAGACGGAGAT

GTTGAGATAACATTTGCTTCAAAACAAAAAGCCTTTCTCCAGTCTAGACCTTCTGTCAGT

CTAGATTCTAGGCCTAGGACAAAACCTCAAAATGTTGTCTATGCTACCTATGAAGACAAC

TCCGACGAACCTTCAATCTCAGACTTCGATATCAATGTTATCGAGCTTGATGTTGGCTTT

GTTATTGCCATAGAAGAAGATGAGTTTGAAATTGATAAAGATTTGCTTAAAAAAGAACTC

AGGCTTCAAAAGAACCGACCCAAGATGAAAAGATACTTTGAAAGGGTCGATGAACCATTT

AGACTAAAGATAAGAGAACTTTGGCACAAAGAAATGAGAGAACAAAGGAAGAACATCTTT

TTCTTTGATTGGTATGAAAGTAGCCAAGTCAGACACTTCGAAGAATTCTTCAAAGGAAAA

AACATGATGAAAAAAGAGCAAAAATCCGAAGCAGAAGATTTAACTGTTATCAAGAAGGTT

TCTACAGAATGGGAGACAACCAGTGGAAACAAGGTAGACTCTGTTCATCCTCCATTTGAA

TCCATTCAATTATCTCATAATGGAGGAAAAGCTTGTCCACTAAAAAGTATTTCTAAAAAT

ACATATGGAGAAACAGCAAAGGTTGAACACATAGGACATTTGGTAGAACAACAAAATTAT

GCAAATATATCTCTACGTTCTCTTGGTCAACAAACAGATCGAATAGAGACAATCCTTATG

GAAGGCTACAAAACAGGAAGACCTGAGGTAAAGATAAATATTCCCTCTAATTCTCAGTCT

TCTAGTTCTCAGTCTGTTAGTCCTATGTTTGTCCCAACAATTGATCCTAACATCAAACTT

GGAAAACAAAAGGCTTTTGGACCCGCAATTTCTGAAGAACTAGTCAGTGAGCTAGCCTTA

AAACTAAATAATTTAAAAGTAAATAAAAATATCAATGAGATTTCTGATAATGAAAAATAT

GATATGGTCAACAAGATTTTTAAACCTTCTACTCTTACTTCCACAACTAGGAATTATTAC

CCTCGACCAACTTATGCAGACCTACAGTTTGAGGAAATGCCTCAGATTCAAAATATGACT

TACTACAATGGCAAAGAAATTGTAGAATGGAACTTAGATGGCTTTACTGAATATCAAATC

TTTACTTTATGTCATCAAATGATTATGTATGCTAATGCCTGCATAGCTAATGGAAACAAA

GAAAGAGAAGCTGCCAATATGATTGTCATTGGGTTTTCTGGTCAATTAAAAGGATGGTGG

AATAATTATTTAAATGAAACTCAAAGACAGGAGATTCTTTGCGCAGTTAAAAGGGATGAT

CAAGGAAGGCCATTGCCCGATAGAGATGGTAATGGGAATCCAACCGGTAACATTTCTGAT

GCCTTAGCCACTTTAATCTATAACATAATTTATCACTTTGCCGGAAACTATCATGATATC

TATGAAAAAAATAGAGAGCAATTGATAAATTTAAAATGTAAAACAATGTCAGATTTTAGA

TGGTATAAAGATACTTTCTTGTCAAAACTATATACCCTACCAGAACCTAATCAAGACTTT

TGGAAAGAAAAATATATTTCTGGTTTACCTCCTTTATTTGCTGAAAAGGTTAGAAATTCC

CTAAGAAAAGAAGGGGGAGGGAGTATAAATTATCACTATCTAGATATTGGAAAAATCACA

CAAAAGATACAATTAGTAGGGGCTGAGCTTTGCAATGACCTAAAAATCAAAGATCAACTC

AAAAAACAAAGGATTCTTGGTAAAAGAGAAATGGGAGATTTTTGCTATCAGTTTGGTTTT

CAAGATCCTTATGTCTATAGAAAAAGAAAAACCCATTCCAAACCTATGACAAAACCAAAT

GATAAAAGCAAGATGAGCTTTCAAGCTACCAAAAGAAAACCAAAAAGAATTTACAATAAG

AATATAAGAACACAAGACACTGAAAGCAAAGAAACCATTTGTTACAAATGTGGCTTGAAA

GGACACATAGCTAACAGATGTTTCAAATCCAAAGTTAAAAAAGAGATCCAAGCCTTGTTA

GATTCAGAATCAGAAGATGTTAAAGAAAAACTTGAAGCAATACTAAATAATATAGACAAT

GACTCAAGTTCAGATGAAGAAAAAAATGCAGAGATTAATTGTTGCCAAGACAGTGGTTGC

TCTTGTTATGAGCCAGATAATTCAGAAGAAGAGTCTGATGAAAATATACTCGTTTTAACC

AGTTTAGAAGAATTTGTTCTCGATACTTTTGAAACCATTCAAGATCCAGAAGAAAAAAGA

AGAGTTCTCGAAAAATTCCTTTCAAGAGTCAAGACTGATAAGGATAAACTAAAAAAGGAT

ATTCAAAAGAGCAAATTCCTCTCTATTGATGAAGTGTTCAAAAGATTAGATGAACAGAAA

AAGAAGAATGAGAAACCAGATTTGATCAGCCTATTCGAAGACCAAAAAATTATGAAACAA

GATTTAGAAGAAATAAGAAAGCGTCTATACATGTTAGAATTAAAAGAAGGATTCCATATG

GAAGAAAAGGACGAACCAATTCAAGAAGATGATCAGGTTGTTGGAACCATTCAAAAATAT

ATGAAGCAAAAATGGTACACAGAAGTTATGTATCGTTTTATAGACGGATCCTACTTTCAG

CATATCACTTTAATTGATTCTGGTGCTGATGTCAATTGCATTCGAGAAGGTATTATCCCT

CATAAATATTTCTGTAAAGCCGCCCACAGAATTCGTGGGGCAGATGGAGGACTTTTAACA

GTAGAATATCAAATCCCTGAAATCTATATTTGCATATCTGAAGTTTGCATTAAAACATCT

TTTCTACTCGTTAAAAATCTTAAGCAAGATGTTATTTTAGGAACACCGTTTTTATCACTC

ATTCGTCCCTTCCTTGTCACAAACGAAGATATTCAATTTGAGATAATGGGCAAACAGGTG

AGCCTAAGATTTTCGTCTAACACAGATGAGATTCTTGACCAGCTTGTTCAGACCAAAAGA

GAACAAGTTGTGAATACAATATATTTACATGACAATTCTTTCCCATCATATCTTCCAAAA

TCTATGGACTTGCCAGGTAAGACATCATGGGAAGATAGATATTTATTAAAAGCTACTAAA

AATTTTGAATATATATGCAGGGAAATGGCTTCACAGAAGTCTGAATGGCTCACGAATATG

AGTTCATGGAGACTGCCCCAACTGCCCAGAGGCCATGGAGAACGATTTAATCACAATTTC

AGAGTAACAGAGCTACAACAAGGGTTATTAAACCTCTTGTGGCAGACAAAAAACAAAAAA

GAAAAAGCTCATGCCTTAAATGGCCTGGCATATTATTTCAAAAATTTCGTGCCCTCAGAT

CAAAAGATAACAGAAAAAAGGGCTAAAATACAAGATATCCCTCATCATGAGGAGAGGTTA

CTAGATTATCGCGAGGAGAAATCCCGTGATGGCCAAGATAAACTTCCCATGGAAGTTGAA

CAATCTATGGCAACAGATAAGAATACCAAGGTATTCTCAGATAAGATTTTGATCTCAACA

GATCCTGCTTCGAGCATGGTTGCTTTCCACATAAATATCAATAACCTATTGCAATCTTTT

CCACATAATAAACCATCATCATCCACAAAAAGGCATGATACCATTCCCCAAACACCATAC

ATACTGCATAATCATCCCAATTCATTCCTTCCACAATATATACTCCGGACCCAGCCAAAA

GCACCATGTAGATCTTGTGCCTACAAAGATTCTTGCCAAAAGAAACTTAAAAGCCAAAAT

CCCATGAGTACTACATACGAAAATCTCCAACCTAGATTAAGCTTCAAGACGGAGCAAACT

TTATCCTGCTTTGGGCAAGACATCCTCGATCTTGTTTGGCAAAAGTTTAAGGAGAGACAA

ATGAAAATATTCCTTGGACTCCAAGATTATTTCTTTGATCTCTTAGAAGGAAGAGAAAGA

AACCCAGCCATACGAGTAATCATATATATGCTCCCTCTCTTAAGGCTAGATGATCAGGCA

ATCTCTGACCCATCCTATAGATTCCTGGTTCTCAAAGCTGAGATAAATCTCCAAAGATTC

CGGAACCTACCCACATACAACCATGAAGAAATATCTCTCCAAACCATTATAGATCATGGT

CTTGTGAATTCCATCTATGCAACCCTCGATCAGATCCTTCAATCTGATTTAGGAAGCGCT

GTGAAAGATGTCTGCAGGAGACTTGGACATGGAAGATACAGGATTATCTTCTCCTCCATA

CCACCAAAATTCACACCACCTGTTCGTCCAGCAATTCATTATATATATATCATGAATGGA

CAATTTAATTTTCAAGAAGACGGTCCTAGCAAACTATCGGATGAAGAGGAAGAAATATAT

ACAACAATTGCTAATCATGAAAATTGGAGATTATTTTCTGAAGCTGCAGAAATTGAAGAA

GCTATCACCACAGATTATGAATATCAGCTGGTTTTCCAAAACAAGATCACAAGGATTTTT

ATCAGCAAATTCTACAACCAAAGTTATGAATACTTCAAGGGAGCAGGGCGCATAATTAAA

CCGGATTTTGGAAATGAATGCTCCAAGAAAAGTTACTACAGAAGCCTGGCTCAGTGGTTT

CAGAAAACAGAGCCATCAGAGATTGACACGGTGCAATCAGCACCCATGATAATCAACGAA

GATAATGAATAAAGATCTTGTCATCCCCGACAAAGATCACTATTCAAGATAAGGAATCTA

CACTATTCCAGATAAGATAAGTCCATAGTCAAAAGGCGATATTATGACATCTTTATTAAA

GGTCTAAAGCGTCATCACCAAAGATTCTCTCCATGAAGTCTAAAGGGGTATCATAAACTC

TTTGAGGTCAAGAAGAGATATCATGAAGATTCTCTACCGAGCGCTATATAAGGGGCTCAC

CATCACATTCCACAGGCATCGCATTTCACACACAACACATCTCACTCAACCATTCCTCTC

ACCTCTCCACCAGCCATCACACTCCACTCCACTCCATCTAAGTGTTAGTACTATAGTATA

ACCTAAATAAAAGACTCATGAGAGTCTAAGTGTGAGAGTCTATGTAAGAGTCCTTGTGAG

AGTCCTGTACCTGTATAATTACGAACGTGTTGTACCAAGTCCTTGGTGTAATCTTCAGAA

GGATCTACCTTCAACCTATCAAGGTATATGAATGAACACCTGGACCGTTCTATATAAACC

TACTATGTTCTAAATATCTAATTTTATATCTTGTACATAATTCGTATTTACAGCTTGTCT

TTATTTCCTGTACTGCATTTATGTTTGCATAATAATATAAAAAAAAATTAACAACCTGGA

TTGCCATCCAATTGTCACATACTAATCTCACATCCAAGATTGCATATAGATACTGCATTC

CTATCCTCATATCTGGTGACTAGACCCCTAGTCACTGCCCTGGGTTAGAGC

>PpersV_sc1 [endogenous-virus-name=Prunus persica virus] [strain=sequence cluster 1] [host=Prunus persica cultivar Lovell] [moltype=genomic DNA] [note=complete genome] 7802 bp

TGGTATCAGAGCCGTAAGAGTTACGGTCTTTGTCTAAGTCTGCTACTCATTCCCGCCCAA

GTGGCTGGTTTAAGCATCTGAGTAGAAGGTTTCATCTTCTTCCTTTTCTTTTCTTTTATT

CTCCAAGTTGTCTGTTCATCTTTCTCTAAGGTCTGACCCGTGAGTCCTTTTTGCTAGATC

TGTAAGTCCCGAGGGTAGGGCGTTTGTGCTGGGCAGGAGACAGAAAATTGAGAAAGGTTA

ATAGGTTATTTGTAGAGTAAACGGTGAGTCTAGGAAGTCCCTGTTTATCTTCTTCCTCAG

TCCGTTTGAGTCAAACACTTGAGTAGCCATGAGTCGTTTTCTTAGGTCGAATTCCTGTAA

GTCAAGCAGTTCTAAGACCAGTATAAGTCGACTCCCTGAGATAGTTAATGAAGAACAATT

TGAATATGATTCTAGTTGCAAAGAACAATTAGATTTTAGAGATTGGAATATCCCTAAAGT

CCCGAGTCAAAAAATCTATAAGAAACATTGGTTGCCTTCCAGTTTTAATAGCACCACACA

TGTCAAAACAGTTGAACAAGTTTACGCTCTTAGTAAAGAACACGAAACTTGCCAGTTGCT

TAATCTTGAGTCAATCAAAAAGCACAAGACTGATGGAAACAATTTCCTGCATATTGGTCT

TGTTCAAGTTGCCGTTAAGCCCCTAACCAGGTTAGGATTGAAAGCCTCCATACTGCTCTG

TTTACGCGATGCCCGGTTCACCGCGTTCAGTGACAGTACGCTAGGAGTTATTGAGTCCAG

TTTGTGTAACGGTCCAGTTCATTTTGATTGTTACCCAGACTTCACTGTAAGCCTTAGTGA

TCCACATATATTGAGAACCCTAACTCTCAATATTAAGACTGAAGGTTATAATGTCCTCCC

AGGAACCCAACCTTTAGCATTAGTTTACAGAATTTACTATAAGGTCACAGGAACCAATAT

GAACTTCCAGGCCTTAAACAAAAGCCCAAAGGATCAGACAGTCCTGATCCAGAGTCATAC

CTCAGACGCCCATATTCAAGTTCCCCAGACCATTAAATGGTCAGAAGTTGCCCTTCCAAC

AGATTGGACCCTAGTTACCGAGAGCCAACCAGCTCCGATTCAACGTAGTCTAAACAATTT

AGACTACATCCAGCAGTACTTAGATGGCACGGTTAAGATTAGGTTCGATAGCCAGCCTCT

ACGAAAGTCTAATGTCCAGTTACACCAGTTACCAACCCCAGGTCAGTCACAAAGGCATGC

CCCAGCTAGGCACTCCTTTACTGCCTCATCCTCAACTCTAGAACGAGACCTAGAGTTAGA

AAAAACCATGATAGATTTAAAGCTTGAGTCCCTGAGAAGATCCTCTCAGGTAACCCAGCC

CTGCTACGGCAAGGCTCCAGTAGAAGAAAAGTCAGAATCCCCTGAGTCACCTACCCAGTC

TGACTTTGTAGTTGAAAACTATGCCACAGTTGATAACCAGTTAAGAACCCTTAATAGAAA

ATTTAAAATTGATTGGTCACGTCTAAACAGTCATCTCAAAGCCCCTGAGAACCTGTCTAG

ACGAGATAGTTATCACCAAGCCTATCCAGAAGCCCAACGCCGCCTAGAAATCTTTAGTGA

ATGGAAAGATTACATGCGGTCAGCCCAAGTTGAAATTTTCTACCTTGATTTTGTTGAAAG

CCGTTATATGTCAAATGATGAGTTAAAAACCTTAACTCAGCAAAAATGGAAAATGGCAGA

TAAGTCTGTCGTTGAGTCTAGCCATCCTCCAGTAGAAACCATAATTATTGAGCATAAAGA

TACTCTTATTCCTGCTACTCCTTTCAAAACATTTGAAAGTAGTGACAGTAATAGAAGAAT

TCTTGAGCAAAATAATTATACCAACCAGAGTCTAATCGTTATAGGAAAACAGTTGGATAC

AATAGAAACTAAAATTGATAAGTTAGGTTCTACAGACGTAGAGTCTAAAAAGAAAGTTGA

AAAACCAATTGTCCAGTTCCAGGATCTTAGGTCTAGCCCGACCCTTAAGATCACCCCAAC

AATGAAGAAAATAGAAGACATGTTAGCCCAGTTGACCCCTGCCAAAGTTGAAAAAACTGA

GAAATCTGGTCTTAAAACACTAGATGCCTTTGCAGTAGAAAACACCTCTGAGTCTGAGTC

TTCAGAAGTAAGTAATATTTCTAAAATTGAAAAAGCCTTTCAAACTTTAGAAATCCAAGT

TGAACCCAAAGTTAAGAAATTAGAAAAGTTTGTCAGCCCCAGTAGTCTGACCAAAAATTG

GTATCCCAGACCAACACCTCCTGACATTCAATTTGAAGAAAGAAACCTGCAGACCCAGTT

CTCTGTTTCTTCCGACAAATTGTATGAATGGAATATTGATGGTCTCTCTGAGCAAGAGAT

CCTCAATAAGTTACAACATATGTCCATGGTTGCCAATAGTTATATCACCAATCACAGTTT

CAGACAGTCTGAAATTGTCCCTCTGTTAGTTACTGGTTTCACCGGTACTCTCAGAAGTTG

GTGGGATAAACATCTGAGTCCTGAGTCTAAAAGCCGAATAACCTTTGCTGTAAAACTTAA

TGATGACGGTCACCCTATCTTTGACGAACGAATAGGTCAAGGTATAGAAGACGGTGTCAA

CACTCTGTTTTACACCATAATCGACCACTTCATAGGAACCCCTAGTAATCAAACTGCTAG

GATCCATGATCAGTTGAGTAATCTTAGATGCCCAAAGTTGTCCGATTTCAGATGGTATAA

GGATGTCTTCATCTCTAGAGTAATGCTTAGAGACGATAGTAATCAACCATTCTGGAAAGA

AAAATTTGTTAATGGTCTGCCAAACCTATTTGCCCATAAGATCAGAACTACCCTCAGTAA

TGAATTAGGTCATATTGATTGGGATAGTTTAACTTATGGTAACATTGTAAGCACAATTAA

TCAAGTTGGCATGAAAATGTGTGTTGATTACAAAATCGGAAAACAAATACAGTCCGATAG

GAAATCAGCCAAGTATGAGTTAGGAAACTTCTGTGAACAATACGGTCTCAGTAGTATTCC

GCCCTCTAGAAGAAACAAGTCTAAGCCAAGTCACCTTAGGAAACGCCGGTTTTCCTCTAA

AAGAAAACATTTTGCTGTTAAAAGTGATAATGAGTTTTATAAGAAAAGAAAAAGTTTTAA

AAGAAAAAATTGGTCTAAAGCCCCCAGAAAAGCCCAGAAGAGTCGACCCAAAACCGATAA

GTCCAAAGTTAAATGTTTTAAATGTCAAAAATTTGGTCATTATGCCTCTGAGTGTAAAGT

TAAAGATGCAATTAAGCAGTTGCAGATCTCAGAAGCTGAAAGAGAAAAGTTGATCCAAGT

TTTAGAGTTGCGAGACTCTGAAACTAGTAGCCCCGAAACCTTAGTTGCCAGCTCTGGATA

TGATTCTGAACAAGCTTCAGATTCCCAGCCTAGTTCCCCTGATATCCAATTTGGTTGCAC

CGATAAGTGTTGTAATAGATTAAAATCTATTTTTGTTCTTACAAAACAGGAAGAACAAGA

AGAGTTGTTAATCGACTTAATCAGCCGAGTAGAAAACCCTGAGTTAAAGTCCGATTATTT

GAAAAAATTGCGAAAAGTCATCAGTCAAGAAAGCTCTAGTAGTCCTAAACCGCAGCCAGT

CAGTTTAGCCACTACTATAGAAAAGTTTGCCCAGAAAAAGAAAGAAGTTACCTTACAAGA

CCTCCAGTTAGAAGTCAAAATAGTCAAACAAGAATTAGCCGAGTTAAGACAGATTAGTAA

CCAGTTGCAGGTTGAAAACCATACAATCAGGCAAGACTTAACAAGTCTTCAAAAAGGCAA

AATGCCTTTAGAGCCTAGTAGCCAGTCAGACAGCCCTGCTGGTTCTGACGAAGAAACCCC

AGTTGTCAACTTAATCAAACAAGTCAATTTCCGAAAATGGTATTCGAAAGTTACAATAGT

TGTCAAAGATTTTGAGTTTACCACAGTTGCCTTATTTGATTCAGGAGCCGACCTTAACTG

TATCCAGGAAGGTCTAATTCCTACTAAGTACTACCAGAAGTCTAGAGAAACCCTAAGCAC

TGCTTCAGGAAAATCTCTCCAGTTGAATTATGAGTTGCCTAAAGCCCATATTTGCCAAAA

CAAAGTTTGTTTTAAAACTTCATTTGTCTTAGTTAAAAATATTACTGATGAGGTTATCTT

AGGATTACCTTTCATAAGCCTGCTTTATCCCTTCCAGGTAGAATACGACGGTGTTATCTC

TACCCAGTTAGGCGAGAAAGTTAAGTTTAGTTTTCTCTCTAAACCTGAGTTGCATAATCT

GAAAGCCCTACAGAAACAGTCTGTCTCTAGGTCCCTTCAGGTTGTCCAGAAAACTAACCA

GTTGAATTTCCTTAAAGAAGAAATTAAGTTTAAAAGAGTTGAACAACAGCTTGCCAGTCA

GTCCTTACAGTTGCAAATTCAAAAATTTGAACAAAGATTAAAAGAAGAAATTTGTTCTGA

GTTGCCCACAGCCTTTTGGTATAGGAAACAGCATGTTGTCCGTCTTCCTTATATCAAAAC

CTTTAGTGAGAAAAATATCCCCACTAAAGCAAGGCCAATCCAAATGAGTCAAGAAATGAT

GGAGTTTTGTAAGAAAGAAATTGAAGAGTTGCTTCAAAAGAAAATAATTCGCAAAAGTAA

GTCCCCCTGGTCATGTCCTGCTTTTTATGTTCAGAAAAATGCTGAATTAGAAAGAGGAGT

TCCCAGATTAGTTATCAACTATAAGCCCCTTAATGCGGTCCTAGAATGGATTAGGTATCC

CATACCTAATAAAAGAGATCTCATTAATCGATTAGAAAAAGCAGTTGTCTATTCTAAGTT

TGATATGAAAAGTGGTTTCTGGCAAATCCAAATAGATGAGTCAGATAGATATAAGACTGC

ATTTGTCACTCCCTTTGGTCATTACGAATGGAATGTAATGCCCTTTGGTTTGAAAAATGC

TCCTAGTGAATTCCAGAATATCATGAATGATATATTCAATCCTTACAGTCAGTTTTCGAT

TGTTTATATTGATGATGTTCTAATCTTTTCTGAGTCAATAGAACAACATTGGAAACATTT

GAATAAGTTCCTTCAAGTTGTCAAACAAAATGGTTTAGTTGTTTCTGCCAAAAAGATCAA

GTTGTTCCAAACCAATATTCGTTTTCTTGGTTTCAATATTTGTCAGTCGCAAATCTCCCC

AATTGATCGAGTCATTCAGTTTGCTGATAAGTTCCCTGATCAAATTCTTGATAAGAGTCA

ACTCCAGAGGTTTTTAGGATCTCTAAATTATATCTCTGACTTCTATCAGAATCTGAGAAA

ACAGTGTAAGCCCCTATTTGATAGGTTGCAGAGTAATCCTCCTCCTTGGTCGTCTACTCA

CACTGAAGTTGTTAAACAGATTAAGTCTCATGTCAAGACACTTCCCTGTCTTGGTATTCC

GTCAGTTGATTCTTTCAAAATAGTTGAAACAGATGCCTCTGACATAGGTTATGGTGGCAT

TCTTAAGCAAAAAGTCAGTCCAAGTTCTCCAGAACAAATTGTTCGATTCCATTCTGGAAC

CTGGACTCAGTCGCAGCATAATTACAGTACTATTAAAAAAGAAATATTAGCTATTGTATT

ATGCATTAGTAAATTTCAAGATGATTTGCTTAATCAAAAGTTTTTAATCAGAGTTGATTG

TAAGTCTGCAAAACATGTTTTAGAAAAAGATGTTCAAAACATTGCATCTAAACAGATTTT

TGCACGATGGCAAAGTATTTTAAGTATATTTGATTTTGAAATTGAATATTTGAAAGGCAG

TGAGAATTCTGTTCCTGATTTTCTCACTCGAGAATTCTTGCAGGATGCACAATGGCTCCC

AAGAAAGACAAACAAAAAGCAGTAGCCAGTCAGCCCAAGTCGCCCAAGTCAACCCAGTCA

TCCCAGTCGCTCAGAGTCCTTCCTTCCTCCATGAGTCAACCTAAGCCAGAGTTGCCCAAG

CAAATAGTTCCTTATCCGGGTCACCCCCCATTATCAGTTGCCACCCCAATCTCAGTTGCA

AACAGATTTTCCTCTTTAGGATCAACAGTTGGCCAAATTCGCCCCAGTTATCAATCTACC

TTAATTTCTAGTTATGACCCCTTTGCAGTTGAAATCCCTGCAGGGCCATCAGTTGTCCAC

AAAAAATCGTCCCCTTATTTGCCTAAAAGTAATTCTCATTTATTTGTCATTGAGTCCGTA

TATGATATTCATGCAAATCCAGTTGAAATTGCCAAACACTATTTCCCTCCTGGATTTCAT

TATATGCCCCAGAGTCCCTATAAGTCCCTCAAGTATTATAGAGATATATTACTTGAGACA

CAATCAGTTGAAATTAAGCCGATAAAGGATCGGGATCATCCTGAAACCATTCTTTATCAT

TCTCTTTACATTCATCAAATTGTCAGTCAACAATCTTTCAGTAGCCATCCTTATGAGTTG

AAAATGCTTAAGTCAAAATTACAATATAATTATTCGGATTATATTGAAGCCTGGTATACT

ATCTTTCTTCACCAATCTGAAGATTTTAGTCATTCCTGGTTTATTAATTTTGATAATAAG

TTCAAAAGCCCTTTCCCCTACTGGTTTCTTCACTGGTGGGAAAAATATGGTCCAGTTGAT

GAAATTCTACCAGTATCAGTTCTTGAGTTGATCCGCCATTATACCAAAAGAGCCAAGTTT

TCCCTAGTTGATTTATTCTTCCCCAAACAGTTGCTGTTTATTGCCAGATACAAGGTACCT

TGGATCCTTAAATGGTCCTATGCCATCACCAAGGACACCCGTGTATTTGCCCGCCAATTC

TCAGTCAAATGGTGGGACAAGTTTGAAGTTGACAGAATTGCCAAATACGTCTACAATGAC

CTCCCCCGGGAGCCAGTTGCTCGCCCAGTATCTCCTACAGGCTCAGTCAGATCATCCTTA

TCAATTGAAGGAAAGTCTAAAGCTGAGCTCCAGGAAATTGCACGCCAGCTGATCATCCAG

GCTTCTCAGATGGATGATGATGAGGACAATGATTCTCCTCCATCGTCTAGTTGCCAGCCC

ATGCAGAGTCCTGCCAAGACAAAGCCATGGTTTGATGACAGCCAGGACCCCTATGATGCC

TACGATCTAGGGGAATCCTCTCCATAATTGCCTCAGTTGCCGACAAGCAAGCCTCGTCAA

AAGAAGTTGCCAATTTGTCAAAAGTAGTTGCCGACAGACCACTCTGTCAAAAGAAGATGC

CAAACCACGTGAAAGCAGCAGACAAAGCATCTCTGCTTTTACTTTAGTCAGATCACCGAA

AAGCAGCAGACAAAGCACTGTTCGCTTTTTGACAATTCACCGAAAGTAGCCGAAGCATCT

GCAGAAGTCAAAAGTTACCTCTACAGTTGCTAAAAGTTGAATCAGGTTGAACACCCGTGC

TTCTACAGCTGTCCAACCATAGTTGCTTTTGTAAACCATAGTTGAAAGTTGTCTATAAAT

TCAGGCTTCGGCCTCAGTTGTAAGGCAGAACTGGTTTTACCAGCTGCATTTTATTTTCTT

AGTTTTAAAGATTCTCTCTGAAACACCTTTAGAGAAATCACGCTTGTACATAGTCTAGTT

GTGTTGAAGTTTGCAGTTGCAACCTTTGTGAGTATAATAAAAGTTGCTATTTTTAAATTA

TTCTATTTTGACTAATTTCATATATGCATTATTTTCTGCATATGCATTAGTCTGAATATT

GAGTTAAGTTGCTGCATCATATTCATCTTCTTTCTCGACTATCTCTCTGTGTCCCCTCGT

TT

>PpersV_sc2 [endogenous-virus-name=Prunus persica virus] [strain=sequence cluster 2] [host=Prunus persica cultivar Lovell] [moltype=genomic DNA] [note=incomplete at 3’ end, mutations in ORF1] 7391 bp

TGGTATCAGAGCCGTAAGAGTTACGGTCTTTGTCTAAGTCTGCTACTCATCCCCGCCCAA

GTGGCTGGTTTAAGCTTCTGAGTAGAAGGTCTTCATTTCCTTCCTTTCCTTCCCTTTTAT

TCTTTGAGTTATTTGTTAATCTTTCTTTAAGGTCTGACCCGTGAGTCCTTTTTCCAAACA

GTAAGTCCCCAGTGTAGAGGGCCTGAGCGGTGGGCAGGAGACAGAAAATTAAGAAAGGTT

AATAGGTTTCTTTTAGGGTAAACGGTCAGTTTAGGAAGTCTCAGTTTATCTTCTTCCTCA

GTTAGCCAAATCCGAACACTTGAGTAGTCATGAGTCGCTTTCTTAGAACAAACTCCTGTA

AGTCAAACAGTTCTAAGACCAGTATAAGCCGACTCCCAGAGATAGTTAATGAGGAACAAT

TTGAATATGATTCCAGTAGTAAAGAACAACTAGACTTTAGAGATTGGAATATACCTAAAG

TCCCGAATCAAAAAATATATAAGAAACATTGGTTGCCTTCTAGTTTTAATAGCACAACTC

ATGTGAAAACAGTTGAACAAGTGTATGCTCTAAGTAAAGAACATGAAACCTGCCAATTGC

TTAATCTTGATTCAATTAAGAAACAAAAAAATGATGGTAATAATTTTCTGCATATTGGGT

TAGTTCAAGTTGCTGTTAAACCCCTAACCAGATTAGGTTTGAAAGCCTCAATCCTTCTCT

GTTTACGTGATGCTCGGTTTATTGAGTTTAGTGATAGCACCTTAGGCGTCATTGAGTCCA

GTCTGTGTAATGGCCCAGTTCATTTTGATTGTTATCCTGATTTCACTGTAAGTCTTAGTG

ATCCTCATATCCTGAGAACTTTAACTCTGAATATTAAGACTGAAGGTTACAATGTCCTCC

CAGGAACCCAGCCTTTAGCCTTAGTTTACAGAATTTATTACAAAGTTACAGGTACTAATA

TGAATTTCCAAGCCCTTAATAAAAGCCCAAAAGACCAAACTGTTTTAATCCAGAGTCATA

CTTCAGATGCCCATATCCAAGTCCCTCAGACCATTAAGTGGTCAGAAGTTGCCCTTCCAA

AAGAATGGACCTTAGTCACTGAAAGCCAACCAGCTCCAATTCAACGAAATCTCAACAATC

TTGATTACATCCAGCAATATTTAGATGGCACAGTAAAAATTAAATTTGAAAATCAACCTT

TAAGGAAATCTAATGTTCAGTTGCAACAATTGCCAACCCCTGGTCAGTCTCAAAGGCATG

CCCCTGCACGGCACTCCTTTACTGCCTCGTCTTCCACTCTAGAACGAGACCTAGAATTAG

AAAAAGCTATGATTGACTTTAAGCTTGAGTCCCTGAGAAAATCCTCTCAGGTAACCCAAC

CTTGCTATGGCAAAGCTCCAGTCCAAGAAGATAAGTCTGAATCACCTGAATCACCAACAC

AGTCAGACTTCTTAGTTGATAATTTTACACCAGTCGATAATCAACTTAGAACCCTTAATA

GAAAATTTGAAATTAATTGGAAACGCTTAAATAGTCATCTCAAAGCCCCTGAAAATCAGC

TCAGGAGAGATAGGTATCACCAAGCCTACCCTGACAGTGAAAGCCGCCAAAAAATCTTTA

ATGAATGGAAAGATTACATGCGGTCAGCCAAAGTTGAAATCTTTTACCTCGATTTCATTG

AAAGCCACTATATGTCTAATGATGAATTAAAAACTTTAACTAAAGAAAAATGGAAAATGA

TCGACAAGTCAGAAGTTGAAGCTAGTCATCCTCCAGTTGATACCATAATCATCAACCATA

AGAATACTACCTATACCTGCCACCCCCTTCAAAACCTTTGAAAACGATGATGATAATAGA

AGACTTATTGAGCAGAATAATTATACCAATCAGAGTTTAATCGTCATAGGAAAGCAATTA

GATACAATTGAAACCAAAATAGACAAAATAAGTTCCCCAGAAATTAAGTCCAAAACAAAA

GTTGAAAAACCAATAGTTCAGTTCCAGGATCTTAAATCCAGCCCCACACTTAAGATAAAA

CCCACAATGAAAAAAATTGAAGAAATGTTAGAACAGTTGACCCCAAGTAAAACTGAGAAA

GATGAAAAATCTGGTCTTAAAACACTAGATTCTTTTCCAAACACAAACTCCTCTGAGTCT

GAGACAACAGAATCTGAAACTAGTAATATTTCAAAAATAGAAAATGCTTTCAAAAATTTA

GAAGTCCAAGTTCAACCCAAAATCAAAAGATTAGATAAACACATAAGCCCCACTAGCTTA

ACCAAAAATTGGTACCCTAGACCAACACCTCCAGATATCCAATTTGAAGAAAGAAACTTC

CAAACCCAGTTTTCAGTTTCTTCTGATAAATTGTATGAATGGAATATTGATGGTCTATCT

GAGCAAGAAATCCTGAATAAGTTACAACATATGTCCATGGTTGCCAACAGTTACATCACC

AATCACAGTTTCAGACAGTCTGAAATCGCTCCCCTGTTAGTTACTGGATTTACTGGTACT

CTCAGATATTGGTGGGACAAACACCTAACCCCTGAGTCTAAAAATCGAATCACTCATGCT

GTAAAACTCAATGAAGATGGTCTCCCTATATTTGACGAACAAATAGGTCAAGGTATAGAA

GACGGAGTCAACACTTTATTTTACACAATAATTGAGCATTTCATTGGAACCCCTAGTAAT

ACCACTGCTAGGATACATGACCAGCTAAGCAACCTTAGATGTCCCAAGTTGTCTGATTTC

AGATGGTACAAAGATGTCTTCATCTCCAGAGTCATGCTTAGAGATGACAGTAATCAACCA

TTCTGGAAAGAAAAATTTGTTAATGGTCTTCCAAACCTATTTGCCCATAAAATACGCACA

ACCCTGAGTAATGAACAAGGTCATATAGATTGGGATAGTTTGACTTACGGAAACATCATA

AGTACAATCAACCAAGTTGGCATGAAAATGTGTATTGACTTTAAAATTAGTAGACAAATA

CAGTCCGATAGGAAATCAGCCAAATACGAATTAGGCAATTTCTGTGAACAGTACGGCCTT

ACCAGCATCCCTCCTTCCAGAAAAAATAAGCCCAGTCACCTTAAAAAAAATCGTTATTAT

TCCCGAAGAAAACAATTTTCTAAACACAATAATGATAATCATGAATTCTATAAAAAAAGA

AAATTTTCCCCCAAGAAAAATTGGTCGAAAACCCCAACAGGCCAAAAGAAGAATCACCCC

AAGAGAAATATGATAAAACCAAAGTCAAATGCTTCAAATGCCAAAAATTTGGTCATTATG

CCTCTGAATGCAAAATAAAAGACACTATTAAACAATTGAAAATTACAAATGAAGAAAAAG

AAAAATTGATAAAAGTTTTGGAGTTGCGAGACTCTGAAACAAGCAATGATGAAACCATAG

TAGCCAGCTCTGAGTCTGATTCTGACCAGTCGACAGAATCACAGTCAAGTTCACCCAATA

TCCAAATTGGTTGCACAGATAAATGTTGCAATAAATTAAAATCTATTTGTGTCCTTACTA

AACAAGAAGAACAAGAAGAATTACTAATAGACCTAATAAGCAAAGTTGAGAATCCCGAAT

TAAAATCAGAATATTTAAAGAAATTAAGAAAGGTCATTAGCCAAGAAAGCCCAAGTCATT

CTAATCCCCAGTCGATTAGTATAAATACTACTTTAGAGAAATTTGCTCAAAAGAAAGAAA

TTACCTTACAAGACCTCCAATTAGAAGTAAAATTGATTAAAAAAGAGATCGTTGAATTAA

AACAAATAAGTCATAAGTTACAAAGTGAGAATTACACGATCAAACAAGATTTAGCCAGTC

TTTTGAAAAAAGAGTCTTTTGAGTCCAAAAGCCAGTCAGAAAGTCCAAACAACTCTGATG

ATGACACCTTCTGATAGCCAACAAGTTGTAAATTTAATTAAACAAGTTAACCTTAGAAAA

TGGTACTCGAAAGTGACAATTTTCGTCCAAGATTTTGAATTTACCACAGTTGCCCTATTT

GACTCAGGAGCTGATCTTAACTGTATTCAGGAAGGTCTAATTCCTACTAAGTTTTACCAG

AAGTCTAGAGAATCCCTAAGCACTGCTTCAGGAAAAACACTCCAGTTAAATTTTGAACTG

CCTAAAGCCCATATCTGCCAGAATAAAGTCTGTTTCAAAACCTCATTTGTCTTAGTCAAA

AACATCACTGACGAAGTCATTTTAGGATTACCTTTTATTGCCTTATTGTATCCCTTCCAG

GTAGACTATGATCGTGTCATCTCTACCCATTTAGGCGAAAAGGTCAAATTTGAATTTCTG

TCTAAACCAGAGTTGCATAATTTAAAAACCTTGCAAAAACAGTCAGTGTCCAAATCAATA

CAAATCATTCGAAAAAATAACCAGTTGAACTTTCTTAAAGAAGAAATCAGATTTAAAAGA

ATTGAACAACAGATTGCTGATAAATCACTACAATCGCAAATCCAACATTTTGAAAACAAA

TTGAAAAATGAGATTTGCTCTGAGTTGCCTAATGCCTTTTGGTACAGAAAACAACATGTT

GTCTGTCTTCCTTATATCAAAACCTTTAATGAAAAAAACATCCCTACCAAAGCCAGACCA

ATCCAGATGAGTCAAGAAATAATGGAATTTTGTAAAAAAGAAATTAACGAGTTACTTCAA

AAGAAAATAATCCACAAAAGCAAGTCACCGTGGTCATGTCCAGCTTTTTATGTCCAGAAA

AATGCTGAACTAGAAAGAGGAGTCCCGAGATTAGTTATCAACTACAAGCCACTTAACTCA

GTTCTAGAATGGATTAGGTACCCCATACCTAACAAAAGAGACCACATTAATAGATTAGAA

AAAGCAGTTGTTTATTCCAAATTTGACATGAAGAGTGGTTTCTGGCAAATCCAAATAGAA

GAGTCAGATAGATATAAGACTGCATTTGTTACTCCCTTTGGTCATTATGAATGGAATGTA

ATGCCATTTGGTCTAAAAAACGCCCCTAGTGAATTCCAAAATATTATGAATGAAATACTC

AATCCCTACAGCCATTTCTCAATTGTCTATACTGATGATGTCCTCATCTTCTCCGAGTCT

ATAGAACAGCATTGGAAACATTTGCATAAATTCTTCCAAATAGTCAAACAGAATGGTCTA

GTTGTTTCCACCAAAAAAATCAAGTTATTCCAAACCAATATCAGATTTCTTGGTTTCAAC

ATATACCAGTCACAAATCTCCCCAATTAATCGAGTCATCCAATTTGCTGACAAATTTCCT

AATCAAATCCTTGATAAAAGTCAACTCCAGAGGTTCCTAAGATATCTAAATTACATTTCT

GACTTTTACCAAAATCTGAGAAAACAATGGTAAGCCCTTATTTGACAGATTATTGCAAAA

CAATCCTCCTCCATGGACAACCACCCATACTGAAGTTGTCAAACAAATCAAAGCCCATGT

CAAGACACTTCCCTGTCTTGGTATTCCCTCAGTCAATTCCTTCAAAATAGTTGAAACCGA

TGCCTCTGACATTGGTTATAGTGGTATCCTCAAACAAAGAATTTGCCCAAAATCTCCAGA

ACAGATTGTTCGTTTTCATTCTGGAACTTGGACCCAGTCGCAAAATAATTATAGTACTAT

TAAAAAAGAAATATTAGCTATTGTACTATGTATTAGTAAATTCCAAGATGACTTGCTCAA

TCAAAAGTTTTTAATACGAGTTGATTGTAAATCTGCAAAACATGTTTTAGAAAAAGATGT

TCAAAACATTGCATCTAAACAGATTTTTGCACGTTGGCAAAGCATTTTAAGTATATTTGA

TTTTGATATTGAATATTTGAGAGGAAGTGAGAATTCAATTCCTGATTTTCTTACTCGAGA

ATTCTTGCAGGATACACAATGGCACCCAAAAAAGACAAGCAAAAAGAATCAGCCAATCAA

CCTAAGCCACCCCAGTCGCTCCGAACCCTTCCTCCATCCATGAGTCAAAATAAGCCAGAA

ATGCCAGAAATGCCCAAACAGTTGATTCCTTTTCCTGGTTGCACTCCCATTTCAGTTGCC

ACGCCAATATCAGTTGCTAATAGGTTTTCTACCCTAGGATCCACAGTTGGCCAAATCCGC

CCCAGTTATCAGTCAACCCTAGTCTCTAGTTATGATCCTTTTTCAGTAGATACCCCAGCT

GGCCCCTCAGTTGCCTTCAAAAAATCATCCCCTTATTTGCCCAAAAGCAATTCTCACTTG

TTTGTTATTGAGCCTGTTTATGATATTCATACTGATCCCATTGAAATAGCCAAACACTAT

TTCCCCCCAGGATTTCATTATATGCCACCCAGTCCATACAAGTCTCTCAAGTATTATAGG

GATATACTACTTGAAACCCAGTCAGTTGAAATCAAACCAATAAAGGATCGAGATCACCCT

GAGATCATCCTTTATCATTCCCTTTATATTCACAAAATCCTGAGCCAAGAATCTTTTAGT

AGCCGCCCTTATGAATTAAAGCTCCTTAAGTCAAAATTACAATATAATTATTCTGATTAT

ATTGAAGCCTGGTACTCTATTTTCCTCCACCAATCTGAGGATTTTAGCCATTCCTGGTTT

ATCAATTTCGACAATAAGTTCAAATGCTCTTTTCCCTATTGGTTCCTCCATTGGTGGGAA

AAACATGGTCCAGTTGATGAAATTTTGCCTGTCTCAGTTCATGAGTTAATTCGCCATTAT

ACCAAAAAAGCCAAATTCTCCAAAGTTGATTTATTCTTCCCAAAACAGTTGCTCTTTATT

GCCAGATATAAAGTCCCTTGGATCCTCAAATGGTCCTACAGAATAACCAAAGATGCCAGA

ATATTTGCCCGTCAATTCTCTGTTAAATGGTGGGACAAATTTGAAGTTGAAAGAATTGCC

AAGTATGTCTACAATGATCTTCCCCATGAGCCTATTCCGCACCCAGTTTCTCCTACTGGT

TCAGTTCGATCTTCTCTCTCAGTCGAAGGGAAATCAAAGTCTGAACTCCAAGAAATTGCT

CGCCAGTTGATCATTCAAGCCTCTCAAATGGATGATGATGAAGACAATGATTCTCCTTCG

TCATCTAGTTGCCAGCCAATGCAGAGCCCTGCCAAGCCTAAGTCCTGGTATGAAGATAGT

CAAGACCCCTATGATGCCTATGACCTGCACTCTGATTAGGCTCCTAGTTGCAGACACAAT

AATTGCCCCAAGTTGCAGACAAAATAATTGCCTCGGTTTGAAGCATCCATCCGCTTTCTC

ATCACTTTACCAAAAGTGGCATGAGCAAAAGAATAGTAAAAGTTATTCCTTCATAAAAAG

ACAAGAGTGAAAGTTGAATCAGTTCCTTCCCGTGCCTCTACAGTAGCATTAACCGTAGTT

GCTTTTTACTA

>PperV_sc3 [endogenous-virus-name=Prunus persica virus] [strain=sequence cluster 3] [host=Prunus persica cultivar Lovell] [moltype=genomic DNA] [note=incomplete at 3’ end, mutations in ORF1] 6866 bp

TGGTATCAGAGCCGTAAGTGTTGAAGAGTCGGTCTAAGTTTGCAATTCATCCGCCCAAGT

GGCTGGTTTAAGATTTCTAAGTCGAAGTCTTCAGTTCCTTCTTCTACTCCTTCTTGTCTT

TCAGTTTACCTGTCTTTTTCTCTCTGGTCCAAACAGTAAGTCCTAGAGTCCAAACAGTAA

GTCCCATGTAGAGGCATGAGCGGTTGCGGCCTGAGCGGAGAGAGAAAGAATCACTAGGTG

TCCTATAAAGCCATAAATGAGGAGTTTCTATTTATCTTCTCCTTTAGAAGTCTAGTCCAG

AACCTTGAGTAATGAGTCGCTTATTTAGAACCAACTCTTGTAACTCTAGCAGTTCTAGAG

CCAGTATAGTAAACTTCCAGAGATAGTTAATGAAGAACAAGTTGAATTTGAGTCTAGTAA

TGAATTAGATTACAAAGATTGGAATATACCGAAAATCTCTAGTCAAGATATTTATAAGAA

AAAATGGTCGCTTTCCAGTTTCAAGTCAACCACCCATGTAAAAACAGTCGAACAAGTTTA

TGCTTTAAGTAAAGAACATGAAACATGCCAGTTGCTTAATCTTGAGTCAATTAAGAAACA

TAAAAATGATGGAAATAATTTCTTTCACATTGGTCTAGTTCAAGTTGTAGTTAAACCTCT

AACCAGATTAAGATTAAAAGCTTCTATCCTTCTCTGTCTTAGAGATGCTAGATTCACCAG

TTTTAGTGACAGTATCCTAGGAATAATTTAATCAAGTCTGTTTAATGGTCCAATTCATTT

TGATTGTTATCCAGATTTACAGTCAGTCTCAATGATACTCACATAAAAACCTTAACTTTA

AATATCAAAACTTCTGGGTATGAAGTCTTACCAGGTACCCAGCCATTAGCCCCAGTCTAC

CGAATTTACTACAAAGTCACAGGTACTAATATGAATTTCCAAGCTTTAAATAAAAGTCCA

AAATATCAAACCTTGTTAATTCAAAGTAACAAAGCCGATGCCAACATTAGAGTTCCCCAT

ACAATTAAATGGTCAGAACTTACCCTTCCATTAGATTGGACGTTAACCACAGAGAATCAA

CCAGTCCCCATTCAACACAGTTTAAACAATTTAGATTACATTCAACAATATTTAGATGGC

ACAGCCAAAATTAATTTTGGAAGTCAACCTCCACAAAAGTCCTCAGTTCAGTTACACCAG

TTGCCTACACCTAGTCAATCCCAGAGACATGCCCCTGCAAGACATTCTTACACAGTTTCT

TCCTCTACTCTAGAGCGAGATCTAGAATTAGAAAAAACCTTAATTGATTTAAAATTAGAA

TCCTTAAGAAAAACTTCCCAAGTTAACCAGCCCTGTTATGGTAAAAAACCAACAAAAGAA

GATACTAAGTCAGAATCTTCAGAGTCACCCACACAAACCGATTTCGAAGCCGGTTTTCAA

GTTGATAACCAATTAAGAATCCTCAATAAAAAATTTAAGATTAATTGAAAAAGTCTAGAT

AATCATTTACATGCCCCAGAGAATCAAAAAAGAAGAGACAATTATCACAAAGCCTACCCT

AGTCGAGAAAAACGACAACAGATCTTTAATGAGTGGAAAGATTATATGCGTACAGCTAAG

TTAGAAATTTTCTACCTTGATTTTATTGAAAGTCATTATTTATCTAATGAGTTAAAAACT

TTAACCCAAGAAAAATGGGAAAAAGAAGATAACACCTAAGTCATAGCCACTCATCCTCCA

GTAGAAACAATAGTCATTACCCATAAAAAGTCAAAAATAGTTGCCAGTCCCTTCAAAGCC

CCTGTCCAAAATGATAGAAAATTAGTTGAACATAATAATTACACCAACCAAAGTCTAATA

GTAATAAGGAATCAATTAGATAAAATAGAAACTAAAATTGACAAAATACCCCTTCAGAAA

CAACCTCTAAGTCACAATAAGAAAAACCTTTAGTCAAATTCCAAAATAAAAGAGTCAATC

CCAACTTTTAAGACAAAACCAACAATGAAAAAAATAGAAGAAATGCTAGAACAGTTAACC

CCAAAGAACGAAAAAGAGAAAATTGAAAAATCTGGTCTTAAGGTCTTAGGATCCTCTGCT

TTAGTCAATACCTCTGATTCCGAGTCAGTAACCAGTATGGAAACAGAAACCAGTAATATC

TCTAAAATTGAAAATGCATTCAAAAATTTAGAATTAGAAAATGATCTAAAAATTAAAAGA

TTAACAAACAGAGTCAGCCCCACGAGTCTAACCAAAAATTGGTACCCTAAGTCAACTCCT

CCAGACATCCAATTTGAAGAGAGAAATTCCCAAAGTCAATTCTCAGTCTCCTCCGATAAG

TTATATGAATGGAATATTGATGGTCTTTCTGAACAAGAAATTCTTAATAAACTCCAACAC

ATGTCAATGGTAGCCAATAGCTATGTAACCAATCACAGTTTTAGGCAGTCAGAAATCGTT

CCTTTGTTAGAAACATGTTTCATCGGAACCTTACGTTCTTGGTGGGATAAGCACTTAATA

GAAGAGTCAAAAAGACAAATAATTCATGCAGTCAAACTTAATGAAGAAGGTCTTCCTATC

TTTGACGAAAAATTAGGTCAAGGTATAGAAGATGGAGTCAATACTTTGTTGTATACAATA

ATTGAACATTTCATTGGAACACCCAGCAATACCACTGCTAGAATTCATGATCAGCTTAGT

AACCTTAGATGCACAGTCCTTAGTAATGATCAAGGTCATATAGATTGAGATAATTTAACC

TATGGTAATATCATTAGTACAATTAATCAAGTCGGAATGAAAATGTGTGTCGACATGAAA

ATTGGTAAACAAATACAGTCAGAAAGAAATTCAGCTAAGTATGAGTTAGGAAATTTCTGT

GAACAATATGGTCTAACATCAATTACCCCGTCTAAAACCAAGTCTAGTCATCATAACCGA

CAATTTAGTAAAAGTCGACATTATTCACGTAGGAAACAATCCTTTAGACGTAATAATGAT

AATAATGAATTTTATAGTAAAAAGAAGTTTCACCCGCGAAAAAATTGGTCAAAAGGTCAC

GGAAAAGCTAAGCAAAGTAACTTTAAGAAAAGAACCAACAAAAGGCAAGTTAAATGCTGG

AAATGTCAAAAGAATGGACACTATGCTCATGAGTGCAAAGTAAAAGACACAATAAAGCAG

TTAAAAATCACGGATGAAGATAAAGAAAAGTTTATTAAAGTTTTAGAGTTAAGAAACTCC

GAGTCAAGTGAAAATGAAACCATGGTTATCAATACTGAGTCAATCGGATATAGTTCCACA

GATTCCCAACCTAGTTCACCCATAATCCAATTAGGGTCTAAAGATAAATGCTGTAATATC

TTAAGTAAGTCTATCAATGTCCTTACTAAACAAGAAGAATAGGAATAGTTGTTAATTAAC

TTAATCAGTAAAGTAGAAAACCCAGAGTTAAAATCCGAGTATTTGAAGAAGTTGAGAAAA

GTCATAAGCCAAGAAGAGCCATGTCAGTCCATTCCCCAGAAGATAAGTGTGAACACTACC

CTAGAAAAATTCAACAAAAGAAAAGAAGTCACTTTACAGTATTTACAATTGGAAGTTAAA

TTAGTCAAGAAAGAAATAGTAAAATTAAGACAAATAAGTCACAAGTTACAAACCAAGAAT

TTTGAAATAAAACAAGATTTAATTGATTTAATGGAAAAGAAAGTTCTGAGTCTAAAGGTC

ATTCAGAGAGCCCTACAAATTCAGATTATGAACCTTCCAATCACTCTATTAATGACCAAG

CCATTAATTTAATTAAACAAGTAAATTTTAGAAAATGGTATTCTAAAGTCACTATTTTTA

TAAACAATTTTGAGTTAAACACAGTAGCCCCTTTCGATTCAGGAGCCTACCTTAATTTTA

TAAGAGAAGGTCTAGTCCCTACCAAATACTACAAAAAATCAAAAGAGTCATTAAGCACTG

CCTCAGGAAATTCACTCCAATTAAATTATGAGATTCCAAAAGCCCATGTCTATCAGAATA

AAATTTGTTTCAAAACCTCATTTGTCTTAGTTAAAAATATTATTGATAAAGTCATCCTAG

GATTACCCTTTATTGTCCTTCTGTACCCTTTTCAGGTAGAATACGATGGAGTCATATCTA

CCCATTTAGGAGAAAATGTTAAGTTTGAATTTTTAACCAAACCTGAGTTACATAATTTAA

AAGCTTTACAAAAGAGTGCAGTTTCAAAAGCAGTCAAAGTAATTCAAAGTAAAAATAAAC

AGTTAGATTTCCTTAAAGAAGAAGTTAAATTTAAAAGAATAGAACAACAGCTTAGTGATA

CGTCCGTACAGTCACAGATTCAAAAGTTTGAAGAAAAGTTAAAACAAGAAGTCTGTGCTG

ATTTACCTAATGCTTTTTGGCACAGGAAACAACACGTAGTCAGTTTACCTTATGTCAATA

ACTTTAGTGAAAAGAATATTCCCACTAAAGCCAGACCAATCCAAATGAGTCAAGAGTCAA

TGGAATTTTGTGAAAAAGAAATCACAGAGTTACTTCAAAAAGGAATAATTAGTAAAAGCA

AGTCACCCTGTTCTTGTCCTTTTCTATGTCCAGAAAAATGCTGAGTTAGAACGAGGAGCC

CAAAACTAGTAATCAACTACAAACCTCTTAATACAGTCTTAGAATGGATTAGGTACCCAA

TACCTAATAAACGAGACTTAATTAACATATTAGGAAATGCAATCGTTTTTTTCTAAGTTT

GATATGAAAAGTGGATTTTGGCAAATTCAGATAGGTATAAAACCGCTTTTGTAACTCTAT

TTGGTCACTATGAGTGGAACGTTATGCCTTTTGGTCTTAAGAATGCGCCCAATGAGTTTC

AAAATATAATAAATGAAATATTTAATCCTTTCAGTCATTTTTCCATAGTCTATATTAATG

ATGTTCTAATCTTTTCCCAGTCATTAGAACAACATTGGAAACATTTGCATAAGTTTCTTC

AAATAGTCAAACAAAATGGTTTAGTTGTTTCTGCTAAAAAGATTAAGTTATTCCGAACCA

ATATTCGATTTCTTGGTTTTAATATTTGTCAGTCGCAAATTAGTCCCATTGACTGAGTCA

TTCAGTTTGCAGAAAAATTCCCAGATGAAATCTTAGACAAGAGTCAATTTCAAAGATTCT

TAGGATCTCTTAATTATGTTTATGATTTTTATAACAAAATATGTGAAAACAATGTAAGCC

ATTATTTGACCGTTTATAGAGTAATCCTCCTCCTCGGTCATCCACTCATACAGAGTTAGT

CAAACAAATCAAAGTCCATGTTAAGACACTTCCTTGTCTTGGTATTCCTTCAGTTGATTC

CTTCAAAATAGTTGAAACTGATGCCTCTGAAATAGGTTATGGAGGTATTCTTAAGCAAAA

AGTCATTTCAAGTCAATTCGAGCAAATCGTTCGATTCCATTCTGGAATTTGGACACAGTC

ACAAAGTAACTATAGTACTATTAACAAAAAAATATTATCTATAGTACTATGCATTAGTAA

ATTTCAAGATGATTTATTAAATAAATTTTTTTTAGTTAGAGTTGATTGTAAAAGTGCAAA

ACATGTTTTAGAAAAGGATGTTCAAAACATTGCATCAAAACAGATTTTTGCACGATGGCA

AGCAATTTTAAGTATTTTTGATTTTGATATTGAGTACTTGAGAGGAAGTGAGAATTCAAT

TCCTGATTTCCTTACCAGAGAATTTCTGCAGGAAAAAGAATGGCAGAAAAAAGAACAGAT

AAGCAAAAAGAAAAGCAAAAAGAGTCTCAGTCATTTAGAACCCTCCCGGCATCAATGAAC

CCAATAAAAACAGAGTCATGTTCTCGAATCCAAATTAGTAACAGATTCACCCCACTTGGT

TCCACAGTCGGCCAAGTTCGTCCTAGTTTCCAGTCTGCCTTAGTCTCTACTCATGATCCT

TTCCAAATCAATACCCAAGTTGCCCAAACTCCAGTTTATTACCAAAAGTCATCTCCTTAT

TTGCCAAGAAGTAGTTGCCACCTATTCATTGTTGAGCCCAAATACAATAACATCACTAAT

CCAGTCACAATTGCAAAGTCATATTTTCCCCCGAATTTCCATTTTGTTCCTCCTGCTCCT

TACAAGTCACTTAAAAATTACAGAGATATCCTCCATGAGACACAATCAATTGAAATTAAG

CCCATTAAATATAGCAATAATCCCCAAGTTATCCTTTTCCATTCCTTGTACATTCATCAA

TTCCTGAGTGAAGAAGAATGGAGTAGTCACCCATATGATCTCAAAATCCTTCAGTCAGAA

CTTCAATATTGTTATTATGACTATATTGAAGCTTGGTATTCAATATTCCTCCACCAAATA

GTCGATTTCAGTCATTCTTGGTTCATTAATTTTGACAATAAATTCAAAAGTCCCTTCCCT

TTGTTGGTTCCTTCACTGGTGGGAGAAACATGGTCAAATCACCGATCTTCTTCCCCAGCA

AATGCAAGAGTTGCTTAATTTTTTTTATGTTAAAAATAAATTCAAAAAATCAGAGTTATT

TTTCCCTAATTTACTTATATTCATTGCCAAGTATAAAGTTCCTTGGATCCTAAAATCGTT

GTATCATGTCAATTGGGATTCCAGAGTTCTCTCTCGCCAATTTTCAGTCAAATGGTGGGA

TAAGTTTAAAATTGACAGAATTGCAGATTGTGTCCACAATGATTTTCCTCCAGTCATCAT

CCCTGCAAAGCCTGCCCAGAAGCCATCTTCTTCTAGTTCATCTCATGCTTCCCTCTCAAC

CGAAGGAAAATCCAAAGCAAAGTTGTAGGAAATTGCCAGACAATTGATTCTTCAAGCTTC

TCAAATGGAAGATGAAGAAGACAGTG

>PtrichV_sc1 [endogenous-virus-name=Populus trichocarpa virus] [strain=sequence cluster 1] [host=Populus trichocarpa] [moltype=genomic DNA] 7866 bp

TGGTATCAGAGCCTACTTTGGGATCTGGGTAGGAATAGTAACATAAAAGTCAGAATTCCT

AGAAAGAACAAGAGACCATTAGACAATAGGTGAATGCAACTGAATGGTGTAAGTCCCGTT

TGAGTTTTAGGGCAATAAATCCATATAACCTGCAGCCTGTTTCCTAGTTTATCTTTCCTA

TCTATGGATTCCGTTTTTCACAGGACTACTTCTATTTCAGCTTCCTCTACCTCTGAATCT

TCTGAAAGCAGAAGGATTGTACAAAGTGAAGAAATAACTATTGAAGATTTTACAAAAAAC

ATTGATGATTGGAAAATTCCAAAAATTTCTCAAACTCAGATTTATCAAAAATCTAAATAT

GATATTTTCAAAACAGATTTTACTATCAAAACTGAAGAAAGAGATATTCAGCTTACAAAA

CCTTTTGAAACCATTCAACTCCTCTCTCAAAAATCCTTACAAAAACATCTAGCCCGAAAC

TATAAATATATTCATGTTGGCCTTGTCCAAGTCGGGATAAAACCCCTTACAAAAGAAGGT

TTAAACACCTCCATTCTAGCTGTCCTCCGAGATGCTAGATTTCAAAACTTCCAAGACTCC

CTCCTCAGTTCTATTGAGTCTAGCCTTTGCAGTGGTCCGGTATCATTTGACTGTTATCCA

AACATAACCATATCCCTCAAAGACAAAAATATCTTGCAAAGTATGCTACTACAAATAAAG

ACCCATAATTATGACATGCTCGAAGGATCTATCCCTGTTGCTCTCATATTCAAAATCCAT

TACAAAGCCATGTTCTCAGCCTTTGCCAGCAAACACAAATTCCAATCTCAAAAAGGAGAA

ACACTCCTTCTACAAACAGATTTATCCCGCTCAAACACTGTTGTCCCAAAAGCAATCCAA

TGGAAAGATATATCCCTTCCAGAAGATTGGATCCTCGAAGGTGCAGCACCACCAGAATTA

CCACAACCACCTCAACCCAATGTCCAAATTAAAAACATTACCCAATACACAGATGGAAAA

GTCAAATTGTCTTTCCATCGACACTCTACAAGTTCCAGATTCTCAGAAGACTCTTCCTCT

TCCAGTACAATAGATCTGGGAAGAATTTCCAAAATCCCTTCTGTTATCAATATCCCTTAT

CATGCAAACCCTCCTAGAAAATCTACATCTGATATCCCTTCTACTAGTTTCCAAAATGCA

GACTATACTACAAATATACCAAAACCAGTTTATACAGACCTTGAGCAACCATCTCCTCCA

ACTTCACCAACCTTTTCAGCTGTTACTGAAAACATCCAAAACGAGCTGAATGTTTTGACT

TCAGAAAAGCATTTTGTCATAAACAAAACTCTTTTCAAAAGAGATTTTTATGGTGATCAT

AATATTGAAAAACGATCTTGGTTTTTTCAACATTTCCTCCAACAGAGAAATGATATCCAA

AAACAATTTTATGCTTACATTGAGACCCATAAAGTACAAATTTTATTCTTTGATTGGTTT

GAATTTCATTATGTACCAGCACAACATATTACATATCCATTTGCATCCATTAAACATGCA

TGTCCTATTACAACCCGTTCCAAAACTCCTACATGGACCCTCACCAGTGGACCTACAGTT

GAGTCTGACCATCCACCTTTACGCAACATACAACTTACTCACAAAGACCAAACAGTTGAT

GCAGCCCCATACAAATTACCCGGAGAAGATACTCTCACAAATACCAAACATATCATCCAA

CAAAACAATTTCACAAATACCAACCTCCATACCATAGGTAAACAGCTTACCAGATTGGAA

AAACAAATACAAAAGAACCCAGTCCACTTCATGACTGATAAAAACCCCATTGACCTTAAG

CTAAAAAACCCAGTATTCAAACCATATCAAGTTACTAAAACAAGCCAAGCACAAATTCAA

GAAAATCAAACAGATTTTCTTAGAGCCATTAAAACACACTTACAAAACCTTGACCGGACA

ACCTTGACTGTCCCAGACACACCTCAACAATCCAACCCTTCATCCTCTACAAATCAGGTT

AATACTCTCCAAAATAACCCCGTATCAACCTCCGATACAGATACTTTTCAAGAACAGCCT

CTTCAACTAAACAGACTTACCTGGCAAGCTCCCAAAACAACCATTGCTCCAGACATTTCC

ATATCTACTGAACCAACTGTCATTAGCCAACATAAATATAATGCATCTTCCCTCTATGAA

TGGAACATAGATGGAATGTCTGAATATAATATCTTAAACACCCTCCAACAAATGACCATG

GCAGCTAATGCCTACAAAACCCAAACAGGAACCCCAGACAAAGCCATAGCTGAACTTCTT

ATTGCAGGTTTCTCTGGCCAGCTTAAAGGATGGTGGGATTACCATCTCACTGAAACAGAT

CACTTACATATTCTAAACTCTATACAAACCTATGAAGACCAAACACCTATCCTTGACCCT

TCAGGAAACACCATCCAAGATGTTGTGTCCACCCTTATCCTTACCATCTCCTTACATTTT

GTTGGAGATCCTTCCCATCTGAAAGACAAAAATGCTGAACTTCTCTCCAACCTTCGGTGC

AAGAAACTCAGTGACTTTCAGTGGTACAAAAATACCTTCCTTACTCGTGTTATGCTTCGA

GAAGATTCAAACCAACCTTTTTGGAAAGAAAAATTCCTTGCAGGCTTACCGATTCTCTTA

GGAGAAAAGGTCCGAAACAAAATCAAGGATACCTTTACAACCAAAACCATACCATATGAC

CAGCTTACATACGGTGAACTTGTGAGCTTCACACAAAAAGAAGGTCTTAAGATCTGCCAG

GACCTCAAACTCCAAAAACACCTAAAGTGGGAAATGAAACGTACCAGGCAAGAACTTGGT

AGTTTCTGTCACCAGTTTGACCTTTCCACCAAAAAACCCTCCTGCAGTGGAACCTGTTCA

CAACCCAAAAAATATTCGAGCCAAAAACCTCCTTATAAACGATCAGCCCATAAATACCGA

CAACAATTTTATTCCAAACCAGATCAACCTTACTACAAAAAGCCCTACAAATTTACCAAA

CCCCACAGACCTTTCCAGTCAAAACCCAAACCAAAATTTGACCCCAAAAACATTACCTGT

TACAAATGTAACCAGAAAGGACATACATCCCGTTTCTGCAAAGTCAATACCAAACTTCAT

GAGCTCCAAATAGATGAAGATACCATCAACCAAATACAAAATCTTTACATTGAAGCCACA

GACACTGACCACTCTCCTTCAGACACTTCTGAAGAAGAATTTCAAATTGATGAAATAGTA

ACCACCAGTGCTACTTCAGATGCCTCTACAAATTCCAAACAAATTAACGTCTTAACCCAA

GACCAAGAATTCATTCTTGAGGCTATCAAAAGACTTGATGATCCTCATCTTCAAAAAACC

TATTTGGATAAACTGTTGAAAGATTTCAACCAACCAGAGCACCCTCCATCTAACCCTCCA

AACCGCTCTATATTACCCTCTACCAGCACCAATACCTATGACCTTACAAAGATCCTTAAC

AAAAAGAAATCCAAAACCACAGTCACCATTCCAGAATTACATTCTGAGATCAAAACCCTC

AAATCCGAATTACAAACCTTAAAACAAGCCCAACAGAAAGACTCTGCCATATTACAACAT

CTTCTGTCCAAAATTGAAAGCCAGTCTGACACTGAATCTGAACCAGAAGATCAAACCATT

GAATCTCATGCATTGCCCCATACACTTACAAACATTGAGCATATTCCAGATGATTTCCTG

AATGTATTAACACAAATATCTTCAAAAAAATATTTGATTAAAATTACTCTTATTTTTTCA

GAAGATTTCAAACTTGACACTATTGCCTTATTTGACACAGGTGCAGATTTAAACTGCATC

AAAGAAGGCGTAGTTCCAAAACGATTTTTACAAAATACCTCTGAAAAACTCTCTGCTGCT

AATAATTCAAAATTGCATATTGCAGGAAAAACCCAAGCCTCTGTTTTCAACAAAGGCATT

TCTCTTAAAACATTCTTTGTTGTTACAAAAGATATTAATCATACCATCATTCTTGGCACT

CCATTTATTGACATGATCACTCCATATCAAGCACATCATAATTGTATTACTTCCAAAATC

AATAGCATCAAACTTGTTTTCCCATTCTTAGAAAAATCTAAGACTAGAAATTTAAATTTG

ATTAAAGCATGCTCCATACATACATATCATATCAATGCACTAATTCATGGAAAACAATTC

CATCTACATGATTTACAAAACCATGTTTCTTTTTGTCGTATCAACAAACAATTACAAAAC

TCTGATTTACAAAAGAAAATCATTGATTTACAAAATAAGATTGAACAACAGATCTGTTCA

GACTTACCTAATGCTTTCTGGAAAAGAAAACAACACATCGTTGATCTTCCTTATGAAGAT

ACTTTTTCAGAAAAACTTATACCTACAAAAGCAAGACCTATACAAATGAATGCTGATTTA

GAACAGCATTGCAGACTTGAAATCCACGATCTCGAGTCTAAAGGTCTTATCCAAAAGTCT

CGATCACCCTGGTCGTGTGCAGCCTTTTATGTAAATAAAAACTCTGAAATTGAAAGAGGC

ACACCAAGACTTGTGATCAATTACAAACCTTTAAATTCTGCTTTAAAATGGATCAGATAT

CCAATCCCCAACAAAAAAGATTTATTGCAAAAATTGCACTCTGCATTCATATTTTCTAAA

TTTGATATGAAATCCGGATTTTGGCAAATCCAGATTGATCCCAAAGATAGATACAAAACT

GCTTTTACAGTTCCTTTTGGCCAATACGAATGGAATGTCATGCCCTTTGGCTTAAAAAAT

GCACCATCTGAATTTCAACGCATCATGAATGACATCTTCAATGCACATTCAAAATTCTGC

ATTGTTTACATTGACGATGTGCTTATTTTTTCACATTCCATAGATCAACATTTCAAACAT

CTACATACATTCTTTCATACTGCAAAACAAAATGGTTTAGTTGTTTCTAAAACAAAAATT

TCTCTATTTCAAACCCGTGTCCGTTTCCTAGGCCATTATATCTGCCAAGGTACTGTAACA

CCAATAGAGAGATCTTTAACCTTTACAAATAAATTTCCTGATAAGATCACTGATAAAACC

CAATTGCAAAGGTTTTTAGGCAGTCTTAATTATGTTCTGGACTACTATCCCAATATTAGT

CGTCTAGCCAAACCTTTACATGACAGGTTAAAAACCAACCCCATTCCTTGGTCTGACCTC

CATACCAACCTAGTCAAACAGATTAAGAAACAGGTCCAAACCATTCCTCTCCTCCATTTA

GCTAACCCGTTAGCTCCTAAGATAGTCGAGACCGATGCCTCTGACCTAGGATATGGTGGG

ATTCTTAAACAAGTACAAGATAACAAAGAACAAATACTCCAGTACACATCCGCCCACTGG

AATGATTGTCAAAAAAATTACTCTACTATAAAAAAGGAGATCCTTTCAATTGTTCTTTGT

ATTACAAAATTTCAAAGTGATTTATTAAATCAAAAATTTCTTTTACGTGTTGACTGCAAA

TCTGCAAAAGAAGTTTTACAAAAAGATGTCCAAAATCTTGCTTCAAAACAGATTTTTGCC

CGATGGCAAGCTATTTTAAGCATTTTTTATTTCGATATTGTCTACATTAAAGGAGATTCA

AATTCCATCCCTGATTTCCTAACCCGAGAATTCCTTCAAAACAGGTCCTGATGCCTCCTA

AAGCAAAAGCCAAAGACAAGGGGAAAGCCCCTCAAACCCAGCCTACAAAACCAGAAAGCC

CCAAGCCTTCTGCAAAACTCATGTCTTCAGCCATCCTAGCTGCCAAACCTATCAAATCCT

GGTATGAGGCAGTTATTGAAGAAGAAGAAACTACAAAACCACCACAGGTACAAACAGACG

CAGTTCAACATTGGGTTGACACTATATCCAAATCACCAGAATTACTCCTGGCATTACAAA

AAGTGTCCCAACAGGCCTCCAGTGACTCCGTATCTCAAACAGGTAGTATTTCCAAATCCC

TTTCAAAACCCCAAGGAGGCATTTCTGCTTCAAAACCCCTCCTTTCTTTACAACAAACCA

GTTTTCCAAAAACCAAATCCAAATATATTTTCAAAACTACTTTCCAAAATATTTTAACTA

TGGAAGAAGGATTTTATCATGAAAACCCTTCCATTGCTGCTTCAAAAATTTTCCCTCCAA

ATTGGCACTACAAACCCTGGAATTTAGCCAAACCACAATCCTACTATGCCGCCATCCTTG

AGATTACCAATTCTGTCAAGTTCAAACACTTCAAACTACATTCTGATCACACTGAACCGG

CATATTCTACATGCATCATTCACAAAGTTATCCACCCTAAAGATTGGGGACAACCCTTAC

ATCAACCTCTCTCCTTCCCCATACACTTCCGTACAAACCCCCAGGACTTCAATACTTCCT

ATACATATTGGGATTACCAACAGGCATGGTTCAATGCCTTCCTCCTACAAAATCAAAACC

ATAGCCATTCTTGGCTTTTCTACTTCCATAGTGCCATGAATACTACAAACCTCCCCCTCT

GGTTCCTCCAATGGTGGGACTACTATGGTTGTCATGTTGACTACCTCAAAGAACATCCTC

TGGTTGAAAATGGTTACCTCCATTTCAAAAATAATTTTCAACCAGCTCCTTCTGAAAGGA

AGTTTTCTTCCCTTCTCATTTTCTGTACAAAATTTTTTGTTCCATGGGTATGTTCATGGT

TCTATGATTATAATCTACAAAACGGACATCCAGTACTTGTTCGCAAATTCAAAATCAAAT

GGTGGGATTCCTTTGCAGCTGAGTCCAAAAGTTCCAAATTAGCTGTTACGGAATGGTTAC

AAAAACATCAAACAGCTCCCATCATTGATCATCATCCTCAATCAAAATTTCTAGCCCGAA

AGGCTCAAACGGCAGCTCTCCTTGCCTCTGCTAGAACTGAGGAAGAATACCTACAAATAG

TACAAGGCATTATACAAAGCCAAGACCCGAAAACGGTCTTGTCCCAATCCAGCTCATCTG

GCTCGACATCTCCTGCCATTTCCCTTGGAGACGATAATGAAGATGACTGCTTTGGTATCC

TGCCACCTATCAAGCGACATTAAAAAAAAAAAGTATTTCAGGCTTTCTCAGGCTCATCTG

TTTAGAGCCCATATCTTTTCGACTCAACTGTTTTGAGTCCCTTTATTTTTAAGCCCATTT

GTTTTAGGCCTTTTTCTACAAATATTACCTGCTACTACAAACAGACAAATCAAGTGTCGG

TGGCAAGAAGACAACAAATGAAAGTCCCACAAAAAACATGGGCAATTTCCATTTTGTCGT

CACCCAATCATGGCAGGCAATACCACATGGGTTCACATGGGTTCTGAAGGAATCTCCAAG

CTTTTCAACAAGACGTTTTGGCCTCTTCCATGCCAACTCGTCTATAAAAGGAGACTCCTA

AAACCTTAGAAGACAGACTAATTTTTTCCCCAGAAAATTTTACTCTCCCCTGTAACTCAT

ATCTGTAACCCATCTCCATTCTCCTCTGTAACCAATATCTCCTGTATTAATTTTCGTTAC

AAATTTAAGTAAGATTATATTATTTTTATTTACATTCTTGTTATATTTTTATTTGTACAT

TTAAGTATGCTCTGTGTTTGATCAAGGATCCTGACATTGTTGGTCTTGCCTTGTTCATTC

GCATCAGAAATCTGTTTATATTCTTATTTTATCTTCTTGTTCTTTTCATAAAATCAGTAT

ATAGGCTGGAAACCTGTGACCCGATCTTTTAGATCATTCTCAGGTATAACAACGCCACGT

ACTGAA

>RcomV_sc1 [endogenous-virus-name=Ricinus communis virus] [strain=sequence cluster 1] [host=Ricinus communis cv. Hale] [moltype=genomic DNA] 7867 bp

TGGTATCAGAGCCTATTGGGTTCCGGGAAAGAAACATCATCTGCATAAAGAAGATTTTAA

ACTCATTTTAATCTGCATCATATCACTGATAAACAAACATTGTTCATTTATGCACAGTAG

TAAGTCCCGTCTGGTGTAAGGGCAGTAAAACTGGTGGCTTGGTTCATGTTTGTTTAGAAA

GTTATTTTGGTTTATTTTAAAATGAATTTAGAACCATTTTTCTGTAGATCTTCTTCTTTC

TCAAACTCTTCTACCTCTGAGTCTTCAAGGTACAAAGGTTTAGTAAATAGTGAAGAAATC

ACTATTGAAGATTTTTCAAAGCATATTGATGATTGGGAAATACCCAAAGTTCAAAAGAAA

CAAATTTATGAGTTTTCAAAGTTTCCTCTCTTCAAAACTGATTTTACTATCAAAACTGAA

GAAAGAGATATTCAGATTTCAAAGCCTTTTGAAGAAATTTATCTTTTAAATCCAAAGACC

CTCCAGAAGCATAAAGAAAAAGACTATAAGTATATCCATATAGGCCTTGTCCAGGTAGGA

ATAAAGCCCCTTACCAGAGAAGGTTTAGATACTTCCATCTTAGCAGTCCTTAGAGATGCT

AGGTTTACAAATTTTTATGATTCTTTATTAGGTACTGTTGAGTCGAGCCTCAATAAAGGA

CCAATTTCTTTCAATTGTTTTCCAAACATTACAATTTCATTAAATGATAAAAATATTTTA

AAGAGCATTGTTCTTCAAATAAAGACACATAATTACAAAATGCTTGAAGGATCTATTCCG

ATAGCATTGATTTTCAAAATACATTACAAAGCCATGATTTCTGCTTTTGGTTCAAAACAC

AAGCTCCATTCACCAAAAGGAGAAACCTTATTCCTACAAACAGATCTCTCCAAATCCAAT

GCAACTATTCCAAAAACCATTCAATGGAAAGATATCACTCTTCCAGAAGAATGGATCCTT

GAAGGTGCAACCCAACCTGAATCACCAAAGCAAACAGAACCAAATACAAATCTCAAAAAT

ATAACCCAGTTTCCAGATGGAAAAGTAAAGCTTACATTCAACAGGAAATCAACCTCTTCT

AGATTTTCTGATGATGATTCTTCAACATCGACCATAGATATTGGAAGGGTATCAAAAATA

CCTTCTGTTATTTATGCTCCTTACAAAGAACCAACTCCATCTCAAACACAACCCAGATTT

TCAACATCTGATATACCAAGCTCAAACAATATACTACAGAATGTTGATTACCAAAGCAAT

ATCCCCAGACCCGTCTACAATGAACCAAAGAAAGAAGATGAGGATATAGAGATCGTCTCT

ACCCCCTCTTCACCATCACCATCTGCAATAACACAAAATCTAGAAGCAGAAATCAACATG

ATTTCAAAAGATTTTCAAATCGATAAAAAGCTTTTACATGAAGATTTCTATTCAAAGAAA

AACCATTCTAAAAAACTTTGGTTTTTTAAGAATTTTTTACAAGAAAAAGACAGTATTCAA

GAAAGATACTATCAATTTTGCATTCAAAACAAAGTCCAAATCAAATTTTTTGATTGGTTT

CAGATTTATTGCAAAGAAAATAGTATTCTTTATCCTTTCAAAGAAAAGCTTATTAGTCCT

GTCACTGTAAGAAACAAGGCTCCAGAGTGGAAGGTCGGAGAAAACCAAACCATCCAGTCT

GAACATCCTCCCCTTAGGAAAATAACCATTCCTTATGGAGAGAATGAAATAGAGGCTACA

CCTTACAAATTGATAGGAGAAGATCTTGAGAAAAATGTCAAGAATATTGTTCAGCAGAAC

AATTTCTCGAATACCTATCTTAACACAATAGGGAAGCAGCTTGTTAGGATAGAAAGCCAG

ATCCAAAAACCTCCTATAGTCTTCACCTCTCCAATCCCCAACACTGATCATCCAAAAGAG

ATAAGCCAATCAGAAAAGCTCAAAAATCCAGTCTTCAAACCCTACCAAATCTCAAAGCCT

AGCCAAGATCAATTTCAGAAGCAAACTGAGTTTGCTAGAGCAGTCAGAGAACAGCTTAGC

AAATTAGCAACTTCTGAGTCTTCCAAAAACCTAGTGCCAGATACTCCTCAAAGCAGTAGG

AATATCAGTGCTATAGATCAAGCCCAATGCGATGAATCCAATGATTCAGAGCTTGAAAGT

GTCACTGAAAAACCCTCCAACATCAACAGATTGGGATGGCAAGCCCCTAGATTGACCTGG

CATACTCCTAGGAATACTGCCCCAGATCTAGGAATAGAAAATAGGAATAATATCCTAACC

CAATCTAGATTCAATGCCTCCTCCGTCTATGAATGGAATATAGATGGAATGTCAGAATAC

AACATTCTTGGTCTCTTACAACAGATGACCATGGCAGCCAATGCTTACAAAACCCAAAGT

GGTACCTCTGATAGAGCCATTGCTGAACTCCTTATAGCTGGATTTTCTGGCCAGCTTAAA

GGATGGTGGGATTACCATCTCACACAAGCACAACAGCTCCAAATCCTAGGTGCCATCCAA

ACCACAATAGAAGGGACCCCCATCTTAGATGAACTAGGAAATCCAATTGAGGATGCTGTG

TCAACCTTAATCCTAACGATTTCCCTCCACTTCATAGGAGACCCTTCTCTTCTAAAAGAT

AAGAATGCTGAGCTCCTTAGCAATCTAACCTGTAAAAAGCTTAGCAATTTCCAAGCCTAC

AAAGACACCTTCCTGACCAGAGTCATGCTTAGGGAAGATTCAAACCAACCTTTCTGGAAA

GAAAAATTCTTAGCTGGTTTACCTAAGATACTAGGTGAAAGAGTTAGGAATACCATAAGA

GAAGCCCATAATGGCCAGATCCCTTATGATTTCTACACTTATGGTGAATTAGTGAGCCTT

ACCCAGAAAGAAGGATTGAAGATTTGCCATGACCTTAAACTCCAAAGACAACTCAAATGG

GAAATGAAGAAGACTAGGCAAGAACTAGGTAGTTTTTGTAACCAGTTTGAGTATAATCCC

ATAGAGCCTTCCCCTACCTGCAAAGGAAAATGCAAAGAAGATTTTTCCAAATCTTTTAAG

AAAAAATGTTTTTTCAAAAATAGAAGAACTCCCAAAAACAAAGAGAATTTCTACAAAAAG

CCATCCTCTTCAAAATTTCCAAAACAAAATTCCAAAAAAGATTTTAGCAAAATTACTTGC

TATAAGTGTGGCAAAAAGGGCCATACTTCAAAATATTGCAAGTTTTCTAAAAAACTCCAT

GAGCTCCAAATAGAAGATGAAATTTTGAGCAAAATTTCAGCTCTTCTTGTTGATTCCTCT

GAATCAGAATTTTCGAATAGTGAGGAAGAGTTTCAAATTGATGAAATCAAAACCTCATCC

GATTATTCTGAATCAGATTCTGAAGATATTTCAAAGAATATTAATATGCTCACTAGGCAA

CAAGAAGCTCTTCTTGAAGCCGTCAAGCATATTAATGACCCCCAAATCCAAAAACAAGTT

TTGAAAGAAATCCTAAAAACTGAAACCCTTGTCCCCTCTTCTAGTAAGAACACTTATGAT

CTTACTATGATCTTGGATAAAGGGAAAAAGCTAAAAAACCCTCTTCCTACCGTCCAAGAA

CTTCAGAAAGAAATCAAAGCCATCAAAACAGAGCTCAAAGATTTGAAAGAAAAACAACAA

AATGATTTTGTTTTACTCCAAAGCCTGATTCTCAAACAGAATAGTGATTCTGATTCTGAA

GAAGAAAATGATTTTCAAAATCTTGAGGTTTCTGAAAATGTTCCAGAAAACTTCATTAAC

GTTTTAAAAGAAATTACCTCAAGAAAATATTTGATTCAAATAAAACTCATTTTCCCTGGT

GATTTCCAAATAGAAACTATCGCTTTATTTGATACAGGAGCAGACCTCAACTGTATCAAT

TATGAGCTGGTTCCTAAAAGGTATCATCAAGAAACTAAAGAAAGGCTTACTTCAGCCAAT

AGTTCAAAGATTAGGATTGCGGGAAAAACTGAAGCTGCAATTCTAAATAATAATATTGCT

TTAAAAAATATTTTTATTCTTACAAAAGATCTTCAGCAAACAGTTATTTTGGGAACTCCT

TTTATCAATTTAATTACTCCTTTCAAAGTAACTACTGCTGGTATTATTTTTAAAGCTAAA

GATTCAAAGATTGTTTTTCCTTTTATAGAAAAACCCAAAAAGAGAAATCTCAATCTTATT

AAAGCCCACTCAATTTATAATTTTGAGATAAATGCTTTGATCAAAGGAAAACAAACCCAG

CTTTTCCACTTGAAACAAGATATGGGTTTGAAAAGGATCCAAAGTCAAATGCAAAATGAT

TTTGTCCAAAGAAAAATCTCTGATTTGCAAAAGAAGATTGAAAATGAAGTTTGTTCTGAT

CTTCCTAATGCTTTTTGGAGACGAAAACAACATATGGTAGATTTACCATATGAAAAAGAT

TTTTCTGATAAACAAATCCCTACCAAAGCAAGACCCATCCAAATGAATGAAACCCTAGAA

AGCCATTGTAGAATAGAAATAAAAGATTTAGAGCAAAAAGGTTTGATTACCAAATCCAGA

TCCCCCTGGTCATGTGCAGCTTTCTATGTCAACAAAAATAGTGAGATAGAAAGAGGAACA

CCTAGGTTGGTGATTAATTACAAACCTTTGAATAAAGCCCTAAAGTGGATTAGATACCCA

ATTCCAAACAAAAAAGATCTTCTTCAAAAGCTTCATTCTGCTTTTATTTTTTCAAAGTTT

GATATGAAATCAGGATTTTGGCAAATCCAGATTCATCCAAAAGACAGGTACAAAACTGCT

TTTACAGTTCCATTTGGCCAGTACGAATGGAATGTAATGCCCTTCGGATTAAAAAATGCT

CCTTCTGAATTTCAAAAAATCATGAATGACATTTTCAATCCTTATTCAAAGTTTTGCATT

GTCTACATTGATGATGTGTTGATATTTTCAAATTCTCTAGAGCAACATTTCAAACACTTG

GAGACATTTTTCTATGTTGTTAAGAAAAATGGTTTAGTGGTTTCAAAATCCAAGATATCT

TTATTCCAAACAAAAATTAGATTTCTTGGACACTATATCTCCCGGGGAACTATCACCCCA

ATTGAAAGGTCTCTAGCCTTTACTTCAAAATTTCCAGACAAAATTTTGGAAAAAACTCAA

TTACAAAGATTCCTTGGTAGTTTAAATTATGTAATGGATTTTTATCCAAATTTGAACTGT

CTAGCAAAGCCCCTCCATGATAGGTTAAAGAAAAACCCCCCTGCATGGACCGATGAGCAT

ACAAAGATTGTCCAAAGCATAAAAAAGCAAGTTTCTGAAATACCATGTTTACACCTAGCT

GATCCATCTGCATTCAAAATTGTTGAAACTGATGCATCAAATCTAGGATATGGTGGGATT

CTTAAGCAAGTCCAAAACAACAAAGAATGTATTGTCCAGTTTACTTCTGCACACTGGAAT

GATACACAAAAAAACTATTCAACTATCAAAAAAGAAATTCTTTCGATAGTTTTGTGCATT

TCAAAATTTCAAAGCGATCTTTTAAACCAAAAATTTCTTTTAAGGATTGATTGTAAATCT

GCTAAAGAAGTTTTACAAAAAGATGTTCAAAACATAGCTTCAAAACAGATTTTTGCCAGA

TGGCAAGCCATTCTTTCTGTTTTTGATTTTGAAATCGAATACATAAGAGGAGATTCGAAT

TCAATTCCAGACTTTCTAACCAGAGAGTTCCTTCAAAAACAGGAGTGAAAATGCCAAGAA

AATCAAAAGCAAAAGAAGAAAAGTCGACAGCAAGTTCAAAGCCTGTCCAAAGTCCAAAGA

AGGAAATTTTACCCTCTTCTTCAAAGCCTATCAGAACCTGGACCGAAATCATCCAGGAAG

AAATTGACAAAAGCAAGGTCGATCCGGCCCAACAACAATTGCAAATTCAGGAGTGGCTTG

CACAGCAAGATCCTGAATTCCTAAAAAGGGTCGAAGAATATTCAAAGCAATTGATACAAC

TTCAACCCGAAGCCTCTCCAAAGTCATCCTCCTCTTCCAACAAGGGTAAAGGTATACTTT

CTATCCCTTTTTCAAAACCTGAGATAGAAATTTTCCCTCAAAACCTATCTCAGAGCTCTA

AAAGTATTTCAAAGCCCAACTCTCAAGAAATAGTTTTGTCACAAACAAAACTTTTCAAAT

CCAATGAGTATATAGAAAAGCAAAATTTTCAAAGCATTTTGACTATTGAGGAAGGATTCT

GTACAGAAAGCCCCTCAAAACTGATTTCAAAGATTTTCCCTCCAGGATGGTATTTCAAGC

CCTGGAATATTTCAAAGCCTCAATCCTACTACCAGTCTATCCTTGAGGTAACTGGTTCTG

CAAAGTTCAAACACTTCAAAAAACACAAAGACCATAAAGACCCTGCATATTCCACATGCA

CCATACAAAGAATCCTCCACCCTACAGATTGGGGAATGGATTTACACTCATCAAGATCTT

TTCCAACTTCCCTCCAAAGAACCTACCCATCAAGCCTAAACACTTCTTTCAACTATTGGG

ATTACCAACAAGCTTGGTTTAATACCTTTCTTCTTCAAAACGAAGGACAAAGCCATTCTT

GGCTTTTCTACTTCGATACAAGCTCAATCCAAACTTCAAAGATCCCCTATTGGTTTAAAC

AATGGTGGAATTTTTATGGCAATGTTCCTGAGACCATAATTCCAGAAGTTCTACCTCTGT

TTAACCTTTTCAAAGCCCACTATAAACCAAACAAAATAGAAAGACGTTTTCCCCCGTTAC

TTCTATTTTGTTCAAACTTTTTTCTCCCCTGGGTATGTGTATGGTATTTCCATTTCAATC

AAGCAGCAGATGGAGAAATCATCCTTACCCGAAGGTTCAAAGTCAAATGGTGGGATAAAT

TTAACCACCAAGAAAAATTATCACTAAAGTTGGTACAAAATTTCCTTTCAAAAAATAGCT

TCTTGCCACCTCCGAAAGAACAGACCACTTTCTTGGCACAGAAGTCCTTCATAATTCCAA

AGCTAGCGGCGGCTCAGACAGAAGAAGATTTCTTACAATTGATCAAACAATTGTCGGAAA

CCGCCTCTTCCAAAGGAAAATCAAAAGCTTCATCTTCCTCTTCCAGCACCAGCTCGGCAG

CAATAGACCTAGATGGTGACTCAAACGAAGATGACTGTTTTGGGATTTTCAAGCCCATCA

AATGACACCTCTGTAAAGCCCTTGGGTTGAAAACCAGAAATGGCAGCCCAAAATATTTAT

GTAATGGGCCAAAAGCCCAATAAAAAAAAAAAAAAAGAGTCATTTAAAAAGAAAAAAGAA

AGAAGACAAAAGACTCTTTAAAAAAGCAAAAGATTCTTTTTCAAAGCATGGCCGACAACT

TCCACAAAAGGAGTGAAGCATCTTACTCCATTATCACAAGACTACAAGAAGAAGTGGAGC

CCCTTATTCCACTAAGAAGAGCATCCAAAGCATCAAGTAGAGAATCCAAAGCCTCAAGAC

CTCTATAAATAGAGGAGTTTCCCAAAGAAGAAGGCAGACTGAAAAATACGAAGAACCTCT

TGTATTCTTCAAGTCACACAAAGAGAAATATAGTGAGAAAAGAGAGAGTATAGTGTGAGA

GTGATAGTGAGAAAAACACCTTCCTCTTATATTCAATTCCTCCTCTTGTAAGTTATATTT

CTTTTGTTTAAAGCTTTCATTTTAAAGCTTTTATTTCAAAGTTTGTATATTTGTTCAAAG

TTTGTGTTCAAAGTTTGTATCTATCAATAAAATTTATCTTCTTATCTACCATTTTTTAAG

ATTTAACAAGCGTATGCTCTGTGCTTGCCTCGTCTGAGGATCCTGCACACCAGAAACTTG

TTGATCT

>RcomV_sc2 [endogenous-virus-name=Ricinus communis virus] [strain=sequence cluster 2] [host=Ricinus communis cv. Hale] [moltype=genomic DNA] 8162 bp

TGGTATCAGAGCCAATTGGGTTCCGGGATTAGAAGCATATCTACATTATAAAGATTTTAC

ATTCATTTTAAACTGCATCAGGTTTCCTATAGGCAAGTTCTGTTCATTTTATGCGCAGTA

GTAAGACCCGACGGACAGGGCAGTAAAGCTGAGAGCTGTGGTCCAGGCTTGTCTAGAAAG

TTATTTTGGTTTATTTTAAAATGAATTTAGAACCTTTCTTCTGTAGATCTTCTTCTTTCT

CAAATTCTTCTGCCTCTGAATCATCGAGGTACAAAGGTCTAGTAAATAGTGAAGAAATCA

CTATTGAAGATTTTTCAAAGCATATAGATGATTGGGAAATACCCAAAGTCCAAAAGAAAC

AAATTTATGAGTTTTCAAAGTTCCCTCTCTTCAAGACTGATTTTACAATCAAAACTGAAG

AAAGAGACATTCAAATTTCAAAGCCTTTTGAAGAGATTTATCTCTTAAATCCAAAGACCC

TCCAAAAGCATATGGAAAAGGATTATAAGTATATCCATATAGGCCTTGTCCAGGTAGGAA

TAAAGCCCCTTACCAGAGAAGGTTTAGATACTTCCATATTAGCAGTCCTTAGGGATGCTA

GGTTTACAAATTTCTATGATTCCTTGTTAGGTACTGTTGAGTCGAGCCTCAATAAAGGAC

CTATTTCTTTCAATTGTTTTCCAAATATCACGATTTCTTTAAACGATAAAAATGTTTTAA

AGAGCATCGTTCTTCAAATAAAAACACACAATTACAAAATGCTTGAAGGATCTATTCCGA

TAGCATTAATTTTCAAAATTCATTACAAAGCTATGATTTCTGCCTTTGGTTCCAAACACA

AACTTCACTCACCAAAAGGAGAAACACTGTTCCTACAAACAGATCTATCCAAATCCAACG

CAACTATTCCAAAAACCATTCAATGGAAAGACATTACACTTCCAGAAGAATGGATCCTTG

AAGGTGCAACCCAACCTACATCTCCAAAGCAAACAGAACCAAACACAAACCTTAAACACA

TAGCCCAATACCCAGATGGAAAGGTAAAGATCACATTCAACAGAAAATCAACCTCCTCTA

GATTTTCTGATGATGGTTCTTCAACCTCGACCATAGATATTGGAAGGGTATCCAAAATAC

CTTCTGTTATCTATGCTCCTTACAAAGAACCAATTCCATCTCAAACTCAACCCAGATTTT

CAACATCTGATGTTCCAAATACACTTCAGAATGTTGATTACCAAAGCAATATCCCCAGAC

CAGTTTACACTGATCCAAAGAAAGATGACGAAGATATAGAGATAACCTCTACCCCATCTT

CACCATCACCATCAGCCATAACCCAAAACCTTGAAGCAGAGATCAACATGATTTCAAAAG

AGTTTGAAATTGACAAAAAACTCTTGCATGAAGATTTCTATTCAAAGAAAAACCATCTTA

AAAAACTTTGGTTTTTTAAGAATTTTTTAAAAGAAAAAGATAGTATTCAAGAAAGATACT

ATCAATTTTGCATTCAAAACAAAGCCCAAATCAAATTTTTTGATTGGTTTCAAATTTACT

GCAAAGAAAATGGTATCCTTTATCCCTTTAAGGATAAGACCATTAGTCCTGTCACAGTAA

GGAACAAAGCCCCTGAGTGGAAGGTAGGAGAAAACCTAACTGTCCAGTCTGAACATCCTC

CCCTTAGGAAGATAACCATTCCTTATGGAGAGAGTGAGATAGAGGCTACACCTTACAAAT

TGGTAGGCGAAGACCTAGATAGGAATGTCAAGAACATTGTTCAGCAGAACAATTTCTCAA

ATACCTGTCTAAACACTATAGGAAAGCAACTGGTTAGGATAGAAAACCAGATTCAAAAGC

CTCCTGTAGTTGTGACCTCTCCAATCCCCAATACTGGTCAACCAAAAGAGTCGAACCAGT

CAGAAAAGCTTAAAAACCCAGTTTTTAAGCCATATCAGCTTACAAAGCCAAGCCAAGACC

ATTTCCAAAAGCAAACTGAGTTTGCCAAAGCAGTCAGAGAACAGCTAAGTAGATTAGCAA

CCTCTGAGTCATCCAAAAACCTAGTACCAGATACTCCTCAAAGTAGTAGGAATATCAGTG

CTATAGGACAAGCCCAAAGCGATGAGTCTAACGATTCAGAGCTTGAAAGCCCTATTCAAA

AACCCTTCAATATCAACAGATTGGGAATCCAATCCCCTAGATTGACCTGGAATACTCCTA

GGAGTACTGCTCCAGATCTAGGAATTGAGAATAGGAACAACATCCTAACCCAGTCTAGAT

TCAATGCCTCCTCCGTCTATGAATGGAATATAGATGGAATGTCTGAGTACAATATCCTTG

GTCTCTTGCAACAGATGACCATGGCAGCCAATGCCTACAAAACCCAAAGTGGTACCTCCG

ATAGAGCCATTGCTGAGCTCCTGATAGCTGGATTTTCTGGCCAGCTTAAAGGATGGTGGG

ATTATCATCTCACTCAAGCACAGCAACTCCAAATCCTAGGTGCTATCCAAACCACAGTAG

AAGGAACTCCCATTCTAGATGAAATAGGAAATCCGATTGAGGATGCAGTGTCAACTCTAA

TCCTCACTATTTCCCTTCATTTCATAGGAGACCCTTCTCTGCTCAAAGACAAAAATGCCG

AGCTCCTTAGCAACCTTACCTGTAAGAAGCTTAGCAACTTCCAAAGTTACAAAGACACTT

TCCTAACCAGAGTCATGCTTAGGGAAGATTCTAGCCAACCTTTCTGGAAAGAAAAGTTCT

TAGCCGGTTTACCTAAGATTTTAGGTGAAAGAGTTAGGAATACCATAAGAGAAGCCCATA

ACGGTCAAATCCCTTATGATTTCTACACTTATGGTGAGTTAGTTAGTCTAACCCAGAAAG

AAGGTTTAAAGATTTGTCATGACCTTAAGCTTCAAAGACAGCTCAAATGGGAGATGAAAA

AGACTAGGCAGGAACTAGGTAGTTTCTGTAACCAGTTTGAGTACAACCCAATAGAGCCCG

TGTCTAGTTGCAAAGGGAAATGCAAAGTTGATTCTTATTCAAAATCTTTCAAAAAGAAAC

GATTTTCCAAATATAGAAAGCCTTCTAAAAGCAAGGAAAATTTCTATAAAAAACCGTCTT

CTTCCAAATTTCTTTCAAAATTTCCAAAGAAAGATTCCAAAAAAGATTTTTCAAAAATTA

CATGCTTTAAATGTGGTAAGAAAGGCCATACTTCAAAGTATTGTAAGTTTTCAAAGAGAC

TCCATGAGCTTCAAATAGAAGAAGAAGTTTTACAAAAAATTTCTGCTCTTCTAGTTGATT

CATCTGAATCAGAGTTCTCAAACAGTGAGGAAGAATTTCAAATTGATGAAATCAAAACCT

CAACTGATTGTTCTGAGACAGATTCAGAGAGCTCTTCAAAGAACATTAATATGCTCACTA

GGCAACAAGAAGCTCTTCTTGAAGCTGTAAAGCATATTAGTGATCCTCTTATTCAAAAAC

AGGTTTTAAAAGAGATTTTAAAAACTGAATCTCTGGTTCCTTCTTCTAGTAAGAATACTT

ATGATCTTACTATGATTTTGGACAAAGGGAAGAAGTTGAAAAACCCTCTTCCTACTGTTC

AGGAACTCCAGAAGGAGATTAAAACCATCAAAACAGAACTCAAAGAGTTGAAAGAAAAAC

AACAAAATGATTCTGTTTTGCTTCAAAGCCTGATCCTCAAGCAGAATAGTGATTCTGATT

CTGATGAGGAAAATGATTTTCAAAACCTTGAAGTTTCTGAAAATGTTCCTGAAAACTTTA

TCAATGTTTTAAAGGAAATTACTTCAAGAAAATATTTGATTCAAATAAAGCTTGTTTTCC

CTGGTGATTTCCAGATAGAAACCATCGCCTTGTTTGATACAGGAGCAGACCTCAATTGTA

TCAACTGCGAACTGGTTCCTAAAAGGTTTCATCAAGAAACCAAAGAAAGGCTGACTTCAG

CCAATTCTTCAAAGATTAAGATTGCTGGTAAAACTGAAGCCGCAATTCTTAATAACAATA

TTGCTTTAAAGAATGTTTTTATTCTTACAAAGGATCTTCAGCAAACTGTTATTTTAGGGA

CTCCGTTTATCAATTTGATAACTCCCTTCAAAGTGACAACTGCTGGAATCATTTTCAAAG

CTAAAGATTCAAAGATTGTTTTTCCTTTTTTAGAAAAACCAAAAAAGAGAAATTTGAATC

TTATCAAAGCACATTCTATTTACAATTTTGAAATCAATGCTTTGATCAAAGGAAAACAGA

CCCATCTTTCTCATCTTCAACAAGATGTGGGTTTGAAGAGAATCCTAGATCAACTGCAAA

ATGATTTTATTCAAAGAAAAATTTCTGATTTTCAAATGAAGATTGAGAATGAACTCTGTT

CTGATCTTCCAAATGCTTTTTGGAATCGAAAGCAACATATGGTAGATTTGCCATATGAAA

ATGATTTTTCAGATAAGCAAATCCCTACTAAAGCCAGACCCATTCAGATGAATGAGTCTT

TAGAAAGCCATTGCAGATTAGAGATTAAAGATCTTGAGTCTAAAGGTTTAATTTCAAAGT

CTAGATCACCTTGGTCTTGTGCAGCTTTCTATGTCAATAAGAATAGCGAGATAGAAAGAG

GAACTCCTAGGCTAGTGATCAACTACAAACCTTTGAACAAAGCTCTAAAGTGGATTAGGT

ATCCTATTCCCAATAAAAAAGATCTTCTTCAAAAACTGCATTCTGCATTCATCTTTTCAA

AGTTTGACATGAAATCAGGATTTTGGCAAATCCAGATTGATCCAAAGGATCGGTACAAAA

CGGCATTTACAGTTCCATTCGGTCAATATGAGTGGAATGTGATGCCTTTTGGATTAAAAA

ATGCCCCTTCTGAATTTCAAAAAATCATGAATGACATTTTTAATCCATTTTCAAAGTTCT

GCATTGTCTACATTGATGATGTTTTAATCTTTTCAAATTCCATTGAACAGCATTTTAAAC

ACTTAGAAACATTTTTCTATGTTGTTAAAAAGAATGGTTTAGTGGTTTCAAAGTCTAAGA

TATCTCTTTTTCAAACGAAAATTAGATTTCTTGGTCATTATATCTCTCGGGGTACAATTA

CTCCAATTGAGAGATCTCTTGCATTCACTTCAAAGTTTCCAGATAAAATTTTGGAAAAAA

CTCAGCTTCAAAGATTTCTTGGTAGTTTAAATTATGTCATGGATTTTTATCCTAATTTAA

ACTGTCTTGCAAAGCCCCTGCATGATAGGCTAAGGAAAAACCCCCCTCCTTGGTCCGATC

AGCATACAAAGATAGTCCAAAGCATTAAAAAACAAGTTTTGGAAATTCCTTGCCTGCATC

TAGCTGATCCTTCTGCATTTAAGATTGTAGAAACGGATGCATCAGAGTTAGGATATGGTG

GGATTCTAAAGCAAGTTCAAAATTCCAAAGAATGTATTGTCCAGTTTACTTCTGCTCACT

GGAACGATACTCAAAAGAATTATTCTACTATCAAAAAGGAAATCCTTTCGATAGTTCTCT

GTATTTCTAAATTTCAAAGCGATCTTTTAAATCAAAAATTTCTTTTAAGGATTGATTGCA

AGTCTGCAAAAGAAGTTTTACAAAAAGATGTCCAAAATATAGCTTCAAAACAGATTTTCG

CCCGTTGGCAAGCTATTCTGTCTGTTTTTGATTTTGAAATTGAATTTATTCGAGGAGAAT

CAAATTCTATTCCTGACTTTCTGACTAGAGAGTTCTTGCAAAAACAGGAGTGATCAAAAT

GCCAAGAAAATCAAAGTCCAAAGAAGAAAAGTCTACGGCTAGTTCAAAGCCGGCCCAAAT

TCAAAGCCCAGTGAAGGAAGTTCTCCCTTCAACTTCAAAGCCCATCAGGACTTGGACTGA

GATAATCCAAGAAGAAATTGATCAACCGGATCCAGTTCAACAACAAATGGATCCTTCCAA

AGCTGATCAAGCCCAACAAATTCAGGAATGGCTTGCTCAGCAAGACCCTGAATTCATTCA

AAGGATTGAAGAATACAAAAGGCAGATGGTTCAATCCCTATCCGAACCATCTTCAAAGCA

AATAATTTCAAAGACAGAAGCGACTCCAAAGTCGTCCTCCTCCTCTGCAAAGGGTAAAGG

TATACTTTCTATCCCTTTTCAAAATACCGAGATAGAGATTTTCCCTCCAAATCTATCTCA

AAGTTCAAACAGTTTTTCAAAACCAAACTCTCAGGAAATTGTTTTGACTCAATCAAAACG

TTTTAAAACAAATGAGTATTTGGAAAAACCAATTTTTCAAAATGTTTTAACAATTGAGGA

AGGATTCTGTACAGAAAGCCCCTCTAAACTGGTTTCAAAGATTTTCCCTCCAGGTTGGTT

TTTCAAACCCTGGAACACTTCAAAGCCTCAGTCTTACTACCAGTCAATCCTCGAGGTTAC

TGGTTCCGCAAAGTTTAAACATTTCAAAAAAGACAAAACCCACAAAGACCCTGCATATTC

AACATGCACAATCCAAAGAATCCTTCACCCGTCTGATTGGGGTATGGATTTACACTCTCC

TCGTTCCTTTCCAACTTCTCTAAAAAGAACCTACCCTTCAAATCTAAACACCTCCTTCTC

CTATTGGGATTACCAGCAAGCCTGGTTCAATACCTTTCTTATTCAAAATGAAGGAAGAAG

CCACTCCTGGCTATTCTACTTCGACACCAGTCTTATCCAAACCTCCAAAATCCCCTACTG

GTTCAAACAGTGGTGGAATTACTACGGTAATGTCCCTGAGACTATCATTCCAGAGGTCTT

ACCCCTGTTCAACCTCTTCAAAGCAAATTACAAACCCAACTCAATAGAAAGGCGTTTTCC

CCCGCTTCTTCTATTCTGTTCAAACTTCTTTTTGCCCTGGGTATGTGTATGGTACTTTCA

CTTCAATCTACTACCAGATGGTGAAATTACACTTATCCGAAGATTCAAAGTAAAATGGTG

GGACAAATTCAACCACCAGGACAAACTGTCCCTTAAAGCCGTTCAAAACTTCCTTACCAA

AAATAGCTTCTTGCCTCCCCCTAAAGAACAAACAACGTTCTTGGCACAGAAGTCATTTCT

CCTTCCAAAGCTTGCTGCTGCCCAAACAGAAGAAGATTTCCTACAACTAATAAAACAGTT

GTCTGAAACAGCTTCTTCCAAAGGCAAATCCAAAGCATCATCATCATCCTCTTCCAGTGC

CAGTACTGCTGCATTTGATTTGGATGATTCAGCTGGAGACGATTGTTATGGGATATTCAA

AATCTCATAAGGTTATCTCCTTGATAAAGCCCATGGGTTGAAAACCGGAAACGGCAGCCC

AAGGATCTTTGTAATGGGCTTAAAAGCCCATATCAAAAGCCAAAAAAGAAAAAGAAAAAG

AAAAGAAAAGAAAAGAAAAAGAAAAAGACAAAAGAAAAAAAAAAAGTCAAAAAAGCAAAA

GATTCTTTCAAAGCATGGCCGACAACTTTCTCAAAAGGTGGTTGCCATTATCACAAGACT

CCAAAGTGTGAAACATCAAACAAAGACAAGTGGAAGACAACTGGAATCATTTCTACTCCA

CTAACCAAAGCATCCAAAGCCCGTGAAAGGTATCCAAAGCTCCAAGACCTCTATATAAAG

AGGAGTCCTCCAAAGAAGAACTCAGAACTTGAAGTAGACAGCATAGAGAGATAAGCTCTT

CTGTTATCTTGTTCTCACAAGAAGAAATTAGAGTGAAAAGAGTGAGTAAGAGAGAGAGAA

ATCTTGTAACTCCTCTTGTAAGTCTATATTTTCTTCTGTTTCAAAGTTTTACAAAGTTGT

ATGTTTGTTTAAAGCTTTCAAAGTTGTTTATATGTCCATGTTCAAAGTCTGTTATTATTA

ATAAAGCAATTCAAAGTTTATCTTCTTTATCTATCTTCTTCTTTTTCCCCATCATCAAGC

GTATGCTCTGTGCTTGCCTCGTCTGAGGATCCTGCACATCAGAAACTTGCTGATCTTTAT

CTTCACTGCTTAATATTTTCTTCTGTTTTCTAGATCTTGTTAGGCTGGAAAACAGATTCT

GCAAGAGCATTTAAAGCTCCCACAGGCTGTGAAAGCGTGAGTCTGGTTACCAACGCCACG

AACTTAGATCTAAATCTTCACTGCTTACTATTTCCTTCTATTTTCTAGATCTCGTTCTAA

TT

>StubV_scSt1 [endogenous-virus-name=Solanum tuberosum virus] [strain=sequence cluster 1] [host=Solanum tuberosum accession 1-3 516 R44] [moltype=genomic DNA] [topology=circular] [note=complete genome] 7705 bp

TGGTATCAGAGCCATGTTGGCAGTATACGTACTTAGTATGAGCAGCTAAATACATATACT

GTCTAAGGCATGGCAAAAATTATTTTAATATAGTATTTTAATAATTTCCGACATGCTGTT

TGTATATATAAGTTTATTATCATACTTATCATGTTCCTATATATTGTTTCTATACTTGGT

GTGAGGTATTCGTTCTTTCCTATGAGTATGAGGTAACCGATTAATATCCGGTTCGTACGT

TGGTCAGCCGAATTTGTAAGGTCGCTGTAGCCAGGAGGGCGTTTAGACCTGTTGATGGTG

GTCCCGTAGCTAAGATGACTCTAGGAATAAGAACTGTTGTAAAACCAAGAGAACCTCCCT

TAGAAACTCTATTTGGATCTTTGATATGTATTTTAATATTCTTTATATACTTACTAGCAT

GTTGATTATTGTTATTATACTTATTAGAATAAACTTTGTTCTGTAAGTCCATTAGACAGT

ATTTTTAAATCATATGGAAAATAATCAAAATATTTTGAATAGTATAAATTTATCTTCACA

ATCTACTAAGATTGCTAAATTAGAAAATGATTTACAAAACTGGAGTATACCAGAAGAACC

TTTCAAAAAGATTTACCACTTAGGTAATTTTAGTTATCTTACTCGTCGTAATATTAAGAC

TTGTGAGTCCACTATTGCTATTAATAATTCTTTAGAAATTATTCGCCTTCTTAAGGATAA

TGATATAAATCGTTATAAATCTGATTATAACTATTTGCATATTGGTTTAGTGCAAGTAGC

TGTTAAGCCTCTCTTTAGAAAAGGCCTTGATATTCCTGTTTGTGTCATCCTTAGAGATGG

CAGGTTTTTGAATTTTGATGATTCTATGTTAGGAGTTTTGCAGAGTAATCTAGCTGATGG

GCCAGTTTACTTTAATTGTTATCCTAACTTCTCTGTTGACATCAATGATCCTAATGTCAT

GGATTCTTTAACTCTCAATGTCAAAACCAAAAACTTGAACAGTAAAATCAATACTCGTGA

AATAGCTATTATTTACCGAGTATATTATCGTCTTATGAAAACCCATCTTGCTCCTAAAGC

TCGCAATGAGAGTCTGAAAGGTGAGACTATGCTTATGGAAGCCAACCATGAACATAGTTC

TATCTTTGTTCCTCGTCTTCTAAAATGGAAAGATATACTCTCCAGTAATGATTGGCGTTT

TGAAAACATAACTCAACCTTTTGCGTCTCATTCTGAAAGATCTCAGATTGAAAGAGTCGT

TCAGTTTCCAGATGGGTCTATAGAACTCAAATTCTTAGACAATTCTGTTAGGAGATCTTC

CTCGAATCGAAGGTCATCCTGGTCCCCTTCTACTTCGTGTCCCTCAAGACCCGTTCCCAG

GCCACCTTCTTCTTCAAAAACCCCGGAGGAAGCAGATGATGATGAAATTATTTCTGTTGC

TGACAACAGTAAAGGTAAAGTCACAGGAGTAGACTTTTCAGGTAACGTACCCAAAGTATT

TTACCAAGATATTCCTGGCAGTCCCACTGCAAGTGAAATGGAGCCTCCTAAGGAAAAACC

ATCTTGGTTGCTTGGAATGCTTAAGACAAGTAAAACCTTTGAGCCCAATCCAAATATCTT

GCATGAACAATGGATTCATCCTGACAATCAGGTAAAAAGAAAATGGTATGTTTCTACCTA

TACTCTTAAGCAAAGAGAATCCTTTAGAGATTTGTGGATGGCTGATATGAGAAGAATCGG

TTGTGAAATAGAATTCTTCAAATGGTTTGAAATGACTGGTAGGATTGAAAATTGTGCTGA

GTCATTACAAGTTATCATTAACAAATGGTACACTACTTCTAACAAAGTTGTTGAATCTAT

CACTCCTCCTCTAGAAGGAATTAACATCCCTATTGCAGGAACAGTAATCAAAGCTTCTCC

TTTCAAAGAGAAAAGTGATAAGACAGGTGGTTTACTCACATCTGCAGATATTGACCGAGT

AGTTGAACAAAACAATTACTCCAACCAAATTCTTCATGTTATCTCTAGACAAATAGAAGA

TACTAAACCTACCATCAGTGGAAGACCAACTCCGGCTTCTACTTCATCTAGCCATAATAT

TGAAACCTATCCAGGATTCAAAATCCCTGAATTTTCCAAAGAAAAATTCCCCAAACTTTC

TGATACTTTTGAAGTAACAGGAAATATCATTGAGAAAATCAATGCACAACTCAACAACTT

CAACATCTCTGTAAAAGATGATAAAACACCAAAAAAGGTTTCTACCCTTCAGGAAGAACC

TATTTCCAAACCTCAAGAGAAATCCAATCTCCTCCAAAAACTTGCTAGTAGTCGTTTTCA

TAATATGAAAAACTACCATAGCAAACCTTCTTTCCCAGACTTGCAGTATGAGGAAAATGC

CTTCTTATCCACTTCCTCTCATGAAGGAAGAAGCATCACTGAATGGAATATTGATGGCCT

AGCTGAGCATCAAGTATACAATAAACTCCATGAAATGGGTGTTGCCATTACTGCATATAA

AATGCGTGGATCGGCAGACAGAGATGCTGCTAATATGATTATTGCAGGTTTCACAGGAAT

GTTGAAACATTGGTGGGATAATTACTGCACTGATGAGATCAAGCAACTCATTATTAATGC

CACTGCAACAGAGACAGTTGTAAAAATGGAAGGAAACACACAATCCACTTCTAATGTCAT

AAGGGAAGATGCTTGTGCCACTCTTCTTTACCACATAGCTAAGCATTTTATTGGAGAACC

AAAATTATTCCAAGATAGGAGTCTCCAAATTCTGAGTAATCTAAGTTGTCCAAATTTAGA

CAACTTTATCCATTACAAACATGCCTTTCTCAGTAAAGTTATGATTCGTCCAGATTGCAA

TTTGGATTTCTGGAAAGAACGCTTCATCAGCGGTTTACCTCCTCTCTTTGCAGATAAAGT

AAGGACAAAAATCCAAGATCGCAATAATGGTAATATTCCTTATGGCAATCTTACTTATGG

TGATTTGGTAAGCACCATCAATGTTGTAGCCCTTGAACTCTGCACAGATATTAAGCTCAA

ACATCAGCTCAAGAAAGAGCAATCTTCTTCTAGAAAGGAACTTGGAAGTTTCTGCAGAGA

TTTTGGTTTTATCACTCCACCAGATAAAATCAAGAAAGACAAAAAGGAAAAATCCCATAG

GAAGAAATCTCGAAGAAGAGATGATTCTACCAGACCAAAGAAAAAGAAATCTAGGTCCAA

AAAACCTAATGATGCCAAAGTAGATGTATGTTGGACTTGTGGCAAGACTGGTCATAAAGC

CAATGAATGTCGTTCCAAAACCAAGAAGAAGAAAATCAATCTTCTTAATATGGATGAAGA

AACCAAAGGTAAGCTTCTTGCTATTCTTGATGAACCCTTTTCTGAATCTTCAGGTACATC

TGATGAATACAGTGATGACGAAGACATTGATCTTGATTATGATTCAGACGAAAGCCAATC

AGGAAAAGATTGCACCTGCACTGAAGCCTTCTGTACTTGTGACAGTACACCTCATATTCG

AGTACTTTCTGACCATTCGAAAGAAGCTCTCTTTGATGTCATTCAACACATTAATGATAA

TGAGGCTAGGAATCATTTTCTCCTAGAACTCAAAAATCTCCTCCTCAATACGGACAAACC

CAAACCCCGTCCGATAATTGAACCTTTCAGCATGAAGCAAATCATGAATCGCTCAGATAA

CCATTCTGAACCATCCATTTCAGATCTTCGCCATGAGGTCTCTAGTCTCAAAGAAGAGAT

TAGAAATATCAAATCTCGACTTTGTATCGTTGAGACTGACATCCTCACCAATCAAGTTCC

TAAAAGAACTACTTTTCAAGACCTTGAGTCTAACCAAGATTCTTCCCAAGAAAATGATGA

TAACATTGGTATTGACCTCCCCAATATCAATAATGATCACCTAGTTGAACCTTTAGTCAC

CAATACAGGTAATGACACTTCTACTTCTGCAGCACCAGGAGTAACGGTAATCTCTTCAAT

TAGACCTCAGAGTCATCATATACCTGTAAAAATAGTTGTTAGTAAACATTTTGTTATTAA

CAAAATTGCTTTGCTTGACAGTGGTGCAGATAGGAATTGCATTGTAAAAGGTATTGTTCC

TACTAAGTATTTACAAAAAAGTACTTCAAAACTATACTCCGCTACAGGAGAACCTTTGAA

AATAAATTATAAGCTTTCTAAAGCTCATATTTGCAATAATGGCATTTGTTTAACAAATGA

TTTTGTTATTACTGAAGATATCAATGAAGATATTATTCTTGGAATACCTTTTATCACTCA

GATAAAACCTTATTTTACTAATCTAGATGGTATTAATACTACTATTCTTGGAAAAGATCT

ACATTTTCCTTTTGTCAAAACCCTTTCACAAGATGAAAGTAATTTTGTAAGAGAAAACAC

TGTTTTCCGTATTAATAACCTTTCACAACATATCACCTTTTTAAAGGATGAAATTCGTGT

CAAGAAAATCGAACAGATTTTAAAAACCCCGGAGATAGTTACTAAAATAGCTAATCTTCA

GAAAAAGTTTGAAGAAGAAATATGTTCTGATTTTCCAAATGCTTTTTGGGAACGCAAAAA

ACATATTGTTGATTTACCATACATTGAAGGATTCAATGAACAGGCTATTACCACTAAGGC

AAGGCCCATTCAAATGAATCATGAAATGATGGAATTTTGTAAGAAAGAGATTGACACTCT

TTTAAAAAATAAGATTATCAGGATTTCTAAATCCCCTTGGAGTTGTTCTGCTTTCTACGT

TAATAAAAACGCGGAGAAAGAAAGAGGAGCACCAAGACTGGTAATCAATTACAAACCTTT

AAACTCCGTTTTAAAATGGATCAGGTATCCCATACCTAACAAACGAGACCTTTTGAAAAG

GACTTTTAAAGCTAATGTTTATAGCAAGTTTGATATGAAATCGGGTTTTTGGCAAATTCA

GATTTCAGAAAAAGATAAATATAAAACTGCTTTCAACGTCCCGTTTGGACAATATGAATG

GAATGTAATGCCTTTTGGGCTCAAGAATGCCCCTTCTGAATTTCAAAATATCATGAATTC

CATATTCAATGATTACTCTTATATGTCAATTGTTTATATTGATGATGTGCTAATCTTTTC

AGAGAACATTGATTCTCATTTTAAACATCTCAACACTTTCTTCAAGATTATTAAGAATAA

TGGTTTAGTTGTCAGTGCCAAGAAAATGGTACTCTTTCAAACCACCATCAGGTTTTTAGG

TCATGACCTTTATCAAGGCACTTATAAACCTATTTGCAGAGCCATAGAGTTCTCTTCTAA

GTTTCCAAATGAAATTCCTGATAAAACCCAACTTCAGAGGTTTTTAGGAAGTTTAAATTA

TGTAGCTGATTTTATTCCAAAAGTCAGACAAGTTTGTGAACCTTTATATAAAAGACTTAG

GAAAAATCCAGTTCCTTGGAGTACTGAACAAACTCAAGCTGTCATCCGTGTCAAAACTTT

AGTCCAAACTATTCCGTGTCTAGGAATCCCAAACCCAGATGCATTTATGATTGTTGAGAC

TGACGCGTCTGATGCAGGTTATGGAGGAATCCTTAAACAAAAAGTAGATCTTGAATCTAC

AGAGCAATTAGTTCGTTTTACATCCGGAATCTGGAATTCCGCCCAAAAAAACTATTCAAC

TGTTAAAAAGGAAGTTTTGTCTATAGTGCTATGTATCACTAAGTTTCAAGATGACCTTAT

CAATAAAGAATTTTTATTAAGAGTAGATTGTAAATCTGCTAAAGAAATCTTGCAAAAAGA

TGTTAAAAACCTTGTTTCAAAACAAATATTTGCTAGATGGCAAGCTCTACTATCTAGCTT

TGATTTCAAAATCGATTTTATCAAAGGAGAAGATAATTCTCTACCAGATTTTCTAACTCG

TGAATTCTTGCAGGGGAAGCATGAGGCCATTTAGACCAAACACTCCTTCCAAAGGAGACA

ACCTCCCAAAAGCAAAAATGGCTAGACCATTTATCCATCCCCCTTTAACACCAACAAAAG

CTGAAACTTCACAAAGGCCTAAACTAACCTTTCAAAACAAGTTCACAGCTCTAGCCGATT

ATCCAAGGCTACCCTGCCCTTCTCAACAAAACCTTCCAAAGCTTCCTTGTCCACCTCAGC

CAAAGATGATCAATCTTAGGCCAATAAAGCCCTTTGAACAAGGCACATCTTCCAGCTCCA

TCCAAACCAAAGAGAGCTATACCATGAAACCACCTGAGTCTTTTGCCCAAGCAGTTAACC

CTGAGTTAACCAAAACCATCCCTTCCAAGCCAATTCCAAAAGAAGAATCTTTTGAATTTA

TTGTTTCACAGGTATTGCCTATAATGGCTCTCAACAAAGAATATGGAAATGTAGATACAA

GAGTCCTAATCAGACCTTGTTACACAGATTCCAATTATGTGGATACTGACAATCCATTGA

AGACCAGAAGATTCTATGAAGCCATTCTTACTGATACGGATTCAATAGAAATTGAGCATT

CTAGAGATGCAAACAATTATATTAATTATTCAAGATTTACTATCAAGAAAATCTTGGATC

CATTCGAATGGTTTGCAGACCATTTACATACCCCCATTGCTTTGACGATGACTCACAAAC

CACAAACTTATAATTGGTATGATTATAAGGCAGCATGGATGAATTTCCTTTATTTGAGAC

CAAGACATACTTGGTTCGTAAAGTATAGTCCAAGTATTATCAAAGCACCCATCCCCAGAT

GGTTCTACGAGTGGTGGAATTTATTCGGAGGAATTGAGAAAATTCTTCCCCAACAATTTC

TCAATAGGTTTGAAGATTTCCAAACCAAAGAAGAAATTACTACTCTACCAATGCATATTA

AATTATGCAAGTATTATATTCAGAAAAGGATATCCTATATTATTTCTTGGAATTTCTCCA

AGAATGATTTTGAACGCATTCAATATTTGTGCAAACAAATTCAAGTCAAAGGTTGGATTC

CCAAACAACCAAATGCTAAAGTCCAAGAGAAGGCAAGATCCTCTACAAAGAATTTATCCA

AAGCAGCTCTCAAACAAAAATTGAAAGAAGCATTGGATAACATGGAAAAATATGATGAAC

ACCAGATCATGAAAATGATTGAAGACGCAGCTTCCACAGAAAGCAGCAGTGAAGACAATG

GAGACATGTGTGACCCCAAAGGTTTAGCCTTGGCTTACATGGATCCTGATTATGAATAGG

AAAAAGACTCTTATGGAAAAATAGAGGCAAAGCTGGAATTATTTTTCCATATAGTGGCAT

TTCACTATTCTAAATTAGCATCCATTGAGAAGCTACAAGACAAAACACTTTTGAGAAAAG

ATGTTCCATTATATCTCTTGAGTCAAAAGAGATTCCCACTCAAGTCACTTCGTCAAAGAA

GCAGATCCCAATGTTTTCCACTAAGGAAAAAGACACTCCACTACCTAGACTTTAATAAAA

GTAATAGGTCCCTCTATTATTGTCGGCAACATCTTAGCCGTCCACTTGTATGCCTAAGAT

ATAAAGGCACCTACTCCCTCTTCTATATAAGAAGACACTCTTTGATCTTTCAAAGGCATC

AGAAAATATTAGGACTTACTCCTATATTCTCAAATCCTTCTTGTAATCTATGTTTTTATT

CCCCTTGCTCGAAGCTATGCAATAA

>StubV_scSt2 [endogenous-virus-name=Solanum tuberosum virus] [strain=sequence cluster 2] [host=Solanum tuberosum accession 1-3 516 R44] [moltype=genomic DNA] [topology=circular] [note=complete genome, mutations in ORFs 1 and 2] 7543 bp

TGGTATCAGAGCCATGTTGGCAGTATACATACTTTGTATGAGCAACTAAGTATAGATACT

GTCTAAGGTAACGCAAAATTATTTTAATGTAGTATGTAATAATTTTCGACATACTATTAT

TATCTATATGTTTATTATTACATCTATCATGTTCCTATATATTATTTCTGTACATGGTGT

TGAGGTATTCTATTCTTTCCTATGAGTATGAGGTAACCGATTAATATCCGGTTCGTGCGT

TGGTCAGCCGTATTTGTATGGTTGTTGTAGCCAGGAGGGCGTGTAGACCTGTTGATGGTG

GTCCCGCAGCTAAGATGACTCTAGGAAAAGATGTTTTGTAAAACTAATAGACCCTTACAT

AGAAACCAGAAGAACCTTTTAAAAAGATTTACCAATTAGGTAATTTTAGTTGGATTACTC

GTCATAATATTAAGACTTGTGAATCTACTATTGCTATTAATAACTCTTTAGAAGTTATTC

GTTTGCTTAAAGAAAATGATATAAATCATTATAAATCTGAATATAATTATTTGCATATTG

GTTTAGTGCAAGTAGCTGTTAAGCCTCTGTTTAGAAAAGGACTTGATATTCCTGTTTGTG

TCATCCTTAGAGATGGCAGATTTTTAAATTTTGATGATTCGTTGTTAGGAGTTTTGCAGA

GTAACCTAGCTGATGGGCCAGTTTACTTTAATTGTTATCCTAACTTTTCTCTTGACATTA

ATGATCCTAATGTCATGGATTCTTTGACTCTTAACGTCAAAACCAAAAATTTGAACAGTA

AAATCAATACTCGTGAAATAGCTATTATTTACCGAGTTTACTATAGATTGATGAAAACCA

CTCTTGCCCCTAAGGCACTAGCAGAAAGTCTGAAAGGTGAGACTATGCTTATGGAAGCCA

ACCATGAACATAGTTCTATCTTTGTTCCTCGTCTCCTCAAATGGAAAGATATACTCTCCA

GTAATGATTGGCGTTTTGAAAACATAACTCAACCCTTTGCGTCTCATTCTGAAAGATCTC

AGATTGAAAGAGTCGTTCAGTTTCCCGATGGGTCAATAGAACTCAAATTTCTTGACAATT

CTATTAGGAGATCTTCTTCGTATCGAAGGTCATCCTGGTCCCATTCTACTTCGTGTCCCT

CAAGAACTGTTCCCAGGCCACCCTCTTCTTCAAAAACCCCAGAGGAAGCAGACGAAGAAA

ACGATGAAACTACTGCCGATGCAGTAGACAACAACAAAGGTAAAGTCACAGGAGTAGACT

TTTCAGGAAACATACCTAAAGTGTTCTACCAAGATATTCCTGGCAGTCCCACAGCAAGTG

AAATGGAACCACCAAAAGAAAATAAACCAGCATGGTTGCTCAAAATGATTAAGACAAGAA

AAACCTTCAAGCCCAATCCAAAAATACTACATGAACAATGGACTCATCCAGATAATCAGG

TAAAGAGAAATTGGTATGTTGCTACCTATACTCTTAAGCAAAGAGAATCCTTTAGAGATT

TGTGGATGACTGATATGAGAAGAATTGGTTGTGAAATCGAATTCTTCAAATGGTTTGAAA

TGACTGGTAAGATTGAAAATTGGACTGAGTCATTACAGGTTATCATTAATAAATGGTATA

CTACTTCAAATAAGGTTGTTGAATCTATTACTCCTCCTCTTGAAGGAATTAACATCCCTG

TTGCAGGTACAGTGATCAAAGCTTCTCCTTTTAAAGAAAAAAGTGATAAGACTAGCACTT

TTCTCACTTCTGCAGACATTGACCGAGTAGTAGAACAAAACAACTACTCAAACCAAATTC

TTCATGTTATCTCTAGACAAATAGAAGATACAAAACCCACTATTAGTAGAAGACCAACTC

CGGCTTCTACTTCATCCAACCATAATATTGAACCTAACCCAGGTTTCAAACTCCCTGAAT

TTTCCAAAGAAAAATTCCCTAAACTTTCTGACACTTTTGAAGTCACAGGAAATATCATTG

ACAAGATCAATGAACAACTCAACAATTTCAACATCTCCAAAAAAGATGGAAAAACCCCAG

ACAAGGTTTCTACCCTTCAGGAGAAATCTGTTTCAAACACTCAGGATAAAAACATCCTTC

TACAAAAGTTATCCAATGATCGTTTCCATAATAGGAAAAACTAACATAGCCGACCATCTT

TCCCAGACTTGCAGTATGAGGAAAATGCATTCTTGTCTACTTCTTCTCATGAAGGAAGAA

GCATTACTGAATGGAACATTGATGGCCTTGCAGAGCATCAAATATACAACAAGCTCCATG

AAATGGGAGTTGCCATTACCGCTTATAAAATGCGAGGTTCAGCAGATAAGGAAGCTGCAA

CTATGATTGCCGCAGGTTTCACCGGTATGATAAAACATTGGTGGGATAACTACTGTAATG

ATGAGACCAAGCACCTCATCATCAATGTGACTGCAACGGAGACAGTTGTAAAAGTTGAAG

GAGGCACCCAGTCTACTTCTCAAGTTACTAGAGAAGATGCTAGTGCCACTCTTCTTTATC

ATATCGCTAAACATTTCATTGGCGATCCAAAACTTTTCCAAGATAGGAGTCTTCAAATCC

TGAAAAATCTCAGTTGTCCTAACCTAGACAACTTTATTCACTACAAGCATGCCTTTCTCA

ATAAGGTTATGATCCGACCAGATTGTCATTTGGATTTTTGGAAAGAACGATTCATCAGCG

GACTTCCACCGCTATTTGCTGACAAAGTAAGGACCAAAATCCAGGATCGCAATGACGATC

GCATTCCTTATAGCAGTTTAACTTATGGTGATTTGGTAAGCACTATCAACATTGTAGGGC

TTGAACTTTGCACAGACATTAAGCTTAAGCATCAACTTAAGAAAGAACAGTCATCTTCTA

GGAGAGAACTTGGAAGTTTTTGTCGAGACTTTGGCTTTATCACTCCTCCTGATAAGGTAA

AAAAGGACAAAAGTGAGAAGAAATCTCACAGGAAGAAATCTCGAAGAAGAGATGATTCTA

CCAGACCTAGGAGAAAGAAATCCAAGTCCAAAAGGCCTAAAGATACCAAAACAGATGTCT

GTTGGACATGTGGTAAGACTGGCCATAAGGCTAATGAATGTCGATCCAAAACCAAGAAGA

AGAAGATCAATCTTCTTAACATAGATGAAGAAACCAAGGGTAAGCTTCTTGCTATACTTG

ATGAACCATTTTCTGAATCTTCAGGTACGTCTGATGAATACAGTGATGATGAAGATATTG

ATCTAGATTATGAATCAGATGCTAGCCAATCAAGAAAAGATTGCACCTGTACTGAAGCAT

TTTGTACTTGCGACAGTACTCCTCAAAATATCAGAGTACTTTCAGATCATTCGAAAGAAG

CTCTCTTTGATGTTATTCAGCATATAGCTGATGATGAGGCTAGGAATTGTTTCCTCCTAG

AACTCAAAAATATCATTCTCAACACGGACAAACCCAAATCCCGTCCGATTGTTGAACCTT

TCAGCATGAAACAAATCATGAGTCGCTCAGATAGCCATTCTGAACCCTCTATATCTAATC

TTCGCAATGAAGTATCAAATCTCAAGGATGAGATTAAAAATATTAAATCTCGACTAGGAA

AAGTCAAAATGGATGTTCTTACAGACCAGGTCCTCAAGAAGGCTACTCTACAGGAACCAG

ATTCAGGTCATGAATCTACACAAGATGATGAAGTTGACTTGATCAACAATGACCATTTGA

TTAAACCAAACATCACGAATACCGGTAATAATCCTTCTAGCTCAACAGCTCCAGGAGTAA

CGGTAATCTCATCAATTAGACCTCAGAGTCATCATATTCCTATAAAAATTGTTGTTAATA

AACATTTTGTTATTAACAAGGTTGCTTTACTTGACAGCGGTGCAGATCGTAACTGCATAG

TAAAAGGTATAGTTCCTACAAAGTACTTACAAAAAAGCACTTCAAAATTATACTCCGCTA

CAGGAGAACCTTTGAAAATTAACTATAAGCTTTCTAAAGCTCATATTTGCAACAATGGCA

TTTGTTTAACAAATGATTTCGTAATAACTGAAGATATCAATGAAGATATCATTCTTGGAA

TACCTTTTATCACTCAGATAAAACCTTATTTTACTGATTTAAATGGTATTAATACTAATA

TACTTGGAAAAGATTTACATTTTCCTTTTGTTAAAACCCTTTCACAAGATGAAAGCAATT

TCATAAGGGAAAATACAGTTTTCCGTATTAATAACCTTTCACAACATATCACCTTTTTAA

AGGATGAAATTCGTGTTAAGAAAATCGAACAGATTTTAAAAACCCCGGAGATGGTTACTA

AAATAGCCAATCTTCAAAAAACTTTTGAAGAAGAAATATGTTCTGATTTTCCAAATGCTT

TTTGGGAACGCAAAAAACATATTGTTGATTTACCATACATTGAAGGATTCAGTGAACAGG

CTATTACCACCAAGGCAAGGCCCATTCAGATGAATCACGAGATGATGGAATTTTGTAAGA

AAGAGATTGACACTCTTTTAAAAAATAAGATTATCAGGATTTCTAAATCACCTTGGAGTT

GTTTTGCTTTCTACGTTAATAAAAATGCGGAGAAAGAAAGAGGAGCACCAAGACTGGTAA

TCAATTACAAACCTTTAAACTCCGTTTTAAAATGGATCAGGTATCCCATACCTAACAAAC

GAGACCTTTTGAAAAGGACTTTTAAAGCTAATGTTTATAGCAAGTTTGATATGAAATCAG

GTTTTTGGCAAATTCAGATTTCTGAGAAAGATAAATACAAAACTGCTTTCAACGTCCCGT

TTGGACAGTATGAATGGAATGTAATGCCTTTTGGGCTTAAGAACGCCCCTTCGGAATTCC

AAAATATAATGAATTCCATTTTTAATAATTATTCTTATATGTCAATTGTTTATATTGATG

ATGTGCTGATTTTCTCTGAGAACATTGATTCTCATTTCAAACACCTTAATACATTCTTCA

AGGTTGTTAAACATAATGGTCTAGTTGTCAGTGCCAAAAAAATGGTGCTCTTTCAAACTA

CCATCAGATTTTTAGGTCATGATCTTTATCAAGGAACCTACAAACCCATTTGTAGAGCCA

TTGAATTCTCGTCCAAGTTTCCTAATGAAATTTCTGATAAAACCCAACTTCAGAGGTTTT

TAGGAAGTTTAAATTATGTTGCTGATTTCATTCCAAAGGTCAGACATGTTTGCGAACCTT

TGTATAAAAGACTTAGAAAAAATCCAGTTCCTTGGAGTTCTGAGCAAACCCAGGCTGTCA

TTCGTGTCAAAGCTTTGGTCCAAAATATTCCATGTCTAGGAATCCCTAACCCTGATGCGT

TTATGATTGTTGAGACTGATGCGTCTGATGCAGGTTATGGAGGAATCCTTAAGCAAAAAG

TAGATCTTGAATCTACAGAGCAACTTGTTCGTTTTACATCTGGAATCTGGAATTCCGCTC

AAAAAAATTATTCAACTGTAAAAAAGGAAGTTTTGTCTATAGTGCTATGTATCACTAAAT

TCCAAGACGACCTTATTAACAAAGAATTTTTACTAAGAGTAGATTGTAAATCTGCAAAGG

AAATTTTACAAAAAGATGTCAAAAATCTTGTTTCTAAACAAATATTTGCTAGATGGCAAG

CTCTACTATCTAGTTTTGATTTCAAAATTGATTTCATCAAAGGAGAAGACAATTCTCTAC

CAGATTTTCTAACTCGTGAATTCCTGCAGGGAAAGCATGAGGCCGTTTAGACCAAACACT

CCTTCCAAAGGAGACAACAACCTCCCCAAAGCAAAAATGGCTAGACCATTTATTCATCCT

CCTTTAACCCCAACAAAAGCTGAATCTCCACAAAGGCCTGAACTAACCTTCCAAAATAAG

TTCACAGCATTAGCCGATTATCCAAGGCTACCTTGTCCTTTGCAACAAAAGCTTCCAAAG

CTTCCTTGTCCACCTCAGCCAAAGTTGATCAACCTTAGACCAACAAAACCCTTTGATCAA

GGAACATCTTCTAGCTCTATTCAAACCAAAGCAAGTTATACTATGAAAGCACCTGAGTCT

TTCGCCCAAGCTGTTAACCCTGAGTTAACCAAAACTATCCCTTCCAAACCAAACCCAAAA

GAAGAATCTTTTGATTTTATTGTTTCACAGGTATTGCCATTAATGGCCCTTAACAAAGAA

TATGGGAGCATGGATACAGGTGTCTTGATCAGACCTTGTTACACAGATTCCAACTATGTG

GATACTGATAATCCACTAAAAACTAGAAGATTCTATGAAGCCATTCTTACTGATACAGAT

TCAATAGAAATTGAGCATTCTAGGGATATAAATAACTGATCATATTAATTATTCAAGATT

TACTATCAAGAAAATCTTGGACCCATTTGAATAGTTTGCAGACCATTTACATACTCCCAT

TGCTTTAACGATGACGCATAGGCCACAAACTTATAACTGGTATGACTACAAGGCAGCATG

GATGAATTTCCTTTATTTGAGACCAAGACATACTTGGTTTGTGAAGTATAGCCCAAGCAT

CACCAAAGCAACCATCCCAAGATGGTTCTACGAGTGGTGGAACTTATTCGGAGGAATTGA

GAAAATTCTCCCCCAACAATTTCTCAATAGGTTTGAAGATTTCCAAACCAAAGAAGAAAT

TACTACCCTACCGGCGCATATTAAATTATGCAAATATTATATTCAGAAAAGGATATCTTA

TATTATTTCTTGGAATTTCTCCAAGAATGATTTTGAACGTATTCAATATTTGTGTAAACA

AATTCAAGTCAAAGGTTGGATTCCCAAACAACCAAATGTTAAAGTCCAGTCAAAGACAAG

GCAAGATCATCTACAAAGAGTTCATCCAAAGCAGCTCTCAAACAAAAATTAAAAGAAGCA

TTGGACAACATAGAAAAATATGATGAAAACCAGATCATGAAAATGATTGAAGATGCAGCT

TCCACAGAAAGCAGCAGTGAAGACAACGGTGACATGTGTAATCCCAAAGGTTTAGCTTTG

GCTTACATGGACCCTGATTATGAATAGGAAAAAGACCCTTATGGAAAAATAGAGGCAAAG

CCGGAATTATTTTTCCATATAGTGGCATTTCACTATTCTAAATTAGCATCCAAAGAGAAG

CTACAAGACAAAACACTTTTGAGAAAAGATGTTTCATTATATCTCTTTAGTCAAAAAGAG

ATTCCCAATCAGTCACTTTTGAAAAAGAAGTAAACCCTAATGTTTTCCACTATGAGAAAA

TTATTCTCCACTTCTACTTTAACAAAAGAAGTAGACCCCTCCTTTTGTCGGTAACATCCA

AGCCTTACACTTGTATGCCTAAGAAATAAAGGCAACCTCTTCTTCTCCTATATAAGGAGA

CTTTGATTTACACTTAGAGGCATCAGAAAATTTCCAATATTATTCTGAAAACCTCTCTCT

CTCTCTATTCCCTCTTGTAAGCTATGTTTGAAGATATTCAATAAACGTCAGTTTCTTGTA

AGTTATTTCTATTTTAATTCCGCATTACTTAAGATCCTTCTTA

>StubV_scSt3 [endogenous-virus-name=Solanum tuberosum virus] [strain=sequence cluster 3] [host=Solanum tuberosum accession 1-3 516 R44] [moltype=genomic DNA] [topology=circular] [note=incomplete at 3end, mutations in ORFs 1 and 2] 6827 bp

TGGTATCAGAGCCATGTTGGCAGTATACGTACTTAGCATGAGCAGCTAAGCACATATATT

GTCTAAGGCATGACAAAATTATTTTAATTAGTATTTTGATAATATACTTTAAGATGTTAG

TATATATACGATTATTATTTCATCTATCATGTTCCTATATATTGTTTCTATATCAGGTGT

GAGGTATTCGTTCTTTCCTAAGTGTGTGAGGTAACCAAATAATACCCGGTGCATACGTTG

GCTAGCCCGATTGTAAGGTCGTTGAAGCCAGGTGGGCGTTTAGACCTGTTAATGGTGATC

ACGTAGCCAAGATAACTCTAGGAAAAGAACTGATGTAAAACCAATAGACCCTCCCTTAGA

AACTCTATTTGGATCTTTGATATGTGTTTTAATACTCCGTACATAATTACATACTTATTA

TATTATTATTGTTATACTTGTTGAAATATTTGCACTGTAAGCCCTTAGGACAATATTCTC

ATTTTATGGAAAATAATCAACATATTTTAAATAGTGTTAATTTATCTTCTCAAGCAACTA

AGATTGCTAAACTTGAAAATGATCTTCAAAACTGGAATATACCAGAAGAACCTTTTAAAA

AGATTTACCATTTAGGTAATTTCAGTTTAATTACTAGACATAATATTAAGACCTGTTAGT

CTACTATTTCTATTAATAATTCTTTAGAAATTATTCTTCTGCTTAAAGATAGTACTATTA

ATCATCATAGGACTGATTTTAACTATCTGCATATTGATTTGGTGGAAGTGGCTATTAAGC

CCCTTTTTCGTAAAGGCCTTCATATTGCTGTTTGTGTCCTCCTTCGAGATGACCGTTTTC

TTAATTTTGATGATTCTTTGTTATGAGTCCTCCAAAGTAACCTCGCTGATGGCCCGATAT

ACTTCAATTGTTATCCTAACTTTTCAGTTGACATTAATGACCCTAATGTCATAGACTCCT

TGACACTCAATGTCAAAACTAAGAACTTGAATAGTAAAGCCAATACTCGTGTGTAGCGAT

TATCTATCAAGTGTATTATAGGCTTATGAAAACCACTCTTGCACCAAAGGCTCGTACTGA

GAGCCAGAAAGGTGTAACTACGCTTATGGAAGCCAACCATGAACATAGTACTATCTTTGT

CCCTCGACTTCTCAATTGGAACGATGTTCTTTCCAGCAATGATTGGCATTTTGAATCCAT

AACCCAGCCTTTCTCAACTCACTCTGAGAGATCTCAAATTGAAAGAGTCATTCAGTTCCC

AAACCATCTTCTTCAAAAACCCCGGAGGAAGCGGAAACGGAAGCTACTGAAGCTATTTCT

GTTGATAACAACAAAGGAAAAGTTACCAGAGTAGACTTTTCGGGAGATATCCCTAAAGTC

TTTTATGAAGATCTCCCTACCAGCCCGACTGCAAGTGAGATGGACCCATTCAGTTGAAAA

AACCATGATGGATCACGATGCTCAAGACAGGTGATACTTTCAATCCTGATCAAGGTATCC

TTCACAAACTTTGGACTCATCCAGATAACCAGACAAAGAGAAAATGGTATGTTGCTACCT

ATACTCTTCAACAAAGAGAAGCCTTCAGAGATTTATGGATGGCTAACATGAGAAAAATCG

GCTATGAGATCGAATTCTTCAAATGGTTTGAGATGACAGGGAAGATCGAAAATCAAATCG

AATCCCTTCAAATGATCATCAATAAATGATATACGACTTCTAACAAGGTTGTTGAATCTA

TCACTCATCCCCTTGAAGGATTCAATATCCCTGTAGCAGGAACCATCATCAAAGCTTCTC

CTTTCAAAGAAAAGTGTTATAAGCCTTCCTCTTTCGTCATCCCAGCTGACATAGATCGAG

TAGTAGAACAAAACAACTACTCTAACCAAATTATTCATGTCATTTCGAAACAAATAGAAG

ATTCTAAACCATTCATTAGTAGGAGACCTACTCCATCTTCTACTTCATCCAGTTGGAACA

TTGAACCAAACCCAGGATTCAAACTTACTGAGTTCTCCAGGGAAAAAATCCCTAAATTCA

AAGATACATTTGAAATTGCCGGAAATGTCATTGACAAGGTCAATGAACAACTTTCAAACT

TCAATATTTCCACCAAAGATGGAAAAACCAGAAGACTGTCTCTACCCTTCAGGAAAAAGA

CTCTCTCATTCATAAACTCTCAAATGATCATTTTCATAATGTGAAAAAATATCATAATCG

TCATTCTTTCCCAAACCTTCAGTATGACGAAAATGCTTTTCTATCCACTTCTTCTCACGA

AGGAAGAGGAATCACTGAATGGAATATGGATGGCCTAGCTGAGCATCAAATCTACAACAA

GCTCCATGAAAAGGGAGTTGCTATTACAACATACAAGATTAGGAGCTCTACTGACAAAGA

AACTGCTATCATGATTGCTGCAGGTTTCACTGGCATGTTAAAACATTGGTGGAATAAGTA

TTGCAAAGATGAAACCAAGCACCTCATCTACAATGCTACTGCGACATAGACCGTAGTCAA

AATTGAAGGAACCGCAAAGACCACTTCTCAAGTTACTAGAGAAGATGCTTGTGCCACACT

TCTCTATCATATCGCTAAGCATTTCATTGGCCAGCCCAAACTCTTTCATGATATGAGTCT

CCAAATCCTTAACAACCTCAGTTGTCCTTCCCTAGGCAACTTCATTAGATACAAACATGA

ATTTCTCAGTAAGGTTATGATCCGTTTATACTGTCATTTGGATTTCTGGAAAGAACGTTT

CATAAGCGGTCTTCCTCCTCTCTTTGCTGACAAAGTCATAAGCAAAATCTAGGATCGCAA

TGATGGTCGTATCCTTTACAGTCAATTGACCTACGGAGACCTCGTCAGCACCATCAACAT

TGTAGGGCTTGAACTATGCACAGATATCAAACTCAAGCATCAGTTTAAGAAAGAACAATC

TTTTTCTGGAAGAGAATTGGGAAGTTTTTGTCAAGACTTTGGCTTTATTACTCCTCCTGA

TCGAGTAAGGAAAGACAAGAAGCCTCACCGATCCAGATCTCAAAGAAGAGACTCTAAAAA

ACCTAGGAAAAAGAAATCCAGGTCCAAGAGGCCCAGAGATTCCAAGCAAGATGTTTGTTG

GACATGCAACAAGACAGGCCACAAATCTAGCGAATGTCGATCCAATACCAAGAAGAAGAA

GATCAGCCTCCTTGGCATTGATGAAGACACCAAGGATAAACTCCTTTCTATTCTTGATGA

ACCTTTTTCTGACTCTTCACACTCTTCTAGTGAATCCAGTGATGATGAAGATATCAACCT

AGATTATGAATCAGATAACAGCCAATCCGGTAGAGATTGCAATTGCACTGAAGCCTTTTG

TACCTGCGACAGTACTCCACAAAAAATCAGAGTCCTCTCTAATGACTCTAAAGAAGCCCT

CTTCGATGTCATCCAACACATCAATGATGAAGAAGCCAAAAAATGTTTTCTTCTTGAACT

TAAACATCTTACTCTCAACACTGACAAGCCTAAACCCCGGCAAGTTGTTGAACCTTTCAG

CATGAAGCAAATCATGAATCGCGTAGACAATTAGTCTGAACCTTCTATTTCAGATCTCCG

ACATGAGGTTTCTAGTATCGAGGAAGAAATCTGAAATATCAAATCTCGACTCGTAAAGGT

CGAAACTGATATCTTCACCGACCAGGTCCTCAAGAAGTTTTCATTATAGGAACCTGATTT

TGATCATGAATCCTCTCAAGATAATGAAGATAAGTCTATTAACAACGATCATTTAGCTGA
[truncated: 176,719 more chars]
